# Checklist of Serengeti Ecosystem Grasses

**DOI:** 10.3897/BDJ.4.e8286

**Published:** 2016-04-15

**Authors:** Emma Victoria Williams, John Elia Ntandu, Paweł Ficinski, Maria Vorontsova

**Affiliations:** ‡ Royal Botanic Gardens, Kew, London, United Kingdom; § National Herbarium of Tanzania; Tropical Pesticide Research Institute, Arusha, Tanzania

**Keywords:** Tanzania, Poaceae, Ngorongoro

## Abstract

We present the first taxonomic checklist of the Poaceae species of the Serengeti, Tanzania. A review of the literature and herbarium specimens recorded 200 species of grasses, in line with similar studies in other parts of East Africa. The checklist is supported by a total of 939 herbarium collections. Full georeferenced collection data is made available alongside a summary checklist in pdf format. More than a quarter of the species are known from a single collection highlighting the need for further research, especially concerning the rare species and their distribution.

## Introduction

The Serengeti Ecosystem Region (abbreviated as SER) is an area of 25,000 km^2^, found south of the Tanzania and Kenya border between 2° and 4° S, defined by the movement of migratory wildebeest (McNaughton 1983). The Serengeti National Park (established in 1940, 14,700 km^2^) and the Ngorongoro Conservation Area (established in 1959, 8,300 km^2^) form the majority of the SER. Both are also designated by UNESCO as World Heritage Sites and Biosphere Reserves. There are a further five smaller reserves surrounding the Serengeti National Park; Maswa in the south; Grumeti, Ikoma, and Ikorongo in the east and the Loliondo Game Controlled Area in the west (World Bank 1993). Over the border in Kenya and contiguous to the Serengeti National Park is the Masai Mara National Reserve (World Bank 1993).

**Vegetation**

The SER is part of White's Somalia-Masia vegetation type (White 1983). The Serengeti plains which range from 1600-1800 m are characterised by "short" and "tall" grasslands and woody savanna with rocky outcrops called "kopje" (Anderson and Talbot 1965, Lind and Morrison 1974, Herlocker 1975). The short grasslands in the east are dominated by *Sporobolus* and *Kyllinga* species whilst in the west the tall grasslands are dominated by *Pennisetum*, *Andropogon* and *Themeda* species (Lind and Morrison 1974). Intermediate grasslands of *Cynodon* and *Sporobolus* are found between the short and tall grassland areas. Seventeen different grassland communities have been characterised (McNaughton 1983) : six short grassland communities, three tall and eight medium. The distribution and growth of grass species in the Serengeti plains depends on a number of factors including soil depth and texture, salt concentration, degree of wind erodability, rainfall and grazing pressure ([Bibr B1632718][Bibr B2872855]).

The Ngorongoro Conservation Area is dominated by a volcano formed 5-7 million years ago with a large central caldera formed 1-2 million years ago (Anderson and Herlocker 1973). The crater reaches over 3350 m high but the crater floor is lower at 1737 m (Anderson and Herlocker 1973). The main vegetation types are grasslands, swamps, montane heath and woodland (Herlocker and Dirschl 1972). Similar to the Serengeti plains, the distribution of the Ngorongoro grasslands are influenced by soils, climate, grazing pressures and fire (Anderson and Herlocker 1973).

**Species**

The grass flora of Tanzania is documented through the Flora of Tropical East Africa (Clayton et al. 1970, Clayton et al. 1974, Clayton and Renvoize 1982). Grassland ecology in the SER has been well studied (for example Anderson and Talbot 1965, McNaughton 1983) but these studies have focused on community structure and distribution not on species composition. There are also vegetation maps for the Serengeti woody species (Herlocker 1975) and the Ngorongoro Conservation Area (Herlocker and Dirschl 1972) but full species checklists are not included. Therefore there is a requirement for a checklist of grass species to support ecological studies on the flora and fauna of the SER.

## Materials and methods

To compile the checklist specimen data were gathered from literature and herbaria sources. Specimens from the Tanzania Serengeti Ecosystem region were databased at K, NHT and at the Serengeti Wildlife Research Centre’s herbaria in 2013 and 2014. Specimens from AAU, MO and US were downloaded from GBIF on 20^th^ May 2014 (GBIF 2014). Specimen data was obtained from EA herbarium on 3^rd^ September 2014 from available digitised records. All specimens were databased using BRAHMS version 7.5 (Botanical Research and Herbarium Management System) software (University of Oxford 2014). Additional specimen literature citations were added from The Flora of Tropical East Africa (Clayton et al. 1970, Clayton et al. 1974, Clayton and Renvoize 1982).

All specimens where possible were georeferenced using the FTEA gazetteer (Polhill 1988) and the Serengeti official map (Hopcraft 2013). Species names, authorities, synonymy and global distribution were checked with the World Checklist of Selected Plant Families (Clayton et al. 2014).

Data was uploaded from BRAHMS following the Darwin Core Formats. The specimen barcode was set as the catalogue number when possible or if a barcode was not available a unique code from the BRAHMS Serengeti database was used. Each herbarium specimen duplicate is listed separately in the checklist. If the record was a literature citation only the institution code was set as "?" as it was unknown at which herbaria the specimen is located. Only commonly used synonyms were compiled and cited in the nomenclature section, full synonymy is available at the World Checklist of Selected Plant Families (Clayton et al. 2014). Places of publication for each species name are available in the checklist in the supplementary file.

## Checklists

### Checklist of Serengeti Ecosystem Grasses

#### Agrostis kilimandscharica

Mez

##### Materials

- **Type status:** Other material. **Occurrence:** catalogNumber: K000984276; recordNumber: 165; recordedBy: Frame, GW; **Taxon:** scientificName: *Agrostis kilimandscharica* Mez; kingdom: Plantae; family: Poaceae; genus: Agrostis; specificEpithet: kilimandscharica; scientificNameAuthorship: Mez; **Location:** continent: Africa; country: Tanzania; stateProvince: Arusha; county: Ngorongoro; locality: Empakai Crater; verbatimLocality: top of north -west rim; minimumElevationInMeters: 2900; decimalLatitude: -2.933333; decimalLongitude: 35.816667; **Event:** eventDate: 1973-06-12; **Record Level:** institutionCode: K; collectionCode: Herbarium; ownerInstitutionCode: K; basisOfRecord: PreservedSpecimen
- **Type status:** Other material. **Occurrence:** catalogNumber: K000984277; recordNumber: 2757; recordedBy: Chuwa, S; **Taxon:** scientificName: *Agrostis kilimandscharica* Mez; kingdom: Plantae; family: Poaceae; genus: Agrostis; specificEpithet: kilimandscharica; scientificNameAuthorship: Mez; **Location:** continent: Africa; country: Tanzania; stateProvince: Arusha; county: Ngorongoro; locality: Mokilal; verbatimLocality: east of Oldean Mountain; minimumElevationInMeters: 2347; decimalLatitude: -3.266667; decimalLongitude: 35.433333; **Event:** eventDate: 1989-05-25; **Record Level:** institutionCode: K; collectionCode: Herbarium; ownerInstitutionCode: K; basisOfRecord: PreservedSpecimen

##### Distribution

Eastern & Central Africa

#### Agrostis producta

Pilg.

##### Materials

- **Type status:** Other material. **Occurrence:** catalogNumber: 853; recordNumber: 14; recordedBy: Vesey-FitzGerald, LDEF; **Taxon:** scientificName: *Agrostis producta* Pilg.; kingdom: Plantae; family: Poaceae; genus: Agrostis; specificEpithet: producta; scientificNameAuthorship: Pilg.; **Location:** continent: Africa; country: Tanzania; stateProvince: Arusha; county: Ngorongoro; locality: Olmoti crater; decimalLatitude: -3; decimalLongitude: 35.633333; **Event:** eventDate: 1959-12-27; **Record Level:** collectionCode: Herbarium; ownerInstitutionCode: ?; basisOfRecord: PreservedSpecimen
- **Type status:** Other material. **Occurrence:** catalogNumber: K001087079; recordNumber: P3; recordedBy: Frame, GW; **Taxon:** scientificName: *Agrostis producta* Pilg.; kingdom: Plantae; family: Poaceae; genus: Agrostis; specificEpithet: producta; scientificNameAuthorship: Pilg.; **Location:** continent: Africa; country: Tanzania; stateProvince: Arusha; county: Ngorongoro; locality: Empakai Crater; verbatimLocality: Empakaai crater; minimumElevationInMeters: 2900; decimalLatitude: -2.933333; decimalLongitude: 35.816667; **Event:** eventDate: 1973-02-28; **Record Level:** institutionCode: K; collectionCode: Herbarium; ownerInstitutionCode: K; basisOfRecord: PreservedSpecimen
- **Type status:** Other material. **Occurrence:** catalogNumber: 1014; recordNumber: P3; recordedBy: Frame, GW; **Taxon:** scientificName: *Agrostis producta* Pilg.; kingdom: Plantae; family: Poaceae; genus: Agrostis; specificEpithet: producta; scientificNameAuthorship: Pilg.; **Location:** continent: Africa; country: Tanzania; stateProvince: Arusha; county: Ngorongoro; locality: Empakai Crater; verbatimLocality: Empakaai crater; minimumElevationInMeters: 2900; decimalLatitude: -2.933333; decimalLongitude: 35.816667; **Event:** eventDate: 1973-02-28; **Record Level:** institutionCode: EA; collectionCode: Herbarium; ownerInstitutionCode: EA; basisOfRecord: PreservedSpecimen

##### Distribution

Tropical Africa

#### Alloteropsis cimicina

(L.) Stapf

##### Materials

- **Type status:** Other material. **Occurrence:** catalogNumber: K001087080; recordNumber: 10197; recordedBy: Greenway, PJ; **Taxon:** scientificName: *Alloteropsis cimicina* (L.) Stapf; kingdom: Plantae; family: Poaceae; genus: Alloteropsis; specificEpithet: cimicina; scientificNameAuthorship: (L.) Stapf; **Location:** continent: Africa; country: Tanzania; stateProvince: Mara; county: Serengeti; locality: Seronera-Klein's camp; verbatimLocality: Seronera to Klein's camp, mile 55-3; minimumElevationInMeters: 1768; decimalLatitude: -1.769167; decimalLongitude: 35.3975; **Event:** eventDate: 1961-05-17; **Record Level:** institutionCode: K; collectionCode: Herbarium; ownerInstitutionCode: K; basisOfRecord: PreservedSpecimen

##### Distribution

Tropical Africa & Asia

#### Andropogon amethystinus

Steud.

Andropogon pratensis Hochst. ex Hack.

##### Materials

- **Type status:** Other material. **Occurrence:** catalogNumber: K000984025; recordNumber: P2; recordedBy: Frame, GW; **Taxon:** scientificName: *Andropogon amethystinus* Steud.; kingdom: Plantae; family: Poaceae; genus: Andropogon; specificEpithet: amethystinus; scientificNameAuthorship: Steud.; **Location:** continent: Africa; country: Tanzania; stateProvince: Arusha; county: Ngorongoro; locality: Empakai Crater; verbatimLocality: top of South rim.; minimumElevationInMeters: 2900; decimalLatitude: -2.933333; decimalLongitude: 35.816667; **Event:** eventDate: 1973-02-28; **Record Level:** institutionCode: K; collectionCode: Herbarium; ownerInstitutionCode: K; basisOfRecord: PreservedSpecimen
- **Type status:** Other material. **Occurrence:** catalogNumber: 803; recordNumber: P2; recordedBy: Frame, GW; **Taxon:** scientificName: *Andropogon amethystinus* Steud.; kingdom: Plantae; family: Poaceae; genus: Andropogon; specificEpithet: amethystinus; scientificNameAuthorship: Steud.; **Location:** continent: Africa; country: Tanzania; stateProvince: Arusha; county: Ngorongoro; locality: Empakai Crater; verbatimLocality: top of South rim.; minimumElevationInMeters: 2900; decimalLatitude: -2.933333; decimalLongitude: 35.816667; **Event:** eventDate: 1973-02-28; **Record Level:** institutionCode: EA; collectionCode: Herbarium; ownerInstitutionCode: EA; basisOfRecord: PreservedSpecimen
- **Type status:** Other material. **Occurrence:** catalogNumber: K001087082; recordNumber: 976; recordedBy: Pole Evans, IB; Erens, J; **Taxon:** scientificName: *Andropogon amethystinus* Steud.; kingdom: Plantae; family: Poaceae; genus: Andropogon; specificEpithet: amethystinus; scientificNameAuthorship: Steud.; **Location:** continent: Africa; country: Tanzania; stateProvince: Arusha; county: Ngorongoro; locality: Ngorongoro Crater; verbatimLocality: On the top ridge of the Ngorongoro crater; decimalLatitude: -3.166667; decimalLongitude: 35.583333; **Event:** eventDate: 1938-06-24; **Record Level:** institutionCode: K; collectionCode: Herbarium; ownerInstitutionCode: K; basisOfRecord: PreservedSpecimen
- **Type status:** Other material. **Occurrence:** catalogNumber: K001087083; recordNumber: 96; recordedBy: Frame, GW; **Taxon:** scientificName: *Andropogon amethystinus* Steud.; kingdom: Plantae; family: Poaceae; genus: Andropogon; specificEpithet: amethystinus; scientificNameAuthorship: Steud.; **Location:** continent: Africa; country: Tanzania; stateProvince: Arusha; county: Ngorongoro; locality: Empakai Crater; verbatimLocality: Empakaai crater, plateau between Jaeger summit and west rim of crater.; minimumElevationInMeters: 3000; decimalLatitude: -2.933333; decimalLongitude: 35.816667; **Event:** eventDate: 1973-03-15; **Record Level:** institutionCode: K; collectionCode: Herbarium; ownerInstitutionCode: K; basisOfRecord: PreservedSpecimen
- **Type status:** Other material. **Occurrence:** catalogNumber: 989; recordNumber: 96; recordedBy: Frame, GW; **Taxon:** scientificName: *Andropogon amethystinus* Steud.; kingdom: Plantae; family: Poaceae; genus: Andropogon; specificEpithet: amethystinus; scientificNameAuthorship: Steud.; **Location:** continent: Africa; country: Tanzania; stateProvince: Arusha; county: Ngorongoro; locality: Empakai Crater; verbatimLocality: Empakaai crater, plateau between Jaeger summit and west rim of crater.; minimumElevationInMeters: 3000; decimalLatitude: -2.933333; decimalLongitude: 35.816667; **Event:** eventDate: 1973-03-15; **Record Level:** institutionCode: EA; collectionCode: Herbarium; ownerInstitutionCode: EA; basisOfRecord: PreservedSpecimen
- **Type status:** Other material. **Occurrence:** catalogNumber: 990; recordNumber: 1959; recordedBy: Terry, PJ; **Taxon:** scientificName: *Andropogon amethystinus* Steud.; kingdom: Plantae; family: Poaceae; genus: Andropogon; specificEpithet: amethystinus; scientificNameAuthorship: Steud.; **Location:** continent: Africa; country: Tanzania; stateProvince: Arusha; county: Ngorongoro; locality: Empakai Crater; verbatimLocality: West of Arusha. Empakaai crater; minimumElevationInMeters: 2900; decimalLatitude: -2.933333; decimalLongitude: 35.816667; **Event:** eventDate: 1973-08-31; **Record Level:** institutionCode: EA; collectionCode: Herbarium; ownerInstitutionCode: EA; basisOfRecord: PreservedSpecimen
- **Type status:** Other material. **Occurrence:** catalogNumber: K001087081; recordNumber: P7; recordedBy: Frame, GW; **Taxon:** scientificName: *Andropogon amethystinus* Steud.; kingdom: Plantae; family: Poaceae; genus: Andropogon; specificEpithet: amethystinus; scientificNameAuthorship: Steud.; **Location:** continent: Africa; country: Tanzania; stateProvince: Arusha; county: Ngorongoro; locality: Empakai Crater; verbatimLocality: Empakaai crater, top of south rim.; minimumElevationInMeters: 2900; decimalLatitude: -2.933333; decimalLongitude: 35.816667; **Event:** eventDate: 1973-02-28; **Record Level:** institutionCode: K; collectionCode: Herbarium; ownerInstitutionCode: K; basisOfRecord: PreservedSpecimen
- **Type status:** Other material. **Occurrence:** catalogNumber: 992; recordNumber: P7; recordedBy: Frame, GW; **Taxon:** scientificName: *Andropogon amethystinus* Steud.; kingdom: Plantae; family: Poaceae; genus: Andropogon; specificEpithet: amethystinus; scientificNameAuthorship: Steud.; **Location:** continent: Africa; country: Tanzania; stateProvince: Arusha; county: Ngorongoro; locality: Empakai Crater; verbatimLocality: Empakaai crater, top of south rim.; minimumElevationInMeters: 2900; decimalLatitude: -2.933333; decimalLongitude: 35.816667; **Event:** eventDate: 1973-02-28; **Record Level:** institutionCode: EA; collectionCode: Herbarium; ownerInstitutionCode: EA; basisOfRecord: PreservedSpecimen
- **Type status:** Other material. **Occurrence:** catalogNumber: 1103; recordNumber: P2; recordedBy: Frame, GW; **Taxon:** scientificName: *Andropogon amethystinus* Steud.; kingdom: Plantae; family: Poaceae; genus: Andropogon; specificEpithet: amethystinus; scientificNameAuthorship: Steud.; **Location:** continent: Africa; country: Tanzania; stateProvince: Arusha; county: Ngorongoro; locality: Empakai Crater; verbatimLocality: top of South rim.; minimumElevationInMeters: 2900; decimalLatitude: -2.933333; decimalLongitude: 35.816667; **Event:** eventDate: 1973-02-28; **Record Level:** institutionCode: NHT; collectionCode: Herbarium; ownerInstitutionCode: NHT; basisOfRecord: PreservedSpecimen
- **Type status:** Other material. **Occurrence:** catalogNumber: 1104; recordNumber: 96; recordedBy: Frame, GW; **Taxon:** scientificName: *Andropogon amethystinus* Steud.; kingdom: Plantae; family: Poaceae; genus: Andropogon; specificEpithet: amethystinus; scientificNameAuthorship: Steud.; **Location:** continent: Africa; country: Tanzania; stateProvince: Arusha; county: Ngorongoro; locality: Empakai Crater; verbatimLocality: Empakaai crater, plateau between Jaeger summit and west rim of crater.; minimumElevationInMeters: 3000; decimalLatitude: -2.933333; decimalLongitude: 35.816667; **Event:** eventDate: 1973-03-15; **Record Level:** institutionCode: NHT; collectionCode: Herbarium; ownerInstitutionCode: NHT; basisOfRecord: PreservedSpecimen
- **Type status:** Other material. **Occurrence:** catalogNumber: 1105; recordNumber: 1959; recordedBy: Terry, PJ; **Taxon:** scientificName: *Andropogon amethystinus* Steud.; kingdom: Plantae; family: Poaceae; genus: Andropogon; specificEpithet: amethystinus; scientificNameAuthorship: Steud.; **Location:** continent: Africa; country: Tanzania; stateProvince: Arusha; county: Ngorongoro; locality: Empakai Crater; verbatimLocality: West of Arusha. Empakaai crater; minimumElevationInMeters: 2900; decimalLatitude: -2.933333; decimalLongitude: 35.816667; **Event:** eventDate: 1973-08-31; **Record Level:** institutionCode: NHT; collectionCode: Herbarium; ownerInstitutionCode: NHT; basisOfRecord: PreservedSpecimen

##### Distribution

Tropical Africa, Yemen, India & Myanmar

#### Andropogon chrysostachyus

Steud.

##### Materials

- **Type status:** Other material. **Occurrence:** catalogNumber: K000984024; recordNumber: 2746; recordedBy: Chuwa, S; **Taxon:** scientificName: *Andropogon chrysostachyus* Steud.; kingdom: Plantae; family: Poaceae; genus: Andropogon; specificEpithet: chrysostachyus; scientificNameAuthorship: Steud.; **Location:** continent: Africa; country: Tanzania; stateProvince: Arusha; county: Ngorongoro; locality: Ngorongoro Crater; verbatimLocality: Ngorongoro Crater rim; decimalLatitude: -3.166667; decimalLongitude: 35.583333; **Event:** eventDate: 1989-05-25; **Record Level:** institutionCode: K; collectionCode: Herbarium; ownerInstitutionCode: K; basisOfRecord: PreservedSpecimen

##### Distribution

Tanzania, Kenya & Ethiopia

#### Andropogon distachyos

L.

##### Materials

- **Type status:** Other material. **Occurrence:** catalogNumber: K000984035; recordNumber: 11842; recordedBy: Greenway, PJ; Kanuri; **Taxon:** scientificName: *Andropogon distachyos* L.; kingdom: Plantae; family: Poaceae; genus: Andropogon; specificEpithet: distachyos; scientificNameAuthorship: L.; **Location:** continent: Africa; country: Tanzania; stateProvince: Arusha; county: Ngorongoro; locality: Ngorongoro Crater; verbatimLocality: Ngorongoro Crater road, south side; minimumElevationInMeters: 1829; decimalLatitude: -3.166667; decimalLongitude: 35.583333; **Event:** eventDate: 1965-06-11; **Record Level:** institutionCode: K; collectionCode: Herbarium; ownerInstitutionCode: K; basisOfRecord: PreservedSpecimen
- **Type status:** Other material. **Occurrence:** catalogNumber: K000984036; recordNumber: 5977; recordedBy: Newbould, JB; **Taxon:** scientificName: *Andropogon distachyos* L.; kingdom: Plantae; family: Poaceae; genus: Andropogon; specificEpithet: distachyos; scientificNameAuthorship: L.; **Location:** continent: Africa; country: Tanzania; stateProvince: Arusha; county: Ngorongoro; locality: Ngorongoro Crater; verbatimLocality: Olairadad Southern Part of Ngorongoro Crater Wall.; minimumElevationInMeters: 2012; decimalLatitude: -3.166667; decimalLongitude: 35.583333; **Event:** eventDate: 1962-02-02; **Record Level:** institutionCode: K; collectionCode: Herbarium; ownerInstitutionCode: K; basisOfRecord: PreservedSpecimen

##### Distribution

Europe, Arabia & Africa

#### Andropogon greenwayi

Napper

##### Materials

- **Type status:** Other material. **Occurrence:** catalogNumber: K000984026; recordNumber: 10148; recordedBy: Greenway, PJ; Talbots, L; **Taxon:** scientificName: *Andropogon greenwayi* Napper; kingdom: Plantae; family: Poaceae; genus: Andropogon; specificEpithet: greenwayi; scientificNameAuthorship: Napper; **Location:** continent: Africa; country: Tanzania; stateProvince: Shinyanga; locality: Naabi Hill; verbatimLocality: S.S.W. of Naabi Hill to Lake Largaja, Serengeti; minimumElevationInMeters: 1478; decimalLatitude: -2.883333; decimalLongitude: 35.033333; **Event:** eventDate: 1961-05-07; **Record Level:** institutionCode: K; collectionCode: Herbarium; ownerInstitutionCode: K; basisOfRecord: PreservedSpecimen
- **Type status:** Other material. **Occurrence:** catalogNumber: K000984027; recordNumber: 207; recordedBy: Robson, TO; **Taxon:** scientificName: *Andropogon greenwayi* Napper; kingdom: Plantae; family: Poaceae; genus: Andropogon; specificEpithet: greenwayi; scientificNameAuthorship: Napper; **Location:** continent: Africa; country: Tanzania; stateProvince: Mara; county: Serengeti; locality: Lake Lagarja; decimalLatitude: -3; decimalLongitude: 35.033333; **Event:** eventDate: 1957-06-13; **Record Level:** institutionCode: K; collectionCode: Herbarium; ownerInstitutionCode: K; basisOfRecord: PreservedSpecimen
- **Type status:** Other material. **Occurrence:** catalogNumber: K000984028; recordNumber: 371; recordedBy: Paulo, S; **Taxon:** scientificName: *Andropogon greenwayi* Napper; kingdom: Plantae; family: Poaceae; genus: Andropogon; specificEpithet: greenwayi; scientificNameAuthorship: Napper; **Location:** continent: Africa; country: Tanzania; stateProvince: Mara; county: Serengeti; locality: Serengeti; verbatimLocality: Central Serengeti; decimalLatitude: -2.333333; decimalLongitude: 34.833333; **Event:** eventDate: 1958-04-24; **Record Level:** institutionCode: K; collectionCode: Herbarium; ownerInstitutionCode: K; basisOfRecord: PreservedSpecimen
- **Type status:** Other material. **Occurrence:** catalogNumber: K000984029; recordNumber: 312; recordedBy: Paulo, S; **Taxon:** scientificName: *Andropogon greenwayi* Napper; kingdom: Plantae; family: Poaceae; genus: Andropogon; specificEpithet: greenwayi; scientificNameAuthorship: Napper; **Location:** continent: Africa; country: Tanzania; stateProvince: Arusha; county: Ngorongoro; locality: Ngorongoro Crater; decimalLatitude: -3.166667; decimalLongitude: 35.583333; **Event:** eventDate: 1958-04-19; **Record Level:** institutionCode: K; collectionCode: Herbarium; ownerInstitutionCode: K; basisOfRecord: PreservedSpecimen
- **Type status:** Other material. **Occurrence:** catalogNumber: K000984030; recordNumber: 173; recordedBy: Robson, TO; **Taxon:** scientificName: *Andropogon greenwayi* Napper; kingdom: Plantae; family: Poaceae; genus: Andropogon; specificEpithet: greenwayi; scientificNameAuthorship: Napper; **Location:** continent: Africa; country: Tanzania; stateProvince: Arusha; county: Ngorongoro; locality: Ngorongoro; verbatimLocality: Milanja Ngorongoro highland; decimalLatitude: -3.166667; decimalLongitude: 35.45; **Event:** eventDate: 1956-05-24; **Record Level:** institutionCode: K; collectionCode: Herbarium; ownerInstitutionCode: K; basisOfRecord: PreservedSpecimen
- **Type status:** Other material. **Occurrence:** catalogNumber: K000984031; recordNumber: 10330; recordedBy: Greenway, PJ; **Taxon:** scientificName: *Andropogon greenwayi* Napper; kingdom: Plantae; family: Poaceae; genus: Andropogon; specificEpithet: greenwayi; scientificNameAuthorship: Napper; **Location:** continent: Africa; country: Tanzania; stateProvince: Mara; county: Serengeti; locality: Seronera; verbatimLocality: Seronera to Naabi Entrance, Mile 36.; minimumElevationInMeters: 1676; decimalLatitude: -2.816667; decimalLongitude: 35.2; **Event:** eventDate: 1961-05-26; **Record Level:** institutionCode: K; collectionCode: Herbarium; ownerInstitutionCode: K; basisOfRecord: PreservedSpecimen
- **Type status:** Other material. **Occurrence:** catalogNumber: K000984032; recordNumber: 2784; recordedBy: Chuwa, S; **Taxon:** scientificName: *Andropogon greenwayi* Napper; kingdom: Plantae; family: Poaceae; genus: Andropogon; specificEpithet: greenwayi; scientificNameAuthorship: Napper; **Location:** continent: Africa; country: Tanzania; stateProvince: Arusha; county: Ngorongoro; locality: Kakesio; minimumElevationInMeters: 1700; decimalLatitude: -3.316667; decimalLongitude: 35.033333; **Event:** eventDate: 1989-05-28; **Record Level:** institutionCode: K; collectionCode: Herbarium; ownerInstitutionCode: K; basisOfRecord: PreservedSpecimen
- **Type status:** Other material. **Occurrence:** catalogNumber: 292; recordNumber: 10148; recordedBy: Greenway, PJ; Talbots, L; **Taxon:** scientificName: *Andropogon greenwayi* Napper; kingdom: Plantae; family: Poaceae; genus: Andropogon; specificEpithet: greenwayi; scientificNameAuthorship: Napper; **Location:** continent: Africa; country: Tanzania; stateProvince: Shinyanga; locality: Naabi Hill; verbatimLocality: S.S.W. of Naabi Hill to Lake Largaja, Serengeti; minimumElevationInMeters: 1478; decimalLatitude: -2.883333; decimalLongitude: 35.033333; **Event:** eventDate: 1961-05-07; **Record Level:** institutionCode: EA; collectionCode: Herbarium; ownerInstitutionCode: EA; basisOfRecord: PreservedSpecimen
- **Type status:** Other material. **Occurrence:** catalogNumber: 293; recordNumber: 326; recordedBy: Kreulen, AR; **Taxon:** scientificName: *Andropogon greenwayi* Napper; kingdom: Plantae; family: Poaceae; genus: Andropogon; specificEpithet: greenwayi; scientificNameAuthorship: Napper; **Location:** continent: Africa; country: Tanzania; stateProvince: Mara; county: Serengeti; locality: Serengeti National Park; verbatimLocality: East Simiyu; decimalLatitude: -2.333333; decimalLongitude: 34.833333; **Event:** eventDate: 1974-05-10; **Record Level:** institutionCode: EA; collectionCode: Herbarium; ownerInstitutionCode: EA; basisOfRecord: PreservedSpecimen
- **Type status:** Other material. **Occurrence:** catalogNumber: 294; recordNumber: 371; recordedBy: Paulo, S; **Taxon:** scientificName: *Andropogon greenwayi* Napper; kingdom: Plantae; family: Poaceae; genus: Andropogon; specificEpithet: greenwayi; scientificNameAuthorship: Napper; **Location:** continent: Africa; country: Tanzania; stateProvince: Mara; county: Serengeti; locality: Serengeti; verbatimLocality: Central Serengeti; decimalLatitude: -2.333333; decimalLongitude: 34.833333; **Event:** eventDate: 1958-04-24; **Record Level:** institutionCode: EA; collectionCode: Herbarium; ownerInstitutionCode: EA; basisOfRecord: PreservedSpecimen
- **Type status:** Other material. **Occurrence:** catalogNumber: 295; recordNumber: 4692; recordedBy: Vesey-FitzGerald, LDEF; **Taxon:** scientificName: *Andropogon greenwayi* Napper; kingdom: Plantae; family: Poaceae; genus: Andropogon; specificEpithet: greenwayi; scientificNameAuthorship: Napper; **Location:** continent: Africa; country: Tanzania; stateProvince: Mara; county: Serengeti; locality: Serengeti National Park; verbatimLocality: Largaja plain.; minimumElevationInMeters: 1524; decimalLatitude: -2.333333; decimalLongitude: 34.833333; **Event:** eventDate: 1965-05-16; **Record Level:** institutionCode: EA; collectionCode: Herbarium; ownerInstitutionCode: EA; basisOfRecord: PreservedSpecimen
- **Type status:** Isotype. **Occurrence:** catalogNumber: K000280542; recordNumber: 10165; recordedBy: Greenway, PJ; Turner, M; **Taxon:** scientificName: *Andropogon greenwayi* Napper; kingdom: Plantae; family: Poaceae; genus: Andropogon; specificEpithet: greenwayi; scientificNameAuthorship: Napper; **Location:** continent: Africa; country: Tanzania; stateProvince: Shinyanga; locality: Naabi Hill; verbatimLocality: NW by W of Naabi Hill. locally common in the southern and south-western area of the Serengeti Plains southwards of Moru Kopjes and slightly eastwards of Naabi Hill then south to Lake Lagarja.; decimalLatitude: -2.883333; decimalLongitude: 35.033333; **Event:** eventDate: 1961-05-10; **Record Level:** institutionCode: K; collectionCode: Herbarium; ownerInstitutionCode: K; basisOfRecord: PreservedSpecimen
- **Type status:** Other material. **Occurrence:** catalogNumber: 539; recordNumber: 24307; recordedBy: Peterson, PM; Soreng, RJ; Romaschenko, K; Mbago, F; **Taxon:** scientificName: *Andropogon greenwayi* Napper; kingdom: Plantae; family: Poaceae; genus: Andropogon; specificEpithet: greenwayi; scientificNameAuthorship: Napper; **Location:** continent: Africa; country: Tanzania; stateProvince: Arusha; county: Ngorongoro; locality: Ngorongoro Crater; verbatimLocality: Ngorongoro Conservation Area, rim of Ngorongoro Crater (descent gate).; minimumElevationInMeters: 2168; decimalLatitude: -3.15462; decimalLongitude: 35.47717; **Event:** eventDate: 2012-06-19; **Record Level:** institutionCode: US; collectionCode: Herbarium; ownerInstitutionCode: US; basisOfRecord: PreservedSpecimen
- **Type status:** Holotype. **Occurrence:** catalogNumber: EA000000509; recordNumber: 10165; recordedBy: Greenway, PJ; Turner, M; **Taxon:** scientificName: *Andropogon greenwayi* Napper; kingdom: Plantae; family: Poaceae; genus: Andropogon; specificEpithet: greenwayi; scientificNameAuthorship: Napper; **Location:** continent: Africa; country: Tanzania; stateProvince: Shinyanga; locality: Naabi Hill; verbatimLocality: NW by W of Naabi Hill. locally common in the southern and south-western area of the Serengeti Plains southwards of Moru Kopjes and slightly eastwards of Naabi Hill then south to Lake Lagarja.; decimalLatitude: -2.883333; decimalLongitude: 35.033333; **Event:** eventDate: 1961-05-10; **Record Level:** institutionCode: EA; collectionCode: Herbarium; ownerInstitutionCode: EA; basisOfRecord: PreservedSpecimen
- **Type status:** Other material. **Occurrence:** catalogNumber: 771; recordNumber: 10330; recordedBy: Greenway, PJ; **Taxon:** scientificName: *Andropogon greenwayi* Napper; kingdom: Plantae; family: Poaceae; genus: Andropogon; specificEpithet: greenwayi; scientificNameAuthorship: Napper; **Location:** continent: Africa; country: Tanzania; stateProvince: Mara; county: Serengeti; locality: Seronera; verbatimLocality: Seronera to Naabi Entrance, Mile 36.; minimumElevationInMeters: 1676; decimalLatitude: -2.816667; decimalLongitude: 35.2; **Event:** eventDate: 1961-05-26; **Record Level:** institutionCode: EA; collectionCode: Herbarium; ownerInstitutionCode: EA; basisOfRecord: PreservedSpecimen
- **Type status:** Other material. **Occurrence:** catalogNumber: 773; recordNumber: 312; recordedBy: Paulo, S; **Taxon:** scientificName: *Andropogon greenwayi* Napper; kingdom: Plantae; family: Poaceae; genus: Andropogon; specificEpithet: greenwayi; scientificNameAuthorship: Napper; **Location:** continent: Africa; country: Tanzania; stateProvince: Arusha; county: Ngorongoro; locality: Ngorongoro Crater; decimalLatitude: -3.166667; decimalLongitude: 35.583333; **Event:** eventDate: 1958-04-19; **Record Level:** institutionCode: EA; collectionCode: Herbarium; ownerInstitutionCode: EA; basisOfRecord: PreservedSpecimen
- **Type status:** Other material. **Occurrence:** catalogNumber: 774; recordNumber: 173; recordedBy: Robson, TO; **Taxon:** scientificName: *Andropogon greenwayi* Napper; kingdom: Plantae; family: Poaceae; genus: Andropogon; specificEpithet: greenwayi; scientificNameAuthorship: Napper; **Location:** continent: Africa; country: Tanzania; stateProvince: Arusha; county: Ngorongoro; locality: Ngorongoro; verbatimLocality: Milanja Ngorongoro highland; decimalLatitude: -3.166667; decimalLongitude: 35.45; **Event:** eventDate: 1956-05-24; **Record Level:** institutionCode: EA; collectionCode: Herbarium; ownerInstitutionCode: EA; basisOfRecord: PreservedSpecimen
- **Type status:** Other material. **Occurrence:** catalogNumber: 775; recordNumber: 207; recordedBy: Robson, TO; **Taxon:** scientificName: *Andropogon greenwayi* Napper; kingdom: Plantae; family: Poaceae; genus: Andropogon; specificEpithet: greenwayi; scientificNameAuthorship: Napper; **Location:** continent: Africa; country: Tanzania; stateProvince: Mara; county: Serengeti; locality: Lake Lagarja; decimalLatitude: -3; decimalLongitude: 35.033333; **Event:** eventDate: 1957-06-13; **Record Level:** institutionCode: EA; collectionCode: Herbarium; ownerInstitutionCode: EA; basisOfRecord: PreservedSpecimen
- **Type status:** Other material. **Occurrence:** catalogNumber: 784; recordNumber: 6108; recordedBy: Newbould, JB; **Taxon:** scientificName: *Andropogon greenwayi* Napper; kingdom: Plantae; family: Poaceae; genus: Andropogon; specificEpithet: greenwayi; scientificNameAuthorship: Napper; **Location:** continent: Africa; country: Tanzania; stateProvince: Arusha; county: Ngorongoro; locality: Olbalbal; verbatimLocality: Olkiu. Between Malanja and Olbalbal scarp.; minimumElevationInMeters: 1829; **Event:** eventDate: 1962-06-30; **Record Level:** institutionCode: EA; collectionCode: Herbarium; ownerInstitutionCode: EA; basisOfRecord: PreservedSpecimen
- **Type status:** Other material. **Occurrence:** catalogNumber: 793; recordNumber: 259; recordedBy: Belsky, JB; **Taxon:** scientificName: *Andropogon greenwayi* Napper; kingdom: Plantae; family: Poaceae; genus: Andropogon; specificEpithet: greenwayi; scientificNameAuthorship: Napper; **Location:** continent: Africa; country: Tanzania; stateProvince: Shinyanga; locality: Naabi Hill Gate; verbatimLocality: South of Naabi gate.; decimalLatitude: -3; decimalLongitude: 35; **Event:** eventDate: 1981-05-29; **Record Level:** institutionCode: EA; collectionCode: Herbarium; ownerInstitutionCode: EA; basisOfRecord: PreservedSpecimen
- **Type status:** Isotype. **Occurrence:** catalogNumber: PRE0664079-0; recordNumber: 10165; recordedBy: Greenway, PJ; Turner, M; **Taxon:** scientificName: *Andropogon greenwayi* Napper; kingdom: Plantae; family: Poaceae; genus: Andropogon; specificEpithet: greenwayi; scientificNameAuthorship: Napper; **Location:** continent: Africa; country: Tanzania; stateProvince: Shinyanga; locality: Naabi Hill; verbatimLocality: NW by W of Naabi Hill. locally common in the southern and south-western area of the Serengeti Plains southwards of Moru Kopjes and slightly eastwards of Naabi Hill then south to Lake Lagarja.; decimalLatitude: -2.883333; decimalLongitude: 35.033333; **Event:** eventDate: 1961-05-10; **Record Level:** institutionCode: PRE; collectionCode: Herbarium; ownerInstitutionCode: PRE; basisOfRecord: PreservedSpecimen
- **Type status:** Other material. **Occurrence:** catalogNumber: 802; recordNumber: 6108; recordedBy: Newbould, JB; **Taxon:** scientificName: *Andropogon greenwayi* Napper; kingdom: Plantae; family: Poaceae; genus: Andropogon; specificEpithet: greenwayi; scientificNameAuthorship: Napper; **Location:** continent: Africa; country: Tanzania; stateProvince: Arusha; county: Ngorongoro; locality: Olbalbal; verbatimLocality: Olkiu. Between Malanja and Olbalbal scarp.; minimumElevationInMeters: 1829; **Event:** eventDate: 1962-06-30; **Record Level:** institutionCode: EA; collectionCode: Herbarium; ownerInstitutionCode: EA; basisOfRecord: PreservedSpecimen

##### Distribution

Tanzania, Kenya, Ethiopia, Somalia & Yemen

#### Andropogon lima

(Hack.) Stapf

##### Materials

- **Type status:** Other material. **Occurrence:** catalogNumber: K000984033; recordNumber: 89004T; recordedBy: Chuwa, S; **Taxon:** scientificName: *Andropogon lima* (Hack.) Stapf; kingdom: Plantae; family: Poaceae; genus: Andropogon; specificEpithet: lima; scientificNameAuthorship: (Hack.) Stapf; **Location:** continent: Africa; country: Tanzania; stateProvince: Arusha; county: Ngorongoro; locality: Oldeani Mt; minimumElevationInMeters: 3200; decimalLatitude: -3.266667; decimalLongitude: 35.433333; **Record Level:** institutionCode: K; collectionCode: Herbarium; ownerInstitutionCode: K; basisOfRecord: PreservedSpecimen
- **Type status:** Other material. **Occurrence:** catalogNumber: K000984034; recordNumber: 2674; recordedBy: Chuwa, S; **Taxon:** scientificName: *Andropogon lima* (Hack.) Stapf; kingdom: Plantae; family: Poaceae; genus: Andropogon; specificEpithet: lima; scientificNameAuthorship: (Hack.) Stapf; **Location:** continent: Africa; country: Tanzania; stateProvince: Arusha; county: Ngorongoro; locality: Lemagarut Mt.; minimumElevationInMeters: 2987; decimalLatitude: -3.166667; decimalLongitude: 35.35; **Event:** eventDate: 1988-10-24; **Record Level:** institutionCode: K; collectionCode: Herbarium; ownerInstitutionCode: K; basisOfRecord: PreservedSpecimen

##### Distribution

Tropical Africa

#### Andropogon schirensis

Hochst.

##### Materials

- **Type status:** Other material. **Occurrence:** catalogNumber: K000984037; recordNumber: 10768; recordedBy: Greenway, PJ; **Taxon:** scientificName: *Andropogon schirensis* Hochst.; kingdom: Plantae; family: Poaceae; genus: Andropogon; specificEpithet: schirensis; scientificNameAuthorship: Hochst.; **Location:** continent: Africa; country: Tanzania; stateProvince: Mara; county: Serengeti; locality: Mara River Guard Post; minimumElevationInMeters: 1280; decimalLatitude: -1.566667; decimalLongitude: 34.683333; **Event:** eventDate: 1962-08-21; **Record Level:** institutionCode: K; collectionCode: Herbarium; ownerInstitutionCode: K; basisOfRecord: PreservedSpecimen
- **Type status:** Other material. **Occurrence:** catalogNumber: K000984038; recordNumber: 12006; recordedBy: Greenway, PJ; Tanner, M; **Taxon:** scientificName: *Andropogon schirensis* Hochst.; kingdom: Plantae; family: Poaceae; genus: Andropogon; specificEpithet: schirensis; scientificNameAuthorship: Hochst.; **Location:** continent: Africa; country: Tanzania; stateProvince: Mara; county: Serengeti; locality: Wogakuria Hill; minimumElevationInMeters: 1707; decimalLatitude: -1.65; decimalLongitude: 35; **Event:** eventDate: 1964-01-30; **Record Level:** institutionCode: K; collectionCode: Herbarium; ownerInstitutionCode: K; basisOfRecord: PreservedSpecimen

##### Distribution

Tropical & Southern Africa

#### Apochiton burttii

C.E.Hubb

##### Materials

- **Type status:** Other material. **Occurrence:** catalogNumber: DB1076; recordNumber: 7266; recordedBy: Vesey-FitzGerald, LDEF; **Taxon:** scientificName: *Apochiton burttii* C.E.Hubb; kingdom: Plantae; family: Poaceae; genus: Apochiton; specificEpithet: burttii; scientificNameAuthorship: C.E.Hubb; **Location:** continent: Africa; country: Tanzania; stateProvince: Arusha; county: Ngorongoro; locality: Olduvai; verbatimLocality: Serengeti plains; minimumElevationInMeters: 1200; decimalLatitude: -2.966667; decimalLongitude: 35.366667; **Event:** eventDate: 1972-04-03; **Record Level:** institutionCode: SWRC; collectionCode: Herbarium; ownerInstitutionCode: SWRC; basisOfRecord: PreservedSpecimen
- **Type status:** Other material. **Occurrence:** catalogNumber: DB1077; recordNumber: 4487; recordedBy: Vesey-FitzGerald, LDEF; **Taxon:** scientificName: *Apochiton burttii* C.E.Hubb; kingdom: Plantae; family: Poaceae; genus: Apochiton; specificEpithet: burttii; scientificNameAuthorship: C.E.Hubb; **Location:** continent: Africa; country: Tanzania; stateProvince: Arusha; county: Ngorongoro; locality: Olduvai; verbatimLocality: Olduvai watercourse.; decimalLatitude: -2.966667; decimalLongitude: 35.366667; **Event:** eventDate: 1965-01-05; **Record Level:** institutionCode: SWRC; collectionCode: Herbarium; ownerInstitutionCode: SWRC; basisOfRecord: PreservedSpecimen
- **Type status:** Other material. **Occurrence:** catalogNumber: 540; recordNumber: 657; recordedBy: Ellemann, L; **Taxon:** scientificName: *Apochiton burttii* C.E.Hubb; kingdom: Plantae; family: Poaceae; genus: Apochiton; specificEpithet: burttii; scientificNameAuthorship: C.E.Hubb; **Location:** continent: Africa; country: Tanzania; stateProvince: Arusha; county: Ngorongoro; locality: Ololgumi; verbatimLocality: Ngorongoro Conservation Area, Ololgumi, along Kakesio river, a seasonal river.; minimumElevationInMeters: 1600; decimalLatitude: -3.333; decimalLongitude: 35.033; **Event:** eventDate: 1993-06-15; **Record Level:** institutionCode: AAU; collectionCode: Herbarium; ownerInstitutionCode: AAU; basisOfRecord: PreservedSpecimen

##### Distribution

Tanzania endemic

#### Aristida sp.

##### Materials

- **Type status:** Other material. **Occurrence:** catalogNumber: 544; recordNumber: 24279; recordedBy: Peterson, PM; Soreng, RJ; Romaschenko, K; Mbago, F; **Taxon:** scientificName: Aristida; kingdom: Plantae; family: Poaceae; genus: Aristida; **Location:** continent: Africa; country: Tanzania; stateProvince: Mara; county: Serengeti; locality: Mbuzi Mare camp; verbatimLocality: Serengeti National Park, near Mbuzi Mare camp.; minimumElevationInMeters: 1552; decimalLatitude: -2.23332; decimalLongitude: 34.96467; **Event:** eventDate: 2012-06-17; **Record Level:** institutionCode: US; collectionCode: Herbarium; ownerInstitutionCode: US; basisOfRecord: PreservedSpecimen

#### Aristida adoensis

Hochst.

##### Materials

- **Type status:** Other material. **Occurrence:** catalogNumber: 5; recordNumber: 6449; recordedBy: Newbould, JB; **Taxon:** scientificName: *Aristida adoensis* Hochst.; kingdom: Plantae; family: Poaceae; genus: Aristida; specificEpithet: adoensis; scientificNameAuthorship: Hochst.; **Location:** continent: Africa; country: Tanzania; stateProvince: Arusha; county: Ngorongoro; locality: Endulen; verbatimLocality: Ngorongoro pasture research scheme. Olgoum nr. Endulen.; decimalLatitude: -3.183333; decimalLongitude: 35.15; **Event:** eventDate: 1962-12-24; **Record Level:** institutionCode: EA; collectionCode: Herbarium; ownerInstitutionCode: EA; basisOfRecord: PreservedSpecimen
- **Type status:** Other material. **Occurrence:** catalogNumber: 6; recordNumber: 5925; recordedBy: Newbould, JB; **Taxon:** scientificName: *Aristida adoensis* Hochst.; kingdom: Plantae; family: Poaceae; genus: Aristida; specificEpithet: adoensis; scientificNameAuthorship: Hochst.; **Location:** continent: Africa; country: Tanzania; stateProvince: Arusha; county: Ngorongoro; locality: Olbalbal; verbatimLocality: Ngorongoro, Longo - Olbalbal.; minimumElevationInMeters: 1417; decimalLatitude: -3; decimalLongitude: 35.5; **Event:** eventDate: 1962-01-30; **Record Level:** institutionCode: EA; collectionCode: Herbarium; ownerInstitutionCode: EA; basisOfRecord: PreservedSpecimen
- **Type status:** Other material. **Occurrence:** catalogNumber: 7; recordNumber: R56; recordedBy: Ludanga, RI; **Taxon:** scientificName: *Aristida adoensis* Hochst.; kingdom: Plantae; family: Poaceae; genus: Aristida; specificEpithet: adoensis; scientificNameAuthorship: Hochst.; **Location:** continent: Africa; country: Tanzania; stateProvince: Mara; county: Serengeti; locality: Seronera; verbatimLocality: Seronera; minimumElevationInMeters: 1524; decimalLatitude: -2.45; decimalLongitude: 34.833333; **Event:** eventDate: 1968-01-24; **Record Level:** institutionCode: EA; collectionCode: Herbarium; ownerInstitutionCode: EA; basisOfRecord: PreservedSpecimen
- **Type status:** Other material. **Occurrence:** catalogNumber: 312; recordNumber: 363; recordedBy: Kirika, P; **Taxon:** scientificName: *Aristida adoensis* Hochst.; kingdom: Plantae; family: Poaceae; genus: Aristida; specificEpithet: adoensis; scientificNameAuthorship: Hochst.; **Location:** continent: Africa; country: Tanzania; stateProvince: Mara; county: Serengeti; locality: Serengeti National Park; decimalLatitude: -2.333333; decimalLongitude: 34.833333; **Event:** eventDate: 1905-05-11; **Record Level:** institutionCode: EA; collectionCode: Herbarium; ownerInstitutionCode: EA; basisOfRecord: PreservedSpecimen
- **Type status:** Other material. **Occurrence:** catalogNumber: 313; recordNumber: 6561; recordedBy: Kirika, P; **Taxon:** scientificName: *Aristida adoensis* Hochst.; kingdom: Plantae; family: Poaceae; genus: Aristida; specificEpithet: adoensis; scientificNameAuthorship: Hochst.; **Location:** continent: Africa; country: Tanzania; stateProvince: Shinyanga; locality: Granite Kopje; verbatimLocality: Granite Kopje 6 Miles North of Naabi, Serengeti.; minimumElevationInMeters: 1676; decimalLatitude: -2.783333; decimalLongitude: 35.333333; **Event:** eventDate: 1905-05-16; **Record Level:** institutionCode: EA; collectionCode: Herbarium; ownerInstitutionCode: EA; basisOfRecord: PreservedSpecimen
- **Type status:** Other material. **Occurrence:** catalogNumber: 314; recordNumber: 5599; recordedBy: Kirika, P; **Taxon:** scientificName: *Aristida adoensis* Hochst.; kingdom: Plantae; family: Poaceae; genus: Aristida; specificEpithet: adoensis; scientificNameAuthorship: Hochst.; **Location:** continent: Africa; country: Tanzania; stateProvince: Mara; county: Serengeti; locality: Serengeti National Park; minimumElevationInMeters: 1600; decimalLatitude: -2.333333; decimalLongitude: 34.833333; **Event:** eventDate: 1905-05-18; **Record Level:** institutionCode: EA; collectionCode: Herbarium; ownerInstitutionCode: EA; basisOfRecord: PreservedSpecimen
- **Type status:** Other material. **Occurrence:** catalogNumber: DB1081; recordNumber: 10050; recordedBy: Greenway, PJ; Tanner, M; **Taxon:** scientificName: *Aristida adoensis* Hochst.; kingdom: Plantae; family: Poaceae; genus: Aristida; specificEpithet: adoensis; scientificNameAuthorship: Hochst.; **Location:** continent: Africa; country: Tanzania; stateProvince: Shinyanga; locality: Lake Magadi; verbatimLocality: N.W. side of Lake Magadi; minimumElevationInMeters: 1410; decimalLatitude: -2.633333; decimalLongitude: 34.9; **Event:** eventDate: 1961-04-13; **Record Level:** institutionCode: SWRC; collectionCode: Herbarium; ownerInstitutionCode: SWRC; basisOfRecord: PreservedSpecimen
- **Type status:** Other material. **Occurrence:** catalogNumber: DB1083; recordNumber: 10183; recordedBy: Greenway, PJ; **Taxon:** scientificName: *Aristida adoensis* Hochst.; kingdom: Plantae; family: Poaceae; genus: Aristida; specificEpithet: adoensis; scientificNameAuthorship: Hochst.; **Location:** continent: Africa; country: Tanzania; stateProvince: Mara; county: Serengeti; locality: Seronera; verbatimLocality: West of Seronera; decimalLatitude: -2.45; decimalLongitude: 34.833333; **Event:** eventDate: 1961-05-15; **Record Level:** institutionCode: SWRC; collectionCode: Herbarium; ownerInstitutionCode: SWRC; basisOfRecord: PreservedSpecimen
- **Type status:** Other material. **Occurrence:** catalogNumber: DB1084; recordNumber: 601A; recordedBy: Herlocker, D; **Taxon:** scientificName: *Aristida adoensis* Hochst.; kingdom: Plantae; family: Poaceae; genus: Aristida; specificEpithet: adoensis; scientificNameAuthorship: Hochst.; **Location:** continent: Africa; country: Tanzania; stateProvince: Mara; county: Serengeti; locality: Serengeti; decimalLatitude: -2.333333; decimalLongitude: 34.833333; **Record Level:** institutionCode: SWRC; collectionCode: Herbarium; ownerInstitutionCode: SWRC; basisOfRecord: PreservedSpecimen
- **Type status:** Other material. **Occurrence:** catalogNumber: DB1085; recordNumber: 109; recordedBy: Schmidt, W; **Taxon:** scientificName: *Aristida adoensis* Hochst.; kingdom: Plantae; family: Poaceae; genus: Aristida; specificEpithet: adoensis; scientificNameAuthorship: Hochst.; **Location:** continent: Africa; country: Tanzania; stateProvince: Mara; county: Serengeti; locality: Seronera; verbatimLocality: South of Seronera, Serengeti plains; decimalLatitude: -2.45; decimalLongitude: 34.833333; **Event:** eventDate: 1972-03-14; **Record Level:** institutionCode: SWRC; collectionCode: Herbarium; ownerInstitutionCode: SWRC; basisOfRecord: PreservedSpecimen
- **Type status:** Other material. **Occurrence:** catalogNumber: DB1087; recordNumber: 12; recordedBy: Vesey-FitzGerald, LDEF; **Taxon:** scientificName: *Aristida adoensis* Hochst.; kingdom: Plantae; family: Poaceae; genus: Aristida; specificEpithet: adoensis; scientificNameAuthorship: Hochst.; **Location:** continent: Africa; country: Tanzania; stateProvince: Arusha; county: Ngorongoro; locality: Ngorongoro; decimalLatitude: -3.25; decimalLongitude: 35.533333; **Event:** eventDate: 1959-12-27; **Record Level:** institutionCode: SWRC; collectionCode: Herbarium; ownerInstitutionCode: SWRC; basisOfRecord: PreservedSpecimen
- **Type status:** Other material. **Occurrence:** catalogNumber: DB1089; recordNumber: 285; recordedBy: Belsky, PJ; **Taxon:** scientificName: *Aristida adoensis* Hochst.; kingdom: Plantae; family: Poaceae; genus: Aristida; specificEpithet: adoensis; scientificNameAuthorship: Hochst.; **Location:** continent: Africa; country: Tanzania; stateProvince: Mara; county: Serengeti; locality: Lobo Lodge; verbatimLocality: 5 km north of lobo lodge; decimalLatitude: -1.933333; decimalLongitude: 35.166667; **Event:** eventDate: 1981-05-31; **Record Level:** institutionCode: SWRC; collectionCode: Herbarium; ownerInstitutionCode: SWRC; basisOfRecord: PreservedSpecimen
- **Type status:** Other material. **Occurrence:** catalogNumber: K000805344; recordNumber: 368; recordedBy: Paulo, S; **Taxon:** scientificName: *Aristida adoensis* Hochst.; kingdom: Plantae; family: Poaceae; genus: Aristida; specificEpithet: adoensis; scientificNameAuthorship: Hochst.; **Location:** continent: Africa; country: Tanzania; stateProvince: Shinyanga; locality: Lake Magadi; verbatimLocality: N. of Lake Magadi, 10-15m. South of Seronera. Serengeti; decimalLatitude: -2.633333; decimalLongitude: 34.9; **Event:** eventDate: 1958-04-24; **Record Level:** institutionCode: K; collectionCode: Herbarium; ownerInstitutionCode: K; basisOfRecord: PreservedSpecimen
- **Type status:** Other material. **Occurrence:** catalogNumber: K000805338; recordNumber: 10183; recordedBy: Greenway, PJ; **Taxon:** scientificName: *Aristida adoensis* Hochst.; kingdom: Plantae; family: Poaceae; genus: Aristida; specificEpithet: adoensis; scientificNameAuthorship: Hochst.; **Location:** continent: Africa; country: Tanzania; stateProvince: Mara; county: Serengeti; locality: Seronera; verbatimLocality: West of Seronera; decimalLatitude: -2.45; decimalLongitude: 34.833333; **Event:** eventDate: 1961-05-15; **Record Level:** institutionCode: K; collectionCode: Herbarium; ownerInstitutionCode: K; basisOfRecord: PreservedSpecimen
- **Type status:** Other material. **Occurrence:** catalogNumber: 533; recordNumber: 10183; recordedBy: Greenway, PJ; **Taxon:** scientificName: *Aristida adoensis* Hochst.; kingdom: Plantae; family: Poaceae; genus: Aristida; specificEpithet: adoensis; scientificNameAuthorship: Hochst.; **Location:** continent: Africa; country: Tanzania; stateProvince: Mara; county: Serengeti; locality: Seronera; verbatimLocality: West of Seronera; decimalLatitude: -2.45; decimalLongitude: 34.833333; **Event:** eventDate: 1961-05-15; **Record Level:** institutionCode: EA; collectionCode: Herbarium; ownerInstitutionCode: EA; basisOfRecord: PreservedSpecimen
- **Type status:** Other material. **Occurrence:** catalogNumber: 534; recordNumber: 6561; recordedBy: Newbould, JB; **Taxon:** scientificName: *Aristida adoensis* Hochst.; kingdom: Plantae; family: Poaceae; genus: Aristida; specificEpithet: adoensis; scientificNameAuthorship: Hochst.; **Location:** continent: Africa; country: Tanzania; stateProvince: Shinyanga; locality: Granite Kopje; verbatimLocality: Granite Kopje 6 Miles North of Naabi, Serengeti.; minimumElevationInMeters: 1676; decimalLatitude: -2.783333; decimalLongitude: 35.333333; **Event:** eventDate: 1963-2; **Record Level:** institutionCode: EA; collectionCode: Herbarium; ownerInstitutionCode: EA; basisOfRecord: PreservedSpecimen
- **Type status:** Other material. **Occurrence:** catalogNumber: K000805339; recordNumber: 9902; recordedBy: Greenway, PJ; **Taxon:** scientificName: *Aristida adoensis* Hochst.; kingdom: Plantae; family: Poaceae; genus: Aristida; specificEpithet: adoensis; scientificNameAuthorship: Hochst.; **Location:** continent: Africa; country: Tanzania; stateProvince: Mara; county: Serengeti; locality: Seronera; verbatimLocality: Seronera to Soitayai, Mile 7, 8.; minimumElevationInMeters: 3018; decimalLatitude: -2.433333; decimalLongitude: 34.866667; **Event:** eventDate: 1961-03-27; **Record Level:** institutionCode: K; collectionCode: Herbarium; ownerInstitutionCode: K; basisOfRecord: PreservedSpecimen
- **Type status:** Other material. **Occurrence:** catalogNumber: 536; recordNumber: 9902; recordedBy: Greenway, PJ; **Taxon:** scientificName: *Aristida adoensis* Hochst.; kingdom: Plantae; family: Poaceae; genus: Aristida; specificEpithet: adoensis; scientificNameAuthorship: Hochst.; **Location:** continent: Africa; country: Tanzania; stateProvince: Mara; county: Serengeti; locality: Seronera; verbatimLocality: Seronera to Soitayai, Mile 7, 8.; minimumElevationInMeters: 3018; decimalLatitude: -2.433333; decimalLongitude: 34.866667; **Event:** eventDate: 1961-03-27; **Record Level:** institutionCode: EA; collectionCode: Herbarium; ownerInstitutionCode: EA; basisOfRecord: PreservedSpecimen
- **Type status:** Other material. **Occurrence:** catalogNumber: K000805337; recordNumber: 5599; recordedBy: Leippert, H; **Taxon:** scientificName: *Aristida adoensis* Hochst.; kingdom: Plantae; family: Poaceae; genus: Aristida; specificEpithet: adoensis; scientificNameAuthorship: Hochst.; **Location:** continent: Africa; country: Tanzania; stateProvince: Mara; county: Serengeti; locality: Banagi; verbatimLocality: Serengeti plain near Banagi; minimumElevationInMeters: 1597; decimalLatitude: -2.3; decimalLongitude: 34.833333; **Event:** eventDate: 1965-03-02; **Record Level:** institutionCode: K; collectionCode: Herbarium; ownerInstitutionCode: K; basisOfRecord: PreservedSpecimen
- **Type status:** Other material. **Occurrence:** catalogNumber: 538; recordNumber: 5599; recordedBy: Leippert, H; **Taxon:** scientificName: *Aristida adoensis* Hochst.; kingdom: Plantae; family: Poaceae; genus: Aristida; specificEpithet: adoensis; scientificNameAuthorship: Hochst.; **Location:** continent: Africa; country: Tanzania; stateProvince: Mara; county: Serengeti; locality: Banagi; verbatimLocality: Serengeti plain near Banagi; minimumElevationInMeters: 1597; decimalLatitude: -2.3; decimalLongitude: 34.833333; **Event:** eventDate: 1965-03-02; **Record Level:** institutionCode: EA; collectionCode: Herbarium; ownerInstitutionCode: EA; basisOfRecord: PreservedSpecimen
- **Type status:** Other material. **Occurrence:** catalogNumber: DB1082; recordNumber: 9902; recordedBy: Greenway, PJ; **Taxon:** scientificName: *Aristida adoensis* Hochst.; kingdom: Plantae; family: Poaceae; genus: Aristida; specificEpithet: adoensis; scientificNameAuthorship: Hochst.; **Location:** continent: Africa; country: Tanzania; stateProvince: Mara; county: Serengeti; locality: Seronera; verbatimLocality: Seronera to Soitayai, Mile 7, 8.; minimumElevationInMeters: 3018; decimalLatitude: -2.433333; decimalLongitude: 34.866667; **Event:** eventDate: 1961-03-27; **Record Level:** institutionCode: SWRC; collectionCode: Herbarium; ownerInstitutionCode: SWRC; basisOfRecord: PreservedSpecimen
- **Type status:** Other material. **Occurrence:** catalogNumber: 808; recordNumber: 10050; recordedBy: Greenway, PJ; Tanner, M; **Taxon:** scientificName: *Aristida adoensis* Hochst.; kingdom: Plantae; family: Poaceae; genus: Aristida; specificEpithet: adoensis; scientificNameAuthorship: Hochst.; **Location:** continent: Africa; country: Tanzania; stateProvince: Shinyanga; locality: Lake Magadi; verbatimLocality: N.W. side of Lake Magadi; minimumElevationInMeters: 1410; decimalLatitude: -2.633333; decimalLongitude: 34.9; **Event:** eventDate: 1961-04-13; **Record Level:** institutionCode: EA; collectionCode: Herbarium; ownerInstitutionCode: EA; basisOfRecord: PreservedSpecimen
- **Type status:** Other material. **Occurrence:** catalogNumber: 809; recordNumber: 6449; recordedBy: Newbould, JB; **Taxon:** scientificName: *Aristida adoensis* Hochst.; kingdom: Plantae; family: Poaceae; genus: Aristida; specificEpithet: adoensis; scientificNameAuthorship: Hochst.; **Location:** continent: Africa; country: Tanzania; stateProvince: Arusha; county: Ngorongoro; locality: Endulen; verbatimLocality: Ngorongoro pasture research scheme. Olgoum nr. Endulen.; decimalLatitude: -3.183333; decimalLongitude: 35.15; **Event:** eventDate: 1962-12-24; **Record Level:** institutionCode: EA; collectionCode: Herbarium; ownerInstitutionCode: EA; basisOfRecord: PreservedSpecimen
- **Type status:** Other material. **Occurrence:** catalogNumber: 824; recordNumber: 6449; recordedBy: Kirika, P; **Taxon:** scientificName: *Aristida adoensis* Hochst.; kingdom: Plantae; family: Poaceae; genus: Aristida; specificEpithet: adoensis; scientificNameAuthorship: Hochst.; **Location:** continent: Africa; country: Tanzania; stateProvince: Arusha; county: Ngorongoro; locality: Endulen; verbatimLocality: Ngorongoro pasture research scheme. Elgoum nr. Endulen.; minimumElevationInMeters: 1981; decimalLatitude: -3.2; decimalLongitude: 35.283; **Event:** eventDate: 1962-12-24; **Record Level:** institutionCode: EA; collectionCode: Herbarium; ownerInstitutionCode: EA; basisOfRecord: PreservedSpecimen
- **Type status:** Other material. **Occurrence:** catalogNumber: 825; recordNumber: 5925; recordedBy: Kirika, P; **Taxon:** scientificName: *Aristida adoensis* Hochst.; kingdom: Plantae; family: Poaceae; genus: Aristida; specificEpithet: adoensis; scientificNameAuthorship: Hochst.; **Location:** continent: Africa; country: Tanzania; stateProvince: Arusha; county: Ngorongoro; locality: Olbalbal; verbatimLocality: Longo - Olbalbal.; minimumElevationInMeters: 1417; decimalLatitude: -3; decimalLongitude: 35.5; **Event:** eventDate: 1962-01-30; **Record Level:** institutionCode: EA; collectionCode: Herbarium; ownerInstitutionCode: EA; basisOfRecord: PreservedSpecimen

##### Distribution

Eastern Africa

#### Aristida adscensionis

L.

##### Materials

- **Type status:** Other material. **Occurrence:** catalogNumber: K000710861; recordNumber: 2613; recordedBy: Chuwa, S; **Taxon:** scientificName: *Aristida adscensionis* L.; kingdom: Plantae; family: Poaceae; genus: Aristida; specificEpithet: adscensionis; scientificNameAuthorship: L.; **Location:** continent: Africa; country: Tanzania; stateProvince: Arusha; county: Ngorongoro; locality: Olduvai; minimumElevationInMeters: 1450; decimalLatitude: -2.966667; decimalLongitude: 35.366667; **Event:** eventDate: 1987-05-01; **Record Level:** institutionCode: K; collectionCode: Herbarium; ownerInstitutionCode: K; basisOfRecord: PreservedSpecimen
- **Type status:** Other material. **Occurrence:** catalogNumber: K000710860; recordNumber: 2798; recordedBy: Chuwa, S; **Taxon:** scientificName: *Aristida adscensionis* L.; kingdom: Plantae; family: Poaceae; genus: Aristida; specificEpithet: adscensionis; scientificNameAuthorship: L.; **Location:** continent: Africa; country: Tanzania; stateProvince: Arusha; county: Ngorongoro; locality: Ngorongoro Crater; minimumElevationInMeters: 1722; decimalLatitude: -2.916667; decimalLongitude: 35.8; **Event:** eventDate: 1989-06-01; **Record Level:** institutionCode: K; collectionCode: Herbarium; ownerInstitutionCode: K; basisOfRecord: PreservedSpecimen
- **Type status:** Other material. **Occurrence:** catalogNumber: 541; recordNumber: s.n.; recordedBy: Mboya, E; **Taxon:** scientificName: *Aristida adscensionis* L.; kingdom: Plantae; family: Poaceae; genus: Aristida; specificEpithet: adscensionis; scientificNameAuthorship: L.; **Location:** continent: Africa; country: Tanzania; stateProvince: Mara; county: Serengeti; locality: Serengeti Research Centre; verbatimLocality: T1. Serengeti Research Centre.; minimumElevationInMeters: 1550; decimalLatitude: -2.38; decimalLongitude: 34.85; **Event:** eventDate: 2004-02-09; **Record Level:** institutionCode: MO; collectionCode: Herbarium; ownerInstitutionCode: MO; basisOfRecord: PreservedSpecimen
- **Type status:** Other material. **Occurrence:** catalogNumber: 826; recordNumber: 6026; recordedBy: Kirika, P; **Taxon:** scientificName: *Aristida adscensionis* L.; kingdom: Plantae; family: Poaceae; genus: Aristida; specificEpithet: adscensionis; scientificNameAuthorship: L.; **Location:** continent: Africa; country: Tanzania; stateProvince: Arusha; county: Ngorongoro; locality: Olbalbal; verbatimLocality: Olbalbal depression. On the North slopes of Olmoti Where they run down to the Salei Desert.; minimumElevationInMeters: 1463; decimalLatitude: -3; decimalLongitude: 35.5; **Event:** eventDate: 1962-5; **Record Level:** institutionCode: EA; collectionCode: Herbarium; ownerInstitutionCode: EA; basisOfRecord: PreservedSpecimen

##### Distribution

Widespread

#### Aristida barbicollis

Trin. & Rupr.

##### Materials

- **Type status:** Other material. **Occurrence:** catalogNumber: DB1090; recordNumber: 13324; recordedBy: Greenway, PJ; Kanuri; Tanner, M; **Taxon:** scientificName: *Aristida barbicollis* Trin. & Rupr.; kingdom: Plantae; family: Poaceae; genus: Aristida; specificEpithet: barbicollis; scientificNameAuthorship: Trin. & Rupr.; **Location:** continent: Africa; country: Tanzania; stateProvince: Mara; county: Serengeti; locality: Serengeti; minimumElevationInMeters: 1230; decimalLatitude: -2.333333; decimalLongitude: 34.833333; **Event:** eventDate: 1968-02-21; **Record Level:** institutionCode: SWRC; collectionCode: Herbarium; ownerInstitutionCode: SWRC; basisOfRecord: PreservedSpecimen
- **Type status:** Other material. **Occurrence:** catalogNumber: DB1091; recordNumber: 10303; recordedBy: Greenway, PJ; **Taxon:** scientificName: *Aristida barbicollis* Trin. & Rupr.; kingdom: Plantae; family: Poaceae; genus: Aristida; specificEpithet: barbicollis; scientificNameAuthorship: Trin. & Rupr.; **Location:** continent: Africa; country: Tanzania; stateProvince: Mara; county: Serengeti; locality: Seronera; verbatimLocality: Seronera to Klein's Camp, mile 55.3.; minimumElevationInMeters: 1798; decimalLatitude: -1.883333; decimalLongitude: 35.383333; **Event:** eventDate: 1961-05-17; **Record Level:** institutionCode: SWRC; collectionCode: Herbarium; ownerInstitutionCode: SWRC; basisOfRecord: PreservedSpecimen
- **Type status:** Other material. **Occurrence:** catalogNumber: K000805098; recordNumber: 10303; recordedBy: Greenway, PJ; **Taxon:** scientificName: *Aristida barbicollis* Trin. & Rupr.; kingdom: Plantae; family: Poaceae; genus: Aristida; specificEpithet: barbicollis; scientificNameAuthorship: Trin. & Rupr.; **Location:** continent: Africa; country: Tanzania; stateProvince: Mara; county: Serengeti; locality: Seronera; verbatimLocality: Seronera to Klein's Camp, mile 55.3.; minimumElevationInMeters: 1798; decimalLatitude: -1.883333; decimalLongitude: 35.383333; **Event:** eventDate: 1961-05-17; **Record Level:** institutionCode: K; collectionCode: Herbarium; ownerInstitutionCode: K; basisOfRecord: PreservedSpecimen
- **Type status:** Other material. **Occurrence:** catalogNumber: 530; recordNumber: 10303; recordedBy: Greenway, PJ; **Taxon:** scientificName: *Aristida barbicollis* Trin. & Rupr.; kingdom: Plantae; family: Poaceae; genus: Aristida; specificEpithet: barbicollis; scientificNameAuthorship: Trin. & Rupr.; **Location:** continent: Africa; country: Tanzania; stateProvince: Mara; county: Serengeti; locality: Seronera; verbatimLocality: Seronera to Klein's Camp, mile 55.3.; minimumElevationInMeters: 1798; decimalLatitude: -1.883333; decimalLongitude: 35.383333; **Event:** eventDate: 1961-05-17; **Record Level:** institutionCode: EA; collectionCode: Herbarium; ownerInstitutionCode: EA; basisOfRecord: PreservedSpecimen

##### Distribution

Eastern & Southern Africa

#### Aristida hordeacea

Kunth

##### Materials

- **Type status:** Other material. **Occurrence:** catalogNumber: DB1116; recordNumber: 1077; recordedBy: Suleiman, HO; Lyaruu, HVM; Lyamuya, N; **Taxon:** scientificName: *Aristida hordeacea* Kunth; kingdom: Plantae; family: Poaceae; genus: Aristida; specificEpithet: hordeacea; scientificNameAuthorship: Kunth; **Location:** continent: Africa; country: Tanzania; stateProvince: Mara; county: Serengeti; locality: Serengeti National Park; verbatimLocality: Mihale game transect (MGT 401); decimalLatitude: -2.333333; decimalLongitude: 34.833333; **Event:** eventDate: 2001-02-15; **Record Level:** institutionCode: SWRC; collectionCode: Herbarium; ownerInstitutionCode: SWRC; basisOfRecord: PreservedSpecimen
- **Type status:** Other material. **Occurrence:** catalogNumber: DB1117; recordNumber: 1077; recordedBy: Suleiman, HO; Lyaruu, HVM; Lyamuya, N; **Taxon:** scientificName: *Aristida hordeacea* Kunth; kingdom: Plantae; family: Poaceae; genus: Aristida; specificEpithet: hordeacea; scientificNameAuthorship: Kunth; **Location:** continent: Africa; country: Tanzania; stateProvince: Mara; county: Serengeti; locality: Serengeti National Park; verbatimLocality: Mihale game transect (MGT 401); decimalLatitude: -2.333333; decimalLongitude: 34.833333; **Event:** eventDate: 2001-02-15; **Record Level:** institutionCode: SWRC; collectionCode: Herbarium; ownerInstitutionCode: SWRC; basisOfRecord: PreservedSpecimen
- **Type status:** Other material. **Occurrence:** catalogNumber: K000805174; recordNumber: 443; recordedBy: Paulo, S; **Taxon:** scientificName: *Aristida hordeacea* Kunth; kingdom: Plantae; family: Poaceae; genus: Aristida; specificEpithet: hordeacea; scientificNameAuthorship: Kunth; **Location:** continent: Africa; country: Tanzania; stateProvince: Mara; county: Serengeti; locality: Serengeti; verbatimLocality: E. of Pandayega.; decimalLatitude: -2.333333; decimalLongitude: 34.833333; **Event:** eventDate: 1958-05-09; **Record Level:** institutionCode: K; collectionCode: Herbarium; ownerInstitutionCode: K; basisOfRecord: PreservedSpecimen
- **Type status:** Other material. **Occurrence:** catalogNumber: 526; recordNumber: 443; recordedBy: Paulo, S; **Taxon:** scientificName: *Aristida hordeacea* Kunth; kingdom: Plantae; family: Poaceae; genus: Aristida; specificEpithet: hordeacea; scientificNameAuthorship: Kunth; **Location:** continent: Africa; country: Tanzania; stateProvince: Mara; county: Serengeti; locality: Serengeti; verbatimLocality: E. of Pandayega.; decimalLatitude: -2.333333; decimalLongitude: 34.833333; **Event:** eventDate: 1958-05-09; **Record Level:** institutionCode: EA; collectionCode: Herbarium; ownerInstitutionCode: EA; basisOfRecord: PreservedSpecimen
- **Type status:** Other material. **Occurrence:** catalogNumber: K000805185; recordNumber: 10089; recordedBy: Greenway, PJ; **Taxon:** scientificName: *Aristida hordeacea* Kunth; kingdom: Plantae; family: Poaceae; genus: Aristida; specificEpithet: hordeacea; scientificNameAuthorship: Kunth; **Location:** continent: Africa; country: Tanzania; stateProvince: Mara; county: Serengeti; locality: Seronera; verbatimLocality: On the South Western side of Nyamuma Range and of Nyamuma Guard post on Mbalageti river. Seronera to Kirawira guard post, mile 68.8. Grumeti River.; minimumElevationInMeters: 1158; **Event:** eventDate: 1961-04-24; **Record Level:** institutionCode: K; collectionCode: Herbarium; ownerInstitutionCode: K; basisOfRecord: PreservedSpecimen
- **Type status:** Other material. **Occurrence:** catalogNumber: 528; recordNumber: 10089; recordedBy: Greenway, PJ; **Taxon:** scientificName: *Aristida hordeacea* Kunth; kingdom: Plantae; family: Poaceae; genus: Aristida; specificEpithet: hordeacea; scientificNameAuthorship: Kunth; **Location:** continent: Africa; country: Tanzania; stateProvince: Mara; county: Serengeti; locality: Seronera; verbatimLocality: On the South Western side of Nyamuma Range and of Nyamuma Guard post on Mbalageti river. Seronera to Kirawira guard post, mile 68.8. Grumeti River.; minimumElevationInMeters: 1158; **Event:** eventDate: 1961-04-24; **Record Level:** institutionCode: EA; collectionCode: Herbarium; ownerInstitutionCode: EA; basisOfRecord: PreservedSpecimen
- **Type status:** Other material. **Occurrence:** catalogNumber: DB1118; recordNumber: 10089; recordedBy: Greenway, PJ; **Taxon:** scientificName: *Aristida hordeacea* Kunth; kingdom: Plantae; family: Poaceae; genus: Aristida; specificEpithet: hordeacea; scientificNameAuthorship: Kunth; **Location:** continent: Africa; country: Tanzania; stateProvince: Mara; county: Serengeti; locality: Seronera; verbatimLocality: On the South Western side of Nyamuma Range and of Nyamuma Guard post on Mbalageti river. Seronera to Kirawira guard post, mile 68.8. Grumeti River.; minimumElevationInMeters: 1158; **Event:** eventDate: 1961-04-24; **Record Level:** institutionCode: SWRC; collectionCode: Herbarium; ownerInstitutionCode: SWRC; basisOfRecord: PreservedSpecimen

##### Distribution

Tropical & Southern Africa

#### Aristida kenyensis

Henrard

##### Materials

- **Type status:** Other material. **Occurrence:** catalogNumber: 1; recordNumber: 163; recordedBy: Newbould, JB; **Taxon:** scientificName: *Aristida kenyensis* Henrard; kingdom: Plantae; family: Poaceae; genus: Aristida; specificEpithet: kenyensis; scientificNameAuthorship: Henrard; **Location:** continent: Africa; country: Tanzania; stateProvince: Arusha; county: Ngorongoro; locality: Lemuta hill; verbatimLocality: E. Serengeti.; decimalLatitude: -2.7; decimalLongitude: 35.266667; **Event:** eventDate: 1962-07-20; **Record Level:** institutionCode: EA; collectionCode: Herbarium; ownerInstitutionCode: EA; basisOfRecord: PreservedSpecimen
- **Type status:** Other material. **Occurrence:** catalogNumber: 2; recordNumber: 6026; recordedBy: Newbould, JB; **Taxon:** scientificName: *Aristida kenyensis* Henrard; kingdom: Plantae; family: Poaceae; genus: Aristida; specificEpithet: kenyensis; scientificNameAuthorship: Henrard; **Location:** continent: Africa; country: Tanzania; stateProvince: Arusha; county: Ngorongoro; locality: Olbalbal; verbatimLocality: Olbalbal depression, on the North slopes of Olmoti where they run down to the Salei Desert.; minimumElevationInMeters: 1463; decimalLatitude: -3.083333; decimalLongitude: 35.416667; **Event:** eventDate: 1962-5; **Record Level:** institutionCode: EA; collectionCode: Herbarium; ownerInstitutionCode: EA; basisOfRecord: PreservedSpecimen
- **Type status:** Other material. **Occurrence:** catalogNumber: DB1096; recordNumber: 10327; recordedBy: Greenway, PJ; **Taxon:** scientificName: *Aristida kenyensis* Henrard; kingdom: Plantae; family: Poaceae; genus: Aristida; specificEpithet: kenyensis; scientificNameAuthorship: Henrard; **Location:** continent: Africa; country: Tanzania; stateProvince: Mara; county: Serengeti; locality: Musoma; verbatimLocality: Eastern boundary; decimalLatitude: -1.5; decimalLongitude: 34.683333; **Event:** eventDate: 1961-05-25; **Record Level:** institutionCode: SWRC; collectionCode: Herbarium; ownerInstitutionCode: SWRC; basisOfRecord: PreservedSpecimen
- **Type status:** Other material. **Occurrence:** catalogNumber: DB1097; recordNumber: 186; recordedBy: Schmidt, W; **Taxon:** scientificName: *Aristida kenyensis* Henrard; kingdom: Plantae; family: Poaceae; genus: Aristida; specificEpithet: kenyensis; scientificNameAuthorship: Henrard; **Location:** continent: Africa; country: Tanzania; stateProvince: Mara; county: Serengeti; locality: Simba Kopjes; verbatimLocality: Serengeti plains, simba Kopjes North; decimalLatitude: -2.7; decimalLongitude: 34.916667; **Event:** eventDate: 1972-03-20; **Record Level:** institutionCode: SWRC; collectionCode: Herbarium; ownerInstitutionCode: SWRC; basisOfRecord: PreservedSpecimen
- **Type status:** Other material. **Occurrence:** catalogNumber: DB1098; recordNumber: 10188; recordedBy: Greenway, PJ; **Taxon:** scientificName: *Aristida kenyensis* Henrard; kingdom: Plantae; family: Poaceae; genus: Aristida; specificEpithet: kenyensis; scientificNameAuthorship: Henrard; **Location:** continent: Africa; country: Tanzania; stateProvince: Mara; county: Serengeti; locality: Seronera; verbatimLocality: W. of Seronera, Serengeti.; minimumElevationInMeters: 1463; decimalLatitude: -2.45; decimalLongitude: 34.833333; **Event:** eventDate: 1961-05-13; **Record Level:** institutionCode: SWRC; collectionCode: Herbarium; ownerInstitutionCode: SWRC; basisOfRecord: PreservedSpecimen
- **Type status:** Other material. **Occurrence:** catalogNumber: DB1099; recordNumber: 9091; recordedBy: Greenway, PJ; **Taxon:** scientificName: *Aristida kenyensis* Henrard; kingdom: Plantae; family: Poaceae; genus: Aristida; specificEpithet: kenyensis; scientificNameAuthorship: Henrard; **Location:** continent: Africa; country: Tanzania; stateProvince: Mara; county: Serengeti; locality: Seronera; verbatimLocality: Seronera to Soitayai, Mile 7.8; minimumElevationInMeters: 1590; decimalLatitude: -2.433333; decimalLongitude: 34.866667; **Event:** eventDate: 1961-03-27; **Record Level:** institutionCode: SWRC; collectionCode: Herbarium; ownerInstitutionCode: SWRC; basisOfRecord: PreservedSpecimen
- **Type status:** Other material. **Occurrence:** catalogNumber: DB1103; recordNumber: 68; recordedBy: Belsky, PJ; **Taxon:** scientificName: *Aristida kenyensis* Henrard; kingdom: Plantae; family: Poaceae; genus: Aristida; specificEpithet: kenyensis; scientificNameAuthorship: Henrard; **Location:** continent: Africa; country: Tanzania; stateProvince: Mara; county: Serengeti; locality: Ndutu Lodge; verbatimLocality: Near road 50 m from Ndutu lodge; decimalLatitude: -3.02; decimalLongitude: 34.996944; **Event:** eventDate: 1981-04-10; **Record Level:** institutionCode: SWRC; collectionCode: Herbarium; ownerInstitutionCode: SWRC; basisOfRecord: PreservedSpecimen
- **Type status:** Other material. **Occurrence:** catalogNumber: DB1104; recordNumber: 120; recordedBy: Belsky, PJ; **Taxon:** scientificName: *Aristida kenyensis* Henrard; kingdom: Plantae; family: Poaceae; genus: Aristida; specificEpithet: kenyensis; scientificNameAuthorship: Henrard; **Location:** continent: Africa; country: Tanzania; stateProvince: Arusha; county: Ngorongoro; locality: Olduvai; verbatimLocality: Rim of Olduvai Gorge, inside Leakey compound; decimalLatitude: -2.966667; decimalLongitude: 35.366667; **Event:** eventDate: 1981-04-20; **Record Level:** institutionCode: SWRC; collectionCode: Herbarium; ownerInstitutionCode: SWRC; basisOfRecord: PreservedSpecimen
- **Type status:** Other material. **Occurrence:** catalogNumber: K000805407; recordNumber: 193; recordedBy: Oteke, J; **Taxon:** scientificName: *Aristida kenyensis* Henrard; kingdom: Plantae; family: Poaceae; genus: Aristida; specificEpithet: kenyensis; scientificNameAuthorship: Henrard; **Location:** continent: Africa; country: Tanzania; stateProvince: Arusha; county: Ngorongoro; locality: Lemuta hill; verbatimLocality: East Serengeti; minimumElevationInMeters: 1524; decimalLatitude: -2.7; decimalLongitude: 35.266667; **Event:** eventDate: 1962-07-20; **Record Level:** institutionCode: K; collectionCode: Herbarium; ownerInstitutionCode: K; basisOfRecord: PreservedSpecimen
- **Type status:** Other material. **Occurrence:** catalogNumber: 511; recordNumber: 5925; recordedBy: Newbould, JB; **Taxon:** scientificName: *Aristida kenyensis* Henrard; kingdom: Plantae; family: Poaceae; genus: Aristida; specificEpithet: kenyensis; scientificNameAuthorship: Henrard; **Location:** continent: Africa; country: Tanzania; stateProvince: Arusha; county: Ngorongoro; locality: Olbalbal; verbatimLocality: Longogo- Olbalbal; minimumElevationInMeters: 1417; decimalLatitude: -2.95; decimalLongitude: 35.283333; **Event:** eventDate: 1962-01-30; **Record Level:** institutionCode: EA; collectionCode: Herbarium; ownerInstitutionCode: EA; basisOfRecord: PreservedSpecimen
- **Type status:** Other material. **Occurrence:** catalogNumber: K000805403; recordNumber: 10188; recordedBy: Greenway, PJ; **Taxon:** scientificName: *Aristida kenyensis* Henrard; kingdom: Plantae; family: Poaceae; genus: Aristida; specificEpithet: kenyensis; scientificNameAuthorship: Henrard; **Location:** continent: Africa; country: Tanzania; stateProvince: Mara; county: Serengeti; locality: Seronera; verbatimLocality: W. of Seronera, Serengeti.; minimumElevationInMeters: 1463; decimalLatitude: -2.45; decimalLongitude: 34.833333; **Event:** eventDate: 1961-05-13; **Record Level:** institutionCode: K; collectionCode: Herbarium; ownerInstitutionCode: K; basisOfRecord: PreservedSpecimen
- **Type status:** Other material. **Occurrence:** catalogNumber: 513; recordNumber: 10188; recordedBy: Greenway, PJ; **Taxon:** scientificName: *Aristida kenyensis* Henrard; kingdom: Plantae; family: Poaceae; genus: Aristida; specificEpithet: kenyensis; scientificNameAuthorship: Henrard; **Location:** continent: Africa; country: Tanzania; stateProvince: Mara; county: Serengeti; locality: Seronera; verbatimLocality: W. of Seronera, Serengeti.; minimumElevationInMeters: 1463; decimalLatitude: -2.45; decimalLongitude: 34.833333; **Event:** eventDate: 1961-05-13; **Record Level:** institutionCode: EA; collectionCode: Herbarium; ownerInstitutionCode: EA; basisOfRecord: PreservedSpecimen
- **Type status:** Other material. **Occurrence:** catalogNumber: K000805397; recordNumber: 10051; recordedBy: Greenway, PJ; Tanner, M; **Taxon:** scientificName: *Aristida kenyensis* Henrard; kingdom: Plantae; family: Poaceae; genus: Aristida; specificEpithet: kenyensis; scientificNameAuthorship: Henrard; **Location:** continent: Africa; country: Tanzania; stateProvince: Shinyanga; locality: Lake Magadi; verbatimLocality: NW side of Lake Magadi.; minimumElevationInMeters: 1432; decimalLatitude: -2.633333; decimalLongitude: 34.9; **Event:** eventDate: 1961-04-13; **Record Level:** institutionCode: K; collectionCode: Herbarium; ownerInstitutionCode: K; basisOfRecord: PreservedSpecimen
- **Type status:** Other material. **Occurrence:** catalogNumber: 515; recordNumber: 10051; recordedBy: Greenway, PJ; Tanner, M; **Taxon:** scientificName: *Aristida kenyensis* Henrard; kingdom: Plantae; family: Poaceae; genus: Aristida; specificEpithet: kenyensis; scientificNameAuthorship: Henrard; **Location:** continent: Africa; country: Tanzania; stateProvince: Shinyanga; locality: Lake Magadi; verbatimLocality: NW side of Lake Magadi.; minimumElevationInMeters: 1432; decimalLatitude: -2.633333; decimalLongitude: 34.9; **Event:** eventDate: 1961-04-13; **Record Level:** institutionCode: EA; collectionCode: Herbarium; ownerInstitutionCode: EA; basisOfRecord: PreservedSpecimen
- **Type status:** Other material. **Occurrence:** catalogNumber: K000805389; recordNumber: 9973; recordedBy: Greenway, PJ; **Taxon:** scientificName: *Aristida kenyensis* Henrard; kingdom: Plantae; family: Poaceae; genus: Aristida; specificEpithet: kenyensis; scientificNameAuthorship: Henrard; **Location:** continent: Africa; country: Tanzania; stateProvince: Mara; county: Serengeti; locality: Seronera; verbatimLocality: Banagi to Seronera river crossing, down the corridor, mile 14.5 from Seronera.; minimumElevationInMeters: 1371; decimalLatitude: -2.283333; decimalLongitude: 34.983333; **Event:** eventDate: 1961-04-04; **Record Level:** institutionCode: K; collectionCode: Herbarium; ownerInstitutionCode: K; basisOfRecord: PreservedSpecimen
- **Type status:** Other material. **Occurrence:** catalogNumber: 517; recordNumber: 9973; recordedBy: Greenway, PJ; **Taxon:** scientificName: *Aristida kenyensis* Henrard; kingdom: Plantae; family: Poaceae; genus: Aristida; specificEpithet: kenyensis; scientificNameAuthorship: Henrard; **Location:** continent: Africa; country: Tanzania; stateProvince: Mara; county: Serengeti; locality: Seronera; verbatimLocality: Banagi to Seronera river crossing, down the corridor, mile 14.5 from Seronera.; minimumElevationInMeters: 1371; decimalLatitude: -2.283333; decimalLongitude: 34.983333; **Event:** eventDate: 1961-04-04; **Record Level:** institutionCode: EA; collectionCode: Herbarium; ownerInstitutionCode: EA; basisOfRecord: PreservedSpecimen
- **Type status:** Other material. **Occurrence:** catalogNumber: K000805402; recordNumber: 9901; recordedBy: Greenway, PJ; **Taxon:** scientificName: *Aristida kenyensis* Henrard; kingdom: Plantae; family: Poaceae; genus: Aristida; specificEpithet: kenyensis; scientificNameAuthorship: Henrard; **Location:** continent: Africa; country: Tanzania; stateProvince: Mara; county: Serengeti; locality: Seronera; verbatimLocality: Seronera to Soitayai, mile 7.8.; minimumElevationInMeters: 1615; decimalLatitude: -2.433333; decimalLongitude: 34.866667; **Event:** eventDate: 1961-03-27; **Record Level:** institutionCode: K; collectionCode: Herbarium; ownerInstitutionCode: K; basisOfRecord: PreservedSpecimen
- **Type status:** Other material. **Occurrence:** catalogNumber: 519; recordNumber: 9901; recordedBy: Greenway, PJ; **Taxon:** scientificName: *Aristida kenyensis* Henrard; kingdom: Plantae; family: Poaceae; genus: Aristida; specificEpithet: kenyensis; scientificNameAuthorship: Henrard; **Location:** continent: Africa; country: Tanzania; stateProvince: Mara; county: Serengeti; locality: Seronera; verbatimLocality: Seronera to Soitayai, mile 7.8.; minimumElevationInMeters: 1615; decimalLatitude: -2.433333; decimalLongitude: 34.866667; **Event:** eventDate: 1961-03-27; **Record Level:** institutionCode: EA; collectionCode: Herbarium; ownerInstitutionCode: EA; basisOfRecord: PreservedSpecimen
- **Type status:** Other material. **Occurrence:** catalogNumber: K000805405; recordNumber: 354; recordedBy: Paulo, S; **Taxon:** scientificName: *Aristida kenyensis* Henrard; kingdom: Plantae; family: Poaceae; genus: Aristida; specificEpithet: kenyensis; scientificNameAuthorship: Henrard; **Location:** continent: Africa; country: Tanzania; stateProvince: Mara; county: Serengeti; locality: Seronera; verbatimLocality: Near rest Camp.; decimalLatitude: -2.45; decimalLongitude: 34.833333; **Event:** eventDate: 1958-04-23; **Record Level:** institutionCode: K; collectionCode: Herbarium; ownerInstitutionCode: K; basisOfRecord: PreservedSpecimen
- **Type status:** Other material. **Occurrence:** catalogNumber: 521; recordNumber: 354; recordedBy: Paulo, S; **Taxon:** scientificName: *Aristida kenyensis* Henrard; kingdom: Plantae; family: Poaceae; genus: Aristida; specificEpithet: kenyensis; scientificNameAuthorship: Henrard; **Location:** continent: Africa; country: Tanzania; stateProvince: Mara; county: Serengeti; locality: Seronera; verbatimLocality: Near rest Camp.; decimalLatitude: -2.45; decimalLongitude: 34.833333; **Event:** eventDate: 1958-04-23; **Record Level:** institutionCode: EA; collectionCode: Herbarium; ownerInstitutionCode: EA; basisOfRecord: PreservedSpecimen
- **Type status:** Other material. **Occurrence:** catalogNumber: K000805390; recordNumber: 326; recordedBy: Paulo, S; **Taxon:** scientificName: *Aristida kenyensis* Henrard; kingdom: Plantae; family: Poaceae; genus: Aristida; specificEpithet: kenyensis; scientificNameAuthorship: Henrard; **Location:** continent: Africa; country: Tanzania; stateProvince: Arusha; county: Ngorongoro; locality: Olbalbal; decimalLatitude: -2.95; decimalLongitude: 35.283333; **Event:** eventDate: 1958-04-20; **Record Level:** institutionCode: K; collectionCode: Herbarium; ownerInstitutionCode: K; basisOfRecord: PreservedSpecimen
- **Type status:** Other material. **Occurrence:** catalogNumber: 524; recordNumber: 11181; recordedBy: Greenway, PJ; **Taxon:** scientificName: *Aristida kenyensis* Henrard; kingdom: Plantae; family: Poaceae; genus: Aristida; specificEpithet: kenyensis; scientificNameAuthorship: Henrard; **Location:** continent: Africa; country: Tanzania; stateProvince: Mara; county: Serengeti; locality: Serengeti Plains; verbatimLocality: mile 27 ESE of Seronera camp.; minimumElevationInMeters: 1707; decimalLatitude: -2.583333; decimalLongitude: 35.183333; **Event:** eventDate: 1956-12-03; **Record Level:** institutionCode: EA; collectionCode: Herbarium; ownerInstitutionCode: EA; basisOfRecord: PreservedSpecimen
- **Type status:** Other material. **Occurrence:** catalogNumber: 542; recordNumber: 24297; recordedBy: Peterson, PM; Soreng, RJ; Romaschenko, K; Mbago, F; **Taxon:** scientificName: *Aristida kenyensis* Henrard; kingdom: Plantae; family: Poaceae; genus: Aristida; specificEpithet: kenyensis; scientificNameAuthorship: Henrard; **Location:** continent: Africa; country: Tanzania; stateProvince: Mara; county: Serengeti; locality: Lobo Lodge; verbatimLocality: Serengeti National Park, on top of Lobo Ridge above Lobo Lodge.; minimumElevationInMeters: 1817; decimalLatitude: -1.99967; decimalLongitude: 35.16778; **Event:** eventDate: 2012-06-17; **Record Level:** institutionCode: US; collectionCode: Herbarium; ownerInstitutionCode: US; basisOfRecord: PreservedSpecimen
- **Type status:** Other material. **Occurrence:** catalogNumber: 543; recordNumber: 24316; recordedBy: Peterson, PM; Soreng, RJ; Romaschenko, K; Mbago, F; **Taxon:** scientificName: *Aristida kenyensis* Henrard; kingdom: Plantae; family: Poaceae; genus: Aristida; specificEpithet: kenyensis; scientificNameAuthorship: Henrard; **Location:** continent: Africa; country: Tanzania; stateProvince: Arusha; county: Ngorongoro; locality: Lake Magadi; verbatimLocality: Ngorongoro Conservation Area, W side of Lake Magadi.; minimumElevationInMeters: 1739; decimalLatitude: -3.17713; decimalLongitude: 35.51548; **Event:** eventDate: 2012-06-19; **Record Level:** institutionCode: US; collectionCode: Herbarium; ownerInstitutionCode: US; basisOfRecord: PreservedSpecimen
- **Type status:** Other material. **Occurrence:** catalogNumber: DB1094; recordNumber: 9973; recordedBy: Greenway, PJ; **Taxon:** scientificName: *Aristida kenyensis* Henrard; kingdom: Plantae; family: Poaceae; genus: Aristida; specificEpithet: kenyensis; scientificNameAuthorship: Henrard; **Location:** continent: Africa; country: Tanzania; stateProvince: Mara; county: Serengeti; locality: Seronera; verbatimLocality: Banagi to Seronera river crossing, down the corridor, mile 14.5 from Seronera.; minimumElevationInMeters: 1371; decimalLatitude: -2.283333; decimalLongitude: 34.983333; **Event:** eventDate: 1961-04-04; **Record Level:** institutionCode: SWRC; collectionCode: Herbarium; ownerInstitutionCode: SWRC; basisOfRecord: PreservedSpecimen
- **Type status:** Other material. **Occurrence:** catalogNumber: DB1095; recordNumber: 10051; recordedBy: Greenway, PJ; Tanner, M; **Taxon:** scientificName: *Aristida kenyensis* Henrard; kingdom: Plantae; family: Poaceae; genus: Aristida; specificEpithet: kenyensis; scientificNameAuthorship: Henrard; **Location:** continent: Africa; country: Tanzania; stateProvince: Shinyanga; locality: Lake Magadi; verbatimLocality: NW side of Lake Magadi.; minimumElevationInMeters: 1432; decimalLatitude: -2.633333; decimalLongitude: 34.9; **Event:** eventDate: 1961-04-13; **Record Level:** institutionCode: SWRC; collectionCode: Herbarium; ownerInstitutionCode: SWRC; basisOfRecord: PreservedSpecimen
- **Type status:** Other material. **Occurrence:** catalogNumber: 804; recordNumber: 10327; recordedBy: Greenway, PJ; **Taxon:** scientificName: *Aristida kenyensis* Henrard; kingdom: Plantae; family: Poaceae; genus: Aristida; specificEpithet: kenyensis; scientificNameAuthorship: Henrard; **Location:** continent: Africa; country: Tanzania; stateProvince: Mara; county: Serengeti; locality: Musoma; verbatimLocality: Eastern boundary; decimalLatitude: -1.5; decimalLongitude: 34.683333; **Event:** eventDate: 1961-05-25; **Record Level:** institutionCode: EA; collectionCode: Herbarium; ownerInstitutionCode: EA; basisOfRecord: PreservedSpecimen
- **Type status:** Other material. **Occurrence:** catalogNumber: 805; recordNumber: 326; recordedBy: Paulo, S; **Taxon:** scientificName: *Aristida kenyensis* Henrard; kingdom: Plantae; family: Poaceae; genus: Aristida; specificEpithet: kenyensis; scientificNameAuthorship: Henrard; **Location:** continent: Africa; country: Tanzania; stateProvince: Arusha; county: Ngorongoro; locality: Olbalbal; decimalLatitude: -2.95; decimalLongitude: 35.283333; **Event:** eventDate: 1958-04-20; **Record Level:** institutionCode: EA; collectionCode: Herbarium; ownerInstitutionCode: EA; basisOfRecord: PreservedSpecimen
- **Type status:** Other material. **Occurrence:** catalogNumber: 806; recordNumber: 9091; recordedBy: Greenway, PJ; **Taxon:** scientificName: *Aristida kenyensis* Henrard; kingdom: Plantae; family: Poaceae; genus: Aristida; specificEpithet: kenyensis; scientificNameAuthorship: Henrard; **Location:** continent: Africa; country: Tanzania; stateProvince: Mara; county: Serengeti; locality: Seronera; verbatimLocality: Seronera to Soitayai, Mile 7.8; minimumElevationInMeters: 1590; decimalLatitude: -2.433333; decimalLongitude: 34.866667; **Event:** eventDate: 1961-03-27; **Record Level:** institutionCode: EA; collectionCode: Herbarium; ownerInstitutionCode: EA; basisOfRecord: PreservedSpecimen
- **Type status:** Other material. **Occurrence:** catalogNumber: 807; recordNumber: 193; recordedBy: Oteke, J; **Taxon:** scientificName: *Aristida kenyensis* Henrard; kingdom: Plantae; family: Poaceae; genus: Aristida; specificEpithet: kenyensis; scientificNameAuthorship: Henrard; **Location:** continent: Africa; country: Tanzania; stateProvince: Arusha; county: Ngorongoro; locality: Lemuta hill; verbatimLocality: East Serengeti; minimumElevationInMeters: 1524; decimalLatitude: -2.7; decimalLongitude: 35.266667; **Event:** eventDate: 1962-07-20; **Record Level:** institutionCode: EA; collectionCode: Herbarium; ownerInstitutionCode: EA; basisOfRecord: PreservedSpecimen

##### Distribution

Eastern Africa

#### Aristida mutabilis

Trin. & Rupr.

##### Materials

- **Type status:** Other material. **Occurrence:** catalogNumber: K000805171; recordNumber: 11349; recordedBy: Greenway, PJ; Hunter; **Taxon:** scientificName: *Aristida mutabilis* Trin. & Rupr.; kingdom: Plantae; family: Poaceae; genus: Aristida; specificEpithet: mutabilis; scientificNameAuthorship: Trin. & Rupr.; **Location:** continent: Africa; country: Tanzania; stateProvince: Arusha; county: Ngorongoro; locality: Ol Doinyo Lengai; verbatimLocality: Footsteps of Ol Doinyo Lengai; minimumElevationInMeters: 945; decimalLatitude: -2.75; decimalLongitude: 35.9; **Event:** eventDate: 1964-03-11; **Record Level:** institutionCode: K; collectionCode: Herbarium; ownerInstitutionCode: K; basisOfRecord: PreservedSpecimen
- **Type status:** Other material. **Occurrence:** catalogNumber: 855; recordNumber: 11349; recordedBy: Greenway, PJ; Hunter; **Taxon:** scientificName: *Aristida mutabilis* Trin. & Rupr.; kingdom: Plantae; family: Poaceae; genus: Aristida; specificEpithet: mutabilis; scientificNameAuthorship: Trin. & Rupr.; **Location:** continent: Africa; country: Tanzania; stateProvince: Arusha; county: Ngorongoro; locality: Ol Doinyo Lengai; verbatimLocality: Footsteps of Ol Doinyo Lengai; minimumElevationInMeters: 945; decimalLatitude: -2.75; decimalLongitude: 35.9; **Event:** eventDate: 1964-03-11; **Record Level:** institutionCode: EA; collectionCode: Herbarium; ownerInstitutionCode: EA; basisOfRecord: PreservedSpecimen

##### Distribution

Tropical Africa & Asia

#### Bothriochloa bladhii

(Retz.) S.T.Blake

Bothriochloa bladhii (Retz.) S.T.Blake var. bladhii

##### Materials

- **Type status:** Other material. **Occurrence:** catalogNumber: K000984062; recordNumber: 13167; recordedBy: Greenway, PJ; Kanuri; Braun, H; **Taxon:** scientificName: *Bothriochloa bladhii* (Retz.) S.T.Blake; kingdom: Plantae; family: Poaceae; genus: Bothriochloa; specificEpithet: bladhii; scientificNameAuthorship: (Retz.) S.T.Blake; **Location:** continent: Africa; country: Tanzania; stateProvince: Mara; county: Serengeti; locality: Grumeti River; verbatimLocality: W. of Musabi; minimumElevationInMeters: 1341; decimalLatitude: -1.9; decimalLongitude: 34.95; **Event:** eventDate: 1968-02-10; **Record Level:** institutionCode: K; collectionCode: Herbarium; ownerInstitutionCode: K; basisOfRecord: PreservedSpecimen
- **Type status:** Other material. **Occurrence:** catalogNumber: DB1140; recordNumber: 13167; recordedBy: Greenway, PJ; Kanuri; Braun, H; **Taxon:** scientificName: *Bothriochloa bladhii* (Retz.) S.T.Blake; kingdom: Plantae; family: Poaceae; genus: Bothriochloa; specificEpithet: bladhii; scientificNameAuthorship: (Retz.) S.T.Blake; **Location:** continent: Africa; country: Tanzania; stateProvince: Mara; county: Serengeti; locality: Grumeti River; verbatimLocality: W. of Musabi; minimumElevationInMeters: 1341; decimalLatitude: -1.9; decimalLongitude: 34.95; **Event:** eventDate: 1968-02-10; **Record Level:** institutionCode: SWRC; collectionCode: Herbarium; ownerInstitutionCode: SWRC; basisOfRecord: PreservedSpecimen
- **Type status:** Other material. **Occurrence:** catalogNumber: DB1141; recordNumber: 13167; recordedBy: Greenway, PJ; Kanuri; Braun, H; **Taxon:** scientificName: *Bothriochloa bladhii* (Retz.) S.T.Blake; kingdom: Plantae; family: Poaceae; genus: Bothriochloa; specificEpithet: bladhii; scientificNameAuthorship: (Retz.) S.T.Blake; **Location:** continent: Africa; country: Tanzania; stateProvince: Mara; county: Serengeti; locality: Grumeti River; verbatimLocality: W. of Musabi; minimumElevationInMeters: 1341; decimalLatitude: -1.9; decimalLongitude: 34.95; **Event:** eventDate: 1968-02-10; **Record Level:** institutionCode: SWRC; collectionCode: Herbarium; ownerInstitutionCode: SWRC; basisOfRecord: PreservedSpecimen

##### Distribution

Widespread

#### Bothriochloa insculpta

(Hochst. ex A.Rich.) A.Camus

##### Materials

- **Type status:** Other material. **Occurrence:** catalogNumber: K000984057; recordNumber: 355; recordedBy: Paulo, S; **Taxon:** scientificName: *Bothriochloa insculpta* (Hochst. ex A.Rich.) A.Camus; kingdom: Plantae; family: Poaceae; genus: Bothriochloa; specificEpithet: insculpta; scientificNameAuthorship: (Hochst. ex A.Rich.) A.Camus; **Location:** continent: Africa; country: Tanzania; stateProvince: Mara; county: Serengeti; locality: Seronera; verbatimLocality: near Rest Camp; decimalLatitude: -2.45; decimalLongitude: 34.833333; **Event:** eventDate: 1958-04-23; **Record Level:** institutionCode: K; collectionCode: Herbarium; ownerInstitutionCode: K; basisOfRecord: PreservedSpecimen
- **Type status:** Other material. **Occurrence:** catalogNumber: K000984058; recordNumber: 365; recordedBy: Paulo, S; **Taxon:** scientificName: *Bothriochloa insculpta* (Hochst. ex A.Rich.) A.Camus; kingdom: Plantae; family: Poaceae; genus: Bothriochloa; specificEpithet: insculpta; scientificNameAuthorship: (Hochst. ex A.Rich.) A.Camus; **Location:** continent: Africa; country: Tanzania; stateProvince: Mara; county: Serengeti; locality: Banagi Hill; decimalLatitude: -2.3; decimalLongitude: 34.833333; **Event:** eventDate: 1958-04-24; **Record Level:** institutionCode: K; collectionCode: Herbarium; ownerInstitutionCode: K; basisOfRecord: PreservedSpecimen
- **Type status:** Other material. **Occurrence:** catalogNumber: K000984059; recordNumber: 10048; recordedBy: Greenway, PJ; Tanner, M; **Taxon:** scientificName: *Bothriochloa insculpta* (Hochst. ex A.Rich.) A.Camus; kingdom: Plantae; family: Poaceae; genus: Bothriochloa; specificEpithet: insculpta; scientificNameAuthorship: (Hochst. ex A.Rich.) A.Camus; **Location:** continent: Africa; country: Tanzania; stateProvince: Shinyanga; locality: Lake Magadi; verbatimLocality: N.W. of Lake Magadi; minimumElevationInMeters: 1432; decimalLatitude: -2.633333; decimalLongitude: 34.9; **Event:** eventDate: 1961-04-13; **Record Level:** institutionCode: K; collectionCode: Herbarium; ownerInstitutionCode: K; basisOfRecord: PreservedSpecimen
- **Type status:** Other material. **Occurrence:** catalogNumber: DB1143; recordNumber: 173; recordedBy: Belsky, PJ; **Taxon:** scientificName: *Bothriochloa insculpta* (Hochst. ex A.Rich.) A.Camus; kingdom: Plantae; family: Poaceae; genus: Bothriochloa; specificEpithet: insculpta; scientificNameAuthorship: (Hochst. ex A.Rich.) A.Camus; **Location:** continent: Africa; country: Tanzania; stateProvince: Mara; county: Serengeti; locality: Serengeti Research Institute; verbatimLocality: Serengeti Research Institute compound; decimalLatitude: -2.433333; decimalLongitude: 34.85; **Event:** eventDate: 1981-05-11; **Record Level:** institutionCode: SWRC; collectionCode: Herbarium; ownerInstitutionCode: SWRC; basisOfRecord: PreservedSpecimen
- **Type status:** Other material. **Occurrence:** catalogNumber: DB1144; recordNumber: 194; recordedBy: Belsky, PJ; **Taxon:** scientificName: *Bothriochloa insculpta* (Hochst. ex A.Rich.) A.Camus; kingdom: Plantae; family: Poaceae; genus: Bothriochloa; specificEpithet: insculpta; scientificNameAuthorship: (Hochst. ex A.Rich.) A.Camus; **Location:** continent: Africa; country: Tanzania; stateProvince: Mara; county: Serengeti; locality: Serengeti Research Institute; verbatimLocality: Serengeti Research Institute compound; decimalLatitude: -2.433333; decimalLongitude: 34.85; **Event:** eventDate: 1981-05-11; **Record Level:** institutionCode: SWRC; collectionCode: Herbarium; ownerInstitutionCode: SWRC; basisOfRecord: PreservedSpecimen
- **Type status:** Other material. **Occurrence:** catalogNumber: 545; recordNumber: 373; recordedBy: Ellemann, L; **Taxon:** scientificName: *Bothriochloa insculpta* (Hochst. ex A.Rich.) A.Camus; kingdom: Plantae; family: Poaceae; genus: Bothriochloa; specificEpithet: insculpta; scientificNameAuthorship: (Hochst. ex A.Rich.) A.Camus; **Location:** continent: Africa; country: Tanzania; stateProvince: Arusha; county: Ngorongoro; locality: Endulen; verbatimLocality: Ngorongoro Conservation Area. Endulen Hospital.; minimumElevationInMeters: 1900; decimalLatitude: -3.183333; decimalLongitude: 35.15; **Event:** eventDate: 1993-04-20; **Record Level:** institutionCode: AAU; collectionCode: Herbarium; ownerInstitutionCode: AAU; basisOfRecord: PreservedSpecimen
- **Type status:** Other material. **Occurrence:** catalogNumber: 546; recordNumber: 375; recordedBy: Ellemann, L; **Taxon:** scientificName: *Bothriochloa insculpta* (Hochst. ex A.Rich.) A.Camus; kingdom: Plantae; family: Poaceae; genus: Bothriochloa; specificEpithet: insculpta; scientificNameAuthorship: (Hochst. ex A.Rich.) A.Camus; **Location:** continent: Africa; country: Tanzania; stateProvince: Arusha; county: Ngorongoro; locality: Endulen; verbatimLocality: Ngorongoro Conservation Area. Endulen Hospital.; minimumElevationInMeters: 1900; decimalLatitude: -3.183333; decimalLongitude: 35.15; **Event:** eventDate: 1993-04-20; **Record Level:** institutionCode: AAU; collectionCode: Herbarium; ownerInstitutionCode: AAU; basisOfRecord: PreservedSpecimen
- **Type status:** Other material. **Occurrence:** catalogNumber: 547; recordNumber: 24274; recordedBy: Peterson, PM; Soreng, RJ; Romaschenko, K; Mbago, F; **Taxon:** scientificName: *Bothriochloa insculpta* (Hochst. ex A.Rich.) A.Camus; kingdom: Plantae; family: Poaceae; genus: Bothriochloa; specificEpithet: insculpta; scientificNameAuthorship: (Hochst. ex A.Rich.) A.Camus; **Location:** continent: Africa; country: Tanzania; stateProvince: Shinyanga; locality: Naabi Hill Gate; verbatimLocality: Serengeti National Park, at 12 km NW of Naabi Hill Gate.; minimumElevationInMeters: 1651; decimalLatitude: -2.73597; decimalLongitude: 34.95284; **Event:** eventDate: 2012-06-16; **Record Level:** institutionCode: US; collectionCode: Herbarium; ownerInstitutionCode: US; basisOfRecord: PreservedSpecimen
- **Type status:** Other material. **Occurrence:** catalogNumber: DB1142; recordNumber: 10048; recordedBy: Greenway, PJ; Tanner, M; **Taxon:** scientificName: *Bothriochloa insculpta* (Hochst. ex A.Rich.) A.Camus; kingdom: Plantae; family: Poaceae; genus: Bothriochloa; specificEpithet: insculpta; scientificNameAuthorship: (Hochst. ex A.Rich.) A.Camus; **Location:** continent: Africa; country: Tanzania; stateProvince: Shinyanga; locality: Lake Magadi; verbatimLocality: N.W. of Lake Magadi; minimumElevationInMeters: 1432; decimalLatitude: -2.633333; decimalLongitude: 34.9; **Event:** eventDate: 1961-04-13; **Record Level:** institutionCode: SWRC; collectionCode: Herbarium; ownerInstitutionCode: SWRC; basisOfRecord: PreservedSpecimen

##### Distribution

Tropical Africa, Southern Africa, Arabia & Asia

#### Bothriochloa pertusa

(L.) A.Camus

##### Materials

- **Type status:** Other material. **Occurrence:** catalogNumber: DB1150; recordNumber: 273; recordedBy: Schmidt, W; **Taxon:** scientificName: *Bothriochloa pertusa* (L.) A.Camus; kingdom: Plantae; family: Poaceae; genus: Bothriochloa; specificEpithet: pertusa; scientificNameAuthorship: (L.) A.Camus; **Location:** continent: Africa; country: Tanzania; stateProvince: Mara; county: Serengeti; locality: Simba Kopjes; verbatimLocality: Simba Kopjes(North) Serengeti Plain; decimalLatitude: -2.7; decimalLongitude: 34.916667; **Event:** eventDate: 1972-04-10; **Record Level:** institutionCode: SWRC; collectionCode: Herbarium; ownerInstitutionCode: SWRC; basisOfRecord: PreservedSpecimen
- **Type status:** Other material. **Occurrence:** catalogNumber: DB1151; recordNumber: 102; recordedBy: Schmidt, W; **Taxon:** scientificName: *Bothriochloa pertusa* (L.) A.Camus; kingdom: Plantae; family: Poaceae; genus: Bothriochloa; specificEpithet: pertusa; scientificNameAuthorship: (L.) A.Camus; **Location:** continent: Africa; country: Tanzania; stateProvince: Mara; county: Serengeti; locality: Seronera; verbatimLocality: South of Seronera, Serengeti plain; decimalLatitude: -2.45; decimalLongitude: 34.833333; **Event:** eventDate: 1972-03-14; **Record Level:** institutionCode: SWRC; collectionCode: Herbarium; ownerInstitutionCode: SWRC; basisOfRecord: PreservedSpecimen
- **Type status:** Other material. **Occurrence:** catalogNumber: DB1152; recordNumber: 439; recordedBy: Schmidt, W; **Taxon:** scientificName: *Bothriochloa pertusa* (L.) A.Camus; kingdom: Plantae; family: Poaceae; genus: Bothriochloa; specificEpithet: pertusa; scientificNameAuthorship: (L.) A.Camus; **Location:** continent: Africa; country: Tanzania; stateProvince: Mara; county: Serengeti; locality: Bolgonja; verbatimLocality: Bologonja; decimalLatitude: -1.783333; decimalLongitude: 35.2; **Event:** eventDate: 1972-04-12; **Record Level:** institutionCode: SWRC; collectionCode: Herbarium; ownerInstitutionCode: SWRC; basisOfRecord: PreservedSpecimen

##### Distribution

Asia & Pacific, not previously recorded for East Africa

#### Bothriochloa radicans

(Lehm.) A.Camus

##### Materials

- **Type status:** Other material. **Occurrence:** catalogNumber: K000984060; recordNumber: 10378; recordedBy: Greenway, PJ; **Taxon:** scientificName: *Bothriochloa radicans* (Lehm.) A.Camus; kingdom: Plantae; family: Poaceae; genus: Bothriochloa; specificEpithet: radicans; scientificNameAuthorship: (Lehm.) A.Camus; **Location:** continent: Africa; country: Tanzania; stateProvince: Mara; county: Serengeti; locality: Ndabaka; decimalLatitude: -2.166667; decimalLongitude: 34.883333; **Event:** eventDate: 1961-06-14; **Record Level:** institutionCode: K; collectionCode: Herbarium; ownerInstitutionCode: K; basisOfRecord: PreservedSpecimen
- **Type status:** Other material. **Occurrence:** catalogNumber: K000984061; recordNumber: 9838; recordedBy: Greenway, PJ; **Taxon:** scientificName: *Bothriochloa radicans* (Lehm.) A.Camus; kingdom: Plantae; family: Poaceae; genus: Bothriochloa; specificEpithet: radicans; scientificNameAuthorship: (Lehm.) A.Camus; **Location:** continent: Africa; country: Tanzania; stateProvince: Mara; county: Serengeti; locality: Seronera; verbatimLocality: Serengeti; minimumElevationInMeters: 1554; decimalLatitude: -2.45; decimalLongitude: 34.833333; **Event:** eventDate: 1961-03-17; **Record Level:** institutionCode: K; collectionCode: Herbarium; ownerInstitutionCode: K; basisOfRecord: PreservedSpecimen
- **Type status:** Other material. **Occurrence:** catalogNumber: DB1155; recordNumber: 39; recordedBy: Schmidt, W; **Taxon:** scientificName: *Bothriochloa radicans* (Lehm.) A.Camus; kingdom: Plantae; family: Poaceae; genus: Bothriochloa; specificEpithet: radicans; scientificNameAuthorship: (Lehm.) A.Camus; **Location:** continent: Africa; country: Tanzania; stateProvince: Mara; county: Serengeti; locality: Simba Kopjes; verbatimLocality: Simba Kopjes Serengeti Plain; decimalLatitude: -2.7; decimalLongitude: 34.916667; **Event:** eventDate: 1972-03-11; **Record Level:** institutionCode: SWRC; collectionCode: Herbarium; ownerInstitutionCode: SWRC; basisOfRecord: PreservedSpecimen
- **Type status:** Other material. **Occurrence:** catalogNumber: DB1156; recordNumber: 2; recordedBy: Belsky, PJ; **Taxon:** scientificName: *Bothriochloa radicans* (Lehm.) A.Camus; kingdom: Plantae; family: Poaceae; genus: Bothriochloa; specificEpithet: radicans; scientificNameAuthorship: (Lehm.) A.Camus; **Location:** continent: Africa; country: Tanzania; stateProvince: Mara; county: Serengeti; locality: Serengeti Research Institute; verbatimLocality: Serengeti Research Institute compound; decimalLatitude: -2.433333; decimalLongitude: 34.85; **Event:** eventDate: 1980-01-21; **Record Level:** institutionCode: SWRC; collectionCode: Herbarium; ownerInstitutionCode: SWRC; basisOfRecord: PreservedSpecimen
- **Type status:** Other material. **Occurrence:** catalogNumber: 810; recordNumber: 10378; recordedBy: Greenway, PJ; **Taxon:** scientificName: *Bothriochloa radicans* (Lehm.) A.Camus; kingdom: Plantae; family: Poaceae; genus: Bothriochloa; specificEpithet: radicans; scientificNameAuthorship: (Lehm.) A.Camus; **Location:** continent: Africa; country: Tanzania; stateProvince: Mara; county: Serengeti; locality: Ndabaka; decimalLatitude: -2.166667; decimalLongitude: 34.883333; **Event:** eventDate: 1961-06-14; **Record Level:** institutionCode: EA; collectionCode: Herbarium; ownerInstitutionCode: EA; basisOfRecord: PreservedSpecimen
- **Type status:** Other material. **Occurrence:** catalogNumber: 811; recordNumber: 9838; recordedBy: Greenway, PJ; **Taxon:** scientificName: *Bothriochloa radicans* (Lehm.) A.Camus; kingdom: Plantae; family: Poaceae; genus: Bothriochloa; specificEpithet: radicans; scientificNameAuthorship: (Lehm.) A.Camus; **Location:** continent: Africa; country: Tanzania; stateProvince: Mara; county: Serengeti; locality: Seronera; verbatimLocality: Serengeti; minimumElevationInMeters: 1554; decimalLatitude: -2.45; decimalLongitude: 34.833333; **Event:** eventDate: 1961-03-17; **Record Level:** institutionCode: EA; collectionCode: Herbarium; ownerInstitutionCode: EA; basisOfRecord: PreservedSpecimen
- **Type status:** Other material. **Occurrence:** catalogNumber: DB1153; recordNumber: 9838; recordedBy: Greenway, PJ; **Taxon:** scientificName: *Bothriochloa radicans* (Lehm.) A.Camus; kingdom: Plantae; family: Poaceae; genus: Bothriochloa; specificEpithet: radicans; scientificNameAuthorship: (Lehm.) A.Camus; **Location:** continent: Africa; country: Tanzania; stateProvince: Mara; county: Serengeti; locality: Seronera; verbatimLocality: Serengeti; minimumElevationInMeters: 1554; decimalLatitude: -2.45; decimalLongitude: 34.833333; **Event:** eventDate: 1961-03-17; **Record Level:** institutionCode: SWRC; collectionCode: Herbarium; ownerInstitutionCode: SWRC; basisOfRecord: PreservedSpecimen
- **Type status:** Other material. **Occurrence:** catalogNumber: 813; recordNumber: 2; recordedBy: Belsky, PJ; **Taxon:** scientificName: *Bothriochloa radicans* (Lehm.) A.Camus; kingdom: Plantae; family: Poaceae; genus: Bothriochloa; specificEpithet: radicans; scientificNameAuthorship: (Lehm.) A.Camus; **Location:** continent: Africa; country: Tanzania; stateProvince: Mara; county: Serengeti; locality: Serengeti Research Institute; verbatimLocality: Serengeti Research Institute compound; decimalLatitude: -2.433333; decimalLongitude: 34.85; **Event:** eventDate: 1980-01-21; **Record Level:** institutionCode: EA; collectionCode: Herbarium; ownerInstitutionCode: EA; basisOfRecord: PreservedSpecimen
- **Type status:** Other material. **Occurrence:** catalogNumber: DB115; recordNumber: 10378; recordedBy: Greenway, PJ; **Taxon:** scientificName: *Bothriochloa radicans* (Lehm.) A.Camus; kingdom: Plantae; family: Poaceae; genus: Bothriochloa; specificEpithet: radicans; scientificNameAuthorship: (Lehm.) A.Camus; **Location:** continent: Africa; country: Tanzania; stateProvince: Mara; county: Serengeti; locality: Ndabaka; decimalLatitude: -2.166667; decimalLongitude: 34.883333; **Event:** eventDate: 1961-06-14; **Record Level:** institutionCode: SWRC; collectionCode: Herbarium; ownerInstitutionCode: SWRC; basisOfRecord: PreservedSpecimen
- **Type status:** Other material. **Occurrence:** catalogNumber: 993; recordNumber: CAWM5549; recordedBy: Mbano, BNN; William; **Taxon:** scientificName: *Bothriochloa radicans* (Lehm.) A.Camus; kingdom: Plantae; family: Poaceae; genus: Bothriochloa; specificEpithet: radicans; scientificNameAuthorship: (Lehm.) A.Camus; **Location:** continent: Africa; country: Tanzania; stateProvince: Mara; county: Serengeti; locality: Seronera plain; verbatimLocality: Serengeti National Park, T1; minimumElevationInMeters: 1524; decimalLatitude: -2.833333; decimalLongitude: 35; **Event:** eventDate: 1972-01-14; **Record Level:** institutionCode: EA; collectionCode: Herbarium; ownerInstitutionCode: EA; basisOfRecord: PreservedSpecimen

##### Distribution

Eastern & Southern Africa, Arabia,

#### Brachiaria sp.

##### Materials

- **Type status:** Other material. **Occurrence:** catalogNumber: 550; recordNumber: 1100; recordedBy: Ellemann, L; **Taxon:** scientificName: Brachiaria; kingdom: Plantae; family: Poaceae; genus: Brachiaria; **Location:** continent: Africa; country: Tanzania; stateProvince: Arusha; county: Ngorongoro; locality: Olbalbal swamp; verbatimLocality: Ngorongoro Conservation Area, south-western part of Olbalbal swamp.; minimumElevationInMeters: 1350; decimalLatitude: -3.05; decimalLongitude: 35.466; **Event:** eventDate: 1994-03-03; **Record Level:** institutionCode: AAU; collectionCode: Herbarium; ownerInstitutionCode: AAU; basisOfRecord: PreservedSpecimen
- **Type status:** Other material. **Occurrence:** catalogNumber: 551; recordNumber: 1101; recordedBy: Ellemann, L; **Taxon:** scientificName: Brachiaria; kingdom: Plantae; family: Poaceae; genus: Brachiaria; **Location:** continent: Africa; country: Tanzania; stateProvince: Arusha; county: Ngorongoro; locality: Olbalbal swamp; verbatimLocality: Ngorongoro Conservation Area, south-western part of Olbalbal swamp.; minimumElevationInMeters: 1350; decimalLatitude: -3.05; decimalLongitude: 35.466; **Event:** eventDate: 1994-03-03; **Record Level:** institutionCode: AAU; collectionCode: Herbarium; ownerInstitutionCode: AAU; basisOfRecord: PreservedSpecimen

#### Brachiaria ambigens

Chiov.

##### Materials

- **Type status:** Other material. **Occurrence:** catalogNumber: K000984127; recordNumber: 6361; recordedBy: Newbould, JB; **Taxon:** scientificName: *Brachiaria ambigens* Chiov.; kingdom: Plantae; family: Poaceae; genus: Brachiaria; specificEpithet: ambigens; scientificNameAuthorship: Chiov.; **Location:** continent: Africa; country: Tanzania; stateProvince: Arusha; county: Ngorongoro; locality: Olbalbal; minimumElevationInMeters: 1371; decimalLatitude: -2.95; decimalLongitude: 35.283333; **Event:** eventDate: 1962-12-10; **Record Level:** institutionCode: K; collectionCode: Herbarium; ownerInstitutionCode: K; basisOfRecord: PreservedSpecimen
- **Type status:** Other material. **Occurrence:** catalogNumber: 772; recordNumber: 6361; recordedBy: Newbould, JB; **Taxon:** scientificName: *Brachiaria ambigens* Chiov.; kingdom: Plantae; family: Poaceae; genus: Brachiaria; specificEpithet: ambigens; scientificNameAuthorship: Chiov.; **Location:** continent: Africa; country: Tanzania; stateProvince: Arusha; county: Ngorongoro; locality: Olbalbal; minimumElevationInMeters: 1371; decimalLatitude: -2.95; decimalLongitude: 35.283333; **Event:** eventDate: 1962-12-10; **Record Level:** institutionCode: EA; collectionCode: Herbarium; ownerInstitutionCode: EA; basisOfRecord: PreservedSpecimen

##### Distribution

Tanzania, Kenya & Ethiopia

#### Brachiaria arrecta

(T.Durand & Schinz) Stent

##### Materials

- **Type status:** Other material. **Occurrence:** catalogNumber: 548; recordNumber: 24275; recordedBy: Peterson, PM; Soreng, RJ; Romaschenko, K; Mbago, F; **Taxon:** scientificName: *Brachiaria arrecta* (T.Durand & Schinz) Stent; kingdom: Plantae; family: Poaceae; genus: Brachiaria; specificEpithet: arrecta; scientificNameAuthorship: (T.Durand & Schinz) Stent; **Location:** continent: Africa; country: Tanzania; stateProvince: Shinyanga; locality: Naabi Hill Gate; verbatimLocality: Serengeti National Park, at 12 km NW of Naabi Hill Gate.; minimumElevationInMeters: 1651; decimalLatitude: -2.73597; decimalLongitude: 34.95284; **Event:** eventDate: 2012-06-16; **Record Level:** institutionCode: US; collectionCode: Herbarium; ownerInstitutionCode: US; basisOfRecord: PreservedSpecimen
- **Type status:** Other material. **Occurrence:** catalogNumber: K001087089; recordNumber: CAWM5525; recordedBy: Mbano, BNN; **Taxon:** scientificName: *Brachiaria arrecta* (T.Durand & Schinz) Stent; kingdom: Plantae; family: Poaceae; genus: Brachiaria; specificEpithet: arrecta; scientificNameAuthorship: (T.Durand & Schinz) Stent; **Location:** continent: Africa; country: Tanzania; stateProvince: Mara; county: Serengeti; locality: Serengeti National Park; minimumElevationInMeters: 1524; decimalLatitude: -2.333333; decimalLongitude: 34.833333; **Event:** eventDate: 1972-01-14; **Record Level:** institutionCode: K; collectionCode: Herbarium; ownerInstitutionCode: K; basisOfRecord: PreservedSpecimen

##### Distribution

Eastern & Southern Africa

#### Brachiaria bovonei

(Chiov.) Robyns

##### Materials

- **Type status:** Other material. **Occurrence:** catalogNumber: K000984131; recordNumber: 10915; recordedBy: Greenway, PJ; Tanner, M; **Taxon:** scientificName: *Brachiaria bovonei* (Chiov.) Robyns; kingdom: Plantae; family: Poaceae; genus: Brachiaria; specificEpithet: bovonei; scientificNameAuthorship: (Chiov.) Robyns; **Location:** continent: Africa; country: Tanzania; stateProvince: Mara; county: Serengeti; locality: Tabora Air Strip; decimalLatitude: -1.816667; decimalLongitude: 34.633333; **Event:** eventDate: 1962-12-29; **Record Level:** institutionCode: K; collectionCode: Herbarium; ownerInstitutionCode: K; basisOfRecord: PreservedSpecimen

##### Distribution

Eastern & Southern Africa

#### Brachiaria brizantha

(A.Rich.) Stapf

##### Materials

- **Type status:** Other material. **Occurrence:** catalogNumber: K000984128; recordNumber: 10749; recordedBy: Greenway, PJ; **Taxon:** scientificName: *Brachiaria brizantha* (A.Rich.) Stapf; kingdom: Plantae; family: Poaceae; genus: Brachiaria; specificEpithet: brizantha; scientificNameAuthorship: (A.Rich.) Stapf; **Location:** continent: Africa; country: Tanzania; stateProvince: Mara; county: Serengeti; locality: Bolgonja river; minimumElevationInMeters: 1463; decimalLatitude: -1.783333; decimalLongitude: 35.2; **Event:** eventDate: 1962-08-17; **Record Level:** institutionCode: K; collectionCode: Herbarium; ownerInstitutionCode: K; basisOfRecord: PreservedSpecimen
- **Type status:** Other material. **Occurrence:** catalogNumber: DB1169; recordNumber: 10749; recordedBy: Greenway, PJ; **Taxon:** scientificName: *Brachiaria brizantha* (A.Rich.) Stapf; kingdom: Plantae; family: Poaceae; genus: Brachiaria; specificEpithet: brizantha; scientificNameAuthorship: (A.Rich.) Stapf; **Location:** continent: Africa; country: Tanzania; stateProvince: Mara; county: Serengeti; locality: Bolgonja river; minimumElevationInMeters: 1463; decimalLatitude: -1.783333; decimalLongitude: 35.2; **Event:** eventDate: 1962-08-17; **Record Level:** institutionCode: SWRC; collectionCode: Herbarium; ownerInstitutionCode: SWRC; basisOfRecord: PreservedSpecimen
- **Type status:** Other material. **Occurrence:** catalogNumber: DB1170; recordNumber: 690; recordedBy: Schmidt, W; **Taxon:** scientificName: *Brachiaria brizantha* (A.Rich.) Stapf; kingdom: Plantae; family: Poaceae; genus: Brachiaria; specificEpithet: brizantha; scientificNameAuthorship: (A.Rich.) Stapf; **Location:** continent: Africa; country: Tanzania; stateProvince: Mara; county: Serengeti; locality: Bolgonja; verbatimLocality: Bologonja; decimalLatitude: -1.783333; decimalLongitude: 35.2; **Event:** eventDate: 1972-04-21; **Record Level:** institutionCode: SWRC; collectionCode: Herbarium; ownerInstitutionCode: SWRC; basisOfRecord: PreservedSpecimen

##### Distribution

Tropical & Southern Africa, introduced elsewhere in the tropics

#### Brachiaria dictyoneura

(Fig. & De Not.) Stapf

Urochloa dictyoneura (Fig. & De Not.) Veldkamp

##### Materials

- **Type status:** Other material. **Occurrence:** catalogNumber: DB1185; recordNumber: 281; recordedBy: Belsky, PJ; **Taxon:** scientificName: *Brachiaria dictyoneura* (Fig. & De Not.) Stapf; kingdom: Plantae; family: Poaceae; genus: Brachiaria; specificEpithet: dictyoneura; scientificNameAuthorship: (Fig. & De Not.) Stapf; **Location:** continent: Africa; country: Tanzania; stateProvince: Mara; county: Serengeti; locality: Lobo Lodge; verbatimLocality: 5km north of Lobo Lodge; decimalLatitude: -1.933333; decimalLongitude: 35.166667; **Event:** eventDate: 1981-05-31; **Record Level:** institutionCode: SWRC; collectionCode: Herbarium; ownerInstitutionCode: SWRC; basisOfRecord: PreservedSpecimen
- **Type status:** Other material. **Occurrence:** catalogNumber: DB1186; recordNumber: 10669; recordedBy: Greenway, PJ; **Taxon:** scientificName: *Brachiaria dictyoneura* (Fig. & De Not.) Stapf; kingdom: Plantae; family: Poaceae; genus: Brachiaria; specificEpithet: dictyoneura; scientificNameAuthorship: (Fig. & De Not.) Stapf; **Location:** continent: Africa; country: Tanzania; stateProvince: Mara; county: Serengeti; locality: Serengeti National Park; verbatimLocality: Eastern boundary of the Park; minimumElevationInMeters: 1650; decimalLatitude: -2.333333; decimalLongitude: 34.833333; **Event:** eventDate: 1962-05-25; **Record Level:** institutionCode: SWRC; collectionCode: Herbarium; ownerInstitutionCode: SWRC; basisOfRecord: PreservedSpecimen
- **Type status:** Other material. **Occurrence:** catalogNumber: DB1189; recordNumber: 328; recordedBy: Schmidt, W; **Taxon:** scientificName: *Brachiaria dictyoneura* (Fig. & De Not.) Stapf; kingdom: Plantae; family: Poaceae; genus: Brachiaria; specificEpithet: dictyoneura; scientificNameAuthorship: (Fig. & De Not.) Stapf; **Location:** continent: Africa; country: Tanzania; stateProvince: Mara; county: Serengeti; locality: Bolgonja; verbatimLocality: Bologonja; decimalLatitude: -1.783333; decimalLongitude: 35.2; **Event:** eventDate: 1972-04-11; **Record Level:** institutionCode: SWRC; collectionCode: Herbarium; ownerInstitutionCode: SWRC; basisOfRecord: PreservedSpecimen
- **Type status:** Other material. **Occurrence:** catalogNumber: 549; recordNumber: 24284; recordedBy: Peterson, PM; Soreng, RJ; Romaschenko, K; Mbago, F; **Taxon:** scientificName: *Brachiaria dictyoneura* (Fig. & De Not.) Stapf; kingdom: Plantae; family: Poaceae; genus: Brachiaria; specificEpithet: dictyoneura; scientificNameAuthorship: (Fig. & De Not.) Stapf; **Location:** continent: Africa; country: Tanzania; stateProvince: Mara; county: Serengeti; locality: Mbuzi Mare camp; verbatimLocality: Serengeti National Park, near Mbuzi Mare camp.; minimumElevationInMeters: 1552; decimalLatitude: -2.23332; decimalLongitude: 34.96467; **Event:** eventDate: 2012-06-17; **Record Level:** institutionCode: US; collectionCode: Herbarium; ownerInstitutionCode: US; basisOfRecord: PreservedSpecimen
- **Type status:** Other material. **Occurrence:** catalogNumber: K000984129; recordNumber: 10771; recordedBy: Greenway, PJ; **Taxon:** scientificName: *Urochloa dictyoneura* (Fig. & De Not.) Veldkamp; kingdom: Plantae; family: Poaceae; genus: Urochloa; specificEpithet: dictyoneura; scientificNameAuthorship: (Fig. & De Not.) Veldkamp; **Location:** continent: Africa; country: Tanzania; stateProvince: Mara; county: Serengeti; locality: Mara River Guard Post; minimumElevationInMeters: 1280; decimalLatitude: -1.566667; decimalLongitude: 34.683333; **Event:** eventDate: 1962-08-21; **Record Level:** institutionCode: K; collectionCode: Herbarium; ownerInstitutionCode: K; basisOfRecord: PreservedSpecimen
- **Type status:** Other material. **Occurrence:** catalogNumber: K000984130; recordNumber: 10115; recordedBy: Greenway, PJ; **Taxon:** scientificName: *Urochloa dictyoneura* (Fig. & De Not.) Veldkamp; kingdom: Plantae; family: Poaceae; genus: Urochloa; specificEpithet: dictyoneura; scientificNameAuthorship: (Fig. & De Not.) Veldkamp; **Location:** continent: Africa; country: Tanzania; stateProvince: Mara; county: Serengeti; locality: Seronera; verbatimLocality: Seronera to Bolgonja river, Mile 30; minimumElevationInMeters: 1524; decimalLatitude: -2.45; decimalLongitude: 34.833333; **Event:** eventDate: 1961-04-29; **Record Level:** institutionCode: K; collectionCode: Herbarium; ownerInstitutionCode: K; basisOfRecord: PreservedSpecimen
- **Type status:** Other material. **Occurrence:** catalogNumber: DB1188; recordNumber: 10771; recordedBy: Greenway, PJ; **Taxon:** scientificName: *Urochloa dictyoneura* (Fig. & De Not.) Veldkamp; kingdom: Plantae; family: Poaceae; genus: Urochloa; specificEpithet: dictyoneura; scientificNameAuthorship: (Fig. & De Not.) Veldkamp; **Location:** continent: Africa; country: Tanzania; stateProvince: Mara; county: Serengeti; locality: Mara River Guard Post; minimumElevationInMeters: 1280; decimalLatitude: -1.566667; decimalLongitude: 34.683333; **Event:** eventDate: 1962-08-21; **Record Level:** institutionCode: SWRC; collectionCode: Herbarium; ownerInstitutionCode: SWRC; basisOfRecord: PreservedSpecimen
- **Type status:** Other material. **Occurrence:** catalogNumber: DB1187; recordNumber: 10115; recordedBy: Greenway, PJ; **Taxon:** scientificName: *Urochloa dictyoneura* (Fig. & De Not.) Veldkamp; kingdom: Plantae; family: Poaceae; genus: Urochloa; specificEpithet: dictyoneura; scientificNameAuthorship: (Fig. & De Not.) Veldkamp; **Location:** continent: Africa; country: Tanzania; stateProvince: Mara; county: Serengeti; locality: Seronera; verbatimLocality: Seronera to Bolgonja river, Mile 30; minimumElevationInMeters: 1524; decimalLatitude: -2.45; decimalLongitude: 34.833333; **Event:** eventDate: 1961-04-29; **Record Level:** institutionCode: SWRC; collectionCode: Herbarium; ownerInstitutionCode: SWRC; basisOfRecord: PreservedSpecimen

##### Distribution

Eastern & Southern Africa, introduced elsewhere

#### Brachiaria eruciformis

(Sm.) Griseb.

##### Materials

- **Type status:** Other material. **Occurrence:** catalogNumber: K000984124; recordNumber: 10334; recordedBy: Greenway, PJ; **Taxon:** scientificName: *Brachiaria eruciformis* (Sm.) Griseb.; kingdom: Plantae; family: Poaceae; genus: Brachiaria; specificEpithet: eruciformis; scientificNameAuthorship: (Sm.) Griseb.; **Location:** continent: Africa; country: Tanzania; stateProvince: Mara; county: Serengeti; locality: Seronera; verbatimLocality: Serengeti; minimumElevationInMeters: 1448; decimalLatitude: -2.45; decimalLongitude: 34.833333; **Event:** eventDate: 1961-05-27; **Record Level:** institutionCode: K; collectionCode: Herbarium; ownerInstitutionCode: K; basisOfRecord: PreservedSpecimen
- **Type status:** Other material. **Occurrence:** catalogNumber: K000984125; recordNumber: 2587; recordedBy: Chuwa, S; **Taxon:** scientificName: *Brachiaria eruciformis* (Sm.) Griseb.; kingdom: Plantae; family: Poaceae; genus: Brachiaria; specificEpithet: eruciformis; scientificNameAuthorship: (Sm.) Griseb.; **Location:** continent: Africa; country: Tanzania; stateProvince: Arusha; county: Ngorongoro; locality: Esere; verbatimLocality: near Kakesio; minimumElevationInMeters: 1600; decimalLatitude: -3.316667; decimalLongitude: 35.033333; **Event:** eventDate: 1987-04-29; **Record Level:** institutionCode: K; collectionCode: Herbarium; ownerInstitutionCode: K; basisOfRecord: PreservedSpecimen
- **Type status:** Other material. **Occurrence:** catalogNumber: DB1177; recordNumber: 9858; recordedBy: Greenway, PJ; **Taxon:** scientificName: *Brachiaria eruciformis* (Sm.) Griseb.; kingdom: Plantae; family: Poaceae; genus: Brachiaria; specificEpithet: eruciformis; scientificNameAuthorship: (Sm.) Griseb.; **Location:** continent: Africa; country: Tanzania; stateProvince: Mara; county: Serengeti; locality: Seronera; verbatimLocality: Seronera, Serengeti; minimumElevationInMeters: 1530; decimalLatitude: -2.45; decimalLongitude: 34.833333; **Event:** eventDate: 1961-03-20; **Record Level:** institutionCode: SWRC; collectionCode: Herbarium; ownerInstitutionCode: SWRC; basisOfRecord: PreservedSpecimen
- **Type status:** Other material. **Occurrence:** catalogNumber: DB1179; recordNumber: 10334; recordedBy: Greenway, PJ; **Taxon:** scientificName: *Brachiaria eruciformis* (Sm.) Griseb.; kingdom: Plantae; family: Poaceae; genus: Brachiaria; specificEpithet: eruciformis; scientificNameAuthorship: (Sm.) Griseb.; **Location:** continent: Africa; country: Tanzania; stateProvince: Mara; county: Serengeti; locality: Seronera; verbatimLocality: Serengeti; minimumElevationInMeters: 1448; decimalLatitude: -2.45; decimalLongitude: 34.833333; **Event:** eventDate: 1961-05-27; **Record Level:** institutionCode: SWRC; collectionCode: Herbarium; ownerInstitutionCode: SWRC; basisOfRecord: PreservedSpecimen
- **Type status:** Other material. **Occurrence:** catalogNumber: DB1178; recordNumber: 212; recordedBy: Schmidt, W; **Taxon:** scientificName: *Brachiaria eruciformis* (Sm.) Griseb.; kingdom: Plantae; family: Poaceae; genus: Brachiaria; specificEpithet: eruciformis; scientificNameAuthorship: (Sm.) Griseb.; **Location:** continent: Africa; country: Tanzania; stateProvince: Mara; county: Serengeti; locality: Serengeti Plains; verbatimLocality: South of Seronera, Serengeti plains; decimalLatitude: -2.333333; decimalLongitude: 34.833333; **Event:** eventDate: 1972-03-21; **Record Level:** institutionCode: SWRC; collectionCode: Herbarium; ownerInstitutionCode: SWRC; basisOfRecord: PreservedSpecimen

##### Distribution

Widespread in Europe, Arabia, Africa & Asia

#### Brachiaria leersioides

(Hochst.) Stapf

##### Materials

- **Type status:** Other material. **Occurrence:** catalogNumber: DB1198; recordNumber: 146; recordedBy: Belsky, PJ; **Taxon:** scientificName: *Brachiaria leersioides* (Hochst.) Stapf; kingdom: Plantae; family: Poaceae; genus: Brachiaria; specificEpithet: leersioides; scientificNameAuthorship: (Hochst.) Stapf; **Location:** continent: Africa; country: Tanzania; stateProvince: Mara; county: Serengeti; locality: Serengeti Research Institute; verbatimLocality: Serengeti Research Institute; decimalLatitude: -2.433333; decimalLongitude: 34.85; **Event:** eventDate: 1981-05-11; **Record Level:** institutionCode: SWRC; collectionCode: Herbarium; ownerInstitutionCode: SWRC; basisOfRecord: PreservedSpecimen
- **Type status:** Other material. **Occurrence:** catalogNumber: DB1201; recordNumber: 6577; recordedBy: Vesey-FitzGerald, LDEF; **Taxon:** scientificName: *Brachiaria leersioides* (Hochst.) Stapf; kingdom: Plantae; family: Poaceae; genus: Brachiaria; specificEpithet: leersioides; scientificNameAuthorship: (Hochst.) Stapf; **Location:** continent: Africa; country: Tanzania; stateProvince: Arusha; county: Ngorongoro; locality: Engaruka; verbatimLocality: Engaruka basin; minimumElevationInMeters: 870; decimalLatitude: -2.983333; decimalLongitude: 35.95; **Event:** eventDate: 1970-02-20; **Record Level:** institutionCode: SWRC; collectionCode: Herbarium; ownerInstitutionCode: SWRC; basisOfRecord: PreservedSpecimen

##### Distribution

Eastern Africa & Arabia

#### Brachiaria rugulosa

Stapf

##### Materials

- **Type status:** Other material. **Occurrence:** catalogNumber: DB1194; recordNumber: 325; recordedBy: Kreulen, AR; **Taxon:** scientificName: *Brachiaria rugulosa* Stapf; kingdom: Plantae; family: Poaceae; genus: Brachiaria; specificEpithet: rugulosa; scientificNameAuthorship: Stapf; **Location:** continent: Africa; country: Tanzania; stateProvince: Mara; county: Serengeti; locality: Simiyu; verbatimLocality: East Simiyu; decimalLatitude: -2.916667; decimalLongitude: 34.8; **Event:** eventDate: 1974-05-10; **Record Level:** institutionCode: SWRC; collectionCode: Herbarium; ownerInstitutionCode: SWRC; basisOfRecord: PreservedSpecimen
- **Type status:** Other material. **Occurrence:** catalogNumber: DB1195; recordNumber: 10645; recordedBy: Greenway, PJ; **Taxon:** scientificName: *Brachiaria rugulosa* Stapf; kingdom: Plantae; family: Poaceae; genus: Brachiaria; specificEpithet: rugulosa; scientificNameAuthorship: Stapf; **Location:** continent: Africa; country: Tanzania; stateProvince: Shinyanga; locality: Naabi Hill; verbatimLocality: Between Moru Kopjes and Naabi Hill Tracks, Central Plains, Serengeti; minimumElevationInMeters: 1425; decimalLatitude: -2.883333; decimalLongitude: 35.033333; **Event:** eventDate: 1962-05-18; **Record Level:** institutionCode: SWRC; collectionCode: Herbarium; ownerInstitutionCode: SWRC; basisOfRecord: PreservedSpecimen

##### Distribution

Tanzania, Kenya, Zambia & Zimbabwe

#### Brachiaria scalaris

(Mez.) Pilg.

##### Materials

- **Type status:** Other material. **Occurrence:** catalogNumber: 827; recordNumber: 5952; recordedBy: Newbould, JB; **Taxon:** scientificName: *Brachiaria scalaris* (Mez.) Pilg.; kingdom: Plantae; family: Poaceae; genus: Brachiaria; specificEpithet: scalaris; scientificNameAuthorship: (Mez.) Pilg.; **Location:** continent: Africa; country: Tanzania; stateProvince: Arusha; county: Ngorongoro; locality: Ngorongoro Crater; verbatimLocality: Olairadad, South side of Ngorongoro crater Rim; decimalLatitude: -3; decimalLongitude: 35.833333; **Event:** eventDate: 1962-02-02; **Record Level:** institutionCode: EA; collectionCode: Herbarium; ownerInstitutionCode: EA; basisOfRecord: PreservedSpecimen

##### Distribution

Tropical Africa & Arabia

#### Brachiaria semiundulata

(Hochst. ex A.Rich.) Stapf

##### Materials

- **Type status:** Other material. **Occurrence:** catalogNumber: K000984122; recordNumber: 13179; recordedBy: Greenway, PJ; Kanuri; **Taxon:** scientificName: *Brachiaria semiundulata* (Hochst. ex A.Rich.) Stapf; kingdom: Plantae; family: Poaceae; genus: Brachiaria; specificEpithet: semiundulata; scientificNameAuthorship: (Hochst. ex A.Rich.) Stapf; **Location:** continent: Africa; country: Tanzania; stateProvince: Shinyanga; locality: Moru Kopjes; minimumElevationInMeters: 1570; decimalLatitude: -2.75; decimalLongitude: 34.75; **Event:** eventDate: 1968-02-13; **Record Level:** institutionCode: K; collectionCode: Herbarium; ownerInstitutionCode: K; basisOfRecord: PreservedSpecimen
- **Type status:** Other material. **Occurrence:** catalogNumber: K000984123; recordNumber: 2753; recordedBy: Chuwa, S; **Taxon:** scientificName: *Brachiaria semiundulata* (Hochst. ex A.Rich.) Stapf; kingdom: Plantae; family: Poaceae; genus: Brachiaria; specificEpithet: semiundulata; scientificNameAuthorship: (Hochst. ex A.Rich.) Stapf; **Location:** continent: Africa; country: Tanzania; stateProvince: Arusha; county: Ngorongoro; locality: Mokilal; verbatimLocality: north east of Oldean Mtn.; minimumElevationInMeters: 2378; decimalLatitude: -3.266667; decimalLongitude: 35.433333; **Event:** eventDate: 1989-05-25; **Record Level:** institutionCode: K; collectionCode: Herbarium; ownerInstitutionCode: K; basisOfRecord: PreservedSpecimen
- **Type status:** Other material. **Occurrence:** catalogNumber: DB1206; recordNumber: 6591; recordedBy: Vesey-FitzGerald, LDEF; **Taxon:** scientificName: *Brachiaria semiundulata* (Hochst. ex A.Rich.) Stapf; kingdom: Plantae; family: Poaceae; genus: Brachiaria; specificEpithet: semiundulata; scientificNameAuthorship: (Hochst. ex A.Rich.) Stapf; **Location:** continent: Africa; country: Tanzania; stateProvince: Shinyanga; locality: Naabi Hill Gate; verbatimLocality: Serengeti National park, Naabi gate; decimalLatitude: -2.333333; decimalLongitude: 34.833333; **Record Level:** institutionCode: SWRC; collectionCode: Herbarium; ownerInstitutionCode: SWRC; basisOfRecord: PreservedSpecimen
- **Type status:** Other material. **Occurrence:** catalogNumber: DB1208; recordNumber: 15; recordedBy: Schmidt, W; **Taxon:** scientificName: *Brachiaria semiundulata* (Hochst. ex A.Rich.) Stapf; kingdom: Plantae; family: Poaceae; genus: Brachiaria; specificEpithet: semiundulata; scientificNameAuthorship: (Hochst. ex A.Rich.) Stapf; **Location:** continent: Africa; country: Tanzania; stateProvince: Mara; county: Serengeti; locality: Simba Kopjes; verbatimLocality: Simba Kopjes Serengeti Plain; decimalLatitude: -2.7; decimalLongitude: 34.916667; **Event:** eventDate: 1972-03-11; **Record Level:** institutionCode: SWRC; collectionCode: Herbarium; ownerInstitutionCode: SWRC; basisOfRecord: PreservedSpecimen
- **Type status:** Other material. **Occurrence:** catalogNumber: DB1209; recordNumber: 249; recordedBy: Braun, HMH; **Taxon:** scientificName: *Brachiaria semiundulata* (Hochst. ex A.Rich.) Stapf; kingdom: Plantae; family: Poaceae; genus: Brachiaria; specificEpithet: semiundulata; scientificNameAuthorship: (Hochst. ex A.Rich.) Stapf; **Location:** continent: Africa; country: Tanzania; stateProvince: Mara; county: Serengeti; locality: Downeys Dam; verbatimLocality: 1500 m South of Downeys Dam, west of road to Nzaki.; decimalLatitude: -2.333333; decimalLongitude: 34.833333; **Event:** eventDate: 1968-02-23; **Record Level:** institutionCode: SWRC; collectionCode: Herbarium; ownerInstitutionCode: SWRC; basisOfRecord: PreservedSpecimen
- **Type status:** Other material. **Occurrence:** catalogNumber: DB1210; recordNumber: 87; recordedBy: Schmidt, W; **Taxon:** scientificName: *Brachiaria semiundulata* (Hochst. ex A.Rich.) Stapf; kingdom: Plantae; family: Poaceae; genus: Brachiaria; specificEpithet: semiundulata; scientificNameAuthorship: (Hochst. ex A.Rich.) Stapf; **Location:** continent: Africa; country: Tanzania; stateProvince: Mara; county: Serengeti; locality: Seronera; verbatimLocality: South of Seronera Serengeti Plain; decimalLatitude: -2.45; decimalLongitude: 34.833333; **Event:** eventDate: 1972-03-13; **Record Level:** institutionCode: SWRC; collectionCode: Herbarium; ownerInstitutionCode: SWRC; basisOfRecord: PreservedSpecimen
- **Type status:** Other material. **Occurrence:** catalogNumber: 828; recordNumber: 5856; recordedBy: Newbould, JB; **Taxon:** scientificName: *Brachiaria semiundulata* (Hochst. ex A.Rich.) Stapf; kingdom: Plantae; family: Poaceae; genus: Brachiaria; specificEpithet: semiundulata; scientificNameAuthorship: (Hochst. ex A.Rich.) Stapf; **Location:** continent: Africa; country: Tanzania; stateProvince: Arusha; county: Ngorongoro; locality: Ngorongoro; decimalLatitude: -3; decimalLongitude: 35.833333; **Event:** eventDate: 1961-04-30; **Record Level:** institutionCode: EA; collectionCode: Herbarium; ownerInstitutionCode: EA; basisOfRecord: PreservedSpecimen
- **Type status:** Other material. **Occurrence:** catalogNumber: 1138; recordNumber: 855; recordedBy: Mollel, NP; Rusch, GM; Mwakalebe, G; **Taxon:** scientificName: *Brachiaria semiundulata* (Hochst. ex A.Rich.) Stapf; kingdom: Plantae; family: Poaceae; genus: Brachiaria; specificEpithet: semiundulata; scientificNameAuthorship: (Hochst. ex A.Rich.) Stapf; **Location:** continent: Africa; country: Tanzania; stateProvince: Mara; county: Serengeti; locality: Robanda village; verbatimLocality: Serengeti district, Robanda village, road to the water pool, CG01 plot. 36M, 0386088, 9763754 UTM.; minimumElevationInMeters: 1416; decimalLatitude: -2.083333; decimalLongitude: 34.666667; **Event:** eventDate: 2003-01-28; **Record Level:** institutionCode: NHT; collectionCode: Herbarium; ownerInstitutionCode: NHT; basisOfRecord: PreservedSpecimen
- **Type status:** Other material. **Occurrence:** catalogNumber: 1139; recordNumber: 885; recordedBy: Mollel, NP; Rusch, GM; Mwakalebe, G; **Taxon:** scientificName: *Brachiaria semiundulata* (Hochst. ex A.Rich.) Stapf; kingdom: Plantae; family: Poaceae; genus: Brachiaria; specificEpithet: semiundulata; scientificNameAuthorship: (Hochst. ex A.Rich.) Stapf; **Location:** continent: Africa; country: Tanzania; stateProvince: Mara; county: Serengeti; locality: Robanda village; verbatimLocality: Near Robanda Hill, 36M, 689097, 9763522 UTM.; minimumElevationInMeters: 1416; decimalLatitude: -2.083333; decimalLongitude: 34.666667; **Event:** eventDate: 2003-01-29; **Record Level:** institutionCode: NHT; collectionCode: Herbarium; ownerInstitutionCode: NHT; basisOfRecord: PreservedSpecimen
- **Type status:** Other material. **Occurrence:** catalogNumber: DB1203; recordNumber: 13179; recordedBy: Greenway, PJ; Kanuri; **Taxon:** scientificName: *Brachiaria semiundulata* (Hochst. ex A.Rich.) Stapf; kingdom: Plantae; family: Poaceae; genus: Brachiaria; specificEpithet: semiundulata; scientificNameAuthorship: (Hochst. ex A.Rich.) Stapf; **Location:** continent: Africa; country: Tanzania; stateProvince: Shinyanga; locality: Moru Kopjes; minimumElevationInMeters: 1570; decimalLatitude: -2.75; decimalLongitude: 34.75; **Event:** eventDate: 1968-02-13; **Record Level:** institutionCode: SWRC; collectionCode: Herbarium; ownerInstitutionCode: SWRC; basisOfRecord: PreservedSpecimen

##### Distribution

Eastern Africa & Asia

#### Brachiaria serrifolia

(Hochst.) Stapf

##### Materials

- **Type status:** Other material. **Occurrence:** catalogNumber: K000984126; recordNumber: 6380; recordedBy: Newbould, JB; **Taxon:** scientificName: *Brachiaria serrifolia* (Hochst.) Stapf; kingdom: Plantae; family: Poaceae; genus: Brachiaria; specificEpithet: serrifolia; scientificNameAuthorship: (Hochst.) Stapf; **Location:** continent: Africa; country: Tanzania; stateProvince: Arusha; county: Ngorongoro; locality: Olongogo; verbatimLocality: Olongogo; minimumElevationInMeters: 1371; decimalLatitude: -3; decimalLongitude: 35.333333; **Event:** eventDate: 1962-12-14; **Record Level:** institutionCode: K; collectionCode: Herbarium; ownerInstitutionCode: K; basisOfRecord: PreservedSpecimen
- **Type status:** Other material. **Occurrence:** catalogNumber: 820; recordNumber: 6380; recordedBy: Newbould, JB; **Taxon:** scientificName: *Brachiaria serrifolia* (Hochst.) Stapf; kingdom: Plantae; family: Poaceae; genus: Brachiaria; specificEpithet: serrifolia; scientificNameAuthorship: (Hochst.) Stapf; **Location:** continent: Africa; country: Tanzania; stateProvince: Arusha; county: Ngorongoro; locality: Olongogo; verbatimLocality: Olongogo; minimumElevationInMeters: 1371; decimalLatitude: -3; decimalLongitude: 35.333333; **Event:** eventDate: 1962-12-14; **Record Level:** institutionCode: EA; collectionCode: Herbarium; ownerInstitutionCode: EA; basisOfRecord: PreservedSpecimen

##### Distribution

Tropical Africa

#### Brachiaria subulifolia

(Mez) Clayton

##### Materials

- **Type status:** Other material. **Occurrence:** catalogNumber: DB1219; recordNumber: 578; recordedBy: Schmidt, W; **Taxon:** scientificName: *Brachiaria subulifolia* (Mez) Clayton; kingdom: Plantae; family: Poaceae; genus: Brachiaria; specificEpithet: subulifolia; scientificNameAuthorship: (Mez) Clayton; **Location:** continent: Africa; country: Tanzania; stateProvince: Mara; county: Serengeti; locality: Bolgonja; verbatimLocality: Bologonja; decimalLatitude: -1.783333; decimalLongitude: 35.2; **Event:** eventDate: 1972-04-18; **Record Level:** institutionCode: SWRC; collectionCode: Herbarium; ownerInstitutionCode: SWRC; basisOfRecord: PreservedSpecimen

##### Distribution

Eastern & Southern Africa

#### Brachiaria xantholeuca

(Hack.) Stapf

##### Materials

- **Type status:** Other material. **Occurrence:** catalogNumber: DB1221; recordNumber: 186; recordedBy: Belsky, PJ; **Taxon:** scientificName: *Brachiaria xantholeuca* (Hack.) Stapf; kingdom: Plantae; family: Poaceae; genus: Brachiaria; specificEpithet: xantholeuca; scientificNameAuthorship: (Hack.) Stapf; **Location:** continent: Africa; country: Tanzania; stateProvince: Mara; county: Serengeti; locality: Serengeti Research Institute; verbatimLocality: SRI tennis court; decimalLatitude: -2.433333; decimalLongitude: 34.85; **Event:** eventDate: 1981-05-11; **Record Level:** institutionCode: SWRC; collectionCode: Herbarium; ownerInstitutionCode: SWRC; basisOfRecord: PreservedSpecimen
- **Type status:** Other material. **Occurrence:** catalogNumber: DB1222; recordNumber: 250; recordedBy: Braun, HMH; **Taxon:** scientificName: *Brachiaria xantholeuca* (Hack.) Stapf; kingdom: Plantae; family: Poaceae; genus: Brachiaria; specificEpithet: xantholeuca; scientificNameAuthorship: (Hack.) Stapf; **Location:** continent: Africa; country: Tanzania; stateProvince: Mara; county: Serengeti; locality: Seronera; verbatimLocality: 2.5 km south of Downey's dam near Seronera.; minimumElevationInMeters: 1500; decimalLatitude: -2.45; decimalLongitude: 34.816667; **Event:** eventDate: 1968-02-23; **Record Level:** institutionCode: SWRC; collectionCode: Herbarium; ownerInstitutionCode: SWRC; basisOfRecord: PreservedSpecimen
- **Type status:** Other material. **Occurrence:** catalogNumber: 350; recordNumber: 250; recordedBy: Braun, HMH; **Taxon:** scientificName: *Brachiaria xantholeuca* (Hack.) Stapf; kingdom: Plantae; family: Poaceae; genus: Brachiaria; specificEpithet: xantholeuca; scientificNameAuthorship: (Hack.) Stapf; **Location:** continent: Africa; country: Tanzania; stateProvince: Mara; county: Serengeti; locality: Seronera; verbatimLocality: 2.5 km south of Downey's dam near Seronera.; minimumElevationInMeters: 1500; decimalLatitude: -2.45; decimalLongitude: 34.816667; **Event:** eventDate: 1968-02-23; **Record Level:** institutionCode: WAG; collectionCode: Herbarium; ownerInstitutionCode: WAG; basisOfRecord: PreservedSpecimen

##### Distribution

Tropical Africa & Arabia

#### Brachypodium flexum

Nees

##### Materials

- **Type status:** Other material. **Occurrence:** catalogNumber: DB1241; recordNumber: 504; recordedBy: Frame, GW; **Taxon:** scientificName: *Brachypodium flexum* Nees; kingdom: Plantae; family: Poaceae; genus: Brachypodium; specificEpithet: flexum; scientificNameAuthorship: Nees; **Location:** continent: Africa; country: Tanzania; stateProvince: Arusha; county: Ngorongoro; locality: Empakai Crater; verbatimLocality: Empakai Crater, outer southern slope,; minimumElevationInMeters: 2600; decimalLatitude: -2.933333; decimalLongitude: 35.816667; **Event:** eventDate: 1974-01-24; **Record Level:** institutionCode: SWRC; collectionCode: Herbarium; ownerInstitutionCode: SWRC; basisOfRecord: PreservedSpecimen

##### Distribution

Tropical Africa

#### Bromus leptoclados

Nees

##### Materials

- **Type status:** Other material. **Occurrence:** catalogNumber: 552; recordNumber: 768; recordedBy: Ellemann, L; **Taxon:** scientificName: *Bromus leptoclados* Nees; kingdom: Plantae; family: Poaceae; genus: Bromus; specificEpithet: leptoclados; scientificNameAuthorship: Nees; **Location:** continent: Africa; country: Tanzania; stateProvince: Arusha; county: Ngorongoro; locality: Oloronyo forest; verbatimLocality: Ngorongoro Conservation Area, Oloronyo Forest.; minimumElevationInMeters: 2650; decimalLatitude: -3.2; decimalLongitude: 35.35; **Event:** eventDate: 1993-08-05; **Record Level:** institutionCode: AAU; collectionCode: Herbarium; ownerInstitutionCode: AAU; basisOfRecord: PreservedSpecimen
- **Type status:** Other material. **Occurrence:** catalogNumber: K001087088; recordNumber: 3363; recordedBy: Greenway, PJ; **Taxon:** scientificName: *Bromus leptoclados* Nees; kingdom: Plantae; family: Poaceae; genus: Bromus; specificEpithet: leptoclados; scientificNameAuthorship: Nees; **Location:** continent: Africa; country: Tanzania; stateProvince: Arusha; county: Ngorongoro; locality: Ngorongoro Crater; verbatimLocality: S.E. slope of crater.; minimumElevationInMeters: 1829; maximumElevationInMeters: 2134; decimalLatitude: -3.166667; decimalLongitude: 35.583333; **Event:** eventDate: 1933-02-22; **Record Level:** institutionCode: K; collectionCode: Herbarium; ownerInstitutionCode: K; basisOfRecord: PreservedSpecimen
- **Type status:** Other material. **Occurrence:** catalogNumber: K001087084; recordNumber: 4362; recordedBy: Burtt, BD; **Taxon:** scientificName: *Bromus leptoclados* Nees; kingdom: Plantae; family: Poaceae; genus: Bromus; specificEpithet: leptoclados; scientificNameAuthorship: Nees; **Location:** continent: Africa; country: Tanzania; stateProvince: Arusha; county: Ngorongoro; locality: Olmoti crater; verbatimLocality: Munge gorge; minimumElevationInMeters: 2530; decimalLatitude: -3; decimalLongitude: 35.633333; **Event:** eventDate: 1932-09-16; **Record Level:** institutionCode: K; collectionCode: Herbarium; ownerInstitutionCode: K; basisOfRecord: PreservedSpecimen
- **Type status:** Other material. **Occurrence:** catalogNumber: K001087085; recordNumber: 9131; recordedBy: Greenway, PJ; **Taxon:** scientificName: *Bromus leptoclados* Nees; kingdom: Plantae; family: Poaceae; genus: Bromus; specificEpithet: leptoclados; scientificNameAuthorship: Nees; **Location:** continent: Africa; country: Tanzania; stateProvince: Arusha; county: Ngorongoro; locality: Empakai Crater; verbatimLocality: rim; minimumElevationInMeters: 3018; decimalLatitude: -2.933333; decimalLongitude: 35.816667; **Event:** eventDate: 1956-12-07; **Record Level:** institutionCode: K; collectionCode: Herbarium; ownerInstitutionCode: K; basisOfRecord: PreservedSpecimen
- **Type status:** Other material. **Occurrence:** catalogNumber: K001087086; recordNumber: 283; recordedBy: Staples, RR; **Taxon:** scientificName: *Bromus leptoclados* Nees; kingdom: Plantae; family: Poaceae; genus: Bromus; specificEpithet: leptoclados; scientificNameAuthorship: Nees; **Location:** continent: Africa; country: Tanzania; stateProvince: Arusha; county: Ngorongoro; locality: Ngorongoro; minimumElevationInMeters: 1371; decimalLatitude: -3; decimalLongitude: 35.5; **Event:** eventDate: 1933-07-08; **Record Level:** institutionCode: K; collectionCode: Herbarium; ownerInstitutionCode: K; basisOfRecord: PreservedSpecimen
- **Type status:** Other material. **Occurrence:** catalogNumber: K001087087; recordNumber: P58; recordedBy: Frame, GW; **Taxon:** scientificName: *Bromus leptoclados* Nees; kingdom: Plantae; family: Poaceae; genus: Bromus; specificEpithet: leptoclados; scientificNameAuthorship: Nees; **Location:** continent: Africa; country: Tanzania; stateProvince: Arusha; county: Ngorongoro; locality: Empakai Crater; verbatimLocality: Ngorongoro conservation area, Empakai, top of south rim elevation.; minimumElevationInMeters: 2900; decimalLatitude: -2.933333; decimalLongitude: 35.816667; **Event:** eventDate: 1973-05-27; **Record Level:** institutionCode: K; collectionCode: Herbarium; ownerInstitutionCode: K; basisOfRecord: PreservedSpecimen

##### Distribution

Eastern & Southern Africa

#### Calamagrostis epigejos

(L.) Roth

##### Materials

- **Type status:** Other material. **Occurrence:** catalogNumber: DB1258; recordNumber: 167; recordedBy: Frame, GW; **Taxon:** scientificName: *Calamagrostis epigejos* (L.) Roth; kingdom: Plantae; family: Poaceae; genus: Calamagrostis; specificEpithet: epigejos; scientificNameAuthorship: (L.) Roth; **Location:** continent: Africa; country: Tanzania; stateProvince: Arusha; county: Ngorongoro; locality: Empakai Crater; verbatimLocality: Empakai Crater top of North - west rim.; minimumElevationInMeters: 2900; decimalLatitude: -2.933333; decimalLongitude: 35.816667; **Event:** eventDate: 1973-06-12; **Record Level:** institutionCode: SWRC; collectionCode: Herbarium; ownerInstitutionCode: SWRC; basisOfRecord: PreservedSpecimen
- **Type status:** Other material. **Occurrence:** catalogNumber: 353; recordNumber: 167; recordedBy: Frame, GW; **Taxon:** scientificName: *Calamagrostis epigejos* (L.) Roth; kingdom: Plantae; family: Poaceae; genus: Calamagrostis; specificEpithet: epigejos; scientificNameAuthorship: (L.) Roth; **Location:** continent: Africa; country: Tanzania; stateProvince: Arusha; county: Ngorongoro; locality: Empakai Crater; verbatimLocality: Empakai Crater top of North - west rim.; minimumElevationInMeters: 2900; decimalLatitude: -2.933333; decimalLongitude: 35.816667; **Event:** eventDate: 1973-06-12; **Record Level:** institutionCode: EA; collectionCode: Herbarium; ownerInstitutionCode: EA; basisOfRecord: PreservedSpecimen

##### Distribution

Widespread

#### Cenchrus abyssinicus

(Hack.) Morrone

Odontelytrum abyssinicum Hack.

##### Materials

- **Type status:** Other material. **Occurrence:** catalogNumber: 936; recordNumber: 118; recordedBy: Goddard, J; **Taxon:** scientificName: *Odontelytrum abyssinicum* Hack.; kingdom: Plantae; family: Poaceae; genus: Odontelytrum; specificEpithet: abyssinicum; scientificNameAuthorship: Hack.; **Location:** continent: Africa; country: Tanzania; stateProvince: Arusha; county: Ngorongoro; locality: Ngorongoro Crater; decimalLatitude: -3.166667; decimalLongitude: 35.583333; **Event:** eventDate: 1965-06-07; **Record Level:** collectionCode: Herbarium; ownerInstitutionCode: ?; basisOfRecord: PreservedSpecimen
- **Type status:** Other material. **Occurrence:** catalogNumber: K001087115; recordNumber: 12617; recordedBy: Greenway, PJ; Kanuri; **Taxon:** scientificName: *Odontelytrum abyssinicum* Hack.; kingdom: Plantae; family: Poaceae; genus: Odontelytrum; specificEpithet: abyssinicum; scientificNameAuthorship: Hack.; **Location:** continent: Africa; country: Tanzania; stateProvince: Arusha; county: Ngorongoro; locality: Ngorongoro Crater; verbatimLocality: Olgoro Njuki, Ngorongoro crater floor.; minimumElevationInMeters: 1707; decimalLatitude: -3.166667; decimalLongitude: 35.583333; **Event:** eventDate: 1966-07-25; **Record Level:** institutionCode: K; collectionCode: Herbarium; ownerInstitutionCode: K; basisOfRecord: PreservedSpecimen
- **Type status:** Other material. **Occurrence:** catalogNumber: K001087116; recordNumber: 12617; recordedBy: Greenway, PJ; Kanuri; **Taxon:** scientificName: *Odontelytrum abyssinicum* Hack.; kingdom: Plantae; family: Poaceae; genus: Odontelytrum; specificEpithet: abyssinicum; scientificNameAuthorship: Hack.; **Location:** continent: Africa; country: Tanzania; stateProvince: Arusha; county: Ngorongoro; locality: Ngorongoro Crater; verbatimLocality: Olgoro Njuki, Ngorongoro crater floor.; minimumElevationInMeters: 1707; decimalLatitude: -3.166667; decimalLongitude: 35.583333; **Event:** eventDate: 1966-07-25; **Record Level:** institutionCode: K; collectionCode: Herbarium; ownerInstitutionCode: K; basisOfRecord: PreservedSpecimen

##### Distribution

Eastern Africa & Arabia

#### Cenchrus ciliaris

L.

##### Materials

- **Type status:** Other material. **Occurrence:** catalogNumber: 300; recordNumber: 2143B; recordedBy: Brown, ES; **Taxon:** scientificName: *Cenchrus ciliaris* L.; kingdom: Plantae; family: Poaceae; genus: Cenchrus; specificEpithet: ciliaris; scientificNameAuthorship: L.; **Location:** continent: Africa; country: Tanzania; stateProvince: Mara; county: Serengeti; locality: Banagi; verbatimLocality: Near Banagi.; decimalLatitude: -2.3; decimalLongitude: 34.833333; **Event:** eventDate: 1971-01-08; **Record Level:** institutionCode: EA; collectionCode: Herbarium; ownerInstitutionCode: EA; basisOfRecord: PreservedSpecimen
- **Type status:** Other material. **Occurrence:** catalogNumber: 302; recordNumber: 73/46; recordedBy: Bonnefille, R; **Taxon:** scientificName: *Cenchrus ciliaris* L.; kingdom: Plantae; family: Poaceae; genus: Cenchrus; specificEpithet: ciliaris; scientificNameAuthorship: L.; **Location:** continent: Africa; country: Tanzania; stateProvince: Mara; county: Serengeti; locality: Serengeti Research Institute; verbatimLocality: Near S.R.I; minimumElevationInMeters: 1600; decimalLatitude: -2.433333; decimalLongitude: 34.85; **Event:** eventDate: 1973-10-18; **Record Level:** institutionCode: EA; collectionCode: Herbarium; ownerInstitutionCode: EA; basisOfRecord: PreservedSpecimen
- **Type status:** Other material. **Occurrence:** catalogNumber: 303; recordNumber: 398; recordedBy: Paulo, S; **Taxon:** scientificName: *Cenchrus ciliaris* L.; kingdom: Plantae; family: Poaceae; genus: Cenchrus; specificEpithet: ciliaris; scientificNameAuthorship: L.; **Location:** continent: Africa; country: Tanzania; stateProvince: Mara; county: Serengeti; locality: Serengeti; decimalLatitude: -2.333333; decimalLongitude: 34.833333; **Event:** eventDate: 1958-04-28; **Record Level:** institutionCode: EA; collectionCode: Herbarium; ownerInstitutionCode: EA; basisOfRecord: PreservedSpecimen
- **Type status:** Other material. **Occurrence:** catalogNumber: 304; recordNumber: 4464; recordedBy: Vesey-FitzGerald, LDEF; **Taxon:** scientificName: *Cenchrus ciliaris* L.; kingdom: Plantae; family: Poaceae; genus: Cenchrus; specificEpithet: ciliaris; scientificNameAuthorship: L.; **Location:** continent: Africa; country: Tanzania; stateProvince: Mara; county: Serengeti; locality: Lake Lagarja; verbatimLocality: Lake Lagarja.; decimalLatitude: -3; decimalLongitude: 35.033333; **Event:** eventDate: 1964-12-15; **Record Level:** institutionCode: EA; collectionCode: Herbarium; ownerInstitutionCode: EA; basisOfRecord: PreservedSpecimen
- **Type status:** Other material. **Occurrence:** catalogNumber: DB1263; recordNumber: 1; recordedBy: Belsky, PJ; **Taxon:** scientificName: *Cenchrus ciliaris* L.; kingdom: Plantae; family: Poaceae; genus: Cenchrus; specificEpithet: ciliaris; scientificNameAuthorship: L.; **Location:** continent: Africa; country: Tanzania; stateProvince: Mara; county: Serengeti; locality: Serengeti Research Institute; verbatimLocality: Serengeti Research Institute Compound.; decimalLatitude: -2.433333; decimalLongitude: 34.85; **Event:** eventDate: 1980-01-21; **Record Level:** institutionCode: SWRC; collectionCode: Herbarium; ownerInstitutionCode: SWRC; basisOfRecord: PreservedSpecimen
- **Type status:** Other material. **Occurrence:** catalogNumber: DB1264; recordNumber: 116; recordedBy: Belsky, PJ; **Taxon:** scientificName: *Cenchrus ciliaris* L.; kingdom: Plantae; family: Poaceae; genus: Cenchrus; specificEpithet: ciliaris; scientificNameAuthorship: L.; **Location:** continent: Africa; country: Tanzania; stateProvince: Arusha; county: Ngorongoro; locality: Olduvai; verbatimLocality: Inside Leakey compound, Olduvai Gorge; decimalLatitude: -2.95; decimalLongitude: 35.166667; **Event:** eventDate: 1981-04-16; **Record Level:** institutionCode: SWRC; collectionCode: Herbarium; ownerInstitutionCode: SWRC; basisOfRecord: PreservedSpecimen
- **Type status:** Other material. **Occurrence:** catalogNumber: DB1265; recordNumber: 69; recordedBy: Belsky, PJ; **Taxon:** scientificName: *Cenchrus ciliaris* L.; kingdom: Plantae; family: Poaceae; genus: Cenchrus; specificEpithet: ciliaris; scientificNameAuthorship: L.; **Location:** continent: Africa; country: Tanzania; stateProvince: Arusha; county: Ngorongoro; locality: Ntudu Lodge; verbatimLocality: Near road 50 m from Ndutu lodge.; decimalLatitude: -3.02; decimalLongitude: 34.996944; **Event:** eventDate: 1981-04-10; **Record Level:** institutionCode: SWRC; collectionCode: Herbarium; ownerInstitutionCode: SWRC; basisOfRecord: PreservedSpecimen
- **Type status:** Other material. **Occurrence:** catalogNumber: DB1266; recordNumber: 165; recordedBy: Belsky, PJ; **Taxon:** scientificName: *Cenchrus ciliaris* L.; kingdom: Plantae; family: Poaceae; genus: Cenchrus; specificEpithet: ciliaris; scientificNameAuthorship: L.; **Location:** continent: Africa; country: Tanzania; stateProvince: Mara; county: Serengeti; locality: Serengeti Research Institute; verbatimLocality: Serengeti Research Institute Compound.; decimalLatitude: -2.433333; decimalLongitude: 34.85; **Event:** eventDate: 1981-05-11; **Record Level:** institutionCode: SWRC; collectionCode: Herbarium; ownerInstitutionCode: SWRC; basisOfRecord: PreservedSpecimen
- **Type status:** Other material. **Occurrence:** catalogNumber: DB1269; recordNumber: 4464; recordedBy: Vesey-FitzGerald, LDEF; **Taxon:** scientificName: *Cenchrus ciliaris* L.; kingdom: Plantae; family: Poaceae; genus: Cenchrus; specificEpithet: ciliaris; scientificNameAuthorship: L.; **Location:** continent: Africa; country: Tanzania; stateProvince: Mara; county: Serengeti; locality: Lake Lagarja; verbatimLocality: Lake Lagarja.; decimalLatitude: -3; decimalLongitude: 35.033333; **Event:** eventDate: 1964-12-15; **Record Level:** institutionCode: SWRC; collectionCode: Herbarium; ownerInstitutionCode: SWRC; basisOfRecord: PreservedSpecimen
- **Type status:** Other material. **Occurrence:** catalogNumber: DB1270; recordNumber: 9856; recordedBy: Greenway, PJ; **Taxon:** scientificName: *Cenchrus ciliaris* L.; kingdom: Plantae; family: Poaceae; genus: Cenchrus; specificEpithet: ciliaris; scientificNameAuthorship: L.; **Location:** continent: Africa; country: Tanzania; stateProvince: Mara; county: Serengeti; locality: Seronera; verbatimLocality: T1, Seronera, Serengeti.; minimumElevationInMeters: 1530; decimalLatitude: -2.45; decimalLongitude: 34.833333; **Event:** eventDate: 1961-03-20; **Record Level:** institutionCode: SWRC; collectionCode: Herbarium; ownerInstitutionCode: SWRC; basisOfRecord: PreservedSpecimen
- **Type status:** Other material. **Occurrence:** catalogNumber: DB1271; recordNumber: 9955; recordedBy: Greenway, PJ; **Taxon:** scientificName: *Cenchrus ciliaris* L.; kingdom: Plantae; family: Poaceae; genus: Cenchrus; specificEpithet: ciliaris; scientificNameAuthorship: L.; **Location:** continent: Africa; country: Tanzania; stateProvince: Mara; county: Serengeti; locality: Seronera; verbatimLocality: T1, Seronera to Banagi, Mile 3.8,; minimumElevationInMeters: 1500; decimalLatitude: -2.45; decimalLongitude: 34.833333; **Event:** eventDate: 1961-04-03; **Record Level:** institutionCode: SWRC; collectionCode: Herbarium; ownerInstitutionCode: SWRC; basisOfRecord: PreservedSpecimen
- **Type status:** Other material. **Occurrence:** catalogNumber: DB1272; recordNumber: 5454; recordedBy: Harris, BJ; **Taxon:** scientificName: *Cenchrus ciliaris* L.; kingdom: Plantae; family: Poaceae; genus: Cenchrus; specificEpithet: ciliaris; scientificNameAuthorship: L.; **Location:** continent: Africa; country: Tanzania; stateProvince: Mara; county: Serengeti; locality: Simba Kopjes; verbatimLocality: T1 Serengeti national Park Simba Kopje; minimumElevationInMeters: 1700; decimalLatitude: -2.7; decimalLongitude: 34.916667; **Event:** eventDate: 1970-12-08; **Record Level:** institutionCode: SWRC; collectionCode: Herbarium; ownerInstitutionCode: SWRC; basisOfRecord: PreservedSpecimen
- **Type status:** Other material. **Occurrence:** catalogNumber: DB1273; recordNumber: 250; recordedBy: Schmidt, W; **Taxon:** scientificName: *Cenchrus ciliaris* L.; kingdom: Plantae; family: Poaceae; genus: Cenchrus; specificEpithet: ciliaris; scientificNameAuthorship: L.; **Location:** continent: Africa; country: Tanzania; stateProvince: Arusha; county: Ngorongoro; locality: Gol Kopjes; verbatimLocality: Gol Kopjes.; decimalLatitude: -2.833333; decimalLongitude: 35; **Event:** eventDate: 1972-03-30; **Record Level:** institutionCode: SWRC; collectionCode: Herbarium; ownerInstitutionCode: SWRC; basisOfRecord: PreservedSpecimen
- **Type status:** Other material. **Occurrence:** catalogNumber: DB1274; recordNumber: 416; recordedBy: Schmidt, W; **Taxon:** scientificName: *Cenchrus ciliaris* L.; kingdom: Plantae; family: Poaceae; genus: Cenchrus; specificEpithet: ciliaris; scientificNameAuthorship: L.; **Location:** continent: Africa; country: Tanzania; stateProvince: Mara; county: Serengeti; locality: Bolgonja; verbatimLocality: Bologonja; decimalLatitude: -1.783333; decimalLongitude: 35.2; **Event:** eventDate: 1972-04-12; **Record Level:** institutionCode: SWRC; collectionCode: Herbarium; ownerInstitutionCode: SWRC; basisOfRecord: PreservedSpecimen
- **Type status:** Other material. **Occurrence:** catalogNumber: 553; recordNumber: s.n.; recordedBy: Mboya, E; **Taxon:** scientificName: *Cenchrus ciliaris* L.; kingdom: Plantae; family: Poaceae; genus: Cenchrus; specificEpithet: ciliaris; scientificNameAuthorship: L.; **Location:** continent: Africa; country: Tanzania; stateProvince: Mara; county: Serengeti; locality: Serengeti National Park; verbatimLocality: T1. Serengeti National Park.; minimumElevationInMeters: 1466; decimalLatitude: -2.36; decimalLongitude: 34.5; **Event:** eventDate: 2004-03-06; **Record Level:** institutionCode: MO; collectionCode: Herbarium; ownerInstitutionCode: MO; basisOfRecord: PreservedSpecimen
- **Type status:** Other material. **Occurrence:** catalogNumber: 554; recordNumber: 24281; recordedBy: Peterson, PM; Soreng, RJ; Romaschenko, K; Mbago, F; **Taxon:** scientificName: *Cenchrus ciliaris* L.; kingdom: Plantae; family: Poaceae; genus: Cenchrus; specificEpithet: ciliaris; scientificNameAuthorship: L.; **Location:** continent: Africa; country: Tanzania; stateProvince: Mara; county: Serengeti; locality: Mbuzi Mare camp; verbatimLocality: Serengeti National Park, near Mbuzi Mare camp.; minimumElevationInMeters: 1552; decimalLatitude: -2.23332; decimalLongitude: 34.96467; **Event:** eventDate: 2012-06-17; **Record Level:** institutionCode: US; collectionCode: Herbarium; ownerInstitutionCode: US; basisOfRecord: PreservedSpecimen
- **Type status:** Other material. **Occurrence:** catalogNumber: 761; recordNumber: 9856; recordedBy: Greenway, PJ; **Taxon:** scientificName: *Cenchrus ciliaris* L.; kingdom: Plantae; family: Poaceae; genus: Cenchrus; specificEpithet: ciliaris; scientificNameAuthorship: L.; **Location:** continent: Africa; country: Tanzania; stateProvince: Mara; county: Serengeti; locality: Seronera; verbatimLocality: T1, Seronera, Serengeti.; minimumElevationInMeters: 1530; decimalLatitude: -2.45; decimalLongitude: 34.833333; **Event:** eventDate: 1961-03-20; **Record Level:** institutionCode: EA; collectionCode: Herbarium; ownerInstitutionCode: EA; basisOfRecord: PreservedSpecimen
- **Type status:** Other material. **Occurrence:** catalogNumber: K000748227; recordNumber: 9955; recordedBy: Greenway, PJ; **Taxon:** scientificName: *Cenchrus ciliaris* L.; kingdom: Plantae; family: Poaceae; genus: Cenchrus; specificEpithet: ciliaris; scientificNameAuthorship: L.; **Location:** continent: Africa; country: Tanzania; stateProvince: Mara; county: Serengeti; locality: Seronera; verbatimLocality: T1, Seronera to Banagi, Mile 3.8,; minimumElevationInMeters: 1500; decimalLatitude: -2.45; decimalLongitude: 34.833333; **Event:** eventDate: 1961-04-03; **Record Level:** institutionCode: K; collectionCode: Herbarium; ownerInstitutionCode: K; basisOfRecord: PreservedSpecimen
- **Type status:** Other material. **Occurrence:** catalogNumber: K000748224; recordNumber: 9856; recordedBy: Greenway, PJ; **Taxon:** scientificName: *Cenchrus ciliaris* L.; kingdom: Plantae; family: Poaceae; genus: Cenchrus; specificEpithet: ciliaris; scientificNameAuthorship: L.; **Location:** continent: Africa; country: Tanzania; stateProvince: Mara; county: Serengeti; locality: Seronera; verbatimLocality: T1, Seronera, Serengeti.; minimumElevationInMeters: 1530; decimalLatitude: -2.45; decimalLongitude: 34.833333; **Event:** eventDate: 1961-03-20; **Record Level:** institutionCode: K; collectionCode: Herbarium; ownerInstitutionCode: K; basisOfRecord: PreservedSpecimen
- **Type status:** Other material. **Occurrence:** catalogNumber: 818; recordNumber: 9955; recordedBy: Greenway, PJ; **Taxon:** scientificName: *Cenchrus ciliaris* L.; kingdom: Plantae; family: Poaceae; genus: Cenchrus; specificEpithet: ciliaris; scientificNameAuthorship: L.; **Location:** continent: Africa; country: Tanzania; stateProvince: Mara; county: Serengeti; locality: Seronera; verbatimLocality: T1, Seronera to Banagi, Mile 3.8,; minimumElevationInMeters: 1500; decimalLatitude: -2.45; decimalLongitude: 34.833333; **Event:** eventDate: 1961-04-03; **Record Level:** institutionCode: EA; collectionCode: Herbarium; ownerInstitutionCode: EA; basisOfRecord: PreservedSpecimen
- **Type status:** Other material. **Occurrence:** catalogNumber: K000748168; recordNumber: 344; recordedBy: Thompson, IH; **Taxon:** scientificName: *Cenchrus ciliaris* L.; kingdom: Plantae; family: Poaceae; genus: Cenchrus; specificEpithet: ciliaris; scientificNameAuthorship: L.; **Location:** continent: Africa; country: Tanzania; stateProvince: Arusha; county: Ngorongoro; locality: Empakai Mt; verbatimLocality: Embagai Mt; West Arusha; minimumElevationInMeters: 1981; decimalLatitude: -2.933333; decimalLongitude: 35.816667; **Event:** eventDate: 1932-02-06; **Record Level:** institutionCode: K; collectionCode: Herbarium; ownerInstitutionCode: K; basisOfRecord: PreservedSpecimen
- **Type status:** Other material. **Occurrence:** catalogNumber: K000748194; recordNumber: 19306; recordedBy: Leakey, MD; **Taxon:** scientificName: *Cenchrus ciliaris* L.; kingdom: Plantae; family: Poaceae; genus: Cenchrus; specificEpithet: ciliaris; scientificNameAuthorship: L.; **Location:** continent: Africa; country: Tanzania; stateProvince: Arusha; county: Ngorongoro; locality: Olduvai; verbatimLocality: Ngorongoro Conservation Area: Olduvai. shifting sands 5-6km NW of confluence of Main and side Gorges; minimumElevationInMeters: 1580; decimalLatitude: -2.95; decimalLongitude: 35.166667; **Event:** eventDate: 1977-09-27; **Record Level:** institutionCode: K; collectionCode: Herbarium; ownerInstitutionCode: K; basisOfRecord: PreservedSpecimen
- **Type status:** Other material. **Occurrence:** catalogNumber: K000748218; recordNumber: 336; recordedBy: Paulo, S; **Taxon:** scientificName: *Cenchrus ciliaris* L.; kingdom: Plantae; family: Poaceae; genus: Cenchrus; specificEpithet: ciliaris; scientificNameAuthorship: L.; **Location:** continent: Africa; country: Tanzania; stateProvince: Mara; county: Serengeti; locality: Serengeti; minimumElevationInMeters: 1341; decimalLatitude: -2.333333; decimalLongitude: 34.833333; **Event:** eventDate: 1958-04-21; **Record Level:** institutionCode: K; collectionCode: Herbarium; ownerInstitutionCode: K; basisOfRecord: PreservedSpecimen
- **Type status:** Other material. **Occurrence:** catalogNumber: 850; recordNumber: 336; recordedBy: Paulo, S; **Taxon:** scientificName: *Cenchrus ciliaris* L.; kingdom: Plantae; family: Poaceae; genus: Cenchrus; specificEpithet: ciliaris; scientificNameAuthorship: L.; **Location:** continent: Africa; country: Tanzania; stateProvince: Mara; county: Serengeti; locality: Serengeti; minimumElevationInMeters: 1341; decimalLatitude: -2.333333; decimalLongitude: 34.833333; **Event:** eventDate: 1958-04-21; **Record Level:** institutionCode: EA; collectionCode: Herbarium; ownerInstitutionCode: EA; basisOfRecord: PreservedSpecimen
- **Type status:** Other material. **Occurrence:** catalogNumber: 994; recordNumber: 5725; recordedBy: Newbould, JB; **Taxon:** scientificName: *Cenchrus ciliaris* L.; kingdom: Plantae; family: Poaceae; genus: Cenchrus; specificEpithet: ciliaris; scientificNameAuthorship: L.; **Location:** continent: Africa; country: Tanzania; stateProvince: Arusha; county: Ngorongoro; locality: Olbalbal; verbatimLocality: Olbalbal escarpment; minimumElevationInMeters: 1524; decimalLatitude: -3.083333; decimalLongitude: 35.416667; **Event:** eventDate: 1961-03-18; **Record Level:** institutionCode: EA; collectionCode: Herbarium; ownerInstitutionCode: EA; basisOfRecord: PreservedSpecimen
- **Type status:** Other material. **Occurrence:** catalogNumber: 995; recordNumber: 5725; recordedBy: Newbould, JB; **Taxon:** scientificName: *Cenchrus ciliaris* L.; kingdom: Plantae; family: Poaceae; genus: Cenchrus; specificEpithet: ciliaris; scientificNameAuthorship: L.; **Location:** continent: Africa; country: Tanzania; stateProvince: Arusha; county: Ngorongoro; locality: Olbalbal; verbatimLocality: Olbalbal escarpment; minimumElevationInMeters: 1524; decimalLatitude: -3.083333; decimalLongitude: 35.416667; **Event:** eventDate: 1961-03-18; **Record Level:** institutionCode: EA; collectionCode: Herbarium; ownerInstitutionCode: EA; basisOfRecord: PreservedSpecimen
- **Type status:** Other material. **Occurrence:** catalogNumber: 996; recordNumber: 5931; recordedBy: Newbould, JB; **Taxon:** scientificName: *Cenchrus ciliaris* L.; kingdom: Plantae; family: Poaceae; genus: Cenchrus; specificEpithet: ciliaris; scientificNameAuthorship: L.; **Location:** continent: Africa; country: Tanzania; stateProvince: Arusha; county: Ngorongoro; locality: Olbalbal; minimumElevationInMeters: 1417; decimalLatitude: -2.95; decimalLongitude: 35.583333; **Event:** eventDate: 1962-01-30; **Record Level:** institutionCode: EA; collectionCode: Herbarium; ownerInstitutionCode: EA; basisOfRecord: PreservedSpecimen
- **Type status:** Other material. **Occurrence:** catalogNumber: 997; recordNumber: 5931; recordedBy: Newbould, JB; **Taxon:** scientificName: *Cenchrus ciliaris* L.; kingdom: Plantae; family: Poaceae; genus: Cenchrus; specificEpithet: ciliaris; scientificNameAuthorship: L.; **Location:** continent: Africa; country: Tanzania; stateProvince: Arusha; county: Ngorongoro; locality: Olbalbal; minimumElevationInMeters: 1417; decimalLatitude: -2.95; decimalLongitude: 35.583333; **Event:** eventDate: 1962-01-30; **Record Level:** institutionCode: EA; collectionCode: Herbarium; ownerInstitutionCode: EA; basisOfRecord: PreservedSpecimen
- **Type status:** Other material. **Occurrence:** catalogNumber: 998; recordNumber: 12; recordedBy: Jacob, J; **Taxon:** scientificName: *Cenchrus ciliaris* L.; kingdom: Plantae; family: Poaceae; genus: Cenchrus; specificEpithet: ciliaris; scientificNameAuthorship: L.; **Location:** continent: Africa; country: Tanzania; stateProvince: Mara; county: Serengeti; locality: Seronera camp; decimalLatitude: -2.333333; decimalLongitude: 34.833333; **Event:** eventDate: 1959-12-22; **Record Level:** institutionCode: EA; collectionCode: Herbarium; ownerInstitutionCode: EA; basisOfRecord: PreservedSpecimen
- **Type status:** Other material. **Occurrence:** catalogNumber: 999; recordNumber: 253; recordedBy: Goddard, J; **Taxon:** scientificName: *Cenchrus ciliaris* L.; kingdom: Plantae; family: Poaceae; genus: Cenchrus; specificEpithet: ciliaris; scientificNameAuthorship: L.; **Location:** continent: Africa; country: Tanzania; stateProvince: Arusha; county: Ngorongoro; locality: Olduvai Gorge; minimumElevationInMeters: 1554; decimalLatitude: -2.95; decimalLongitude: 35.166667; **Event:** eventDate: 1966-02-16; **Record Level:** institutionCode: EA; collectionCode: Herbarium; ownerInstitutionCode: EA; basisOfRecord: PreservedSpecimen
- **Type status:** Other material. **Occurrence:** catalogNumber: 1145; recordNumber: 883; recordedBy: Mollel, NP; Rusch, GM; Mwakalebe, G; **Taxon:** scientificName: *Cenchrus ciliaris* L.; kingdom: Plantae; family: Poaceae; genus: Cenchrus; specificEpithet: ciliaris; scientificNameAuthorship: L.; **Location:** continent: Africa; country: Tanzania; stateProvince: Mara; county: Serengeti; locality: Robanda village; verbatimLocality: Near Robanda Hill, 36M, 689097, 9763522 UTM.; minimumElevationInMeters: 1416; decimalLatitude: -2.083333; decimalLongitude: 34.666667; **Event:** eventDate: 2003-01-29; **Record Level:** institutionCode: NHT; collectionCode: Herbarium; ownerInstitutionCode: NHT; basisOfRecord: PreservedSpecimen
- **Type status:** Other material. **Occurrence:** catalogNumber: 1146; recordNumber: 1111; recordedBy: Macha; Leliyo, G; Mboya, EI; **Taxon:** scientificName: *Cenchrus ciliaris* L.; kingdom: Plantae; family: Poaceae; genus: Cenchrus; specificEpithet: ciliaris; scientificNameAuthorship: L.; **Location:** continent: Africa; country: Tanzania; stateProvince: Arusha; county: Ngorongoro; locality: Loliondo; verbatimLocality: Ngorongoro conservation area, past Loliondo township, northward along the road to Tanzania- Kenya border; minimumElevationInMeters: 2080; decimalLatitude: -2.05; decimalLongitude: 35.616667; **Event:** eventDate: 1992-02-18; **Record Level:** institutionCode: NHT; collectionCode: Herbarium; ownerInstitutionCode: NHT; basisOfRecord: PreservedSpecimen

##### Distribution

Widespread

#### Chloris sp.

##### Materials

- **Type status:** Other material. **Occurrence:** catalogNumber: 573; recordNumber: 24262; recordedBy: Peterson, PM; Soreng, RJ; Romaschenko, K; Mbago, F; **Taxon:** scientificName: Chloris; kingdom: Plantae; family: Poaceae; genus: Chloris; **Location:** continent: Africa; country: Tanzania; stateProvince: Shinyanga; locality: Naabi Hill Gate; verbatimLocality: Serengeti National Park, Naabi Hill Gate (at 0.5 km N).; minimumElevationInMeters: 1734; decimalLatitude: -2.83139; decimalLongitude: 34.99672; **Event:** eventDate: 2012-06-16; **Record Level:** institutionCode: US; collectionCode: Herbarium; ownerInstitutionCode: US; basisOfRecord: PreservedSpecimen
- **Type status:** Other material. **Occurrence:** catalogNumber: 574; recordNumber: 24286; recordedBy: Peterson, PM; Soreng, RJ; Romaschenko, K; Mbago, F; **Taxon:** scientificName: Chloris; kingdom: Plantae; family: Poaceae; genus: Chloris; **Location:** continent: Africa; country: Tanzania; stateProvince: Mara; county: Serengeti; locality: Mbuzi Mare camp; verbatimLocality: Serengeti National Park, near Mbuzi Mare camp.; minimumElevationInMeters: 1552; decimalLatitude: -2.23332; decimalLongitude: 34.96467; **Event:** eventDate: 2012-06-17; **Record Level:** institutionCode: US; collectionCode: Herbarium; ownerInstitutionCode: US; basisOfRecord: PreservedSpecimen
- **Type status:** Other material. **Occurrence:** catalogNumber: 575; recordNumber: 24296; recordedBy: Peterson, PM; Soreng, RJ; Romaschenko, K; Mbago, F; **Taxon:** scientificName: Chloris; kingdom: Plantae; family: Poaceae; genus: Chloris; **Location:** continent: Africa; country: Tanzania; stateProvince: Mara; county: Serengeti; locality: Lobo Lodge; verbatimLocality: Serengeti National Park, on top of Lobo Ridge above Lobo Lodge.; minimumElevationInMeters: 1817; decimalLatitude: -1.99967; decimalLongitude: 35.16778; **Event:** eventDate: 2012-06-17; **Record Level:** institutionCode: US; collectionCode: Herbarium; ownerInstitutionCode: US; basisOfRecord: PreservedSpecimen
- **Type status:** Other material. **Occurrence:** catalogNumber: 576; recordNumber: 24305; recordedBy: Peterson, PM; Soreng, RJ; Romaschenko, K; Mbago, F; **Taxon:** scientificName: Chloris; kingdom: Plantae; family: Poaceae; genus: Chloris; **Location:** continent: Africa; country: Tanzania; stateProvince: Arusha; county: Ngorongoro; locality: Ngorongoro Crater; verbatimLocality: Ngorongoro Conservation Area, rim of Ngorongoro Crater (descent gate).; minimumElevationInMeters: 2168; decimalLatitude: -3.15462; decimalLongitude: 35.47717; **Event:** eventDate: 2012-06-19; **Record Level:** institutionCode: US; collectionCode: Herbarium; ownerInstitutionCode: US; basisOfRecord: PreservedSpecimen

#### Chloris gayana

Kunth

##### Materials

- **Type status:** Other material. **Occurrence:** catalogNumber: K000984200; recordNumber: 317; recordedBy: Paulo, S; **Taxon:** scientificName: *Chloris gayana* Kunth; kingdom: Plantae; family: Poaceae; genus: Chloris; specificEpithet: gayana; scientificNameAuthorship: Kunth; **Location:** continent: Africa; country: Tanzania; stateProvince: Arusha; county: Ngorongoro; locality: Ngorongoro Crater; minimumElevationInMeters: 1676; decimalLatitude: -3.166667; decimalLongitude: 35.583333; **Event:** eventDate: 1958-04-19; **Record Level:** institutionCode: K; collectionCode: Herbarium; ownerInstitutionCode: K; basisOfRecord: PreservedSpecimen
- **Type status:** Other material. **Occurrence:** catalogNumber: K000984201; recordNumber: 34; recordedBy: Brooks, GP; **Taxon:** scientificName: *Chloris gayana* Kunth; kingdom: Plantae; family: Poaceae; genus: Chloris; specificEpithet: gayana; scientificNameAuthorship: Kunth; **Location:** continent: Africa; country: Tanzania; stateProvince: Mara; county: Serengeti; locality: Ikoma; minimumElevationInMeters: 1371; decimalLatitude: -2.066667; decimalLongitude: 34.616667; **Event:** eventDate: 1954-02-19; **Record Level:** institutionCode: K; collectionCode: Herbarium; ownerInstitutionCode: K; basisOfRecord: PreservedSpecimen
- **Type status:** Other material. **Occurrence:** catalogNumber: K000984202; recordNumber: 9969; recordedBy: Greenway, PJ; **Taxon:** scientificName: *Chloris gayana* Kunth; kingdom: Plantae; family: Poaceae; genus: Chloris; specificEpithet: gayana; scientificNameAuthorship: Kunth; **Location:** continent: Africa; country: Tanzania; stateProvince: Mara; county: Serengeti; locality: Seronera river; verbatimLocality: the 3rd . crossing from Seronera on the Banagi road.; minimumElevationInMeters: 1432; decimalLatitude: -2.45; decimalLongitude: 34.833333; **Event:** eventDate: 1961-04-03; **Record Level:** institutionCode: K; collectionCode: Herbarium; ownerInstitutionCode: K; basisOfRecord: PreservedSpecimen
- **Type status:** Other material. **Occurrence:** catalogNumber: K000984203; recordNumber: 681; recordedBy: Paulo, S; **Taxon:** scientificName: *Chloris gayana* Kunth; kingdom: Plantae; family: Poaceae; genus: Chloris; specificEpithet: gayana; scientificNameAuthorship: Kunth; **Location:** continent: Africa; country: Tanzania; stateProvince: Arusha; county: Ngorongoro; locality: Olduvai; verbatimLocality: Serengeti plains; decimalLatitude: -2.95; decimalLongitude: 35.166667; **Event:** eventDate: 1956-07-04; **Record Level:** institutionCode: K; collectionCode: Herbarium; ownerInstitutionCode: K; basisOfRecord: PreservedSpecimen
- **Type status:** Other material. **Occurrence:** catalogNumber: K000984204; recordNumber: 5650; recordedBy: Newbould, JB; **Taxon:** scientificName: *Chloris gayana* Kunth; kingdom: Plantae; family: Poaceae; genus: Chloris; specificEpithet: gayana; scientificNameAuthorship: Kunth; **Location:** continent: Africa; country: Tanzania; stateProvince: Arusha; county: Ngorongoro; locality: Ngorongoro; verbatimLocality: Old Boma; minimumElevationInMeters: 2134; decimalLatitude: -3; decimalLongitude: 35.5; **Event:** eventDate: 1971-11; **Record Level:** institutionCode: K; collectionCode: Herbarium; ownerInstitutionCode: K; basisOfRecord: PreservedSpecimen
- **Type status:** Other material. **Occurrence:** catalogNumber: K000984205; recordNumber: 6302; recordedBy: Newbould, JB; **Taxon:** scientificName: *Chloris gayana* Kunth; kingdom: Plantae; family: Poaceae; genus: Chloris; specificEpithet: gayana; scientificNameAuthorship: Kunth; **Location:** continent: Africa; country: Tanzania; stateProvince: Mara; county: Serengeti; locality: Engare Nanyuki; verbatimLocality: Serengeti; minimumElevationInMeters: 1768; decimalLatitude: -2.616667; decimalLongitude: 35.216667; **Event:** eventDate: 1962-11-22; **Record Level:** institutionCode: K; collectionCode: Herbarium; ownerInstitutionCode: K; basisOfRecord: PreservedSpecimen
- **Type status:** Other material. **Occurrence:** catalogNumber: DB1275; recordNumber: 164; recordedBy: Belsky, PJ; **Taxon:** scientificName: *Chloris gayana* Kunth; kingdom: Plantae; family: Poaceae; genus: Chloris; specificEpithet: gayana; scientificNameAuthorship: Kunth; **Location:** continent: Africa; country: Tanzania; stateProvince: Mara; county: Serengeti; locality: Serengeti Research Institute; verbatimLocality: Serengeti Research Institute compound.; decimalLatitude: -2.433333; decimalLongitude: 34.85; **Event:** eventDate: 1981-05-11; **Record Level:** institutionCode: SWRC; collectionCode: Herbarium; ownerInstitutionCode: SWRC; basisOfRecord: PreservedSpecimen
- **Type status:** Other material. **Occurrence:** catalogNumber: DB1277; recordNumber: 4462; recordedBy: Vesey-FitzGerald, LDEF; **Taxon:** scientificName: *Chloris gayana* Kunth; kingdom: Plantae; family: Poaceae; genus: Chloris; specificEpithet: gayana; scientificNameAuthorship: Kunth; **Location:** continent: Africa; country: Tanzania; stateProvince: Mara; county: Serengeti; locality: Lake Lagarja; verbatimLocality: Lake Lagarja.; decimalLatitude: -3; decimalLongitude: 35.033333; **Event:** eventDate: 1964-12-15; **Record Level:** institutionCode: SWRC; collectionCode: Herbarium; ownerInstitutionCode: SWRC; basisOfRecord: PreservedSpecimen
- **Type status:** Other material. **Occurrence:** catalogNumber: DB1278; recordNumber: 130; recordedBy: Vesey-FitzGerald, LDEF; **Taxon:** scientificName: *Chloris gayana* Kunth; kingdom: Plantae; family: Poaceae; genus: Chloris; specificEpithet: gayana; scientificNameAuthorship: Kunth; **Location:** continent: Africa; country: Tanzania; stateProvince: Arusha; county: Ngorongoro; locality: Ngorongoro; verbatimLocality: Ngorongoro; minimumElevationInMeters: 2100; decimalLatitude: -3; decimalLongitude: 35.5; **Event:** eventDate: 1960-02-11; **Record Level:** institutionCode: SWRC; collectionCode: Herbarium; ownerInstitutionCode: SWRC; basisOfRecord: PreservedSpecimen
- **Type status:** Other material. **Occurrence:** catalogNumber: DB1281; recordNumber: 656; recordedBy: Schmidt, W; **Taxon:** scientificName: *Chloris gayana* Kunth; kingdom: Plantae; family: Poaceae; genus: Chloris; specificEpithet: gayana; scientificNameAuthorship: Kunth; **Location:** continent: Africa; country: Tanzania; stateProvince: Mara; county: Serengeti; locality: Bolgonja; verbatimLocality: Bolgonja; decimalLatitude: -1.783333; decimalLongitude: 35.2; **Event:** eventDate: 1972-04-19; **Record Level:** institutionCode: SWRC; collectionCode: Herbarium; ownerInstitutionCode: SWRC; basisOfRecord: PreservedSpecimen
- **Type status:** Other material. **Occurrence:** catalogNumber: DB1282; recordNumber: 575; recordedBy: Schmidt, W; **Taxon:** scientificName: *Chloris gayana* Kunth; kingdom: Plantae; family: Poaceae; genus: Chloris; specificEpithet: gayana; scientificNameAuthorship: Kunth; **Location:** continent: Africa; country: Tanzania; stateProvince: Mara; county: Serengeti; locality: Bolgonja; verbatimLocality: Bolgonja; decimalLatitude: -1.783333; decimalLongitude: 35.2; **Event:** eventDate: 1972-04-18; **Record Level:** institutionCode: SWRC; collectionCode: Herbarium; ownerInstitutionCode: SWRC; basisOfRecord: PreservedSpecimen
- **Type status:** Other material. **Occurrence:** catalogNumber: DB1283; recordNumber: 531; recordedBy: Frame, GW; **Taxon:** scientificName: *Chloris gayana* Kunth; kingdom: Plantae; family: Poaceae; genus: Chloris; specificEpithet: gayana; scientificNameAuthorship: Kunth; **Location:** continent: Africa; country: Tanzania; stateProvince: Arusha; county: Ngorongoro; locality: Empakai Crater; verbatimLocality: Empakaai Crater, outer northen slope.; minimumElevationInMeters: 2000; decimalLatitude: -2.933333; decimalLongitude: 35.816667; **Event:** eventDate: 1974-02-01; **Record Level:** institutionCode: SWRC; collectionCode: Herbarium; ownerInstitutionCode: SWRC; basisOfRecord: PreservedSpecimen
- **Type status:** Other material. **Occurrence:** catalogNumber: 557; recordNumber: 372; recordedBy: Ellemann, L; **Taxon:** scientificName: *Chloris gayana* Kunth; kingdom: Plantae; family: Poaceae; genus: Chloris; specificEpithet: gayana; scientificNameAuthorship: Kunth; **Location:** continent: Africa; country: Tanzania; stateProvince: Arusha; county: Ngorongoro; locality: Endulen; verbatimLocality: Ngorongoro Conservation Area. Endulen Hospital.; minimumElevationInMeters: 1900; decimalLatitude: -3.2; decimalLongitude: 35.266; **Event:** eventDate: 1993-04-20; **Record Level:** institutionCode: AAU; collectionCode: Herbarium; ownerInstitutionCode: AAU; basisOfRecord: PreservedSpecimen
- **Type status:** Other material. **Occurrence:** catalogNumber: 558; recordNumber: 376; recordedBy: Ellemann, L; **Taxon:** scientificName: *Chloris gayana* Kunth; kingdom: Plantae; family: Poaceae; genus: Chloris; specificEpithet: gayana; scientificNameAuthorship: Kunth; **Location:** continent: Africa; country: Tanzania; stateProvince: Arusha; county: Ngorongoro; locality: Endulen; verbatimLocality: Ngorongoro Conservation Area. Endulen Hospital.; minimumElevationInMeters: 1900; decimalLatitude: -3.2; decimalLongitude: 35.266; **Event:** eventDate: 1993-04-20; **Record Level:** institutionCode: AAU; collectionCode: Herbarium; ownerInstitutionCode: AAU; basisOfRecord: PreservedSpecimen
- **Type status:** Other material. **Occurrence:** catalogNumber: 559; recordNumber: 703; recordedBy: Ellemann, L; **Taxon:** scientificName: *Chloris gayana* Kunth; kingdom: Plantae; family: Poaceae; genus: Chloris; specificEpithet: gayana; scientificNameAuthorship: Kunth; **Location:** continent: Africa; country: Tanzania; stateProvince: Arusha; county: Ngorongoro; locality: Endulen; verbatimLocality: Ngorongoro Conservation Area, Ndeyan 4 km North-west of Endulen.; minimumElevationInMeters: 2000; decimalLatitude: -3.2; decimalLongitude: 35.283; **Event:** eventDate: 1993-07-16; **Record Level:** institutionCode: AAU; collectionCode: Herbarium; ownerInstitutionCode: AAU; basisOfRecord: PreservedSpecimen
- **Type status:** Other material. **Occurrence:** catalogNumber: 560; recordNumber: 714; recordedBy: Ellemann, L; **Taxon:** scientificName: *Chloris gayana* Kunth; kingdom: Plantae; family: Poaceae; genus: Chloris; specificEpithet: gayana; scientificNameAuthorship: Kunth; **Location:** continent: Africa; country: Tanzania; stateProvince: Arusha; county: Ngorongoro; locality: Endulen; verbatimLocality: Ngorongoro Conservation Area, ca. 12 km Northwest of Endulen Village; minimumElevationInMeters: 1700; decimalLatitude: -3.166; decimalLongitude: 35.2; **Event:** eventDate: 1993-07-19; **Record Level:** institutionCode: AAU; collectionCode: Herbarium; ownerInstitutionCode: AAU; basisOfRecord: PreservedSpecimen
- **Type status:** Other material. **Occurrence:** catalogNumber: 561; recordNumber: 766; recordedBy: Ellemann, L; **Taxon:** scientificName: *Chloris gayana* Kunth; kingdom: Plantae; family: Poaceae; genus: Chloris; specificEpithet: gayana; scientificNameAuthorship: Kunth; **Location:** continent: Africa; country: Tanzania; stateProvince: Arusha; county: Ngorongoro; locality: Oloronyo forest; verbatimLocality: Ngorongoro Conservation Area, Oloronyo Forest.; minimumElevationInMeters: 2650; decimalLatitude: -3.2; decimalLongitude: 35.35; **Event:** eventDate: 1993-08-05; **Record Level:** institutionCode: AAU; collectionCode: Herbarium; ownerInstitutionCode: AAU; basisOfRecord: PreservedSpecimen
- **Type status:** Other material. **Occurrence:** catalogNumber: 562; recordNumber: 819; recordedBy: Ellemann, L; **Taxon:** scientificName: *Chloris gayana* Kunth; kingdom: Plantae; family: Poaceae; genus: Chloris; specificEpithet: gayana; scientificNameAuthorship: Kunth; **Location:** continent: Africa; country: Tanzania; stateProvince: Arusha; county: Ngorongoro; locality: Nasiporiong; verbatimLocality: Ngorongoro Conservation Area, Nasiporiong'.; minimumElevationInMeters: 1800; decimalLatitude: -3.2; decimalLongitude: 35.2; **Event:** eventDate: 1993-08-19; **Record Level:** institutionCode: AAU; collectionCode: Herbarium; ownerInstitutionCode: AAU; basisOfRecord: PreservedSpecimen
- **Type status:** Other material. **Occurrence:** catalogNumber: 563; recordNumber: 923; recordedBy: Ellemann, L; **Taxon:** scientificName: *Chloris gayana* Kunth; kingdom: Plantae; family: Poaceae; genus: Chloris; specificEpithet: gayana; scientificNameAuthorship: Kunth; **Location:** continent: Africa; country: Tanzania; stateProvince: Arusha; county: Ngorongoro; locality: Masek; verbatimLocality: Ngorongoro Conservation Area, Masek Southwest of Lake Magadi; minimumElevationInMeters: 1600; decimalLatitude: -3.033; decimalLongitude: 35.05; **Event:** eventDate: 1993-09-21; **Record Level:** institutionCode: AAU; collectionCode: Herbarium; ownerInstitutionCode: AAU; basisOfRecord: PreservedSpecimen
- **Type status:** Other material. **Occurrence:** catalogNumber: 564; recordNumber: 942; recordedBy: Ellemann, L; **Taxon:** scientificName: *Chloris gayana* Kunth; kingdom: Plantae; family: Poaceae; genus: Chloris; specificEpithet: gayana; scientificNameAuthorship: Kunth; **Location:** continent: Africa; country: Tanzania; stateProvince: Arusha; county: Ngorongoro; locality: Irmisigiyo; verbatimLocality: Ngorongoro Conservation Area, Irmisigiyo; minimumElevationInMeters: 2350; decimalLatitude: -3.2; decimalLongitude: 35.366; **Event:** eventDate: 1993-09-30; **Record Level:** institutionCode: AAU; collectionCode: Herbarium; ownerInstitutionCode: AAU; basisOfRecord: PreservedSpecimen
- **Type status:** Other material. **Occurrence:** catalogNumber: 565; recordNumber: s.n.; recordedBy: Mboya, E; **Taxon:** scientificName: *Chloris gayana* Kunth; kingdom: Plantae; family: Poaceae; genus: Chloris; specificEpithet: gayana; scientificNameAuthorship: Kunth; **Location:** continent: Africa; country: Tanzania; stateProvince: Mara; county: Serengeti; locality: Seronera Lodge; verbatimLocality: T1. Area around Frankfurt Zoological Society Headquarters and Seronera Lodge.; minimumElevationInMeters: 1512; decimalLatitude: -2.44; decimalLongitude: 34.82; **Event:** eventDate: 2004-02-09; **Record Level:** institutionCode: MO; collectionCode: Herbarium; ownerInstitutionCode: MO; basisOfRecord: PreservedSpecimen
- **Type status:** Other material. **Occurrence:** catalogNumber: 566; recordNumber: s.n.; recordedBy: Mboya, E; **Taxon:** scientificName: *Chloris gayana* Kunth; kingdom: Plantae; family: Poaceae; genus: Chloris; specificEpithet: gayana; scientificNameAuthorship: Kunth; **Location:** continent: Africa; country: Tanzania; stateProvince: Mara; county: Serengeti; locality: Serengeti Central Plains; verbatimLocality: T1. Serengeti National Park. Ndabaka-Seronera Road.; minimumElevationInMeters: 1286; decimalLatitude: -2.27; decimalLongitude: 34.5; **Event:** eventDate: 2004-03-09; **Record Level:** institutionCode: MO; collectionCode: Herbarium; ownerInstitutionCode: MO; basisOfRecord: PreservedSpecimen
- **Type status:** Other material. **Occurrence:** catalogNumber: 1117; recordNumber: 1186; recordedBy: Mollel, NP; Mboya, EI; **Taxon:** scientificName: *Chloris gayana* Kunth; kingdom: Plantae; family: Poaceae; genus: Chloris; specificEpithet: gayana; scientificNameAuthorship: Kunth; **Location:** continent: Africa; country: Tanzania; stateProvince: Arusha; county: Ngorongoro; locality: Laetoli valley; verbatimLocality: Esere-Laetoli road, Laetoli road 3000m from the junction at the footprints site.; minimumElevationInMeters: 1646; decimalLatitude: -3.271389; decimalLongitude: 35.173889; **Event:** eventDate: 2008-04-09; **Record Level:** institutionCode: NHT; collectionCode: Herbarium; ownerInstitutionCode: NHT; basisOfRecord: PreservedSpecimen
- **Type status:** Other material. **Occurrence:** catalogNumber: 1118; recordNumber: 1186; recordedBy: Mollel, NP; Mboya, EI; **Taxon:** scientificName: *Chloris gayana* Kunth; kingdom: Plantae; family: Poaceae; genus: Chloris; specificEpithet: gayana; scientificNameAuthorship: Kunth; **Location:** continent: Africa; country: Tanzania; stateProvince: Arusha; county: Ngorongoro; locality: Laetoli valley; verbatimLocality: Esere-Laetoli road, Laetoli road 3000m from the junction at the footprints site.; minimumElevationInMeters: 1646; decimalLatitude: -3.271389; decimalLongitude: 35.173889; **Event:** eventDate: 2008-04-09; **Record Level:** institutionCode: NCAA; collectionCode: Herbarium; ownerInstitutionCode: NCAA; basisOfRecord: PreservedSpecimen
- **Type status:** Other material. **Occurrence:** catalogNumber: 1119; recordNumber: 935; recordedBy: Mollel, NP; Rusch, GM; Mwakalebe, G; **Taxon:** scientificName: *Chloris gayana* Kunth; kingdom: Plantae; family: Poaceae; genus: Chloris; specificEpithet: gayana; scientificNameAuthorship: Kunth; **Location:** continent: Africa; country: Tanzania; stateProvince: Mara; county: Serengeti; locality: Serengeti NP; verbatimLocality: Along the road to TAWIRI research centre. 36 M, 0360699, 9732458 UTM.; minimumElevationInMeters: 1519; decimalLatitude: -2.25; decimalLongitude: 34.333333; **Event:** eventDate: 2003-02-02; **Record Level:** institutionCode: NHT; collectionCode: Herbarium; ownerInstitutionCode: NHT; basisOfRecord: PreservedSpecimen
- **Type status:** Other material. **Occurrence:** catalogNumber: 1120; recordNumber: 1048; recordedBy: Macha; Leliyo, G; Mboya, EI; **Taxon:** scientificName: *Chloris gayana* Kunth; kingdom: Plantae; family: Poaceae; genus: Chloris; specificEpithet: gayana; scientificNameAuthorship: Kunth; **Location:** continent: Africa; country: Tanzania; stateProvince: Arusha; county: Ngorongoro; locality: Ngorongoro Crater; verbatimLocality: Ngorongoro conservation area; minimumElevationInMeters: 1700; decimalLatitude: -3.166667; decimalLongitude: 35.583333; **Event:** eventDate: 1992-02-16; **Record Level:** institutionCode: NHT; collectionCode: Herbarium; ownerInstitutionCode: NHT; basisOfRecord: PreservedSpecimen

##### Distribution

Widespread, native to Tropical Africa

#### Chloris pycnothrix

Trin.

##### Materials

- **Type status:** Other material. **Occurrence:** catalogNumber: K000984196; recordNumber: 303; recordedBy: Paulo, S; **Taxon:** scientificName: *Chloris pycnothrix* Trin.; kingdom: Plantae; family: Poaceae; genus: Chloris; specificEpithet: pycnothrix; scientificNameAuthorship: Trin.; **Location:** continent: Africa; country: Tanzania; stateProvince: Arusha; county: Ngorongoro; locality: Ngorongoro Crater; minimumElevationInMeters: 1676; decimalLatitude: -3.166667; decimalLongitude: 35.583333; **Event:** eventDate: 1958-04-19; **Record Level:** institutionCode: K; collectionCode: Herbarium; ownerInstitutionCode: K; basisOfRecord: PreservedSpecimen
- **Type status:** Other material. **Occurrence:** catalogNumber: K000984197; recordNumber: 329; recordedBy: Paulo, S; **Taxon:** scientificName: *Chloris pycnothrix* Trin.; kingdom: Plantae; family: Poaceae; genus: Chloris; specificEpithet: pycnothrix; scientificNameAuthorship: Trin.; **Location:** continent: Africa; country: Tanzania; stateProvince: Arusha; county: Ngorongoro; locality: Olbalbal; verbatimLocality: Serengeti; minimumElevationInMeters: 1250; decimalLatitude: -2.95; decimalLongitude: 35.283333; **Event:** eventDate: 1958-04-20; **Record Level:** institutionCode: K; collectionCode: Herbarium; ownerInstitutionCode: K; basisOfRecord: PreservedSpecimen
- **Type status:** Other material. **Occurrence:** catalogNumber: K000984198; recordNumber: 440; recordedBy: Paulo, S; **Taxon:** scientificName: *Chloris pycnothrix* Trin.; kingdom: Plantae; family: Poaceae; genus: Chloris; specificEpithet: pycnothrix; scientificNameAuthorship: Trin.; **Location:** continent: Africa; country: Tanzania; stateProvince: Mara; county: Serengeti; locality: Serengeti; verbatimLocality: Serengeti, Endarbark; decimalLatitude: -2.333333; decimalLongitude: 34.833333; **Event:** eventDate: 1958-05-09; **Record Level:** institutionCode: K; collectionCode: Herbarium; ownerInstitutionCode: K; basisOfRecord: PreservedSpecimen
- **Type status:** Other material. **Occurrence:** catalogNumber: K000984199; recordNumber: 9874; recordedBy: Greenway, PJ; **Taxon:** scientificName: *Chloris pycnothrix* Trin.; kingdom: Plantae; family: Poaceae; genus: Chloris; specificEpithet: pycnothrix; scientificNameAuthorship: Trin.; **Location:** continent: Africa; country: Tanzania; stateProvince: Mara; county: Serengeti; locality: Seronera; verbatimLocality: Serengeti; minimumElevationInMeters: 1554; decimalLatitude: -2.45; decimalLongitude: 34.833333; **Event:** eventDate: 1961-03-21; **Record Level:** institutionCode: K; collectionCode: Herbarium; ownerInstitutionCode: K; basisOfRecord: PreservedSpecimen
- **Type status:** Other material. **Occurrence:** catalogNumber: DB1287; recordNumber: 50; recordedBy: Belsky, PJ; **Taxon:** scientificName: *Chloris pycnothrix* Trin.; kingdom: Plantae; family: Poaceae; genus: Chloris; specificEpithet: pycnothrix; scientificNameAuthorship: Trin.; **Location:** continent: Africa; country: Tanzania; stateProvince: Mara; county: Serengeti; locality: Ndutu Lodge; verbatimLocality: 5 km west of Ndutu Lodge; decimalLatitude: -3.02; decimalLongitude: 34.996944; **Event:** eventDate: 1981-04-10; **Record Level:** institutionCode: SWRC; collectionCode: Herbarium; ownerInstitutionCode: SWRC; basisOfRecord: PreservedSpecimen
- **Type status:** Other material. **Occurrence:** catalogNumber: DB1289; recordNumber: 192; recordedBy: Belsky, PJ; **Taxon:** scientificName: *Chloris pycnothrix* Trin.; kingdom: Plantae; family: Poaceae; genus: Chloris; specificEpithet: pycnothrix; scientificNameAuthorship: Trin.; **Location:** continent: Africa; country: Tanzania; stateProvince: Mara; county: Serengeti; locality: Serengeti Research Institute; verbatimLocality: SRI Tennis Court.; decimalLatitude: -2.433333; decimalLongitude: 34.85; **Event:** eventDate: 1981-05-11; **Record Level:** institutionCode: SWRC; collectionCode: Herbarium; ownerInstitutionCode: SWRC; basisOfRecord: PreservedSpecimen
- **Type status:** Other material. **Occurrence:** catalogNumber: 567; recordNumber: 366; recordedBy: Ellemann, L; **Taxon:** scientificName: *Chloris pycnothrix* Trin.; kingdom: Plantae; family: Poaceae; genus: Chloris; specificEpithet: pycnothrix; scientificNameAuthorship: Trin.; **Location:** continent: Africa; country: Tanzania; stateProvince: Arusha; county: Ngorongoro; locality: Endulen; verbatimLocality: Ngorongoro Conservation Area. Endulen Hospital.; minimumElevationInMeters: 1900; decimalLatitude: -3.2; decimalLongitude: 35.266; **Event:** eventDate: 1993-04-20; **Record Level:** institutionCode: AAU; collectionCode: Herbarium; ownerInstitutionCode: AAU; basisOfRecord: PreservedSpecimen
- **Type status:** Other material. **Occurrence:** catalogNumber: 568; recordNumber: 706; recordedBy: Ellemann, L; **Taxon:** scientificName: *Chloris pycnothrix* Trin.; kingdom: Plantae; family: Poaceae; genus: Chloris; specificEpithet: pycnothrix; scientificNameAuthorship: Trin.; **Location:** continent: Africa; country: Tanzania; stateProvince: Arusha; county: Ngorongoro; locality: Endulen; verbatimLocality: Ngorongoro Conservation Area, Endulen Hospital,; minimumElevationInMeters: 1900; decimalLatitude: -3.2; decimalLongitude: 35.266; **Event:** eventDate: 1993-07-19; **Record Level:** institutionCode: AAU; collectionCode: Herbarium; ownerInstitutionCode: AAU; basisOfRecord: PreservedSpecimen
- **Type status:** Other material. **Occurrence:** catalogNumber: 569; recordNumber: 814; recordedBy: Ellemann, L; **Taxon:** scientificName: *Chloris pycnothrix* Trin.; kingdom: Plantae; family: Poaceae; genus: Chloris; specificEpithet: pycnothrix; scientificNameAuthorship: Trin.; **Location:** continent: Africa; country: Tanzania; stateProvince: Arusha; county: Ngorongoro; locality: Nasiporiong; verbatimLocality: Ngorongoro Conservation Area, Nasiporiong'.; minimumElevationInMeters: 1800; decimalLatitude: -3.2; decimalLongitude: 35.2; **Event:** eventDate: 1993-08-19; **Record Level:** institutionCode: AAU; collectionCode: Herbarium; ownerInstitutionCode: AAU; basisOfRecord: PreservedSpecimen
- **Type status:** Other material. **Occurrence:** catalogNumber: 570; recordNumber: 882; recordedBy: Ellemann, L; **Taxon:** scientificName: *Chloris pycnothrix* Trin.; kingdom: Plantae; family: Poaceae; genus: Chloris; specificEpithet: pycnothrix; scientificNameAuthorship: Trin.; **Location:** continent: Africa; country: Tanzania; stateProvince: Arusha; county: Ngorongoro; locality: Olbalbal; verbatimLocality: Ngorongoro Conservation Area, Olbalbal.; minimumElevationInMeters: 1300; decimalLatitude: -3.05; decimalLongitude: 35.4; **Event:** eventDate: 1993-09-10; **Record Level:** institutionCode: AAU; collectionCode: Herbarium; ownerInstitutionCode: AAU; basisOfRecord: PreservedSpecimen
- **Type status:** Other material. **Occurrence:** catalogNumber: DB1284; recordNumber: 9874; recordedBy: Greenway, PJ; **Taxon:** scientificName: *Chloris pycnothrix* Trin.; kingdom: Plantae; family: Poaceae; genus: Chloris; specificEpithet: pycnothrix; scientificNameAuthorship: Trin.; **Location:** continent: Africa; country: Tanzania; stateProvince: Mara; county: Serengeti; locality: Seronera; verbatimLocality: Serengeti; minimumElevationInMeters: 1554; decimalLatitude: -2.45; decimalLongitude: 34.833333; **Event:** eventDate: 1961-03-21; **Record Level:** institutionCode: SWRC; collectionCode: Herbarium; ownerInstitutionCode: SWRC; basisOfRecord: PreservedSpecimen
- **Type status:** Other material. **Occurrence:** catalogNumber: DB1288; recordNumber: 9874; recordedBy: Greenway, PJ; **Taxon:** scientificName: *Chloris pycnothrix* Trin.; kingdom: Plantae; family: Poaceae; genus: Chloris; specificEpithet: pycnothrix; scientificNameAuthorship: Trin.; **Location:** continent: Africa; country: Tanzania; stateProvince: Mara; county: Serengeti; locality: Seronera; verbatimLocality: Serengeti; minimumElevationInMeters: 1554; decimalLatitude: -2.45; decimalLongitude: 34.833333; **Event:** eventDate: 1961-03-21; **Record Level:** institutionCode: SWRC; collectionCode: Herbarium; ownerInstitutionCode: SWRC; basisOfRecord: PreservedSpecimen

##### Distribution

Widespread

#### Chloris virgata

Sw.

##### Materials

- **Type status:** Other material. **Occurrence:** catalogNumber: K000984206; recordNumber: 9863; recordedBy: Greenway, PJ; **Taxon:** scientificName: *Chloris virgata* Sw.; kingdom: Plantae; family: Poaceae; genus: Chloris; specificEpithet: virgata; scientificNameAuthorship: Sw.; **Location:** continent: Africa; country: Tanzania; stateProvince: Mara; county: Serengeti; locality: Seronera; verbatimLocality: Serengeti; minimumElevationInMeters: 1554; decimalLatitude: -2.45; decimalLongitude: 34.833333; **Event:** eventDate: 1961-03-20; **Record Level:** institutionCode: K; collectionCode: Herbarium; ownerInstitutionCode: K; basisOfRecord: PreservedSpecimen
- **Type status:** Other material. **Occurrence:** catalogNumber: DB1296; recordNumber: 204; recordedBy: Belsky, PJ; **Taxon:** scientificName: *Chloris virgata* Sw.; kingdom: Plantae; family: Poaceae; genus: Chloris; specificEpithet: virgata; scientificNameAuthorship: Sw.; **Location:** continent: Africa; country: Tanzania; stateProvince: Mara; county: Serengeti; locality: Serengeti Research Institute; verbatimLocality: SRI tennis court; decimalLatitude: -2.433333; decimalLongitude: 34.85; **Event:** eventDate: 1981-05-11; **Record Level:** institutionCode: SWRC; collectionCode: Herbarium; ownerInstitutionCode: SWRC; basisOfRecord: PreservedSpecimen
- **Type status:** Other material. **Occurrence:** catalogNumber: 572; recordNumber: s.n.; recordedBy: Mboya, E; **Taxon:** scientificName: *Chloris virgata* Sw.; kingdom: Plantae; family: Poaceae; genus: Chloris; specificEpithet: virgata; scientificNameAuthorship: Sw.; **Location:** continent: Africa; country: Tanzania; stateProvince: Mara; county: Serengeti; locality: Serengeti National Park; verbatimLocality: T1. Serengeti National Park. Ndabaka-Seronera Road.; minimumElevationInMeters: 1297; decimalLatitude: -2.28; decimalLongitude: 34.5; **Event:** eventDate: 2004-02-07; **Record Level:** institutionCode: MO; collectionCode: Herbarium; ownerInstitutionCode: MO; basisOfRecord: PreservedSpecimen
- **Type status:** Other material. **Occurrence:** catalogNumber: DB1297; recordNumber: 9863; recordedBy: Greenway, PJ; **Taxon:** scientificName: *Chloris virgata* Sw.; kingdom: Plantae; family: Poaceae; genus: Chloris; specificEpithet: virgata; scientificNameAuthorship: Sw.; **Location:** continent: Africa; country: Tanzania; stateProvince: Mara; county: Serengeti; locality: Seronera; verbatimLocality: Serengeti; minimumElevationInMeters: 1554; decimalLatitude: -2.45; decimalLongitude: 34.833333; **Event:** eventDate: 1961-03-20; **Record Level:** institutionCode: SWRC; collectionCode: Herbarium; ownerInstitutionCode: SWRC; basisOfRecord: PreservedSpecimen

##### Distribution

Widespread

#### Chrysochloa orientalis

(C.E.Hubb) Swallen

##### Materials

- **Type status:** Other material. **Occurrence:** catalogNumber: DB1300; recordNumber: 2004; recordedBy: Suleiman, HO; Lyaruu, HVM; Lyamuya, N; **Taxon:** scientificName: *Chrysochloa orientalis* (C.E.Hubb) Swallen; kingdom: Plantae; family: Poaceae; genus: Chrysochloa; specificEpithet: orientalis; scientificNameAuthorship: (C.E.Hubb) Swallen; **Location:** continent: Africa; country: Tanzania; stateProvince: Mara; county: Serengeti; locality: Serengeti National Park; verbatimLocality: T1 Serengeti National Park Mihale game Transect (MGT 202); decimalLatitude: -2.333333; decimalLongitude: 34.833333; **Event:** eventDate: 2001-02-14; **Record Level:** institutionCode: SWRC; collectionCode: Herbarium; ownerInstitutionCode: SWRC; basisOfRecord: PreservedSpecimen
- **Type status:** Other material. **Occurrence:** catalogNumber: 380; recordNumber: 2004; recordedBy: Suleiman, HO; Lyaruu, HVM; Lyamuya, N; **Taxon:** scientificName: *Chrysochloa orientalis* (C.E.Hubb) Swallen; kingdom: Plantae; family: Poaceae; genus: Chrysochloa; specificEpithet: orientalis; scientificNameAuthorship: (C.E.Hubb) Swallen; **Location:** continent: Africa; country: Tanzania; stateProvince: Mara; county: Serengeti; locality: Serengeti National Park; verbatimLocality: T1 Serengeti National Park Mihale game Transect (MGT 202); decimalLatitude: -2.333333; decimalLongitude: 34.833333; **Event:** eventDate: 2001-02-14; **Record Level:** institutionCode: DSM; collectionCode: Herbarium; ownerInstitutionCode: DSM; basisOfRecord: PreservedSpecimen
- **Type status:** Other material. **Occurrence:** catalogNumber: DB1301; recordNumber: 2004; recordedBy: Suleiman, HO; Lyaruu, HVM; Lyamuya, N; **Taxon:** scientificName: *Chrysochloa orientalis* (C.E.Hubb) Swallen; kingdom: Plantae; family: Poaceae; genus: Chrysochloa; specificEpithet: orientalis; scientificNameAuthorship: (C.E.Hubb) Swallen; **Location:** continent: Africa; country: Tanzania; stateProvince: Mara; county: Serengeti; locality: Serengeti National Park; verbatimLocality: T1 Serengeti National Park Mihale game Transect (MGT 202); decimalLatitude: -2.333333; decimalLongitude: 34.833333; **Event:** eventDate: 2001-02-14; **Record Level:** institutionCode: SWRC; collectionCode: Herbarium; ownerInstitutionCode: SWRC; basisOfRecord: PreservedSpecimen
- **Type status:** Other material. **Occurrence:** catalogNumber: DB1302; recordNumber: 219; recordedBy: Belsky, PJ; **Taxon:** scientificName: *Chrysochloa orientalis* (C.E.Hubb) Swallen; kingdom: Plantae; family: Poaceae; genus: Chrysochloa; specificEpithet: orientalis; scientificNameAuthorship: (C.E.Hubb) Swallen; **Location:** continent: Africa; country: Tanzania; stateProvince: Mara; county: Serengeti; locality: Serengeti Research Institute; verbatimLocality: SRI Airfield; decimalLatitude: -2.45; decimalLongitude: 34.833333; **Event:** eventDate: 1981-05-15; **Record Level:** institutionCode: SWRC; collectionCode: Herbarium; ownerInstitutionCode: SWRC; basisOfRecord: PreservedSpecimen
- **Type status:** Other material. **Occurrence:** catalogNumber: DB1303; recordNumber: 4655; recordedBy: Vesey-FitzGerald, LDEF; **Taxon:** scientificName: *Chrysochloa orientalis* (C.E.Hubb) Swallen; kingdom: Plantae; family: Poaceae; genus: Chrysochloa; specificEpithet: orientalis; scientificNameAuthorship: (C.E.Hubb) Swallen; **Location:** continent: Africa; country: Tanzania; stateProvince: Mara; county: Serengeti; locality: Grumeti Plains; verbatimLocality: Grumetii plains.; minimumElevationInMeters: 1200; decimalLatitude: -2.333333; decimalLongitude: 34.833333; **Event:** eventDate: 1965-04-26; **Record Level:** institutionCode: SWRC; collectionCode: Herbarium; ownerInstitutionCode: SWRC; basisOfRecord: PreservedSpecimen
- **Type status:** Other material. **Occurrence:** catalogNumber: DB1304; recordNumber: 216; recordedBy: Schmidt, W; **Taxon:** scientificName: *Chrysochloa orientalis* (C.E.Hubb) Swallen; kingdom: Plantae; family: Poaceae; genus: Chrysochloa; specificEpithet: orientalis; scientificNameAuthorship: (C.E.Hubb) Swallen; **Location:** continent: Africa; country: Tanzania; stateProvince: Mara; county: Serengeti; locality: Serengeti Plains; verbatimLocality: South of Seronera.; decimalLatitude: -2.333333; decimalLongitude: 34.833333; **Event:** eventDate: 1972-03-21; **Record Level:** institutionCode: SWRC; collectionCode: Herbarium; ownerInstitutionCode: SWRC; basisOfRecord: PreservedSpecimen
- **Type status:** Other material. **Occurrence:** catalogNumber: DB1306; recordNumber: 216; recordedBy: Schmidt, W; **Taxon:** scientificName: *Chrysochloa orientalis* (C.E.Hubb) Swallen; kingdom: Plantae; family: Poaceae; genus: Chrysochloa; specificEpithet: orientalis; scientificNameAuthorship: (C.E.Hubb) Swallen; **Location:** continent: Africa; country: Tanzania; stateProvince: Mara; county: Serengeti; locality: Serengeti Plains; verbatimLocality: South of Seronera.; decimalLatitude: -2.333333; decimalLongitude: 34.833333; **Event:** eventDate: 1972-03-21; **Record Level:** institutionCode: SWRC; collectionCode: Herbarium; ownerInstitutionCode: SWRC; basisOfRecord: PreservedSpecimen
- **Type status:** Other material. **Occurrence:** catalogNumber: K001087090; recordNumber: 9873; recordedBy: Greenway, PJ; **Taxon:** scientificName: *Chrysochloa orientalis* (C.E.Hubb) Swallen; kingdom: Plantae; family: Poaceae; genus: Chrysochloa; specificEpithet: orientalis; scientificNameAuthorship: (C.E.Hubb) Swallen; **Location:** continent: Africa; country: Tanzania; stateProvince: Mara; county: Serengeti; locality: Seronera; verbatimLocality: Serengeti; minimumElevationInMeters: 1554; decimalLatitude: -2.45; decimalLongitude: 34.833333; **Event:** eventDate: 1961-03-21; **Record Level:** institutionCode: K; collectionCode: Herbarium; ownerInstitutionCode: K; basisOfRecord: PreservedSpecimen
- **Type status:** Other material. **Occurrence:** catalogNumber: K001087091; recordNumber: 438; recordedBy: Paulo, S; **Taxon:** scientificName: *Chrysochloa orientalis* (C.E.Hubb) Swallen; kingdom: Plantae; family: Poaceae; genus: Chrysochloa; specificEpithet: orientalis; scientificNameAuthorship: (C.E.Hubb) Swallen; **Location:** continent: Africa; country: Tanzania; stateProvince: Mara; county: Serengeti; locality: Endarbark; verbatimLocality: Serengeti area.; **Event:** eventDate: 1958-05-09; **Record Level:** institutionCode: K; collectionCode: Herbarium; ownerInstitutionCode: K; basisOfRecord: PreservedSpecimen

##### Distribution

Tanzania, Kenya, Uganda & Democratic Republic of Congo

#### Chrysopogon plumulosus

Hochst.

Chrysopogon aucheri (Boiss.) Stapf var. *quinqueplumis* (A.Rich) Stapf

##### Materials

- **Type status:** Other material. **Occurrence:** catalogNumber: K000984066; recordNumber: EAH12695; recordedBy: Tanner, M; **Taxon:** scientificName: *Chrysopogon plumulosus* Hochst.; kingdom: Plantae; family: Poaceae; genus: Chrysopogon; specificEpithet: plumulosus; scientificNameAuthorship: Hochst.; **Location:** continent: Africa; country: Tanzania; stateProvince: Mara; county: Serengeti; locality: Lake Lagarja; verbatimLocality: Serengeti; decimalLatitude: -3; decimalLongitude: 35.033333; **Event:** eventDate: 1968-3; **Record Level:** institutionCode: K; collectionCode: Herbarium; ownerInstitutionCode: K; basisOfRecord: PreservedSpecimen
- **Type status:** Other material. **Occurrence:** catalogNumber: K001087095; recordNumber: 251; recordedBy: Oteke, J; **Taxon:** scientificName: *Chrysopogon plumulosus* Hochst.; kingdom: Plantae; family: Poaceae; genus: Chrysopogon; specificEpithet: plumulosus; scientificNameAuthorship: Hochst.; **Location:** continent: Africa; country: Tanzania; stateProvince: Arusha; county: Ngorongoro; locality: Lemuta; verbatimLocality: East Serengeti; minimumElevationInMeters: 1722; decimalLatitude: -2.7; decimalLongitude: 35.266667; **Event:** eventDate: 1962-11-26; **Record Level:** institutionCode: K; collectionCode: Herbarium; ownerInstitutionCode: K; basisOfRecord: PreservedSpecimen
- **Type status:** Other material. **Occurrence:** catalogNumber: K001087096; recordNumber: 6396; recordedBy: Newbould, JB; **Taxon:** scientificName: *Chrysopogon plumulosus* Hochst.; kingdom: Plantae; family: Poaceae; genus: Chrysopogon; specificEpithet: plumulosus; scientificNameAuthorship: Hochst.; **Location:** continent: Africa; country: Tanzania; stateProvince: Arusha; county: Ngorongoro; locality: Olkarien; verbatimLocality: Olgarien; minimumElevationInMeters: 1371; decimalLatitude: -2.6; decimalLongitude: 35.366667; **Event:** eventDate: 1962-12-20; **Record Level:** institutionCode: K; collectionCode: Herbarium; ownerInstitutionCode: K; basisOfRecord: PreservedSpecimen

##### Distribution

Tropical Africa & Arabia

#### Ctenium somalense

(Chiov.) Chiov.

Ctenium concinnum Nees var. minus Pilg.

##### Materials

- **Type status:** Other material. **Occurrence:** catalogNumber: K001087094; recordNumber: 10767; recordedBy: Greenway, PJ; **Taxon:** scientificName: Ctenium concinnum Nees var. minus Pilg.; kingdom: Plantae; family: Poaceae; genus: Ctenium; specificEpithet: concinnum; infraspecificEpithet: minus; scientificNameAuthorship: Pilg.; **Location:** continent: Africa; country: Tanzania; stateProvince: Mara; county: Serengeti; locality: Mara River Guard Post; minimumElevationInMeters: 1280; decimalLatitude: -1.583333; decimalLongitude: 34.833333; **Event:** eventDate: 1962-08-21; **Record Level:** institutionCode: K; collectionCode: Herbarium; ownerInstitutionCode: K; basisOfRecord: PreservedSpecimen

##### Distribution

Tropical Africa

#### Cymbopogon sp.

##### Materials

- **Type status:** Other material. **Occurrence:** catalogNumber: 1148; recordNumber: 917; recordedBy: Mollel, NP; Rusch, GM; Mwakalebe, G; **Taxon:** scientificName: Cymbopogon; kingdom: Plantae; family: Poaceae; genus: Cymbopogon; **Location:** continent: Africa; country: Tanzania; stateProvince: Mara; county: Serengeti; locality: Serengeti NP; verbatimLocality: Serengeti National Park, along the road to Nabaka gate. 36M, 0668594, 9748618 UTM.; minimumElevationInMeters: 1305; decimalLatitude: -2.25; decimalLongitude: 34.333333; **Event:** eventDate: 2003-02-01; **Record Level:** institutionCode: NHT; collectionCode: Herbarium; ownerInstitutionCode: NHT; basisOfRecord: PreservedSpecimen

#### Cymbopogon caesius

(Hook. & Arn.) Stapf

Cymbopogon excavatus (Hochst.) Stapf ex Burtt Davy

##### Materials

- **Type status:** Other material. **Occurrence:** catalogNumber: K000984046; recordNumber: 10046; recordedBy: Greenway, PJ; Tanner, M; **Taxon:** scientificName: *Cymbopogon caesius* (Hook. & Arn.) Stapf; kingdom: Plantae; family: Poaceae; genus: Cymbopogon; specificEpithet: caesius; scientificNameAuthorship: (Hook. & Arn.) Stapf; **Location:** continent: Africa; country: Tanzania; stateProvince: Shinyanga; locality: Lake Magadi; verbatimLocality: N.W. and W. of Lake Magadi; minimumElevationInMeters: 1432; decimalLatitude: -2.633333; decimalLongitude: 34.9; **Event:** eventDate: 1961-04-13; **Record Level:** institutionCode: K; collectionCode: Herbarium; ownerInstitutionCode: K; basisOfRecord: PreservedSpecimen
- **Type status:** Other material. **Occurrence:** catalogNumber: K000984047; recordNumber: 12607; recordedBy: Greenway, PJ; Tanner, M; **Taxon:** scientificName: *Cymbopogon caesius* (Hook. & Arn.) Stapf; kingdom: Plantae; family: Poaceae; genus: Cymbopogon; specificEpithet: caesius; scientificNameAuthorship: (Hook. & Arn.) Stapf; **Location:** continent: Africa; country: Tanzania; stateProvince: Arusha; county: Ngorongoro; locality: Engitati Hill; verbatimLocality: W. side of Ngorongoro Crater floor; minimumElevationInMeters: 1707; decimalLatitude: -3.133333; decimalLongitude: 35.55; **Event:** eventDate: 1966-07-24; **Record Level:** institutionCode: K; collectionCode: Herbarium; ownerInstitutionCode: K; basisOfRecord: PreservedSpecimen
- **Type status:** Other material. **Occurrence:** catalogNumber: K000984049; recordNumber: 10186; recordedBy: Greenway, PJ; **Taxon:** scientificName: *Cymbopogon caesius* (Hook. & Arn.) Stapf; kingdom: Plantae; family: Poaceae; genus: Cymbopogon; specificEpithet: caesius; scientificNameAuthorship: (Hook. & Arn.) Stapf; **Location:** continent: Africa; country: Tanzania; stateProvince: Mara; county: Serengeti; locality: Seronera; verbatimLocality: W. of Seronera, Serengeti; minimumElevationInMeters: 1463; decimalLatitude: -2.45; decimalLongitude: 34.833333; **Event:** eventDate: 1961-05-15; **Record Level:** institutionCode: K; collectionCode: Herbarium; ownerInstitutionCode: K; basisOfRecord: PreservedSpecimen
- **Type status:** Other material. **Occurrence:** catalogNumber: K000984048; recordNumber: 10129; recordedBy: Greenway, PJ; **Taxon:** scientificName: *Cymbopogon excavatus* (Hochst.) Stapf ex Burtt Davy; kingdom: Plantae; family: Poaceae; genus: Cymbopogon; specificEpithet: excavatus; scientificNameAuthorship: (Hochst.) Stapf ex Burtt Davy; **Location:** continent: Africa; country: Tanzania; stateProvince: Mara; county: Serengeti; locality: Tabora; verbatimLocality: mile 46.6 from Bologonja river via Kleins Camp; minimumElevationInMeters: 1615; decimalLatitude: -1.816667; decimalLongitude: 34.633333; **Event:** eventDate: 1961-04-30; **Record Level:** institutionCode: K; collectionCode: Herbarium; ownerInstitutionCode: K; basisOfRecord: PreservedSpecimen
- **Type status:** Other material. **Occurrence:** catalogNumber: K000984050; recordNumber: 39; recordedBy: Brooks, GP; **Taxon:** scientificName: *Cymbopogon excavatus* (Hochst.) Stapf ex Burtt Davy; kingdom: Plantae; family: Poaceae; genus: Cymbopogon; specificEpithet: excavatus; scientificNameAuthorship: (Hochst.) Stapf ex Burtt Davy; **Location:** continent: Africa; country: Tanzania; stateProvince: Mara; county: Serengeti; locality: Ikoma; minimumElevationInMeters: 1371; decimalLatitude: -2.066667; decimalLongitude: 34.616667; **Event:** eventDate: 1954-02-19; **Record Level:** institutionCode: K; collectionCode: Herbarium; ownerInstitutionCode: K; basisOfRecord: PreservedSpecimen

##### Distribution

Tropical Africa & Asia

#### Cymbopogon pospischilii

(K.Schum.) C.E.Hubb

##### Materials

- **Type status:** Other material. **Occurrence:** catalogNumber: K000984051; recordNumber: 10380; recordedBy: Greenway, PJ; Tanner, M; **Taxon:** scientificName: *Cymbopogon pospischilii* (K.Schum.) C.E.Hubb; kingdom: Plantae; family: Poaceae; genus: Cymbopogon; specificEpithet: pospischilii; scientificNameAuthorship: (K.Schum.) C.E.Hubb; **Location:** continent: Africa; country: Tanzania; stateProvince: Shinyanga; locality: Simiyu river; verbatimLocality: S. boundary, Mile 10.2 west; minimumElevationInMeters: 1463; decimalLatitude: -2.916667; decimalLongitude: 34.8; **Event:** eventDate: 1961-06-18; **Record Level:** institutionCode: K; collectionCode: Herbarium; ownerInstitutionCode: K; basisOfRecord: PreservedSpecimen
- **Type status:** Other material. **Occurrence:** catalogNumber: K000984052; recordNumber: 2; recordedBy: Herlocker, D; Hoeck, H; Kreulen, AR; **Taxon:** scientificName: *Cymbopogon pospischilii* (K.Schum.) C.E.Hubb; kingdom: Plantae; family: Poaceae; genus: Cymbopogon; specificEpithet: pospischilii; scientificNameAuthorship: (K.Schum.) C.E.Hubb; **Location:** continent: Africa; country: Tanzania; stateProvince: Mara; county: Serengeti; locality: Serengeti National Park; verbatimLocality: E. plains; decimalLatitude: -2.333333; decimalLongitude: 34.833333; **Event:** eventDate: 1973-02-07; **Record Level:** institutionCode: K; collectionCode: Herbarium; ownerInstitutionCode: K; basisOfRecord: PreservedSpecimen

##### Distribution

Tropical Africa & Asia

#### Cynodon sp.

##### Materials

- **Type status:** Other material. **Occurrence:** catalogNumber: 581; recordNumber: 452; recordedBy: Ellemann, L; **Taxon:** scientificName: Cynodon; kingdom: Plantae; family: Poaceae; genus: Cynodon; **Location:** continent: Africa; country: Tanzania; stateProvince: Arusha; county: Ngorongoro; locality: Endulen; verbatimLocality: Ngorongoro Conservation Area, ca. 12 km Northwest of Endulen Village; minimumElevationInMeters: 1700; decimalLatitude: -3.116; decimalLongitude: 35.2; **Event:** eventDate: 1993-04-27; **Record Level:** institutionCode: AAU; collectionCode: Herbarium; ownerInstitutionCode: AAU; basisOfRecord: PreservedSpecimen
- **Type status:** Other material. **Occurrence:** catalogNumber: 582; recordNumber: 24259; recordedBy: Peterson, PM; Soreng, RJ; Romaschenko, K; Mbago, F; **Taxon:** scientificName: Cynodon; kingdom: Plantae; family: Poaceae; genus: Cynodon; **Location:** continent: Africa; country: Tanzania; stateProvince: Shinyanga; locality: Naabi Hill Gate; verbatimLocality: Serengeti National Park, Naabi Hill Gate (at 0.5 km N).; minimumElevationInMeters: 1734; decimalLatitude: -2.83139; decimalLongitude: 34.99672; **Event:** eventDate: 2012-06-16; **Record Level:** institutionCode: US; collectionCode: Herbarium; ownerInstitutionCode: US; basisOfRecord: PreservedSpecimen
- **Type status:** Other material. **Occurrence:** catalogNumber: 583; recordNumber: 24268; recordedBy: Peterson, PM; Soreng, RJ; Romaschenko, K; Mbago, F; **Taxon:** scientificName: Cynodon; kingdom: Plantae; family: Poaceae; genus: Cynodon; **Location:** continent: Africa; country: Tanzania; stateProvince: Shinyanga; locality: Naabi Hill Gate; verbatimLocality: Serengeti National Park, at 12 km NW of Naabi Hill Gate.; minimumElevationInMeters: 1651; decimalLatitude: -2.73597; decimalLongitude: 34.95284; **Event:** eventDate: 2012-06-16; **Record Level:** institutionCode: US; collectionCode: Herbarium; ownerInstitutionCode: US; basisOfRecord: PreservedSpecimen
- **Type status:** Other material. **Occurrence:** catalogNumber: 584; recordNumber: 24270; recordedBy: Peterson, PM; Soreng, RJ; Romaschenko, K; Mbago, F; **Taxon:** scientificName: Cynodon; kingdom: Plantae; family: Poaceae; genus: Cynodon; **Location:** continent: Africa; country: Tanzania; stateProvince: Shinyanga; locality: Naabi Hill Gate; verbatimLocality: Serengeti National Park, at 12 km NW of Naabi Hill Gate.; minimumElevationInMeters: 1651; decimalLatitude: -2.73597; decimalLongitude: 34.95284; **Event:** eventDate: 2012-06-16; **Record Level:** institutionCode: US; collectionCode: Herbarium; ownerInstitutionCode: US; basisOfRecord: PreservedSpecimen
- **Type status:** Other material. **Occurrence:** catalogNumber: 585; recordNumber: 24318; recordedBy: Peterson, PM; Soreng, RJ; Romaschenko, K; Mbago, F; **Taxon:** scientificName: Cynodon; kingdom: Plantae; family: Poaceae; genus: Cynodon; **Location:** continent: Africa; country: Tanzania; stateProvince: Arusha; county: Ngorongoro; locality: Lake Magadi; verbatimLocality: Ngorongoro Conservation Area, W side of Lake Magadi.; minimumElevationInMeters: 1739; decimalLatitude: -3.17713; decimalLongitude: 35.51548; **Event:** eventDate: 2012-06-19; **Record Level:** institutionCode: US; collectionCode: Herbarium; ownerInstitutionCode: US; basisOfRecord: PreservedSpecimen
- **Type status:** Other material. **Occurrence:** catalogNumber: 1114; recordNumber: 5656; recordedBy: Chuwa, S; Amiyo, T; **Taxon:** scientificName: Cynodon; kingdom: Plantae; family: Poaceae; genus: Cynodon; **Location:** continent: Africa; country: Tanzania; stateProvince: Arusha; county: Ngorongoro; locality: Ngorongoro Crater; verbatimLocality: Gorgor swamp; minimumElevationInMeters: 1707; decimalLatitude: -3.166667; decimalLongitude: 35.583333; **Event:** eventDate: 1994-11-20; **Record Level:** institutionCode: NHT; collectionCode: Herbarium; ownerInstitutionCode: NHT; basisOfRecord: PreservedSpecimen

#### Cynodon aethiopicus

Clayton & Harlan

##### Materials

- **Type status:** Other material. **Occurrence:** catalogNumber: 578; recordNumber: 446; recordedBy: Ellemann, L; **Taxon:** scientificName: *Cynodon aethiopicus* Clayton & Harlan; kingdom: Plantae; family: Poaceae; genus: Cynodon; specificEpithet: aethiopicus; scientificNameAuthorship: Clayton & Harlan; **Location:** continent: Africa; country: Tanzania; stateProvince: Arusha; county: Ngorongoro; locality: Endulen; verbatimLocality: Ngorongoro Conservation Area, 15 km Northwest of Endulen village.; minimumElevationInMeters: 1700; decimalLatitude: -3.116; decimalLongitude: 35.2; **Event:** eventDate: 1993-04-27; **Record Level:** institutionCode: AAU; collectionCode: Herbarium; ownerInstitutionCode: AAU; basisOfRecord: PreservedSpecimen
- **Type status:** Other material. **Occurrence:** catalogNumber: 579; recordNumber: 708; recordedBy: Ellemann, L; **Taxon:** scientificName: *Cynodon aethiopicus* Clayton & Harlan; kingdom: Plantae; family: Poaceae; genus: Cynodon; specificEpithet: aethiopicus; scientificNameAuthorship: Clayton & Harlan; **Location:** continent: Africa; country: Tanzania; stateProvince: Arusha; county: Ngorongoro; locality: Enja Shori; verbatimLocality: Ngorongoro Conservation Area, along Enja Shori,; minimumElevationInMeters: 1500; decimalLatitude: -3.033; decimalLongitude: 35.266; **Event:** eventDate: 1993-07-19; **Record Level:** institutionCode: AAU; collectionCode: Herbarium; ownerInstitutionCode: AAU; basisOfRecord: PreservedSpecimen

##### Distribution

Eastern & Southern Africa

#### Cynodon dactylon

(L.) Pers.

##### Materials

- **Type status:** Other material. **Occurrence:** catalogNumber: K000984212; recordNumber: 383; recordedBy: Paulo, S; **Taxon:** scientificName: *Cynodon dactylon* (L.) Pers.; kingdom: Plantae; family: Poaceae; genus: Cynodon; specificEpithet: dactylon; scientificNameAuthorship: (L.) Pers.; **Location:** continent: Africa; country: Tanzania; stateProvince: Arusha; county: Ngorongoro; locality: Salei Plain; decimalLatitude: -3.166667; decimalLongitude: 35.583333; **Event:** eventDate: 1958-04-26; **Record Level:** institutionCode: K; collectionCode: Herbarium; ownerInstitutionCode: K; basisOfRecord: PreservedSpecimen
- **Type status:** Other material. **Occurrence:** catalogNumber: K000984213; recordNumber: 321; recordedBy: Paulo, S; **Taxon:** scientificName: *Cynodon dactylon* (L.) Pers.; kingdom: Plantae; family: Poaceae; genus: Cynodon; specificEpithet: dactylon; scientificNameAuthorship: (L.) Pers.; **Location:** continent: Africa; country: Tanzania; stateProvince: Arusha; county: Ngorongoro; locality: Olbalbal; minimumElevationInMeters: 1250; decimalLatitude: -2.95; decimalLongitude: 35.283333; **Event:** eventDate: 1958-04-20; **Record Level:** institutionCode: K; collectionCode: Herbarium; ownerInstitutionCode: K; basisOfRecord: PreservedSpecimen
- **Type status:** Other material. **Occurrence:** catalogNumber: K000984225; recordNumber: 6315; recordedBy: Newbould, JB; **Taxon:** scientificName: *Cynodon dactylon* (L.) Pers.; kingdom: Plantae; family: Poaceae; genus: Cynodon; specificEpithet: dactylon; scientificNameAuthorship: (L.) Pers.; **Location:** continent: Africa; country: Tanzania; stateProvince: Mara; county: Serengeti; locality: Engare Nanyuki; verbatimLocality: E. Serengeti; minimumElevationInMeters: 1707; decimalLatitude: -2.616667; decimalLongitude: 35.216667; **Event:** eventDate: 1962-11; **Record Level:** institutionCode: K; collectionCode: Herbarium; ownerInstitutionCode: K; basisOfRecord: PreservedSpecimen

##### Distribution

Widespread

#### Cynodon nlemfuensis

Vanderyst

##### Materials

- **Type status:** Other material. **Occurrence:** catalogNumber: K000984223; recordNumber: 19178; recordedBy: Raynal, J; **Taxon:** scientificName: *Cynodon nlemfuensis* Vanderyst; kingdom: Plantae; family: Poaceae; genus: Cynodon; specificEpithet: nlemfuensis; scientificNameAuthorship: Vanderyst; **Location:** continent: Africa; country: Tanzania; stateProvince: Arusha; county: Ngorongoro; locality: Ngorongoro Crater; verbatimLocality: Ngorongoro crater, South Rim, outer slopes, road to Karatu, 5 km before junction to Lemala; minimumElevationInMeters: 2350; decimalLatitude: -3.166667; decimalLongitude: 35.583333; **Event:** eventDate: 1977-09-20; **Record Level:** institutionCode: K; collectionCode: Herbarium; ownerInstitutionCode: K; basisOfRecord: PreservedSpecimen
- **Type status:** Other material. **Occurrence:** catalogNumber: K000984224; recordNumber: 19305; recordedBy: Raynal, J; **Taxon:** scientificName: *Cynodon nlemfuensis* Vanderyst; kingdom: Plantae; family: Poaceae; genus: Cynodon; specificEpithet: nlemfuensis; scientificNameAuthorship: Vanderyst; **Location:** continent: Africa; country: Tanzania; stateProvince: Arusha; county: Ngorongoro; locality: Olduvai; verbatimLocality: shifting sands 5-6 km NW of confluence of Main and Side Gorges; minimumElevationInMeters: 1580; decimalLatitude: -2.95; decimalLongitude: 35.166667; **Event:** eventDate: 1977-09-27; **Record Level:** institutionCode: K; collectionCode: Herbarium; ownerInstitutionCode: K; basisOfRecord: PreservedSpecimen

##### Distribution

Tropical Africa, introduced elsewhere

#### Cynodon nlemfuensisvar.nlemfuensis

##### Materials

- **Type status:** Other material. **Occurrence:** catalogNumber: K000984216; recordNumber: 2791; recordedBy: Chuwa, S; **Taxon:** scientificName: Cynodon nlemfuensis Vanderyst var. nlemfuensis; kingdom: Plantae; family: Poaceae; genus: Cynodon; specificEpithet: nlemfuensis; infraspecificEpithet: nlemfuensis; scientificNameAuthorship: Vanderyst; **Location:** continent: Africa; country: Tanzania; stateProvince: Arusha; county: Ngorongoro; locality: Olduvai Gorge; minimumElevationInMeters: 1400; decimalLatitude: -2.95; decimalLongitude: 35.166667; **Event:** eventDate: 1989-05-30; **Record Level:** institutionCode: K; collectionCode: Herbarium; ownerInstitutionCode: K; basisOfRecord: PreservedSpecimen
- **Type status:** Other material. **Occurrence:** catalogNumber: K000984217; recordNumber: 2867; recordedBy: Chuwa, S; **Taxon:** scientificName: Cynodon nlemfuensis Vanderyst var. nlemfuensis; kingdom: Plantae; family: Poaceae; genus: Cynodon; specificEpithet: nlemfuensis; infraspecificEpithet: nlemfuensis; scientificNameAuthorship: Vanderyst; **Location:** continent: Africa; country: Tanzania; stateProvince: Arusha; county: Ngorongoro; locality: Gol Kopjes; minimumElevationInMeters: 1679; decimalLatitude: -2.7; decimalLongitude: 35.433333; **Event:** eventDate: 1989-10-19; **Record Level:** institutionCode: K; collectionCode: Herbarium; ownerInstitutionCode: K; basisOfRecord: PreservedSpecimen
- **Type status:** Other material. **Occurrence:** catalogNumber: K000984218; recordNumber: 9859; recordedBy: Greenway, PJ; **Taxon:** scientificName: Cynodon nlemfuensis Vanderyst var. nlemfuensis; kingdom: Plantae; family: Poaceae; genus: Cynodon; specificEpithet: nlemfuensis; infraspecificEpithet: nlemfuensis; scientificNameAuthorship: Vanderyst; **Location:** continent: Africa; country: Tanzania; stateProvince: Mara; county: Serengeti; locality: Seronera; verbatimLocality: Serengeti; minimumElevationInMeters: 1554; decimalLatitude: -2.45; decimalLongitude: 34.833333; **Event:** eventDate: 1961-03-20; **Record Level:** institutionCode: K; collectionCode: Herbarium; ownerInstitutionCode: K; basisOfRecord: PreservedSpecimen
- **Type status:** Other material. **Occurrence:** catalogNumber: K000984219; recordNumber: 360; recordedBy: Paulo, S; **Taxon:** scientificName: Cynodon nlemfuensis Vanderyst var. nlemfuensis; kingdom: Plantae; family: Poaceae; genus: Cynodon; specificEpithet: nlemfuensis; infraspecificEpithet: nlemfuensis; scientificNameAuthorship: Vanderyst; **Location:** continent: Africa; country: Tanzania; stateProvince: Mara; county: Serengeti; locality: Engare Nanyuki; verbatimLocality: North of Engare Nanyuki, North Serengeti; decimalLatitude: -2.616667; decimalLongitude: 35.216667; **Event:** eventDate: 1958-04-23; **Record Level:** institutionCode: K; collectionCode: Herbarium; ownerInstitutionCode: K; basisOfRecord: PreservedSpecimen
- **Type status:** Other material. **Occurrence:** catalogNumber: K000984220; recordNumber: 80; recordedBy: Frame, GW; **Taxon:** scientificName: Cynodon nlemfuensis Vanderyst var. nlemfuensis; kingdom: Plantae; family: Poaceae; genus: Cynodon; specificEpithet: nlemfuensis; infraspecificEpithet: nlemfuensis; scientificNameAuthorship: Vanderyst; **Location:** continent: Africa; country: Tanzania; stateProvince: Arusha; county: Ngorongoro; locality: Ngorongoro Crater; verbatimLocality: floor; minimumElevationInMeters: 1520; decimalLatitude: -3.166667; decimalLongitude: 35.583333; **Event:** eventDate: 1972-12-12; **Record Level:** institutionCode: K; collectionCode: Herbarium; ownerInstitutionCode: K; basisOfRecord: PreservedSpecimen
- **Type status:** Other material. **Occurrence:** catalogNumber: K000984221; recordNumber: 95; recordedBy: Frame, GW; **Taxon:** scientificName: Cynodon nlemfuensis Vanderyst var. nlemfuensis; kingdom: Plantae; family: Poaceae; genus: Cynodon; specificEpithet: nlemfuensis; infraspecificEpithet: nlemfuensis; scientificNameAuthorship: Vanderyst; **Location:** continent: Africa; country: Tanzania; stateProvince: Arusha; county: Ngorongoro; locality: Empakai Crater; verbatimLocality: top east rim; minimumElevationInMeters: 2600; decimalLatitude: -2.933333; decimalLongitude: 35.816667; **Event:** eventDate: 1973-03-15; **Record Level:** institutionCode: K; collectionCode: Herbarium; ownerInstitutionCode: K; basisOfRecord: PreservedSpecimen
- **Type status:** Other material. **Occurrence:** catalogNumber: K000984222; recordNumber: 350; recordedBy: Paulo, S; **Taxon:** scientificName: Cynodon nlemfuensis Vanderyst var. nlemfuensis; kingdom: Plantae; family: Poaceae; genus: Cynodon; specificEpithet: nlemfuensis; infraspecificEpithet: nlemfuensis; scientificNameAuthorship: Vanderyst; **Location:** continent: Africa; country: Tanzania; stateProvince: Arusha; county: Ngorongoro; locality: Lemuta hill; verbatimLocality: South of Lemuta hill; decimalLatitude: -2.7; decimalLongitude: 35.266667; **Event:** eventDate: 1958-04-22; **Record Level:** institutionCode: K; collectionCode: Herbarium; ownerInstitutionCode: K; basisOfRecord: PreservedSpecimen

##### Distribution

Tropical Africa, introduced elsewhere

#### Cynodon nlemfuensisvar.robustus

Clayton & J.R.Harlan

##### Materials

- **Type status:** Other material. **Occurrence:** catalogNumber: K000984215; recordNumber: 300; recordedBy: Paulo, S; **Taxon:** scientificName: Cynodon nlemfuensis Vanderyst var. robustus Clayton & J.R.Harlan; kingdom: Plantae; family: Poaceae; genus: Cynodon; specificEpithet: nlemfuensis; infraspecificEpithet: robustus; scientificNameAuthorship: Clayton & J.R.Harlan; **Location:** continent: Africa; country: Tanzania; stateProvince: Mara; county: Serengeti; locality: Seronera; decimalLatitude: -2.45; decimalLongitude: 34.833333; **Event:** eventDate: 1958-04-18; **Record Level:** institutionCode: K; collectionCode: Herbarium; ownerInstitutionCode: K; basisOfRecord: PreservedSpecimen

##### Distribution

Tropical Africa

#### Cynodon plectostachyus

(K.Schum.) Pilg.

##### Materials

- **Type status:** Other material. **Occurrence:** catalogNumber: K000984210; recordNumber: 2790; recordedBy: Chuwa, S; **Taxon:** scientificName: *Cynodon plectostachyus* (K.Schum.) Pilg.; kingdom: Plantae; family: Poaceae; genus: Cynodon; specificEpithet: plectostachyus; scientificNameAuthorship: (K.Schum.) Pilg.; **Location:** continent: Africa; country: Tanzania; stateProvince: Arusha; county: Ngorongoro; locality: Olduvai Gorge; minimumElevationInMeters: 1400; decimalLatitude: -2.95; decimalLongitude: 35.166667; **Event:** eventDate: 1989-05-30; **Record Level:** institutionCode: K; collectionCode: Herbarium; ownerInstitutionCode: K; basisOfRecord: PreservedSpecimen
- **Type status:** Other material. **Occurrence:** catalogNumber: K000984211; recordNumber: 10190; recordedBy: Greenway, PJ; **Taxon:** scientificName: *Cynodon plectostachyus* (K.Schum.) Pilg.; kingdom: Plantae; family: Poaceae; genus: Cynodon; specificEpithet: plectostachyus; scientificNameAuthorship: (K.Schum.) Pilg.; **Location:** continent: Africa; country: Tanzania; stateProvince: Mara; county: Serengeti; locality: Seronera; verbatimLocality: Seronera lodge, Serengeti; minimumElevationInMeters: 1448; decimalLatitude: -2.45; decimalLongitude: 34.833333; **Event:** eventDate: 1961-05-16; **Record Level:** institutionCode: K; collectionCode: Herbarium; ownerInstitutionCode: K; basisOfRecord: PreservedSpecimen
- **Type status:** Other material. **Occurrence:** catalogNumber: K000984214; recordNumber: 9855; recordedBy: Greenway, PJ; **Taxon:** scientificName: *Cynodon plectostachyus* (K.Schum.) Pilg.; kingdom: Plantae; family: Poaceae; genus: Cynodon; specificEpithet: plectostachyus; scientificNameAuthorship: (K.Schum.) Pilg.; **Location:** continent: Africa; country: Tanzania; stateProvince: Mara; county: Serengeti; locality: Seronera; verbatimLocality: Serengeti; minimumElevationInMeters: 1554; decimalLatitude: -2.45; decimalLongitude: 34.833333; **Event:** eventDate: 1961-03-20; **Record Level:** institutionCode: K; collectionCode: Herbarium; ownerInstitutionCode: K; basisOfRecord: PreservedSpecimen
- **Type status:** Other material. **Occurrence:** catalogNumber: 580; recordNumber: s.n.; recordedBy: Mboya, E; **Taxon:** scientificName: *Cynodon plectostachyus* (K.Schum.) Pilg.; kingdom: Plantae; family: Poaceae; genus: Cynodon; specificEpithet: plectostachyus; scientificNameAuthorship: (K.Schum.) Pilg.; **Location:** continent: Africa; country: Tanzania; stateProvince: Mara; county: Serengeti; locality: Seronera Lodge; verbatimLocality: T1. Area around Frankfurt Zoological Society Headquarters and Seronera Lodge.; minimumElevationInMeters: 1512; decimalLatitude: -2.44; decimalLongitude: 34.82; **Event:** eventDate: 2004-02-09; **Record Level:** institutionCode: MO; collectionCode: Herbarium; ownerInstitutionCode: MO; basisOfRecord: PreservedSpecimen
- **Type status:** Other material. **Occurrence:** catalogNumber: K001087092; recordNumber: 410; recordedBy: Paulo, S; **Taxon:** scientificName: *Cynodon plectostachyus* (K.Schum.) Pilg.; kingdom: Plantae; family: Poaceae; genus: Cynodon; specificEpithet: plectostachyus; scientificNameAuthorship: (K.Schum.) Pilg.; **Location:** continent: Africa; country: Tanzania; stateProvince: Arusha; county: Ngorongoro; locality: Olbalbal; verbatimLocality: North of; decimalLatitude: -2.95; decimalLongitude: 35.583333; **Event:** eventDate: 1958-04-29; **Record Level:** institutionCode: K; collectionCode: Herbarium; ownerInstitutionCode: K; basisOfRecord: PreservedSpecimen
- **Type status:** Other material. **Occurrence:** catalogNumber: K001087093; recordNumber: 6356; recordedBy: Leippert; **Taxon:** scientificName: *Cynodon plectostachyus* (K.Schum.) Pilg.; kingdom: Plantae; family: Poaceae; genus: Cynodon; specificEpithet: plectostachyus; scientificNameAuthorship: (K.Schum.) Pilg.; **Location:** continent: Africa; country: Tanzania; stateProvince: Arusha; county: Ngorongoro; locality: Engaruka; decimalLatitude: -2.983333; decimalLongitude: 35.616667; **Event:** eventDate: 1966-02-24; **Record Level:** institutionCode: K; collectionCode: Herbarium; ownerInstitutionCode: K; basisOfRecord: PreservedSpecimen

##### Distribution

Eastern Africa, introduced elsewhere

#### Dactyloctenium sp.

##### Materials

- **Type status:** Other material. **Occurrence:** catalogNumber: 590; recordNumber: 24306; recordedBy: Peterson, PM; Soreng, RJ; Romaschenko, K; Mbago, F; **Taxon:** scientificName: Dactyloctenium; kingdom: Plantae; family: Poaceae; genus: Dactyloctenium; **Location:** continent: Africa; country: Tanzania; stateProvince: Arusha; county: Ngorongoro; locality: Ngorongoro Crater; verbatimLocality: Ngorongoro Conservation Area, rim of Ngorongoro Crater (descent gate).; minimumElevationInMeters: 2168; decimalLatitude: -3.15462; decimalLongitude: 35.47717; **Event:** eventDate: 2012-06-19; **Record Level:** institutionCode: US; collectionCode: Herbarium; ownerInstitutionCode: US; basisOfRecord: PreservedSpecimen

#### Dactyloctenium aegyptium

(L.) Willd.

##### Materials

- **Type status:** Other material. **Occurrence:** catalogNumber: 586; recordNumber: s.n.; recordedBy: Mboya, E; **Taxon:** scientificName: *Dactyloctenium aegyptium* (L.) Willd.; kingdom: Plantae; family: Poaceae; genus: Dactyloctenium; specificEpithet: aegyptium; scientificNameAuthorship: (L.) Willd.; **Location:** continent: Africa; country: Tanzania; stateProvince: Mara; county: Serengeti; locality: Seronera Lodge; verbatimLocality: T1. Area around Frankfurt Zoological Society Headquarters and Seronera Lodge.; minimumElevationInMeters: 1512; decimalLatitude: -2.44; decimalLongitude: 34.82; **Event:** eventDate: 2004-02-09; **Record Level:** institutionCode: MO; collectionCode: Herbarium; ownerInstitutionCode: MO; basisOfRecord: PreservedSpecimen

##### Distribution

Widespread

#### Dactyloctenium geminatum

Hack.

Dactyloctenium bogdanii S.M.Phillips

##### Materials

- **Type status:** Other material. **Occurrence:** catalogNumber: 587; recordNumber: 487; recordedBy: Ellemann, L; **Taxon:** scientificName: *Dactyloctenium bogdanii* S.M.Phillips; kingdom: Plantae; family: Poaceae; genus: Dactyloctenium; specificEpithet: bogdanii; scientificNameAuthorship: S.M.Phillips; **Location:** continent: Africa; country: Tanzania; stateProvince: Arusha; county: Ngorongoro; locality: Orkuman; verbatimLocality: Ngorongoro Conservation Area. Along a dry riverine close to Orkuman river; minimumElevationInMeters: 1550; decimalLatitude: -3.033; decimalLongitude: 35.283; **Event:** eventDate: 1993-05-03; **Record Level:** institutionCode: AAU; collectionCode: Herbarium; ownerInstitutionCode: AAU; basisOfRecord: PreservedSpecimen
- **Type status:** Other material. **Occurrence:** catalogNumber: 588; recordNumber: 707; recordedBy: Ellemann, L; **Taxon:** scientificName: *Dactyloctenium bogdanii* S.M.Phillips; kingdom: Plantae; family: Poaceae; genus: Dactyloctenium; specificEpithet: bogdanii; scientificNameAuthorship: S.M.Phillips; **Location:** continent: Africa; country: Tanzania; stateProvince: Arusha; county: Ngorongoro; locality: Enja Shori; verbatimLocality: Ngorongoro Conservation Area, along Enja Shori,; minimumElevationInMeters: 1500; decimalLatitude: -3.033; decimalLongitude: 35.266; **Event:** eventDate: 1993-07-19; **Record Level:** institutionCode: AAU; collectionCode: Herbarium; ownerInstitutionCode: AAU; basisOfRecord: PreservedSpecimen
- **Type status:** Other material. **Occurrence:** catalogNumber: 589; recordNumber: 1128; recordedBy: Ellemann, L; **Taxon:** scientificName: *Dactyloctenium bogdanii* S.M.Phillips; kingdom: Plantae; family: Poaceae; genus: Dactyloctenium; specificEpithet: bogdanii; scientificNameAuthorship: S.M.Phillips; **Location:** continent: Africa; country: Tanzania; stateProvince: Arusha; county: Ngorongoro; locality: Oldonyo-o-ogol; verbatimLocality: Ngorongoro Conservation Area, Oldonyo-o-ogol.; minimumElevationInMeters: 1700; decimalLatitude: -2.75; decimalLongitude: 35.45; **Event:** eventDate: 1994-03-07; **Record Level:** institutionCode: AAU; collectionCode: Herbarium; ownerInstitutionCode: AAU; basisOfRecord: PreservedSpecimen

##### Distribution

Eastern & Southern Africa

#### Deschampsia flexuosa

(L.) Trin.

Aira caryophyllaea Leers

##### Materials

- **Type status:** Other material. **Occurrence:** catalogNumber: 852; recordNumber: 6097; recordedBy: Newbould, JB; **Taxon:** scientificName: *Aira caryophyllaea* Leers; kingdom: Plantae; family: Poaceae; genus: Aira; specificEpithet: caryophyllaea; scientificNameAuthorship: Leers; **Location:** continent: Africa; country: Tanzania; stateProvince: Arusha; county: Ngorongoro; locality: Oldeani Mt; decimalLatitude: -3.266667; decimalLongitude: 35.433333; **Event:** eventDate: 1962-06-14; **Record Level:** collectionCode: Herbarium; ownerInstitutionCode: ?; basisOfRecord: PreservedSpecimen

##### Distribution

Widespread

#### Dichanthium annulatum

(Forssk.) Stapf

##### Materials

- **Type status:** Other material. **Occurrence:** catalogNumber: 591; recordNumber: s.n.; recordedBy: Mboya, E; **Taxon:** scientificName: *Dichanthium annulatum* (Forssk.) Stapf; kingdom: Plantae; family: Poaceae; genus: Dichanthium; specificEpithet: annulatum; scientificNameAuthorship: (Forssk.) Stapf; **Location:** continent: Africa; country: Tanzania; stateProvince: Mara; county: Serengeti; locality: Serengeti National Park; verbatimLocality: T1. Serengeti National Park. Ndabaka-Seronera Road.; minimumElevationInMeters: 1417; decimalLatitude: -2.32; decimalLongitude: 34.73; **Event:** eventDate: 2004-02-07; **Record Level:** institutionCode: MO; collectionCode: Herbarium; ownerInstitutionCode: MO; basisOfRecord: PreservedSpecimen

##### Distribution

Widespread

#### Dichanthium annulatumvar.annulatum

##### Materials

- **Type status:** Other material. **Occurrence:** catalogNumber: 1147; recordNumber: 819B; recordedBy: Mollel, NP; Rusch, GM; Mwakalebe, G; **Taxon:** scientificName: Dichanthium annulatum (Forssk.) Stapf var. annulatum; kingdom: Plantae; family: Poaceae; genus: Dichanthium; specificEpithet: annulatum; infraspecificEpithet: annulatum; scientificNameAuthorship: (Forssk.) Stapf; **Location:** continent: Africa; country: Tanzania; stateProvince: Mara; county: Serengeti; locality: Robanda village; verbatimLocality: Robanda village, road to the water pool, CG01 plot 36M, 0386088, 9763754 UTM.; minimumElevationInMeters: 1388; decimalLatitude: -2.083333; decimalLongitude: 34.666667; **Event:** eventDate: 2003-01-28; **Record Level:** institutionCode: NHT; collectionCode: Herbarium; ownerInstitutionCode: NHT; basisOfRecord: PreservedSpecimen

##### Distribution

Widespread

#### Digitaria sp.

##### Materials

- **Type status:** Other material. **Occurrence:** catalogNumber: 597; recordNumber: 482; recordedBy: Ellemann, L; **Taxon:** scientificName: Digitaria; kingdom: Plantae; family: Poaceae; genus: Digitaria; **Location:** continent: Africa; country: Tanzania; stateProvince: Arusha; county: Ngorongoro; locality: Kelogi; verbatimLocality: Ngorongoro Conservation Area, Kelogi.; minimumElevationInMeters: 1550; decimalLatitude: -3.033; decimalLongitude: 35.283; **Event:** eventDate: 1993-05-03; **Record Level:** institutionCode: AAU; collectionCode: Herbarium; ownerInstitutionCode: AAU; basisOfRecord: PreservedSpecimen
- **Type status:** Other material. **Occurrence:** catalogNumber: 598; recordNumber: 709; recordedBy: Ellemann, L; **Taxon:** scientificName: Digitaria; kingdom: Plantae; family: Poaceae; genus: Digitaria; **Location:** continent: Africa; country: Tanzania; stateProvince: Arusha; county: Ngorongoro; locality: Enja Shori; verbatimLocality: Ngorongoro Conservation Area, along Enja Shori,; minimumElevationInMeters: 1500; decimalLatitude: -3.033; decimalLongitude: 35.266; **Event:** eventDate: 1993-07-19; **Record Level:** institutionCode: AAU; collectionCode: Herbarium; ownerInstitutionCode: AAU; basisOfRecord: PreservedSpecimen
- **Type status:** Other material. **Occurrence:** catalogNumber: 599; recordNumber: 880; recordedBy: Ellemann, L; **Taxon:** scientificName: Digitaria; kingdom: Plantae; family: Poaceae; genus: Digitaria; **Location:** continent: Africa; country: Tanzania; stateProvince: Arusha; county: Ngorongoro; locality: Olmekeke; verbatimLocality: Ngorongoro Conservation Area, below the lower water tank at Olmekeke pipeline.; minimumElevationInMeters: 1700; decimalLatitude: -3.25; decimalLongitude: 35.516; **Event:** eventDate: 1993-09-09; **Record Level:** institutionCode: AAU; collectionCode: Herbarium; ownerInstitutionCode: AAU; basisOfRecord: PreservedSpecimen
- **Type status:** Other material. **Occurrence:** catalogNumber: 600; recordNumber: 1094; recordedBy: Ellemann, L; **Taxon:** scientificName: Digitaria; kingdom: Plantae; family: Poaceae; genus: Digitaria; **Location:** continent: Africa; country: Tanzania; stateProvince: Arusha; county: Ngorongoro; locality: Olbalbal swamp; verbatimLocality: Ngorongoro Conservation Area, south-western part of Olbalbal swamp.; minimumElevationInMeters: 1350; decimalLatitude: -3.05; decimalLongitude: 35.466; **Event:** eventDate: 1994-03-03; **Record Level:** institutionCode: AAU; collectionCode: Herbarium; ownerInstitutionCode: AAU; basisOfRecord: PreservedSpecimen
- **Type status:** Other material. **Occurrence:** catalogNumber: 601; recordNumber: 1119; recordedBy: Ellemann, L; **Taxon:** scientificName: Digitaria; kingdom: Plantae; family: Poaceae; genus: Digitaria; **Location:** continent: Africa; country: Tanzania; stateProvince: Arusha; county: Ngorongoro; locality: Oldonyo-o-ogol; verbatimLocality: Ngorongoro Conservation Area, Oldonyo-o-ogol.; minimumElevationInMeters: 1700; decimalLatitude: -2.75; decimalLongitude: 35.45; **Event:** eventDate: 1994-03-07; **Record Level:** institutionCode: AAU; collectionCode: Herbarium; ownerInstitutionCode: AAU; basisOfRecord: PreservedSpecimen

#### Digitaria abyssinica

(Hochst. ex A.Rich.) Stapf

Digitaria scalarum (Schweinf.) Chiov.

##### Materials

- **Type status:** Other material. **Occurrence:** catalogNumber: 306; recordNumber: 283; recordedBy: Braun, HMH; **Taxon:** scientificName: *Digitaria abyssinica* (Hochst. ex A.Rich.) Stapf; kingdom: Plantae; family: Poaceae; genus: Digitaria; specificEpithet: abyssinica; scientificNameAuthorship: (Hochst. ex A.Rich.) Stapf; **Location:** continent: Africa; country: Tanzania; stateProvince: Mara; county: Serengeti; locality: Serengeti National Park; verbatimLocality: 1km South of South East Kopjes.; minimumElevationInMeters: 1500; decimalLatitude: -2.033333; decimalLongitude: 34.833333; **Event:** eventDate: 1968-04-15; **Record Level:** institutionCode: EA; collectionCode: Herbarium; ownerInstitutionCode: EA; basisOfRecord: PreservedSpecimen
- **Type status:** Other material. **Occurrence:** catalogNumber: K001087106; recordNumber: 10488; recordedBy: Greenway, PJ; **Taxon:** scientificName: *Digitaria abyssinica* (Hochst. ex A.Rich.) Stapf; kingdom: Plantae; family: Poaceae; genus: Digitaria; specificEpithet: abyssinica; scientificNameAuthorship: (Hochst. ex A.Rich.) Stapf; **Location:** continent: Africa; country: Tanzania; stateProvince: Mara; county: Serengeti; locality: Serengeti National Park; verbatimLocality: Eastern boundary, Serengeti 10.6miles from Soitayai.; minimumElevationInMeters: 1646; decimalLatitude: -2.333333; decimalLongitude: 34.833333; **Event:** eventDate: 1962-03-01; **Record Level:** institutionCode: K; collectionCode: Herbarium; ownerInstitutionCode: K; basisOfRecord: PreservedSpecimen
- **Type status:** Other material. **Occurrence:** catalogNumber: K001087110; recordNumber: 372; recordedBy: Paulo, S; **Taxon:** scientificName: *Digitaria abyssinica* (Hochst. ex A.Rich.) Stapf; kingdom: Plantae; family: Poaceae; genus: Digitaria; specificEpithet: abyssinica; scientificNameAuthorship: (Hochst. ex A.Rich.) Stapf; **Location:** continent: Africa; country: Tanzania; stateProvince: Mara; county: Serengeti; locality: Serengeti Plains; verbatimLocality: Central Serengeti; decimalLatitude: -2.333333; decimalLongitude: 34.833333; **Event:** eventDate: 1958-04-24; **Record Level:** institutionCode: K; collectionCode: Herbarium; ownerInstitutionCode: K; basisOfRecord: PreservedSpecimen
- **Type status:** Other material. **Occurrence:** catalogNumber: K001087109; recordNumber: 6304; recordedBy: Newbould, JB; **Taxon:** scientificName: *Digitaria abyssinica* (Hochst. ex A.Rich.) Stapf; kingdom: Plantae; family: Poaceae; genus: Digitaria; specificEpithet: abyssinica; scientificNameAuthorship: (Hochst. ex A.Rich.) Stapf; **Location:** continent: Africa; country: Tanzania; stateProvince: Mara; county: Serengeti; locality: Engare Nanyuki; verbatimLocality: Engare Nanyuki, Serengeti.; minimumElevationInMeters: 1768; decimalLatitude: -2.616667; decimalLongitude: 35.216667; **Event:** eventDate: 1962-11-22; **Record Level:** institutionCode: K; collectionCode: Herbarium; ownerInstitutionCode: K; basisOfRecord: PreservedSpecimen
- **Type status:** Other material. **Occurrence:** catalogNumber: 310; recordNumber: 6617; recordedBy: Vesey-FitzGerald, LDEF; **Taxon:** scientificName: *Digitaria abyssinica* (Hochst. ex A.Rich.) Stapf; kingdom: Plantae; family: Poaceae; genus: Digitaria; specificEpithet: abyssinica; scientificNameAuthorship: (Hochst. ex A.Rich.) Stapf; **Location:** continent: Africa; country: Tanzania; stateProvince: Mara; county: Serengeti; locality: Simba Kopjes; verbatimLocality: Seronera -Naabi hill road. 6 miles North of Simba Kopje.; minimumElevationInMeters: 1585; decimalLatitude: -2.633333; decimalLongitude: 34.916667; **Event:** eventDate: 1970-03-18; **Record Level:** institutionCode: EA; collectionCode: Herbarium; ownerInstitutionCode: EA; basisOfRecord: PreservedSpecimen
- **Type status:** Other material. **Occurrence:** catalogNumber: 592; recordNumber: 370; recordedBy: Ellemann, L; **Taxon:** scientificName: *Digitaria abyssinica* (Hochst. ex A.Rich.) Stapf; kingdom: Plantae; family: Poaceae; genus: Digitaria; specificEpithet: abyssinica; scientificNameAuthorship: (Hochst. ex A.Rich.) Stapf; **Location:** continent: Africa; country: Tanzania; stateProvince: Arusha; county: Ngorongoro; locality: Endulen; verbatimLocality: Ngorongoro Conservation Area. Endulen Hospital.; minimumElevationInMeters: 1900; decimalLatitude: -3.2; decimalLongitude: 35.266; **Event:** eventDate: 1993-04-20; **Record Level:** institutionCode: AAU; collectionCode: Herbarium; ownerInstitutionCode: AAU; basisOfRecord: PreservedSpecimen
- **Type status:** Other material. **Occurrence:** catalogNumber: 593; recordNumber: 24321; recordedBy: Peterson, PM; Soreng, RJ; Romaschenko, K; Mbago, F; **Taxon:** scientificName: *Digitaria abyssinica* (Hochst. ex A.Rich.) Stapf; kingdom: Plantae; family: Poaceae; genus: Digitaria; specificEpithet: abyssinica; scientificNameAuthorship: (Hochst. ex A.Rich.) Stapf; **Location:** continent: Africa; country: Tanzania; stateProvince: Arusha; county: Ngorongoro; locality: Lake Magadi; verbatimLocality: Ngorongoro Conservation Area, W side of Lake Magadi.; minimumElevationInMeters: 1739; decimalLatitude: -3.17713; decimalLongitude: 35.51548; **Event:** eventDate: 2012-06-19; **Record Level:** institutionCode: US; collectionCode: Herbarium; ownerInstitutionCode: US; basisOfRecord: PreservedSpecimen
- **Type status:** Other material. **Occurrence:** catalogNumber: 829; recordNumber: 10488; recordedBy: Greenway, PJ; **Taxon:** scientificName: *Digitaria abyssinica* (Hochst. ex A.Rich.) Stapf; kingdom: Plantae; family: Poaceae; genus: Digitaria; specificEpithet: abyssinica; scientificNameAuthorship: (Hochst. ex A.Rich.) Stapf; **Location:** continent: Africa; country: Tanzania; stateProvince: Mara; county: Serengeti; locality: Serengeti National Park; verbatimLocality: Eastern boundary, Serengeti 10.6miles from Soitayai.; minimumElevationInMeters: 1646; decimalLatitude: -2.333333; decimalLongitude: 34.833333; **Event:** eventDate: 1962-03-01; **Record Level:** institutionCode: EA; collectionCode: Herbarium; ownerInstitutionCode: EA; basisOfRecord: PreservedSpecimen
- **Type status:** Other material. **Occurrence:** catalogNumber: 830; recordNumber: 372; recordedBy: Paulo, S; **Taxon:** scientificName: *Digitaria abyssinica* (Hochst. ex A.Rich.) Stapf; kingdom: Plantae; family: Poaceae; genus: Digitaria; specificEpithet: abyssinica; scientificNameAuthorship: (Hochst. ex A.Rich.) Stapf; **Location:** continent: Africa; country: Tanzania; stateProvince: Mara; county: Serengeti; locality: Serengeti Plains; verbatimLocality: Central Serengeti; decimalLatitude: -2.333333; decimalLongitude: 34.833333; **Event:** eventDate: 1958-04-24; **Record Level:** institutionCode: EA; collectionCode: Herbarium; ownerInstitutionCode: EA; basisOfRecord: PreservedSpecimen
- **Type status:** Other material. **Occurrence:** catalogNumber: 831; recordNumber: 6304; recordedBy: Newbould, JB; **Taxon:** scientificName: *Digitaria abyssinica* (Hochst. ex A.Rich.) Stapf; kingdom: Plantae; family: Poaceae; genus: Digitaria; specificEpithet: abyssinica; scientificNameAuthorship: (Hochst. ex A.Rich.) Stapf; **Location:** continent: Africa; country: Tanzania; stateProvince: Mara; county: Serengeti; locality: Engare Nanyuki; verbatimLocality: Engare Nanyuki, Serengeti.; minimumElevationInMeters: 1768; decimalLatitude: -2.616667; decimalLongitude: 35.216667; **Event:** eventDate: 1962-11-22; **Record Level:** institutionCode: EA; collectionCode: Herbarium; ownerInstitutionCode: EA; basisOfRecord: PreservedSpecimen
- **Type status:** Other material. **Occurrence:** catalogNumber: K001087105; recordNumber: 3366; recordedBy: Greenway, PJ; **Taxon:** scientificName: *Digitaria abyssinica* (Hochst. ex A.Rich.) Stapf; kingdom: Plantae; family: Poaceae; genus: Digitaria; specificEpithet: abyssinica; scientificNameAuthorship: (Hochst. ex A.Rich.) Stapf; **Location:** continent: Africa; country: Tanzania; stateProvince: Arusha; county: Ngorongoro; locality: Ngorongoro Crater; verbatimLocality: On SE slope of crater; minimumElevationInMeters: 2439; decimalLatitude: -3.166667; decimalLongitude: 35.583333; **Event:** eventDate: 1933-02-22; **Record Level:** institutionCode: K; collectionCode: Herbarium; ownerInstitutionCode: K; basisOfRecord: PreservedSpecimen
- **Type status:** Other material. **Occurrence:** catalogNumber: 1001; recordNumber: 3366; recordedBy: Greenway, PJ; **Taxon:** scientificName: *Digitaria abyssinica* (Hochst. ex A.Rich.) Stapf; kingdom: Plantae; family: Poaceae; genus: Digitaria; specificEpithet: abyssinica; scientificNameAuthorship: (Hochst. ex A.Rich.) Stapf; **Location:** continent: Africa; country: Tanzania; stateProvince: Arusha; county: Ngorongoro; locality: Ngorongoro Crater; verbatimLocality: On SE slope of crater; minimumElevationInMeters: 2439; decimalLatitude: -3.166667; decimalLongitude: 35.583333; **Event:** eventDate: 1933-02-22; **Record Level:** institutionCode: EA; collectionCode: Herbarium; ownerInstitutionCode: EA; basisOfRecord: PreservedSpecimen
- **Type status:** Other material. **Occurrence:** catalogNumber: 1002; recordNumber: E27; recordedBy: Gilbert, VC; **Taxon:** scientificName: *Digitaria abyssinica* (Hochst. ex A.Rich.) Stapf; kingdom: Plantae; family: Poaceae; genus: Digitaria; specificEpithet: abyssinica; scientificNameAuthorship: (Hochst. ex A.Rich.) Stapf; **Location:** continent: Africa; country: Tanzania; stateProvince: Arusha; county: Ngorongoro; locality: Ngorongoro Crater; verbatimLocality: crater floor; minimumElevationInMeters: 1737; decimalLatitude: -3.166667; decimalLongitude: 35.583333; **Event:** eventDate: 1966-10-10; **Record Level:** institutionCode: EA; collectionCode: Herbarium; ownerInstitutionCode: EA; basisOfRecord: PreservedSpecimen
- **Type status:** Other material. **Occurrence:** catalogNumber: 1003; recordNumber: 510; recordedBy: Frame, GW; **Taxon:** scientificName: *Digitaria abyssinica* (Hochst. ex A.Rich.) Stapf; kingdom: Plantae; family: Poaceae; genus: Digitaria; specificEpithet: abyssinica; scientificNameAuthorship: (Hochst. ex A.Rich.) Stapf; **Location:** continent: Africa; country: Tanzania; stateProvince: Arusha; county: Ngorongoro; locality: Empakai Crater; verbatimLocality: Empakaai crater, outer eastern slope.; minimumElevationInMeters: 2200; decimalLatitude: -2.933333; decimalLongitude: 35.816667; **Event:** eventDate: 1974-01-26; **Record Level:** institutionCode: EA; collectionCode: Herbarium; ownerInstitutionCode: EA; basisOfRecord: PreservedSpecimen
- **Type status:** Other material. **Occurrence:** catalogNumber: K001087107; recordNumber: 147; recordedBy: Frame, GW; **Taxon:** scientificName: *Digitaria abyssinica* (Hochst. ex A.Rich.) Stapf; kingdom: Plantae; family: Poaceae; genus: Digitaria; specificEpithet: abyssinica; scientificNameAuthorship: (Hochst. ex A.Rich.) Stapf; **Location:** continent: Africa; country: Tanzania; stateProvince: Arusha; county: Ngorongoro; locality: Empakai Crater; verbatimLocality: Empakaai crater floor north of main lake; minimumElevationInMeters: 2200; decimalLatitude: -2.933333; decimalLongitude: 35.816667; **Event:** eventDate: 1973-05-30; **Record Level:** institutionCode: K; collectionCode: Herbarium; ownerInstitutionCode: K; basisOfRecord: PreservedSpecimen
- **Type status:** Other material. **Occurrence:** catalogNumber: 1005; recordNumber: 147; recordedBy: Frame, GW; **Taxon:** scientificName: *Digitaria abyssinica* (Hochst. ex A.Rich.) Stapf; kingdom: Plantae; family: Poaceae; genus: Digitaria; specificEpithet: abyssinica; scientificNameAuthorship: (Hochst. ex A.Rich.) Stapf; **Location:** continent: Africa; country: Tanzania; stateProvince: Arusha; county: Ngorongoro; locality: Empakai Crater; verbatimLocality: Empakaai crater floor north of main lake; minimumElevationInMeters: 2200; decimalLatitude: -2.933333; decimalLongitude: 35.816667; **Event:** eventDate: 1973-05-30; **Record Level:** institutionCode: EA; collectionCode: Herbarium; ownerInstitutionCode: EA; basisOfRecord: PreservedSpecimen
- **Type status:** Other material. **Occurrence:** catalogNumber: 1006; recordNumber: 535; recordedBy: Frame, GW; **Taxon:** scientificName: *Digitaria abyssinica* (Hochst. ex A.Rich.) Stapf; kingdom: Plantae; family: Poaceae; genus: Digitaria; specificEpithet: abyssinica; scientificNameAuthorship: (Hochst. ex A.Rich.) Stapf; **Location:** continent: Africa; country: Tanzania; stateProvince: Arusha; county: Ngorongoro; locality: Empakai Crater; verbatimLocality: Empakaai crater, outer northern slope.; minimumElevationInMeters: 2200; decimalLatitude: -2.933333; decimalLongitude: 35.816667; **Event:** eventDate: 1974-02-01; **Record Level:** institutionCode: EA; collectionCode: Herbarium; ownerInstitutionCode: EA; basisOfRecord: PreservedSpecimen
- **Type status:** Other material. **Occurrence:** catalogNumber: 1007; recordNumber: 81; recordedBy: Goddard, J; **Taxon:** scientificName: *Digitaria abyssinica* (Hochst. ex A.Rich.) Stapf; kingdom: Plantae; family: Poaceae; genus: Digitaria; specificEpithet: abyssinica; scientificNameAuthorship: (Hochst. ex A.Rich.) Stapf; **Location:** continent: Africa; country: Tanzania; stateProvince: Arusha; county: Ngorongoro; locality: Ngorongoro Crater; minimumElevationInMeters: 1700; decimalLatitude: -3.166667; decimalLongitude: 35.583333; **Event:** eventDate: 1965-03-04; **Record Level:** institutionCode: EA; collectionCode: Herbarium; ownerInstitutionCode: EA; basisOfRecord: PreservedSpecimen
- **Type status:** Other material. **Occurrence:** catalogNumber: 1008; recordNumber: 89; recordedBy: Goddard, J; **Taxon:** scientificName: *Digitaria abyssinica* (Hochst. ex A.Rich.) Stapf; kingdom: Plantae; family: Poaceae; genus: Digitaria; specificEpithet: abyssinica; scientificNameAuthorship: (Hochst. ex A.Rich.) Stapf; **Location:** continent: Africa; country: Tanzania; stateProvince: Arusha; county: Ngorongoro; locality: Ngorongoro Crater; decimalLatitude: -3.166667; decimalLongitude: 35.583333; **Event:** eventDate: 1965-04-07; **Record Level:** institutionCode: EA; collectionCode: Herbarium; ownerInstitutionCode: EA; basisOfRecord: PreservedSpecimen
- **Type status:** Other material. **Occurrence:** catalogNumber: 1009; recordNumber: 97; recordedBy: Goddard, J; **Taxon:** scientificName: *Digitaria abyssinica* (Hochst. ex A.Rich.) Stapf; kingdom: Plantae; family: Poaceae; genus: Digitaria; specificEpithet: abyssinica; scientificNameAuthorship: (Hochst. ex A.Rich.) Stapf; **Location:** continent: Africa; country: Tanzania; stateProvince: Arusha; county: Ngorongoro; locality: Ngorongoro Crater; minimumElevationInMeters: 1700; decimalLatitude: -3.166667; decimalLongitude: 35.583333; **Event:** eventDate: 1965-04-08; **Record Level:** institutionCode: EA; collectionCode: Herbarium; ownerInstitutionCode: EA; basisOfRecord: PreservedSpecimen
- **Type status:** Other material. **Occurrence:** catalogNumber: K001087108; recordNumber: 972; recordedBy: Pole Evans, IB; Erens, J; **Taxon:** scientificName: *Digitaria abyssinica* (Hochst. ex A.Rich.) Stapf; kingdom: Plantae; family: Poaceae; genus: Digitaria; specificEpithet: abyssinica; scientificNameAuthorship: (Hochst. ex A.Rich.) Stapf; **Location:** continent: Africa; country: Tanzania; stateProvince: Arusha; county: Ngorongoro; locality: Ngorongoro Crater; verbatimLocality: At the top of Ngorongoro crater.; decimalLatitude: -3.166667; decimalLongitude: 35.583333; **Event:** eventDate: 1936-06-24; **Record Level:** institutionCode: K; collectionCode: Herbarium; ownerInstitutionCode: K; basisOfRecord: PreservedSpecimen
- **Type status:** Other material. **Occurrence:** catalogNumber: K001087111; recordNumber: 4155; recordedBy: West, D; **Taxon:** scientificName: *Digitaria abyssinica* (Hochst. ex A.Rich.) Stapf; kingdom: Plantae; family: Poaceae; genus: Digitaria; specificEpithet: abyssinica; scientificNameAuthorship: (Hochst. ex A.Rich.) Stapf; **Location:** continent: Africa; country: Tanzania; stateProvince: Arusha; county: Ngorongoro; locality: Ngorongoro Crater; verbatimLocality: floor of Ngorongoro crater; decimalLatitude: -3.166667; decimalLongitude: 35.583333; **Event:** eventDate: 1962-05-04; **Record Level:** institutionCode: K; collectionCode: Herbarium; ownerInstitutionCode: K; basisOfRecord: PreservedSpecimen

##### Distribution

Tropical Africa & Arabia

#### Digitaria diagonalis

(Nees) Stapf

Digitaria diagonalis Stapf var. uniglumis (A.Rich) Pilg.

##### Materials

- **Type status:** Other material. **Occurrence:** catalogNumber: K001087216; recordNumber: 10127; recordedBy: Greenway, PJ; **Taxon:** scientificName: Digitaria diagonalis Stapf var. uniglumis (A.Rich) Pilg.; kingdom: Plantae; family: Poaceae; genus: Digitaria; specificEpithet: diagonalis; infraspecificEpithet: uniglumis; scientificNameAuthorship: (A.Rich) Pilg.; **Location:** continent: Africa; country: Tanzania; stateProvince: Mara; county: Serengeti; locality: Tabora river; verbatimLocality: Tabora river, mile 46.6 from the Bologonja river via Klein's camp.; minimumElevationInMeters: 1615; decimalLatitude: -1.816667; decimalLongitude: 34.633333; **Event:** eventDate: 1961-04-30; **Record Level:** institutionCode: K; collectionCode: Herbarium; ownerInstitutionCode: K; basisOfRecord: PreservedSpecimen
- **Type status:** Other material. **Occurrence:** catalogNumber: K001087218; recordNumber: 5634; recordedBy: Newbould, JB; **Taxon:** scientificName: Digitaria diagonalis Stapf var. uniglumis (A.Rich) Pilg.; kingdom: Plantae; family: Poaceae; genus: Digitaria; specificEpithet: diagonalis; infraspecificEpithet: uniglumis; scientificNameAuthorship: (A.Rich) Pilg.; **Location:** continent: Africa; country: Tanzania; stateProvince: Arusha; county: Ngorongoro; locality: Lerong; verbatimLocality: West side of Lemagrut; minimumElevationInMeters: 2286; decimalLatitude: -3.166667; decimalLongitude: 35.35; **Event:** eventDate: 1961-02-02; **Record Level:** institutionCode: K; collectionCode: Herbarium; ownerInstitutionCode: K; basisOfRecord: PreservedSpecimen
- **Type status:** Other material. **Occurrence:** catalogNumber: K001087219; recordNumber: 6498; recordedBy: Newbould, JB; **Taxon:** scientificName: Digitaria diagonalis Stapf var. uniglumis (A.Rich) Pilg.; kingdom: Plantae; family: Poaceae; genus: Digitaria; specificEpithet: diagonalis; infraspecificEpithet: uniglumis; scientificNameAuthorship: (A.Rich) Pilg.; **Location:** continent: Africa; country: Tanzania; stateProvince: Arusha; county: Ngorongoro; locality: Mokilal; verbatimLocality: Southern foothills of Lemagrut.; minimumElevationInMeters: 2439; decimalLatitude: -3.166667; decimalLongitude: 35.35; **Event:** eventDate: 1963-1; **Record Level:** institutionCode: K; collectionCode: Herbarium; ownerInstitutionCode: K; basisOfRecord: PreservedSpecimen

##### Distribution

Tropical Africa & Arabia

#### Digitaria macroblephara

(Hack. ex Schinz) Paoli

##### Materials

- **Type status:** Other material. **Occurrence:** catalogNumber: K000984085; recordNumber: 5595; recordedBy: Leippert, H; **Taxon:** scientificName: *Digitaria macroblephara* (Hack. ex Schinz) Paoli; kingdom: Plantae; family: Poaceae; genus: Digitaria; specificEpithet: macroblephara; scientificNameAuthorship: (Hack. ex Schinz) Paoli; **Location:** continent: Africa; country: Tanzania; stateProvince: Shinyanga; locality: Lake Magadi; verbatimLocality: Serengeti plain entrance of National Park near Lake Magadi; minimumElevationInMeters: 1600; decimalLatitude: -2.75; decimalLongitude: 34.833333; **Event:** eventDate: 1965-03-02; **Record Level:** institutionCode: K; collectionCode: Herbarium; ownerInstitutionCode: K; basisOfRecord: PreservedSpecimen
- **Type status:** Other material. **Occurrence:** catalogNumber: K000984086; recordNumber: 19304; recordedBy: Raynal, J; **Taxon:** scientificName: *Digitaria macroblephara* (Hack. ex Schinz) Paoli; kingdom: Plantae; family: Poaceae; genus: Digitaria; specificEpithet: macroblephara; scientificNameAuthorship: (Hack. ex Schinz) Paoli; **Location:** continent: Africa; country: Tanzania; stateProvince: Arusha; county: Ngorongoro; locality: Olduvai; verbatimLocality: Ngorongoro Conservation Area; minimumElevationInMeters: 1580; decimalLatitude: -2.95; decimalLongitude: 35.166667; **Event:** eventDate: 1977-09-27; **Record Level:** institutionCode: K; collectionCode: Herbarium; ownerInstitutionCode: K; basisOfRecord: PreservedSpecimen
- **Type status:** Other material. **Occurrence:** catalogNumber: K000984087; recordNumber: 362; recordedBy: Paulo, S; **Taxon:** scientificName: *Digitaria macroblephara* (Hack. ex Schinz) Paoli; kingdom: Plantae; family: Poaceae; genus: Digitaria; specificEpithet: macroblephara; scientificNameAuthorship: (Hack. ex Schinz) Paoli; **Location:** continent: Africa; country: Tanzania; stateProvince: Mara; county: Serengeti; locality: Soitayai; verbatimLocality: North of Soityai (Serengeti area); decimalLatitude: -2.466667; decimalLongitude: 35.333333; **Event:** eventDate: 1958-04-23; **Record Level:** institutionCode: K; collectionCode: Herbarium; ownerInstitutionCode: K; basisOfRecord: PreservedSpecimen
- **Type status:** Other material. **Occurrence:** catalogNumber: K000984088; recordNumber: 339; recordedBy: Paulo, S; **Taxon:** scientificName: *Digitaria macroblephara* (Hack. ex Schinz) Paoli; kingdom: Plantae; family: Poaceae; genus: Digitaria; specificEpithet: macroblephara; scientificNameAuthorship: (Hack. ex Schinz) Paoli; **Location:** continent: Africa; country: Tanzania; stateProvince: Mara; county: Serengeti; locality: Serengeti; verbatimLocality: Central Serengeti Plains; decimalLatitude: -2.333333; decimalLongitude: 34.833333; **Event:** eventDate: 1958-04-21; **Record Level:** institutionCode: K; collectionCode: Herbarium; ownerInstitutionCode: K; basisOfRecord: PreservedSpecimen
- **Type status:** Other material. **Occurrence:** catalogNumber: K000984089; recordNumber: 401; recordedBy: Paulo, S; **Taxon:** scientificName: *Digitaria macroblephara* (Hack. ex Schinz) Paoli; kingdom: Plantae; family: Poaceae; genus: Digitaria; specificEpithet: macroblephara; scientificNameAuthorship: (Hack. ex Schinz) Paoli; **Location:** continent: Africa; country: Tanzania; stateProvince: Shinyanga; locality: Lake Magadi; verbatimLocality: Lake Magadi, South Seronera; decimalLatitude: -2.633333; decimalLongitude: 34.9; **Event:** eventDate: 1958-04-28; **Record Level:** institutionCode: K; collectionCode: Herbarium; ownerInstitutionCode: K; basisOfRecord: PreservedSpecimen
- **Type status:** Other material. **Occurrence:** catalogNumber: K000984090; recordNumber: 351; recordedBy: Paulo, S; **Taxon:** scientificName: *Digitaria macroblephara* (Hack. ex Schinz) Paoli; kingdom: Plantae; family: Poaceae; genus: Digitaria; specificEpithet: macroblephara; scientificNameAuthorship: (Hack. ex Schinz) Paoli; **Location:** continent: Africa; country: Tanzania; stateProvince: Arusha; county: Ngorongoro; locality: Lemuta hill; verbatimLocality: South of Lemuta hill; decimalLatitude: -2.7; decimalLongitude: 35.266667; **Event:** eventDate: 1958-04-22; **Record Level:** institutionCode: K; collectionCode: Herbarium; ownerInstitutionCode: K; basisOfRecord: PreservedSpecimen
- **Type status:** Other material. **Occurrence:** catalogNumber: K000984091; recordNumber: 9837; recordedBy: Greenway, PJ; **Taxon:** scientificName: *Digitaria macroblephara* (Hack. ex Schinz) Paoli; kingdom: Plantae; family: Poaceae; genus: Digitaria; specificEpithet: macroblephara; scientificNameAuthorship: (Hack. ex Schinz) Paoli; **Location:** continent: Africa; country: Tanzania; stateProvince: Mara; county: Serengeti; locality: Seronera; verbatimLocality: Serengeti; decimalLatitude: -2.45; decimalLongitude: 34.833333; **Event:** eventDate: 1961-03-17; **Record Level:** institutionCode: K; collectionCode: Herbarium; ownerInstitutionCode: K; basisOfRecord: PreservedSpecimen
- **Type status:** Other material. **Occurrence:** catalogNumber: K000984092; recordNumber: 96; recordedBy: Brooks, GP; **Taxon:** scientificName: *Digitaria macroblephara* (Hack. ex Schinz) Paoli; kingdom: Plantae; family: Poaceae; genus: Digitaria; specificEpithet: macroblephara; scientificNameAuthorship: (Hack. ex Schinz) Paoli; **Location:** continent: Africa; country: Tanzania; stateProvince: Mara; county: Serengeti; locality: Banagi Hill; verbatimLocality: W. Serengeti plains; decimalLatitude: -2.3; decimalLongitude: 34.833333; **Event:** eventDate: 1954-03-16; **Record Level:** institutionCode: K; collectionCode: Herbarium; ownerInstitutionCode: K; basisOfRecord: PreservedSpecimen
- **Type status:** Other material. **Occurrence:** catalogNumber: K000984093; recordNumber: 48; recordedBy: Brooks, GP; **Taxon:** scientificName: *Digitaria macroblephara* (Hack. ex Schinz) Paoli; kingdom: Plantae; family: Poaceae; genus: Digitaria; specificEpithet: macroblephara; scientificNameAuthorship: (Hack. ex Schinz) Paoli; **Location:** continent: Africa; country: Tanzania; stateProvince: Mara; county: Serengeti; locality: Sand hill; verbatimLocality: Central Serengeti National Park; decimalLatitude: -2.833333; decimalLongitude: 35; **Event:** eventDate: 1954-01-24; **Record Level:** institutionCode: K; collectionCode: Herbarium; ownerInstitutionCode: K; basisOfRecord: PreservedSpecimen
- **Type status:** Other material. **Occurrence:** catalogNumber: K000984094; recordNumber: 99; recordedBy: Braun, HMH; **Taxon:** scientificName: *Digitaria macroblephara* (Hack. ex Schinz) Paoli; kingdom: Plantae; family: Poaceae; genus: Digitaria; specificEpithet: macroblephara; scientificNameAuthorship: (Hack. ex Schinz) Paoli; **Location:** continent: Africa; country: Tanzania; stateProvince: Mara; county: Serengeti; locality: Nyaraswiga; verbatimLocality: North West Escarpment of Nyaraswiga plateau; decimalLatitude: -2.366667; decimalLongitude: 34.783333; **Event:** eventDate: 1967-01-22; **Record Level:** institutionCode: K; collectionCode: Herbarium; ownerInstitutionCode: K; basisOfRecord: PreservedSpecimen
- **Type status:** Other material. **Occurrence:** catalogNumber: K000984095; recordNumber: 2107; recordedBy: Hornby; **Taxon:** scientificName: *Digitaria macroblephara* (Hack. ex Schinz) Paoli; kingdom: Plantae; family: Poaceae; genus: Digitaria; specificEpithet: macroblephara; scientificNameAuthorship: (Hack. ex Schinz) Paoli; **Location:** continent: Africa; country: Tanzania; stateProvince: Arusha; county: Ngorongoro; locality: Kakesio River; verbatimLocality: S. Serengeti; decimalLatitude: -3.316667; decimalLongitude: 35.033333; **Event:** eventDate: 1941-02-06; **Record Level:** institutionCode: K; collectionCode: Herbarium; ownerInstitutionCode: K; basisOfRecord: PreservedSpecimen
- **Type status:** Other material. **Occurrence:** catalogNumber: K000984096; recordNumber: 6544; recordedBy: Newbould, JB; **Taxon:** scientificName: *Digitaria macroblephara* (Hack. ex Schinz) Paoli; kingdom: Plantae; family: Poaceae; genus: Digitaria; specificEpithet: macroblephara; scientificNameAuthorship: (Hack. ex Schinz) Paoli; **Location:** continent: Africa; country: Tanzania; stateProvince: Arusha; county: Ngorongoro; locality: Oldiang'arang'ar Ang'ata; decimalLatitude: -2.8; decimalLongitude: 35.416667; **Event:** eventDate: 1963-2; **Record Level:** institutionCode: K; collectionCode: Herbarium; ownerInstitutionCode: K; basisOfRecord: PreservedSpecimen
- **Type status:** Other material. **Occurrence:** catalogNumber: 594; recordNumber: 24257; recordedBy: Peterson, PM; Soreng, RJ; Romaschenko, K; Mbago, F; **Taxon:** scientificName: *Digitaria macroblephara* (Hack. ex Schinz) Paoli; kingdom: Plantae; family: Poaceae; genus: Digitaria; specificEpithet: macroblephara; scientificNameAuthorship: (Hack. ex Schinz) Paoli; **Location:** continent: Africa; country: Tanzania; stateProvince: Shinyanga; locality: Naabi Hill Gate; verbatimLocality: Serengeti National Park, Naabi Hill Gate (at 0.5 km N).; minimumElevationInMeters: 1734; decimalLatitude: -2.83139; decimalLongitude: 34.99672; **Event:** eventDate: 2012-06-16; **Record Level:** institutionCode: US; collectionCode: Herbarium; ownerInstitutionCode: US; basisOfRecord: PreservedSpecimen
- **Type status:** Other material. **Occurrence:** catalogNumber: 1144; recordNumber: 829; recordedBy: Mollel, NP; Rusch, GM; Mwakalebe, G; **Taxon:** scientificName: *Digitaria macroblephara* (Hack. ex Schinz) Paoli; kingdom: Plantae; family: Poaceae; genus: Digitaria; specificEpithet: macroblephara; scientificNameAuthorship: (Hack. ex Schinz) Paoli; **Location:** continent: Africa; country: Tanzania; stateProvince: Mara; county: Serengeti; locality: Robanda village; verbatimLocality: Serengeti district, Robanda village, road to the water pool, CG01 plot. 36M, 0386088, 9763754 UTM.; minimumElevationInMeters: 1388; decimalLatitude: -2.083333; decimalLongitude: 34.666667; **Event:** eventDate: 2003-01-28; **Record Level:** institutionCode: NHT; collectionCode: Herbarium; ownerInstitutionCode: NHT; basisOfRecord: PreservedSpecimen

##### Distribution

Tanzania, Kenya, Uganda, Ethiopia, Somalia & Sudan

#### Digitaria nodosa

Parl.

##### Materials

- **Type status:** Other material. **Occurrence:** catalogNumber: K001087104; recordNumber: 6521; recordedBy: Newbould, JB; **Taxon:** scientificName: *Digitaria nodosa* Parl.; kingdom: Plantae; family: Poaceae; genus: Digitaria; specificEpithet: nodosa; scientificNameAuthorship: Parl.; **Location:** continent: Africa; country: Tanzania; stateProvince: Arusha; county: Ngorongoro; locality: Endoinyo Emboleh; verbatimLocality: Endoinyo Emboleh, Ngorongoro crater; minimumElevationInMeters: 1768; decimalLatitude: -3.083333; decimalLongitude: 35.416667; **Event:** eventDate: 1963-1; **Record Level:** institutionCode: K; collectionCode: Herbarium; ownerInstitutionCode: K; basisOfRecord: PreservedSpecimen

##### Distribution

Tropical Africa, Asia & Arabia

#### Digitaria rivae

(Chiov.) Stapf

##### Materials

- **Type status:** Other material. **Occurrence:** catalogNumber: 595; recordNumber: 24294; recordedBy: Peterson, PM; Soreng, RJ; Romaschenko, K; Mbago, F; **Taxon:** scientificName: *Digitaria rivae* (Chiov.) Stapf; kingdom: Plantae; family: Poaceae; genus: Digitaria; specificEpithet: rivae; scientificNameAuthorship: (Chiov.) Stapf; **Location:** continent: Africa; country: Tanzania; stateProvince: Mara; county: Serengeti; locality: Lobo Lodge; verbatimLocality: Serengeti National Park, on top of Lobo Ridge above Lobo Lodge.; minimumElevationInMeters: 1817; decimalLatitude: -1.99967; decimalLongitude: 35.16778; **Event:** eventDate: 2012-06-17; **Record Level:** institutionCode: US; collectionCode: Herbarium; ownerInstitutionCode: US; basisOfRecord: PreservedSpecimen

##### Distribution

Eastern Africa & Arabia

#### Digitaria ternata

A.Rich.Stapf

##### Materials

- **Type status:** Other material. **Occurrence:** catalogNumber: 596; recordNumber: 367; recordedBy: Ellemann, L; **Taxon:** scientificName: *Digitaria ternata* A.Rich.Stapf; kingdom: Plantae; family: Poaceae; genus: Digitaria; specificEpithet: ternata; scientificNameAuthorship: A.Rich.Stapf; **Location:** continent: Africa; country: Tanzania; stateProvince: Arusha; county: Ngorongoro; locality: Endulen; verbatimLocality: Ngorongoro Conservation Area. Endulen Hospital.; minimumElevationInMeters: 1900; decimalLatitude: -3.2; decimalLongitude: 35.266; **Event:** eventDate: 1993-04-20; **Record Level:** institutionCode: AAU; collectionCode: Herbarium; ownerInstitutionCode: AAU; basisOfRecord: PreservedSpecimen
- **Type status:** Other material. **Occurrence:** catalogNumber: 1141; recordNumber: 87; recordedBy: Frame, GW; **Taxon:** scientificName: *Digitaria ternata* A.Rich.Stapf; kingdom: Plantae; family: Poaceae; genus: Digitaria; specificEpithet: ternata; scientificNameAuthorship: A.Rich.Stapf; **Location:** continent: Africa; country: Tanzania; stateProvince: Arusha; county: Ngorongoro; locality: Empakaai crater; verbatimLocality: Ngorongoro conservation area, Empakaai crater, top of east rim.; minimumElevationInMeters: 2400; decimalLatitude: -2.933333; decimalLongitude: 35.816667; **Event:** eventDate: 1973-03-13; **Record Level:** institutionCode: NHT; collectionCode: Herbarium; ownerInstitutionCode: NHT; basisOfRecord: PreservedSpecimen
- **Type status:** Other material. **Occurrence:** catalogNumber: 1142; recordNumber: 2637; recordedBy: Chuwa, S; **Taxon:** scientificName: *Digitaria ternata* A.Rich.Stapf; kingdom: Plantae; family: Poaceae; genus: Digitaria; specificEpithet: ternata; scientificNameAuthorship: A.Rich.Stapf; **Location:** continent: Africa; country: Tanzania; stateProvince: Arusha; county: Ngorongoro; locality: Endulen; verbatimLocality: Ngorongoro conservation area.; minimumElevationInMeters: 1524; decimalLatitude: -3.183333; decimalLongitude: 35.15; **Event:** eventDate: 1988-04-18; **Record Level:** institutionCode: NHT; collectionCode: Herbarium; ownerInstitutionCode: NHT; basisOfRecord: PreservedSpecimen

##### Distribution

Widespread

#### Digitaria velutina

(Forssk.) P.Beauv.

##### Materials

- **Type status:** Other material. **Occurrence:** catalogNumber: K000984097; recordNumber: 10573; recordedBy: Greenway, PJ; **Taxon:** scientificName: *Digitaria velutina* (Forssk.) P.Beauv.; kingdom: Plantae; family: Poaceae; genus: Digitaria; specificEpithet: velutina; scientificNameAuthorship: (Forssk.) P.Beauv.; **Location:** continent: Africa; country: Tanzania; stateProvince: Mara; county: Serengeti; locality: Seronera; verbatimLocality: Serengeti; decimalLatitude: -2.45; decimalLongitude: 34.833333; **Event:** eventDate: 1962-04-09; **Record Level:** institutionCode: K; collectionCode: Herbarium; ownerInstitutionCode: K; basisOfRecord: PreservedSpecimen
- **Type status:** Other material. **Occurrence:** catalogNumber: K000984098; recordNumber: 9972; recordedBy: Greenway, PJ; **Taxon:** scientificName: *Digitaria velutina* (Forssk.) P.Beauv.; kingdom: Plantae; family: Poaceae; genus: Digitaria; specificEpithet: velutina; scientificNameAuthorship: (Forssk.) P.Beauv.; **Location:** continent: Africa; country: Tanzania; stateProvince: Mara; county: Serengeti; locality: Banagi; verbatimLocality: Banagi to Seronera river crossing down the corridor, mile 14.5 from Seronera.; decimalLatitude: -2.3; decimalLongitude: 34.966667; **Event:** eventDate: 1961-04-04; **Record Level:** institutionCode: K; collectionCode: Herbarium; ownerInstitutionCode: K; basisOfRecord: PreservedSpecimen
- **Type status:** Other material. **Occurrence:** catalogNumber: K000984099; recordNumber: 6409; recordedBy: Newbould, JB; **Taxon:** scientificName: *Digitaria velutina* (Forssk.) P.Beauv.; kingdom: Plantae; family: Poaceae; genus: Digitaria; specificEpithet: velutina; scientificNameAuthorship: (Forssk.) P.Beauv.; **Location:** continent: Africa; country: Tanzania; stateProvince: Arusha; county: Ngorongoro; locality: Olkarien; decimalLatitude: -2.6; decimalLongitude: 35.366667; **Event:** eventDate: 1961-11-20; **Record Level:** institutionCode: K; collectionCode: Herbarium; ownerInstitutionCode: K; basisOfRecord: PreservedSpecimen

##### Distribution

Tropical Africa & Arabia

#### Diheteropogon amplectensvar.amplectens

(Nees) Clayton

##### Materials

- **Type status:** Other material. **Occurrence:** catalogNumber: K000984040; recordNumber: 10770; recordedBy: Greenway, PJ; **Taxon:** scientificName: Diheteropogon amplectens (Nees) Clayton var. amplectens; kingdom: Plantae; family: Poaceae; genus: Diheteropogon; specificEpithet: amplectens; infraspecificEpithet: amplectens; scientificNameAuthorship: (Nees) Clayton; **Location:** continent: Africa; country: Tanzania; stateProvince: Mara; county: Serengeti; locality: Mara River Guard Post; minimumElevationInMeters: 1280; decimalLatitude: -1.566667; decimalLongitude: 34.683333; **Event:** eventDate: 1962-08-09; **Record Level:** institutionCode: K; collectionCode: Herbarium; ownerInstitutionCode: K; basisOfRecord: PreservedSpecimen
- **Type status:** Other material. **Occurrence:** catalogNumber: K000984041; recordNumber: 10905; recordedBy: Greenway, PJ; Tanner, M; **Taxon:** scientificName: Diheteropogon amplectens (Nees) Clayton var. amplectens; kingdom: Plantae; family: Poaceae; genus: Diheteropogon; specificEpithet: amplectens; infraspecificEpithet: amplectens; scientificNameAuthorship: (Nees) Clayton; **Location:** continent: Africa; country: Tanzania; stateProvince: Mara; county: Serengeti; locality: Tutishi river; verbatimLocality: N.W. of Tutishi river near the Lake Magadi track; minimumElevationInMeters: 1402; decimalLatitude: -2.616667; decimalLongitude: 34.766667; **Event:** eventDate: 1962-12-22; **Record Level:** institutionCode: K; collectionCode: Herbarium; ownerInstitutionCode: K; basisOfRecord: PreservedSpecimen
- **Type status:** Other material. **Occurrence:** catalogNumber: K000984042; recordNumber: 9900; recordedBy: Greenway, PJ; **Taxon:** scientificName: Diheteropogon amplectens (Nees) Clayton var. amplectens; kingdom: Plantae; family: Poaceae; genus: Diheteropogon; specificEpithet: amplectens; infraspecificEpithet: amplectens; scientificNameAuthorship: (Nees) Clayton; **Location:** continent: Africa; country: Tanzania; stateProvince: Mara; county: Serengeti; locality: Seronera; verbatimLocality: Seronera to Soitayai, Mile, 7.8; minimumElevationInMeters: 1615; decimalLatitude: -2.45; decimalLongitude: 34.833333; **Event:** eventDate: 1961-03-27; **Record Level:** institutionCode: K; collectionCode: Herbarium; ownerInstitutionCode: K; basisOfRecord: PreservedSpecimen
- **Type status:** Other material. **Occurrence:** catalogNumber: K000984043; recordNumber: 9975; recordedBy: Greenway, PJ; **Taxon:** scientificName: Diheteropogon amplectens (Nees) Clayton var. amplectens; kingdom: Plantae; family: Poaceae; genus: Diheteropogon; specificEpithet: amplectens; infraspecificEpithet: amplectens; scientificNameAuthorship: (Nees) Clayton; **Location:** continent: Africa; country: Tanzania; stateProvince: Mara; county: Serengeti; locality: Banagi; verbatimLocality: The corridor, Banagi to Orangi river, mile 26.8 from Seronera; minimumElevationInMeters: 1371; decimalLatitude: -2.183333; decimalLongitude: 35.1; **Event:** eventDate: 1961-04-04; **Record Level:** institutionCode: K; collectionCode: Herbarium; ownerInstitutionCode: K; basisOfRecord: PreservedSpecimen

##### Distribution

Tropical Africa

#### Dinebra caudata

(K.Schum.) P.M. Peterson & N.Snow

Leptochloa caudata (K.Schum.) N.Snow

##### Materials

- **Type status:** Other material. **Occurrence:** catalogNumber: K000984264; recordNumber: 10152; recordedBy: Greenway, PJ; **Taxon:** scientificName: *Dinebra caudata* (K.Schum.) P.M. Peterson & N.Snow; kingdom: Plantae; family: Poaceae; genus: Dinebra; specificEpithet: caudata; scientificNameAuthorship: (K.Schum.) P.M. Peterson & N.Snow; **Location:** continent: Africa; country: Tanzania; stateProvince: Mara; county: Serengeti; locality: Seronera; verbatimLocality: Serengeti; minimumElevationInMeters: 1463; decimalLatitude: -2.45; decimalLongitude: 34.833333; **Event:** eventDate: 1961-05-09; **Record Level:** institutionCode: K; collectionCode: Herbarium; ownerInstitutionCode: K; basisOfRecord: PreservedSpecimen
- **Type status:** Other material. **Occurrence:** catalogNumber: K000984265; recordNumber: 13345; recordedBy: Greenway, PJ; Kanuri; **Taxon:** scientificName: *Dinebra caudata* (K.Schum.) P.M. Peterson & N.Snow; kingdom: Plantae; family: Poaceae; genus: Dinebra; specificEpithet: caudata; scientificNameAuthorship: (K.Schum.) P.M. Peterson & N.Snow; **Location:** continent: Africa; country: Tanzania; stateProvince: Mara; county: Serengeti; locality: Campi ya Mawi; verbatimLocality: N.E. of Campi ya Mawi and the Togora plains; minimumElevationInMeters: 1768; decimalLatitude: -2.166667; decimalLongitude: 35; **Event:** eventDate: 1968-02-28; **Record Level:** institutionCode: K; collectionCode: Herbarium; ownerInstitutionCode: K; basisOfRecord: PreservedSpecimen
- **Type status:** Other material. **Occurrence:** catalogNumber: K000984266; recordNumber: 10665; recordedBy: Greenway, PJ; **Taxon:** scientificName: *Dinebra caudata* (K.Schum.) P.M. Peterson & N.Snow; kingdom: Plantae; family: Poaceae; genus: Dinebra; specificEpithet: caudata; scientificNameAuthorship: (K.Schum.) P.M. Peterson & N.Snow; **Location:** continent: Africa; country: Tanzania; stateProvince: Arusha; county: Ngorongoro; locality: Klein's camp; minimumElevationInMeters: 1707; decimalLatitude: -1.7; decimalLongitude: 35.216667; **Event:** eventDate: 1962-05-23; **Record Level:** institutionCode: K; collectionCode: Herbarium; ownerInstitutionCode: K; basisOfRecord: PreservedSpecimen

##### Distribution

Tanzania, Kenya, Uganda, Rwanda and Democratic Republic of Congo

#### Dinebra retroflexa

(Vahl) Panz.

##### Materials

- **Type status:** Other material. **Occurrence:** catalogNumber: 1112; recordNumber: 1202; recordedBy: Mollel, NP; Mboya, EI; **Taxon:** scientificName: *Dinebra retroflexa* (Vahl) Panz.; kingdom: Plantae; family: Poaceae; genus: Dinebra; specificEpithet: retroflexa; scientificNameAuthorship: (Vahl) Panz.; **Location:** continent: Africa; country: Tanzania; stateProvince: Arusha; county: Ngorongoro; locality: Laetoli valley; verbatimLocality: Esere-Laetoli road, Laetoli valley on the hip of stones covering the hominid footprints site.; minimumElevationInMeters: 1708; decimalLatitude: -3.226944; decimalLongitude: 35.191111; **Event:** eventDate: 2008-04-10; **Record Level:** institutionCode: NHT; collectionCode: Herbarium; ownerInstitutionCode: NHT; basisOfRecord: PreservedSpecimen
- **Type status:** Other material. **Occurrence:** catalogNumber: 1113; recordNumber: 1202; recordedBy: Mollel, NP; Mboya, EI; **Taxon:** scientificName: *Dinebra retroflexa* (Vahl) Panz.; kingdom: Plantae; family: Poaceae; genus: Dinebra; specificEpithet: retroflexa; scientificNameAuthorship: (Vahl) Panz.; **Location:** continent: Africa; country: Tanzania; stateProvince: Arusha; county: Ngorongoro; locality: Laetoli valley; verbatimLocality: Esere-Laetoli road, Laetoli valley on the hip of stones covering the hominid footprints site.; minimumElevationInMeters: 1708; decimalLatitude: -3.226944; decimalLongitude: 35.191111; **Event:** eventDate: 2008-04-10; **Record Level:** institutionCode: NCAA; collectionCode: Herbarium; ownerInstitutionCode: NCAA; basisOfRecord: PreservedSpecimen

##### Distribution

Widespread

#### Dinebra retroflexavar.condensata

S.M.Philips

##### Materials

- **Type status:** Other material. **Occurrence:** catalogNumber: 872; recordNumber: 13168; recordedBy: Greenway, PJ; Kanuri; Braun; **Taxon:** scientificName: Dinebra retroflexa (Vahl) Panz. var. condensata S.M.Philips; kingdom: Plantae; family: Poaceae; genus: Dinebra; specificEpithet: retroflexa; infraspecificEpithet: condensata; scientificNameAuthorship: S.M.Philips; **Location:** continent: Africa; country: Tanzania; stateProvince: Mara; county: Serengeti; locality: Musabi; verbatimLocality: East of Musabi, Grumeti river.; decimalLatitude: -2.233333; decimalLongitude: 34.466667; **Event:** eventDate: 1968-02-10; **Record Level:** collectionCode: Herbarium; ownerInstitutionCode: ?; basisOfRecord: PreservedSpecimen
- **Type status:** Other material. **Occurrence:** catalogNumber: K001087167; recordNumber: 10613; recordedBy: Greenway, PJ; Watson, M; **Taxon:** scientificName: Dinebra retroflexa (Vahl) Panz. var. condensata S.M.Philips; kingdom: Plantae; family: Poaceae; genus: Dinebra; specificEpithet: retroflexa; infraspecificEpithet: condensata; scientificNameAuthorship: S.M.Philips; **Location:** continent: Africa; country: Tanzania; stateProvince: Mara; county: Serengeti; locality: Nyakoromo Guard Post; verbatimLocality: Nyakaoromo guard post to Ndabaka.; minimumElevationInMeters: 1097; decimalLatitude: -2.2; decimalLongitude: 34.05; **Event:** eventDate: 1962-04-19; **Record Level:** institutionCode: K; collectionCode: Herbarium; ownerInstitutionCode: K; basisOfRecord: PreservedSpecimen

##### Distribution

Tropical Africa & Asia

#### Diplachne fusca

(L.) P.Beauv. ex Roem. & Schult.

Leptochloa fusca (L.) Kunth | Leptochloa fusca (L.) Kunth subsp. fuscafusca

##### Materials

- **Type status:** Other material. **Occurrence:** catalogNumber: K001087168; recordNumber: 12593; recordedBy: Greenway, PJ; Kanuri; **Taxon:** scientificName: *Diplachne fusca* (L.) P.Beauv. ex Roem. & Schult.; kingdom: Plantae; family: Poaceae; genus: Diplachne; specificEpithet: fusca; scientificNameAuthorship: (L.) P.Beauv. ex Roem. & Schult.; **Location:** continent: Africa; country: Tanzania; stateProvince: Arusha; county: Ngorongoro; locality: Ngorongoro Crater; verbatimLocality: SE side of Ngorongoro crater floor. Hippo pool.; decimalLatitude: -3.25; decimalLongitude: 35.583333; **Event:** eventDate: 1966-07-21; **Record Level:** institutionCode: K; collectionCode: Herbarium; ownerInstitutionCode: K; basisOfRecord: PreservedSpecimen
- **Type status:** Other material. **Occurrence:** catalogNumber: K001087170; recordNumber: 19521; recordedBy: Raynal, J; **Taxon:** scientificName: *Diplachne fusca* (L.) P.Beauv. ex Roem. & Schult.; kingdom: Plantae; family: Poaceae; genus: Diplachne; specificEpithet: fusca; scientificNameAuthorship: (L.) P.Beauv. ex Roem. & Schult.; **Location:** continent: Africa; country: Tanzania; stateProvince: Arusha; county: Ngorongoro; locality: Ngorongoro Crater; verbatimLocality: Ngorongoro crater floor, Gorigor swamp, western end.; decimalLatitude: -3.166667; decimalLongitude: 35.583333; **Event:** eventDate: 1977-10-09; **Record Level:** institutionCode: K; collectionCode: Herbarium; ownerInstitutionCode: K; basisOfRecord: PreservedSpecimen
- **Type status:** Other material. **Occurrence:** catalogNumber: K001087171; recordNumber: 10043; recordedBy: Greenway, PJ; Turner, M; **Taxon:** scientificName: *Diplachne fusca* (L.) P.Beauv. ex Roem. & Schult.; kingdom: Plantae; family: Poaceae; genus: Diplachne; specificEpithet: fusca; scientificNameAuthorship: (L.) P.Beauv. ex Roem. & Schult.; **Location:** continent: Africa; country: Tanzania; stateProvince: Shinyanga; locality: Lake Magadi; verbatimLocality: Titushi river near Lake Magadi, Musoma district.; minimumElevationInMeters: 1432; decimalLatitude: -2.75; decimalLongitude: 34.833333; **Event:** eventDate: 1961-04-13; **Record Level:** institutionCode: K; collectionCode: Herbarium; ownerInstitutionCode: K; basisOfRecord: PreservedSpecimen
- **Type status:** Other material. **Occurrence:** catalogNumber: K000984261; recordNumber: 12535; recordedBy: Greenway, PJ; Kanuri; **Taxon:** scientificName: *Leptochloa fusca* (L.) Kunth; kingdom: Plantae; family: Poaceae; genus: Leptochloa; specificEpithet: fusca; scientificNameAuthorship: (L.) Kunth; **Location:** continent: Africa; country: Tanzania; stateProvince: Shinyanga; locality: Lake Magadi; verbatimLocality: Ngorongoro Crater; minimumElevationInMeters: 1646; decimalLatitude: -3.183333; decimalLongitude: 35.533333; **Event:** eventDate: 1966-07-05; **Record Level:** institutionCode: K; collectionCode: Herbarium; ownerInstitutionCode: K; basisOfRecord: PreservedSpecimen
- **Type status:** Other material. **Occurrence:** catalogNumber: K000984262; recordNumber: 9027; recordedBy: Greenway, PJ; **Taxon:** scientificName: *Leptochloa fusca* (L.) Kunth; kingdom: Plantae; family: Poaceae; genus: Leptochloa; specificEpithet: fusca; scientificNameAuthorship: (L.) Kunth; **Location:** continent: Africa; country: Tanzania; stateProvince: Shinyanga; locality: Mbalageti river; verbatimLocality: N.W. of Serengeti; decimalLatitude: -2.883333; decimalLongitude: 34.9; **Event:** eventDate: 1956-12-16; **Record Level:** institutionCode: K; collectionCode: Herbarium; ownerInstitutionCode: K; basisOfRecord: PreservedSpecimen
- **Type status:** Other material. **Occurrence:** catalogNumber: K000984263; recordNumber: 10501; recordedBy: Greenway, PJ; **Taxon:** scientificName: *Leptochloa fusca* (L.) Kunth; kingdom: Plantae; family: Poaceae; genus: Leptochloa; specificEpithet: fusca; scientificNameAuthorship: (L.) Kunth; **Location:** continent: Africa; country: Tanzania; stateProvince: Mara; county: Serengeti; locality: Titushi river flats; verbatimLocality: mile 14 from Seronera, Serengeti; minimumElevationInMeters: 800; decimalLatitude: -2.616667; decimalLongitude: 34.766667; **Event:** eventDate: 1962-03-06; **Record Level:** institutionCode: K; collectionCode: Herbarium; ownerInstitutionCode: K; basisOfRecord: PreservedSpecimen
- **Type status:** Other material. **Occurrence:** catalogNumber: 641; recordNumber: 24322; recordedBy: Peterson, PM; Soreng, RJ; Romaschenko, K; Mbago, F; **Taxon:** scientificName: *Leptochloa fusca* (L.) Kunth; kingdom: Plantae; family: Poaceae; genus: Leptochloa; specificEpithet: fusca; scientificNameAuthorship: (L.) Kunth; **Location:** continent: Africa; country: Tanzania; stateProvince: Arusha; county: Ngorongoro; locality: Lake Magadi; verbatimLocality: Ngorongoro Conservation Area, W side of Lake Magadi.; minimumElevationInMeters: 1739; decimalLatitude: -3.17713; decimalLongitude: 35.51548; **Event:** eventDate: 2012-06-19; **Record Level:** institutionCode: US; collectionCode: Herbarium; ownerInstitutionCode: US; basisOfRecord: PreservedSpecimen
- **Type status:** Other material. **Occurrence:** catalogNumber: K001087169; recordNumber: 13183; recordedBy: Greenway, PJ; Kanuri; Boer de; Braun, H; **Taxon:** scientificName: Leptochloa fusca (L.) Kunth subsp. fusca; kingdom: Plantae; family: Poaceae; genus: Leptochloa; specificEpithet: fusca; infraspecificEpithet: fusca; scientificNameAuthorship: (L.) Kunth; **Location:** continent: Africa; country: Tanzania; stateProvince: Mara; county: Serengeti; locality: Serengeti Plains; verbatimLocality: Serengeti plains, head waters of the Mbalangeti river.; minimumElevationInMeters: 1554; decimalLatitude: -2.333333; decimalLongitude: 34.833333; **Event:** eventDate: 1968-02-14; **Record Level:** institutionCode: K; collectionCode: Herbarium; ownerInstitutionCode: K; basisOfRecord: PreservedSpecimen

##### Distribution

Widespread

#### Disakisperma obtusiflorum

(Hochst.) P.M. Peterson & N.Snow

Leptochloa obtusiflora Hochst.

##### Materials

- **Type status:** Other material. **Occurrence:** catalogNumber: K000984259; recordNumber: 9971; recordedBy: Greenway, PJ; **Taxon:** scientificName: *Leptochloa obtusiflora* Hochst.; kingdom: Plantae; family: Poaceae; genus: Leptochloa; specificEpithet: obtusiflora; scientificNameAuthorship: Hochst.; **Location:** continent: Africa; country: Tanzania; stateProvince: Mara; county: Serengeti; locality: Banagi; verbatimLocality: Banagi to Seronera river crossing the corridor, mile 14.5 from Seronera; minimumElevationInMeters: 1371; decimalLatitude: -2.3; decimalLongitude: 34.966667; **Event:** eventDate: 1961-04-04; **Record Level:** institutionCode: K; collectionCode: Herbarium; ownerInstitutionCode: K; basisOfRecord: PreservedSpecimen
- **Type status:** Other material. **Occurrence:** catalogNumber: K000984260; recordNumber: 10666; recordedBy: Greenway, PJ; **Taxon:** scientificName: *Leptochloa obtusiflora* Hochst.; kingdom: Plantae; family: Poaceae; genus: Leptochloa; specificEpithet: obtusiflora; scientificNameAuthorship: Hochst.; **Location:** continent: Africa; country: Tanzania; stateProvince: Arusha; county: Ngorongoro; locality: Klein's camp; verbatimLocality: Kleins Camp to Bolgonja river; minimumElevationInMeters: 1646; decimalLatitude: -1.7; decimalLongitude: 35.216667; **Event:** eventDate: 1962-05-25; **Record Level:** institutionCode: K; collectionCode: Herbarium; ownerInstitutionCode: K; basisOfRecord: PreservedSpecimen

##### Distribution

Tropical Africa & Arabia

#### Disakisperma yemenicum

(Schweinf.) P.M.Peterson & N.Snow

Coelachyrum yemenicum (Schweinf.) S.M.Phillips; Cypholepis yemenica (Schweinf.) Chiov.

##### Materials

- **Type status:** Other material. **Occurrence:** catalogNumber: 577; recordNumber: 24254; recordedBy: Peterson, PM; Soreng, RJ; Romaschenko, K; Mbago, F; **Taxon:** scientificName: *Coelachyrum yemenicum* (Schweinf.) S.M.Phillips; kingdom: Plantae; family: Poaceae; genus: Coelachyrum; specificEpithet: yemenicum; scientificNameAuthorship: (Schweinf.) S.M.Phillips; **Location:** continent: Africa; country: Tanzania; stateProvince: Shinyanga; locality: Naabi Hill Gate; verbatimLocality: Serengeti National Park, Naabi Hill Gate (at 0.5 km N).; minimumElevationInMeters: 1734; decimalLatitude: -2.83139; decimalLongitude: 34.99672; **Event:** eventDate: 2012-06-16; **Record Level:** institutionCode: US; collectionCode: Herbarium; ownerInstitutionCode: US; basisOfRecord: PreservedSpecimen
- **Type status:** Other material. **Occurrence:** catalogNumber: K001087097; recordNumber: 10694; recordedBy: Greenway, PJ; Turner; **Taxon:** scientificName: *Cypholepis yemenica* (Schweinf.) Chiov.; kingdom: Plantae; family: Poaceae; genus: Cypholepis; specificEpithet: yemenica; scientificNameAuthorship: (Schweinf.) Chiov.; **Location:** continent: Africa; country: Tanzania; stateProvince: Mara; county: Serengeti; locality: Engare Nanyuki springs; verbatimLocality: Ngare Nanyuki springs, Serengeti Central Plains; minimumElevationInMeters: 1615; decimalLatitude: -2.616667; decimalLongitude: 35.216667; **Event:** eventDate: 1962-06-04; **Record Level:** institutionCode: K; collectionCode: Herbarium; ownerInstitutionCode: K; basisOfRecord: PreservedSpecimen
- **Type status:** Other material. **Occurrence:** catalogNumber: K001087098; recordNumber: 6543; recordedBy: Newbould, JB; **Taxon:** scientificName: *Cypholepis yemenica* (Schweinf.) Chiov.; kingdom: Plantae; family: Poaceae; genus: Cypholepis; specificEpithet: yemenica; scientificNameAuthorship: (Schweinf.) Chiov.; **Location:** continent: Africa; country: Tanzania; stateProvince: Arusha; county: Ngorongoro; locality: Oldiang'arangar; verbatimLocality: East Serengeti; minimumElevationInMeters: 1707; decimalLatitude: -2.8; decimalLongitude: 35.416667; **Event:** eventDate: 1963-2; **Record Level:** institutionCode: K; collectionCode: Herbarium; ownerInstitutionCode: K; basisOfRecord: PreservedSpecimen
- **Type status:** Other material. **Occurrence:** catalogNumber: K001087099; recordNumber: 25551; recordedBy: Richards, M; **Taxon:** scientificName: *Cypholepis yemenica* (Schweinf.) Chiov.; kingdom: Plantae; family: Poaceae; genus: Cypholepis; specificEpithet: yemenica; scientificNameAuthorship: (Schweinf.) Chiov.; **Location:** continent: Africa; country: Tanzania; stateProvince: Arusha; county: Ngorongoro; locality: Engaruka; verbatimLocality: Masai district, gorge below the Engaruka road. Top of the escarpment above the gorge.; minimumElevationInMeters: 974; decimalLatitude: -2.983333; decimalLongitude: 35.616667; **Event:** eventDate: 1970-02-27; **Record Level:** institutionCode: K; collectionCode: Herbarium; ownerInstitutionCode: K; basisOfRecord: PreservedSpecimen
- **Type status:** Other material. **Occurrence:** catalogNumber: K001087100; recordNumber: 6362; recordedBy: Leippert; **Taxon:** scientificName: *Cypholepis yemenica* (Schweinf.) Chiov.; kingdom: Plantae; family: Poaceae; genus: Cypholepis; specificEpithet: yemenica; scientificNameAuthorship: (Schweinf.) Chiov.; **Location:** continent: Africa; country: Tanzania; stateProvince: Arusha; county: Ngorongoro; locality: Engaruka; minimumElevationInMeters: 1000; decimalLatitude: -2.983333; decimalLongitude: 35.95; **Event:** eventDate: 1966-02-24; **Record Level:** institutionCode: K; collectionCode: Herbarium; ownerInstitutionCode: K; basisOfRecord: PreservedSpecimen
- **Type status:** Other material. **Occurrence:** catalogNumber: K001087101; recordNumber: 2608; recordedBy: Chuwa, S; **Taxon:** scientificName: *Cypholepis yemenica* (Schweinf.) Chiov.; kingdom: Plantae; family: Poaceae; genus: Cypholepis; specificEpithet: yemenica; scientificNameAuthorship: (Schweinf.) Chiov.; **Location:** continent: Africa; country: Tanzania; stateProvince: Arusha; county: Ngorongoro; locality: Olduvai Gorge; minimumElevationInMeters: 1460; decimalLatitude: -2.95; decimalLongitude: 35.166667; **Event:** eventDate: 1987-05-01; **Record Level:** institutionCode: K; collectionCode: Herbarium; ownerInstitutionCode: K; basisOfRecord: PreservedSpecimen
- **Type status:** Other material. **Occurrence:** catalogNumber: K001087102; recordNumber: 2857; recordedBy: Chuwa, S; **Taxon:** scientificName: *Cypholepis yemenica* (Schweinf.) Chiov.; kingdom: Plantae; family: Poaceae; genus: Cypholepis; specificEpithet: yemenica; scientificNameAuthorship: (Schweinf.) Chiov.; **Location:** continent: Africa; country: Tanzania; stateProvince: Arusha; county: Ngorongoro; locality: Olduvai Gorge; minimumElevationInMeters: 1400; decimalLatitude: -2.95; decimalLongitude: 35.166667; **Event:** eventDate: 1989-12-27; **Record Level:** institutionCode: K; collectionCode: Herbarium; ownerInstitutionCode: K; basisOfRecord: PreservedSpecimen
- **Type status:** Other material. **Occurrence:** catalogNumber: K001087103; recordNumber: 10678; recordedBy: Greenway, PJ; Watson, M; **Taxon:** scientificName: *Cypholepis yemenica* (Schweinf.) Chiov.; kingdom: Plantae; family: Poaceae; genus: Cypholepis; specificEpithet: yemenica; scientificNameAuthorship: (Schweinf.) Chiov.; **Location:** continent: Africa; country: Tanzania; stateProvince: Mara; county: Serengeti; locality: Serengeti; verbatimLocality: Serengeti central plains, 2 miles west of the eastern boundary.; minimumElevationInMeters: 1737; decimalLatitude: -2.333333; decimalLongitude: 34.833333; **Event:** eventDate: 1962-05-30; **Record Level:** institutionCode: K; collectionCode: Herbarium; ownerInstitutionCode: K; basisOfRecord: PreservedSpecimen
- **Type status:** Other material. **Occurrence:** catalogNumber: 1111; recordNumber: 2; recordedBy: Banyikwa, FF; **Taxon:** scientificName: *Cypholepis yemenica* (Schweinf.) Chiov.; kingdom: Plantae; family: Poaceae; genus: Cypholepis; specificEpithet: yemenica; scientificNameAuthorship: (Schweinf.) Chiov.; **Location:** continent: Africa; country: Tanzania; stateProvince: Mara; county: Serengeti; locality: Serengeti; minimumElevationInMeters: -9999; decimalLatitude: -2.333333; decimalLongitude: 34.833333; **Event:** eventDate: 1973-12-06; **Record Level:** institutionCode: NHT; collectionCode: Herbarium; ownerInstitutionCode: NHT; basisOfRecord: PreservedSpecimen

##### Distribution

Arabia, Eastern & Southern Africa

#### Echinochloa brevipedicellata

(Peter) Clayton

##### Materials

- **Type status:** Other material. **Occurrence:** catalogNumber: 291; recordNumber: 3B; recordedBy: Banyikwa, FF; **Taxon:** scientificName: *Echinochloa brevipedicellata* (Peter) Clayton; kingdom: Plantae; family: Poaceae; genus: Echinochloa; specificEpithet: brevipedicellata; scientificNameAuthorship: (Peter) Clayton; **Location:** continent: Africa; country: Tanzania; stateProvince: Mara; county: Serengeti; locality: Serengeti National Park; decimalLatitude: -2.333333; decimalLongitude: 34.833333; **Event:** eventDate: 1905-05-28; **Record Level:** institutionCode: EA; collectionCode: Herbarium; ownerInstitutionCode: EA; basisOfRecord: PreservedSpecimen

##### Distribution

Tanzania & Kenya

#### Echinochloa colona

(L.) Link

##### Materials

- **Type status:** Other material. **Occurrence:** catalogNumber: 602; recordNumber: s.n.; recordedBy: Mboya, E; **Taxon:** scientificName: *Echinochloa colona* (L.) Link; kingdom: Plantae; family: Poaceae; genus: Echinochloa; specificEpithet: colona; scientificNameAuthorship: (L.) Link; **Location:** continent: Africa; country: Tanzania; stateProvince: Mara; county: Serengeti; locality: Seronera Lodge; verbatimLocality: T1. Area around Frankfurt Zoological Society Headquarters and Seronera Lodge.; minimumElevationInMeters: 1512; decimalLatitude: -2.44; decimalLongitude: 34.82; **Event:** eventDate: 2004-02-09; **Record Level:** institutionCode: MO; collectionCode: Herbarium; ownerInstitutionCode: MO; basisOfRecord: PreservedSpecimen
- **Type status:** Other material. **Occurrence:** catalogNumber: 603; recordNumber: s.n.; recordedBy: Mboya, E; **Taxon:** scientificName: *Echinochloa colona* (L.) Link; kingdom: Plantae; family: Poaceae; genus: Echinochloa; specificEpithet: colona; scientificNameAuthorship: (L.) Link; **Location:** continent: Africa; country: Tanzania; stateProvince: Mara; county: Serengeti; locality: Serengeti National Park; verbatimLocality: T1. Serengeti National Park. Ndabaka-Seronera Road.; minimumElevationInMeters: 1297; decimalLatitude: -2.27; decimalLongitude: 34.58; **Event:** eventDate: 2004-03-09; **Record Level:** institutionCode: MO; collectionCode: Herbarium; ownerInstitutionCode: MO; basisOfRecord: PreservedSpecimen
- **Type status:** Other material. **Occurrence:** catalogNumber: 604; recordNumber: s.n.; recordedBy: Mboya, E; **Taxon:** scientificName: *Echinochloa colona* (L.) Link; kingdom: Plantae; family: Poaceae; genus: Echinochloa; specificEpithet: colona; scientificNameAuthorship: (L.) Link; **Location:** continent: Africa; country: Tanzania; stateProvince: Mara; county: Serengeti; locality: Serengeti Research Centre; verbatimLocality: T1. Serengeti Research Centre.; minimumElevationInMeters: 1548; decimalLatitude: -2.38; decimalLongitude: 34.85; **Event:** eventDate: 2004-03-12; **Record Level:** institutionCode: MO; collectionCode: Herbarium; ownerInstitutionCode: MO; basisOfRecord: PreservedSpecimen

##### Distribution

Widespread

#### Echinochloa haploclada

(Stapf) Stapf

##### Materials

- **Type status:** Other material. **Occurrence:** catalogNumber: K001087221; recordNumber: 5605; recordedBy: Leippert; **Taxon:** scientificName: *Echinochloa haploclada* (Stapf) Stapf; kingdom: Plantae; family: Poaceae; genus: Echinochloa; specificEpithet: haploclada; scientificNameAuthorship: (Stapf) Stapf; **Location:** continent: Africa; country: Tanzania; stateProvince: Mara; county: Serengeti; locality: Banagi; verbatimLocality: Riverside, Serengeti National Park, Banagi.; minimumElevationInMeters: 1350; decimalLatitude: -2.3; decimalLongitude: 34.833333; **Event:** eventDate: 1965-03-03; **Record Level:** institutionCode: K; collectionCode: Herbarium; ownerInstitutionCode: K; basisOfRecord: PreservedSpecimen
- **Type status:** Other material. **Occurrence:** catalogNumber: K001087222; recordNumber: 9839; recordedBy: Greenway, PJ; **Taxon:** scientificName: *Echinochloa haploclada* (Stapf) Stapf; kingdom: Plantae; family: Poaceae; genus: Echinochloa; specificEpithet: haploclada; scientificNameAuthorship: (Stapf) Stapf; **Location:** continent: Africa; country: Tanzania; stateProvince: Mara; county: Serengeti; locality: Seronera; minimumElevationInMeters: 1554; decimalLatitude: -2.433333; decimalLongitude: 34.816667; **Event:** eventDate: 1961-03-17; **Record Level:** institutionCode: K; collectionCode: Herbarium; ownerInstitutionCode: K; basisOfRecord: PreservedSpecimen
- **Type status:** Other material. **Occurrence:** catalogNumber: K001087223; recordNumber: 5391; recordedBy: Vesey-FitzGerald, LDEF; **Taxon:** scientificName: *Echinochloa haploclada* (Stapf) Stapf; kingdom: Plantae; family: Poaceae; genus: Echinochloa; specificEpithet: haploclada; scientificNameAuthorship: (Stapf) Stapf; **Location:** continent: Africa; country: Tanzania; stateProvince: Mara; county: Serengeti; locality: Lobo springs; verbatimLocality: Serengeti national park.; minimumElevationInMeters: -9999; decimalLatitude: -2.033333; decimalLongitude: 35.216667; **Event:** eventDate: 1967-09-01; **Record Level:** institutionCode: K; collectionCode: Herbarium; ownerInstitutionCode: K; basisOfRecord: PreservedSpecimen

##### Distribution

Tropical Africa

#### Echinochloa ugandensis

Snowden & C.E.Hubb.

##### Materials

- **Type status:** Other material. **Occurrence:** catalogNumber: 934; recordNumber: 9860; recordedBy: Greenway, PJ; **Taxon:** scientificName: *Echinochloa ugandensis* Snowden & C.E.Hubb.; kingdom: Plantae; family: Poaceae; genus: Echinochloa; specificEpithet: ugandensis; scientificNameAuthorship: Snowden & C.E.Hubb.; **Location:** continent: Africa; country: Tanzania; stateProvince: Mara; county: Serengeti; locality: Seronera; decimalLatitude: -2.45; decimalLongitude: 34.833333; **Event:** eventDate: 1961-03-20; **Record Level:** collectionCode: Herbarium; ownerInstitutionCode: ?; basisOfRecord: PreservedSpecimen
- **Type status:** Other material. **Occurrence:** catalogNumber: 935; recordNumber: 12616; recordedBy: Greenway, PJ; Kanuri; **Taxon:** scientificName: *Echinochloa ugandensis* Snowden & C.E.Hubb.; kingdom: Plantae; family: Poaceae; genus: Echinochloa; specificEpithet: ugandensis; scientificNameAuthorship: Snowden & C.E.Hubb.; **Location:** continent: Africa; country: Tanzania; stateProvince: Arusha; county: Ngorongoro; locality: Ngorongoro Crater; decimalLatitude: -3.166667; decimalLongitude: 35.583333; **Event:** eventDate: 1966-07-25; **Record Level:** collectionCode: Herbarium; ownerInstitutionCode: ?; basisOfRecord: PreservedSpecimen

##### Distribution

Eastern & Southern Africa

#### Ehrharta erecta

Lam.

Ehrharta erecta Lam. var. abyssinica (Hochst.) Pilg.

##### Materials

- **Type status:** Other material. **Occurrence:** catalogNumber: K000984284; recordNumber: 186; recordedBy: Frame, GW; **Taxon:** scientificName: Ehrharta erecta Lam. var. abyssinica (Hochst.) Pilg.; kingdom: Plantae; family: Poaceae; genus: Ehrharta; specificEpithet: erecta; infraspecificEpithet: abyssinica; scientificNameAuthorship: (Hochst.) Pilg.; **Location:** continent: Africa; country: Tanzania; stateProvince: Arusha; county: Ngorongoro; locality: Empakai Crater; verbatimLocality: valley north-west of Jaeger Summit. Ngorongoro Conservation Area.; minimumElevationInMeters: 3047; decimalLatitude: -2.933333; decimalLongitude: 35.816667; **Event:** eventDate: 1973-06-23; **Record Level:** institutionCode: K; collectionCode: Herbarium; ownerInstitutionCode: K; basisOfRecord: PreservedSpecimen
- **Type status:** Other material. **Occurrence:** catalogNumber: K000984285; recordNumber: 6248; recordedBy: Newbould, JB; **Taxon:** scientificName: Ehrharta erecta Lam. var. abyssinica (Hochst.) Pilg.; kingdom: Plantae; family: Poaceae; genus: Ehrharta; specificEpithet: erecta; infraspecificEpithet: abyssinica; scientificNameAuthorship: (Hochst.) Pilg.; **Location:** continent: Africa; country: Tanzania; stateProvince: Arusha; county: Ngorongoro; locality: Nainokanoka; verbatimLocality: Crater Highlands; minimumElevationInMeters: 2591; decimalLatitude: -3.016667; decimalLongitude: 35.683333; **Event:** eventDate: 1962-07-30; **Record Level:** institutionCode: K; collectionCode: Herbarium; ownerInstitutionCode: K; basisOfRecord: PreservedSpecimen
- **Type status:** Other material. **Occurrence:** catalogNumber: 1143; recordNumber: EN803; recordedBy: Njau, E; Leliyo, G; Andrew, P; Bamford, M; **Taxon:** scientificName: Ehrharta erecta Lam. var. abyssinica (Hochst.) Pilg.; kingdom: Plantae; family: Poaceae; genus: Ehrharta; specificEpithet: erecta; infraspecificEpithet: abyssinica; scientificNameAuthorship: (Hochst.) Pilg.; **Location:** continent: Africa; country: Tanzania; stateProvince: Arusha; county: Ngorongoro; locality: Lemigrut; verbatimLocality: Ngorongoro conservation area, Lemigrut, south-east slope.; minimumElevationInMeters: 2625; decimalLatitude: -3.186667; decimalLongitude: 35.378889; **Event:** eventDate: 2004-07-04; **Record Level:** institutionCode: NHT; collectionCode: Herbarium; ownerInstitutionCode: NHT; basisOfRecord: PreservedSpecimen

##### Distribution

Tropical Africa & Arabia

#### Eleusine jaegeri

Pilg.

##### Materials

- **Type status:** Other material. **Occurrence:** catalogNumber: 605; recordNumber: 664; recordedBy: Ellemann, L; **Taxon:** scientificName: *Eleusine jaegeri* Pilg.; kingdom: Plantae; family: Poaceae; genus: Eleusine; specificEpithet: jaegeri; scientificNameAuthorship: Pilg.; **Location:** continent: Africa; country: Tanzania; stateProvince: Arusha; county: Ngorongoro; locality: Oloronyo; verbatimLocality: Ngorongoro Conservation Area, Oloronyo,; minimumElevationInMeters: 2800; decimalLatitude: -3; decimalLongitude: 35.35; **Event:** eventDate: 1993-06-17; **Record Level:** institutionCode: AAU; collectionCode: Herbarium; ownerInstitutionCode: AAU; basisOfRecord: PreservedSpecimen
- **Type status:** Other material. **Occurrence:** catalogNumber: 606; recordNumber: 718; recordedBy: Ellemann, L; **Taxon:** scientificName: *Eleusine jaegeri* Pilg.; kingdom: Plantae; family: Poaceae; genus: Eleusine; specificEpithet: jaegeri; scientificNameAuthorship: Pilg.; **Location:** continent: Africa; country: Tanzania; stateProvince: Arusha; county: Ngorongoro; locality: Sendui; verbatimLocality: Ngorongoro Conservation Area, Sendui, close to ole Senguyans boma.; minimumElevationInMeters: 2650; decimalLatitude: -2.916; decimalLongitude: 35.716; **Event:** eventDate: 1993-07-22; **Record Level:** institutionCode: AAU; collectionCode: Herbarium; ownerInstitutionCode: AAU; basisOfRecord: PreservedSpecimen
- **Type status:** Other material. **Occurrence:** catalogNumber: 607; recordNumber: 861; recordedBy: Ellemann, L; **Taxon:** scientificName: *Eleusine jaegeri* Pilg.; kingdom: Plantae; family: Poaceae; genus: Eleusine; specificEpithet: jaegeri; scientificNameAuthorship: Pilg.; **Location:** continent: Africa; country: Tanzania; stateProvince: Arusha; county: Ngorongoro; locality: Rhino lodge; verbatimLocality: Ngorongoro Conservation Area, Entim e Rotian, close to Rhino-lodge.; minimumElevationInMeters: 2200; decimalLatitude: -3.25; decimalLongitude: 35.516; **Event:** eventDate: 1993-08-29; **Record Level:** institutionCode: AAU; collectionCode: Herbarium; ownerInstitutionCode: AAU; basisOfRecord: PreservedSpecimen
- **Type status:** Other material. **Occurrence:** catalogNumber: 608; recordNumber: 909; recordedBy: Ellemann, L; **Taxon:** scientificName: *Eleusine jaegeri* Pilg.; kingdom: Plantae; family: Poaceae; genus: Eleusine; specificEpithet: jaegeri; scientificNameAuthorship: Pilg.; **Location:** continent: Africa; country: Tanzania; stateProvince: Arusha; county: Ngorongoro; locality: Armakutian; verbatimLocality: Ngorongoro Conservation Area, Armakutian, above Alchorai and the upper water tank in Loomyoni.; minimumElevationInMeters: 2000; decimalLatitude: -3.2; decimalLongitude: 35.483; **Event:** eventDate: 1993-09-15; **Record Level:** institutionCode: AAU; collectionCode: Herbarium; ownerInstitutionCode: AAU; basisOfRecord: PreservedSpecimen
- **Type status:** Other material. **Occurrence:** catalogNumber: 609; recordNumber: 24299; recordedBy: Peterson, PM; Soreng, RJ; Romaschenko, K; Mbago, F; **Taxon:** scientificName: *Eleusine jaegeri* Pilg.; kingdom: Plantae; family: Poaceae; genus: Eleusine; specificEpithet: jaegeri; scientificNameAuthorship: Pilg.; **Location:** continent: Africa; country: Tanzania; stateProvince: Arusha; county: Ngorongoro; locality: Ngorongoro Crater; verbatimLocality: Ngorongoro Conservation Area Headquarters, upper edge of crater.; minimumElevationInMeters: 2405; decimalLatitude: -3.24529; decimalLongitude: 35.48797; **Event:** eventDate: 2012-06-18; **Record Level:** institutionCode: US; collectionCode: Herbarium; ownerInstitutionCode: US; basisOfRecord: PreservedSpecimen
- **Type status:** Other material. **Occurrence:** catalogNumber: 796; recordNumber: s.n.; recordedBy: Napper, D; **Taxon:** scientificName: *Eleusine jaegeri* Pilg.; kingdom: Plantae; family: Poaceae; genus: Eleusine; specificEpithet: jaegeri; scientificNameAuthorship: Pilg.; **Location:** continent: Africa; country: Tanzania; stateProvince: Arusha; county: Ngorongoro; locality: Ngorongoro Crater; verbatimLocality: Crater; **Event:** eventDate: 1956-08-03; **Record Level:** institutionCode: EA; collectionCode: Herbarium; ownerInstitutionCode: EA; basisOfRecord: PreservedSpecimen
- **Type status:** Other material. **Occurrence:** catalogNumber: 797; recordNumber: 122; recordedBy: Goddard, J; **Taxon:** scientificName: *Eleusine jaegeri* Pilg.; kingdom: Plantae; family: Poaceae; genus: Eleusine; specificEpithet: jaegeri; scientificNameAuthorship: Pilg.; **Location:** continent: Africa; country: Tanzania; stateProvince: Arusha; county: Ngorongoro; locality: Ngorongoro; minimumElevationInMeters: 2317; maximumElevationInMeters: 2317; **Event:** eventDate: 1965-06-10; **Record Level:** institutionCode: EA; collectionCode: Herbarium; ownerInstitutionCode: EA; basisOfRecord: PreservedSpecimen
- **Type status:** Other material. **Occurrence:** catalogNumber: 798; recordNumber: 244; recordedBy: Khayota, B; **Taxon:** scientificName: *Eleusine jaegeri* Pilg.; kingdom: Plantae; family: Poaceae; genus: Eleusine; specificEpithet: jaegeri; scientificNameAuthorship: Pilg.; **Location:** continent: Africa; country: Tanzania; stateProvince: Arusha; county: Ngorongoro; locality: Ngorongoro Crater; verbatimLocality: The rim of the crater.; minimumElevationInMeters: 2042; **Event:** eventDate: 1985-12-09; **Record Level:** institutionCode: EA; collectionCode: Herbarium; ownerInstitutionCode: EA; basisOfRecord: PreservedSpecimen
- **Type status:** Other material. **Occurrence:** catalogNumber: 799; recordNumber: 89; recordedBy: Moreau, RE; Moreau, WM; **Taxon:** scientificName: *Eleusine jaegeri* Pilg.; kingdom: Plantae; family: Poaceae; genus: Eleusine; specificEpithet: jaegeri; scientificNameAuthorship: Pilg.; **Location:** continent: Africa; country: Tanzania; stateProvince: Arusha; county: Ngorongoro; locality: Ngorongoro; minimumElevationInMeters: 2286; maximumElevationInMeters: 2286; **Event:** eventDate: 1935-1; **Record Level:** institutionCode: EA; collectionCode: Herbarium; ownerInstitutionCode: EA; basisOfRecord: PreservedSpecimen
- **Type status:** Other material. **Occurrence:** catalogNumber: K000766126; recordNumber: 3368; recordedBy: Greenway, PJ; **Taxon:** scientificName: *Eleusine jaegeri* Pilg.; kingdom: Plantae; family: Poaceae; genus: Eleusine; specificEpithet: jaegeri; scientificNameAuthorship: Pilg.; **Location:** continent: Africa; country: Tanzania; stateProvince: Arusha; county: Ngorongoro; locality: Ngorongoro Crater; verbatimLocality: South East slope of the crater.; minimumElevationInMeters: 2439; maximumElevationInMeters: 2439; **Event:** eventDate: 1933-02-22; **Record Level:** institutionCode: K; collectionCode: Herbarium; ownerInstitutionCode: K; basisOfRecord: PreservedSpecimen
- **Type status:** Other material. **Occurrence:** catalogNumber: 819; recordNumber: 3368; recordedBy: Greenway, PJ; **Taxon:** scientificName: *Eleusine jaegeri* Pilg.; kingdom: Plantae; family: Poaceae; genus: Eleusine; specificEpithet: jaegeri; scientificNameAuthorship: Pilg.; **Location:** continent: Africa; country: Tanzania; stateProvince: Arusha; county: Ngorongoro; locality: Ngorongoro Crater; verbatimLocality: South East slope of the crater.; minimumElevationInMeters: 2439; maximumElevationInMeters: 2439; **Event:** eventDate: 1933-02-22; **Record Level:** institutionCode: EA; collectionCode: Herbarium; ownerInstitutionCode: EA; basisOfRecord: PreservedSpecimen
- **Type status:** Other material. **Occurrence:** catalogNumber: 821; recordNumber: 46; recordedBy: Frame, GW; **Taxon:** scientificName: *Eleusine jaegeri* Pilg.; kingdom: Plantae; family: Poaceae; genus: Eleusine; specificEpithet: jaegeri; scientificNameAuthorship: Pilg.; **Location:** continent: Africa; country: Tanzania; stateProvince: Arusha; county: Ngorongoro; locality: Empakai Crater; verbatimLocality: Empakaai crater, Ngorongoro, top of south rim.; minimumElevationInMeters: 3018; decimalLatitude: -2.916667; decimalLongitude: 35.8; **Event:** eventDate: 1972-09-03; **Record Level:** institutionCode: EA; collectionCode: Herbarium; ownerInstitutionCode: EA; basisOfRecord: PreservedSpecimen
- **Type status:** Other material. **Occurrence:** catalogNumber: 822; recordNumber: 43; recordedBy: Frame, GW; **Taxon:** scientificName: *Eleusine jaegeri* Pilg.; kingdom: Plantae; family: Poaceae; genus: Eleusine; specificEpithet: jaegeri; scientificNameAuthorship: Pilg.; **Location:** continent: Africa; country: Tanzania; stateProvince: Arusha; county: Ngorongoro; locality: Empakai Crater; verbatimLocality: Empakaai crater, outer eastern slope of Ngorongoro.; minimumElevationInMeters: 2130; decimalLatitude: -2.916667; decimalLongitude: 35.8; **Event:** eventDate: 1972-08-14; **Record Level:** institutionCode: EA; collectionCode: Herbarium; ownerInstitutionCode: EA; basisOfRecord: PreservedSpecimen
- **Type status:** Other material. **Occurrence:** catalogNumber: K000766124; recordNumber: s.n.; recordedBy: Napper, D; **Taxon:** scientificName: *Eleusine jaegeri* Pilg.; kingdom: Plantae; family: Poaceae; genus: Eleusine; specificEpithet: jaegeri; scientificNameAuthorship: Pilg.; **Location:** continent: Africa; country: Tanzania; stateProvince: Arusha; county: Ngorongoro; locality: Ngorongoro Crater; verbatimLocality: Crater; **Event:** eventDate: 1956-08-03; **Record Level:** institutionCode: K; collectionCode: Herbarium; ownerInstitutionCode: K; basisOfRecord: PreservedSpecimen
- **Type status:** Other material. **Occurrence:** catalogNumber: K000766132; recordNumber: 46; recordedBy: Frame, GW; **Taxon:** scientificName: *Eleusine jaegeri* Pilg.; kingdom: Plantae; family: Poaceae; genus: Eleusine; specificEpithet: jaegeri; scientificNameAuthorship: Pilg.; **Location:** continent: Africa; country: Tanzania; stateProvince: Arusha; county: Ngorongoro; locality: Empakai Crater; verbatimLocality: Empakaai crater, Ngorongoro, top of south rim.; minimumElevationInMeters: 3018; decimalLatitude: -2.916667; decimalLongitude: 35.8; **Event:** eventDate: 1972-09-03; **Record Level:** institutionCode: K; collectionCode: Herbarium; ownerInstitutionCode: K; basisOfRecord: PreservedSpecimen
- **Type status:** Other material. **Occurrence:** catalogNumber: K000766136; recordNumber: 4302; recordedBy: Burtt, BD; **Taxon:** scientificName: *Eleusine jaegeri* Pilg.; kingdom: Plantae; family: Poaceae; genus: Eleusine; specificEpithet: jaegeri; scientificNameAuthorship: Pilg.; **Location:** continent: Africa; country: Tanzania; stateProvince: Arusha; county: Ngorongoro; locality: Ngorongoro Crater; verbatimLocality: Crater wall above Laroda.; minimumElevationInMeters: 2439; decimalLatitude: -3.166667; decimalLongitude: 35.583333; **Event:** eventDate: 1932-09-12; **Record Level:** institutionCode: K; collectionCode: Herbarium; ownerInstitutionCode: K; basisOfRecord: PreservedSpecimen
- **Type status:** Other material. **Occurrence:** catalogNumber: K000766127; recordNumber: 90; recordedBy: Moreau; Moreau; **Taxon:** scientificName: *Eleusine jaegeri* Pilg.; kingdom: Plantae; family: Poaceae; genus: Eleusine; specificEpithet: jaegeri; scientificNameAuthorship: Pilg.; **Location:** continent: Africa; country: Tanzania; stateProvince: Arusha; county: Ngorongoro; locality: Oldeani Mt; decimalLatitude: -3.266667; decimalLongitude: 35.433333; **Event:** eventDate: 1935-1; **Record Level:** institutionCode: K; collectionCode: Herbarium; ownerInstitutionCode: K; basisOfRecord: PreservedSpecimen
- **Type status:** Other material. **Occurrence:** catalogNumber: 1012; recordNumber: 90; recordedBy: Moreau; Moreau; **Taxon:** scientificName: *Eleusine jaegeri* Pilg.; kingdom: Plantae; family: Poaceae; genus: Eleusine; specificEpithet: jaegeri; scientificNameAuthorship: Pilg.; **Location:** continent: Africa; country: Tanzania; stateProvince: Arusha; county: Ngorongoro; locality: Oldeani Mt; decimalLatitude: -3.266667; decimalLongitude: 35.433333; **Event:** eventDate: 1935-1; **Record Level:** institutionCode: EA; collectionCode: Herbarium; ownerInstitutionCode: EA; basisOfRecord: PreservedSpecimen
- **Type status:** Other material. **Occurrence:** catalogNumber: K000766129; recordNumber: 19056; recordedBy: Raynal, J; **Taxon:** scientificName: *Eleusine jaegeri* Pilg.; kingdom: Plantae; family: Poaceae; genus: Eleusine; specificEpithet: jaegeri; scientificNameAuthorship: Pilg.; **Location:** continent: Africa; country: Tanzania; stateProvince: Arusha; county: Ngorongoro; locality: Ngorongoro Crater; verbatimLocality: Crater south rim, outer slopes. Junction to Lemala.; minimumElevationInMeters: 2100; maximumElevationInMeters: 2350; decimalLatitude: -3.166667; decimalLongitude: 35.583333; **Event:** eventDate: 1977-09-15; **Record Level:** institutionCode: K; collectionCode: Herbarium; ownerInstitutionCode: K; basisOfRecord: PreservedSpecimen
- **Type status:** Other material. **Occurrence:** catalogNumber: K000766125; recordNumber: 9149; recordedBy: Greenway, PJ; **Taxon:** scientificName: *Eleusine jaegeri* Pilg.; kingdom: Plantae; family: Poaceae; genus: Eleusine; specificEpithet: jaegeri; scientificNameAuthorship: Pilg.; **Location:** continent: Africa; country: Tanzania; stateProvince: Arusha; county: Ngorongoro; locality: Nainokanoka; minimumElevationInMeters: 2682; decimalLatitude: -3.016667; decimalLongitude: 35.683333; **Event:** eventDate: 1956-12-09; **Record Level:** institutionCode: K; collectionCode: Herbarium; ownerInstitutionCode: K; basisOfRecord: PreservedSpecimen
- **Type status:** Other material. **Occurrence:** catalogNumber: K000766137; recordNumber: 49; recordedBy: Salt, G; **Taxon:** scientificName: *Eleusine jaegeri* Pilg.; kingdom: Plantae; family: Poaceae; genus: Eleusine; specificEpithet: jaegeri; scientificNameAuthorship: Pilg.; **Location:** continent: Africa; country: Tanzania; stateProvince: Arusha; county: Ngorongoro; locality: Lemagarut Mt.; verbatimLocality: M.Oldeani.; minimumElevationInMeters: 2896; decimalLatitude: -3.166667; decimalLongitude: 35.35; **Event:** eventDate: 1948-11-01; **Record Level:** institutionCode: K; collectionCode: Herbarium; ownerInstitutionCode: K; basisOfRecord: PreservedSpecimen
- **Type status:** Other material. **Occurrence:** catalogNumber: K000766138; recordNumber: G41; recordedBy: Bogdan; **Taxon:** scientificName: *Eleusine jaegeri* Pilg.; kingdom: Plantae; family: Poaceae; genus: Eleusine; specificEpithet: jaegeri; scientificNameAuthorship: Pilg.; **Location:** continent: Africa; country: Tanzania; stateProvince: Arusha; county: Ngorongoro; locality: Ngorongoro Crater; verbatimLocality: West rim of crater.; minimumElevationInMeters: 2439; decimalLatitude: -3.166667; decimalLongitude: 35.583333; **Event:** eventDate: 1961-6; **Record Level:** institutionCode: K; collectionCode: Herbarium; ownerInstitutionCode: K; basisOfRecord: PreservedSpecimen
- **Type status:** Other material. **Occurrence:** catalogNumber: K000766139; recordNumber: 898; recordedBy: Pole Evans, IB; Erens, J; **Taxon:** scientificName: *Eleusine jaegeri* Pilg.; kingdom: Plantae; family: Poaceae; genus: Eleusine; specificEpithet: jaegeri; scientificNameAuthorship: Pilg.; **Location:** continent: Africa; country: Tanzania; stateProvince: Arusha; county: Ngorongoro; locality: Ngorongoro Crater; verbatimLocality: crater edge.; decimalLatitude: -3.166667; decimalLongitude: 35.583333; **Event:** eventDate: 1938-06-21; **Record Level:** institutionCode: K; collectionCode: Herbarium; ownerInstitutionCode: K; basisOfRecord: PreservedSpecimen
- **Type status:** Other material. **Occurrence:** catalogNumber: K001087163; recordNumber: 981; recordedBy: Pole Evans, IB; Erens, J; **Taxon:** scientificName: *Eleusine jaegeri* Pilg.; kingdom: Plantae; family: Poaceae; genus: Eleusine; specificEpithet: jaegeri; scientificNameAuthorship: Pilg.; **Location:** continent: Africa; country: Tanzania; stateProvince: Arusha; county: Ngorongoro; locality: Ngorongoro Crater; verbatimLocality: On the top ridge of Ngorongoro crater.; decimalLatitude: -3.166667; decimalLongitude: 35.583333; **Event:** eventDate: 1938-06-24; **Record Level:** institutionCode: K; collectionCode: Herbarium; ownerInstitutionCode: K; basisOfRecord: PreservedSpecimen
- **Type status:** Other material. **Occurrence:** catalogNumber: K001087164; recordNumber: 979; recordedBy: Pole Evans, IB; Erens, J; **Taxon:** scientificName: *Eleusine jaegeri* Pilg.; kingdom: Plantae; family: Poaceae; genus: Eleusine; specificEpithet: jaegeri; scientificNameAuthorship: Pilg.; **Location:** continent: Africa; country: Tanzania; stateProvince: Arusha; county: Ngorongoro; locality: Ngorongoro Crater; verbatimLocality: On the top of the Ngorongoro crater.; decimalLatitude: -3.166667; decimalLongitude: 35.583333; **Event:** eventDate: 1938-06-24; **Record Level:** institutionCode: K; collectionCode: Herbarium; ownerInstitutionCode: K; basisOfRecord: PreservedSpecimen

##### Distribution

East Africa

#### Eleusine multiflora

Hochst. ex A.Rich.

##### Materials

- **Type status:** Other material. **Occurrence:** catalogNumber: 297; recordNumber: 10574; recordedBy: Greenway, PJ; **Taxon:** scientificName: *Eleusine multiflora* Hochst. ex A.Rich.; kingdom: Plantae; family: Poaceae; genus: Eleusine; specificEpithet: multiflora; scientificNameAuthorship: Hochst. ex A.Rich.; **Location:** continent: Africa; country: Tanzania; stateProvince: Mara; county: Serengeti; locality: Seronera; verbatimLocality: Serengeti.; minimumElevationInMeters: 1432; decimalLatitude: -2.45; decimalLongitude: 34.833333; **Event:** eventDate: 1962-04-09; **Record Level:** institutionCode: EA; collectionCode: Herbarium; ownerInstitutionCode: EA; basisOfRecord: PreservedSpecimen
- **Type status:** Other material. **Occurrence:** catalogNumber: 610; recordNumber: 24272; recordedBy: Peterson, PM; Soreng, RJ; Romaschenko, K; Mbago, F; **Taxon:** scientificName: *Eleusine multiflora* Hochst. ex A.Rich.; kingdom: Plantae; family: Poaceae; genus: Eleusine; specificEpithet: multiflora; scientificNameAuthorship: Hochst. ex A.Rich.; **Location:** continent: Africa; country: Tanzania; stateProvince: Shinyanga; locality: Naabi Hill Gate; verbatimLocality: Serengeti National Park, at 12 km NW of Naabi Hill Gate.; minimumElevationInMeters: 1651; decimalLatitude: -2.73597; decimalLongitude: 34.95284; **Event:** eventDate: 2012-06-16; **Record Level:** institutionCode: US; collectionCode: Herbarium; ownerInstitutionCode: US; basisOfRecord: PreservedSpecimen
- **Type status:** Other material. **Occurrence:** catalogNumber: 787; recordNumber: B2584; recordedBy: Bally, PRO; **Taxon:** scientificName: *Eleusine multiflora* Hochst. ex A.Rich.; kingdom: Plantae; family: Poaceae; genus: Eleusine; specificEpithet: multiflora; scientificNameAuthorship: Hochst. ex A.Rich.; **Location:** continent: Africa; country: Tanzania; stateProvince: Arusha; county: Ngorongoro; locality: Ngorongoro Crater; minimumElevationInMeters: 1524; decimalLatitude: -3; decimalLongitude: 35; **Event:** eventDate: 1941-4; **Record Level:** institutionCode: EA; collectionCode: Herbarium; ownerInstitutionCode: EA; basisOfRecord: PreservedSpecimen
- **Type status:** Other material. **Occurrence:** catalogNumber: 788; recordNumber: 5652; recordedBy: Newbould, JB; **Taxon:** scientificName: *Eleusine multiflora* Hochst. ex A.Rich.; kingdom: Plantae; family: Poaceae; genus: Eleusine; specificEpithet: multiflora; scientificNameAuthorship: Hochst. ex A.Rich.; **Location:** continent: Africa; country: Tanzania; stateProvince: Arusha; county: Ngorongoro; locality: Ngorongoro; verbatimLocality: Old Boma.; minimumElevationInMeters: 2134; decimalLatitude: -3; decimalLongitude: 35; **Event:** eventDate: 1961-02-11; **Record Level:** institutionCode: EA; collectionCode: Herbarium; ownerInstitutionCode: EA; basisOfRecord: PreservedSpecimen
- **Type status:** Other material. **Occurrence:** catalogNumber: 789; recordNumber: 501; recordedBy: Frame, GW; **Taxon:** scientificName: *Eleusine multiflora* Hochst. ex A.Rich.; kingdom: Plantae; family: Poaceae; genus: Eleusine; specificEpithet: multiflora; scientificNameAuthorship: Hochst. ex A.Rich.; **Location:** continent: Africa; country: Tanzania; stateProvince: Arusha; county: Ngorongoro; locality: Ngorongoro Crater; verbatimLocality: Empakaai Crater, Conservation Area; top of East rim.; minimumElevationInMeters: 2450; decimalLatitude: -3; decimalLongitude: 35; **Event:** eventDate: 1974-01-22; **Record Level:** institutionCode: EA; collectionCode: Herbarium; ownerInstitutionCode: EA; basisOfRecord: PreservedSpecimen
- **Type status:** Other material. **Occurrence:** catalogNumber: K001087160; recordNumber: 10574; recordedBy: Greenway, PJ; **Taxon:** scientificName: *Eleusine multiflora* Hochst. ex A.Rich.; kingdom: Plantae; family: Poaceae; genus: Eleusine; specificEpithet: multiflora; scientificNameAuthorship: Hochst. ex A.Rich.; **Location:** continent: Africa; country: Tanzania; stateProvince: Mara; county: Serengeti; locality: Seronera; verbatimLocality: Serengeti.; minimumElevationInMeters: 1432; decimalLatitude: -2.45; decimalLongitude: 34.833333; **Event:** eventDate: 1962-04-09; **Record Level:** institutionCode: K; collectionCode: Herbarium; ownerInstitutionCode: K; basisOfRecord: PreservedSpecimen
- **Type status:** Other material. **Occurrence:** catalogNumber: K001087161; recordNumber: 5652; recordedBy: Newbould, JB; **Taxon:** scientificName: *Eleusine multiflora* Hochst. ex A.Rich.; kingdom: Plantae; family: Poaceae; genus: Eleusine; specificEpithet: multiflora; scientificNameAuthorship: Hochst. ex A.Rich.; **Location:** continent: Africa; country: Tanzania; stateProvince: Arusha; county: Ngorongoro; locality: Ngorongoro; verbatimLocality: Old Boma.; minimumElevationInMeters: 2134; decimalLatitude: -3; decimalLongitude: 35; **Event:** eventDate: 1961-02-11; **Record Level:** institutionCode: K; collectionCode: Herbarium; ownerInstitutionCode: K; basisOfRecord: PreservedSpecimen
- **Type status:** Other material. **Occurrence:** catalogNumber: K001087162; recordNumber: 2787; recordedBy: Chuwa, S; **Taxon:** scientificName: *Eleusine multiflora* Hochst. ex A.Rich.; kingdom: Plantae; family: Poaceae; genus: Eleusine; specificEpithet: multiflora; scientificNameAuthorship: Hochst. ex A.Rich.; **Location:** continent: Africa; country: Tanzania; stateProvince: Arusha; county: Ngorongoro; locality: Olduvai Gorge; minimumElevationInMeters: 1400; decimalLatitude: -2.95; decimalLongitude: 35.166667; **Event:** eventDate: 1989-05-30; **Record Level:** institutionCode: K; collectionCode: Herbarium; ownerInstitutionCode: K; basisOfRecord: PreservedSpecimen

##### Distribution

Eastern Africa & Arabia

#### Elionurus muticus

(Spreng.) Kuntze

Elionurus argenteus Nees

##### Materials

- **Type status:** Other material. **Occurrence:** catalogNumber: K000984053; recordNumber: 10769; recordedBy: Greenway, PJ; **Taxon:** scientificName: *Elionurus argenteus* Nees; kingdom: Plantae; family: Poaceae; genus: Elionurus; specificEpithet: argenteus; scientificNameAuthorship: Nees; **Location:** continent: Africa; country: Tanzania; stateProvince: Mara; county: Serengeti; locality: Mara River Guard Post; minimumElevationInMeters: 1280; decimalLatitude: -1.583333; decimalLongitude: 34.833333; **Event:** eventDate: 1962-08-21; **Record Level:** institutionCode: K; collectionCode: Herbarium; ownerInstitutionCode: K; basisOfRecord: PreservedSpecimen
- **Type status:** Other material. **Occurrence:** catalogNumber: K000984054; recordNumber: 10238; recordedBy: Greenway, PJ; **Taxon:** scientificName: *Elionurus argenteus* Nees; kingdom: Plantae; family: Poaceae; genus: Elionurus; specificEpithet: argenteus; scientificNameAuthorship: Nees; **Location:** continent: Africa; country: Tanzania; stateProvince: Mara; county: Serengeti; locality: Mara River Guard Post; verbatimLocality: Mara River guard post, mile 87 from Seronera.; minimumElevationInMeters: 1280; decimalLatitude: -1.583333; decimalLongitude: 34.833333; **Event:** eventDate: 1961-10-04; **Record Level:** institutionCode: K; collectionCode: Herbarium; ownerInstitutionCode: K; basisOfRecord: PreservedSpecimen
- **Type status:** Other material. **Occurrence:** catalogNumber: K000984055; recordNumber: 10307; recordedBy: Greenway, PJ; **Taxon:** scientificName: *Elionurus argenteus* Nees; kingdom: Plantae; family: Poaceae; genus: Elionurus; specificEpithet: argenteus; scientificNameAuthorship: Nees; **Location:** continent: Africa; country: Tanzania; stateProvince: Mara; county: Serengeti; locality: Lobo range; verbatimLocality: East boundary, Lobo range; minimumElevationInMeters: 1981; decimalLatitude: -1.966667; decimalLongitude: 35.216667; **Event:** eventDate: 1961-05-18; **Record Level:** institutionCode: K; collectionCode: Herbarium; ownerInstitutionCode: K; basisOfRecord: PreservedSpecimen

##### Distribution

Widespread

#### Enneapogon sp.

##### Materials

- **Type status:** Other material. **Occurrence:** catalogNumber: 612; recordNumber: 24265; recordedBy: Peterson, PM; Soreng, RJ; Romaschenko, K; Mbago, F; **Taxon:** scientificName: Enneapogon; kingdom: Plantae; family: Poaceae; genus: Enneapogon; **Location:** continent: Africa; country: Tanzania; stateProvince: Shinyanga; locality: Naabi Hill Gate; verbatimLocality: Serengeti National Park, Naabi Hill Gate (at 0.5 km N).; minimumElevationInMeters: 1734; decimalLatitude: -2.83139; decimalLongitude: 34.99672; **Event:** eventDate: 2012-06-16; **Record Level:** institutionCode: US; collectionCode: Herbarium; ownerInstitutionCode: US; basisOfRecord: PreservedSpecimen

#### Enneapogon cenchroides

(Licht.) C.E.Hubb

##### Materials

- **Type status:** Other material. **Occurrence:** catalogNumber: K000984272; recordNumber: 6356; recordedBy: Newbould, JB; **Taxon:** scientificName: *Enneapogon cenchroides* (Licht.) C.E.Hubb; kingdom: Plantae; family: Poaceae; genus: Enneapogon; specificEpithet: cenchroides; scientificNameAuthorship: (Licht.) C.E.Hubb; **Location:** continent: Africa; country: Tanzania; stateProvince: Arusha; county: Ngorongoro; locality: Mosonik; verbatimLocality: West of Lake Natron; minimumElevationInMeters: 1219; decimalLatitude: -2.583333; decimalLongitude: 35.8; **Event:** eventDate: 1962-11-29; **Record Level:** institutionCode: K; collectionCode: Herbarium; ownerInstitutionCode: K; basisOfRecord: PreservedSpecimen
- **Type status:** Other material. **Occurrence:** catalogNumber: K000984273; recordNumber: 10345; recordedBy: Greenway, PJ; **Taxon:** scientificName: *Enneapogon cenchroides* (Licht.) C.E.Hubb; kingdom: Plantae; family: Poaceae; genus: Enneapogon; specificEpithet: cenchroides; scientificNameAuthorship: (Licht.) C.E.Hubb; **Location:** continent: Africa; country: Tanzania; stateProvince: Mara; county: Serengeti; locality: Banagi; verbatimLocality: Mgungu River; minimumElevationInMeters: 1341; decimalLatitude: -2.3; decimalLongitude: 34.833333; **Event:** eventDate: 1961-05-31; **Record Level:** institutionCode: K; collectionCode: Herbarium; ownerInstitutionCode: K; basisOfRecord: PreservedSpecimen
- **Type status:** Other material. **Occurrence:** catalogNumber: K000984274; recordNumber: 6294; recordedBy: Newbould, JB; **Taxon:** scientificName: *Enneapogon cenchroides* (Licht.) C.E.Hubb; kingdom: Plantae; family: Poaceae; genus: Enneapogon; specificEpithet: cenchroides; scientificNameAuthorship: (Licht.) C.E.Hubb; **Location:** continent: Africa; country: Tanzania; stateProvince: Mara; county: Serengeti; locality: Salambala; verbatimLocality: E. Serengeti; minimumElevationInMeters: 1829; decimalLatitude: -2.333333; decimalLongitude: 34.833333; **Event:** eventDate: 1962-11-21; **Record Level:** institutionCode: K; collectionCode: Herbarium; ownerInstitutionCode: K; basisOfRecord: PreservedSpecimen
- **Type status:** Other material. **Occurrence:** catalogNumber: 611; recordNumber: s.n.; recordedBy: Mboya, E; **Taxon:** scientificName: *Enneapogon cenchroides* (Licht.) C.E.Hubb; kingdom: Plantae; family: Poaceae; genus: Enneapogon; specificEpithet: cenchroides; scientificNameAuthorship: (Licht.) C.E.Hubb; **Location:** continent: Africa; country: Tanzania; stateProvince: Mara; county: Serengeti; locality: Serengeti Research Centre; verbatimLocality: T1. Serengeti National Park. Serengeti Research Centre. Tawiri Hostel.; minimumElevationInMeters: 1551; decimalLatitude: -2.38; decimalLongitude: 34.85; **Event:** eventDate: 2004-03-04; **Record Level:** institutionCode: MO; collectionCode: Herbarium; ownerInstitutionCode: MO; basisOfRecord: PreservedSpecimen

##### Distribution

Tropical Africa, Arabia & Asia

#### Enneapogon desvauxii

P.Beauv.

Enneapogon brachystachyus Stapf

##### Materials

- **Type status:** Other material. **Occurrence:** catalogNumber: K001087166; recordNumber: 202; recordedBy: Robson, TO; **Taxon:** scientificName: *Enneapogon brachystachyus* Stapf; kingdom: Plantae; family: Poaceae; genus: Enneapogon; specificEpithet: brachystachyus; scientificNameAuthorship: Stapf; **Location:** continent: Africa; country: Tanzania; stateProvince: Arusha; county: Ngorongoro; locality: Ngorongoro; verbatimLocality: Slope of Ibalba, from Ngorongoro.; decimalLatitude: -3; decimalLongitude: 35.5; **Event:** eventDate: 1957-03-12; **Record Level:** institutionCode: K; collectionCode: Herbarium; ownerInstitutionCode: K; basisOfRecord: PreservedSpecimen
- **Type status:** Other material. **Occurrence:** catalogNumber: K001087165; recordNumber: 412; recordedBy: Paulo, S; **Taxon:** scientificName: *Enneapogon desvauxii* P.Beauv.; kingdom: Plantae; family: Poaceae; genus: Enneapogon; specificEpithet: desvauxii; scientificNameAuthorship: P.Beauv.; **Location:** continent: Africa; country: Tanzania; stateProvince: Arusha; county: Ngorongoro; locality: Olbalbal; verbatimLocality: North of Olbalbal.; decimalLatitude: -2.95; decimalLongitude: 35.583333; **Event:** eventDate: 1958-04-29; **Record Level:** institutionCode: K; collectionCode: Herbarium; ownerInstitutionCode: K; basisOfRecord: PreservedSpecimen

##### Distribution

Tropical Africa, Asia & Americas

#### Enneapogon persicus

Boiss.

Enneapogon elegans (Nees ex Steud.) Stapf

##### Materials

- **Type status:** Other material. **Occurrence:** catalogNumber: K000984267; recordNumber: 10071; recordedBy: Greenway, PJ; **Taxon:** scientificName: *Enneapogon persicus* Boiss.; kingdom: Plantae; family: Poaceae; genus: Enneapogon; specificEpithet: persicus; scientificNameAuthorship: Boiss.; **Location:** continent: Africa; country: Tanzania; stateProvince: Mara; county: Serengeti; locality: Seronera; verbatimLocality: Dam Paddock, Serengeti; minimumElevationInMeters: 1448; decimalLatitude: -2.45; decimalLongitude: 34.833333; **Event:** eventDate: 1961-04-19; **Record Level:** institutionCode: K; collectionCode: Herbarium; ownerInstitutionCode: K; basisOfRecord: PreservedSpecimen
- **Type status:** Other material. **Occurrence:** catalogNumber: K000984268; recordNumber: 13605; recordedBy: Greenway, PJ; **Taxon:** scientificName: *Enneapogon persicus* Boiss.; kingdom: Plantae; family: Poaceae; genus: Enneapogon; specificEpithet: persicus; scientificNameAuthorship: Boiss.; **Location:** continent: Africa; country: Tanzania; stateProvince: Arusha; county: Ngorongoro; locality: Ngorongoro Crater; verbatimLocality: floor; decimalLatitude: -3.166667; decimalLongitude: 35.583333; **Event:** eventDate: 1966-7; **Record Level:** institutionCode: K; collectionCode: Herbarium; ownerInstitutionCode: K; basisOfRecord: PreservedSpecimen
- **Type status:** Other material. **Occurrence:** catalogNumber: K000984269; recordNumber: 10524; recordedBy: Greenway, PJ; **Taxon:** scientificName: *Enneapogon persicus* Boiss.; kingdom: Plantae; family: Poaceae; genus: Enneapogon; specificEpithet: persicus; scientificNameAuthorship: Boiss.; **Location:** continent: Africa; country: Tanzania; stateProvince: Arusha; county: Ngorongoro; locality: Gol Kopjes; verbatimLocality: mile 8 E. of Naabi Hill and three miles from the Eastern boundary; minimumElevationInMeters: 1646; decimalLatitude: -2.7; decimalLongitude: 35.433333; **Event:** eventDate: 1962-03-15; **Record Level:** institutionCode: K; collectionCode: Herbarium; ownerInstitutionCode: K; basisOfRecord: PreservedSpecimen
- **Type status:** Other material. **Occurrence:** catalogNumber: K000984270; recordNumber: 10367; recordedBy: Greenway, PJ; Tanner, M; **Taxon:** scientificName: *Enneapogon persicus* Boiss.; kingdom: Plantae; family: Poaceae; genus: Enneapogon; specificEpithet: persicus; scientificNameAuthorship: Boiss.; **Location:** continent: Africa; country: Tanzania; stateProvince: Shinyanga; locality: Ipumba Kopje; verbatimLocality: S. Boundary; minimumElevationInMeters: 1524; decimalLatitude: -3.266667; decimalLongitude: 34.833333; **Event:** eventDate: 1961-06-06; **Record Level:** institutionCode: K; collectionCode: Herbarium; ownerInstitutionCode: K; basisOfRecord: PreservedSpecimen
- **Type status:** Other material. **Occurrence:** catalogNumber: K000984271; recordNumber: 10347; recordedBy: Greenway, PJ; **Taxon:** scientificName: *Enneapogon persicus* Boiss.; kingdom: Plantae; family: Poaceae; genus: Enneapogon; specificEpithet: persicus; scientificNameAuthorship: Boiss.; **Location:** continent: Africa; country: Tanzania; stateProvince: Mara; county: Serengeti; locality: Banagi; verbatimLocality: Mgungu river; minimumElevationInMeters: 1341; decimalLatitude: -2.3; decimalLongitude: 34.833333; **Event:** eventDate: 1961-05-31; **Record Level:** institutionCode: K; collectionCode: Herbarium; ownerInstitutionCode: K; basisOfRecord: PreservedSpecimen

##### Distribution

Tropical Africa & Asia

#### Enteropogon sp.

##### Materials

- **Type status:** Other material. **Occurrence:** catalogNumber: 613; recordNumber: s.n.; recordedBy: Mboya, E; **Taxon:** scientificName: Enteropogon; kingdom: Plantae; family: Poaceae; genus: Enteropogon; **Location:** continent: Africa; country: Tanzania; stateProvince: Mara; county: Serengeti; locality: Musabi plains; verbatimLocality: T1. Serengeti National Park. Musabi plains.; minimumElevationInMeters: 1297; decimalLatitude: -2.28; decimalLongitude: 34.55; **Event:** eventDate: 2004-03-07; **Record Level:** institutionCode: MO; collectionCode: Herbarium; ownerInstitutionCode: MO; basisOfRecord: PreservedSpecimen

#### Enteropogon macrostachyus

(Hochst. ex A.Rich) Munro ex Benth.

##### Materials

- **Type status:** Other material. **Occurrence:** catalogNumber: K000766110; recordNumber: 9928; recordedBy: Greenway, PJ; **Taxon:** scientificName: *Enteropogon macrostachyus* (Hochst. ex A.Rich) Munro ex Benth.; kingdom: Plantae; family: Poaceae; genus: Enteropogon; specificEpithet: macrostachyus; scientificNameAuthorship: (Hochst. ex A.Rich) Munro ex Benth.; **Location:** continent: Africa; country: Tanzania; stateProvince: Mara; county: Serengeti; locality: Seronera; minimumElevationInMeters: 1554; decimalLatitude: -2.45; decimalLongitude: 34.833333; **Event:** eventDate: 1961-03-29; **Record Level:** institutionCode: K; collectionCode: Herbarium; ownerInstitutionCode: K; basisOfRecord: PreservedSpecimen

##### Distribution

Tropical Africa & Arabia

#### Eragrostis sp.

##### Materials

- **Type status:** Other material. **Occurrence:** catalogNumber: 617; recordNumber: 24261; recordedBy: Peterson, PM; Soreng, RJ; Romaschenko, K; Mbago, F; **Taxon:** scientificName: Eragrostis; kingdom: Plantae; family: Poaceae; genus: Eragrostis; **Location:** continent: Africa; country: Tanzania; stateProvince: Shinyanga; locality: Naabi Hill Gate; verbatimLocality: Serengeti National Park, Naabi Hill Gate (at 0.5 km N).; minimumElevationInMeters: 1734; decimalLatitude: -2.83139; decimalLongitude: 34.99672; **Event:** eventDate: 2012-06-16; **Record Level:** institutionCode: US; collectionCode: Herbarium; ownerInstitutionCode: US; basisOfRecord: PreservedSpecimen
- **Type status:** Other material. **Occurrence:** catalogNumber: 618; recordNumber: 24267; recordedBy: Peterson, PM; Soreng, RJ; Romaschenko, K; Mbago, F; **Taxon:** scientificName: Eragrostis; kingdom: Plantae; family: Poaceae; genus: Eragrostis; **Location:** continent: Africa; country: Tanzania; stateProvince: Shinyanga; locality: Naabi Hill Gate; verbatimLocality: Serengeti National Park, at 12 km NW of Naabi Hill Gate.; minimumElevationInMeters: 1651; decimalLatitude: -2.73597; decimalLongitude: 34.95284; **Event:** eventDate: 2012-06-16; **Record Level:** institutionCode: US; collectionCode: Herbarium; ownerInstitutionCode: US; basisOfRecord: PreservedSpecimen
- **Type status:** Other material. **Occurrence:** catalogNumber: 619; recordNumber: 24271; recordedBy: Peterson, PM; Soreng, RJ; Romaschenko, K; Mbago, F; **Taxon:** scientificName: Eragrostis; kingdom: Plantae; family: Poaceae; genus: Eragrostis; **Location:** continent: Africa; country: Tanzania; stateProvince: Shinyanga; locality: Naabi Hill Gate; verbatimLocality: Serengeti National Park, at 12 km NW of Naabi Hill Gate.; minimumElevationInMeters: 1651; decimalLatitude: -2.73597; decimalLongitude: 34.95284; **Event:** eventDate: 2012-06-16; **Record Level:** institutionCode: US; collectionCode: Herbarium; ownerInstitutionCode: US; basisOfRecord: PreservedSpecimen
- **Type status:** Other material. **Occurrence:** catalogNumber: 620; recordNumber: 24283; recordedBy: Peterson, PM; Soreng, RJ; Romaschenko, K; Mbago, F; **Taxon:** scientificName: Eragrostis; kingdom: Plantae; family: Poaceae; genus: Eragrostis; **Location:** continent: Africa; country: Tanzania; stateProvince: Mara; county: Serengeti; locality: Mbuzi Mare camp; verbatimLocality: Serengeti National Park, near Mbuzi Mare camp.; minimumElevationInMeters: 1552; decimalLatitude: -2.23332; decimalLongitude: 34.96467; **Event:** eventDate: 2012-06-17; **Record Level:** institutionCode: US; collectionCode: Herbarium; ownerInstitutionCode: US; basisOfRecord: PreservedSpecimen
- **Type status:** Other material. **Occurrence:** catalogNumber: 621; recordNumber: 24290; recordedBy: Peterson, PM; Soreng, RJ; Romaschenko, K; Mbago, F; **Taxon:** scientificName: Eragrostis; kingdom: Plantae; family: Poaceae; genus: Eragrostis; **Location:** continent: Africa; country: Tanzania; stateProvince: Mara; county: Serengeti; locality: Lobo Lodge; verbatimLocality: Serengeti National Park, on top of Lobo Ridge above Lobo Lodge.; minimumElevationInMeters: 1817; decimalLatitude: -1.99967; decimalLongitude: 35.16778; **Event:** eventDate: 2012-06-17; **Record Level:** institutionCode: US; collectionCode: Herbarium; ownerInstitutionCode: US; basisOfRecord: PreservedSpecimen
- **Type status:** Other material. **Occurrence:** catalogNumber: 622; recordNumber: 24291; recordedBy: Peterson, PM; Soreng, RJ; Romaschenko, K; Mbago, F; **Taxon:** scientificName: Eragrostis; kingdom: Plantae; family: Poaceae; genus: Eragrostis; **Location:** continent: Africa; country: Tanzania; stateProvince: Mara; county: Serengeti; locality: Lobo Lodge; verbatimLocality: Serengeti National Park, on top of Lobo Ridge above Lobo Lodge.; minimumElevationInMeters: 1817; decimalLatitude: -1.99967; decimalLongitude: 35.16778; **Event:** eventDate: 2012-06-17; **Record Level:** institutionCode: US; collectionCode: Herbarium; ownerInstitutionCode: US; basisOfRecord: PreservedSpecimen
- **Type status:** Other material. **Occurrence:** catalogNumber: 623; recordNumber: 24292; recordedBy: Peterson, PM; Soreng, RJ; Romaschenko, K; Mbago, F; **Taxon:** scientificName: Eragrostis; kingdom: Plantae; family: Poaceae; genus: Eragrostis; **Location:** continent: Africa; country: Tanzania; stateProvince: Mara; county: Serengeti; locality: Lobo Lodge; verbatimLocality: Serengeti National Park, on top of Lobo Ridge above Lobo Lodge.; minimumElevationInMeters: 1817; decimalLatitude: -1.99967; decimalLongitude: 35.16778; **Event:** eventDate: 2012-06-17; **Record Level:** institutionCode: US; collectionCode: Herbarium; ownerInstitutionCode: US; basisOfRecord: PreservedSpecimen
- **Type status:** Other material. **Occurrence:** catalogNumber: 624; recordNumber: 24302; recordedBy: Peterson, PM; Soreng, RJ; Romaschenko, K; Mbago, F; **Taxon:** scientificName: Eragrostis; kingdom: Plantae; family: Poaceae; genus: Eragrostis; **Location:** continent: Africa; country: Tanzania; stateProvince: Arusha; county: Ngorongoro; locality: Ngorongoro Crater; verbatimLocality: Ngorongoro Conservation Area, rim of Ngorongoro Crater (descent gate).; minimumElevationInMeters: 2168; decimalLatitude: -3.15462; decimalLongitude: 35.47717; **Event:** eventDate: 2012-06-19; **Record Level:** institutionCode: US; collectionCode: Herbarium; ownerInstitutionCode: US; basisOfRecord: PreservedSpecimen

#### Eragrostis aethiopica

Chiov.

##### Materials

- **Type status:** Other material. **Occurrence:** catalogNumber: K000984237; recordNumber: 388; recordedBy: Paulo, S; **Taxon:** scientificName: *Eragrostis aethiopica* Chiov.; kingdom: Plantae; family: Poaceae; genus: Eragrostis; specificEpithet: aethiopica; scientificNameAuthorship: Chiov.; **Location:** continent: Africa; country: Tanzania; stateProvince: Shinyanga; locality: Moru Kopjes; decimalLatitude: -2.75; decimalLongitude: 34.75; **Event:** eventDate: 1958-04-27; **Record Level:** institutionCode: K; collectionCode: Herbarium; ownerInstitutionCode: K; basisOfRecord: PreservedSpecimen
- **Type status:** Other material. **Occurrence:** catalogNumber: K000984238; recordNumber: 10542; recordedBy: Greenway, PJ; **Taxon:** scientificName: *Eragrostis aethiopica* Chiov.; kingdom: Plantae; family: Poaceae; genus: Eragrostis; specificEpithet: aethiopica; scientificNameAuthorship: Chiov.; **Location:** continent: Africa; country: Tanzania; stateProvince: Mara; county: Serengeti; locality: Seronera; verbatimLocality: Serengeti; minimumElevationInMeters: 1432; decimalLatitude: -2.45; decimalLongitude: 34.833333; **Event:** eventDate: 1962-03-23; **Record Level:** institutionCode: K; collectionCode: Herbarium; ownerInstitutionCode: K; basisOfRecord: PreservedSpecimen

##### Distribution

Tropical Africa & Arabia

#### Eragrostis aspera

(Jacq.) Nees

##### Materials

- **Type status:** Other material. **Occurrence:** catalogNumber: K000984227; recordNumber: 10359; recordedBy: Greenway, PJ; **Taxon:** scientificName: *Eragrostis aspera* (Jacq.) Nees; kingdom: Plantae; family: Poaceae; genus: Eragrostis; specificEpithet: aspera; scientificNameAuthorship: (Jacq.) Nees; **Location:** continent: Africa; country: Tanzania; stateProvince: Shinyanga; locality: Naabi Hill; verbatimLocality: Eastern Hillock; minimumElevationInMeters: 1676; decimalLatitude: -2.883333; decimalLongitude: 35.033333; **Event:** eventDate: 1961-06-02; **Record Level:** institutionCode: K; collectionCode: Herbarium; ownerInstitutionCode: K; basisOfRecord: PreservedSpecimen
- **Type status:** Other material. **Occurrence:** catalogNumber: K000984228; recordNumber: 10192; recordedBy: Greenway, PJ; **Taxon:** scientificName: *Eragrostis aspera* (Jacq.) Nees; kingdom: Plantae; family: Poaceae; genus: Eragrostis; specificEpithet: aspera; scientificNameAuthorship: (Jacq.) Nees; **Location:** continent: Africa; country: Tanzania; stateProvince: Mara; county: Serengeti; locality: Seronera; verbatimLocality: Serengeti; minimumElevationInMeters: 1448; decimalLatitude: -2.45; decimalLongitude: 34.833333; **Event:** eventDate: 1961-05-16; **Record Level:** institutionCode: K; collectionCode: Herbarium; ownerInstitutionCode: K; basisOfRecord: PreservedSpecimen

##### Distribution

Tropical Africa, Arabia & Asia

#### Eragrostis cilianensis

(All.) Janch.

Eragrostis polysperma Peter

##### Materials

- **Type status:** Other material. **Occurrence:** catalogNumber: K000984244; recordNumber: 13313; recordedBy: Greenway, PJ; Kanuri; Tanner, M; **Taxon:** scientificName: *Eragrostis cilianensis* (All.) Janch.; kingdom: Plantae; family: Poaceae; genus: Eragrostis; specificEpithet: cilianensis; scientificNameAuthorship: (All.) Janch.; **Location:** continent: Africa; country: Tanzania; stateProvince: Mara; county: Serengeti; locality: Kirawira Plains; minimumElevationInMeters: 1189; decimalLatitude: -2.166667; decimalLongitude: 34.15; **Event:** eventDate: 1968-02-20; **Record Level:** institutionCode: K; collectionCode: Herbarium; ownerInstitutionCode: K; basisOfRecord: PreservedSpecimen
- **Type status:** Other material. **Occurrence:** catalogNumber: K000984245; recordNumber: 10495; recordedBy: Greenway, PJ; **Taxon:** scientificName: *Eragrostis cilianensis* (All.) Janch.; kingdom: Plantae; family: Poaceae; genus: Eragrostis; specificEpithet: cilianensis; scientificNameAuthorship: (All.) Janch.; **Location:** continent: Africa; country: Tanzania; stateProvince: Mara; county: Serengeti; locality: Nyaraswiga Hill; minimumElevationInMeters: 1341; decimalLatitude: -2.366667; decimalLongitude: 34.783333; **Event:** eventDate: 1962-03-05; **Record Level:** institutionCode: K; collectionCode: Herbarium; ownerInstitutionCode: K; basisOfRecord: PreservedSpecimen
- **Type status:** Other material. **Occurrence:** catalogNumber: K000984246; recordNumber: 6392; recordedBy: Newbould, JB; **Taxon:** scientificName: *Eragrostis cilianensis* (All.) Janch.; kingdom: Plantae; family: Poaceae; genus: Eragrostis; specificEpithet: cilianensis; scientificNameAuthorship: (All.) Janch.; **Location:** continent: Africa; country: Tanzania; stateProvince: Arusha; county: Ngorongoro; locality: Olkarien; verbatimLocality: Olgarien; minimumElevationInMeters: 1371; decimalLatitude: -2.6; decimalLongitude: 35.366667; **Event:** eventDate: 1962-12-20; **Record Level:** institutionCode: K; collectionCode: Herbarium; ownerInstitutionCode: K; basisOfRecord: PreservedSpecimen
- **Type status:** Other material. **Occurrence:** catalogNumber: K000984247; recordNumber: 2799; recordedBy: Chuwa, S; **Taxon:** scientificName: *Eragrostis cilianensis* (All.) Janch.; kingdom: Plantae; family: Poaceae; genus: Eragrostis; specificEpithet: cilianensis; scientificNameAuthorship: (All.) Janch.; **Location:** continent: Africa; country: Tanzania; stateProvince: Arusha; county: Ngorongoro; locality: Ngorongoro Crater; verbatimLocality: floor; minimumElevationInMeters: 1707; decimalLatitude: -3.166667; decimalLongitude: 35.583333; **Event:** eventDate: 1989-06-01; **Record Level:** institutionCode: K; collectionCode: Herbarium; ownerInstitutionCode: K; basisOfRecord: PreservedSpecimen
- **Type status:** Other material. **Occurrence:** catalogNumber: K000984248; recordNumber: 9983; recordedBy: Greenway, PJ; **Taxon:** scientificName: *Eragrostis cilianensis* (All.) Janch.; kingdom: Plantae; family: Poaceae; genus: Eragrostis; specificEpithet: cilianensis; scientificNameAuthorship: (All.) Janch.; **Location:** continent: Africa; country: Tanzania; stateProvince: Mara; county: Serengeti; locality: Seronera; verbatimLocality: Dam site; minimumElevationInMeters: 1448; decimalLatitude: -2.433333; decimalLongitude: 34.816667; **Event:** eventDate: 1961-04-05; **Record Level:** institutionCode: K; collectionCode: Herbarium; ownerInstitutionCode: K; basisOfRecord: PreservedSpecimen
- **Type status:** Other material. **Occurrence:** catalogNumber: K000984249; recordNumber: 11353; recordedBy: Greenway, PJ; Hunter, J; **Taxon:** scientificName: *Eragrostis cilianensis* (All.) Janch.; kingdom: Plantae; family: Poaceae; genus: Eragrostis; specificEpithet: cilianensis; scientificNameAuthorship: (All.) Janch.; **Location:** continent: Africa; country: Tanzania; stateProvince: Arusha; county: Ngorongoro; locality: Ol Doinyo Lengai; verbatimLocality: In gorge N.E. Ol Doinyo Lengai; minimumElevationInMeters: 853; decimalLatitude: -2.75; decimalLongitude: 35.9; **Event:** eventDate: 1964-03-12; **Record Level:** institutionCode: K; collectionCode: Herbarium; ownerInstitutionCode: K; basisOfRecord: PreservedSpecimen
- **Type status:** Other material. **Occurrence:** catalogNumber: K000984250; recordNumber: 10504; recordedBy: Greenway, PJ; **Taxon:** scientificName: *Eragrostis cilianensis* (All.) Janch.; kingdom: Plantae; family: Poaceae; genus: Eragrostis; specificEpithet: cilianensis; scientificNameAuthorship: (All.) Janch.; **Location:** continent: Africa; country: Tanzania; stateProvince: Shinyanga; locality: Lake Magadi; verbatimLocality: South side of Lake Magadi, Mile 18 from Seronera, Serengeti; minimumElevationInMeters: 1463; decimalLatitude: -2.633333; decimalLongitude: 34.9; **Event:** eventDate: 1962-03-06; **Record Level:** institutionCode: K; collectionCode: Herbarium; ownerInstitutionCode: K; basisOfRecord: PreservedSpecimen
- **Type status:** Other material. **Occurrence:** catalogNumber: K000984251; recordNumber: 6359; recordedBy: Leippert, H; **Taxon:** scientificName: *Eragrostis cilianensis* (All.) Janch.; kingdom: Plantae; family: Poaceae; genus: Eragrostis; specificEpithet: cilianensis; scientificNameAuthorship: (All.) Janch.; **Location:** continent: Africa; country: Tanzania; stateProvince: Arusha; county: Ngorongoro; locality: Engaruka; minimumElevationInMeters: 997; decimalLatitude: -2.983333; decimalLongitude: 35.95; **Event:** eventDate: 1966-02-24; **Record Level:** institutionCode: K; collectionCode: Herbarium; ownerInstitutionCode: K; basisOfRecord: PreservedSpecimen
- **Type status:** Other material. **Occurrence:** catalogNumber: K000984252; recordNumber: 322; recordedBy: Paulo, S; **Taxon:** scientificName: *Eragrostis cilianensis* (All.) Janch.; kingdom: Plantae; family: Poaceae; genus: Eragrostis; specificEpithet: cilianensis; scientificNameAuthorship: (All.) Janch.; **Location:** continent: Africa; country: Tanzania; stateProvince: Arusha; county: Ngorongoro; locality: Olbalbal; minimumElevationInMeters: 1250; decimalLatitude: -2.95; decimalLongitude: 35.283333; **Event:** eventDate: 1958-04-20; **Record Level:** institutionCode: K; collectionCode: Herbarium; ownerInstitutionCode: K; basisOfRecord: PreservedSpecimen
- **Type status:** Isotype. **Occurrence:** catalogNumber: W0027741; recordNumber: 43114; recordedBy: Peter, A; **Taxon:** scientificName: *Eragrostis polysperma* Peter; kingdom: Plantae; family: Poaceae; genus: Eragrostis; specificEpithet: polysperma; scientificNameAuthorship: Peter; **Location:** continent: Africa; country: Tanzania; stateProvince: Arusha; county: Ngorongoro; locality: Ngorongoro Crater; verbatimLocality: Arusha: Deutsch Ost-Afrika: Landschaft Winter Hochland: Rand des Ngorongoro Kessels.; minimumElevationInMeters: 2200; decimalLatitude: -3.166667; decimalLongitude: 35.583333; **Event:** eventDate: 1926-07-22; **Record Level:** institutionCode: W; collectionCode: Herbarium; ownerInstitutionCode: W; basisOfRecord: PreservedSpecimen

##### Distribution

Widespread

#### Eragrostis exasperata

Peter

##### Materials

- **Type status:** Other material. **Occurrence:** catalogNumber: K000984229; recordNumber: 5392; recordedBy: Vesey-FitzGerald, LDEF; **Taxon:** scientificName: *Eragrostis exasperata* Peter; kingdom: Plantae; family: Poaceae; genus: Eragrostis; specificEpithet: exasperata; scientificNameAuthorship: Peter; **Location:** continent: Africa; country: Tanzania; stateProvince: Mara; county: Serengeti; locality: Lobo springs; verbatimLocality: Serengeti N.P.; decimalLatitude: -1.966667; decimalLongitude: 35.216667; **Event:** eventDate: 1967-09-01; **Record Level:** institutionCode: K; collectionCode: Herbarium; ownerInstitutionCode: K; basisOfRecord: PreservedSpecimen
- **Type status:** Other material. **Occurrence:** catalogNumber: K000984230; recordNumber: 10388; recordedBy: Greenway, PJ; **Taxon:** scientificName: *Eragrostis exasperata* Peter; kingdom: Plantae; family: Poaceae; genus: Eragrostis; specificEpithet: exasperata; scientificNameAuthorship: Peter; **Location:** continent: Africa; country: Tanzania; stateProvince: Mara; county: Serengeti; locality: Tabora; minimumElevationInMeters: 1554; decimalLatitude: -1.816667; decimalLongitude: 34.633333; **Event:** eventDate: 1961-06-22; **Record Level:** institutionCode: K; collectionCode: Herbarium; ownerInstitutionCode: K; basisOfRecord: PreservedSpecimen
- **Type status:** Other material. **Occurrence:** catalogNumber: K000984231; recordNumber: 9952; recordedBy: Greenway, PJ; **Taxon:** scientificName: *Eragrostis exasperata* Peter; kingdom: Plantae; family: Poaceae; genus: Eragrostis; specificEpithet: exasperata; scientificNameAuthorship: Peter; **Location:** continent: Africa; country: Tanzania; stateProvince: Mara; county: Serengeti; locality: Seronera; minimumElevationInMeters: 1554; decimalLatitude: -2.45; decimalLongitude: 34.833333; **Event:** eventDate: 1961-04-02; **Record Level:** institutionCode: K; collectionCode: Herbarium; ownerInstitutionCode: K; basisOfRecord: PreservedSpecimen

##### Distribution

Tropical Africa

#### Eragrostis heteromera

Stapf

##### Materials

- **Type status:** Other material. **Occurrence:** catalogNumber: K000984232; recordNumber: 9840; recordedBy: Greenway, PJ; **Taxon:** scientificName: *Eragrostis heteromera* Stapf; kingdom: Plantae; family: Poaceae; genus: Eragrostis; specificEpithet: heteromera; scientificNameAuthorship: Stapf; **Location:** continent: Africa; country: Tanzania; stateProvince: Mara; county: Serengeti; locality: Seronera; minimumElevationInMeters: 1554; decimalLatitude: -2.45; decimalLongitude: 34.833333; **Event:** eventDate: 1961-03-17; **Record Level:** institutionCode: K; collectionCode: Herbarium; ownerInstitutionCode: K; basisOfRecord: PreservedSpecimen
- **Type status:** Other material. **Occurrence:** catalogNumber: K000984233; recordNumber: 13330; recordedBy: Greenway, PJ; Kanuri; Tanner, M; **Taxon:** scientificName: *Eragrostis heteromera* Stapf; kingdom: Plantae; family: Poaceae; genus: Eragrostis; specificEpithet: heteromera; scientificNameAuthorship: Stapf; **Location:** continent: Africa; country: Tanzania; stateProvince: Mara; county: Serengeti; locality: Ndabaka Guard Post; minimumElevationInMeters: 1128; decimalLatitude: -2.166667; decimalLongitude: 34.883333; **Event:** eventDate: 1968-02-22; **Record Level:** institutionCode: K; collectionCode: Herbarium; ownerInstitutionCode: K; basisOfRecord: PreservedSpecimen
- **Type status:** Other material. **Occurrence:** catalogNumber: K000984234; recordNumber: 37; recordedBy: Brooks, GP; **Taxon:** scientificName: *Eragrostis heteromera* Stapf; kingdom: Plantae; family: Poaceae; genus: Eragrostis; specificEpithet: heteromera; scientificNameAuthorship: Stapf; **Location:** continent: Africa; country: Tanzania; stateProvince: Mara; county: Serengeti; locality: Ikoma; minimumElevationInMeters: 1371; decimalLatitude: -2.066667; decimalLongitude: 34.616667; **Event:** eventDate: 1954-02-19; **Record Level:** institutionCode: K; collectionCode: Herbarium; ownerInstitutionCode: K; basisOfRecord: PreservedSpecimen
- **Type status:** Other material. **Occurrence:** catalogNumber: K000984235; recordNumber: 228; recordedBy: Braun, HMH; **Taxon:** scientificName: *Eragrostis heteromera* Stapf; kingdom: Plantae; family: Poaceae; genus: Eragrostis; specificEpithet: heteromera; scientificNameAuthorship: Stapf; **Location:** continent: Africa; country: Tanzania; stateProvince: Mara; county: Serengeti; locality: Simba Kopjes; verbatimLocality: Excavation 6 km north of Simba kopjes; minimumElevationInMeters: 1500; decimalLatitude: -2.633333; decimalLongitude: 34.916667; **Event:** eventDate: 1967-05-23; **Record Level:** institutionCode: K; collectionCode: Herbarium; ownerInstitutionCode: K; basisOfRecord: PreservedSpecimen
- **Type status:** Other material. **Occurrence:** catalogNumber: 614; recordNumber: s.n.; recordedBy: Mboya, E; **Taxon:** scientificName: *Eragrostis heteromera* Stapf; kingdom: Plantae; family: Poaceae; genus: Eragrostis; specificEpithet: heteromera; scientificNameAuthorship: Stapf; **Location:** continent: Africa; country: Tanzania; stateProvince: Mara; county: Serengeti; locality: Serengeti National Park; verbatimLocality: T1. Serengeti National Park. Ndabaka-Seronera Road.; minimumElevationInMeters: 1417; decimalLatitude: -2.23; decimalLongitude: 34.73; **Event:** eventDate: 2004-02-07; **Record Level:** institutionCode: MO; collectionCode: Herbarium; ownerInstitutionCode: MO; basisOfRecord: PreservedSpecimen

##### Distribution

Tropical Africa

#### Eragrostis hispida

K.Schum.

##### Materials

- **Type status:** Other material. **Occurrence:** catalogNumber: K000984226; recordNumber: 9996; recordedBy: Greenway, PJ; Tanner, M; **Taxon:** scientificName: *Eragrostis hispida* K.Schum.; kingdom: Plantae; family: Poaceae; genus: Eragrostis; specificEpithet: hispida; scientificNameAuthorship: K.Schum.; **Location:** continent: Africa; country: Tanzania; stateProvince: Mara; county: Serengeti; locality: Seronera; verbatimLocality: Seronera to Kleins Camp, Mile 57; minimumElevationInMeters: 1646; decimalLatitude: -1.85; decimalLongitude: 35.416667; **Event:** eventDate: 1961-04-06; **Record Level:** institutionCode: K; collectionCode: Herbarium; ownerInstitutionCode: K; basisOfRecord: PreservedSpecimen
- **Type status:** Other material. **Occurrence:** catalogNumber: K001087157; recordNumber: 10939; recordedBy: Greenway, PJ; Turner; **Taxon:** scientificName: *Eragrostis hispida* K.Schum.; kingdom: Plantae; family: Poaceae; genus: Eragrostis; specificEpithet: hispida; scientificNameAuthorship: K.Schum.; **Location:** continent: Africa; country: Tanzania; stateProvince: Arusha; county: Ngorongoro; locality: Klein's camp; verbatimLocality: Between Campi ya Mpofu and Klein's camp; minimumElevationInMeters: 1554; decimalLatitude: -1.7; decimalLongitude: 35.216667; **Event:** eventDate: 1963-01-14; **Record Level:** institutionCode: K; collectionCode: Herbarium; ownerInstitutionCode: K; basisOfRecord: PreservedSpecimen

##### Distribution

Tropical Africa

#### Eragrostis humidicola

Napper

##### Materials

- **Type status:** Other material. **Occurrence:** catalogNumber: K001087220; recordNumber: 10134; recordedBy: Greenway, PJ; **Taxon:** scientificName: *Eragrostis humidicola* Napper; kingdom: Plantae; family: Poaceae; genus: Eragrostis; specificEpithet: humidicola; scientificNameAuthorship: Napper; **Location:** continent: Africa; country: Tanzania; stateProvince: Mara; county: Serengeti; locality: Tabora; verbatimLocality: Mile 46.6 from the Bologonja river via Klein's camp.; minimumElevationInMeters: 1615; decimalLatitude: -1.816667; decimalLongitude: 34.633333; **Event:** eventDate: 1961-05-01; **Record Level:** institutionCode: K; collectionCode: Herbarium; ownerInstitutionCode: K; basisOfRecord: PreservedSpecimen

##### Distribution

Kenya, Tanzania, Uganda, Democratic Republic of Congo & Rwanda

#### Eragrostis papposa

(Roem. & Schult.) Steud.

Eragrostis aulacosperma (Fresen.) Steud.

##### Materials

- **Type status:** Other material. **Occurrence:** catalogNumber: K000984253; recordNumber: 6374; recordedBy: Newbould, JB; **Taxon:** scientificName: *Eragrostis papposa* (Roem. & Schult.) Steud.; kingdom: Plantae; family: Poaceae; genus: Eragrostis; specificEpithet: papposa; scientificNameAuthorship: (Roem. & Schult.) Steud.; **Location:** continent: Africa; country: Tanzania; stateProvince: Arusha; county: Ngorongoro; locality: Olbalbal; verbatimLocality: Olbalbal, Olbaata; minimumElevationInMeters: 1371; decimalLatitude: -2.95; decimalLongitude: 35.283333; **Event:** eventDate: 1962-12-14; **Record Level:** institutionCode: K; collectionCode: Herbarium; ownerInstitutionCode: K; basisOfRecord: PreservedSpecimen
- **Type status:** Other material. **Occurrence:** catalogNumber: 298; recordNumber: 99; recordedBy: Kreulen, AR; **Taxon:** scientificName: *Eragrostis papposa* (Roem. & Schult.) Steud.; kingdom: Plantae; family: Poaceae; genus: Eragrostis; specificEpithet: papposa; scientificNameAuthorship: (Roem. & Schult.) Steud.; **Location:** continent: Africa; country: Tanzania; stateProvince: Mara; county: Serengeti; locality: Serengeti National Park; verbatimLocality: SE part of park.; decimalLatitude: -2.333333; decimalLongitude: 34.833333; **Event:** eventDate: 1972-11-21; **Record Level:** institutionCode: EA; collectionCode: Herbarium; ownerInstitutionCode: EA; basisOfRecord: PreservedSpecimen
- **Type status:** Other material. **Occurrence:** catalogNumber: 299; recordNumber: 4687; recordedBy: Vesey-FitzGerald, LDEF; **Taxon:** scientificName: *Eragrostis papposa* (Roem. & Schult.) Steud.; kingdom: Plantae; family: Poaceae; genus: Eragrostis; specificEpithet: papposa; scientificNameAuthorship: (Roem. & Schult.) Steud.; **Location:** continent: Africa; country: Tanzania; stateProvince: Arusha; county: Ngorongoro; locality: Olduvai Gorge; verbatimLocality: Serengeti.; minimumElevationInMeters: 1524; decimalLatitude: -2.95; decimalLongitude: 35.166667; **Event:** eventDate: 1965-05-16; **Record Level:** institutionCode: EA; collectionCode: Herbarium; ownerInstitutionCode: EA; basisOfRecord: PreservedSpecimen
- **Type status:** Other material. **Occurrence:** catalogNumber: 790; recordNumber: 5991; recordedBy: Newbould, JB; **Taxon:** scientificName: *Eragrostis papposa* (Roem. & Schult.) Steud.; kingdom: Plantae; family: Poaceae; genus: Eragrostis; specificEpithet: papposa; scientificNameAuthorship: (Roem. & Schult.) Steud.; **Location:** continent: Africa; country: Tanzania; stateProvince: Arusha; county: Ngorongoro; locality: Olbalbal; verbatimLocality: Depression. Olbaata; minimumElevationInMeters: 1371; **Event:** eventDate: 1962-02-07; **Record Level:** institutionCode: EA; collectionCode: Herbarium; ownerInstitutionCode: EA; basisOfRecord: PreservedSpecimen
- **Type status:** Other material. **Occurrence:** catalogNumber: 791; recordNumber: 5934; recordedBy: Newbould, JB; **Taxon:** scientificName: *Eragrostis papposa* (Roem. & Schult.) Steud.; kingdom: Plantae; family: Poaceae; genus: Eragrostis; specificEpithet: papposa; scientificNameAuthorship: (Roem. & Schult.) Steud.; **Location:** continent: Africa; country: Tanzania; stateProvince: Arusha; county: Ngorongoro; locality: Olbalbal; verbatimLocality: Longogo.; minimumElevationInMeters: 1417; **Event:** eventDate: 1962-01-30; **Record Level:** institutionCode: EA; collectionCode: Herbarium; ownerInstitutionCode: EA; basisOfRecord: PreservedSpecimen
- **Type status:** Other material. **Occurrence:** catalogNumber: 794; recordNumber: 256; recordedBy: Braun, HMH; **Taxon:** scientificName: *Eragrostis papposa* (Roem. & Schult.) Steud.; kingdom: Plantae; family: Poaceae; genus: Eragrostis; specificEpithet: papposa; scientificNameAuthorship: (Roem. & Schult.) Steud.; **Location:** continent: Africa; country: Tanzania; stateProvince: Shinyanga; locality: Naabi Hill; verbatimLocality: 10km to the SE.; minimumElevationInMeters: 1500; decimalLatitude: -2.933333; decimalLongitude: 35.083333; **Event:** eventDate: 1968-02-27; **Record Level:** institutionCode: EA; collectionCode: Herbarium; ownerInstitutionCode: EA; basisOfRecord: PreservedSpecimen
- **Type status:** Other material. **Occurrence:** catalogNumber: 795; recordNumber: 274; recordedBy: Braun, HMH; **Taxon:** scientificName: *Eragrostis papposa* (Roem. & Schult.) Steud.; kingdom: Plantae; family: Poaceae; genus: Eragrostis; specificEpithet: papposa; scientificNameAuthorship: (Roem. & Schult.) Steud.; **Location:** continent: Africa; country: Tanzania; stateProvince: Shinyanga; locality: Naabi Hill; verbatimLocality: Northern slope of Naabi Hill.; minimumElevationInMeters: 1500; decimalLatitude: -2.883333; decimalLongitude: 35.083333; **Event:** eventDate: 1968-03-29; **Record Level:** institutionCode: EA; collectionCode: Herbarium; ownerInstitutionCode: EA; basisOfRecord: PreservedSpecimen
- **Type status:** Other material. **Occurrence:** catalogNumber: 816; recordNumber: 6374; recordedBy: Newbould, JB; **Taxon:** scientificName: *Eragrostis papposa* (Roem. & Schult.) Steud.; kingdom: Plantae; family: Poaceae; genus: Eragrostis; specificEpithet: papposa; scientificNameAuthorship: (Roem. & Schult.) Steud.; **Location:** continent: Africa; country: Tanzania; stateProvince: Arusha; county: Ngorongoro; locality: Olbalbal; verbatimLocality: Olbalbal, Olbaata; minimumElevationInMeters: 1371; decimalLatitude: -2.95; decimalLongitude: 35.283333; **Event:** eventDate: 1962-12-14; **Record Level:** institutionCode: EA; collectionCode: Herbarium; ownerInstitutionCode: EA; basisOfRecord: PreservedSpecimen
- **Type status:** Other material. **Occurrence:** catalogNumber: 823; recordNumber: 1675; recordedBy: Heady, HF; **Taxon:** scientificName: *Eragrostis papposa* (Roem. & Schult.) Steud.; kingdom: Plantae; family: Poaceae; genus: Eragrostis; specificEpithet: papposa; scientificNameAuthorship: (Roem. & Schult.) Steud.; **Location:** continent: Africa; country: Tanzania; stateProvince: Arusha; county: Ngorongoro; locality: Ngorongoro; decimalLatitude: -3; decimalLongitude: 35.5; **Event:** eventDate: 1959-02-19; **Record Level:** institutionCode: EA; collectionCode: Herbarium; ownerInstitutionCode: EA; basisOfRecord: PreservedSpecimen
- **Type status:** Other material. **Occurrence:** catalogNumber: 1010; recordNumber: 1695; recordedBy: Heady, AF; **Taxon:** scientificName: *Eragrostis papposa* (Roem. & Schult.) Steud.; kingdom: Plantae; family: Poaceae; genus: Eragrostis; specificEpithet: papposa; scientificNameAuthorship: (Roem. & Schult.) Steud.; **Location:** continent: Africa; country: Tanzania; stateProvince: Arusha; county: Ngorongoro; locality: Ngorongoro Crater; decimalLatitude: -3.166667; decimalLongitude: 35.583333; **Event:** eventDate: 1959-02-19; **Record Level:** institutionCode: EA; collectionCode: Herbarium; ownerInstitutionCode: EA; basisOfRecord: PreservedSpecimen

##### Distribution

Africa & Asia

#### Eragrostis pilosa

(L.) P.Beauv.

##### Materials

- **Type status:** Other material. **Occurrence:** catalogNumber: K000984236; recordNumber: 395; recordedBy: Paulo, S; **Taxon:** scientificName: *Eragrostis pilosa* (L.) P.Beauv.; kingdom: Plantae; family: Poaceae; genus: Eragrostis; specificEpithet: pilosa; scientificNameAuthorship: (L.) P.Beauv.; **Location:** continent: Africa; country: Tanzania; stateProvince: Mara; county: Serengeti; locality: Nyaraswiga Hill; decimalLatitude: -2.366667; decimalLongitude: 34.783333; **Event:** eventDate: 1958-04-27; **Record Level:** institutionCode: K; collectionCode: Herbarium; ownerInstitutionCode: K; basisOfRecord: PreservedSpecimen
- **Type status:** Other material. **Occurrence:** catalogNumber: K001087156; recordNumber: 9875; recordedBy: Greenway, PJ; **Taxon:** scientificName: *Eragrostis pilosa* (L.) P.Beauv.; kingdom: Plantae; family: Poaceae; genus: Eragrostis; specificEpithet: pilosa; scientificNameAuthorship: (L.) P.Beauv.; **Location:** continent: Africa; country: Tanzania; stateProvince: Mara; county: Serengeti; locality: Seronera; minimumElevationInMeters: 1554; decimalLatitude: -2.45; decimalLongitude: 34.833333; **Event:** eventDate: 1961-03-21; **Record Level:** institutionCode: K; collectionCode: Herbarium; ownerInstitutionCode: K; basisOfRecord: PreservedSpecimen

##### Distribution

Widespread

#### Eragrostis racemosa

(Thunb.) Steud.

##### Materials

- **Type status:** Other material. **Occurrence:** catalogNumber: K000984241; recordNumber: 10302; recordedBy: Greenway, PJ; **Taxon:** scientificName: *Eragrostis racemosa* (Thunb.) Steud.; kingdom: Plantae; family: Poaceae; genus: Eragrostis; specificEpithet: racemosa; scientificNameAuthorship: (Thunb.) Steud.; **Location:** continent: Africa; country: Tanzania; stateProvince: Mara; county: Serengeti; locality: Seronera; verbatimLocality: Seronera to Klein's Camp, Mile 55.3; minimumElevationInMeters: 1798; decimalLatitude: -1.866667; decimalLongitude: 35.4; **Event:** eventDate: 1961-05-17; **Record Level:** institutionCode: K; collectionCode: Herbarium; ownerInstitutionCode: K; basisOfRecord: PreservedSpecimen
- **Type status:** Other material. **Occurrence:** catalogNumber: K000984242; recordNumber: 13318; recordedBy: Greenway, PJ; Kanuri; Tanner, M; **Taxon:** scientificName: *Eragrostis racemosa* (Thunb.) Steud.; kingdom: Plantae; family: Poaceae; genus: Eragrostis; specificEpithet: racemosa; scientificNameAuthorship: (Thunb.) Steud.; **Location:** continent: Africa; country: Tanzania; stateProvince: Shinyanga; locality: Handajenga; minimumElevationInMeters: 1219; decimalLatitude: -2.25; decimalLongitude: 34.1; **Event:** eventDate: 1968-02-21; **Record Level:** institutionCode: K; collectionCode: Herbarium; ownerInstitutionCode: K; basisOfRecord: PreservedSpecimen
- **Type status:** Other material. **Occurrence:** catalogNumber: K000984243; recordNumber: 10130; recordedBy: Greenway, PJ; **Taxon:** scientificName: *Eragrostis racemosa* (Thunb.) Steud.; kingdom: Plantae; family: Poaceae; genus: Eragrostis; specificEpithet: racemosa; scientificNameAuthorship: (Thunb.) Steud.; **Location:** continent: Africa; country: Tanzania; stateProvince: Mara; county: Serengeti; locality: Tabora; verbatimLocality: Mile 46.6 from Bolgonja river via Kleins Camp; minimumElevationInMeters: 1615; decimalLatitude: -1.816667; decimalLongitude: 34.633333; **Event:** eventDate: 1961-04-30; **Record Level:** institutionCode: K; collectionCode: Herbarium; ownerInstitutionCode: K; basisOfRecord: PreservedSpecimen
- **Type status:** Other material. **Occurrence:** catalogNumber: 615; recordNumber: 776; recordedBy: Ellemann, L; **Taxon:** scientificName: *Eragrostis racemosa* (Thunb.) Steud.; kingdom: Plantae; family: Poaceae; genus: Eragrostis; specificEpithet: racemosa; scientificNameAuthorship: (Thunb.) Steud.; **Location:** continent: Africa; country: Tanzania; stateProvince: Arusha; county: Ngorongoro; locality: Endulen; verbatimLocality: Ngorongoro Conservation Area 3 km south of Endulen Village.; minimumElevationInMeters: 2000; decimalLatitude: -3.2; decimalLongitude: 35.283; **Event:** eventDate: 1993-08-06; **Record Level:** institutionCode: AAU; collectionCode: Herbarium; ownerInstitutionCode: AAU; basisOfRecord: PreservedSpecimen

##### Distribution

Tropical Africa

#### Eragrostis schweinfurthii

Chiov.

##### Materials

- **Type status:** Other material. **Occurrence:** catalogNumber: K000984239; recordNumber: 908; recordedBy: Pole Evans, IB; Erens, J; **Taxon:** scientificName: *Eragrostis schweinfurthii* Chiov.; kingdom: Plantae; family: Poaceae; genus: Eragrostis; specificEpithet: schweinfurthii; scientificNameAuthorship: Chiov.; **Location:** continent: Africa; country: Tanzania; stateProvince: Arusha; county: Ngorongoro; locality: Ngorongoro Crater; verbatimLocality: on top of the Ngorongoro crater ridge; decimalLatitude: -3.166667; decimalLongitude: 35.583333; **Event:** eventDate: 1938-06-24; **Record Level:** institutionCode: K; collectionCode: Herbarium; ownerInstitutionCode: K; basisOfRecord: PreservedSpecimen
- **Type status:** Other material. **Occurrence:** catalogNumber: K000984240; recordNumber: 2751; recordedBy: Chuwa, S; **Taxon:** scientificName: *Eragrostis schweinfurthii* Chiov.; kingdom: Plantae; family: Poaceae; genus: Eragrostis; specificEpithet: schweinfurthii; scientificNameAuthorship: Chiov.; **Location:** continent: Africa; country: Tanzania; stateProvince: Arusha; county: Ngorongoro; locality: Mokilal; verbatimLocality: north east of Oldean Mtn.; minimumElevationInMeters: 2378; decimalLatitude: -3.266667; decimalLongitude: 35.433333; **Event:** eventDate: 1989-05-25; **Record Level:** institutionCode: K; collectionCode: Herbarium; ownerInstitutionCode: K; basisOfRecord: PreservedSpecimen

##### Distribution

Tropical Africa & Arabia

#### Eragrostis tenuifolia

(A.Rich.) Hochst. ex Steud.

##### Materials

- **Type status:** Other material. **Occurrence:** catalogNumber: K000984254; recordNumber: 2751b; recordedBy: Chuwa, S; **Taxon:** scientificName: *Eragrostis tenuifolia* (A.Rich.) Hochst. ex Steud.; kingdom: Plantae; family: Poaceae; genus: Eragrostis; specificEpithet: tenuifolia; scientificNameAuthorship: (A.Rich.) Hochst. ex Steud.; **Location:** continent: Africa; country: Tanzania; stateProvince: Arusha; county: Ngorongoro; locality: Mokilal; verbatimLocality: N. E. of Oldean Mountain; minimumElevationInMeters: 2378; decimalLatitude: -3.266667; decimalLongitude: 35.433333; **Event:** eventDate: 1989-05-25; **Record Level:** institutionCode: K; collectionCode: Herbarium; ownerInstitutionCode: K; basisOfRecord: PreservedSpecimen
- **Type status:** Other material. **Occurrence:** catalogNumber: K000984255; recordNumber: 90; recordedBy: Frame, GW; **Taxon:** scientificName: *Eragrostis tenuifolia* (A.Rich.) Hochst. ex Steud.; kingdom: Plantae; family: Poaceae; genus: Eragrostis; specificEpithet: tenuifolia; scientificNameAuthorship: (A.Rich.) Hochst. ex Steud.; **Location:** continent: Africa; country: Tanzania; stateProvince: Arusha; county: Ngorongoro; locality: Empakai Crater; verbatimLocality: top of east rim; minimumElevationInMeters: 2600; decimalLatitude: -2.933333; decimalLongitude: 35.816667; **Event:** eventDate: 1973-03-14; **Record Level:** institutionCode: K; collectionCode: Herbarium; ownerInstitutionCode: K; basisOfRecord: PreservedSpecimen
- **Type status:** Other material. **Occurrence:** catalogNumber: K000984256; recordNumber: 25130; recordedBy: Pole Evans, IB; Erens, J; **Taxon:** scientificName: *Eragrostis tenuifolia* (A.Rich.) Hochst. ex Steud.; kingdom: Plantae; family: Poaceae; genus: Eragrostis; specificEpithet: tenuifolia; scientificNameAuthorship: (A.Rich.) Hochst. ex Steud.; **Location:** continent: Africa; country: Tanzania; stateProvince: Arusha; county: Ngorongoro; locality: Ngorongoro Crater; decimalLatitude: -3.166667; decimalLongitude: 35.583333; **Event:** eventDate: 1938-06-24; **Record Level:** institutionCode: K; collectionCode: Herbarium; ownerInstitutionCode: K; basisOfRecord: PreservedSpecimen
- **Type status:** Other material. **Occurrence:** catalogNumber: K000984257; recordNumber: 72; recordedBy: Brooks, GP; **Taxon:** scientificName: *Eragrostis tenuifolia* (A.Rich.) Hochst. ex Steud.; kingdom: Plantae; family: Poaceae; genus: Eragrostis; specificEpithet: tenuifolia; scientificNameAuthorship: (A.Rich.) Hochst. ex Steud.; **Location:** continent: Africa; country: Tanzania; stateProvince: Mara; county: Serengeti; locality: Banagi Hill; minimumElevationInMeters: 1463; decimalLatitude: -2.3; decimalLongitude: 34.833333; **Event:** eventDate: 1954-02-08; **Record Level:** institutionCode: K; collectionCode: Herbarium; ownerInstitutionCode: K; basisOfRecord: PreservedSpecimen
- **Type status:** Other material. **Occurrence:** catalogNumber: K000984258; recordNumber: 9847; recordedBy: Greenway, PJ; **Taxon:** scientificName: *Eragrostis tenuifolia* (A.Rich.) Hochst. ex Steud.; kingdom: Plantae; family: Poaceae; genus: Eragrostis; specificEpithet: tenuifolia; scientificNameAuthorship: (A.Rich.) Hochst. ex Steud.; **Location:** continent: Africa; country: Tanzania; stateProvince: Mara; county: Serengeti; locality: Seronera; verbatimLocality: Serengeti; minimumElevationInMeters: 1554; decimalLatitude: -2.45; decimalLongitude: 34.833333; **Event:** eventDate: 1961-03-18; **Record Level:** institutionCode: K; collectionCode: Herbarium; ownerInstitutionCode: K; basisOfRecord: PreservedSpecimen
- **Type status:** Other material. **Occurrence:** catalogNumber: 616; recordNumber: 717; recordedBy: Ellemann, L; **Taxon:** scientificName: *Eragrostis tenuifolia* (A.Rich.) Hochst. ex Steud.; kingdom: Plantae; family: Poaceae; genus: Eragrostis; specificEpithet: tenuifolia; scientificNameAuthorship: (A.Rich.) Hochst. ex Steud.; **Location:** continent: Africa; country: Tanzania; stateProvince: Arusha; county: Ngorongoro; locality: Sendui; verbatimLocality: Ngorongoro Conservation Area, Sendui, close to ole Senguyans boma.; minimumElevationInMeters: 2650; decimalLatitude: -2.916; decimalLongitude: 35.716; **Event:** eventDate: 1993-07-22; **Record Level:** institutionCode: AAU; collectionCode: Herbarium; ownerInstitutionCode: AAU; basisOfRecord: PreservedSpecimen
- **Type status:** Other material. **Occurrence:** catalogNumber: 1094; recordNumber: 90; recordedBy: Frame, GW; **Taxon:** scientificName: *Eragrostis tenuifolia* (A.Rich.) Hochst. ex Steud.; kingdom: Plantae; family: Poaceae; genus: Eragrostis; specificEpithet: tenuifolia; scientificNameAuthorship: (A.Rich.) Hochst. ex Steud.; **Location:** continent: Africa; country: Tanzania; stateProvince: Arusha; county: Ngorongoro; locality: Empakai Crater; verbatimLocality: top of east rim; minimumElevationInMeters: 2600; decimalLatitude: -2.933333; decimalLongitude: 35.816667; **Event:** eventDate: 1973-03-14; **Record Level:** institutionCode: NHT; collectionCode: Herbarium; ownerInstitutionCode: NHT; basisOfRecord: PreservedSpecimen

##### Distribution

Tropical Africa

#### Eragrostis viscosa

Trin.

##### Materials

- **Type status:** Other material. **Occurrence:** catalogNumber: K001087158; recordNumber: 10703; recordedBy: Greenway, PJ; Turner; **Taxon:** scientificName: *Eragrostis viscosa* Trin.; kingdom: Plantae; family: Poaceae; genus: Eragrostis; specificEpithet: viscosa; scientificNameAuthorship: Trin.; **Location:** continent: Africa; country: Tanzania; stateProvince: Mara; county: Serengeti; locality: Engare Nanyuki springs; verbatimLocality: Eastern boundary of Serengeti National Park, Engare Nanyuki (Nyanuki) Springs.; minimumElevationInMeters: 1615; decimalLatitude: -2.616667; decimalLongitude: 35.216667; **Event:** eventDate: 1962-06-05; **Record Level:** institutionCode: K; collectionCode: Herbarium; ownerInstitutionCode: K; basisOfRecord: PreservedSpecimen
- **Type status:** Other material. **Occurrence:** catalogNumber: K001087159; recordNumber: 4028; recordedBy: Verdcourt, B; **Taxon:** scientificName: *Eragrostis viscosa* Trin.; kingdom: Plantae; family: Poaceae; genus: Eragrostis; specificEpithet: viscosa; scientificNameAuthorship: Trin.; **Location:** continent: Africa; country: Tanzania; stateProvince: Arusha; county: Ngorongoro; locality: Olduvai Gorge; verbatimLocality: At junction of Olduvai to Loliondo roads and Bangi-Seronera old road, just north of Olduvai gorge.; minimumElevationInMeters: 1524; decimalLatitude: -2.95; decimalLongitude: 35.166667; **Event:** eventDate: 1964-04-02; **Record Level:** institutionCode: K; collectionCode: Herbarium; ownerInstitutionCode: K; basisOfRecord: PreservedSpecimen

##### Distribution

Tropical Africa, Asia & Arabia

#### Eriochloa sp.

##### Materials

- **Type status:** Other material. **Occurrence:** catalogNumber: 626; recordNumber: s.n.; recordedBy: Mboya, E; **Taxon:** scientificName: Eriochloa; kingdom: Plantae; family: Poaceae; genus: Eriochloa; **Location:** continent: Africa; country: Tanzania; stateProvince: Mara; county: Serengeti; locality: Serengeti National Park; verbatimLocality: T1. Serengeti National Park. Ndabaka-Seronera Road.; minimumElevationInMeters: 1297; decimalLatitude: -2.27; decimalLongitude: 34.58; **Event:** eventDate: 2004-03-09; **Record Level:** institutionCode: MO; collectionCode: Herbarium; ownerInstitutionCode: MO; basisOfRecord: PreservedSpecimen

#### Eriochloa fatmensis

(Hochst. & Steud.) Clayton

Eriochloa nubica (Steud.) Hack. & Stapf ex Thell.

##### Materials

- **Type status:** Other material. **Occurrence:** catalogNumber: 625; recordNumber: 371; recordedBy: Ellemann, L; **Taxon:** scientificName: *Eriochloa fatmensis* (Hochst. & Steud.) Clayton; kingdom: Plantae; family: Poaceae; genus: Eriochloa; specificEpithet: fatmensis; scientificNameAuthorship: (Hochst. & Steud.) Clayton; **Location:** continent: Africa; country: Tanzania; stateProvince: Arusha; county: Ngorongoro; locality: Endulen; verbatimLocality: Ngorongoro Conservation Area. Endulen Hospital.; minimumElevationInMeters: 1900; decimalLatitude: -3.2; decimalLongitude: 35.266; **Event:** eventDate: 1993-04-20; **Record Level:** institutionCode: AAU; collectionCode: Herbarium; ownerInstitutionCode: AAU; basisOfRecord: PreservedSpecimen
- **Type status:** Other material. **Occurrence:** catalogNumber: 1136; recordNumber: 899; recordedBy: Mollel, NP; Rusch, GM; Mwakalebe, G; **Taxon:** scientificName: *Eriochloa fatmensis* (Hochst. & Steud.) Clayton; kingdom: Plantae; family: Poaceae; genus: Eriochloa; specificEpithet: fatmensis; scientificNameAuthorship: (Hochst. & Steud.) Clayton; **Location:** continent: Africa; country: Tanzania; stateProvince: Mara; county: Serengeti; locality: Serengeti NP; verbatimLocality: Along the road to Nabaka gate, 36M, 068713, 9750232 UTM.; minimumElevationInMeters: 1314; decimalLatitude: -2.25; decimalLongitude: 34.333333; **Event:** eventDate: 2003-01-31; **Record Level:** institutionCode: NHT; collectionCode: Herbarium; ownerInstitutionCode: NHT; basisOfRecord: PreservedSpecimen
- **Type status:** Other material. **Occurrence:** catalogNumber: 1137; recordNumber: 839; recordedBy: Mollel, NP; Rusch, GM; Mwakalebe, G; **Taxon:** scientificName: *Eriochloa fatmensis* (Hochst. & Steud.) Clayton; kingdom: Plantae; family: Poaceae; genus: Eriochloa; specificEpithet: fatmensis; scientificNameAuthorship: (Hochst. & Steud.) Clayton; **Location:** continent: Africa; country: Tanzania; stateProvince: Mara; county: Serengeti; locality: Robanda village; verbatimLocality: Serengeti district, Robanda village, road to the water pool, CG01 plot, 36M, 0386088; 9763754 UTM.; minimumElevationInMeters: 1388; decimalLatitude: -2.083333; decimalLongitude: 34.666667; **Event:** eventDate: 2003-01-28; **Record Level:** institutionCode: NHT; collectionCode: Herbarium; ownerInstitutionCode: NHT; basisOfRecord: PreservedSpecimen
- **Type status:** Other material. **Occurrence:** catalogNumber: K000765974; recordNumber: 6515; recordedBy: Newbould, JB; **Taxon:** scientificName: *Eriochloa nubica* (Steud.) Hack. & Stapf ex Thell.; kingdom: Plantae; family: Poaceae; genus: Eriochloa; specificEpithet: nubica; scientificNameAuthorship: (Steud.) Hack. & Stapf ex Thell.; **Location:** continent: Africa; country: Tanzania; stateProvince: Arusha; county: Ngorongoro; locality: Naibardad Hill; verbatimLocality: Eluwanit Dam, near Naibardad, South East Serengeti.; minimumElevationInMeters: 1753; decimalLatitude: -3.116667; decimalLongitude: 35.1; **Event:** eventDate: 1965-1; **Record Level:** institutionCode: K; collectionCode: Herbarium; ownerInstitutionCode: K; basisOfRecord: PreservedSpecimen
- **Type status:** Other material. **Occurrence:** catalogNumber: K000765975; recordNumber: 319; recordedBy: Paulo, S; **Taxon:** scientificName: *Eriochloa nubica* (Steud.) Hack. & Stapf ex Thell.; kingdom: Plantae; family: Poaceae; genus: Eriochloa; specificEpithet: nubica; scientificNameAuthorship: (Steud.) Hack. & Stapf ex Thell.; **Location:** continent: Africa; country: Tanzania; stateProvince: Arusha; county: Ngorongoro; locality: Ngorongoro Crater; minimumElevationInMeters: 1676; decimalLatitude: -3.166667; decimalLongitude: 35.583333; **Event:** eventDate: 1958-04-19; **Record Level:** institutionCode: K; collectionCode: Herbarium; ownerInstitutionCode: K; basisOfRecord: PreservedSpecimen
- **Type status:** Other material. **Occurrence:** catalogNumber: K000765979; recordNumber: 9861; recordedBy: Greenway, PJ; **Taxon:** scientificName: *Eriochloa nubica* (Steud.) Hack. & Stapf ex Thell.; kingdom: Plantae; family: Poaceae; genus: Eriochloa; specificEpithet: nubica; scientificNameAuthorship: (Steud.) Hack. & Stapf ex Thell.; **Location:** continent: Africa; country: Tanzania; stateProvince: Mara; county: Serengeti; locality: Seronera; minimumElevationInMeters: 1554; decimalLatitude: -2.45; decimalLongitude: 34.833333; **Event:** eventDate: 1961-03-20; **Record Level:** institutionCode: K; collectionCode: Herbarium; ownerInstitutionCode: K; basisOfRecord: PreservedSpecimen
- **Type status:** Other material. **Occurrence:** catalogNumber: K000766014; recordNumber: 2585; recordedBy: Chuwa, S; **Taxon:** scientificName: *Eriochloa nubica* (Steud.) Hack. & Stapf ex Thell.; kingdom: Plantae; family: Poaceae; genus: Eriochloa; specificEpithet: nubica; scientificNameAuthorship: (Steud.) Hack. & Stapf ex Thell.; **Location:** continent: Africa; country: Tanzania; stateProvince: Arusha; county: Ngorongoro; locality: Esere; verbatimLocality: Esere, near Kakesio, Ngorongoro conservation area.; minimumElevationInMeters: 1760; decimalLatitude: -3.316667; decimalLongitude: 35.033333; **Event:** eventDate: 1987-04-29; **Record Level:** institutionCode: K; collectionCode: Herbarium; ownerInstitutionCode: K; basisOfRecord: PreservedSpecimen
- **Type status:** Other material. **Occurrence:** catalogNumber: K000766016; recordNumber: 414; recordedBy: Paulo, S; **Taxon:** scientificName: *Eriochloa nubica* (Steud.) Hack. & Stapf ex Thell.; kingdom: Plantae; family: Poaceae; genus: Eriochloa; specificEpithet: nubica; scientificNameAuthorship: (Steud.) Hack. & Stapf ex Thell.; **Location:** continent: Africa; country: Tanzania; stateProvince: Mara; county: Serengeti; locality: Olndulen; verbatimLocality: Olndulen, Serengeti area.; **Event:** eventDate: 1958-04-29; **Record Level:** institutionCode: K; collectionCode: Herbarium; ownerInstitutionCode: K; basisOfRecord: PreservedSpecimen
- **Type status:** Other material. **Occurrence:** catalogNumber: K001087112; recordNumber: 2097K; recordedBy: Chuwa, SM; **Taxon:** scientificName: *Eriochloa nubica* (Steud.) Hack. & Stapf ex Thell.; kingdom: Plantae; family: Poaceae; genus: Eriochloa; specificEpithet: nubica; scientificNameAuthorship: (Steud.) Hack. & Stapf ex Thell.; **Location:** continent: Africa; country: Tanzania; stateProvince: Arusha; county: Ngorongoro; locality: Ngorongoro Crater; decimalLatitude: -3.166667; decimalLongitude: 35.583333; **Event:** eventDate: 1981-05-15; **Record Level:** institutionCode: K; collectionCode: Herbarium; ownerInstitutionCode: K; basisOfRecord: PreservedSpecimen
- **Type status:** Other material. **Occurrence:** catalogNumber: 1100; recordNumber: 2585; recordedBy: Chuwa, S; **Taxon:** scientificName: *Eriochloa nubica* (Steud.) Hack. & Stapf ex Thell.; kingdom: Plantae; family: Poaceae; genus: Eriochloa; specificEpithet: nubica; scientificNameAuthorship: (Steud.) Hack. & Stapf ex Thell.; **Location:** continent: Africa; country: Tanzania; stateProvince: Arusha; county: Ngorongoro; locality: Esere; verbatimLocality: Esere, near Kakesio, Ngorongoro conservation area.; minimumElevationInMeters: 1760; decimalLatitude: -3.316667; decimalLongitude: 35.033333; **Event:** eventDate: 1987-04-29; **Record Level:** institutionCode: NHT; collectionCode: Herbarium; ownerInstitutionCode: NHT; basisOfRecord: PreservedSpecimen

##### Distribution

Tropical Africa & Arabia

#### Eriochloa meyeriana

(Nees) Pilg.

##### Materials

- **Type status:** Other material. **Occurrence:** catalogNumber: K000984119; recordNumber: 9036; recordedBy: Greenway, PJ; **Taxon:** scientificName: *Eriochloa meyeriana* (Nees) Pilg.; kingdom: Plantae; family: Poaceae; genus: Eriochloa; specificEpithet: meyeriana; scientificNameAuthorship: (Nees) Pilg.; **Location:** continent: Africa; country: Tanzania; stateProvince: Mara; county: Serengeti; locality: Banagi Hill; verbatimLocality: westwards on the Grumeti river; decimalLatitude: -2.3; decimalLongitude: 34.833333; **Event:** eventDate: 1956-11-19; **Record Level:** institutionCode: K; collectionCode: Herbarium; ownerInstitutionCode: K; basisOfRecord: PreservedSpecimen
- **Type status:** Other material. **Occurrence:** catalogNumber: K000984120; recordNumber: 10179; recordedBy: Greenway, PJ; **Taxon:** scientificName: *Eriochloa meyeriana* (Nees) Pilg.; kingdom: Plantae; family: Poaceae; genus: Eriochloa; specificEpithet: meyeriana; scientificNameAuthorship: (Nees) Pilg.; **Location:** continent: Africa; country: Tanzania; stateProvince: Mara; county: Serengeti; locality: Seronera river; verbatimLocality: Serengeti; decimalLatitude: -2.45; decimalLongitude: 34.833333; **Event:** eventDate: 1961-05-12; **Record Level:** institutionCode: K; collectionCode: Herbarium; ownerInstitutionCode: K; basisOfRecord: PreservedSpecimen
- **Type status:** Other material. **Occurrence:** catalogNumber: DB0360; recordNumber: 10179; recordedBy: Greenway, PJ; **Taxon:** scientificName: *Eriochloa meyeriana* (Nees) Pilg.; kingdom: Plantae; family: Poaceae; genus: Eriochloa; specificEpithet: meyeriana; scientificNameAuthorship: (Nees) Pilg.; **Location:** continent: Africa; country: Tanzania; stateProvince: Mara; county: Serengeti; locality: Seronera river; verbatimLocality: Serengeti; decimalLatitude: -2.45; decimalLongitude: 34.833333; **Event:** eventDate: 1961-05-12; **Record Level:** institutionCode: SWRC; collectionCode: Herbarium; ownerInstitutionCode: SWRC; basisOfRecord: PreservedSpecimen
- **Type status:** Other material. **Occurrence:** catalogNumber: DB0361; recordNumber: 10600; recordedBy: Greenway, PJ; **Taxon:** scientificName: *Eriochloa meyeriana* (Nees) Pilg.; kingdom: Plantae; family: Poaceae; genus: Eriochloa; specificEpithet: meyeriana; scientificNameAuthorship: (Nees) Pilg.; **Location:** continent: Africa; country: Tanzania; stateProvince: Mara; county: Serengeti; locality: Seronera river; verbatimLocality: Seronera River below Sid Downeys Dam; minimumElevationInMeters: 1410; decimalLatitude: -2.433333; decimalLongitude: 34.816667; **Event:** eventDate: 1962-04-15; **Record Level:** institutionCode: SWRC; collectionCode: Herbarium; ownerInstitutionCode: SWRC; basisOfRecord: PreservedSpecimen

##### Distribution

Tropical Africa & Arabia

#### Eustachys paspaloides

(Vahl) Lanza & Mattei

##### Materials

- **Type status:** Other material. **Occurrence:** catalogNumber: 627; recordNumber: 24264; recordedBy: Peterson, PM; Soreng, RJ; Romaschenko, K; Mbago, F; **Taxon:** scientificName: *Eustachys paspaloides* (Vahl) Lanza & Mattei; kingdom: Plantae; family: Poaceae; genus: Eustachys; specificEpithet: paspaloides; scientificNameAuthorship: (Vahl) Lanza & Mattei; **Location:** continent: Africa; country: Tanzania; stateProvince: Shinyanga; locality: Naabi Hill Gate; verbatimLocality: Serengeti National Park, Naabi Hill Gate (at 0.5 km N).; minimumElevationInMeters: 1734; decimalLatitude: -2.83139; decimalLongitude: 34.99672; **Event:** eventDate: 2012-06-16; **Record Level:** institutionCode: US; collectionCode: Herbarium; ownerInstitutionCode: US; basisOfRecord: PreservedSpecimen
- **Type status:** Other material. **Occurrence:** catalogNumber: 628; recordNumber: 24295; recordedBy: Peterson, PM; Soreng, RJ; Romaschenko, K; Mbago, F; **Taxon:** scientificName: *Eustachys paspaloides* (Vahl) Lanza & Mattei; kingdom: Plantae; family: Poaceae; genus: Eustachys; specificEpithet: paspaloides; scientificNameAuthorship: (Vahl) Lanza & Mattei; **Location:** continent: Africa; country: Tanzania; stateProvince: Mara; county: Serengeti; locality: Lobo Lodge; verbatimLocality: Serengeti National Park, on top of Lobo Ridge above Lobo Lodge.; minimumElevationInMeters: 1817; decimalLatitude: -1.99967; decimalLongitude: 35.16778; **Event:** eventDate: 2012-06-17; **Record Level:** institutionCode: US; collectionCode: Herbarium; ownerInstitutionCode: US; basisOfRecord: PreservedSpecimen
- **Type status:** Other material. **Occurrence:** catalogNumber: K001087147; recordNumber: 9093; recordedBy: Greenway, PJ; **Taxon:** scientificName: *Eustachys paspaloides* (Vahl) Lanza & Mattei; kingdom: Plantae; family: Poaceae; genus: Eustachys; specificEpithet: paspaloides; scientificNameAuthorship: (Vahl) Lanza & Mattei; **Location:** continent: Africa; country: Tanzania; stateProvince: Mara; county: Serengeti; locality: Ndabaka Plains; verbatimLocality: South of Ndalaka plains; minimumElevationInMeters: 1280; decimalLatitude: -2.116667; decimalLongitude: 33.916667; **Event:** eventDate: 1956-12-01; **Record Level:** institutionCode: K; collectionCode: Herbarium; ownerInstitutionCode: K; basisOfRecord: PreservedSpecimen
- **Type status:** Other material. **Occurrence:** catalogNumber: K001087145; recordNumber: 347; recordedBy: Paulo, S; **Taxon:** scientificName: *Eustachys paspaloides* (Vahl) Lanza & Mattei; kingdom: Plantae; family: Poaceae; genus: Eustachys; specificEpithet: paspaloides; scientificNameAuthorship: (Vahl) Lanza & Mattei; **Location:** continent: Africa; country: Tanzania; stateProvince: Mara; county: Serengeti; locality: Serengeti Plains; decimalLatitude: -2.333333; decimalLongitude: 34.833333; **Event:** eventDate: 1958-04-22; **Record Level:** institutionCode: K; collectionCode: Herbarium; ownerInstitutionCode: K; basisOfRecord: PreservedSpecimen
- **Type status:** Other material. **Occurrence:** catalogNumber: K001087146; recordNumber: 65; recordedBy: Brooks, GP; **Taxon:** scientificName: *Eustachys paspaloides* (Vahl) Lanza & Mattei; kingdom: Plantae; family: Poaceae; genus: Eustachys; specificEpithet: paspaloides; scientificNameAuthorship: (Vahl) Lanza & Mattei; **Location:** continent: Africa; country: Tanzania; stateProvince: Mara; county: Serengeti; locality: Banagi Hill; verbatimLocality: West Serengeti; minimumElevationInMeters: 1463; decimalLatitude: -2.3; decimalLongitude: 34.833333; **Event:** eventDate: 1954-02-08; **Record Level:** institutionCode: K; collectionCode: Herbarium; ownerInstitutionCode: K; basisOfRecord: PreservedSpecimen
- **Type status:** Other material. **Occurrence:** catalogNumber: K001087148; recordNumber: 5596; recordedBy: Leippert, H; **Taxon:** scientificName: *Eustachys paspaloides* (Vahl) Lanza & Mattei; kingdom: Plantae; family: Poaceae; genus: Eustachys; specificEpithet: paspaloides; scientificNameAuthorship: (Vahl) Lanza & Mattei; **Location:** continent: Africa; country: Tanzania; stateProvince: Mara; county: Serengeti; locality: Serengeti Plains; verbatimLocality: Entrance to National Park, Maswa district.; minimumElevationInMeters: 1600; decimalLatitude: -2.333333; decimalLongitude: 34.833333; **Event:** eventDate: 1965-03-02; **Record Level:** institutionCode: K; collectionCode: Herbarium; ownerInstitutionCode: K; basisOfRecord: PreservedSpecimen
- **Type status:** Other material. **Occurrence:** catalogNumber: K001087149; recordNumber: 9845; recordedBy: Greenway, PJ; **Taxon:** scientificName: *Eustachys paspaloides* (Vahl) Lanza & Mattei; kingdom: Plantae; family: Poaceae; genus: Eustachys; specificEpithet: paspaloides; scientificNameAuthorship: (Vahl) Lanza & Mattei; **Location:** continent: Africa; country: Tanzania; stateProvince: Mara; county: Serengeti; locality: Seronera; verbatimLocality: Serengeti.; minimumElevationInMeters: 1554; decimalLatitude: -2.333333; decimalLongitude: 34.833333; **Event:** eventDate: 1961-03-18; **Record Level:** institutionCode: K; collectionCode: Herbarium; ownerInstitutionCode: K; basisOfRecord: PreservedSpecimen
- **Type status:** Other material. **Occurrence:** catalogNumber: K001087150; recordNumber: 364; recordedBy: Paulo, S; **Taxon:** scientificName: *Eustachys paspaloides* (Vahl) Lanza & Mattei; kingdom: Plantae; family: Poaceae; genus: Eustachys; specificEpithet: paspaloides; scientificNameAuthorship: (Vahl) Lanza & Mattei; **Location:** continent: Africa; country: Tanzania; stateProvince: Mara; county: Serengeti; locality: Banagi Hill; verbatimLocality: Serengeti.; decimalLatitude: -2.3; decimalLongitude: 34.833333; **Event:** eventDate: 1958-04-24; **Record Level:** institutionCode: K; collectionCode: Herbarium; ownerInstitutionCode: K; basisOfRecord: PreservedSpecimen
- **Type status:** Other material. **Occurrence:** catalogNumber: 1115; recordNumber: 909; recordedBy: Mollel, NP; Rusch, GM; Mwakalebe, G; **Taxon:** scientificName: *Eustachys paspaloides* (Vahl) Lanza & Mattei; kingdom: Plantae; family: Poaceae; genus: Eustachys; specificEpithet: paspaloides; scientificNameAuthorship: (Vahl) Lanza & Mattei; **Location:** continent: Africa; country: Tanzania; stateProvince: Mara; county: Serengeti; locality: Serengeti NP; verbatimLocality: Along the road to Nabaka gate, 36M, 0680713, 9750232 UTM; minimumElevationInMeters: 1314; decimalLatitude: -2.25; decimalLongitude: 34.333333; **Event:** eventDate: 2003-01-31; **Record Level:** institutionCode: NHT; collectionCode: Herbarium; ownerInstitutionCode: NHT; basisOfRecord: PreservedSpecimen

##### Distribution

Tropical Africa & Arabia

#### Exotheca abyssinica

(Hochst. ex A.Rich.) Andersson

##### Materials

- **Type status:** Other material. **Occurrence:** catalogNumber: 629; recordNumber: 771; recordedBy: Ellemann, L; **Taxon:** scientificName: *Exotheca abyssinica* (Hochst. ex A.Rich.) Andersson; kingdom: Plantae; family: Poaceae; genus: Exotheca; specificEpithet: abyssinica; scientificNameAuthorship: (Hochst. ex A.Rich.) Andersson; **Location:** continent: Africa; country: Tanzania; stateProvince: Arusha; county: Ngorongoro; locality: Oloronyo; verbatimLocality: Ngorongoro Conservation Area, Oloronyo,; minimumElevationInMeters: 2700; decimalLatitude: -3.2; decimalLongitude: 35.35; **Event:** eventDate: 1993-08-05; **Record Level:** institutionCode: AAU; collectionCode: Herbarium; ownerInstitutionCode: AAU; basisOfRecord: PreservedSpecimen
- **Type status:** Other material. **Occurrence:** catalogNumber: 1156; recordNumber: EN758; recordedBy: Njau, E; Leliyo, G; Andrew, P; Bamford, M; **Taxon:** scientificName: *Exotheca abyssinica* (Hochst. ex A.Rich.) Andersson; kingdom: Plantae; family: Poaceae; genus: Exotheca; specificEpithet: abyssinica; scientificNameAuthorship: (Hochst. ex A.Rich.) Andersson; **Location:** continent: Africa; country: Tanzania; stateProvince: Arusha; county: Ngorongoro; locality: Lemigrut; verbatimLocality: Ngorongoro conservation area, Lemigrut, south east slope.; minimumElevationInMeters: 2625; decimalLatitude: -3.186667; decimalLongitude: 35.378889; **Event:** eventDate: 2004-07-04; **Record Level:** institutionCode: NHT; collectionCode: Herbarium; ownerInstitutionCode: NHT; basisOfRecord: PreservedSpecimen

##### Distribution

Eastern & Southern Africa

#### Festuca obturbans

St.-Yves

##### Materials

- **Type status:** Other material. **Occurrence:** catalogNumber: K000984283; recordNumber: 9140; recordedBy: Greenway, PJ; **Taxon:** scientificName: *Festuca obturbans* St.-Yves; kingdom: Plantae; family: Poaceae; genus: Festuca; specificEpithet: obturbans; scientificNameAuthorship: St.-Yves; **Location:** continent: Africa; country: Tanzania; stateProvince: Arusha; county: Ngorongoro; locality: Empakai Crater; verbatimLocality: rim; minimumElevationInMeters: 3018; decimalLatitude: -2.933333; decimalLongitude: 35.816667; **Event:** eventDate: 1905-05-14; **Record Level:** institutionCode: K; collectionCode: Herbarium; ownerInstitutionCode: K; basisOfRecord: PreservedSpecimen

##### Distribution

Tanzania, Kenya & Yemen

#### Harpachne schimperi

A.Rich.

##### Materials

- **Type status:** Other material. **Occurrence:** catalogNumber: 630; recordNumber: s.n.; recordedBy: Mboya, E; **Taxon:** scientificName: *Harpachne schimperi* A.Rich.; kingdom: Plantae; family: Poaceae; genus: Harpachne; specificEpithet: schimperi; scientificNameAuthorship: A.Rich.; **Location:** continent: Africa; country: Tanzania; stateProvince: Mara; county: Serengeti; locality: Serengeti Research Centre; verbatimLocality: T1. Serengeti Research Centre.; minimumElevationInMeters: 1550; decimalLatitude: -2.38; decimalLongitude: 34.85; **Event:** eventDate: 2004-02-09; **Record Level:** institutionCode: MO; collectionCode: Herbarium; ownerInstitutionCode: MO; basisOfRecord: PreservedSpecimen
- **Type status:** Other material. **Occurrence:** catalogNumber: 631; recordNumber: 24266; recordedBy: Peterson, PM; Soreng, RJ; Romaschenko, K; Mbago, F; **Taxon:** scientificName: *Harpachne schimperi* A.Rich.; kingdom: Plantae; family: Poaceae; genus: Harpachne; specificEpithet: schimperi; scientificNameAuthorship: A.Rich.; **Location:** continent: Africa; country: Tanzania; stateProvince: Shinyanga; locality: Naabi Hill Gate; verbatimLocality: Serengeti National Park, Naabi Hill Gate (at 0.5 km N).; minimumElevationInMeters: 1734; decimalLatitude: -2.83139; decimalLongitude: 34.99672; **Event:** eventDate: 2012-06-16; **Record Level:** institutionCode: US; collectionCode: Herbarium; ownerInstitutionCode: US; basisOfRecord: PreservedSpecimen
- **Type status:** Other material. **Occurrence:** catalogNumber: 632; recordNumber: 24285; recordedBy: Peterson, PM; Soreng, RJ; Romaschenko, K; Mbago, F; **Taxon:** scientificName: *Harpachne schimperi* A.Rich.; kingdom: Plantae; family: Poaceae; genus: Harpachne; specificEpithet: schimperi; scientificNameAuthorship: A.Rich.; **Location:** continent: Africa; country: Tanzania; stateProvince: Mara; county: Serengeti; locality: Mbuzi Mare camp; verbatimLocality: Serengeti National Park, near Mbuzi Mare camp.; minimumElevationInMeters: 1552; decimalLatitude: -2.23332; decimalLongitude: 34.96467; **Event:** eventDate: 2012-06-17; **Record Level:** institutionCode: US; collectionCode: Herbarium; ownerInstitutionCode: US; basisOfRecord: PreservedSpecimen
- **Type status:** Other material. **Occurrence:** catalogNumber: 633; recordNumber: 24293; recordedBy: Peterson, PM; Soreng, RJ; Romaschenko, K; Mbago, F; **Taxon:** scientificName: *Harpachne schimperi* A.Rich.; kingdom: Plantae; family: Poaceae; genus: Harpachne; specificEpithet: schimperi; scientificNameAuthorship: A.Rich.; **Location:** continent: Africa; country: Tanzania; stateProvince: Mara; county: Serengeti; locality: Lobo Lodge; verbatimLocality: Serengeti National Park, on top of Lobo Ridge above Lobo Lodge.; minimumElevationInMeters: 1817; decimalLatitude: -1.99967; decimalLongitude: 35.16778; **Event:** eventDate: 2012-06-17; **Record Level:** institutionCode: US; collectionCode: Herbarium; ownerInstitutionCode: US; basisOfRecord: PreservedSpecimen
- **Type status:** Other material. **Occurrence:** catalogNumber: 634; recordNumber: 24319; recordedBy: Peterson, PM; Soreng, RJ; Romaschenko, K; Mbago, F; **Taxon:** scientificName: *Harpachne schimperi* A.Rich.; kingdom: Plantae; family: Poaceae; genus: Harpachne; specificEpithet: schimperi; scientificNameAuthorship: A.Rich.; **Location:** continent: Africa; country: Tanzania; stateProvince: Arusha; county: Ngorongoro; locality: Lake Magadi; verbatimLocality: Ngorongoro Conservation Area, W side of Lake Magadi.; minimumElevationInMeters: 1739; decimalLatitude: -3.17713; decimalLongitude: 35.51548; **Event:** eventDate: 2012-06-19; **Record Level:** institutionCode: US; collectionCode: Herbarium; ownerInstitutionCode: US; basisOfRecord: PreservedSpecimen

##### Distribution

Eastern Africa & Arabia

#### Helictotrichon elongatum

(Hochst. ex A.Rich.) C.E.Hubb

##### Materials

- **Type status:** Other material. **Occurrence:** catalogNumber: K000984279; recordNumber: P82; recordedBy: Frame, GW; **Taxon:** scientificName: *Helictotrichon elongatum* (Hochst. ex A.Rich.) C.E.Hubb; kingdom: Plantae; family: Poaceae; genus: Helictotrichon; specificEpithet: elongatum; scientificNameAuthorship: (Hochst. ex A.Rich.) C.E.Hubb; **Location:** continent: Africa; country: Tanzania; stateProvince: Arusha; county: Ngorongoro; locality: Empakai Crater; verbatimLocality: Ngorongoro Conservation Area; minimumElevationInMeters: 2900; decimalLatitude: -2.933333; decimalLongitude: 35.816667; **Event:** eventDate: 1974-02-26; **Record Level:** institutionCode: K; collectionCode: Herbarium; ownerInstitutionCode: K; basisOfRecord: PreservedSpecimen

##### Distribution

Tropical Africa

#### Helictotrichon lachnanthum

(Hochst. ex A.Rich.) C.E.Hubb.

##### Materials

- **Type status:** Other material. **Occurrence:** catalogNumber: K001087142; recordNumber: 9130; recordedBy: Greenway, PJ; **Taxon:** scientificName: *Helictotrichon lachnanthum* (Hochst. ex A.Rich.) C.E.Hubb.; kingdom: Plantae; family: Poaceae; genus: Helictotrichon; specificEpithet: lachnanthum; scientificNameAuthorship: (Hochst. ex A.Rich.) C.E.Hubb.; **Location:** continent: Africa; country: Tanzania; stateProvince: Arusha; county: Ngorongoro; locality: Empakai Crater; verbatimLocality: On crater rim. Embagai Crater; minimumElevationInMeters: 3018; decimalLatitude: -2.933333; decimalLongitude: 35.816667; **Event:** eventDate: 1956-12-07; **Record Level:** institutionCode: K; collectionCode: Herbarium; ownerInstitutionCode: K; basisOfRecord: PreservedSpecimen
- **Type status:** Other material. **Occurrence:** catalogNumber: K001087144; recordNumber: 5614; recordedBy: Newbould, JB; **Taxon:** scientificName: *Helictotrichon lachnanthum* (Hochst. ex A.Rich.) C.E.Hubb.; kingdom: Plantae; family: Poaceae; genus: Helictotrichon; specificEpithet: lachnanthum; scientificNameAuthorship: (Hochst. ex A.Rich.) C.E.Hubb.; **Location:** continent: Africa; country: Tanzania; stateProvince: Arusha; county: Ngorongoro; locality: Nainokanoka; decimalLatitude: -3.016667; decimalLongitude: 35.683333; **Event:** eventDate: 1961-01-07; **Record Level:** institutionCode: K; collectionCode: Herbarium; ownerInstitutionCode: K; basisOfRecord: PreservedSpecimen
- **Type status:** Other material. **Occurrence:** catalogNumber: K001087143; recordNumber: 189; recordedBy: Frame, GW; **Taxon:** scientificName: *Helictotrichon lachnanthum* (Hochst. ex A.Rich.) C.E.Hubb.; kingdom: Plantae; family: Poaceae; genus: Helictotrichon; specificEpithet: lachnanthum; scientificNameAuthorship: (Hochst. ex A.Rich.) C.E.Hubb.; **Location:** continent: Africa; country: Tanzania; stateProvince: Arusha; county: Ngorongoro; locality: Empakai Crater; verbatimLocality: Ngorongoro conservation area, Empakaai crater, north slope of Jaeger summit.; minimumElevationInMeters: 3100; decimalLatitude: -2.933333; decimalLongitude: 35.816667; **Event:** eventDate: 1973-06-23; **Record Level:** institutionCode: K; collectionCode: Herbarium; ownerInstitutionCode: K; basisOfRecord: PreservedSpecimen

##### Distribution

Ghana, Ethiopia, Kenya, Tanzania & Uganda

#### Heteropogon contortus

(L.) P.Beauv. ex Roem. & Schult.

##### Materials

- **Type status:** Other material. **Occurrence:** catalogNumber: K000984039; recordNumber: 9857; recordedBy: Greenway, PJ; **Taxon:** scientificName: *Heteropogon contortus* (L.) P.Beauv. ex Roem. & Schult.; kingdom: Plantae; family: Poaceae; genus: Heteropogon; specificEpithet: contortus; scientificNameAuthorship: (L.) P.Beauv. ex Roem. & Schult.; **Location:** continent: Africa; country: Tanzania; stateProvince: Mara; county: Serengeti; locality: Seronera; verbatimLocality: Serengeti; minimumElevationInMeters: 1554; decimalLatitude: -2.45; decimalLongitude: 34.833333; **Event:** eventDate: 1961-03-20; **Record Level:** institutionCode: K; collectionCode: Herbarium; ownerInstitutionCode: K; basisOfRecord: PreservedSpecimen
- **Type status:** Other material. **Occurrence:** catalogNumber: 635; recordNumber: s.n.; recordedBy: Mboya, E; **Taxon:** scientificName: *Heteropogon contortus* (L.) P.Beauv. ex Roem. & Schult.; kingdom: Plantae; family: Poaceae; genus: Heteropogon; specificEpithet: contortus; scientificNameAuthorship: (L.) P.Beauv. ex Roem. & Schult.; **Location:** continent: Africa; country: Tanzania; stateProvince: Mara; county: Serengeti; locality: Serengeti Research Centre; verbatimLocality: T1. Serengeti Research Centre.; minimumElevationInMeters: 1550; decimalLatitude: -2.38; decimalLongitude: 34.85; **Event:** eventDate: 2004-02-09; **Record Level:** institutionCode: MO; collectionCode: Herbarium; ownerInstitutionCode: MO; basisOfRecord: PreservedSpecimen
- **Type status:** Other material. **Occurrence:** catalogNumber: 1154; recordNumber: 847; recordedBy: Mollel, NP; Rusch, GM; Mwakalebe, G; **Taxon:** scientificName: *Heteropogon contortus* (L.) P.Beauv. ex Roem. & Schult.; kingdom: Plantae; family: Poaceae; genus: Heteropogon; specificEpithet: contortus; scientificNameAuthorship: (L.) P.Beauv. ex Roem. & Schult.; **Location:** continent: Africa; country: Tanzania; stateProvince: Mara; county: Serengeti; locality: Robanda village; verbatimLocality: Serengeti district, Robanda village, road to the water pool, CG01 plot. 36M, 0386088, 9763754 UTM.; minimumElevationInMeters: 1388; decimalLatitude: -2.083333; decimalLongitude: 34.666667; **Event:** eventDate: 2003-01-28; **Record Level:** institutionCode: NHT; collectionCode: Herbarium; ownerInstitutionCode: NHT; basisOfRecord: PreservedSpecimen
- **Type status:** Other material. **Occurrence:** catalogNumber: 1155; recordNumber: 3; recordedBy: Metele, P; **Taxon:** scientificName: *Heteropogon contortus* (L.) P.Beauv. ex Roem. & Schult.; kingdom: Plantae; family: Poaceae; genus: Heteropogon; specificEpithet: contortus; scientificNameAuthorship: (L.) P.Beauv. ex Roem. & Schult.; **Location:** continent: Africa; country: Tanzania; stateProvince: Arusha; county: Ngorongoro; locality: Ngorongoro Crater; verbatimLocality: Ngorongoro crater, Misigiyo, 15.1km from NCAA headquarters.; minimumElevationInMeters: 2270; decimalLatitude: -3.21; decimalLongitude: 35.354167; **Event:** eventDate: 1997-06-17; **Record Level:** institutionCode: NHT; collectionCode: Herbarium; ownerInstitutionCode: NHT; basisOfRecord: PreservedSpecimen

##### Distribution

Widespread

#### Hyparrhenia sp.

##### Materials

- **Type status:** Other material. **Occurrence:** catalogNumber: 638; recordNumber: s.n.; recordedBy: Mboya, E; **Taxon:** scientificName: Hyparrhenia; kingdom: Plantae; family: Poaceae; genus: Hyparrhenia; **Location:** continent: Africa; country: Tanzania; stateProvince: Mara; county: Serengeti; locality: Serengeti National Park; verbatimLocality: T1. Serengeti National Park. Ndabaka-Seronera Road.; minimumElevationInMeters: 1307; decimalLatitude: -2.27; decimalLongitude: 34.53; **Event:** eventDate: 2004-03-09; **Record Level:** institutionCode: MO; collectionCode: Herbarium; ownerInstitutionCode: MO; basisOfRecord: PreservedSpecimen
- **Type status:** Other material. **Occurrence:** catalogNumber: 1150; recordNumber: EN789; recordedBy: Njau, E; Leliyo, G; Andrew, P; Bamford, M; **Taxon:** scientificName: Hyparrhenia; kingdom: Plantae; family: Poaceae; genus: Hyparrhenia; **Location:** continent: Africa; country: Tanzania; stateProvince: Arusha; county: Ngorongoro; locality: Lemigrut; verbatimLocality: Ngorongoro conservation area, Lemigrut, south east slope.; minimumElevationInMeters: 2625; decimalLatitude: -3.186667; decimalLongitude: 35.378889; **Event:** eventDate: 2004-07-04; **Record Level:** institutionCode: NHT; collectionCode: Herbarium; ownerInstitutionCode: NHT; basisOfRecord: PreservedSpecimen

#### Hyparrhenia anamesa

Clayton

##### Materials

- **Type status:** Other material. **Occurrence:** catalogNumber: K000984012; recordNumber: 12598; recordedBy: Greenway, PJ; Kanuri; **Taxon:** scientificName: *Hyparrhenia anamesa* Clayton; kingdom: Plantae; family: Poaceae; genus: Hyparrhenia; specificEpithet: anamesa; scientificNameAuthorship: Clayton; **Location:** continent: Africa; country: Tanzania; stateProvince: Arusha; county: Ngorongoro; locality: Ngorongoro Crater; verbatimLocality: Between Ngoitoktok springs and Oljoro Nyuki River, Ngorongoro crater floor; minimumElevationInMeters: 1768; decimalLatitude: -3.166667; decimalLongitude: 35.583333; **Event:** eventDate: 1966-07-21; **Record Level:** institutionCode: K; collectionCode: Herbarium; ownerInstitutionCode: K; basisOfRecord: PreservedSpecimen

##### Distribution

Tropical Africa

#### Hyparrhenia anthistirioides

(Hochst. & A.Rich.) Andersson ex Stapf

##### Materials

- **Type status:** Other material. **Occurrence:** catalogNumber: K000984015; recordNumber: 10363; recordedBy: Greenway, PJ; Tanner, M; **Taxon:** scientificName: *Hyparrhenia anthistirioides* (Hochst. & A.Rich.) Andersson ex Stapf; kingdom: Plantae; family: Poaceae; genus: Hyparrhenia; specificEpithet: anthistirioides; scientificNameAuthorship: (Hochst. & A.Rich.) Andersson ex Stapf; **Location:** continent: Africa; country: Tanzania; stateProvince: Shinyanga; locality: Ndugani Kopjes; verbatimLocality: S.W. Beacon, mile 26; minimumElevationInMeters: 1493; decimalLatitude: -3.25; decimalLongitude: 34.833333; **Event:** eventDate: 1961-06-06; **Record Level:** institutionCode: K; collectionCode: Herbarium; ownerInstitutionCode: K; basisOfRecord: PreservedSpecimen
- **Type status:** Other material. **Occurrence:** catalogNumber: K000984016; recordNumber: 13177; recordedBy: Greenway, PJ; Kanuri; **Taxon:** scientificName: *Hyparrhenia anthistirioides* (Hochst. & A.Rich.) Andersson ex Stapf; kingdom: Plantae; family: Poaceae; genus: Hyparrhenia; specificEpithet: anthistirioides; scientificNameAuthorship: (Hochst. & A.Rich.) Andersson ex Stapf; **Location:** continent: Africa; country: Tanzania; stateProvince: Shinyanga; locality: Moru Kopjes; minimumElevationInMeters: 1585; decimalLatitude: -2.75; decimalLongitude: 34.75; **Event:** eventDate: 1968-02-13; **Record Level:** institutionCode: K; collectionCode: Herbarium; ownerInstitutionCode: K; basisOfRecord: PreservedSpecimen
- **Type status:** Other material. **Occurrence:** catalogNumber: 1152; recordNumber: 1165; recordedBy: Mollel, NP; Mboya, EI; **Taxon:** scientificName: *Hyparrhenia anthistirioides* (Hochst. & A.Rich.) Andersson ex Stapf; kingdom: Plantae; family: Poaceae; genus: Hyparrhenia; specificEpithet: anthistirioides; scientificNameAuthorship: (Hochst. & A.Rich.) Andersson ex Stapf; **Location:** continent: Africa; country: Tanzania; stateProvince: Arusha; county: Ngorongoro; locality: Laetoli; verbatimLocality: Ngorongoro, Esere-Laetoli road, Laetoli road 2100m from the junction to the footprints site.; minimumElevationInMeters: 1630; decimalLatitude: -3.279444; decimalLongitude: 35.174444; **Event:** eventDate: 2008-04-08; **Record Level:** institutionCode: NHT; collectionCode: Herbarium; ownerInstitutionCode: NHT; basisOfRecord: PreservedSpecimen

##### Distribution

Tropical Africa

#### Hyparrhenia cymbaria

(L.) Stapf

##### Materials

- **Type status:** Other material. **Occurrence:** catalogNumber: K000984017; recordNumber: 119; recordedBy: Tosbrooke, J; **Taxon:** scientificName: *Hyparrhenia cymbaria* (L.) Stapf; kingdom: Plantae; family: Poaceae; genus: Hyparrhenia; specificEpithet: cymbaria; scientificNameAuthorship: (L.) Stapf; **Location:** continent: Africa; country: Tanzania; stateProvince: Arusha; county: Ngorongoro; locality: Ngorongoro; minimumElevationInMeters: 2286; decimalLatitude: -3; decimalLongitude: 35.5; **Event:** eventDate: 1936-6; **Record Level:** institutionCode: K; collectionCode: Herbarium; ownerInstitutionCode: K; basisOfRecord: PreservedSpecimen

##### Distribution

Tropical Africa & Asia

#### Hyparrhenia dregeana

(Nees) Stapf ex Stent

##### Materials

- **Type status:** Other material. **Occurrence:** catalogNumber: K000984014; recordNumber: 1343; recordedBy: Heady, AF; **Taxon:** scientificName: *Hyparrhenia dregeana* (Nees) Stapf ex Stent; kingdom: Plantae; family: Poaceae; genus: Hyparrhenia; specificEpithet: dregeana; scientificNameAuthorship: (Nees) Stapf ex Stent; **Location:** continent: Africa; country: Tanzania; stateProvince: Arusha; county: Ngorongoro; locality: Ngorongoro Crater; verbatimLocality: Ngorongoro crater; minimumElevationInMeters: 1829; decimalLatitude: -3.166667; decimalLongitude: 35.583333; **Event:** eventDate: 1959-02-15; **Record Level:** institutionCode: K; collectionCode: Herbarium; ownerInstitutionCode: K; basisOfRecord: PreservedSpecimen

##### Distribution

Tropical Africa & Arabia

#### Hyparrhenia filipendula

(Hochst.) Stapf

##### Materials

- **Type status:** Other material. **Occurrence:** catalogNumber: K000765579; recordNumber: 10911; recordedBy: Greenway, PJ; **Taxon:** scientificName: *Hyparrhenia filipendula* (Hochst.) Stapf; kingdom: Plantae; family: Poaceae; genus: Hyparrhenia; specificEpithet: filipendula; scientificNameAuthorship: (Hochst.) Stapf; **Location:** continent: Africa; country: Tanzania; stateProvince: Mara; county: Serengeti; locality: Duma river air strip; decimalLatitude: -2.666667; decimalLongitude: 34.333333; **Event:** eventDate: 1962-12-26; **Record Level:** institutionCode: K; collectionCode: Herbarium; ownerInstitutionCode: K; basisOfRecord: PreservedSpecimen
- **Type status:** Other material. **Occurrence:** catalogNumber: K000765598; recordNumber: 10128; recordedBy: Greenway, PJ; **Taxon:** scientificName: *Hyparrhenia filipendula* (Hochst.) Stapf; kingdom: Plantae; family: Poaceae; genus: Hyparrhenia; specificEpithet: filipendula; scientificNameAuthorship: (Hochst.) Stapf; **Location:** continent: Africa; country: Tanzania; stateProvince: Mara; county: Serengeti; locality: Tabora; verbatimLocality: Tabora, Mile 46.6 from the Bolongonja river via Klein's camp.; minimumElevationInMeters: 1615; decimalLatitude: -1.816667; decimalLongitude: 34.633333; **Event:** eventDate: 1961-04-30; **Record Level:** institutionCode: K; collectionCode: Herbarium; ownerInstitutionCode: K; basisOfRecord: PreservedSpecimen
- **Type status:** Other material. **Occurrence:** catalogNumber: K000765627; recordNumber: 10125; recordedBy: Greenway, PJ; **Taxon:** scientificName: *Hyparrhenia filipendula* (Hochst.) Stapf; kingdom: Plantae; family: Poaceae; genus: Hyparrhenia; specificEpithet: filipendula; scientificNameAuthorship: (Hochst.) Stapf; **Location:** continent: Africa; country: Tanzania; stateProvince: Arusha; county: Ngorongoro; locality: Klein's camp; verbatimLocality: Bolongonja river to Tabora river via Klein's camp Mile 25; minimumElevationInMeters: 1585; decimalLatitude: -1.7; decimalLongitude: 35.216667; **Event:** eventDate: 1961-04-30; **Record Level:** institutionCode: K; collectionCode: Herbarium; ownerInstitutionCode: K; basisOfRecord: PreservedSpecimen

##### Distribution

Tropical Africa & Asia

#### Hyparrhenia finitima

(Hochst.) Andersson ex Stapf

##### Materials

- **Type status:** Other material. **Occurrence:** catalogNumber: K000984006; recordNumber: 10072; recordedBy: Greenway, PJ; **Taxon:** scientificName: *Hyparrhenia finitima* (Hochst.) Andersson ex Stapf; kingdom: Plantae; family: Poaceae; genus: Hyparrhenia; specificEpithet: finitima; scientificNameAuthorship: (Hochst.) Andersson ex Stapf; **Location:** continent: Africa; country: Tanzania; stateProvince: Mara; county: Serengeti; locality: Seronera; verbatimLocality: Seronera Dam Paddock, Serengeti; minimumElevationInMeters: 1158; decimalLatitude: -2.45; decimalLongitude: 34.833333; **Event:** eventDate: 1961-04-19; **Record Level:** institutionCode: K; collectionCode: Herbarium; ownerInstitutionCode: K; basisOfRecord: PreservedSpecimen

##### Distribution

Tropical Africa

#### Hyparrhenia hirta

(L.) Stapf

##### Materials

- **Type status:** Other material. **Occurrence:** catalogNumber: K000984002; recordNumber: 12608; recordedBy: Greenway, PJ; Kanuri; **Taxon:** scientificName: *Hyparrhenia hirta* (L.) Stapf; kingdom: Plantae; family: Poaceae; genus: Hyparrhenia; specificEpithet: hirta; scientificNameAuthorship: (L.) Stapf; **Location:** continent: Africa; country: Tanzania; stateProvince: Arusha; county: Ngorongoro; locality: Engitati Hill; verbatimLocality: W. side of Ngorongoro Crater floor; minimumElevationInMeters: 1646; decimalLatitude: -3.133333; decimalLongitude: 35.55; **Event:** eventDate: 1966-07-24; **Record Level:** institutionCode: K; collectionCode: Herbarium; ownerInstitutionCode: K; basisOfRecord: PreservedSpecimen
- **Type status:** Other material. **Occurrence:** catalogNumber: K000984003; recordNumber: 2227; recordedBy: Chuwa, S; **Taxon:** scientificName: *Hyparrhenia hirta* (L.) Stapf; kingdom: Plantae; family: Poaceae; genus: Hyparrhenia; specificEpithet: hirta; scientificNameAuthorship: (L.) Stapf; **Location:** continent: Africa; country: Tanzania; stateProvince: Arusha; county: Ngorongoro; locality: Endulen; decimalLatitude: -3.183333; decimalLongitude: 35.15; **Event:** eventDate: 1983-01-10; **Record Level:** institutionCode: K; collectionCode: Herbarium; ownerInstitutionCode: K; basisOfRecord: PreservedSpecimen
- **Type status:** Other material. **Occurrence:** catalogNumber: K000984004; recordNumber: 2745; recordedBy: Chuwa, S; **Taxon:** scientificName: *Hyparrhenia hirta* (L.) Stapf; kingdom: Plantae; family: Poaceae; genus: Hyparrhenia; specificEpithet: hirta; scientificNameAuthorship: (L.) Stapf; **Location:** continent: Africa; country: Tanzania; stateProvince: Arusha; county: Ngorongoro; locality: Ngorongoro Crater; verbatimLocality: Ngorongoro Crater Rim; decimalLatitude: -3.166667; decimalLongitude: 35.583333; **Event:** eventDate: 1989-05-25; **Record Level:** institutionCode: K; collectionCode: Herbarium; ownerInstitutionCode: K; basisOfRecord: PreservedSpecimen
- **Type status:** Other material. **Occurrence:** catalogNumber: K000984005; recordNumber: 9999; recordedBy: Greenway, PJ; Tanner, M; **Taxon:** scientificName: *Hyparrhenia hirta* (L.) Stapf; kingdom: Plantae; family: Poaceae; genus: Hyparrhenia; specificEpithet: hirta; scientificNameAuthorship: (L.) Stapf; **Location:** continent: Africa; country: Tanzania; stateProvince: Arusha; county: Ngorongoro; locality: Klein's camp; minimumElevationInMeters: 1448; decimalLatitude: -1.7; decimalLongitude: 35.216667; **Event:** eventDate: 1961-04-06; **Record Level:** institutionCode: K; collectionCode: Herbarium; ownerInstitutionCode: K; basisOfRecord: PreservedSpecimen
- **Type status:** Other material. **Occurrence:** catalogNumber: K000984013; recordNumber: 1637; recordedBy: Heady, AF; **Taxon:** scientificName: *Hyparrhenia hirta* (L.) Stapf; kingdom: Plantae; family: Poaceae; genus: Hyparrhenia; specificEpithet: hirta; scientificNameAuthorship: (L.) Stapf; **Location:** continent: Africa; country: Tanzania; stateProvince: Arusha; county: Ngorongoro; locality: Ngorongoro Crater; verbatimLocality: Ngorongoro crater floor; minimumElevationInMeters: 1920; decimalLatitude: -3.166667; decimalLongitude: 35.583333; **Event:** eventDate: 1959-02-16; **Record Level:** institutionCode: K; collectionCode: Herbarium; ownerInstitutionCode: K; basisOfRecord: PreservedSpecimen
- **Type status:** Other material. **Occurrence:** catalogNumber: 636; recordNumber: s.n.; recordedBy: Metele, P; **Taxon:** scientificName: *Hyparrhenia hirta* (L.) Stapf; kingdom: Plantae; family: Poaceae; genus: Hyparrhenia; specificEpithet: hirta; scientificNameAuthorship: (L.) Stapf; **Location:** continent: Africa; country: Tanzania; stateProvince: Arusha; county: Ngorongoro; locality: Ngorongoro Crater; verbatimLocality: T2. Ngorongoro Crater, Misigiyo, 15.1 km from NCAA headquarters; minimumElevationInMeters: 2270; decimalLatitude: -3; decimalLongitude: 35; **Event:** eventDate: 1997-06-17; **Record Level:** institutionCode: MO; collectionCode: Herbarium; ownerInstitutionCode: MO; basisOfRecord: PreservedSpecimen
- **Type status:** Other material. **Occurrence:** catalogNumber: 1106; recordNumber: 12608; recordedBy: Greenway, PJ; Kanuri; **Taxon:** scientificName: *Hyparrhenia hirta* (L.) Stapf; kingdom: Plantae; family: Poaceae; genus: Hyparrhenia; specificEpithet: hirta; scientificNameAuthorship: (L.) Stapf; **Location:** continent: Africa; country: Tanzania; stateProvince: Arusha; county: Ngorongoro; locality: Engitati Hill; verbatimLocality: W. side of Ngorongoro Crater floor; minimumElevationInMeters: 1646; decimalLatitude: -3.133333; decimalLongitude: 35.55; **Event:** eventDate: 1966-07-24; **Record Level:** institutionCode: NHT; collectionCode: Herbarium; ownerInstitutionCode: NHT; basisOfRecord: PreservedSpecimen
- **Type status:** Other material. **Occurrence:** catalogNumber: 1107; recordNumber: 2745; recordedBy: Chuwa, S; **Taxon:** scientificName: *Hyparrhenia hirta* (L.) Stapf; kingdom: Plantae; family: Poaceae; genus: Hyparrhenia; specificEpithet: hirta; scientificNameAuthorship: (L.) Stapf; **Location:** continent: Africa; country: Tanzania; stateProvince: Arusha; county: Ngorongoro; locality: Ngorongoro Crater; verbatimLocality: Ngorongoro Crater Rim; decimalLatitude: -3.166667; decimalLongitude: 35.583333; **Event:** eventDate: 1989-05-25; **Record Level:** institutionCode: NHT; collectionCode: Herbarium; ownerInstitutionCode: NHT; basisOfRecord: PreservedSpecimen
- **Type status:** Other material. **Occurrence:** catalogNumber: 1149; recordNumber: 182; recordedBy: Frame, GW; **Taxon:** scientificName: *Hyparrhenia hirta* (L.) Stapf; kingdom: Plantae; family: Poaceae; genus: Hyparrhenia; specificEpithet: hirta; scientificNameAuthorship: (L.) Stapf; **Location:** continent: Africa; country: Tanzania; stateProvince: Arusha; county: Ngorongoro; locality: Empakaai crater; verbatimLocality: Ngorongoro conservation area, Empakaai crater, inside east rim.; minimumElevationInMeters: 2350; decimalLatitude: -2.933333; decimalLongitude: 35.816667; **Event:** eventDate: 1973-06-22; **Record Level:** institutionCode: NHT; collectionCode: Herbarium; ownerInstitutionCode: NHT; basisOfRecord: PreservedSpecimen

##### Distribution

Widespread

#### Hyparrhenia papillipes

(Hochst. ex A.Rich.) Andersson ex Stapf

##### Materials

- **Type status:** Other material. **Occurrence:** catalogNumber: K000984020; recordNumber: 10704; recordedBy: Greenway, PJ; Tanner, M; **Taxon:** scientificName: *Hyparrhenia papillipes* (Hochst. ex A.Rich.) Andersson ex Stapf; kingdom: Plantae; family: Poaceae; genus: Hyparrhenia; specificEpithet: papillipes; scientificNameAuthorship: (Hochst. ex A.Rich.) Andersson ex Stapf; **Location:** continent: Africa; country: Tanzania; stateProvince: Mara; county: Serengeti; locality: Engare Nanyuki; verbatimLocality: On an inselberg three miles west of the Eastern boundary near the Engari Nanyuki headwaters; minimumElevationInMeters: 1646; decimalLatitude: -2.616667; decimalLongitude: 35.216667; **Event:** eventDate: 1962-06-05; **Record Level:** institutionCode: K; collectionCode: Herbarium; ownerInstitutionCode: K; basisOfRecord: PreservedSpecimen
- **Type status:** Other material. **Occurrence:** catalogNumber: K000984021; recordNumber: 10690; recordedBy: Greenway, PJ; Tanner, M; **Taxon:** scientificName: *Hyparrhenia papillipes* (Hochst. ex A.Rich.) Andersson ex Stapf; kingdom: Plantae; family: Poaceae; genus: Hyparrhenia; specificEpithet: papillipes; scientificNameAuthorship: (Hochst. ex A.Rich.) Andersson ex Stapf; **Location:** continent: Africa; country: Tanzania; stateProvince: Mara; county: Serengeti; locality: Serengeti; verbatimLocality: Serengeti central plain, N.E. side; minimumElevationInMeters: 1524; decimalLatitude: -2.333333; decimalLongitude: 34.833333; **Event:** eventDate: 1962-06-04; **Record Level:** institutionCode: K; collectionCode: Herbarium; ownerInstitutionCode: K; basisOfRecord: PreservedSpecimen
- **Type status:** Other material. **Occurrence:** catalogNumber: K000984022; recordNumber: 6185; recordedBy: Newbould, JB; **Taxon:** scientificName: *Hyparrhenia papillipes* (Hochst. ex A.Rich.) Andersson ex Stapf; kingdom: Plantae; family: Poaceae; genus: Hyparrhenia; specificEpithet: papillipes; scientificNameAuthorship: (Hochst. ex A.Rich.) Andersson ex Stapf; **Location:** continent: Africa; country: Tanzania; stateProvince: Mara; county: Serengeti; locality: Engare Nanyuki; verbatimLocality: E. Serengeti; minimumElevationInMeters: 1524; decimalLatitude: -2.616667; decimalLongitude: 35.216667; **Event:** eventDate: 1962-07-18; **Record Level:** institutionCode: K; collectionCode: Herbarium; ownerInstitutionCode: K; basisOfRecord: PreservedSpecimen
- **Type status:** Other material. **Occurrence:** catalogNumber: K000984023; recordNumber: 494; recordedBy: Giemeisel, QL; Rephart, LW; **Taxon:** scientificName: *Hyparrhenia papillipes* (Hochst. ex A.Rich.) Andersson ex Stapf; kingdom: Plantae; family: Poaceae; genus: Hyparrhenia; specificEpithet: papillipes; scientificNameAuthorship: (Hochst. ex A.Rich.) Andersson ex Stapf; **Location:** continent: Africa; country: Tanzania; stateProvince: Arusha; county: Ngorongoro; locality: Ngorongoro; minimumElevationInMeters: 1730; decimalLatitude: -3.166667; decimalLongitude: 35.583333; **Event:** eventDate: 1927-09-18; **Record Level:** institutionCode: K; collectionCode: Herbarium; ownerInstitutionCode: K; basisOfRecord: PreservedSpecimen

##### Distribution

Eastern Africa, Yemen & Madagascar

#### Hyparrhenia pilgeriana

C.E.Hubb

##### Materials

- **Type status:** Other material. **Occurrence:** catalogNumber: K000984018; recordNumber: 140; recordedBy: Frame, GW; **Taxon:** scientificName: *Hyparrhenia pilgeriana* C.E.Hubb; kingdom: Plantae; family: Poaceae; genus: Hyparrhenia; specificEpithet: pilgeriana; scientificNameAuthorship: C.E.Hubb; **Location:** continent: Africa; country: Tanzania; stateProvince: Arusha; county: Ngorongoro; locality: Empakai Crater; verbatimLocality: Ngorongoro Conservation Area, top of northeast rim; minimumElevationInMeters: 2600; decimalLatitude: -2.933333; decimalLongitude: 35.816667; **Event:** eventDate: 1973-05-29; **Record Level:** institutionCode: K; collectionCode: Herbarium; ownerInstitutionCode: K; basisOfRecord: PreservedSpecimen
- **Type status:** Other material. **Occurrence:** catalogNumber: K000984019; recordNumber: 92; recordedBy: Frame, GW; **Taxon:** scientificName: *Hyparrhenia pilgeriana* C.E.Hubb; kingdom: Plantae; family: Poaceae; genus: Hyparrhenia; specificEpithet: pilgeriana; scientificNameAuthorship: C.E.Hubb; **Location:** continent: Africa; country: Tanzania; stateProvince: Arusha; county: Ngorongoro; locality: Empakai Crater; verbatimLocality: Ngorongoro Conservation Area, top of east rim; minimumElevationInMeters: 2600; decimalLatitude: -2.933333; decimalLongitude: 35.816667; **Event:** eventDate: 1973-03-14; **Record Level:** institutionCode: K; collectionCode: Herbarium; ownerInstitutionCode: K; basisOfRecord: PreservedSpecimen
- **Type status:** Other material. **Occurrence:** catalogNumber: 1108; recordNumber: 92; recordedBy: Frame, GW; **Taxon:** scientificName: *Hyparrhenia pilgeriana* C.E.Hubb; kingdom: Plantae; family: Poaceae; genus: Hyparrhenia; specificEpithet: pilgeriana; scientificNameAuthorship: C.E.Hubb; **Location:** continent: Africa; country: Tanzania; stateProvince: Arusha; county: Ngorongoro; locality: Empakai Crater; verbatimLocality: Ngorongoro Conservation Area, top of east rim; minimumElevationInMeters: 2600; decimalLatitude: -2.933333; decimalLongitude: 35.816667; **Event:** eventDate: 1973-03-14; **Record Level:** institutionCode: NHT; collectionCode: Herbarium; ownerInstitutionCode: NHT; basisOfRecord: PreservedSpecimen
- **Type status:** Other material. **Occurrence:** catalogNumber: 1109; recordNumber: 140; recordedBy: Frame, GW; **Taxon:** scientificName: *Hyparrhenia pilgeriana* C.E.Hubb; kingdom: Plantae; family: Poaceae; genus: Hyparrhenia; specificEpithet: pilgeriana; scientificNameAuthorship: C.E.Hubb; **Location:** continent: Africa; country: Tanzania; stateProvince: Arusha; county: Ngorongoro; locality: Empakai Crater; verbatimLocality: Ngorongoro Conservation Area, top of northeast rim; minimumElevationInMeters: 2600; decimalLatitude: -2.933333; decimalLongitude: 35.816667; **Event:** eventDate: 1973-05-29; **Record Level:** institutionCode: NHT; collectionCode: Herbarium; ownerInstitutionCode: NHT; basisOfRecord: PreservedSpecimen

##### Distribution

Tropical Africa

#### Hyparrhenia quarrei

Robyns

##### Materials

- **Type status:** Other material. **Occurrence:** catalogNumber: 637; recordNumber: 24309; recordedBy: Peterson, PM; Soreng, RJ; Romaschenko, K; Mbago, F; **Taxon:** scientificName: *Hyparrhenia quarrei* Robyns; kingdom: Plantae; family: Poaceae; genus: Hyparrhenia; specificEpithet: quarrei; scientificNameAuthorship: Robyns; **Location:** continent: Africa; country: Tanzania; stateProvince: Arusha; county: Ngorongoro; locality: Ngorongoro Crater; verbatimLocality: Ngorongoro Conservation Area, rim of Ngorongoro Crater (descent gate).; minimumElevationInMeters: 2168; decimalLatitude: -3.15462; decimalLongitude: 35.47717; **Event:** eventDate: 2012-06-19; **Record Level:** institutionCode: US; collectionCode: Herbarium; ownerInstitutionCode: US; basisOfRecord: PreservedSpecimen

##### Distribution

Tropical Africa

#### Hyparrhenia rufa

(Nees) Stapf

##### Materials

- **Type status:** Other material. **Occurrence:** catalogNumber: K000984007; recordNumber: 426; recordedBy: Paulo, S; **Taxon:** scientificName: *Hyparrhenia rufa* (Nees) Stapf; kingdom: Plantae; family: Poaceae; genus: Hyparrhenia; specificEpithet: rufa; scientificNameAuthorship: (Nees) Stapf; **Location:** continent: Africa; country: Tanzania; stateProvince: Shinyanga; locality: Subiti Hill; verbatimLocality: East of Subiti Hill; decimalLatitude: -3.433333; decimalLongitude: 34.95; **Event:** eventDate: 1958-05-03; **Record Level:** institutionCode: K; collectionCode: Herbarium; ownerInstitutionCode: K; basisOfRecord: PreservedSpecimen
- **Type status:** Other material. **Occurrence:** catalogNumber: K000984008; recordNumber: 10619; recordedBy: Greenway, PJ; Tanner, M; **Taxon:** scientificName: *Hyparrhenia rufa* (Nees) Stapf; kingdom: Plantae; family: Poaceae; genus: Hyparrhenia; specificEpithet: rufa; scientificNameAuthorship: (Nees) Stapf; **Location:** continent: Africa; country: Tanzania; stateProvince: Mara; county: Serengeti; locality: Musabi; verbatimLocality: Mile 8, W. of Musabi; decimalLatitude: -2.233333; decimalLongitude: 34.466667; **Event:** eventDate: 1962-04-20; **Record Level:** institutionCode: K; collectionCode: Herbarium; ownerInstitutionCode: K; basisOfRecord: PreservedSpecimen
- **Type status:** Other material. **Occurrence:** catalogNumber: K000984009; recordNumber: 10554; recordedBy: Greenway, PJ; Tanner, M; Harvey, G; **Taxon:** scientificName: *Hyparrhenia rufa* (Nees) Stapf; kingdom: Plantae; family: Poaceae; genus: Hyparrhenia; specificEpithet: rufa; scientificNameAuthorship: (Nees) Stapf; **Location:** continent: Africa; country: Tanzania; stateProvince: Shinyanga; locality: Beacon Area; verbatimLocality: S.W. Beacon Area; minimumElevationInMeters: 1524; decimalLatitude: -3.283333; decimalLongitude: 34.8; **Event:** eventDate: 1962-03-27; **Record Level:** institutionCode: K; collectionCode: Herbarium; ownerInstitutionCode: K; basisOfRecord: PreservedSpecimen
- **Type status:** Other material. **Occurrence:** catalogNumber: K000984010; recordNumber: 10507; recordedBy: Greenway, PJ; Tanner, M; Allen, J; **Taxon:** scientificName: *Hyparrhenia rufa* (Nees) Stapf; kingdom: Plantae; family: Poaceae; genus: Hyparrhenia; specificEpithet: rufa; scientificNameAuthorship: (Nees) Stapf; **Location:** continent: Africa; country: Tanzania; stateProvince: Mara; county: Serengeti; locality: Nyamakachowe; minimumElevationInMeters: 1189; decimalLatitude: -2.45; decimalLongitude: 34.55; **Event:** eventDate: 1962-03-07; **Record Level:** institutionCode: K; collectionCode: Herbarium; ownerInstitutionCode: K; basisOfRecord: PreservedSpecimen

##### Distribution

Tropical Africa

#### Hyperthelia dissoluta

(Nees ex Steud.) Clayton

##### Materials

- **Type status:** Other material. **Occurrence:** catalogNumber: K000984044; recordNumber: 9992; recordedBy: Greenway, PJ; Tanner, M; **Taxon:** scientificName: *Hyperthelia dissoluta* (Nees ex Steud.) Clayton; kingdom: Plantae; family: Poaceae; genus: Hyperthelia; specificEpithet: dissoluta; scientificNameAuthorship: (Nees ex Steud.) Clayton; **Location:** continent: Africa; country: Tanzania; stateProvince: Mara; county: Serengeti; locality: Seronera; verbatimLocality: Seronera to Kleins Camp, Mile 57; minimumElevationInMeters: 1646; decimalLatitude: -1.85; decimalLongitude: 35.416667; **Event:** eventDate: 1961-04-06; **Record Level:** institutionCode: K; collectionCode: Herbarium; ownerInstitutionCode: K; basisOfRecord: PreservedSpecimen
- **Type status:** Other material. **Occurrence:** catalogNumber: K000984045; recordNumber: 10090; recordedBy: Greenway, PJ; **Taxon:** scientificName: *Hyperthelia dissoluta* (Nees ex Steud.) Clayton; kingdom: Plantae; family: Poaceae; genus: Hyperthelia; specificEpithet: dissoluta; scientificNameAuthorship: (Nees ex Steud.) Clayton; **Location:** continent: Africa; country: Tanzania; stateProvince: Mara; county: Serengeti; locality: Kirawira Guard Post; verbatimLocality: From Kirawira Guard Post to Seronera on the return, Mile 95.1; minimumElevationInMeters: 1250; decimalLatitude: -2.166667; decimalLongitude: 34.15; **Event:** eventDate: 1961-04-24; **Record Level:** institutionCode: K; collectionCode: Herbarium; ownerInstitutionCode: K; basisOfRecord: PreservedSpecimen
- **Type status:** Other material. **Occurrence:** catalogNumber: 639; recordNumber: 24287; recordedBy: Peterson, PM; Soreng, RJ; Romaschenko, K; Mbago, F; **Taxon:** scientificName: *Hyperthelia dissoluta* (Nees ex Steud.) Clayton; kingdom: Plantae; family: Poaceae; genus: Hyperthelia; specificEpithet: dissoluta; scientificNameAuthorship: (Nees ex Steud.) Clayton; **Location:** continent: Africa; country: Tanzania; stateProvince: Mara; county: Serengeti; locality: Mbuzi Mare camp; verbatimLocality: Serengeti National Park, near Mbuzi Mare camp.; minimumElevationInMeters: 1552; decimalLatitude: -2.23332; decimalLongitude: 34.96467; **Event:** eventDate: 2012-06-17; **Record Level:** institutionCode: US; collectionCode: Herbarium; ownerInstitutionCode: US; basisOfRecord: PreservedSpecimen

##### Distribution

Tropical Africa

#### Ischaemum afrum

(J.F.Gmel.) Dandy

##### Materials

- **Type status:** Other material. **Occurrence:** catalogNumber: K000984056; recordNumber: 11846; recordedBy: Greenway, PJ; Kanuri; **Taxon:** scientificName: *Ischaemum afrum* (J.F.Gmel.) Dandy; kingdom: Plantae; family: Poaceae; genus: Ischaemum; specificEpithet: afrum; scientificNameAuthorship: (J.F.Gmel.) Dandy; **Location:** continent: Africa; country: Tanzania; stateProvince: Arusha; county: Ngorongoro; locality: Ngorongoro Crater; verbatimLocality: N.E. side; minimumElevationInMeters: 1524; decimalLatitude: -3.166667; decimalLongitude: 35.583333; **Event:** eventDate: 1965-06-11; **Record Level:** institutionCode: K; collectionCode: Herbarium; ownerInstitutionCode: K; basisOfRecord: PreservedSpecimen
- **Type status:** Other material. **Occurrence:** catalogNumber: 1151; recordNumber: EN729; recordedBy: Njau, E; Leliyo, G; Andrew, P; Bamford, M; **Taxon:** scientificName: *Ischaemum afrum* (J.F.Gmel.) Dandy; kingdom: Plantae; family: Poaceae; genus: Ischaemum; specificEpithet: afrum; scientificNameAuthorship: (J.F.Gmel.) Dandy; **Location:** continent: Africa; country: Tanzania; stateProvince: Arusha; county: Ngorongoro; locality: Ngorongoro CA; verbatimLocality: Ngorongoro conservation area, Noiti volcanic lahar, alkaline rocks area.; minimumElevationInMeters: 1800; decimalLatitude: -3.210833; decimalLongitude: 35.220556; **Event:** eventDate: 2004-07-02; **Record Level:** institutionCode: NHT; collectionCode: Herbarium; ownerInstitutionCode: NHT; basisOfRecord: PreservedSpecimen

##### Distribution

Tropical Africa & India

#### Leersia sp.

##### Materials

- **Type status:** Other material. **Occurrence:** catalogNumber: 640; recordNumber: 24323; recordedBy: Peterson, PM; Soreng, RJ; Romaschenko, K; Mbago, F; **Taxon:** scientificName: Leersia; kingdom: Plantae; family: Poaceae; genus: Leersia; **Location:** continent: Africa; country: Tanzania; stateProvince: Arusha; county: Ngorongoro; locality: Lake Magadi; verbatimLocality: Ngorongoro Conservation Area, Shore of Lake Ngoitoktok.; minimumElevationInMeters: 1752; decimalLatitude: -3.21029; decimalLongitude: 35.60078; **Event:** eventDate: 2012-06-19; **Record Level:** institutionCode: US; collectionCode: Herbarium; ownerInstitutionCode: US; basisOfRecord: PreservedSpecimen

#### Leersia denudata

Launert

##### Materials

- **Type status:** Other material. **Occurrence:** catalogNumber: 785; recordNumber: E25; recordedBy: Gilbert, VC; **Taxon:** scientificName: *Leersia denudata* Launert; kingdom: Plantae; family: Poaceae; genus: Leersia; specificEpithet: denudata; scientificNameAuthorship: Launert; **Location:** continent: Africa; country: Tanzania; stateProvince: Arusha; county: Ngorongoro; locality: Ngorongoro Crater; verbatimLocality: Crater floor.; decimalLatitude: -3; decimalLongitude: 35; **Event:** eventDate: 1966-10-10; **Record Level:** institutionCode: EA; collectionCode: Herbarium; ownerInstitutionCode: EA; basisOfRecord: PreservedSpecimen
- **Type status:** Other material. **Occurrence:** catalogNumber: 786; recordNumber: 12570; recordedBy: Greenway, PJ; Kanuri, K; **Taxon:** scientificName: *Leersia denudata* Launert; kingdom: Plantae; family: Poaceae; genus: Leersia; specificEpithet: denudata; scientificNameAuthorship: Launert; **Location:** continent: Africa; country: Tanzania; stateProvince: Arusha; county: Ngorongoro; locality: Ngorongoro Crater; verbatimLocality: Floor. Munge swamp N. W. Ngorongoro.; decimalLatitude: -3; decimalLongitude: 35; **Event:** eventDate: 1966-07-14; **Record Level:** institutionCode: EA; collectionCode: Herbarium; ownerInstitutionCode: EA; basisOfRecord: PreservedSpecimen

##### Distribution

Eastern & Southern Africa

#### Leersia hexandra

Sw.

##### Materials

- **Type status:** Other material. **Occurrence:** catalogNumber: K001087151; recordNumber: 6523; recordedBy: Newbould, JB; **Taxon:** scientificName: *Leersia hexandra* Sw.; kingdom: Plantae; family: Poaceae; genus: Leersia; specificEpithet: hexandra; scientificNameAuthorship: Sw.; **Location:** continent: Africa; country: Tanzania; stateProvince: Arusha; county: Ngorongoro; locality: Ngorongoro Crater; verbatimLocality: Near Fig- Tree kopje; minimumElevationInMeters: 1676; decimalLatitude: -3.166667; decimalLongitude: 35.583333; **Event:** eventDate: 1963-1; **Record Level:** institutionCode: K; collectionCode: Herbarium; ownerInstitutionCode: K; basisOfRecord: PreservedSpecimen

##### Distribution

Widespread

#### Loudetia sp.

##### Materials

- **Type status:** Other material. **Occurrence:** catalogNumber: 643; recordNumber: s.n.; recordedBy: Mboya, E; **Taxon:** scientificName: Loudetia; kingdom: Plantae; family: Poaceae; genus: Loudetia; **Location:** continent: Africa; country: Tanzania; stateProvince: Mara; county: Serengeti; locality: Serengeti Research Centre; verbatimLocality: T1. Serengeti Research Centre.; minimumElevationInMeters: 1550; decimalLatitude: -2.38; decimalLongitude: 34.85; **Event:** eventDate: 2004-02-09; **Record Level:** institutionCode: MO; collectionCode: Herbarium; ownerInstitutionCode: MO; basisOfRecord: PreservedSpecimen

#### Loudetia arundinacea

(Hochst. ex A.Rich.) Hochst. ex Steud.

##### Materials

- **Type status:** Other material. **Occurrence:** catalogNumber: K000984070; recordNumber: 10087; recordedBy: Greenway, PJ; **Taxon:** scientificName: *Loudetia arundinacea* (Hochst. ex A.Rich.) Hochst. ex Steud.; kingdom: Plantae; family: Poaceae; genus: Loudetia; specificEpithet: arundinacea; scientificNameAuthorship: (Hochst. ex A.Rich.) Hochst. ex Steud.; **Location:** continent: Africa; country: Tanzania; stateProvince: Mara; county: Serengeti; locality: Kirawira; verbatimLocality: Mile 55 from Seronera to Kirawira Guard Post; minimumElevationInMeters: 1219; decimalLatitude: -2.166667; decimalLongitude: 34.15; **Event:** eventDate: 1961-04-24; **Record Level:** institutionCode: K; collectionCode: Herbarium; ownerInstitutionCode: K; basisOfRecord: PreservedSpecimen
- **Type status:** Other material. **Occurrence:** catalogNumber: 642; recordNumber: 24282; recordedBy: Peterson, PM; Soreng, RJ; Romaschenko, K; Mbago, F; **Taxon:** scientificName: *Loudetia arundinacea* (Hochst. ex A.Rich.) Hochst. ex Steud.; kingdom: Plantae; family: Poaceae; genus: Loudetia; specificEpithet: arundinacea; scientificNameAuthorship: (Hochst. ex A.Rich.) Hochst. ex Steud.; **Location:** continent: Africa; country: Tanzania; stateProvince: Mara; county: Serengeti; locality: Mbuzi Mare camp; verbatimLocality: Serengeti National Park, near Mbuzi Mare camp.; minimumElevationInMeters: 1552; decimalLatitude: -2.23332; decimalLongitude: 34.96467; **Event:** eventDate: 2012-06-17; **Record Level:** institutionCode: US; collectionCode: Herbarium; ownerInstitutionCode: US; basisOfRecord: PreservedSpecimen

##### Distribution

Tropical Africa

#### Loudetia kagerensis

(K.Schum.) C.E.Hubb

##### Materials

- **Type status:** Other material. **Occurrence:** catalogNumber: K000984067; recordNumber: 13347; recordedBy: Greenway, PJ; Kanuri; **Taxon:** scientificName: *Loudetia kagerensis* (K.Schum.) C.E.Hubb; kingdom: Plantae; family: Poaceae; genus: Loudetia; specificEpithet: kagerensis; scientificNameAuthorship: (K.Schum.) C.E.Hubb; **Location:** continent: Africa; country: Tanzania; stateProvince: Mara; county: Serengeti; locality: Kampi ya pofu; minimumElevationInMeters: 1768; decimalLatitude: -2.166667; decimalLongitude: 35; **Event:** eventDate: 1968-02-28; **Record Level:** institutionCode: K; collectionCode: Herbarium; ownerInstitutionCode: K; basisOfRecord: PreservedSpecimen
- **Type status:** Other material. **Occurrence:** catalogNumber: K000984068; recordNumber: 9991; recordedBy: Greenway, PJ; Tanner, M; **Taxon:** scientificName: *Loudetia kagerensis* (K.Schum.) C.E.Hubb; kingdom: Plantae; family: Poaceae; genus: Loudetia; specificEpithet: kagerensis; scientificNameAuthorship: (K.Schum.) C.E.Hubb; **Location:** continent: Africa; country: Tanzania; stateProvince: Mara; county: Serengeti; locality: Seronera; verbatimLocality: Seronera to Kleins Camp, Mile 57; minimumElevationInMeters: 1646; decimalLatitude: -1.85; decimalLongitude: 35.416667; **Event:** eventDate: 1961-04-06; **Record Level:** institutionCode: K; collectionCode: Herbarium; ownerInstitutionCode: K; basisOfRecord: PreservedSpecimen
- **Type status:** Other material. **Occurrence:** catalogNumber: K000984069; recordNumber: 10133; recordedBy: Greenway, PJ; **Taxon:** scientificName: *Loudetia kagerensis* (K.Schum.) C.E.Hubb; kingdom: Plantae; family: Poaceae; genus: Loudetia; specificEpithet: kagerensis; scientificNameAuthorship: (K.Schum.) C.E.Hubb; **Location:** continent: Africa; country: Tanzania; stateProvince: Mara; county: Serengeti; locality: Tabora; verbatimLocality: Mile 46.6 from the Bolgonja River via Kleins Camp; minimumElevationInMeters: 1615; decimalLatitude: -1.816667; decimalLongitude: 34.633333; **Event:** eventDate: 1961-04-30; **Record Level:** institutionCode: K; collectionCode: Herbarium; ownerInstitutionCode: K; basisOfRecord: PreservedSpecimen
- **Type status:** Other material. **Occurrence:** catalogNumber: K000984071; recordNumber: 397; recordedBy: Paulo, S; **Taxon:** scientificName: *Loudetia kagerensis* (K.Schum.) C.E.Hubb; kingdom: Plantae; family: Poaceae; genus: Loudetia; specificEpithet: kagerensis; scientificNameAuthorship: (K.Schum.) C.E.Hubb; **Location:** continent: Africa; country: Tanzania; stateProvince: Mara; county: Serengeti; locality: Nyamakachowe Hill; decimalLatitude: -2.45; decimalLongitude: 34.55; **Event:** eventDate: 1958-04-28; **Record Level:** institutionCode: K; collectionCode: Herbarium; ownerInstitutionCode: K; basisOfRecord: PreservedSpecimen

##### Distribution

Tropical Africa

#### Melinis sp.

##### Materials

- **Type status:** Other material. **Occurrence:** catalogNumber: 645; recordNumber: s.n.; recordedBy: Mboya, E; **Taxon:** scientificName: Melinis; kingdom: Plantae; family: Poaceae; genus: Melinis; **Location:** continent: Africa; country: Tanzania; stateProvince: Mara; county: Serengeti; locality: Serengeti National Park; verbatimLocality: T1. Serengeti National Park.; minimumElevationInMeters: 1466; decimalLatitude: -2.36; decimalLongitude: 34.5; **Event:** eventDate: 2004-03-06; **Record Level:** institutionCode: MO; collectionCode: Herbarium; ownerInstitutionCode: MO; basisOfRecord: PreservedSpecimen

#### Melinis repens

(Willd.) Zizka

##### Materials

- **Type status:** Other material. **Occurrence:** catalogNumber: 644; recordNumber: s.n.; recordedBy: Mboya, E; **Taxon:** scientificName: *Melinis repens* (Willd.) Zizka; kingdom: Plantae; family: Poaceae; genus: Melinis; specificEpithet: repens; scientificNameAuthorship: (Willd.) Zizka; **Location:** continent: Africa; country: Tanzania; stateProvince: Mara; county: Serengeti; locality: Serengeti Research Centre; verbatimLocality: T1. Serengeti National Park. Serengeti Research Centre. Tawiri Hostel.; minimumElevationInMeters: 1551; decimalLatitude: -2.38; decimalLongitude: 34.85; **Event:** eventDate: 2004-03-04; **Record Level:** institutionCode: MO; collectionCode: Herbarium; ownerInstitutionCode: MO; basisOfRecord: PreservedSpecimen

##### Distribution

Widespread

#### Microchloa kunthii

Desv.

##### Materials

- **Type status:** Other material. **Occurrence:** catalogNumber: DB0225; recordNumber: 12969; recordedBy: Tanner, M; **Taxon:** scientificName: *Microchloa kunthii* Desv.; kingdom: Plantae; family: Poaceae; genus: Microchloa; specificEpithet: kunthii; scientificNameAuthorship: Desv.; **Location:** continent: Africa; country: Tanzania; stateProvince: Mara; county: Serengeti; locality: Seronera; verbatimLocality: T1 Seronera Serengeti.; decimalLatitude: -2.45; decimalLongitude: 34.833333; **Event:** eventDate: 1963-12-14; **Record Level:** institutionCode: SWRC; collectionCode: Herbarium; ownerInstitutionCode: SWRC; basisOfRecord: PreservedSpecimen
- **Type status:** Other material. **Occurrence:** catalogNumber: 316; recordNumber: 12969; recordedBy: Tanner, M; **Taxon:** scientificName: *Microchloa kunthii* Desv.; kingdom: Plantae; family: Poaceae; genus: Microchloa; specificEpithet: kunthii; scientificNameAuthorship: Desv.; **Location:** continent: Africa; country: Tanzania; stateProvince: Mara; county: Serengeti; locality: Seronera; verbatimLocality: T1 Seronera Serengeti.; decimalLatitude: -2.45; decimalLongitude: 34.833333; **Event:** eventDate: 1963-12-14; **Record Level:** institutionCode: EA; collectionCode: Herbarium; ownerInstitutionCode: EA; basisOfRecord: PreservedSpecimen
- **Type status:** Other material. **Occurrence:** catalogNumber: 646; recordNumber: 710; recordedBy: Ellemann, L; **Taxon:** scientificName: *Microchloa kunthii* Desv.; kingdom: Plantae; family: Poaceae; genus: Microchloa; specificEpithet: kunthii; scientificNameAuthorship: Desv.; **Location:** continent: Africa; country: Tanzania; stateProvince: Arusha; county: Ngorongoro; locality: Enja Shori; verbatimLocality: Ngorongoro Conservation Area, along Enja Shori,; minimumElevationInMeters: 1500; decimalLatitude: -3.033; decimalLongitude: 35.266; **Event:** eventDate: 1993-07-19; **Record Level:** institutionCode: AAU; collectionCode: Herbarium; ownerInstitutionCode: AAU; basisOfRecord: PreservedSpecimen
- **Type status:** Other material. **Occurrence:** catalogNumber: 647; recordNumber: 1016; recordedBy: Ellemann, L; **Taxon:** scientificName: *Microchloa kunthii* Desv.; kingdom: Plantae; family: Poaceae; genus: Microchloa; specificEpithet: kunthii; scientificNameAuthorship: Desv.; **Location:** continent: Africa; country: Tanzania; stateProvince: Arusha; county: Ngorongoro; locality: Olbili-Esere; verbatimLocality: Ngorongoro Conservation Area, Olbili-Esere.; minimumElevationInMeters: 1600; decimalLatitude: -3.283; decimalLongitude: 35.166; **Event:** eventDate: 1994-01-23; **Record Level:** institutionCode: AAU; collectionCode: Herbarium; ownerInstitutionCode: AAU; basisOfRecord: PreservedSpecimen
- **Type status:** Other material. **Occurrence:** catalogNumber: 648; recordNumber: 24255; recordedBy: Peterson, PM; Soreng, RJ; Romaschenko, K; Mbago, F; **Taxon:** scientificName: *Microchloa kunthii* Desv.; kingdom: Plantae; family: Poaceae; genus: Microchloa; specificEpithet: kunthii; scientificNameAuthorship: Desv.; **Location:** continent: Africa; country: Tanzania; stateProvince: Shinyanga; locality: Naabi Hill Gate; verbatimLocality: Serengeti National Park, Naabi Hill Gate (at 0.5 km N).; minimumElevationInMeters: 1734; decimalLatitude: -2.83139; decimalLongitude: 34.99672; **Event:** eventDate: 2012-06-16; **Record Level:** institutionCode: US; collectionCode: Herbarium; ownerInstitutionCode: US; basisOfRecord: PreservedSpecimen
- **Type status:** Other material. **Occurrence:** catalogNumber: 649; recordNumber: 24288; recordedBy: Peterson, PM; Soreng, RJ; Romaschenko, K; Mbago, F; **Taxon:** scientificName: *Microchloa kunthii* Desv.; kingdom: Plantae; family: Poaceae; genus: Microchloa; specificEpithet: kunthii; scientificNameAuthorship: Desv.; **Location:** continent: Africa; country: Tanzania; stateProvince: Mara; county: Serengeti; locality: Mbuzi Mare camp; verbatimLocality: Serengeti National Park, near Mbuzi Mare camp.; minimumElevationInMeters: 1552; decimalLatitude: -2.23332; decimalLongitude: 34.96467; **Event:** eventDate: 2012-06-17; **Record Level:** institutionCode: US; collectionCode: Herbarium; ownerInstitutionCode: US; basisOfRecord: PreservedSpecimen

##### Distribution

Widespread

#### Odyssea paucinervis

(Nees) Stapf

##### Materials

- **Type status:** Other material. **Occurrence:** catalogNumber: 650; recordNumber: 24312; recordedBy: Peterson, PM; Soreng, RJ; Romaschenko, K; Mbago, F; **Taxon:** scientificName: *Odyssea paucinervis* (Nees) Stapf; kingdom: Plantae; family: Poaceae; genus: Odyssea; specificEpithet: paucinervis; scientificNameAuthorship: (Nees) Stapf; **Location:** continent: Africa; country: Tanzania; stateProvince: Arusha; county: Ngorongoro; locality: Lake Magadi; verbatimLocality: Ngorongoro Conservation Area, W side of Lake Magadi.; minimumElevationInMeters: 1739; decimalLatitude: -3.17713; decimalLongitude: 35.51548; **Event:** eventDate: 2012-06-19; **Record Level:** institutionCode: US; collectionCode: Herbarium; ownerInstitutionCode: US; basisOfRecord: PreservedSpecimen
- **Type status:** Other material. **Occurrence:** catalogNumber: K001087154; recordNumber: E40; recordedBy: Gilbert, VC; **Taxon:** scientificName: *Odyssea paucinervis* (Nees) Stapf; kingdom: Plantae; family: Poaceae; genus: Odyssea; specificEpithet: paucinervis; scientificNameAuthorship: (Nees) Stapf; **Location:** continent: Africa; country: Tanzania; stateProvince: Arusha; county: Ngorongoro; locality: Lake Magadi; verbatimLocality: Ngorongoro crater, Lake Magadi (soda lake); minimumElevationInMeters: 1545; decimalLatitude: -3.183333; decimalLongitude: 35.533333; **Event:** eventDate: 1966-09-10; **Record Level:** institutionCode: K; collectionCode: Herbarium; ownerInstitutionCode: K; basisOfRecord: PreservedSpecimen
- **Type status:** Other material. **Occurrence:** catalogNumber: K001087152; recordNumber: 12576; recordedBy: Greenway, PJ; Kanuri; **Taxon:** scientificName: *Odyssea paucinervis* (Nees) Stapf; kingdom: Plantae; family: Poaceae; genus: Odyssea; specificEpithet: paucinervis; scientificNameAuthorship: (Nees) Stapf; **Location:** continent: Africa; country: Tanzania; stateProvince: Arusha; county: Ngorongoro; locality: Lake Magadi; verbatimLocality: East side of Lake Magadi, Ngorongoro crater floor.; minimumElevationInMeters: 1661; decimalLatitude: -3.183333; decimalLongitude: 35.533333; **Event:** eventDate: 1966-07-14; **Record Level:** institutionCode: K; collectionCode: Herbarium; ownerInstitutionCode: K; basisOfRecord: PreservedSpecimen
- **Type status:** Other material. **Occurrence:** catalogNumber: K001087153; recordNumber: 12576; recordedBy: Greenway, PJ; Kanuri; **Taxon:** scientificName: *Odyssea paucinervis* (Nees) Stapf; kingdom: Plantae; family: Poaceae; genus: Odyssea; specificEpithet: paucinervis; scientificNameAuthorship: (Nees) Stapf; **Location:** continent: Africa; country: Tanzania; stateProvince: Arusha; county: Ngorongoro; locality: Lake Magadi; verbatimLocality: East side of Lake Magadi, Ngorongoro crater floor.; minimumElevationInMeters: 1661; decimalLatitude: -3.183333; decimalLongitude: 35.533333; **Event:** eventDate: 1966-07-14; **Record Level:** institutionCode: K; collectionCode: Herbarium; ownerInstitutionCode: K; basisOfRecord: PreservedSpecimen
- **Type status:** Other material. **Occurrence:** catalogNumber: K001087155; recordNumber: 13597; recordedBy: Greenway, PJ; Kanuri; **Taxon:** scientificName: *Odyssea paucinervis* (Nees) Stapf; kingdom: Plantae; family: Poaceae; genus: Odyssea; specificEpithet: paucinervis; scientificNameAuthorship: (Nees) Stapf; **Location:** continent: Africa; country: Tanzania; stateProvince: Arusha; county: Ngorongoro; locality: Lake Magadi; verbatimLocality: West side of Lake Magadi, Ngorongoro crater floor.; minimumElevationInMeters: 1661; decimalLatitude: -3.183333; decimalLongitude: 35.533333; **Event:** eventDate: 1966-7; **Record Level:** institutionCode: K; collectionCode: Herbarium; ownerInstitutionCode: K; basisOfRecord: PreservedSpecimen

##### Distribution

Eastern & Southern Africa

#### Oldeania alpina

(K.Schum.) Stapleton

Arundinaria alpina K.Schum.

##### Materials

- **Type status:** Other material. **Occurrence:** catalogNumber: K001087114; recordNumber: 4223; recordedBy: Burtt, BD; **Taxon:** scientificName: *Arundinaria alpina* K.Schum.; kingdom: Plantae; family: Poaceae; genus: Arundinaria; specificEpithet: alpina; scientificNameAuthorship: K.Schum.; **Location:** continent: Africa; country: Tanzania; stateProvince: Arusha; county: Ngorongoro; locality: Oldeani Mt; verbatimLocality: Whole south and south east side of the mountain to the crater rim and part of the crater wall.; minimumElevationInMeters: 2896; maximumElevationInMeters: 3185; decimalLatitude: -3.266667; decimalLongitude: 35.433333; **Event:** eventDate: 1932-09-23; **Record Level:** institutionCode: K; collectionCode: Herbarium; ownerInstitutionCode: K; basisOfRecord: PreservedSpecimen
- **Type status:** Other material. **Occurrence:** catalogNumber: K001087113; recordNumber: 2679; recordedBy: Chuwa, S; **Taxon:** scientificName: *Arundinaria alpina* K.Schum.; kingdom: Plantae; family: Poaceae; genus: Arundinaria; specificEpithet: alpina; scientificNameAuthorship: K.Schum.; **Location:** continent: Africa; country: Tanzania; stateProvince: Arusha; county: Ngorongoro; locality: Oldeani Mt; verbatimLocality: Ngorongoro Conservation Area, Oldean Mountain; minimumElevationInMeters: 2439; decimalLatitude: -3.266667; decimalLongitude: 35.433333; **Event:** eventDate: 1988-10-21; **Record Level:** institutionCode: K; collectionCode: Herbarium; ownerInstitutionCode: K; basisOfRecord: PreservedSpecimen

##### Distribution

Tropical Africa

#### Oplismenus compositus

(L.) P.Beauv.

##### Materials

- **Type status:** Other material. **Occurrence:** catalogNumber: DB0232; recordNumber: 10754; recordedBy: Greenway, PJ; **Taxon:** scientificName: *Oplismenus compositus* (L.) P.Beauv.; kingdom: Plantae; family: Poaceae; genus: Oplismenus; specificEpithet: compositus; scientificNameAuthorship: (L.) P.Beauv.; **Location:** continent: Africa; country: Tanzania; stateProvince: Mara; county: Serengeti; locality: Bolgonja; verbatimLocality: T1, Bologonja River.; minimumElevationInMeters: 1440; decimalLatitude: -1.783333; decimalLongitude: 35.2; **Event:** eventDate: 1962-08-18; **Record Level:** institutionCode: SWRC; collectionCode: Herbarium; ownerInstitutionCode: SWRC; basisOfRecord: PreservedSpecimen

##### Distribution

Widespread

#### Oplismenus hirtellus

(L.) P.Beauv.

##### Materials

- **Type status:** Other material. **Occurrence:** catalogNumber: DB0238; recordNumber: 4657; recordedBy: Vesey-FitzGerald, LDEF; **Taxon:** scientificName: *Oplismenus hirtellus* (L.) P.Beauv.; kingdom: Plantae; family: Poaceae; genus: Oplismenus; specificEpithet: hirtellus; scientificNameAuthorship: (L.) P.Beauv.; **Location:** continent: Africa; country: Tanzania; stateProvince: Mara; county: Serengeti; locality: Serengeti National Park; verbatimLocality: Serengeti National Park, Kirawin; decimalLatitude: -2.333333; decimalLongitude: 34.833333; **Event:** eventDate: 1965-04-27; **Record Level:** institutionCode: SWRC; collectionCode: Herbarium; ownerInstitutionCode: SWRC; basisOfRecord: PreservedSpecimen

##### Distribution

Widespread

#### Oropetium capense

Stapf

##### Materials

- **Type status:** Other material. **Occurrence:** catalogNumber: DB0241; recordNumber: 9854; recordedBy: Greenway, PJ; **Taxon:** scientificName: *Oropetium capense* Stapf; kingdom: Plantae; family: Poaceae; genus: Oropetium; specificEpithet: capense; scientificNameAuthorship: Stapf; **Location:** continent: Africa; country: Tanzania; stateProvince: Mara; county: Serengeti; locality: Seronera; verbatimLocality: T1, Seronera, Serengeti; minimumElevationInMeters: 1530; decimalLatitude: -2.45; decimalLongitude: 34.833333; **Event:** eventDate: 1961-03-20; **Record Level:** institutionCode: SWRC; collectionCode: Herbarium; ownerInstitutionCode: SWRC; basisOfRecord: PreservedSpecimen

##### Distribution

Africa & Arabia

#### Oryza eichingeri

Peter

##### Materials

- **Type status:** Other material. **Occurrence:** catalogNumber: DB0246; recordNumber: 7591; recordedBy: Vesey-FitzGerald, LDEF; **Taxon:** scientificName: *Oryza eichingeri* Peter; kingdom: Plantae; family: Poaceae; genus: Oryza; specificEpithet: eichingeri; scientificNameAuthorship: Peter; **Location:** continent: Africa; country: Tanzania; stateProvince: Mara; county: Serengeti; locality: Serengeti National Park; verbatimLocality: Serengeti National Park Nyamburi; minimumElevationInMeters: 1500; decimalLatitude: -2.333333; decimalLongitude: 34.833333; **Event:** eventDate: 1973-11-04; **Record Level:** institutionCode: SWRC; collectionCode: Herbarium; ownerInstitutionCode: SWRC; basisOfRecord: PreservedSpecimen

##### Distribution

Tropical Africa & Sri Lanka

#### Panicum sp.

##### Materials

- **Type status:** Other material. **Occurrence:** catalogNumber: DB0405; recordNumber: 26; recordedBy: Braun, HMH; **Taxon:** scientificName: Panicum; kingdom: Plantae; family: Poaceae; genus: Panicum; **Location:** continent: Africa; country: Tanzania; stateProvince: Shinyanga; locality: Moru Kopjes; verbatimLocality: Moru Kapjes area; decimalLatitude: -2.75; decimalLongitude: 34.75; **Event:** eventDate: 1969-02-27; **Record Level:** institutionCode: SWRC; collectionCode: Herbarium; ownerInstitutionCode: SWRC; basisOfRecord: PreservedSpecimen
- **Type status:** Other material. **Occurrence:** catalogNumber: 1125; recordNumber: 1146; recordedBy: Macha; Leliyo, G; Mboya, EI; **Taxon:** scientificName: Panicum; kingdom: Plantae; family: Poaceae; genus: Panicum; **Location:** continent: Africa; country: Tanzania; stateProvince: Mara; county: Serengeti; locality: Serengeti Plains; verbatimLocality: Along the road from Muguma to Musoma on the Serengeti Plains at Nyichoka village.; minimumElevationInMeters: 1460; **Event:** eventDate: 1992-02-20; **Record Level:** institutionCode: NHT; collectionCode: Herbarium; ownerInstitutionCode: NHT; basisOfRecord: PreservedSpecimen

#### Panicum atrosanguineum

Hochst. ex A.Rich.

##### Materials

- **Type status:** Other material. **Occurrence:** catalogNumber: K000984144; recordNumber: 6569; recordedBy: Newbould, JB; **Taxon:** scientificName: *Panicum atrosanguineum* Hochst. ex A.Rich.; kingdom: Plantae; family: Poaceae; genus: Panicum; specificEpithet: atrosanguineum; scientificNameAuthorship: Hochst. ex A.Rich.; **Location:** continent: Africa; country: Tanzania; stateProvince: Arusha; county: Ngorongoro; locality: Endoinyo Emboleh; verbatimLocality: Olbalbal Escarpment; minimumElevationInMeters: 1829; decimalLatitude: -3.083333; decimalLongitude: 35.416667; **Event:** eventDate: 1963-2; **Record Level:** institutionCode: K; collectionCode: Herbarium; ownerInstitutionCode: K; basisOfRecord: PreservedSpecimen
- **Type status:** Other material. **Occurrence:** catalogNumber: K000984146; recordNumber: 2586; recordedBy: Chuwa, S; **Taxon:** scientificName: *Panicum atrosanguineum* Hochst. ex A.Rich.; kingdom: Plantae; family: Poaceae; genus: Panicum; specificEpithet: atrosanguineum; scientificNameAuthorship: Hochst. ex A.Rich.; **Location:** continent: Africa; country: Tanzania; stateProvince: Arusha; county: Ngorongoro; locality: Endulen; verbatimLocality: Ngorongoro Conservation Area; minimumElevationInMeters: 1890; decimalLatitude: -3.183333; decimalLongitude: 35.15; **Event:** eventDate: 1987-04-29; **Record Level:** institutionCode: K; collectionCode: Herbarium; ownerInstitutionCode: K; basisOfRecord: PreservedSpecimen
- **Type status:** Other material. **Occurrence:** catalogNumber: K000984147; recordNumber: 10149; recordedBy: Greenway, PJ; Talbots, L; **Taxon:** scientificName: *Panicum atrosanguineum* Hochst. ex A.Rich.; kingdom: Plantae; family: Poaceae; genus: Panicum; specificEpithet: atrosanguineum; scientificNameAuthorship: Hochst. ex A.Rich.; **Location:** continent: Africa; country: Tanzania; stateProvince: Mara; county: Serengeti; locality: Lake Lagarja; verbatimLocality: N.W. side of Lake Lagarja; minimumElevationInMeters: 1478; decimalLatitude: -3; decimalLongitude: 35.033333; **Event:** eventDate: 1961-05-07; **Record Level:** institutionCode: K; collectionCode: Herbarium; ownerInstitutionCode: K; basisOfRecord: PreservedSpecimen
- **Type status:** Other material. **Occurrence:** catalogNumber: K000984148; recordNumber: 10621; recordedBy: Greenway, PJ; Tanner, M; Watson; **Taxon:** scientificName: *Panicum atrosanguineum* Hochst. ex A.Rich.; kingdom: Plantae; family: Poaceae; genus: Panicum; specificEpithet: atrosanguineum; scientificNameAuthorship: Hochst. ex A.Rich.; **Location:** continent: Africa; country: Tanzania; stateProvince: Shinyanga; locality: Kitu Hill; verbatimLocality: Duma river - Mamerehe Guard Post, S.E. of Kitu Hill; minimumElevationInMeters: 1219; decimalLatitude: -2.45; decimalLongitude: 34.316667; **Event:** eventDate: 1962-04-20; **Record Level:** institutionCode: K; collectionCode: Herbarium; ownerInstitutionCode: K; basisOfRecord: PreservedSpecimen
- **Type status:** Other material. **Occurrence:** catalogNumber: DB0250; recordNumber: 128; recordedBy: Schmidt, W; **Taxon:** scientificName: *Panicum atrosanguineum* Hochst. ex A.Rich.; kingdom: Plantae; family: Poaceae; genus: Panicum; specificEpithet: atrosanguineum; scientificNameAuthorship: Hochst. ex A.Rich.; **Location:** continent: Africa; country: Tanzania; stateProvince: Mara; county: Serengeti; locality: Serengeti Plains; verbatimLocality: Naabi south Serengeti Plains; decimalLatitude: -2.333333; decimalLongitude: 34.833333; **Event:** eventDate: 1972-03-16; **Record Level:** institutionCode: SWRC; collectionCode: Herbarium; ownerInstitutionCode: SWRC; basisOfRecord: PreservedSpecimen
- **Type status:** Other material. **Occurrence:** catalogNumber: DB0251; recordNumber: 270; recordedBy: Brown, HM; **Taxon:** scientificName: *Panicum atrosanguineum* Hochst. ex A.Rich.; kingdom: Plantae; family: Poaceae; genus: Panicum; specificEpithet: atrosanguineum; scientificNameAuthorship: Hochst. ex A.Rich.; **Location:** continent: Africa; country: Tanzania; stateProvince: Shinyanga; locality: Naabi Hill; verbatimLocality: 9 km south -east of Naabi Hill.; minimumElevationInMeters: 1500; decimalLatitude: -2.333333; decimalLongitude: 34.833333; **Event:** eventDate: 1969-03-26; **Record Level:** institutionCode: SWRC; collectionCode: Herbarium; ownerInstitutionCode: SWRC; basisOfRecord: PreservedSpecimen
- **Type status:** Other material. **Occurrence:** catalogNumber: 323; recordNumber: 270; recordedBy: Brown, HM; **Taxon:** scientificName: *Panicum atrosanguineum* Hochst. ex A.Rich.; kingdom: Plantae; family: Poaceae; genus: Panicum; specificEpithet: atrosanguineum; scientificNameAuthorship: Hochst. ex A.Rich.; **Location:** continent: Africa; country: Tanzania; stateProvince: Shinyanga; locality: Naabi Hill; verbatimLocality: 9 km south -east of Naabi Hill.; minimumElevationInMeters: 1500; decimalLatitude: -2.333333; decimalLongitude: 34.833333; **Event:** eventDate: 1969-03-26; **Record Level:** institutionCode: WAG; collectionCode: Herbarium; ownerInstitutionCode: WAG; basisOfRecord: PreservedSpecimen
- **Type status:** Other material. **Occurrence:** catalogNumber: DB0252; recordNumber: 10621; recordedBy: Greenway, PJ; Tanner, M; Watson; **Taxon:** scientificName: *Panicum atrosanguineum* Hochst. ex A.Rich.; kingdom: Plantae; family: Poaceae; genus: Panicum; specificEpithet: atrosanguineum; scientificNameAuthorship: Hochst. ex A.Rich.; **Location:** continent: Africa; country: Tanzania; stateProvince: Shinyanga; locality: Kitu Hill; verbatimLocality: Duma river - Mamerehe Guard Post, S.E. of Kitu Hill; minimumElevationInMeters: 1219; decimalLatitude: -2.45; decimalLongitude: 34.316667; **Event:** eventDate: 1962-04-20; **Record Level:** institutionCode: SWRC; collectionCode: Herbarium; ownerInstitutionCode: SWRC; basisOfRecord: PreservedSpecimen
- **Type status:** Other material. **Occurrence:** catalogNumber: DB0253; recordNumber: 10081; recordedBy: Greenway, PJ; Tanner, M; **Taxon:** scientificName: *Panicum atrosanguineum* Hochst. ex A.Rich.; kingdom: Plantae; family: Poaceae; genus: Panicum; specificEpithet: atrosanguineum; scientificNameAuthorship: Hochst. ex A.Rich.; **Location:** continent: Africa; country: Tanzania; stateProvince: Shinyanga; locality: Lake Magadi; verbatimLocality: T1, N. of lake Magadi on the Seronera track.; minimumElevationInMeters: 1380; decimalLatitude: -2.75; decimalLongitude: 34.833333; **Event:** eventDate: 1961-04-20; **Record Level:** institutionCode: SWRC; collectionCode: Herbarium; ownerInstitutionCode: SWRC; basisOfRecord: PreservedSpecimen
- **Type status:** Other material. **Occurrence:** catalogNumber: DB0254; recordNumber: 79; recordedBy: Belsky, PJ; **Taxon:** scientificName: *Panicum atrosanguineum* Hochst. ex A.Rich.; kingdom: Plantae; family: Poaceae; genus: Panicum; specificEpithet: atrosanguineum; scientificNameAuthorship: Hochst. ex A.Rich.; **Location:** continent: Africa; country: Tanzania; stateProvince: Mara; county: Serengeti; locality: Lake Lagarja; verbatimLocality: 10 km north of lake Lagarje; decimalLatitude: -3; decimalLongitude: 35.033333; **Event:** eventDate: 1981-04-10; **Record Level:** institutionCode: SWRC; collectionCode: Herbarium; ownerInstitutionCode: SWRC; basisOfRecord: PreservedSpecimen
- **Type status:** Other material. **Occurrence:** catalogNumber: DB0378; recordNumber: 128; recordedBy: Schmidt, W; **Taxon:** scientificName: *Panicum atrosanguineum* Hochst. ex A.Rich.; kingdom: Plantae; family: Poaceae; genus: Panicum; specificEpithet: atrosanguineum; scientificNameAuthorship: Hochst. ex A.Rich.; **Location:** continent: Africa; country: Tanzania; stateProvince: Mara; county: Serengeti; locality: Serengeti Plains; verbatimLocality: Naabi south Serengeti Plains; decimalLatitude: -2.333333; decimalLongitude: 34.833333; **Event:** eventDate: 1972-03-16; **Record Level:** institutionCode: SWRC; collectionCode: Herbarium; ownerInstitutionCode: SWRC; basisOfRecord: PreservedSpecimen
- **Type status:** Other material. **Occurrence:** catalogNumber: 1124; recordNumber: 858A; recordedBy: Mollel, NP; Rusch, GM; Mwakalebe, G; **Taxon:** scientificName: *Panicum atrosanguineum* Hochst. ex A.Rich.; kingdom: Plantae; family: Poaceae; genus: Panicum; specificEpithet: atrosanguineum; scientificNameAuthorship: Hochst. ex A.Rich.; **Location:** continent: Africa; country: Tanzania; stateProvince: Mara; county: Serengeti; locality: Robanda village; verbatimLocality: ca. 5km from the Serengeti National Park boundary towards the west. Near Robanda hill. 36M, 0689110, 97634640 UTM.; minimumElevationInMeters: 1416; decimalLatitude: -2.083333; decimalLongitude: 34.666667; **Event:** eventDate: 2003-01-28; **Record Level:** institutionCode: NHT; collectionCode: Herbarium; ownerInstitutionCode: NHT; basisOfRecord: PreservedSpecimen

##### Distribution

Widespread

#### Panicum calvum

Stapf

##### Materials

- **Type status:** Other material. **Occurrence:** catalogNumber: K000984152; recordNumber: 89038C; recordedBy: Pocs, T; Chuwa, S; **Taxon:** scientificName: *Panicum calvum* Stapf; kingdom: Plantae; family: Poaceae; genus: Panicum; specificEpithet: calvum; scientificNameAuthorship: Stapf; **Location:** continent: Africa; country: Tanzania; stateProvince: Arusha; county: Ngorongoro; locality: Ngorongoro; verbatimLocality: 3 km from Crater view point; minimumElevationInMeters: 2250; decimalLatitude: -3.166667; decimalLongitude: 35.583333; **Event:** eventDate: 1989-01-24; **Record Level:** institutionCode: K; collectionCode: Herbarium; ownerInstitutionCode: K; basisOfRecord: PreservedSpecimen

##### Distribution

Tropical Africa

#### Panicum chionachne

Mez

##### Materials

- **Type status:** Other material. **Occurrence:** catalogNumber: DB0267; recordNumber: 7606; recordedBy: Vesey-FitzGerald, LDEF; **Taxon:** scientificName: *Panicum chionachne* Mez; kingdom: Plantae; family: Poaceae; genus: Panicum; specificEpithet: chionachne; scientificNameAuthorship: Mez; **Location:** continent: Africa; country: Tanzania; stateProvince: Arusha; county: Ngorongoro; locality: Empakai Crater; verbatimLocality: Empakai Crater east rim; minimumElevationInMeters: 2460; decimalLatitude: -2.933333; decimalLongitude: 35.816667; **Event:** eventDate: 1973-11-13; **Record Level:** institutionCode: SWRC; collectionCode: Herbarium; ownerInstitutionCode: SWRC; basisOfRecord: PreservedSpecimen
- **Type status:** Other material. **Occurrence:** catalogNumber: DB0268; recordNumber: 181; recordedBy: Frame, GW; **Taxon:** scientificName: *Panicum chionachne* Mez; kingdom: Plantae; family: Poaceae; genus: Panicum; specificEpithet: chionachne; scientificNameAuthorship: Mez; **Location:** continent: Africa; country: Tanzania; stateProvince: Arusha; county: Ngorongoro; locality: Empakai Crater; verbatimLocality: Empakaai Crater, Inside east rim elevation.; minimumElevationInMeters: 2350; decimalLatitude: -2.933333; decimalLongitude: 35.816667; **Event:** eventDate: 1973-06-22; **Record Level:** institutionCode: SWRC; collectionCode: Herbarium; ownerInstitutionCode: SWRC; basisOfRecord: PreservedSpecimen
- **Type status:** Other material. **Occurrence:** catalogNumber: 329; recordNumber: 181; recordedBy: Frame, GW; **Taxon:** scientificName: *Panicum chionachne* Mez; kingdom: Plantae; family: Poaceae; genus: Panicum; specificEpithet: chionachne; scientificNameAuthorship: Mez; **Location:** continent: Africa; country: Tanzania; stateProvince: Arusha; county: Ngorongoro; locality: Empakai Crater; verbatimLocality: Empakaai Crater, Inside east rim elevation.; minimumElevationInMeters: 2350; decimalLatitude: -2.933333; decimalLongitude: 35.816667; **Event:** eventDate: 1973-06-22; **Record Level:** institutionCode: EA; collectionCode: Herbarium; ownerInstitutionCode: EA; basisOfRecord: PreservedSpecimen

##### Distribution

Tropical Africa

#### Panicum coloratum

L.

Panicum coloratum L. var. minus Stapf ex Chiov.

##### Materials

- **Type status:** Other material. **Occurrence:** catalogNumber: K000984132; recordNumber: 208; recordedBy: Oteke, J; **Taxon:** scientificName: *Panicum coloratum* L.; kingdom: Plantae; family: Poaceae; genus: Panicum; specificEpithet: coloratum; scientificNameAuthorship: L.; **Location:** continent: Africa; country: Tanzania; stateProvince: Arusha; county: Ngorongoro; locality: Lemuta; verbatimLocality: E. of Serengeti; minimumElevationInMeters: 1585; decimalLatitude: -2.7; decimalLongitude: 35.266667; **Event:** eventDate: 1962-07-23; **Record Level:** institutionCode: K; collectionCode: Herbarium; ownerInstitutionCode: K; basisOfRecord: PreservedSpecimen
- **Type status:** Other material. **Occurrence:** catalogNumber: K000984135; recordNumber: 10187; recordedBy: Greenway, PJ; **Taxon:** scientificName: *Panicum coloratum* L.; kingdom: Plantae; family: Poaceae; genus: Panicum; specificEpithet: coloratum; scientificNameAuthorship: L.; **Location:** continent: Africa; country: Tanzania; stateProvince: Mara; county: Serengeti; locality: Seronera; verbatimLocality: W. of Seronera, Serengeti; minimumElevationInMeters: 1463; decimalLatitude: -2.45; decimalLongitude: 34.833333; **Event:** eventDate: 1961-05-15; **Record Level:** institutionCode: K; collectionCode: Herbarium; ownerInstitutionCode: K; basisOfRecord: PreservedSpecimen
- **Type status:** Other material. **Occurrence:** catalogNumber: K000984136; recordNumber: 369; recordedBy: Paulo, S; **Taxon:** scientificName: *Panicum coloratum* L.; kingdom: Plantae; family: Poaceae; genus: Panicum; specificEpithet: coloratum; scientificNameAuthorship: L.; **Location:** continent: Africa; country: Tanzania; stateProvince: Shinyanga; locality: Naabi; verbatimLocality: N.W. of Naabi; decimalLatitude: -2.933333; decimalLongitude: 35.083333; **Event:** eventDate: 1958-04-24; **Record Level:** institutionCode: K; collectionCode: Herbarium; ownerInstitutionCode: K; basisOfRecord: PreservedSpecimen
- **Type status:** Other material. **Occurrence:** catalogNumber: K000984137; recordNumber: 2583; recordedBy: Chuwa, S; **Taxon:** scientificName: *Panicum coloratum* L.; kingdom: Plantae; family: Poaceae; genus: Panicum; specificEpithet: coloratum; scientificNameAuthorship: L.; **Location:** continent: Africa; country: Tanzania; stateProvince: Arusha; county: Ngorongoro; locality: Esere; verbatimLocality: near Kakesio; minimumElevationInMeters: 1600; decimalLatitude: -3.316667; decimalLongitude: 35.033333; **Event:** eventDate: 1987-04-28; **Record Level:** institutionCode: K; collectionCode: Herbarium; ownerInstitutionCode: K; basisOfRecord: PreservedSpecimen
- **Type status:** Other material. **Occurrence:** catalogNumber: K000984138; recordNumber: 10505; recordedBy: Greenway, PJ; **Taxon:** scientificName: *Panicum coloratum* L.; kingdom: Plantae; family: Poaceae; genus: Panicum; specificEpithet: coloratum; scientificNameAuthorship: L.; **Location:** continent: Africa; country: Tanzania; stateProvince: Shinyanga; locality: Lake Magadi; verbatimLocality: South of Lake Magadi, Mile 10.6 from Seronera, Serengeti; minimumElevationInMeters: 1463; decimalLatitude: -2.633333; decimalLongitude: 34.9; **Event:** eventDate: 1962-03-06; **Record Level:** institutionCode: K; collectionCode: Herbarium; ownerInstitutionCode: K; basisOfRecord: PreservedSpecimen
- **Type status:** Other material. **Occurrence:** catalogNumber: DB0276; recordNumber: 10505; recordedBy: Greenway, PJ; **Taxon:** scientificName: *Panicum coloratum* L.; kingdom: Plantae; family: Poaceae; genus: Panicum; specificEpithet: coloratum; scientificNameAuthorship: L.; **Location:** continent: Africa; country: Tanzania; stateProvince: Shinyanga; locality: Lake Magadi; verbatimLocality: South of Lake Magadi, Mile 10.6 from Seronera, Serengeti; minimumElevationInMeters: 1463; decimalLatitude: -2.633333; decimalLongitude: 34.9; **Event:** eventDate: 1962-03-06; **Record Level:** institutionCode: SWRC; collectionCode: Herbarium; ownerInstitutionCode: SWRC; basisOfRecord: PreservedSpecimen
- **Type status:** Other material. **Occurrence:** catalogNumber: DB0277; recordNumber: 228; recordedBy: Schmidt, W; **Taxon:** scientificName: *Panicum coloratum* L.; kingdom: Plantae; family: Poaceae; genus: Panicum; specificEpithet: coloratum; scientificNameAuthorship: L.; **Location:** continent: Africa; country: Tanzania; stateProvince: Shinyanga; locality: Naabi Hill; verbatimLocality: Naabi Hill (North) Serengeti Plains.; decimalLatitude: -2.883333; decimalLongitude: 35.033333; **Event:** eventDate: 1972-03-22; **Record Level:** institutionCode: SWRC; collectionCode: Herbarium; ownerInstitutionCode: SWRC; basisOfRecord: PreservedSpecimen
- **Type status:** Other material. **Occurrence:** catalogNumber: DB0278; recordNumber: 166; recordedBy: Schmidt, W; **Taxon:** scientificName: *Panicum coloratum* L.; kingdom: Plantae; family: Poaceae; genus: Panicum; specificEpithet: coloratum; scientificNameAuthorship: L.; **Location:** continent: Africa; country: Tanzania; stateProvince: Mara; county: Serengeti; locality: Serengeti Plains; verbatimLocality: Simba North Serengeti Plains; decimalLatitude: -2.333333; decimalLongitude: 34.833333; **Event:** eventDate: 1972-03-17; **Record Level:** institutionCode: SWRC; collectionCode: Herbarium; ownerInstitutionCode: SWRC; basisOfRecord: PreservedSpecimen
- **Type status:** Other material. **Occurrence:** catalogNumber: DB0279; recordNumber: 237; recordedBy: Belsky, PJ; **Taxon:** scientificName: *Panicum coloratum* L.; kingdom: Plantae; family: Poaceae; genus: Panicum; specificEpithet: coloratum; scientificNameAuthorship: L.; **Location:** continent: Africa; country: Tanzania; stateProvince: Mara; county: Serengeti; locality: Lobo Lodge; verbatimLocality: 15 km south of Lobo Lodge; decimalLatitude: -2.116667; decimalLongitude: 35.166667; **Event:** eventDate: 1981-5; **Record Level:** institutionCode: SWRC; collectionCode: Herbarium; ownerInstitutionCode: SWRC; basisOfRecord: PreservedSpecimen
- **Type status:** Other material. **Occurrence:** catalogNumber: DB0280; recordNumber: 212; recordedBy: Belsky, PJ; **Taxon:** scientificName: *Panicum coloratum* L.; kingdom: Plantae; family: Poaceae; genus: Panicum; specificEpithet: coloratum; scientificNameAuthorship: L.; **Location:** continent: Africa; country: Tanzania; stateProvince: Mara; county: Serengeti; locality: Serengeti Research Institute; verbatimLocality: SRI Compound; decimalLatitude: -2.433333; decimalLongitude: 34.85; **Event:** eventDate: 1981-05-11; **Record Level:** institutionCode: SWRC; collectionCode: Herbarium; ownerInstitutionCode: SWRC; basisOfRecord: PreservedSpecimen
- **Type status:** Other material. **Occurrence:** catalogNumber: DB0281; recordNumber: 10187; recordedBy: Greenway, PJ; **Taxon:** scientificName: *Panicum coloratum* L.; kingdom: Plantae; family: Poaceae; genus: Panicum; specificEpithet: coloratum; scientificNameAuthorship: L.; **Location:** continent: Africa; country: Tanzania; stateProvince: Mara; county: Serengeti; locality: Seronera; verbatimLocality: W. of Seronera, Serengeti; minimumElevationInMeters: 1463; decimalLatitude: -2.45; decimalLongitude: 34.833333; **Event:** eventDate: 1961-05-15; **Record Level:** institutionCode: SWRC; collectionCode: Herbarium; ownerInstitutionCode: SWRC; basisOfRecord: PreservedSpecimen
- **Type status:** Other material. **Occurrence:** catalogNumber: DB0282; recordNumber: 244; recordedBy: Belsky, PJ; **Taxon:** scientificName: *Panicum coloratum* L.; kingdom: Plantae; family: Poaceae; genus: Panicum; specificEpithet: coloratum; scientificNameAuthorship: L.; **Location:** continent: Africa; country: Tanzania; stateProvince: Mara; county: Serengeti; locality: Serengeti Research Institute; verbatimLocality: SRI compound; decimalLatitude: -2.433333; decimalLongitude: 34.85; **Event:** eventDate: 1981-05-11; **Record Level:** institutionCode: SWRC; collectionCode: Herbarium; ownerInstitutionCode: SWRC; basisOfRecord: PreservedSpecimen
- **Type status:** Other material. **Occurrence:** catalogNumber: DB0283; recordNumber: 9862; recordedBy: Greenway, PJ; **Taxon:** scientificName: *Panicum coloratum* L.; kingdom: Plantae; family: Poaceae; genus: Panicum; specificEpithet: coloratum; scientificNameAuthorship: L.; **Location:** continent: Africa; country: Tanzania; stateProvince: Mara; county: Serengeti; locality: Seronera; verbatimLocality: T1, Seronera, Serengeti; minimumElevationInMeters: 1530; decimalLatitude: -2.45; decimalLongitude: 34.833333; **Event:** eventDate: 1961-03-20; **Record Level:** institutionCode: SWRC; collectionCode: Herbarium; ownerInstitutionCode: SWRC; basisOfRecord: PreservedSpecimen
- **Type status:** Other material. **Occurrence:** catalogNumber: DB0294; recordNumber: 365; recordedBy: Braun, MH; **Taxon:** scientificName: *Panicum coloratum* L.; kingdom: Plantae; family: Poaceae; genus: Panicum; specificEpithet: coloratum; scientificNameAuthorship: L.; **Location:** continent: Africa; country: Tanzania; stateProvince: Mara; county: Serengeti; locality: Serengeti National Park; verbatimLocality: Serengeti National park. Zebra Kopjes; decimalLatitude: -2.833333; decimalLongitude: 34.833333; **Event:** eventDate: 1970-02-10; **Record Level:** institutionCode: SWRC; collectionCode: Herbarium; ownerInstitutionCode: SWRC; basisOfRecord: PreservedSpecimen
- **Type status:** Other material. **Occurrence:** catalogNumber: K000984133; recordNumber: 10529; recordedBy: Greenway, PJ; Tanner, M; **Taxon:** scientificName: Panicum coloratum L. var. minus Stapf ex Chiov.; kingdom: Plantae; family: Poaceae; genus: Panicum; specificEpithet: coloratum; infraspecificEpithet: minus; scientificNameAuthorship: Stapf ex Chiov.; **Location:** continent: Africa; country: Tanzania; stateProvince: Shinyanga; locality: Naabi Hill; verbatimLocality: Central plains, N.E. of Naabi hill; minimumElevationInMeters: 1646; decimalLatitude: -2.883333; decimalLongitude: 35.033333; **Event:** eventDate: 1962-03-15; **Record Level:** institutionCode: K; collectionCode: Herbarium; ownerInstitutionCode: K; basisOfRecord: PreservedSpecimen
- **Type status:** Other material. **Occurrence:** catalogNumber: K000984134; recordNumber: 89033; recordedBy: Pocs, T; Chuwa, S; **Taxon:** scientificName: Panicum coloratum L. var. minus Stapf ex Chiov.; kingdom: Plantae; family: Poaceae; genus: Panicum; specificEpithet: coloratum; infraspecificEpithet: minus; scientificNameAuthorship: Stapf ex Chiov.; **Location:** continent: Africa; country: Tanzania; stateProvince: Arusha; county: Ngorongoro; locality: Kambi ya Nyoka; decimalLatitude: -3.3; decimalLongitude: 35.583333; **Event:** eventDate: 1989-01-21; **Record Level:** institutionCode: K; collectionCode: Herbarium; ownerInstitutionCode: K; basisOfRecord: PreservedSpecimen
- **Type status:** Other material. **Occurrence:** catalogNumber: DB0399; recordNumber: 10529; recordedBy: Greenway, PJ; Tanner, M; **Taxon:** scientificName: Panicum coloratum L. var. minus Stapf ex Chiov.; kingdom: Plantae; family: Poaceae; genus: Panicum; specificEpithet: coloratum; infraspecificEpithet: minus; scientificNameAuthorship: Stapf ex Chiov.; **Location:** continent: Africa; country: Tanzania; stateProvince: Shinyanga; locality: Naabi Hill; verbatimLocality: Central plains, N.E. of Naabi hill; minimumElevationInMeters: 1646; decimalLatitude: -2.883333; decimalLongitude: 35.033333; **Event:** eventDate: 1962-03-15; **Record Level:** institutionCode: SWRC; collectionCode: Herbarium; ownerInstitutionCode: SWRC; basisOfRecord: PreservedSpecimen

##### Distribution

Widespread

#### Panicum commutatum

Schult.

Panicum joorii Vasey

##### Materials

- **Type status:** Other material. **Occurrence:** catalogNumber: 652; recordNumber: 24263; recordedBy: Peterson, PM; Soreng, RJ; Romaschenko, K; Mbago, F; **Taxon:** scientificName: *Panicum joorii* Vasey; kingdom: Plantae; family: Poaceae; genus: Panicum; specificEpithet: joorii; scientificNameAuthorship: Vasey; **Location:** continent: Africa; country: Tanzania; stateProvince: Shinyanga; locality: Naabi Hill Gate; verbatimLocality: Serengeti National Park, Naabi Hill Gate (at 0.5 km N).; minimumElevationInMeters: 1734; decimalLatitude: -2.83139; decimalLongitude: 34.99672; **Event:** eventDate: 2012-06-16; **Record Level:** institutionCode: US; collectionCode: Herbarium; ownerInstitutionCode: US; basisOfRecord: PreservedSpecimen
- **Type status:** Other material. **Occurrence:** catalogNumber: 653; recordNumber: 24277; recordedBy: Peterson, PM; Soreng, RJ; Romaschenko, K; Mbago, F; **Taxon:** scientificName: *Panicum joorii* Vasey; kingdom: Plantae; family: Poaceae; genus: Panicum; specificEpithet: joorii; scientificNameAuthorship: Vasey; **Location:** continent: Africa; country: Tanzania; stateProvince: Shinyanga; locality: Naabi Hill Gate; verbatimLocality: Serengeti National Park, at 12 km NW of Naabi Hill Gate.; minimumElevationInMeters: 1651; decimalLatitude: -2.73597; decimalLongitude: 34.95284; **Event:** eventDate: 2012-06-16; **Record Level:** institutionCode: US; collectionCode: Herbarium; ownerInstitutionCode: US; basisOfRecord: PreservedSpecimen
- **Type status:** Other material. **Occurrence:** catalogNumber: 654; recordNumber: 24278; recordedBy: Peterson, PM; Soreng, RJ; Romaschenko, K; Mbago, F; **Taxon:** scientificName: *Panicum joorii* Vasey; kingdom: Plantae; family: Poaceae; genus: Panicum; specificEpithet: joorii; scientificNameAuthorship: Vasey; **Location:** continent: Africa; country: Tanzania; stateProvince: Shinyanga; locality: Naabi Hill Gate; verbatimLocality: Serengeti National Park, at 12 km NW of Naabi Hill Gate.; minimumElevationInMeters: 1651; decimalLatitude: -2.73597; decimalLongitude: 34.95284; **Event:** eventDate: 2012-06-16; **Record Level:** institutionCode: US; collectionCode: Herbarium; ownerInstitutionCode: US; basisOfRecord: PreservedSpecimen

##### Distribution

North & South America

#### Panicum deustum

Thunb.

##### Materials

- **Type status:** Other material. **Occurrence:** catalogNumber: DB0296; recordNumber: 522; recordedBy: Schmidt, W; **Taxon:** scientificName: *Panicum deustum* Thunb.; kingdom: Plantae; family: Poaceae; genus: Panicum; specificEpithet: deustum; scientificNameAuthorship: Thunb.; **Location:** continent: Africa; country: Tanzania; stateProvince: Mara; county: Serengeti; locality: Bolgonja; verbatimLocality: Bologonja; decimalLatitude: -1.783333; decimalLongitude: 35.2; **Event:** eventDate: 1972-04-13; **Record Level:** institutionCode: SWRC; collectionCode: Herbarium; ownerInstitutionCode: SWRC; basisOfRecord: PreservedSpecimen
- **Type status:** Other material. **Occurrence:** catalogNumber: DB0297; recordNumber: 418; recordedBy: Schmidt, W; **Taxon:** scientificName: *Panicum deustum* Thunb.; kingdom: Plantae; family: Poaceae; genus: Panicum; specificEpithet: deustum; scientificNameAuthorship: Thunb.; **Location:** continent: Africa; country: Tanzania; stateProvince: Mara; county: Serengeti; locality: Bolgonja; verbatimLocality: Bologonja; decimalLatitude: -1.783333; decimalLongitude: 35.2; **Event:** eventDate: 1972-04-12; **Record Level:** institutionCode: SWRC; collectionCode: Herbarium; ownerInstitutionCode: SWRC; basisOfRecord: PreservedSpecimen
- **Type status:** Other material. **Occurrence:** catalogNumber: DB0298; recordNumber: 523; recordedBy: Schmidt, W; **Taxon:** scientificName: *Panicum deustum* Thunb.; kingdom: Plantae; family: Poaceae; genus: Panicum; specificEpithet: deustum; scientificNameAuthorship: Thunb.; **Location:** continent: Africa; country: Tanzania; stateProvince: Mara; county: Serengeti; locality: Bolgonja; verbatimLocality: Bologonja; decimalLatitude: -1.783333; decimalLongitude: 35.2; **Event:** eventDate: 1972-04-13; **Record Level:** institutionCode: SWRC; collectionCode: Herbarium; ownerInstitutionCode: SWRC; basisOfRecord: PreservedSpecimen
- **Type status:** Other material. **Occurrence:** catalogNumber: DB0304; recordNumber: s.n.; recordedBy: Unknown; **Taxon:** scientificName: *Panicum deustum* Thunb.; kingdom: Plantae; family: Poaceae; genus: Panicum; specificEpithet: deustum; scientificNameAuthorship: Thunb.; **Location:** continent: Africa; country: Tanzania; stateProvince: Mara; county: Serengeti; locality: Seronera; verbatimLocality: Seronera Kopjes; decimalLatitude: -2.45; decimalLongitude: 34.833333; **Event:** eventDate: 1971-06-22; **Record Level:** institutionCode: SWRC; collectionCode: Herbarium; ownerInstitutionCode: SWRC; basisOfRecord: PreservedSpecimen
- **Type status:** Other material. **Occurrence:** catalogNumber: 651; recordNumber: 24311; recordedBy: Peterson, PM; Soreng, RJ; Romaschenko, K; Mbago, F; **Taxon:** scientificName: *Panicum deustum* Thunb.; kingdom: Plantae; family: Poaceae; genus: Panicum; specificEpithet: deustum; scientificNameAuthorship: Thunb.; **Location:** continent: Africa; country: Tanzania; stateProvince: Arusha; county: Ngorongoro; locality: Ngorongoro Crater; verbatimLocality: Ngorongoro Conservation Area, rim of Ngorongoro Crater (descent gate).; minimumElevationInMeters: 2168; decimalLatitude: -3.15462; decimalLongitude: 35.47717; **Event:** eventDate: 2012-06-19; **Record Level:** institutionCode: US; collectionCode: Herbarium; ownerInstitutionCode: US; basisOfRecord: PreservedSpecimen

##### Distribution

Tropical Africa & Afghanistan

#### Panicum humile

Steud.

Panicum watense Mez

##### Materials

- **Type status:** Other material. **Occurrence:** catalogNumber: 658; recordNumber: 24304; recordedBy: Peterson, PM; Soreng, RJ; Romaschenko, K; Mbago, F; **Taxon:** scientificName: *Panicum watense* Mez; kingdom: Plantae; family: Poaceae; genus: Panicum; specificEpithet: watense; scientificNameAuthorship: Mez; **Location:** continent: Africa; country: Tanzania; stateProvince: Arusha; county: Ngorongoro; locality: Ngorongoro Crater; verbatimLocality: Ngorongoro Conservation Area, rim of Ngorongoro Crater (descent gate).; minimumElevationInMeters: 2168; decimalLatitude: -3.15462; decimalLongitude: 35.47717; **Event:** eventDate: 2012-06-19; **Record Level:** institutionCode: US; collectionCode: Herbarium; ownerInstitutionCode: US; basisOfRecord: PreservedSpecimen

##### Distribution

Widespread

#### Panicum hygrocharis

Steud.

##### Materials

- **Type status:** Other material. **Occurrence:** catalogNumber: K000984139; recordNumber: 10599; recordedBy: Greenway, PJ; Tanner, M; **Taxon:** scientificName: *Panicum hygrocharis* Steud.; kingdom: Plantae; family: Poaceae; genus: Panicum; specificEpithet: hygrocharis; scientificNameAuthorship: Steud.; **Location:** continent: Africa; country: Tanzania; stateProvince: Mara; county: Serengeti; locality: Seronera river; verbatimLocality: below Sid Downeys Dam; minimumElevationInMeters: 1432; decimalLatitude: -2.433333; decimalLongitude: 34.816667; **Event:** eventDate: 1962-04-15; **Record Level:** institutionCode: K; collectionCode: Herbarium; ownerInstitutionCode: K; basisOfRecord: PreservedSpecimen
- **Type status:** Other material. **Occurrence:** catalogNumber: K000984140; recordNumber: 12573; recordedBy: Greenway, PJ; Kanuri; **Taxon:** scientificName: *Panicum hygrocharis* Steud.; kingdom: Plantae; family: Poaceae; genus: Panicum; specificEpithet: hygrocharis; scientificNameAuthorship: Steud.; **Location:** continent: Africa; country: Tanzania; stateProvince: Arusha; county: Ngorongoro; locality: Munge Swamp; verbatimLocality: N. W. side of Ngorongoro Crater floor; minimumElevationInMeters: 1661; decimalLatitude: -3.083333; decimalLongitude: 35.666667; **Event:** eventDate: 1966-07-14; **Record Level:** institutionCode: K; collectionCode: Herbarium; ownerInstitutionCode: K; basisOfRecord: PreservedSpecimen
- **Type status:** Other material. **Occurrence:** catalogNumber: K000984141; recordNumber: 10354; recordedBy: Greenway, PJ; **Taxon:** scientificName: *Panicum hygrocharis* Steud.; kingdom: Plantae; family: Poaceae; genus: Panicum; specificEpithet: hygrocharis; scientificNameAuthorship: Steud.; **Location:** continent: Africa; country: Tanzania; stateProvince: Mara; county: Serengeti; locality: Seronera; verbatimLocality: Seronera to Naabi hill, mile 15.3; minimumElevationInMeters: 1585; decimalLatitude: -2.6; decimalLongitude: 34.983333; **Event:** eventDate: 1961-06-03; **Record Level:** institutionCode: K; collectionCode: Herbarium; ownerInstitutionCode: K; basisOfRecord: PreservedSpecimen
- **Type status:** Other material. **Occurrence:** catalogNumber: K000984142; recordNumber: 6252; recordedBy: Newbould, JB; **Taxon:** scientificName: *Panicum hygrocharis* Steud.; kingdom: Plantae; family: Poaceae; genus: Panicum; specificEpithet: hygrocharis; scientificNameAuthorship: Steud.; **Location:** continent: Africa; country: Tanzania; stateProvince: Arusha; county: Ngorongoro; locality: Lerai stream; verbatimLocality: Ngorongoro Crater; minimumElevationInMeters: 1676; decimalLatitude: -3.216667; decimalLongitude: 35.5; **Event:** eventDate: 1962-07-31; **Record Level:** institutionCode: K; collectionCode: Herbarium; ownerInstitutionCode: K; basisOfRecord: PreservedSpecimen
- **Type status:** Other material. **Occurrence:** catalogNumber: DB0401; recordNumber: 10599; recordedBy: Greenway, PJ; Tanner, M; **Taxon:** scientificName: *Panicum hygrocharis* Steud.; kingdom: Plantae; family: Poaceae; genus: Panicum; specificEpithet: hygrocharis; scientificNameAuthorship: Steud.; **Location:** continent: Africa; country: Tanzania; stateProvince: Mara; county: Serengeti; locality: Seronera river; verbatimLocality: below Sid Downeys Dam; minimumElevationInMeters: 1432; decimalLatitude: -2.433333; decimalLongitude: 34.816667; **Event:** eventDate: 1962-04-15; **Record Level:** institutionCode: SWRC; collectionCode: Herbarium; ownerInstitutionCode: SWRC; basisOfRecord: PreservedSpecimen
- **Type status:** Other material. **Occurrence:** catalogNumber: DB0400; recordNumber: 10354; recordedBy: Greenway, PJ; **Taxon:** scientificName: *Panicum hygrocharis* Steud.; kingdom: Plantae; family: Poaceae; genus: Panicum; specificEpithet: hygrocharis; scientificNameAuthorship: Steud.; **Location:** continent: Africa; country: Tanzania; stateProvince: Mara; county: Serengeti; locality: Seronera; verbatimLocality: Seronera to Naabi hill, mile 15.3; minimumElevationInMeters: 1585; decimalLatitude: -2.6; decimalLongitude: 34.983333; **Event:** eventDate: 1961-06-03; **Record Level:** institutionCode: SWRC; collectionCode: Herbarium; ownerInstitutionCode: SWRC; basisOfRecord: PreservedSpecimen
- **Type status:** Other material. **Occurrence:** catalogNumber: DB0396; recordNumber: 4770; recordedBy: Vesey-FitzGerald, LDEF; **Taxon:** scientificName: *Panicum hygrocharis* Steud.; kingdom: Plantae; family: Poaceae; genus: Panicum; specificEpithet: hygrocharis; scientificNameAuthorship: Steud.; **Location:** continent: Africa; country: Tanzania; stateProvince: Arusha; county: Ngorongoro; locality: Ngorongoro; minimumElevationInMeters: 1500; decimalLatitude: -3; decimalLongitude: 35.5; **Event:** eventDate: 1965-07-27; **Record Level:** institutionCode: SWRC; collectionCode: Herbarium; ownerInstitutionCode: SWRC; basisOfRecord: PreservedSpecimen
- **Type status:** Other material. **Occurrence:** catalogNumber: DB0391; recordNumber: 321; recordedBy: Kreulen, AR; **Taxon:** scientificName: *Panicum hygrocharis* Steud.; kingdom: Plantae; family: Poaceae; genus: Panicum; specificEpithet: hygrocharis; scientificNameAuthorship: Steud.; **Location:** continent: Africa; country: Tanzania; stateProvince: Mara; county: Serengeti; locality: Serengeti; verbatimLocality: Serengeti; decimalLatitude: -2.333333; decimalLongitude: 34.833333; **Event:** eventDate: 1974-05-04; **Record Level:** institutionCode: SWRC; collectionCode: Herbarium; ownerInstitutionCode: SWRC; basisOfRecord: PreservedSpecimen

##### Distribution

Tropical Africa

#### Panicum hymeniochilum

Nees

##### Materials

- **Type status:** Other material. **Occurrence:** catalogNumber: K000984145; recordNumber: 2471; recordedBy: Chuwa, S; **Taxon:** scientificName: *Panicum hymeniochilum* Nees; kingdom: Plantae; family: Poaceae; genus: Panicum; specificEpithet: hymeniochilum; scientificNameAuthorship: Nees; **Location:** continent: Africa; country: Tanzania; stateProvince: Arusha; county: Ngorongoro; locality: Oldeani Mt; verbatimLocality: Ngorongoro Conservation area, Oldean Mountain; decimalLatitude: -3.266667; decimalLongitude: 35.433333; **Event:** eventDate: 1983-04-19; **Record Level:** institutionCode: K; collectionCode: Herbarium; ownerInstitutionCode: K; basisOfRecord: PreservedSpecimen

##### Distribution

Tropical Africa

#### Panicum infestum

Andersson

##### Materials

- **Type status:** Other material. **Occurrence:** catalogNumber: DB0320; recordNumber: 4658; recordedBy: Vesey-FitzGerald, LDEF; **Taxon:** scientificName: *Panicum infestum* Andersson; kingdom: Plantae; family: Poaceae; genus: Panicum; specificEpithet: infestum; scientificNameAuthorship: Andersson; **Location:** continent: Africa; country: Tanzania; stateProvince: Mara; county: Serengeti; locality: Kirawira; verbatimLocality: Serengeti National Park. Kiriwira; decimalLatitude: -2.166667; decimalLongitude: 34.15; **Event:** eventDate: 1965-04-27; **Record Level:** institutionCode: SWRC; collectionCode: Herbarium; ownerInstitutionCode: SWRC; basisOfRecord: PreservedSpecimen

##### Distribution

Tropical Africa

#### Panicum massaiense

Stapf

##### Materials

- **Type status:** Other material. **Occurrence:** catalogNumber: K000984149; recordNumber: 10551; recordedBy: Greenway, PJ; Tanner, M; Harvey, G; **Taxon:** scientificName: *Panicum massaiense* Stapf; kingdom: Plantae; family: Poaceae; genus: Panicum; specificEpithet: massaiense; scientificNameAuthorship: Stapf; **Location:** continent: Africa; country: Tanzania; stateProvince: Shinyanga; locality: Beacon Area; verbatimLocality: S.W. Beacon area; minimumElevationInMeters: 1524; decimalLatitude: -2.083333; decimalLongitude: 34.333333; **Event:** eventDate: 1962-03-27; **Record Level:** institutionCode: K; collectionCode: Herbarium; ownerInstitutionCode: K; basisOfRecord: PreservedSpecimen
- **Type status:** Other material. **Occurrence:** catalogNumber: K000984150; recordNumber: 417; recordedBy: Paulo, S; **Taxon:** scientificName: *Panicum massaiense* Stapf; kingdom: Plantae; family: Poaceae; genus: Panicum; specificEpithet: massaiense; scientificNameAuthorship: Stapf; **Location:** continent: Africa; country: Tanzania; stateProvince: Shinyanga; locality: Naabi Hill; verbatimLocality: S.W. of Naabi Hill; decimalLatitude: -2.883333; decimalLongitude: 35.033333; **Event:** eventDate: 1958-05-03; **Record Level:** institutionCode: K; collectionCode: Herbarium; ownerInstitutionCode: K; basisOfRecord: PreservedSpecimen
- **Type status:** Other material. **Occurrence:** catalogNumber: K000984151; recordNumber: 10371; recordedBy: Greenway, PJ; Tanner, M; **Taxon:** scientificName: *Panicum massaiense* Stapf; kingdom: Plantae; family: Poaceae; genus: Panicum; specificEpithet: massaiense; scientificNameAuthorship: Stapf; **Location:** continent: Africa; country: Tanzania; stateProvince: Shinyanga; locality: Mbono river; verbatimLocality: Mbono and Simiyu river junctions; minimumElevationInMeters: 1387; decimalLatitude: -3.116667; decimalLongitude: 34.833333; **Event:** eventDate: 1961-06-07; **Record Level:** institutionCode: K; collectionCode: Herbarium; ownerInstitutionCode: K; basisOfRecord: PreservedSpecimen
- **Type status:** Other material. **Occurrence:** catalogNumber: DB0330; recordNumber: 10371; recordedBy: Greenway, PJ; Tanner, M; **Taxon:** scientificName: *Panicum massaiense* Stapf; kingdom: Plantae; family: Poaceae; genus: Panicum; specificEpithet: massaiense; scientificNameAuthorship: Stapf; **Location:** continent: Africa; country: Tanzania; stateProvince: Shinyanga; locality: Mbono river; verbatimLocality: Mbono and Simiyu river junctions; minimumElevationInMeters: 1387; decimalLatitude: -3.116667; decimalLongitude: 34.833333; **Event:** eventDate: 1961-06-07; **Record Level:** institutionCode: SWRC; collectionCode: Herbarium; ownerInstitutionCode: SWRC; basisOfRecord: PreservedSpecimen
- **Type status:** Other material. **Occurrence:** catalogNumber: DB0373; recordNumber: 10551; recordedBy: Greenway, PJ; Tanner, M; Harvey, G; **Taxon:** scientificName: *Panicum massaiense* Stapf; kingdom: Plantae; family: Poaceae; genus: Panicum; specificEpithet: massaiense; scientificNameAuthorship: Stapf; **Location:** continent: Africa; country: Tanzania; stateProvince: Shinyanga; locality: Beacon Area; verbatimLocality: S.W. Beacon area; minimumElevationInMeters: 1524; decimalLatitude: -2.083333; decimalLongitude: 34.333333; **Event:** eventDate: 1962-03-27; **Record Level:** institutionCode: SWRC; collectionCode: Herbarium; ownerInstitutionCode: SWRC; basisOfRecord: PreservedSpecimen

##### Distribution

Tropical Africa

#### Panicum maximum

Jacq.

Megathyrsus maximus (Jacq.) B.K.Simon & S.W.L.Jacobs

##### Materials

- **Type status:** Other material. **Occurrence:** catalogNumber: DB0344; recordNumber: 371; recordedBy: Schmidt, W; **Taxon:** scientificName: *Megathyrsus maximus* (Jacq.) B.K.Simon & S.W.L.Jacobs; kingdom: Plantae; family: Poaceae; genus: Megathyrsus; specificEpithet: maximus; scientificNameAuthorship: (Jacq.) B.K.Simon & S.W.L.Jacobs; **Location:** continent: Africa; country: Tanzania; stateProvince: Mara; county: Serengeti; locality: Bolgonja; verbatimLocality: Bologonja; decimalLatitude: -1.783333; decimalLongitude: 35.2; **Event:** eventDate: 1972-04-11; **Record Level:** institutionCode: SWRC; collectionCode: Herbarium; ownerInstitutionCode: SWRC; basisOfRecord: PreservedSpecimen
- **Type status:** Other material. **Occurrence:** catalogNumber: DB0345; recordNumber: 5; recordedBy: Belsky, PJ; **Taxon:** scientificName: *Megathyrsus maximus* (Jacq.) B.K.Simon & S.W.L.Jacobs; kingdom: Plantae; family: Poaceae; genus: Megathyrsus; specificEpithet: maximus; scientificNameAuthorship: (Jacq.) B.K.Simon & S.W.L.Jacobs; **Location:** continent: Africa; country: Tanzania; stateProvince: Mara; county: Serengeti; locality: Serengeti Research Institute; verbatimLocality: Serengeti Research Institute; decimalLatitude: -2.433333; decimalLongitude: 34.85; **Event:** eventDate: 1980-01-21; **Record Level:** institutionCode: SWRC; collectionCode: Herbarium; ownerInstitutionCode: SWRC; basisOfRecord: PreservedSpecimen
- **Type status:** Other material. **Occurrence:** catalogNumber: DB0346; recordNumber: 226; recordedBy: Belsky, PJ; **Taxon:** scientificName: *Megathyrsus maximus* (Jacq.) B.K.Simon & S.W.L.Jacobs; kingdom: Plantae; family: Poaceae; genus: Megathyrsus; specificEpithet: maximus; scientificNameAuthorship: (Jacq.) B.K.Simon & S.W.L.Jacobs; **Location:** continent: Africa; country: Tanzania; stateProvince: Mara; county: Serengeti; locality: Serengeti; verbatimLocality: Serengeti, Sri Airstrip (Serengeti Research Institute); decimalLatitude: -2.45; decimalLongitude: 34.833333; **Event:** eventDate: 1981-05-15; **Record Level:** institutionCode: SWRC; collectionCode: Herbarium; ownerInstitutionCode: SWRC; basisOfRecord: PreservedSpecimen
- **Type status:** Other material. **Occurrence:** catalogNumber: 655; recordNumber: 817; recordedBy: Ellemann, L; **Taxon:** scientificName: *Panicum maximum* Jacq.; kingdom: Plantae; family: Poaceae; genus: Panicum; specificEpithet: maximum; scientificNameAuthorship: Jacq.; **Location:** continent: Africa; country: Tanzania; stateProvince: Arusha; county: Ngorongoro; locality: Nasiporiong; verbatimLocality: Ngorongoro Conservation Area, Nasiporiong'.; minimumElevationInMeters: 1800; decimalLatitude: -3.2; decimalLongitude: 35.2; **Event:** eventDate: 1993-08-19; **Record Level:** institutionCode: AAU; collectionCode: Herbarium; ownerInstitutionCode: AAU; basisOfRecord: PreservedSpecimen
- **Type status:** Other material. **Occurrence:** catalogNumber: 656; recordNumber: s.n.; recordedBy: Mboya, E; **Taxon:** scientificName: *Panicum maximum* Jacq.; kingdom: Plantae; family: Poaceae; genus: Panicum; specificEpithet: maximum; scientificNameAuthorship: Jacq.; **Location:** continent: Africa; country: Tanzania; stateProvince: Mara; county: Serengeti; locality: Serengeti Research Centre; verbatimLocality: T1. Serengeti Research Centre.; minimumElevationInMeters: 1550; decimalLatitude: -2.38; decimalLongitude: 34.85; **Event:** eventDate: 2004-02-09; **Record Level:** institutionCode: MO; collectionCode: Herbarium; ownerInstitutionCode: MO; basisOfRecord: PreservedSpecimen
- **Type status:** Other material. **Occurrence:** catalogNumber: 657; recordNumber: s.n.; recordedBy: Mboya, E; **Taxon:** scientificName: *Panicum maximum* Jacq.; kingdom: Plantae; family: Poaceae; genus: Panicum; specificEpithet: maximum; scientificNameAuthorship: Jacq.; **Location:** continent: Africa; country: Tanzania; stateProvince: Mara; county: Serengeti; locality: Serengeti Research Centre; verbatimLocality: T1. Serengeti Research Centre.; minimumElevationInMeters: 1550; decimalLatitude: -2.38; decimalLongitude: 34.85; **Event:** eventDate: 2004-02-09; **Record Level:** institutionCode: MO; collectionCode: Herbarium; ownerInstitutionCode: MO; basisOfRecord: PreservedSpecimen
- **Type status:** Other material. **Occurrence:** catalogNumber: K000765296; recordNumber: 6434; recordedBy: Newbould, JB; **Taxon:** scientificName: *Panicum maximum* Jacq.; kingdom: Plantae; family: Poaceae; genus: Panicum; specificEpithet: maximum; scientificNameAuthorship: Jacq.; **Location:** continent: Africa; country: Tanzania; stateProvince: Arusha; county: Ngorongoro; locality: Naibardad Hill; verbatimLocality: South Serengeti; minimumElevationInMeters: 1798; decimalLatitude: -3.116667; decimalLongitude: 35.1; **Event:** eventDate: 1962-12-22; **Record Level:** institutionCode: K; collectionCode: Herbarium; ownerInstitutionCode: K; basisOfRecord: PreservedSpecimen
- **Type status:** Other material. **Occurrence:** catalogNumber: K000765311; recordNumber: 9981; recordedBy: Greenway, PJ; **Taxon:** scientificName: *Panicum maximum* Jacq.; kingdom: Plantae; family: Poaceae; genus: Panicum; specificEpithet: maximum; scientificNameAuthorship: Jacq.; **Location:** continent: Africa; country: Tanzania; stateProvince: Mara; county: Serengeti; locality: Seronera dam; minimumElevationInMeters: 1448; decimalLatitude: -2.433333; decimalLongitude: 34.816667; **Event:** eventDate: 1961-04-05; **Record Level:** institutionCode: K; collectionCode: Herbarium; ownerInstitutionCode: K; basisOfRecord: PreservedSpecimen
- **Type status:** Other material. **Occurrence:** catalogNumber: DB0343; recordNumber: 9981; recordedBy: Greenway, PJ; **Taxon:** scientificName: *Panicum maximum* Jacq.; kingdom: Plantae; family: Poaceae; genus: Panicum; specificEpithet: maximum; scientificNameAuthorship: Jacq.; **Location:** continent: Africa; country: Tanzania; stateProvince: Mara; county: Serengeti; locality: Seronera dam; minimumElevationInMeters: 1448; decimalLatitude: -2.433333; decimalLongitude: 34.816667; **Event:** eventDate: 1961-04-05; **Record Level:** institutionCode: SWRC; collectionCode: Herbarium; ownerInstitutionCode: SWRC; basisOfRecord: PreservedSpecimen
- **Type status:** Other material. **Occurrence:** catalogNumber: K000765310; recordNumber: 6279; recordedBy: Newbould, JB; **Taxon:** scientificName: *Panicum maximum* Jacq.; kingdom: Plantae; family: Poaceae; genus: Panicum; specificEpithet: maximum; scientificNameAuthorship: Jacq.; **Location:** continent: Africa; country: Tanzania; stateProvince: Shinyanga; locality: Ngamuriak; verbatimLocality: East Serengeti.; minimumElevationInMeters: 1981; **Event:** eventDate: 1962-11-21; **Record Level:** institutionCode: K; collectionCode: Herbarium; ownerInstitutionCode: K; basisOfRecord: PreservedSpecimen

##### Distribution

Widespread

#### Panicum monticola

Hook.f.

##### Materials

- **Type status:** Other material. **Occurrence:** catalogNumber: K000984153; recordNumber: 154; recordedBy: Frame, GW; **Taxon:** scientificName: *Panicum monticola* Hook.f.; kingdom: Plantae; family: Poaceae; genus: Panicum; specificEpithet: monticola; scientificNameAuthorship: Hook.f.; **Location:** continent: Africa; country: Tanzania; stateProvince: Arusha; county: Ngorongoro; locality: Empakai Crater; verbatimLocality: Ngorongoro Conservation Area, inside east rim; minimumElevationInMeters: 2300; decimalLatitude: -2.933333; decimalLongitude: 35.816667; **Event:** eventDate: 1973-05-30; **Record Level:** institutionCode: K; collectionCode: Herbarium; ownerInstitutionCode: K; basisOfRecord: PreservedSpecimen
- **Type status:** Other material. **Occurrence:** catalogNumber: DB0261; recordNumber: 154; recordedBy: Frame, GW; **Taxon:** scientificName: *Panicum monticola* Hook.f.; kingdom: Plantae; family: Poaceae; genus: Panicum; specificEpithet: monticola; scientificNameAuthorship: Hook.f.; **Location:** continent: Africa; country: Tanzania; stateProvince: Arusha; county: Ngorongoro; locality: Empakai Crater; verbatimLocality: Ngorongoro Conservation Area, inside east rim; minimumElevationInMeters: 2300; decimalLatitude: -2.933333; decimalLongitude: 35.816667; **Event:** eventDate: 1973-05-30; **Record Level:** institutionCode: SWRC; collectionCode: Herbarium; ownerInstitutionCode: SWRC; basisOfRecord: PreservedSpecimen
- **Type status:** Other material. **Occurrence:** catalogNumber: 1159; recordNumber: 154; recordedBy: Frame, GW; **Taxon:** scientificName: *Panicum monticola* Hook.f.; kingdom: Plantae; family: Poaceae; genus: Panicum; specificEpithet: monticola; scientificNameAuthorship: Hook.f.; **Location:** continent: Africa; country: Tanzania; stateProvince: Arusha; county: Ngorongoro; locality: Empakai Crater; verbatimLocality: Ngorongoro Conservation Area, inside east rim; minimumElevationInMeters: 2300; decimalLatitude: -2.933333; decimalLongitude: 35.816667; **Event:** eventDate: 1973-05-30; **Record Level:** institutionCode: EA; collectionCode: Herbarium; ownerInstitutionCode: EA; basisOfRecord: PreservedSpecimen
- **Type status:** Other material. **Occurrence:** catalogNumber: 1160; recordNumber: 154; recordedBy: Frame, GW; **Taxon:** scientificName: *Panicum monticola* Hook.f.; kingdom: Plantae; family: Poaceae; genus: Panicum; specificEpithet: monticola; scientificNameAuthorship: Hook.f.; **Location:** continent: Africa; country: Tanzania; stateProvince: Arusha; county: Ngorongoro; locality: Empakai Crater; verbatimLocality: Ngorongoro Conservation Area, inside east rim; minimumElevationInMeters: 2300; decimalLatitude: -2.933333; decimalLongitude: 35.816667; **Event:** eventDate: 1973-05-30; **Record Level:** institutionCode: NHT; collectionCode: Herbarium; ownerInstitutionCode: NHT; basisOfRecord: PreservedSpecimen

##### Distribution

Tropical Africa

#### Panicum poioides

Stapf

Panicum graciliculme Napper

##### Materials

- **Type status:** Isotype. **Occurrence:** catalogNumber: K000255564; recordNumber: 9841; recordedBy: Greenway, PJ; **Taxon:** scientificName: *Panicum graciliculme* Napper; kingdom: Plantae; family: Poaceae; genus: Panicum; specificEpithet: graciliculme; scientificNameAuthorship: Napper; **Location:** continent: Africa; country: Tanzania; stateProvince: Mara; county: Serengeti; locality: Seronera; verbatimLocality: Seronera, Serengeti; minimumElevationInMeters: 1530; decimalLatitude: -2.45; decimalLongitude: 34.833333; **Event:** eventDate: 1961-03-17; **Record Level:** institutionCode: K; collectionCode: Herbarium; ownerInstitutionCode: K; basisOfRecord: PreservedSpecimen
- **Type status:** Other material. **Occurrence:** catalogNumber: DB0374; recordNumber: 9841; recordedBy: Greenway, PJ; **Taxon:** scientificName: *Panicum graciliculme* Napper; kingdom: Plantae; family: Poaceae; genus: Panicum; specificEpithet: graciliculme; scientificNameAuthorship: Napper; **Location:** continent: Africa; country: Tanzania; stateProvince: Mara; county: Serengeti; locality: Seronera; verbatimLocality: Seronera, Serengeti; minimumElevationInMeters: 1530; decimalLatitude: -2.45; decimalLongitude: 34.833333; **Event:** eventDate: 1961-03-17; **Record Level:** institutionCode: SWRC; collectionCode: Herbarium; ownerInstitutionCode: SWRC; basisOfRecord: PreservedSpecimen
- **Type status:** Isotype. **Occurrence:** catalogNumber: PRE0591637-0; recordNumber: 9841; recordedBy: Greenway, PJ; **Taxon:** scientificName: *Panicum graciliculme* Napper; kingdom: Plantae; family: Poaceae; genus: Panicum; specificEpithet: graciliculme; scientificNameAuthorship: Napper; **Location:** continent: Africa; country: Tanzania; stateProvince: Mara; county: Serengeti; locality: Seronera; verbatimLocality: Seronera, Serengeti; minimumElevationInMeters: 1530; decimalLatitude: -2.45; decimalLongitude: 34.833333; **Event:** eventDate: 1961-03-17; **Record Level:** institutionCode: PRE; collectionCode: Herbarium; ownerInstitutionCode: PRE; basisOfRecord: PreservedSpecimen
- **Type status:** Isotype. **Occurrence:** catalogNumber: NY00414078; recordNumber: 9841; recordedBy: Greenway, PJ; **Taxon:** scientificName: *Panicum graciliculme* Napper; kingdom: Plantae; family: Poaceae; genus: Panicum; specificEpithet: graciliculme; scientificNameAuthorship: Napper; **Location:** continent: Africa; country: Tanzania; stateProvince: Mara; county: Serengeti; locality: Seronera; verbatimLocality: Seronera, Serengeti; minimumElevationInMeters: 1530; decimalLatitude: -2.45; decimalLongitude: 34.833333; **Event:** eventDate: 1961-03-17; **Record Level:** institutionCode: NY; collectionCode: Herbarium; ownerInstitutionCode: NY; basisOfRecord: PreservedSpecimen
- **Type status:** Isotype. **Occurrence:** catalogNumber: US00731055; recordNumber: 9841; recordedBy: Greenway, PJ; **Taxon:** scientificName: *Panicum graciliculme* Napper; kingdom: Plantae; family: Poaceae; genus: Panicum; specificEpithet: graciliculme; scientificNameAuthorship: Napper; **Location:** continent: Africa; country: Tanzania; stateProvince: Mara; county: Serengeti; locality: Seronera; verbatimLocality: Seronera, Serengeti; minimumElevationInMeters: 1530; decimalLatitude: -2.45; decimalLongitude: 34.833333; **Event:** eventDate: 1961-03-17; **Record Level:** institutionCode: US; collectionCode: Herbarium; ownerInstitutionCode: US; basisOfRecord: PreservedSpecimen
- **Type status:** Isotype. **Occurrence:** catalogNumber: PRE0031839-0; recordNumber: 9841; recordedBy: Greenway, PJ; **Taxon:** scientificName: *Panicum graciliculme* Napper; kingdom: Plantae; family: Poaceae; genus: Panicum; specificEpithet: graciliculme; scientificNameAuthorship: Napper; **Location:** continent: Africa; country: Tanzania; stateProvince: Mara; county: Serengeti; locality: Seronera; verbatimLocality: Seronera, Serengeti; minimumElevationInMeters: 1530; decimalLatitude: -2.45; decimalLongitude: 34.833333; **Event:** eventDate: 1961-03-17; **Record Level:** institutionCode: PRE; collectionCode: Herbarium; ownerInstitutionCode: PRE; basisOfRecord: PreservedSpecimen
- **Type status:** Other material. **Occurrence:** catalogNumber: DB0369; recordNumber: 323; recordedBy: Kreulen, AR; **Taxon:** scientificName: *Panicum poioides* Stapf; kingdom: Plantae; family: Poaceae; genus: Panicum; specificEpithet: poioides; scientificNameAuthorship: Stapf; **Location:** continent: Africa; country: Tanzania; stateProvince: Mara; county: Serengeti; locality: Serengeti; decimalLatitude: -2.333333; decimalLongitude: 34.833333; **Event:** eventDate: 1974-05-10; **Record Level:** institutionCode: SWRC; collectionCode: Herbarium; ownerInstitutionCode: SWRC; basisOfRecord: PreservedSpecimen
- **Type status:** Other material. **Occurrence:** catalogNumber: DB0375; recordNumber: 247; recordedBy: Greenway, PJ; **Taxon:** scientificName: *Panicum poioides* Stapf; kingdom: Plantae; family: Poaceae; genus: Panicum; specificEpithet: poioides; scientificNameAuthorship: Stapf; **Location:** continent: Africa; country: Tanzania; stateProvince: Mara; county: Serengeti; locality: Serengeti; minimumElevationInMeters: 1470; decimalLatitude: -2.333333; decimalLongitude: 34.833333; **Event:** eventDate: 1960-01-24; **Record Level:** institutionCode: SWRC; collectionCode: Herbarium; ownerInstitutionCode: SWRC; basisOfRecord: PreservedSpecimen
- **Type status:** Other material. **Occurrence:** catalogNumber: DB0376; recordNumber: 269; recordedBy: Hubert, MHB; **Taxon:** scientificName: *Panicum poioides* Stapf; kingdom: Plantae; family: Poaceae; genus: Panicum; specificEpithet: poioides; scientificNameAuthorship: Stapf; **Location:** continent: Africa; country: Tanzania; stateProvince: Mara; county: Serengeti; locality: Seronera; verbatimLocality: Near small Kopje, west of Seronera offices and workshops; minimumElevationInMeters: 1470; decimalLatitude: -2.45; decimalLongitude: 34.833333; **Event:** eventDate: 1968-03-21; **Record Level:** institutionCode: SWRC; collectionCode: Herbarium; ownerInstitutionCode: SWRC; basisOfRecord: PreservedSpecimen
- **Type status:** Other material. **Occurrence:** catalogNumber: DB0377; recordNumber: 264; recordedBy: Hubert, MHB; **Taxon:** scientificName: *Panicum poioides* Stapf; kingdom: Plantae; family: Poaceae; genus: Panicum; specificEpithet: poioides; scientificNameAuthorship: Stapf; **Location:** continent: Africa; country: Tanzania; stateProvince: Mara; county: Serengeti; locality: Seronera; minimumElevationInMeters: 1470; decimalLatitude: -2.45; decimalLongitude: 34.833333; **Event:** eventDate: 1968-03-09; **Record Level:** institutionCode: SWRC; collectionCode: Herbarium; ownerInstitutionCode: SWRC; basisOfRecord: PreservedSpecimen

##### Distribution

Tropical Africa

#### Panicum repens

L.

##### Materials

- **Type status:** Other material. **Occurrence:** catalogNumber: K000984143; recordNumber: 2678; recordedBy: Chuwa, S; **Taxon:** scientificName: *Panicum repens* L.; kingdom: Plantae; family: Poaceae; genus: Panicum; specificEpithet: repens; scientificNameAuthorship: L.; **Location:** continent: Africa; country: Tanzania; stateProvince: Arusha; county: Ngorongoro; locality: Oldeani Mt; verbatimLocality: Ngorongoro Conservation Area, Oldean Mountain; minimumElevationInMeters: 2378; decimalLatitude: -3.266667; decimalLongitude: 35.433333; **Event:** eventDate: 1988-10-21; **Record Level:** institutionCode: K; collectionCode: Herbarium; ownerInstitutionCode: K; basisOfRecord: PreservedSpecimen
- **Type status:** Other material. **Occurrence:** catalogNumber: DB0382; recordNumber: 1094; recordedBy: Suleiman, HO; **Taxon:** scientificName: *Panicum repens* L.; kingdom: Plantae; family: Poaceae; genus: Panicum; specificEpithet: repens; scientificNameAuthorship: L.; **Location:** continent: Africa; country: Tanzania; stateProvince: Mara; county: Serengeti; locality: Serengeti National Park; decimalLatitude: -2.333333; decimalLongitude: 34.833333; **Event:** eventDate: 2001-02-15; **Record Level:** institutionCode: SWRC; collectionCode: Herbarium; ownerInstitutionCode: SWRC; basisOfRecord: PreservedSpecimen

##### Distribution

Widespread

#### Paspalidium geminatum

(Forssk.) Stapf

Setaria geminata (Forssk.) Veldkamp

##### Materials

- **Type status:** Other material. **Occurrence:** catalogNumber: DB0456; recordNumber: 10348; recordedBy: Greenway, PJ; **Taxon:** scientificName: *Paspalidium geminatum* (Forssk.) Stapf; kingdom: Plantae; family: Poaceae; genus: Paspalidium; specificEpithet: geminatum; scientificNameAuthorship: (Forssk.) Stapf; **Location:** continent: Africa; country: Tanzania; stateProvince: Mara; county: Serengeti; locality: Banagi; verbatimLocality: Banagi Mgungu river; minimumElevationInMeters: 1320; decimalLatitude: -2.3; decimalLongitude: 34.833333; **Event:** eventDate: 1961-05-31; **Record Level:** institutionCode: SWRC; collectionCode: Herbarium; ownerInstitutionCode: SWRC; basisOfRecord: PreservedSpecimen
- **Type status:** Other material. **Occurrence:** catalogNumber: K001087172; recordNumber: 10348; recordedBy: Greenway, PJ; **Taxon:** scientificName: *Paspalidium geminatum* (Forssk.) Stapf; kingdom: Plantae; family: Poaceae; genus: Paspalidium; specificEpithet: geminatum; scientificNameAuthorship: (Forssk.) Stapf; **Location:** continent: Africa; country: Tanzania; stateProvince: Mara; county: Serengeti; locality: Banagi; verbatimLocality: Banagi Mgungu river; minimumElevationInMeters: 1320; decimalLatitude: -2.3; decimalLongitude: 34.833333; **Event:** eventDate: 1961-05-31; **Record Level:** institutionCode: K; collectionCode: Herbarium; ownerInstitutionCode: K; basisOfRecord: PreservedSpecimen

##### Distribution

Widespread

#### Paspalum scrobiculatum

L.

Paspalum commersonii Lam.

##### Materials

- **Type status:** Other material. **Occurrence:** catalogNumber: K000984121; recordNumber: 10744; recordedBy: Greenway, PJ; **Taxon:** scientificName: *Paspalum commersonii* Lam.; kingdom: Plantae; family: Poaceae; genus: Paspalum; specificEpithet: commersonii; scientificNameAuthorship: Lam.; **Location:** continent: Africa; country: Tanzania; stateProvince: Mara; county: Serengeti; locality: Bolgonja river; minimumElevationInMeters: 1463; decimalLatitude: -1.783333; decimalLongitude: 35.2; **Event:** eventDate: 1962-08-17; **Record Level:** institutionCode: K; collectionCode: Herbarium; ownerInstitutionCode: K; basisOfRecord: PreservedSpecimen

##### Distribution

Widespread

#### Pennisetum sp.

##### Materials

- **Type status:** Other material. **Occurrence:** catalogNumber: 678; recordNumber: 481; recordedBy: Ellemann, L; **Taxon:** scientificName: Pennisetum; kingdom: Plantae; family: Poaceae; genus: Pennisetum; **Location:** continent: Africa; country: Tanzania; stateProvince: Arusha; county: Ngorongoro; locality: Kelogi; verbatimLocality: Ngorongoro Conservation Area, Kelogi.; minimumElevationInMeters: 1550; decimalLatitude: -3.033; decimalLongitude: 35.283; **Event:** eventDate: 1993-05-03; **Record Level:** institutionCode: AAU; collectionCode: Herbarium; ownerInstitutionCode: AAU; basisOfRecord: PreservedSpecimen

#### Pennisetum clandestinum

Hochst. ex Chiov.

Cenchrus clandestinus (Hochst. ex Chiov.) Morrone

##### Materials

- **Type status:** Other material. **Occurrence:** catalogNumber: 659; recordNumber: 715; recordedBy: Ellemann, L; **Taxon:** scientificName: *Pennisetum clandestinum* Hochst. ex Chiov.; kingdom: Plantae; family: Poaceae; genus: Pennisetum; specificEpithet: clandestinum; scientificNameAuthorship: Hochst. ex Chiov.; **Location:** continent: Africa; country: Tanzania; stateProvince: Arusha; county: Ngorongoro; locality: Sendui; verbatimLocality: Ngorongoro Conservation Area, Sendui, close to ole Senguyans boma.; minimumElevationInMeters: 2650; decimalLatitude: -2.916; decimalLongitude: 35.716; **Event:** eventDate: 1993-07-22; **Record Level:** institutionCode: AAU; collectionCode: Herbarium; ownerInstitutionCode: AAU; basisOfRecord: PreservedSpecimen
- **Type status:** Other material. **Occurrence:** catalogNumber: 660; recordNumber: 774; recordedBy: Ellemann, L; **Taxon:** scientificName: *Pennisetum clandestinum* Hochst. ex Chiov.; kingdom: Plantae; family: Poaceae; genus: Pennisetum; specificEpithet: clandestinum; scientificNameAuthorship: Hochst. ex Chiov.; **Location:** continent: Africa; country: Tanzania; stateProvince: Arusha; county: Ngorongoro; locality: Irmisigiyo; verbatimLocality: Ngorongoro Conservation Area, Irmisigiyo; minimumElevationInMeters: 2400; decimalLatitude: -3.2; decimalLongitude: 35.366; **Event:** eventDate: 1993-08-05; **Record Level:** institutionCode: AAU; collectionCode: Herbarium; ownerInstitutionCode: AAU; basisOfRecord: PreservedSpecimen
- **Type status:** Other material. **Occurrence:** catalogNumber: 661; recordNumber: 833; recordedBy: Ellemann, L; **Taxon:** scientificName: *Pennisetum clandestinum* Hochst. ex Chiov.; kingdom: Plantae; family: Poaceae; genus: Pennisetum; specificEpithet: clandestinum; scientificNameAuthorship: Hochst. ex Chiov.; **Location:** continent: Africa; country: Tanzania; stateProvince: Arusha; county: Ngorongoro; locality: Esongoyo; verbatimLocality: Ngorongoro Conservation Area, Esongoyo, forest 23 km from Alaililai.; decimalLatitude: -3.166; decimalLongitude: 35.466; **Event:** eventDate: 1993-08-22; **Record Level:** institutionCode: AAU; collectionCode: Herbarium; ownerInstitutionCode: AAU; basisOfRecord: PreservedSpecimen
- **Type status:** Other material. **Occurrence:** catalogNumber: 662; recordNumber: 863; recordedBy: Ellemann, L; **Taxon:** scientificName: *Pennisetum clandestinum* Hochst. ex Chiov.; kingdom: Plantae; family: Poaceae; genus: Pennisetum; specificEpithet: clandestinum; scientificNameAuthorship: Hochst. ex Chiov.; **Location:** continent: Africa; country: Tanzania; stateProvince: Arusha; county: Ngorongoro; locality: Rhino lodge; verbatimLocality: Ngorongoro Conservation Area, Entim e Rotian, close to Rhino-lodge.; minimumElevationInMeters: 2200; decimalLatitude: -3.25; decimalLongitude: 35.516; **Event:** eventDate: 1993-08-29; **Record Level:** institutionCode: AAU; collectionCode: Herbarium; ownerInstitutionCode: AAU; basisOfRecord: PreservedSpecimen
- **Type status:** Other material. **Occurrence:** catalogNumber: 663; recordNumber: 944; recordedBy: Ellemann, L; **Taxon:** scientificName: *Pennisetum clandestinum* Hochst. ex Chiov.; kingdom: Plantae; family: Poaceae; genus: Pennisetum; specificEpithet: clandestinum; scientificNameAuthorship: Hochst. ex Chiov.; **Location:** continent: Africa; country: Tanzania; stateProvince: Arusha; county: Ngorongoro; locality: Irmisigiyo; verbatimLocality: Ngorongoro Conservation Area, Irmisigiyo; minimumElevationInMeters: 2350; decimalLatitude: -3.2; decimalLongitude: 35.366; **Event:** eventDate: 1993-09-30; **Record Level:** institutionCode: AAU; collectionCode: Herbarium; ownerInstitutionCode: AAU; basisOfRecord: PreservedSpecimen
- **Type status:** Other material. **Occurrence:** catalogNumber: 664; recordNumber: 910b; recordedBy: Ellemann, L; **Taxon:** scientificName: *Pennisetum clandestinum* Hochst. ex Chiov.; kingdom: Plantae; family: Poaceae; genus: Pennisetum; specificEpithet: clandestinum; scientificNameAuthorship: Hochst. ex Chiov.; **Location:** continent: Africa; country: Tanzania; stateProvince: Arusha; county: Ngorongoro; locality: Armakutian; verbatimLocality: Ngorongoro Conservation Area, Armakutian, above Alchorai and the upper water tank in Loomyoni.; minimumElevationInMeters: 2000; decimalLatitude: -3.2; decimalLongitude: 35.483; **Event:** eventDate: 1993-09-15; **Record Level:** institutionCode: AAU; collectionCode: Herbarium; ownerInstitutionCode: AAU; basisOfRecord: PreservedSpecimen
- **Type status:** Other material. **Occurrence:** catalogNumber: K000766339; recordNumber: 103; recordedBy: Frame, GW; **Taxon:** scientificName: *Pennisetum clandestinum* Hochst. ex Chiov.; kingdom: Plantae; family: Poaceae; genus: Pennisetum; specificEpithet: clandestinum; scientificNameAuthorship: Hochst. ex Chiov.; **Location:** continent: Africa; country: Tanzania; stateProvince: Arusha; county: Ngorongoro; locality: Empakai Crater; verbatimLocality: Empakaai crater, top of east rim.; minimumElevationInMeters: 2600; decimalLatitude: -2.933333; decimalLongitude: 35.816667; **Event:** eventDate: 1973-03-17; **Record Level:** institutionCode: K; collectionCode: Herbarium; ownerInstitutionCode: K; basisOfRecord: PreservedSpecimen
- **Type status:** Other material. **Occurrence:** catalogNumber: DB0422; recordNumber: 103; recordedBy: Frame, GW; **Taxon:** scientificName: *Pennisetum clandestinum* Hochst. ex Chiov.; kingdom: Plantae; family: Poaceae; genus: Pennisetum; specificEpithet: clandestinum; scientificNameAuthorship: Hochst. ex Chiov.; **Location:** continent: Africa; country: Tanzania; stateProvince: Arusha; county: Ngorongoro; locality: Empakai Crater; verbatimLocality: Empakaai crater, top of east rim.; minimumElevationInMeters: 2600; decimalLatitude: -2.933333; decimalLongitude: 35.816667; **Event:** eventDate: 1973-03-17; **Record Level:** institutionCode: SWRC; collectionCode: Herbarium; ownerInstitutionCode: SWRC; basisOfRecord: PreservedSpecimen

##### Distribution

Native to Eastern Africa, introduced widely

#### Pennisetum mezianum

Leeke

##### Materials

- **Type status:** Other material. **Occurrence:** catalogNumber: DB0432; recordNumber: 17; recordedBy: Schmidt, W; **Taxon:** scientificName: *Pennisetum mezianum* Leeke; kingdom: Plantae; family: Poaceae; genus: Pennisetum; specificEpithet: mezianum; scientificNameAuthorship: Leeke; **Location:** continent: Africa; country: Tanzania; stateProvince: Mara; county: Serengeti; locality: Simba Kopjes; verbatimLocality: Simba Kopja, Serengeti Plain; decimalLatitude: -2.7; decimalLongitude: 34.916667; **Event:** eventDate: 1972-03-11; **Record Level:** institutionCode: SWRC; collectionCode: Herbarium; ownerInstitutionCode: SWRC; basisOfRecord: PreservedSpecimen
- **Type status:** Other material. **Occurrence:** catalogNumber: DB0429; recordNumber: 172; recordedBy: Belsky, PJ; **Taxon:** scientificName: *Pennisetum mezianum* Leeke; kingdom: Plantae; family: Poaceae; genus: Pennisetum; specificEpithet: mezianum; scientificNameAuthorship: Leeke; **Location:** continent: Africa; country: Tanzania; stateProvince: Mara; county: Serengeti; locality: Serengeti Research Institute; verbatimLocality: Serengeti National Park. Serengeti Research Institute compound; decimalLatitude: -2.433333; decimalLongitude: 34.85; **Event:** eventDate: 1981-05-11; **Record Level:** institutionCode: SWRC; collectionCode: Herbarium; ownerInstitutionCode: SWRC; basisOfRecord: PreservedSpecimen
- **Type status:** Other material. **Occurrence:** catalogNumber: DB0428; recordNumber: 229; recordedBy: Kreulen, AR; **Taxon:** scientificName: *Pennisetum mezianum* Leeke; kingdom: Plantae; family: Poaceae; genus: Pennisetum; specificEpithet: mezianum; scientificNameAuthorship: Leeke; **Location:** continent: Africa; country: Tanzania; stateProvince: Shinyanga; locality: Naabi Hill; verbatimLocality: Near Naabi Hill; decimalLatitude: -2.883333; decimalLongitude: 35.033333; **Event:** eventDate: 1974-01-07; **Record Level:** institutionCode: SWRC; collectionCode: Herbarium; ownerInstitutionCode: SWRC; basisOfRecord: PreservedSpecimen
- **Type status:** Other material. **Occurrence:** catalogNumber: 665; recordNumber: 985; recordedBy: Ellemann, L; **Taxon:** scientificName: *Pennisetum mezianum* Leeke; kingdom: Plantae; family: Poaceae; genus: Pennisetum; specificEpithet: mezianum; scientificNameAuthorship: Leeke; **Location:** continent: Africa; country: Tanzania; stateProvince: Arusha; county: Ngorongoro; locality: Olbalbal; verbatimLocality: Ngorongoro Conservation Area, Olbalbal west of the swamp.; minimumElevationInMeters: 1350; decimalLatitude: -3.05; decimalLongitude: 35.466; **Event:** eventDate: 1993-12-10; **Record Level:** institutionCode: AAU; collectionCode: Herbarium; ownerInstitutionCode: AAU; basisOfRecord: PreservedSpecimen
- **Type status:** Other material. **Occurrence:** catalogNumber: 666; recordNumber: 1017; recordedBy: Ellemann, L; **Taxon:** scientificName: *Pennisetum mezianum* Leeke; kingdom: Plantae; family: Poaceae; genus: Pennisetum; specificEpithet: mezianum; scientificNameAuthorship: Leeke; **Location:** continent: Africa; country: Tanzania; stateProvince: Arusha; county: Ngorongoro; locality: Olbili-Esere; verbatimLocality: Ngorongoro Conservation Area, Olbili-Esere.; minimumElevationInMeters: 1600; decimalLatitude: -3.283; decimalLongitude: 35.166; **Event:** eventDate: 1994-01-23; **Record Level:** institutionCode: AAU; collectionCode: Herbarium; ownerInstitutionCode: AAU; basisOfRecord: PreservedSpecimen
- **Type status:** Other material. **Occurrence:** catalogNumber: 667; recordNumber: s.n.; recordedBy: Mboya, E; **Taxon:** scientificName: *Pennisetum mezianum* Leeke; kingdom: Plantae; family: Poaceae; genus: Pennisetum; specificEpithet: mezianum; scientificNameAuthorship: Leeke; **Location:** continent: Africa; country: Tanzania; stateProvince: Mara; county: Serengeti; locality: Serengeti National Park; verbatimLocality: T1. Serengeti National Park. Ndabaka-Seronera Road.; minimumElevationInMeters: 1297; decimalLatitude: -2.28; decimalLongitude: 34.5; **Event:** eventDate: 2004-02-04; **Record Level:** institutionCode: MO; collectionCode: Herbarium; ownerInstitutionCode: MO; basisOfRecord: PreservedSpecimen
- **Type status:** Other material. **Occurrence:** catalogNumber: K000766238; recordNumber: 318; recordedBy: Paulo, S; **Taxon:** scientificName: *Pennisetum mezianum* Leeke; kingdom: Plantae; family: Poaceae; genus: Pennisetum; specificEpithet: mezianum; scientificNameAuthorship: Leeke; **Location:** continent: Africa; country: Tanzania; stateProvince: Arusha; county: Ngorongoro; locality: Ngorongoro Crater; minimumElevationInMeters: 1676; decimalLatitude: -3.166667; decimalLongitude: 35.583333; **Event:** eventDate: 1958-04-19; **Record Level:** institutionCode: K; collectionCode: Herbarium; ownerInstitutionCode: K; basisOfRecord: PreservedSpecimen
- **Type status:** Other material. **Occurrence:** catalogNumber: K000766243; recordNumber: 361; recordedBy: Paulo, S; **Taxon:** scientificName: *Pennisetum mezianum* Leeke; kingdom: Plantae; family: Poaceae; genus: Pennisetum; specificEpithet: mezianum; scientificNameAuthorship: Leeke; **Location:** continent: Africa; country: Tanzania; stateProvince: Mara; county: Serengeti; locality: Engare Nanyuki; verbatimLocality: North Serengeti, North of Engare Nanyuki.; decimalLatitude: -2.616667; decimalLongitude: 35.216667; **Event:** eventDate: 1958-04-23; **Record Level:** institutionCode: K; collectionCode: Herbarium; ownerInstitutionCode: K; basisOfRecord: PreservedSpecimen
- **Type status:** Other material. **Occurrence:** catalogNumber: K000766244; recordNumber: 1505; recordedBy: Heady, AF; **Taxon:** scientificName: *Pennisetum mezianum* Leeke; kingdom: Plantae; family: Poaceae; genus: Pennisetum; specificEpithet: mezianum; scientificNameAuthorship: Leeke; **Location:** continent: Africa; country: Tanzania; stateProvince: Arusha; county: Ngorongoro; locality: Ngorongoro Crater; decimalLatitude: -3.166667; decimalLongitude: 35.583333; **Event:** eventDate: 1959-02-15; **Record Level:** institutionCode: K; collectionCode: Herbarium; ownerInstitutionCode: K; basisOfRecord: PreservedSpecimen
- **Type status:** Other material. **Occurrence:** catalogNumber: K000766254; recordNumber: 9836; recordedBy: Greenway, PJ; **Taxon:** scientificName: *Pennisetum mezianum* Leeke; kingdom: Plantae; family: Poaceae; genus: Pennisetum; specificEpithet: mezianum; scientificNameAuthorship: Leeke; **Location:** continent: Africa; country: Tanzania; stateProvince: Mara; county: Serengeti; locality: Seronera; minimumElevationInMeters: 1554; decimalLatitude: -2.45; decimalLongitude: 34.833333; **Event:** eventDate: 1961-03-17; **Record Level:** institutionCode: K; collectionCode: Herbarium; ownerInstitutionCode: K; basisOfRecord: PreservedSpecimen
- **Type status:** Other material. **Occurrence:** catalogNumber: DB0433; recordNumber: 9836; recordedBy: Greenway, PJ; **Taxon:** scientificName: *Pennisetum mezianum* Leeke; kingdom: Plantae; family: Poaceae; genus: Pennisetum; specificEpithet: mezianum; scientificNameAuthorship: Leeke; **Location:** continent: Africa; country: Tanzania; stateProvince: Mara; county: Serengeti; locality: Seronera; minimumElevationInMeters: 1554; decimalLatitude: -2.45; decimalLongitude: 34.833333; **Event:** eventDate: 1961-03-17; **Record Level:** institutionCode: SWRC; collectionCode: Herbarium; ownerInstitutionCode: SWRC; basisOfRecord: PreservedSpecimen
- **Type status:** Other material. **Occurrence:** catalogNumber: K000766255; recordNumber: 19340; recordedBy: Raynal, J; **Taxon:** scientificName: *Pennisetum mezianum* Leeke; kingdom: Plantae; family: Poaceae; genus: Pennisetum; specificEpithet: mezianum; scientificNameAuthorship: Leeke; **Location:** continent: Africa; country: Tanzania; stateProvince: Mara; county: Serengeti; locality: Lake Lagarja; verbatimLocality: Ngorongoro conservation areas, Serengeti National Park, Southern edge of Lake Lagarja (=Ndutu); minimumElevationInMeters: 1700; maximumElevationInMeters: 1730; decimalLatitude: -3; decimalLongitude: 35.033333; **Event:** eventDate: 1977-10-01; **Record Level:** institutionCode: K; collectionCode: Herbarium; ownerInstitutionCode: K; basisOfRecord: PreservedSpecimen
- **Type status:** Other material. **Occurrence:** catalogNumber: K000766240; recordNumber: 70; recordedBy: Brooks, GP; **Taxon:** scientificName: *Pennisetum mezianum* Leeke; kingdom: Plantae; family: Poaceae; genus: Pennisetum; specificEpithet: mezianum; scientificNameAuthorship: Leeke; **Location:** continent: Africa; country: Tanzania; stateProvince: Mara; county: Serengeti; locality: Banagi Hill; verbatimLocality: Serengeti Plain; minimumElevationInMeters: 1432; decimalLatitude: -2.3; decimalLongitude: 34.833333; **Event:** eventDate: 1954-02-08; **Record Level:** institutionCode: K; collectionCode: Herbarium; ownerInstitutionCode: K; basisOfRecord: PreservedSpecimen
- **Type status:** Other material. **Occurrence:** catalogNumber: K000766246; recordNumber: 294; recordedBy: Paulo, S; **Taxon:** scientificName: *Pennisetum mezianum* Leeke; kingdom: Plantae; family: Poaceae; genus: Pennisetum; specificEpithet: mezianum; scientificNameAuthorship: Leeke; **Location:** continent: Africa; country: Tanzania; stateProvince: Mara; county: Serengeti; locality: Seronera airdrome; verbatimLocality: Musoma district.; minimumElevationInMeters: 1524; decimalLatitude: -2.333333; decimalLongitude: 34.833333; **Event:** eventDate: 1958-04-18; **Record Level:** institutionCode: K; collectionCode: Herbarium; ownerInstitutionCode: K; basisOfRecord: PreservedSpecimen

##### Distribution

Tropical Africa

#### Pennisetum polystachion

(L.) Schult.

##### Materials

- **Type status:** Other material. **Occurrence:** catalogNumber: 669; recordNumber: 658; recordedBy: Ellemann, L; **Taxon:** scientificName: *Pennisetum polystachion* (L.) Schult.; kingdom: Plantae; family: Poaceae; genus: Pennisetum; specificEpithet: polystachion; scientificNameAuthorship: (L.) Schult.; **Location:** continent: Africa; country: Tanzania; stateProvince: Arusha; county: Ngorongoro; locality: Ololgumi; verbatimLocality: Ngorongoro Conservation Area, Ololgumi, along Kakesio river, a seasonal river.; minimumElevationInMeters: 1600; decimalLatitude: -3.333; decimalLongitude: 35.033; **Event:** eventDate: 1993-06-15; **Record Level:** institutionCode: AAU; collectionCode: Herbarium; ownerInstitutionCode: AAU; basisOfRecord: PreservedSpecimen

##### Distribution

Widespread

#### Pennisetum polystachion atrichum

(Stapf & C.E.Hubb) Brunken

##### Materials

- **Type status:** Other material. **Occurrence:** catalogNumber: 668; recordNumber: 355; recordedBy: Ellemann, L; **Taxon:** scientificName: Pennisetum polystachion (L.) Schult. subsp. atrichum (Stapf & C.E.Hubb) Brunken; kingdom: Plantae; family: Poaceae; genus: Pennisetum; specificEpithet: polystachion; infraspecificEpithet: atrichum; scientificNameAuthorship: (Stapf & C.E.Hubb) Brunken; **Location:** continent: Africa; country: Tanzania; stateProvince: Arusha; county: Ngorongoro; locality: Endulen; verbatimLocality: Ngorongoro Conservation Area. West of Endulen airstrip.; minimumElevationInMeters: 1800; decimalLatitude: -3.2; decimalLongitude: 35.2; **Event:** eventDate: 1993-04-14; **Record Level:** institutionCode: AAU; collectionCode: Herbarium; ownerInstitutionCode: AAU; basisOfRecord: PreservedSpecimen

##### Distribution

Tropical Africa

#### Pennisetum polystachion polystachion

##### Materials

- **Type status:** Other material. **Occurrence:** catalogNumber: 670; recordNumber: 721; recordedBy: Ellemann, L; **Taxon:** scientificName: Pennisetum polystachion (L.) Schult. subsp. polystachion; kingdom: Plantae; family: Poaceae; genus: Pennisetum; specificEpithet: polystachion; infraspecificEpithet: polystachion; scientificNameAuthorship: (L.) Schult.; **Location:** continent: Africa; country: Tanzania; stateProvince: Arusha; county: Ngorongoro; locality: Sendui; verbatimLocality: Ngorongoro Conservation Area Sendui.; minimumElevationInMeters: 2450; decimalLatitude: -2.916; decimalLongitude: 35.7; **Event:** eventDate: 1993-07-22; **Record Level:** institutionCode: AAU; collectionCode: Herbarium; ownerInstitutionCode: AAU; basisOfRecord: PreservedSpecimen
- **Type status:** Other material. **Occurrence:** catalogNumber: 671; recordNumber: 934; recordedBy: Ellemann, L; **Taxon:** scientificName: Pennisetum polystachion (L.) Schult. subsp. polystachion; kingdom: Plantae; family: Poaceae; genus: Pennisetum; specificEpithet: polystachion; infraspecificEpithet: polystachion; scientificNameAuthorship: (L.) Schult.; **Location:** continent: Africa; country: Tanzania; stateProvince: Arusha; county: Ngorongoro; locality: Oltebesi; verbatimLocality: Ngorongoro Conservation Area, Oltebesi above Ndiyan village; minimumElevationInMeters: 2100; decimalLatitude: -3.166; decimalLongitude: 35.283; **Event:** eventDate: 1993-09-29; **Record Level:** institutionCode: AAU; collectionCode: Herbarium; ownerInstitutionCode: AAU; basisOfRecord: PreservedSpecimen
- **Type status:** Other material. **Occurrence:** catalogNumber: 672; recordNumber: 945; recordedBy: Ellemann, L; **Taxon:** scientificName: Pennisetum polystachion (L.) Schult. subsp. polystachion; kingdom: Plantae; family: Poaceae; genus: Pennisetum; specificEpithet: polystachion; infraspecificEpithet: polystachion; scientificNameAuthorship: (L.) Schult.; **Location:** continent: Africa; country: Tanzania; stateProvince: Arusha; county: Ngorongoro; locality: Irmisigiyo; verbatimLocality: Ngorongoro Conservation Area, Irmisigiyo; minimumElevationInMeters: 2350; decimalLatitude: -3.2; decimalLongitude: 35.366; **Event:** eventDate: 1993-09-30; **Record Level:** institutionCode: AAU; collectionCode: Herbarium; ownerInstitutionCode: AAU; basisOfRecord: PreservedSpecimen
- **Type status:** Other material. **Occurrence:** catalogNumber: 673; recordNumber: 7763; recordedBy: Ellemann, L; **Taxon:** scientificName: Pennisetum polystachion (L.) Schult. subsp. polystachion; kingdom: Plantae; family: Poaceae; genus: Pennisetum; specificEpithet: polystachion; infraspecificEpithet: polystachion; scientificNameAuthorship: (L.) Schult.; **Location:** continent: Africa; country: Tanzania; stateProvince: Arusha; county: Ngorongoro; locality: Oldonyo; verbatimLocality: Ngorongoro Conservation Area, Oloronyo,; minimumElevationInMeters: 2700; decimalLatitude: -2.75; decimalLongitude: 35.45; **Event:** eventDate: 1993-08-05; **Record Level:** institutionCode: AAU; collectionCode: Herbarium; ownerInstitutionCode: AAU; basisOfRecord: PreservedSpecimen

##### Distribution

Widespread

#### Pennisetum riparium

Hochst. ex A.Rich.

Cenchrus dowsonii (Stapf & C.E.Hubb) Morrone | Pennisetum dowsonii Stapf

##### Materials

- **Type status:** Other material. **Occurrence:** catalogNumber: K000984072; recordNumber: 1663; recordedBy: Heady, HF; **Taxon:** scientificName: *Pennisetum dowsonii* Stapf; kingdom: Plantae; family: Poaceae; genus: Pennisetum; specificEpithet: dowsonii; scientificNameAuthorship: Stapf; **Location:** continent: Africa; country: Tanzania; stateProvince: Arusha; county: Ngorongoro; locality: Ngorongoro Crater; decimalLatitude: -3.166667; decimalLongitude: 35.583333; **Event:** eventDate: 1959-02-17; **Record Level:** institutionCode: K; collectionCode: Herbarium; ownerInstitutionCode: K; basisOfRecord: PreservedSpecimen
- **Type status:** Other material. **Occurrence:** catalogNumber: K000984073; recordNumber: 31; recordedBy: Senga, C; **Taxon:** scientificName: *Pennisetum dowsonii* Stapf; kingdom: Plantae; family: Poaceae; genus: Pennisetum; specificEpithet: dowsonii; scientificNameAuthorship: Stapf; **Location:** continent: Africa; country: Tanzania; stateProvince: Arusha; county: Ngorongoro; locality: Ngorongoro; minimumElevationInMeters: 2195; decimalLatitude: -3; decimalLongitude: 35.5; **Event:** eventDate: 1961-02-08; **Record Level:** institutionCode: K; collectionCode: Herbarium; ownerInstitutionCode: K; basisOfRecord: PreservedSpecimen
- **Type status:** Other material. **Occurrence:** catalogNumber: K000984074; recordNumber: 89016A; recordedBy: Pocs, T; Chuwa, S; **Taxon:** scientificName: *Pennisetum dowsonii* Stapf; kingdom: Plantae; family: Poaceae; genus: Pennisetum; specificEpithet: dowsonii; scientificNameAuthorship: Stapf; **Location:** continent: Africa; country: Tanzania; stateProvince: Arusha; county: Ngorongoro; locality: Ngorongoro; verbatimLocality: Ngorongoro Conservation Area, Rotian glade; minimumElevationInMeters: 2300; decimalLatitude: -3; decimalLongitude: 35.5; **Event:** eventDate: 1989-01-13; **Record Level:** institutionCode: K; collectionCode: Herbarium; ownerInstitutionCode: K; basisOfRecord: PreservedSpecimen
- **Type status:** Other material. **Occurrence:** catalogNumber: 939; recordNumber: 1663; recordedBy: Heady, AF; **Taxon:** scientificName: *Pennisetum dowsonii* Stapf; kingdom: Plantae; family: Poaceae; genus: Pennisetum; specificEpithet: dowsonii; scientificNameAuthorship: Stapf; **Location:** continent: Africa; country: Tanzania; stateProvince: Arusha; county: Ngorongoro; locality: Ngorongoro Crater; decimalLatitude: -3.166667; decimalLongitude: 35.583333; **Event:** eventDate: 1959-02-17; **Record Level:** collectionCode: Herbarium; ownerInstitutionCode: ?; basisOfRecord: PreservedSpecimen
- **Type status:** Other material. **Occurrence:** catalogNumber: K001087173; recordNumber: 12547; recordedBy: Greenway, PJ; Kanuri; **Taxon:** scientificName: *Pennisetum dowsonii* Stapf; kingdom: Plantae; family: Poaceae; genus: Pennisetum; specificEpithet: dowsonii; scientificNameAuthorship: Stapf; **Location:** continent: Africa; country: Tanzania; stateProvince: Arusha; county: Ngorongoro; locality: Munge Swamp; verbatimLocality: NW end of Munge Swamp, Ngorongoro crater.; minimumElevationInMeters: 1676; decimalLatitude: -3; decimalLongitude: 35.65; **Event:** eventDate: 1966-07-08; **Record Level:** institutionCode: K; collectionCode: Herbarium; ownerInstitutionCode: K; basisOfRecord: PreservedSpecimen

##### Distribution

Tanzania, Kenya, Uganda, Ethiopia & Burundi

#### Pennisetum setaceum

(Forssk.) Chiov.

Cenchrus setaceus (Forssk.) Morrone

##### Materials

- **Type status:** Other material. **Occurrence:** catalogNumber: K000984081; recordNumber: 10356; recordedBy: Greenway, PJ; **Taxon:** scientificName: *Pennisetum setaceum* (Forssk.) Chiov.; kingdom: Plantae; family: Poaceae; genus: Pennisetum; specificEpithet: setaceum; scientificNameAuthorship: (Forssk.) Chiov.; **Location:** continent: Africa; country: Tanzania; stateProvince: Mara; county: Serengeti; locality: Seronera; verbatimLocality: Seronera to Naabi hill, mile 20; minimumElevationInMeters: 1585; decimalLatitude: -2.65; decimalLongitude: 35.333333; **Event:** eventDate: 1961-06-03; **Record Level:** institutionCode: K; collectionCode: Herbarium; ownerInstitutionCode: K; basisOfRecord: PreservedSpecimen
- **Type status:** Other material. **Occurrence:** catalogNumber: K000984082; recordNumber: 13152; recordedBy: Greenway, PJ; Kanuri; Limbrey, H; Braun, H; **Taxon:** scientificName: *Pennisetum setaceum* (Forssk.) Chiov.; kingdom: Plantae; family: Poaceae; genus: Pennisetum; specificEpithet: setaceum; scientificNameAuthorship: (Forssk.) Chiov.; **Location:** continent: Africa; country: Tanzania; stateProvince: Mara; county: Serengeti; locality: Simba Kopjes; verbatimLocality: Serengeti plain; minimumElevationInMeters: 1524; decimalLatitude: -2.7; decimalLongitude: 34.916667; **Event:** eventDate: 1968-02-08; **Record Level:** institutionCode: K; collectionCode: Herbarium; ownerInstitutionCode: K; basisOfRecord: PreservedSpecimen
- **Type status:** Other material. **Occurrence:** catalogNumber: K000984083; recordNumber: 4; recordedBy: Newbould, JB; Thesiger, WP; **Taxon:** scientificName: *Pennisetum setaceum* (Forssk.) Chiov.; kingdom: Plantae; family: Poaceae; genus: Pennisetum; specificEpithet: setaceum; scientificNameAuthorship: (Forssk.) Chiov.; **Location:** continent: Africa; country: Tanzania; stateProvince: Arusha; county: Ngorongoro; locality: Lolgarien; verbatimLocality: Oldoinyo-Gol; minimumElevationInMeters: 1524; decimalLatitude: -2.6; decimalLongitude: 35.383333; **Event:** eventDate: 1961-5; **Record Level:** institutionCode: K; collectionCode: Herbarium; ownerInstitutionCode: K; basisOfRecord: PreservedSpecimen
- **Type status:** Other material. **Occurrence:** catalogNumber: K000984084; recordNumber: 2599; recordedBy: Chuwa, S; **Taxon:** scientificName: *Pennisetum setaceum* (Forssk.) Chiov.; kingdom: Plantae; family: Poaceae; genus: Pennisetum; specificEpithet: setaceum; scientificNameAuthorship: (Forssk.) Chiov.; **Location:** continent: Africa; country: Tanzania; stateProvince: Arusha; county: Ngorongoro; locality: Ngorongoro; verbatimLocality: Nasera rock; minimumElevationInMeters: 1700; decimalLatitude: -3; decimalLongitude: 35.5; **Event:** eventDate: 1987-04-30; **Record Level:** institutionCode: K; collectionCode: Herbarium; ownerInstitutionCode: K; basisOfRecord: PreservedSpecimen
- **Type status:** Other material. **Occurrence:** catalogNumber: DB0442; recordNumber: 10356; recordedBy: Greenway, PJ; **Taxon:** scientificName: *Pennisetum setaceum* (Forssk.) Chiov.; kingdom: Plantae; family: Poaceae; genus: Pennisetum; specificEpithet: setaceum; scientificNameAuthorship: (Forssk.) Chiov.; **Location:** continent: Africa; country: Tanzania; stateProvince: Mara; county: Serengeti; locality: Seronera; verbatimLocality: Seronera to Naabi hill, mile 20; minimumElevationInMeters: 1585; decimalLatitude: -2.65; decimalLongitude: 35.333333; **Event:** eventDate: 1961-06-03; **Record Level:** institutionCode: SWRC; collectionCode: Herbarium; ownerInstitutionCode: SWRC; basisOfRecord: PreservedSpecimen
- **Type status:** Other material. **Occurrence:** catalogNumber: 674; recordNumber: 483; recordedBy: Ellemann, L; **Taxon:** scientificName: *Pennisetum setaceum* (Forssk.) Chiov.; kingdom: Plantae; family: Poaceae; genus: Pennisetum; specificEpithet: setaceum; scientificNameAuthorship: (Forssk.) Chiov.; **Location:** continent: Africa; country: Tanzania; stateProvince: Arusha; county: Ngorongoro; locality: Kelogi; verbatimLocality: Ngorongoro Conservation Area, Kelogi.; minimumElevationInMeters: 1550; decimalLatitude: -3.033; decimalLongitude: 35.283; **Event:** eventDate: 1993-05-03; **Record Level:** institutionCode: AAU; collectionCode: Herbarium; ownerInstitutionCode: AAU; basisOfRecord: PreservedSpecimen
- **Type status:** Other material. **Occurrence:** catalogNumber: DB0443; recordNumber: 13152; recordedBy: Greenway, PJ; Kanuri; Limbrey, H; Braun, H; **Taxon:** scientificName: *Pennisetum setaceum* (Forssk.) Chiov.; kingdom: Plantae; family: Poaceae; genus: Pennisetum; specificEpithet: setaceum; scientificNameAuthorship: (Forssk.) Chiov.; **Location:** continent: Africa; country: Tanzania; stateProvince: Mara; county: Serengeti; locality: Simba Kopjes; verbatimLocality: Serengeti plain; minimumElevationInMeters: 1524; decimalLatitude: -2.7; decimalLongitude: 34.916667; **Event:** eventDate: 1968-02-08; **Record Level:** institutionCode: SWRC; collectionCode: Herbarium; ownerInstitutionCode: SWRC; basisOfRecord: PreservedSpecimen

##### Distribution

Widespread

#### Pennisetum sphacelatum

(Nees) T.Durand & Schinz

Pennisetum schimperi A.Rich. | Cenchrus sphacelatus (Nees) Morrone

##### Materials

- **Type status:** Other material. **Occurrence:** catalogNumber: 555; recordNumber: 24308; recordedBy: Peterson, PM; Soreng, RJ; Romaschenko, K; Mbago, F; **Taxon:** scientificName: *Cenchrus sphacelatus* (Nees) Morrone; kingdom: Plantae; family: Poaceae; genus: Cenchrus; specificEpithet: sphacelatus; scientificNameAuthorship: (Nees) Morrone; **Location:** continent: Africa; country: Tanzania; stateProvince: Arusha; county: Ngorongoro; locality: Ngorongoro Crater; verbatimLocality: Ngorongoro Conservation Area, rim of Ngorongoro Crater (descent gate).; minimumElevationInMeters: 2168; decimalLatitude: -3.15462; decimalLongitude: 35.47717; **Event:** eventDate: 2012-06-19; **Record Level:** institutionCode: US; collectionCode: Herbarium; ownerInstitutionCode: US; basisOfRecord: PreservedSpecimen
- **Type status:** Other material. **Occurrence:** catalogNumber: K000984075; recordNumber: 601; recordedBy: Welch, JR; **Taxon:** scientificName: *Pennisetum schimperi* A.Rich.; kingdom: Plantae; family: Poaceae; genus: Pennisetum; specificEpithet: schimperi; scientificNameAuthorship: A.Rich.; **Location:** continent: Africa; country: Tanzania; stateProvince: Arusha; county: Ngorongoro; locality: Ngorongoro; verbatimLocality: Ngorongoro crater rim near windy Gap, 8 miles N. of Crater lodge; minimumElevationInMeters: 1829; decimalLatitude: -3.166667; decimalLongitude: 35.583333; **Event:** eventDate: 1964-06-18; **Record Level:** institutionCode: K; collectionCode: Herbarium; ownerInstitutionCode: K; basisOfRecord: PreservedSpecimen
- **Type status:** Other material. **Occurrence:** catalogNumber: K000766298; recordNumber: 5651; recordedBy: Newbould, JB; **Taxon:** scientificName: *Pennisetum schimperi* A.Rich.; kingdom: Plantae; family: Poaceae; genus: Pennisetum; specificEpithet: schimperi; scientificNameAuthorship: A.Rich.; **Location:** continent: Africa; country: Tanzania; stateProvince: Arusha; county: Ngorongoro; locality: Ngorongoro; verbatimLocality: Old Boma; minimumElevationInMeters: 2134; decimalLatitude: -3.166667; decimalLongitude: 35.583333; **Event:** eventDate: 1961-02-11; **Record Level:** institutionCode: K; collectionCode: Herbarium; ownerInstitutionCode: K; basisOfRecord: PreservedSpecimen
- **Type status:** Other material. **Occurrence:** catalogNumber: K000766300; recordNumber: 1670; recordedBy: Heady, AF; **Taxon:** scientificName: *Pennisetum schimperi* A.Rich.; kingdom: Plantae; family: Poaceae; genus: Pennisetum; specificEpithet: schimperi; scientificNameAuthorship: A.Rich.; **Location:** continent: Africa; country: Tanzania; stateProvince: Arusha; county: Ngorongoro; locality: Ngorongoro Crater; decimalLatitude: -3.166667; decimalLongitude: 35.583333; **Event:** eventDate: 1959-02-18; **Record Level:** institutionCode: K; collectionCode: Herbarium; ownerInstitutionCode: K; basisOfRecord: PreservedSpecimen
- **Type status:** Other material. **Occurrence:** catalogNumber: K000766299; recordNumber: 970; recordedBy: Pole Evans, IB; Erens, J; **Taxon:** scientificName: *Pennisetum schimperi* A.Rich.; kingdom: Plantae; family: Poaceae; genus: Pennisetum; specificEpithet: schimperi; scientificNameAuthorship: A.Rich.; **Location:** continent: Africa; country: Tanzania; stateProvince: Arusha; county: Ngorongoro; locality: Ngorongoro Crater; verbatimLocality: On the top ridge of the Ngorongoro crater.; decimalLatitude: -3.166667; decimalLongitude: 35.583333; **Event:** eventDate: 1938-06-24; **Record Level:** institutionCode: K; collectionCode: Herbarium; ownerInstitutionCode: K; basisOfRecord: PreservedSpecimen
- **Type status:** Other material. **Occurrence:** catalogNumber: DB0445; recordNumber: 124; recordedBy: Frame, GW; **Taxon:** scientificName: *Pennisetum sphacelatum* (Nees) T.Durand & Schinz; kingdom: Plantae; family: Poaceae; genus: Pennisetum; specificEpithet: sphacelatum; scientificNameAuthorship: (Nees) T.Durand & Schinz; **Location:** continent: Africa; country: Tanzania; stateProvince: Arusha; county: Ngorongoro; locality: Empakai Crater; verbatimLocality: Empakaai Crater, 1 mile southwest, along main road.; minimumElevationInMeters: 2300; decimalLatitude: -2.933333; decimalLongitude: 35.816667; **Event:** eventDate: 1973-05-03; **Record Level:** institutionCode: SWRC; collectionCode: Herbarium; ownerInstitutionCode: SWRC; basisOfRecord: PreservedSpecimen
- **Type status:** Other material. **Occurrence:** catalogNumber: 675; recordNumber: 665; recordedBy: Ellemann, L; **Taxon:** scientificName: *Pennisetum sphacelatum* (Nees) T.Durand & Schinz; kingdom: Plantae; family: Poaceae; genus: Pennisetum; specificEpithet: sphacelatum; scientificNameAuthorship: (Nees) T.Durand & Schinz; **Location:** continent: Africa; country: Tanzania; stateProvince: Arusha; county: Ngorongoro; locality: Oloronyo; verbatimLocality: Ngorongoro Conservation Area, Oloronyo,; minimumElevationInMeters: 2800; decimalLatitude: -3; decimalLongitude: 35.35; **Event:** eventDate: 1993-06-17; **Record Level:** institutionCode: AAU; collectionCode: Herbarium; ownerInstitutionCode: AAU; basisOfRecord: PreservedSpecimen
- **Type status:** Other material. **Occurrence:** catalogNumber: 676; recordNumber: 698; recordedBy: Ellemann, L; **Taxon:** scientificName: *Pennisetum sphacelatum* (Nees) T.Durand & Schinz; kingdom: Plantae; family: Poaceae; genus: Pennisetum; specificEpithet: sphacelatum; scientificNameAuthorship: (Nees) T.Durand & Schinz; **Location:** continent: Africa; country: Tanzania; stateProvince: Arusha; county: Ngorongoro; locality: Endulen; verbatimLocality: Ngorongoro Conservation Area, Ndeyan 4 km North-west of Endulen Village.; minimumElevationInMeters: 1900; decimalLatitude: -3.2; decimalLongitude: 35.283; **Event:** eventDate: 1993-07-16; **Record Level:** institutionCode: AAU; collectionCode: Herbarium; ownerInstitutionCode: AAU; basisOfRecord: PreservedSpecimen
- **Type status:** Other material. **Occurrence:** catalogNumber: 677; recordNumber: 910a; recordedBy: Ellemann, L; **Taxon:** scientificName: *Pennisetum sphacelatum* (Nees) T.Durand & Schinz; kingdom: Plantae; family: Poaceae; genus: Pennisetum; specificEpithet: sphacelatum; scientificNameAuthorship: (Nees) T.Durand & Schinz; **Location:** continent: Africa; country: Tanzania; stateProvince: Arusha; county: Ngorongoro; locality: Armakutian; verbatimLocality: Ngorongoro Conservation Area, Armakutian, above Alchorai and the upper water tank in Loomyoni.; minimumElevationInMeters: 2000; decimalLatitude: -3.2; decimalLongitude: 35.483; **Event:** eventDate: 1993-09-15; **Record Level:** institutionCode: AAU; collectionCode: Herbarium; ownerInstitutionCode: AAU; basisOfRecord: PreservedSpecimen
- **Type status:** Other material. **Occurrence:** catalogNumber: 1102; recordNumber: 124; recordedBy: Frame, GW; **Taxon:** scientificName: *Pennisetum sphacelatum* (Nees) T.Durand & Schinz; kingdom: Plantae; family: Poaceae; genus: Pennisetum; specificEpithet: sphacelatum; scientificNameAuthorship: (Nees) T.Durand & Schinz; **Location:** continent: Africa; country: Tanzania; stateProvince: Arusha; county: Ngorongoro; locality: Empakai Crater; verbatimLocality: Empakaai Crater, 1 mile southwest, along main road.; minimumElevationInMeters: 2300; decimalLatitude: -2.933333; decimalLongitude: 35.816667; **Event:** eventDate: 1973-05-03; **Record Level:** institutionCode: NHT; collectionCode: Herbarium; ownerInstitutionCode: NHT; basisOfRecord: PreservedSpecimen

##### Distribution

Tropical Africa

#### Pennisetum squamulatum

Fresen.

Cenchrus squamulatum (Fresen.) Morrone

##### Materials

- **Type status:** Other material. **Occurrence:** catalogNumber: K001087174; recordNumber: 13173; recordedBy: Greenway, PJ; Kanuri; Turner, M; **Taxon:** scientificName: *Pennisetum squamulatum* Fresen.; kingdom: Plantae; family: Poaceae; genus: Pennisetum; specificEpithet: squamulatum; scientificNameAuthorship: Fresen.; **Location:** continent: Africa; country: Tanzania; stateProvince: Shinyanga; locality: Barafu Kopjes; minimumElevationInMeters: 1768; decimalLatitude: -2.5; decimalLongitude: 35.416667; **Event:** eventDate: 1968-02-12; **Record Level:** institutionCode: K; collectionCode: Herbarium; ownerInstitutionCode: K; basisOfRecord: PreservedSpecimen
- **Type status:** Other material. **Occurrence:** catalogNumber: DB0449; recordNumber: 13173; recordedBy: Greenway, PJ; Kanuri; Turner, M; **Taxon:** scientificName: *Pennisetum squamulatum* Fresen.; kingdom: Plantae; family: Poaceae; genus: Pennisetum; specificEpithet: squamulatum; scientificNameAuthorship: Fresen.; **Location:** continent: Africa; country: Tanzania; stateProvince: Shinyanga; locality: Barafu Kopjes; minimumElevationInMeters: 1768; decimalLatitude: -2.5; decimalLongitude: 35.416667; **Event:** eventDate: 1968-02-12; **Record Level:** institutionCode: SWRC; collectionCode: Herbarium; ownerInstitutionCode: SWRC; basisOfRecord: PreservedSpecimen
- **Type status:** Other material. **Occurrence:** catalogNumber: K001087175; recordNumber: 6401; recordedBy: Newbould, JB; **Taxon:** scientificName: *Pennisetum squamulatum* Fresen.; kingdom: Plantae; family: Poaceae; genus: Pennisetum; specificEpithet: squamulatum; scientificNameAuthorship: Fresen.; **Location:** continent: Africa; country: Tanzania; stateProvince: Arusha; county: Ngorongoro; locality: Olkarien; minimumElevationInMeters: 1371; decimalLatitude: -2.6; decimalLongitude: 35.366667; **Event:** eventDate: 1962-12-20; **Record Level:** institutionCode: K; collectionCode: Herbarium; ownerInstitutionCode: K; basisOfRecord: PreservedSpecimen

##### Distribution

Tanzania, Kenya, Ethiopia & Eritrea

#### Pennisetum stramineum

Peter

Cenchrus stramineus (A. Peter) Morrone

##### Materials

- **Type status:** Other material. **Occurrence:** catalogNumber: DB0441; recordNumber: 43; recordedBy: Schmidt, W; **Taxon:** scientificName: *Cenchrus stramineus* (A. Peter) Morrone; kingdom: Plantae; family: Poaceae; genus: Cenchrus; specificEpithet: stramineus; scientificNameAuthorship: (A. Peter) Morrone; **Location:** continent: Africa; country: Tanzania; stateProvince: Mara; county: Serengeti; locality: Simba Kopjes; verbatimLocality: Simba Kopjes (North) Serengeti plains; decimalLatitude: -2.7; decimalLongitude: 34.916667; **Event:** eventDate: 1972-03-11; **Record Level:** institutionCode: SWRC; collectionCode: Herbarium; ownerInstitutionCode: SWRC; basisOfRecord: PreservedSpecimen
- **Type status:** Other material. **Occurrence:** catalogNumber: DB0439; recordNumber: 66; recordedBy: Belsky, PJ; **Taxon:** scientificName: *Cenchrus stramineus* (A. Peter) Morrone; kingdom: Plantae; family: Poaceae; genus: Cenchrus; specificEpithet: stramineus; scientificNameAuthorship: (A. Peter) Morrone; **Location:** continent: Africa; country: Tanzania; stateProvince: Mara; county: Serengeti; locality: Ndutu Lodge; verbatimLocality: Near road 50 m from Ndutu Lodge; decimalLatitude: -3.02; decimalLongitude: 34.996944; **Event:** eventDate: 1981-04-10; **Record Level:** institutionCode: SWRC; collectionCode: Herbarium; ownerInstitutionCode: SWRC; basisOfRecord: PreservedSpecimen
- **Type status:** Other material. **Occurrence:** catalogNumber: DB0438; recordNumber: 533; recordedBy: Frame, GW; **Taxon:** scientificName: *Cenchrus stramineus* (A. Peter) Morrone; kingdom: Plantae; family: Poaceae; genus: Cenchrus; specificEpithet: stramineus; scientificNameAuthorship: (A. Peter) Morrone; **Location:** continent: Africa; country: Tanzania; stateProvince: Arusha; county: Ngorongoro; locality: Empakai Crater; verbatimLocality: Ngorongoro Conservation area. Empakaai Crater, outer northern slope.; minimumElevationInMeters: 2200; decimalLatitude: -2.933333; decimalLongitude: 35.816667; **Event:** eventDate: 1974-02-01; **Record Level:** institutionCode: SWRC; collectionCode: Herbarium; ownerInstitutionCode: SWRC; basisOfRecord: PreservedSpecimen
- **Type status:** Other material. **Occurrence:** catalogNumber: DB0437; recordNumber: 230; recordedBy: Kreulen, AR; **Taxon:** scientificName: *Cenchrus stramineus* (A. Peter) Morrone; kingdom: Plantae; family: Poaceae; genus: Cenchrus; specificEpithet: stramineus; scientificNameAuthorship: (A. Peter) Morrone; **Location:** continent: Africa; country: Tanzania; stateProvince: Shinyanga; locality: Naabi Hill; verbatimLocality: Near Naabi Hill; decimalLatitude: -2.883333; decimalLongitude: 35.033333; **Event:** eventDate: 1974-01-07; **Record Level:** institutionCode: SWRC; collectionCode: Herbarium; ownerInstitutionCode: SWRC; basisOfRecord: PreservedSpecimen
- **Type status:** Other material. **Occurrence:** catalogNumber: 556; recordNumber: 24276; recordedBy: Peterson, PM; Soreng, RJ; Romaschenko, K; Mbago, F; **Taxon:** scientificName: *Cenchrus stramineus* (A. Peter) Morrone; kingdom: Plantae; family: Poaceae; genus: Cenchrus; specificEpithet: stramineus; scientificNameAuthorship: (A. Peter) Morrone; **Location:** continent: Africa; country: Tanzania; stateProvince: Shinyanga; locality: Naabi Hill Gate; verbatimLocality: Serengeti National Park, at 12 km NW of Naabi Hill Gate.; minimumElevationInMeters: 1651; decimalLatitude: -2.73597; decimalLongitude: 34.95284; **Event:** eventDate: 2012-06-16; **Record Level:** institutionCode: US; collectionCode: Herbarium; ownerInstitutionCode: US; basisOfRecord: PreservedSpecimen
- **Type status:** Other material. **Occurrence:** catalogNumber: K000984076; recordNumber: 9159; recordedBy: Greenway, PJ; **Taxon:** scientificName: *Pennisetum stramineum* Peter; kingdom: Plantae; family: Poaceae; genus: Pennisetum; specificEpithet: stramineum; scientificNameAuthorship: Peter; **Location:** continent: Africa; country: Tanzania; stateProvince: Shinyanga; locality: Naabi Hill; verbatimLocality: W. of Naabi Hill; minimumElevationInMeters: 1676; decimalLatitude: -2.883333; decimalLongitude: 35.033333; **Event:** eventDate: 1956-12-11; **Record Level:** institutionCode: K; collectionCode: Herbarium; ownerInstitutionCode: K; basisOfRecord: PreservedSpecimen
- **Type status:** Other material. **Occurrence:** catalogNumber: K000984077; recordNumber: 10053; recordedBy: Greenway, PJ; **Taxon:** scientificName: *Pennisetum stramineum* Peter; kingdom: Plantae; family: Poaceae; genus: Pennisetum; specificEpithet: stramineum; scientificNameAuthorship: Peter; **Location:** continent: Africa; country: Tanzania; stateProvince: Mara; county: Serengeti; locality: Seronera; verbatimLocality: Serengeti; minimumElevationInMeters: 1448; decimalLatitude: -2.45; decimalLongitude: 34.833333; **Event:** eventDate: 1961-04-14; **Record Level:** institutionCode: K; collectionCode: Herbarium; ownerInstitutionCode: K; basisOfRecord: PreservedSpecimen
- **Type status:** Other material. **Occurrence:** catalogNumber: K000984078; recordNumber: 1503; recordedBy: Heady, HF; **Taxon:** scientificName: *Pennisetum stramineum* Peter; kingdom: Plantae; family: Poaceae; genus: Pennisetum; specificEpithet: stramineum; scientificNameAuthorship: Peter; **Location:** continent: Africa; country: Tanzania; stateProvince: Arusha; county: Ngorongoro; locality: Ngorongoro Crater; decimalLatitude: -3.166667; decimalLongitude: 35.583333; **Event:** eventDate: 1959-02-15; **Record Level:** institutionCode: K; collectionCode: Herbarium; ownerInstitutionCode: K; basisOfRecord: PreservedSpecimen
- **Type status:** Other material. **Occurrence:** catalogNumber: K000984079; recordNumber: 341; recordedBy: Paulo, S; **Taxon:** scientificName: *Pennisetum stramineum* Peter; kingdom: Plantae; family: Poaceae; genus: Pennisetum; specificEpithet: stramineum; scientificNameAuthorship: Peter; **Location:** continent: Africa; country: Tanzania; stateProvince: Mara; county: Serengeti; locality: Serengeti; verbatimLocality: Central Serengeti plains; decimalLatitude: -2.333333; decimalLongitude: 34.833333; **Event:** eventDate: 1958-04-21; **Record Level:** institutionCode: K; collectionCode: Herbarium; ownerInstitutionCode: K; basisOfRecord: PreservedSpecimen
- **Type status:** Other material. **Occurrence:** catalogNumber: K000984080; recordNumber: 299; recordedBy: Paulo, S; **Taxon:** scientificName: *Pennisetum stramineum* Peter; kingdom: Plantae; family: Poaceae; genus: Pennisetum; specificEpithet: stramineum; scientificNameAuthorship: Peter; **Location:** continent: Africa; country: Tanzania; stateProvince: Mara; county: Serengeti; locality: Seronera; decimalLatitude: -2.45; decimalLongitude: 34.833333; **Event:** eventDate: 1958-04-18; **Record Level:** institutionCode: K; collectionCode: Herbarium; ownerInstitutionCode: K; basisOfRecord: PreservedSpecimen
- **Type status:** Other material. **Occurrence:** catalogNumber: DB0440; recordNumber: 10053; recordedBy: Greenway, PJ; **Taxon:** scientificName: *Pennisetum stramineum* Peter; kingdom: Plantae; family: Poaceae; genus: Pennisetum; specificEpithet: stramineum; scientificNameAuthorship: Peter; **Location:** continent: Africa; country: Tanzania; stateProvince: Mara; county: Serengeti; locality: Seronera; verbatimLocality: Serengeti; minimumElevationInMeters: 1448; decimalLatitude: -2.45; decimalLongitude: 34.833333; **Event:** eventDate: 1961-04-14; **Record Level:** institutionCode: SWRC; collectionCode: Herbarium; ownerInstitutionCode: SWRC; basisOfRecord: PreservedSpecimen

##### Distribution

Eastern Africa & Arabia

#### Pennisetum trachyphyllum

Pilg.

Cenchrus trachyphyllum (Pilg.) Morrone

##### Materials

- **Type status:** Other material. **Occurrence:** catalogNumber: DB0451; recordNumber: 238; recordedBy: Frame, GW; **Taxon:** scientificName: *Cenchrus trachyphyllum* (Pilg.) Morrone; kingdom: Plantae; family: Poaceae; genus: Cenchrus; specificEpithet: trachyphyllum; scientificNameAuthorship: (Pilg.) Morrone; **Location:** continent: Africa; country: Tanzania; stateProvince: Arusha; county: Ngorongoro; locality: Empakai Crater; verbatimLocality: Ngorongoro Conservation area north east slope,; minimumElevationInMeters: 2300; decimalLatitude: -2.933333; decimalLongitude: 35.816667; **Event:** eventDate: 1973-09-09; **Record Level:** institutionCode: SWRC; collectionCode: Herbarium; ownerInstitutionCode: SWRC; basisOfRecord: PreservedSpecimen

##### Distribution

Eastern & Central Africa

#### Perotis patens

Gand.

##### Materials

- **Type status:** Other material. **Occurrence:** catalogNumber: DB0478; recordNumber: 495; recordedBy: Schmidt, W; **Taxon:** scientificName: *Perotis patens* Gand.; kingdom: Plantae; family: Poaceae; genus: Perotis; specificEpithet: patens; scientificNameAuthorship: Gand.; **Location:** continent: Africa; country: Tanzania; stateProvince: Mara; county: Serengeti; locality: Bolgonja; verbatimLocality: Bologonja; decimalLatitude: -1.783333; decimalLongitude: 35.2; **Event:** eventDate: 1972-04-13; **Record Level:** institutionCode: SWRC; collectionCode: Herbarium; ownerInstitutionCode: SWRC; basisOfRecord: PreservedSpecimen
- **Type status:** Other material. **Occurrence:** catalogNumber: DB0474; recordNumber: 10198; recordedBy: Greenway, PJ; **Taxon:** scientificName: *Perotis patens* Gand.; kingdom: Plantae; family: Poaceae; genus: Perotis; specificEpithet: patens; scientificNameAuthorship: Gand.; **Location:** continent: Africa; country: Tanzania; stateProvince: Mara; county: Serengeti; locality: Seronera; verbatimLocality: Seronera to Kleins Camp Mile 55. 3; minimumElevationInMeters: 1140; decimalLatitude: -1.883333; decimalLongitude: 35.383333; **Event:** eventDate: 1961-05-17; **Record Level:** institutionCode: SWRC; collectionCode: Herbarium; ownerInstitutionCode: SWRC; basisOfRecord: PreservedSpecimen

##### Distribution

Tropical Africa

#### Phalaris arundinacea

L.

##### Materials

- **Type status:** Other material. **Occurrence:** catalogNumber: 679; recordNumber: s.n.; recordedBy: Metele, P; **Taxon:** scientificName: *Phalaris arundinacea* L.; kingdom: Plantae; family: Poaceae; genus: Phalaris; specificEpithet: arundinacea; scientificNameAuthorship: L.; **Location:** continent: Africa; country: Tanzania; stateProvince: Arusha; county: Ngorongoro; locality: Ngorongoro Crater; verbatimLocality: T2. Ngorongoro Crater, NCAA headquarters; minimumElevationInMeters: 2400; decimalLatitude: -3; decimalLongitude: 36; **Event:** eventDate: 1997-08-21; **Record Level:** institutionCode: MO; collectionCode: Herbarium; ownerInstitutionCode: MO; basisOfRecord: PreservedSpecimen
- **Type status:** Other material. **Occurrence:** catalogNumber: K001065326; recordNumber: 9147; recordedBy: Greenway, PJ; **Taxon:** scientificName: *Phalaris arundinacea* L.; kingdom: Plantae; family: Poaceae; genus: Phalaris; specificEpithet: arundinacea; scientificNameAuthorship: L.; **Location:** continent: Africa; country: Tanzania; stateProvince: Arusha; county: Ngorongoro; locality: Olmoti crater; verbatimLocality: Ol'Moti crater, floor on eastern side.; minimumElevationInMeters: 3048; decimalLatitude: -3; decimalLongitude: 35.633333; **Event:** eventDate: 1956-12-08; **Record Level:** institutionCode: K; collectionCode: Herbarium; ownerInstitutionCode: K; basisOfRecord: PreservedSpecimen
- **Type status:** Other material. **Occurrence:** catalogNumber: K001065328; recordNumber: 3270; recordedBy: Tanner, M; **Taxon:** scientificName: *Phalaris arundinacea* L.; kingdom: Plantae; family: Poaceae; genus: Phalaris; specificEpithet: arundinacea; scientificNameAuthorship: L.; **Location:** continent: Africa; country: Tanzania; stateProvince: Arusha; county: Ngorongoro; locality: Ngorongoro; minimumElevationInMeters: 1981; decimalLatitude: -3.166667; decimalLongitude: 35.583333; **Event:** eventDate: 1956-11-21; **Record Level:** institutionCode: K; collectionCode: Herbarium; ownerInstitutionCode: K; basisOfRecord: PreservedSpecimen
- **Type status:** Other material. **Occurrence:** catalogNumber: K001065327; recordNumber: 1682; recordedBy: Napper, D; **Taxon:** scientificName: *Phalaris arundinacea* L.; kingdom: Plantae; family: Poaceae; genus: Phalaris; specificEpithet: arundinacea; scientificNameAuthorship: L.; **Location:** continent: Africa; country: Tanzania; stateProvince: Arusha; county: Ngorongoro; locality: Ngorongoro; verbatimLocality: Upland grassland near Rest Camp, Ngorongoro.; decimalLatitude: -3.166667; decimalLongitude: 35.583333; **Event:** eventDate: 1963-01-07; **Record Level:** institutionCode: K; collectionCode: Herbarium; ownerInstitutionCode: K; basisOfRecord: PreservedSpecimen
- **Type status:** Other material. **Occurrence:** catalogNumber: K001065322; recordNumber: 187; recordedBy: Frame, GW; **Taxon:** scientificName: *Phalaris arundinacea* L.; kingdom: Plantae; family: Poaceae; genus: Phalaris; specificEpithet: arundinacea; scientificNameAuthorship: L.; **Location:** continent: Africa; country: Tanzania; stateProvince: Arusha; county: Ngorongoro; locality: Empakai Crater; verbatimLocality: Ngorongoro conservation area. Empakaai crater, deep depression, northeast of Jaeger Summit.; minimumElevationInMeters: 2200; decimalLatitude: -2.933333; decimalLongitude: 35.816667; **Event:** eventDate: 1973-06-23; **Record Level:** institutionCode: K; collectionCode: Herbarium; ownerInstitutionCode: K; basisOfRecord: PreservedSpecimen
- **Type status:** Other material. **Occurrence:** catalogNumber: DB0480; recordNumber: 187; recordedBy: Frame, GW; **Taxon:** scientificName: *Phalaris arundinacea* L.; kingdom: Plantae; family: Poaceae; genus: Phalaris; specificEpithet: arundinacea; scientificNameAuthorship: L.; **Location:** continent: Africa; country: Tanzania; stateProvince: Arusha; county: Ngorongoro; locality: Empakai Crater; verbatimLocality: Ngorongoro conservation area. Empakaai crater, deep depression, northeast of Jaeger Summit.; minimumElevationInMeters: 2200; decimalLatitude: -2.933333; decimalLongitude: 35.816667; **Event:** eventDate: 1973-06-23; **Record Level:** institutionCode: SWRC; collectionCode: Herbarium; ownerInstitutionCode: SWRC; basisOfRecord: PreservedSpecimen
- **Type status:** Other material. **Occurrence:** catalogNumber: K001065323; recordNumber: 2725; recordedBy: Chuwa, S; **Taxon:** scientificName: *Phalaris arundinacea* L.; kingdom: Plantae; family: Poaceae; genus: Phalaris; specificEpithet: arundinacea; scientificNameAuthorship: L.; **Location:** continent: Africa; country: Tanzania; stateProvince: Arusha; county: Ngorongoro; locality: Lemala Forest; verbatimLocality: Ngorongoro conservation area. Lemala forest (Rotian glade); minimumElevationInMeters: 2134; decimalLatitude: -3.116667; decimalLongitude: 35.666667; **Event:** eventDate: 1988-10-28; **Record Level:** institutionCode: K; collectionCode: Herbarium; ownerInstitutionCode: K; basisOfRecord: PreservedSpecimen
- **Type status:** Other material. **Occurrence:** catalogNumber: K001065324; recordNumber: 89016E; recordedBy: Pocs, T; Chuwa, S; **Taxon:** scientificName: *Phalaris arundinacea* L.; kingdom: Plantae; family: Poaceae; genus: Phalaris; specificEpithet: arundinacea; scientificNameAuthorship: L.; **Location:** continent: Africa; country: Tanzania; stateProvince: Arusha; county: Ngorongoro; locality: Rotian glade; verbatimLocality: Ngorongoro conservation area. Rotian glade, N.H.F.R.; minimumElevationInMeters: 2300; decimalLatitude: -3.116667; decimalLongitude: 35.666667; **Event:** eventDate: 1989-01-15; **Record Level:** institutionCode: K; collectionCode: Herbarium; ownerInstitutionCode: K; basisOfRecord: PreservedSpecimen
- **Type status:** Other material. **Occurrence:** catalogNumber: K001065325; recordNumber: 19559; recordedBy: Raynal, J; **Taxon:** scientificName: *Phalaris arundinacea* L.; kingdom: Plantae; family: Poaceae; genus: Phalaris; specificEpithet: arundinacea; scientificNameAuthorship: L.; **Location:** continent: Africa; country: Tanzania; stateProvince: Arusha; county: Ngorongoro; locality: Olmoti crater; verbatimLocality: Ngorongoro conservation area. Nainokanoka. Olmoti crater floor.; minimumElevationInMeters: 2800; decimalLatitude: -3; decimalLongitude: 35.633333; **Event:** eventDate: 1977-10-10; **Record Level:** institutionCode: K; collectionCode: Herbarium; ownerInstitutionCode: K; basisOfRecord: PreservedSpecimen

##### Distribution

Widespread

#### Phragmites australis

(Cav.) Trin. ex Steud.

##### Materials

- **Type status:** Other material. **Occurrence:** catalogNumber: K000984275; recordNumber: 10377; recordedBy: Greenway, PJ; **Taxon:** scientificName: *Phragmites australis* (Cav.) Trin. ex Steud.; kingdom: Plantae; family: Poaceae; genus: Phragmites; specificEpithet: australis; scientificNameAuthorship: (Cav.) Trin. ex Steud.; **Location:** continent: Africa; country: Tanzania; stateProvince: Mara; county: Serengeti; locality: Kirawira; decimalLatitude: -2.166667; decimalLongitude: 34.15; **Event:** eventDate: 1961-06-13; **Record Level:** institutionCode: K; collectionCode: Herbarium; ownerInstitutionCode: K; basisOfRecord: PreservedSpecimen

##### Distribution

Widespread

#### Poa annua

L.

##### Materials

- **Type status:** Other material. **Occurrence:** catalogNumber: K000984282; recordNumber: 12531; recordedBy: Greenway, PJ; Kanuri; **Taxon:** scientificName: *Poa annua* L.; kingdom: Plantae; family: Poaceae; genus: Poa; specificEpithet: annua; scientificNameAuthorship: L.; **Location:** continent: Africa; country: Tanzania; stateProvince: Arusha; county: Ngorongoro; locality: Ngorongoro Crater; verbatimLocality: rim, S. side; minimumElevationInMeters: 2256; decimalLatitude: -3.166667; decimalLongitude: 35.583333; **Event:** eventDate: 1966-07-05; **Record Level:** institutionCode: K; collectionCode: Herbarium; ownerInstitutionCode: K; basisOfRecord: PreservedSpecimen
- **Type status:** Other material. **Occurrence:** catalogNumber: 680; recordNumber: 24301; recordedBy: Peterson, PM; Soreng, RJ; Romaschenko, K; Mbago, F; **Taxon:** scientificName: *Poa annua* L.; kingdom: Plantae; family: Poaceae; genus: Poa; specificEpithet: annua; scientificNameAuthorship: L.; **Location:** continent: Africa; country: Tanzania; stateProvince: Arusha; county: Ngorongoro; locality: Ngorongoro Crater; verbatimLocality: Ngorongoro Conservation Area Headquarters, upper edge of crater.; minimumElevationInMeters: 2405; decimalLatitude: -3.24529; decimalLongitude: 35.48797; **Event:** eventDate: 2012-06-18; **Record Level:** institutionCode: US; collectionCode: Herbarium; ownerInstitutionCode: US; basisOfRecord: PreservedSpecimen

##### Distribution

Widespread

#### Poa leptoclada

Hochst. ex A.Rich.

##### Materials

- **Type status:** Other material. **Occurrence:** catalogNumber: K000984280; recordNumber: 6244; recordedBy: Newbould, JB; **Taxon:** scientificName: *Poa leptoclada* Hochst. ex A.Rich.; kingdom: Plantae; family: Poaceae; genus: Poa; specificEpithet: leptoclada; scientificNameAuthorship: Hochst. ex A.Rich.; **Location:** continent: Africa; country: Tanzania; stateProvince: Arusha; county: Ngorongoro; locality: Nainokanoka; verbatimLocality: Crater highlands; minimumElevationInMeters: 2591; decimalLatitude: -3.016667; decimalLongitude: 35.683333; **Event:** eventDate: 1962-07-30; **Record Level:** institutionCode: K; collectionCode: Herbarium; ownerInstitutionCode: K; basisOfRecord: PreservedSpecimen
- **Type status:** Other material. **Occurrence:** catalogNumber: K000984281; recordNumber: 9137; recordedBy: Greenway, PJ; **Taxon:** scientificName: *Poa leptoclada* Hochst. ex A.Rich.; kingdom: Plantae; family: Poaceae; genus: Poa; specificEpithet: leptoclada; scientificNameAuthorship: Hochst. ex A.Rich.; **Location:** continent: Africa; country: Tanzania; stateProvince: Arusha; county: Ngorongoro; locality: Empakai Crater; minimumElevationInMeters: 3018; decimalLatitude: -2.933333; decimalLongitude: 35.816667; **Event:** eventDate: 1956-12-07; **Record Level:** institutionCode: K; collectionCode: Herbarium; ownerInstitutionCode: K; basisOfRecord: PreservedSpecimen
- **Type status:** Other material. **Occurrence:** catalogNumber: 681; recordNumber: 24300; recordedBy: Peterson, PM; Soreng, RJ; Romaschenko, K; Mbago, F; **Taxon:** scientificName: *Poa leptoclada* Hochst. ex A.Rich.; kingdom: Plantae; family: Poaceae; genus: Poa; specificEpithet: leptoclada; scientificNameAuthorship: Hochst. ex A.Rich.; **Location:** continent: Africa; country: Tanzania; stateProvince: Arusha; county: Ngorongoro; locality: Ngorongoro Crater; verbatimLocality: Ngorongoro Conservation Area Headquarters, upper edge of crater.; minimumElevationInMeters: 2405; decimalLatitude: -3.24529; decimalLongitude: 35.48797; **Event:** eventDate: 2012-06-18; **Record Level:** institutionCode: US; collectionCode: Herbarium; ownerInstitutionCode: US; basisOfRecord: PreservedSpecimen
- **Type status:** Other material. **Occurrence:** catalogNumber: K001087127; recordNumber: 12530; recordedBy: Greenway, PJ; Kanuri; **Taxon:** scientificName: *Poa leptoclada* Hochst. ex A.Rich.; kingdom: Plantae; family: Poaceae; genus: Poa; specificEpithet: leptoclada; scientificNameAuthorship: Hochst. ex A.Rich.; **Location:** continent: Africa; country: Tanzania; stateProvince: Arusha; county: Ngorongoro; locality: Ngorongoro Crater; verbatimLocality: Ngorongoro crater rim, south side.; minimumElevationInMeters: 2256; decimalLatitude: -3.166667; decimalLongitude: 35.583333; **Event:** eventDate: 1966-07-05; **Record Level:** institutionCode: K; collectionCode: Herbarium; ownerInstitutionCode: K; basisOfRecord: PreservedSpecimen
- **Type status:** Other material. **Occurrence:** catalogNumber: K001087128; recordNumber: 3361; recordedBy: Greenway, PJ; **Taxon:** scientificName: *Poa leptoclada* Hochst. ex A.Rich.; kingdom: Plantae; family: Poaceae; genus: Poa; specificEpithet: leptoclada; scientificNameAuthorship: Hochst. ex A.Rich.; **Location:** continent: Africa; country: Tanzania; stateProvince: Arusha; county: Ngorongoro; locality: Ngorongoro Crater; verbatimLocality: On the south east slope of crater.; minimumElevationInMeters: 2134; decimalLatitude: -3.166667; decimalLongitude: 35.583333; **Event:** eventDate: 1933-02-22; **Record Level:** institutionCode: K; collectionCode: Herbarium; ownerInstitutionCode: K; basisOfRecord: PreservedSpecimen
- **Type status:** Other material. **Occurrence:** catalogNumber: K001087130; recordNumber: 96; recordedBy: Moreau; Moreau; **Taxon:** scientificName: *Poa leptoclada* Hochst. ex A.Rich.; kingdom: Plantae; family: Poaceae; genus: Poa; specificEpithet: leptoclada; scientificNameAuthorship: Hochst. ex A.Rich.; **Location:** continent: Africa; country: Tanzania; stateProvince: Arusha; county: Ngorongoro; locality: Oldeani Mt; verbatimLocality: Oldiani; minimumElevationInMeters: 2286; decimalLatitude: -3.266667; decimalLongitude: 35.433333; **Event:** eventDate: 1935-1; **Record Level:** institutionCode: K; collectionCode: Herbarium; ownerInstitutionCode: K; basisOfRecord: PreservedSpecimen
- **Type status:** Other material. **Occurrence:** catalogNumber: K001087129; recordNumber: P17; recordedBy: Frame, GW; **Taxon:** scientificName: *Poa leptoclada* Hochst. ex A.Rich.; kingdom: Plantae; family: Poaceae; genus: Poa; specificEpithet: leptoclada; scientificNameAuthorship: Hochst. ex A.Rich.; **Location:** continent: Africa; country: Tanzania; stateProvince: Arusha; county: Ngorongoro; locality: Empakai Crater; verbatimLocality: Empakaai crater, top of south rim.; minimumElevationInMeters: 2900; decimalLatitude: -2.933333; decimalLongitude: 35.816667; **Event:** eventDate: 1973-02-28; **Record Level:** institutionCode: K; collectionCode: Herbarium; ownerInstitutionCode: K; basisOfRecord: PreservedSpecimen
- **Type status:** Other material. **Occurrence:** catalogNumber: K001087131; recordNumber: 5681; recordedBy: Newbould, JB; **Taxon:** scientificName: *Poa leptoclada* Hochst. ex A.Rich.; kingdom: Plantae; family: Poaceae; genus: Poa; specificEpithet: leptoclada; scientificNameAuthorship: Hochst. ex A.Rich.; **Location:** continent: Africa; country: Tanzania; stateProvince: Arusha; county: Ngorongoro; locality: Ngorongoro FR; verbatimLocality: Ngorongoro forest reserve, near crater road.; decimalLatitude: 3.166667; decimalLongitude: 35.583333; **Event:** eventDate: 1961-03-04; **Record Level:** institutionCode: K; collectionCode: Herbarium; ownerInstitutionCode: K; basisOfRecord: PreservedSpecimen

##### Distribution

Tropical Africa & Arabia

#### Pogonarthria squarrosa

(Roem. & Schult.) Pilg.

##### Materials

- **Type status:** Other material. **Occurrence:** catalogNumber: K001087132; recordNumber: 10199; recordedBy: Greenway, PJ; **Taxon:** scientificName: *Pogonarthria squarrosa* (Roem. & Schult.) Pilg.; kingdom: Plantae; family: Poaceae; genus: Pogonarthria; specificEpithet: squarrosa; scientificNameAuthorship: (Roem. & Schult.) Pilg.; **Location:** continent: Africa; country: Tanzania; stateProvince: Mara; county: Serengeti; locality: Seronera-Klein's camp; verbatimLocality: Seronera to Klein's camp, mile 55.3.; minimumElevationInMeters: 1768; decimalLatitude: -1.769167; decimalLongitude: 35.3975; **Event:** eventDate: 1961-05-17; **Record Level:** institutionCode: K; collectionCode: Herbarium; ownerInstitutionCode: K; basisOfRecord: PreservedSpecimen

##### Distribution

Tropical Africa

#### Pogononeura biflora

Napper

##### Materials

- **Type status:** Isotype. **Occurrence:** catalogNumber: K000366539; recordNumber: 10091; recordedBy: Greenway, PJ; **Taxon:** scientificName: *Pogononeura biflora* Napper; kingdom: Plantae; family: Poaceae; genus: Pogononeura; specificEpithet: biflora; scientificNameAuthorship: Napper; **Location:** continent: Africa; country: Tanzania; stateProvince: Mara; county: Serengeti; locality: Seronera river; verbatimLocality: 80 km from Seronera via Musabi Guard Post.; minimumElevationInMeters: 1310; decimalLatitude: -2.166667; decimalLongitude: 35.5; **Event:** eventDate: 1961-04-25; **Record Level:** institutionCode: K; collectionCode: Herbarium; ownerInstitutionCode: K; basisOfRecord: PreservedSpecimen
- **Type status:** Holotype. **Occurrence:** catalogNumber: EA000000530; recordNumber: 10091; recordedBy: Greenway, PJ; **Taxon:** scientificName: *Pogononeura biflora* Napper; kingdom: Plantae; family: Poaceae; genus: Pogononeura; specificEpithet: biflora; scientificNameAuthorship: Napper; **Location:** continent: Africa; country: Tanzania; stateProvince: Mara; county: Serengeti; locality: Seronera river; verbatimLocality: 80 km from Seronera via Musabi Guard Post.; minimumElevationInMeters: 1310; decimalLatitude: -2.166667; decimalLongitude: 35.5; **Event:** eventDate: 1961-04-25; **Record Level:** institutionCode: EA; collectionCode: Herbarium; ownerInstitutionCode: EA; basisOfRecord: PreservedSpecimen
- **Type status:** Holotype. **Occurrence:** catalogNumber: EA000000531; recordNumber: 10091; recordedBy: Greenway, PJ; **Taxon:** scientificName: *Pogononeura biflora* Napper; kingdom: Plantae; family: Poaceae; genus: Pogononeura; specificEpithet: biflora; scientificNameAuthorship: Napper; **Location:** continent: Africa; country: Tanzania; stateProvince: Mara; county: Serengeti; locality: Seronera river; verbatimLocality: 80 km from Seronera via Musabi Guard Post.; minimumElevationInMeters: 1310; decimalLatitude: -2.166667; decimalLongitude: 35.5; **Event:** eventDate: 1961-04-25; **Record Level:** institutionCode: EA; collectionCode: Herbarium; ownerInstitutionCode: EA; basisOfRecord: PreservedSpecimen
- **Type status:** Isotype. **Occurrence:** catalogNumber: NY00431431; recordNumber: 10091; recordedBy: Greenway, PJ; **Taxon:** scientificName: *Pogononeura biflora* Napper; kingdom: Plantae; family: Poaceae; genus: Pogononeura; specificEpithet: biflora; scientificNameAuthorship: Napper; **Location:** continent: Africa; country: Tanzania; stateProvince: Mara; county: Serengeti; locality: Seronera river; verbatimLocality: 80 km from Seronera via Musabi Guard Post.; minimumElevationInMeters: 1310; decimalLatitude: -2.166667; decimalLongitude: 35.5; **Event:** eventDate: 1961-04-25; **Record Level:** institutionCode: NY; collectionCode: Herbarium; ownerInstitutionCode: NY; basisOfRecord: PreservedSpecimen
- **Type status:** Isotype. **Occurrence:** catalogNumber: PRE0784719-0; recordNumber: 10091; recordedBy: Greenway, PJ; **Taxon:** scientificName: *Pogononeura biflora* Napper; kingdom: Plantae; family: Poaceae; genus: Pogononeura; specificEpithet: biflora; scientificNameAuthorship: Napper; **Location:** continent: Africa; country: Tanzania; stateProvince: Mara; county: Serengeti; locality: Seronera river; verbatimLocality: 80 km from Seronera via Musabi Guard Post.; minimumElevationInMeters: 1310; decimalLatitude: -2.166667; decimalLongitude: 35.5; **Event:** eventDate: 1961-04-25; **Record Level:** institutionCode: PRE; collectionCode: Herbarium; ownerInstitutionCode: PRE; basisOfRecord: PreservedSpecimen
- **Type status:** Isotype. **Occurrence:** catalogNumber: PRE0696292-0; recordNumber: 10091; recordedBy: Greenway, PJ; **Taxon:** scientificName: *Pogononeura biflora* Napper; kingdom: Plantae; family: Poaceae; genus: Pogononeura; specificEpithet: biflora; scientificNameAuthorship: Napper; **Location:** continent: Africa; country: Tanzania; stateProvince: Mara; county: Serengeti; locality: Seronera river; verbatimLocality: 80 km from Seronera via Musabi Guard Post.; minimumElevationInMeters: 1310; decimalLatitude: -2.166667; decimalLongitude: 35.5; **Event:** eventDate: 1961-04-25; **Record Level:** institutionCode: PRE; collectionCode: Herbarium; ownerInstitutionCode: PRE; basisOfRecord: PreservedSpecimen
- **Type status:** Isotype. **Occurrence:** catalogNumber: US00289117; recordNumber: 10091; recordedBy: Greenway, PJ; **Taxon:** scientificName: *Pogononeura biflora* Napper; kingdom: Plantae; family: Poaceae; genus: Pogononeura; specificEpithet: biflora; scientificNameAuthorship: Napper; **Location:** continent: Africa; country: Tanzania; stateProvince: Mara; county: Serengeti; locality: Seronera river; verbatimLocality: 80 km from Seronera via Musabi Guard Post.; minimumElevationInMeters: 1310; decimalLatitude: -2.166667; decimalLongitude: 35.5; **Event:** eventDate: 1961-04-25; **Record Level:** institutionCode: US; collectionCode: Herbarium; ownerInstitutionCode: US; basisOfRecord: PreservedSpecimen
- **Type status:** Other material. **Occurrence:** catalogNumber: K001087133; recordNumber: 10608; recordedBy: Greenway, PJ; Turner, M; **Taxon:** scientificName: *Pogononeura biflora* Napper; kingdom: Plantae; family: Poaceae; genus: Pogononeura; specificEpithet: biflora; scientificNameAuthorship: Napper; **Location:** continent: Africa; country: Tanzania; stateProvince: Mara; county: Serengeti; locality: Kirawira; verbatimLocality: Kirawira guard post, Musoma district.; minimumElevationInMeters: 1128; decimalLatitude: -2.166667; decimalLongitude: 34.15; **Event:** eventDate: 1962-04-18; **Record Level:** institutionCode: K; collectionCode: Herbarium; ownerInstitutionCode: K; basisOfRecord: PreservedSpecimen
- **Type status:** Other material. **Occurrence:** catalogNumber: K001087134; recordNumber: 10620; recordedBy: Greenway, PJ; Turner, M; Watson, M; **Taxon:** scientificName: *Pogononeura biflora* Napper; kingdom: Plantae; family: Poaceae; genus: Pogononeura; specificEpithet: biflora; scientificNameAuthorship: Napper; **Location:** continent: Africa; country: Tanzania; stateProvince: Mara; county: Serengeti; locality: Musabi-Nyamuma; verbatimLocality: Musabi - Nyamuma Guard post. Musoma district.; minimumElevationInMeters: 1234; decimalLatitude: -2.333333; decimalLongitude: 34.333333; **Event:** eventDate: 1962-04-20; **Record Level:** institutionCode: K; collectionCode: Herbarium; ownerInstitutionCode: K; basisOfRecord: PreservedSpecimen

##### Distribution

Tanzania & Uganda

#### Polypogon monspeliensis

(L.) Desf.

##### Materials

- **Type status:** Other material. **Occurrence:** catalogNumber: K001087135; recordNumber: 1738; recordedBy: Tanner, M; **Taxon:** scientificName: *Polypogon monspeliensis* (L.) Desf.; kingdom: Plantae; family: Poaceae; genus: Polypogon; specificEpithet: monspeliensis; scientificNameAuthorship: (L.) Desf.; **Location:** continent: Africa; country: Tanzania; stateProvince: Arusha; county: Ngorongoro; locality: Klein's camp; verbatimLocality: Musabi - Nyamuma Guard post. Musoma district.; minimumElevationInMeters: 1676; decimalLatitude: -1.7; decimalLongitude: 35.216667; **Event:** eventDate: 1953-11-09; **Record Level:** institutionCode: K; collectionCode: Herbarium; ownerInstitutionCode: K; basisOfRecord: PreservedSpecimen
- **Type status:** Other material. **Occurrence:** catalogNumber: 866; recordNumber: H217; recordedBy: Herlocker, D; **Taxon:** scientificName: *Polypogon monspeliensis* (L.) Desf.; kingdom: Plantae; family: Poaceae; genus: Polypogon; specificEpithet: monspeliensis; scientificNameAuthorship: (L.) Desf.; **Location:** continent: Africa; country: Tanzania; stateProvince: Arusha; county: Ngorongoro; locality: Endulen; decimalLatitude: -3.183333; decimalLongitude: 35.15; **Event:** eventDate: 1965-11-17; **Record Level:** collectionCode: Herbarium; ownerInstitutionCode: ?; basisOfRecord: PreservedSpecimen
- **Type status:** Other material. **Occurrence:** catalogNumber: K001087136; recordNumber: 5; recordedBy: Ole Sayalal, P; **Taxon:** scientificName: *Polypogon monspeliensis* (L.) Desf.; kingdom: Plantae; family: Poaceae; genus: Polypogon; specificEpithet: monspeliensis; scientificNameAuthorship: (L.) Desf.; **Location:** continent: Africa; country: Tanzania; stateProvince: Mara; county: Serengeti; locality: Lengusa; verbatimLocality: T.N.P. Serengeti South; **Event:** eventDate: 1969-12-19; **Record Level:** institutionCode: K; collectionCode: Herbarium; ownerInstitutionCode: K; basisOfRecord: PreservedSpecimen
- **Type status:** Other material. **Occurrence:** catalogNumber: K001087137; recordNumber: 10657; recordedBy: Greenway, PJ; **Taxon:** scientificName: *Polypogon monspeliensis* (L.) Desf.; kingdom: Plantae; family: Poaceae; genus: Polypogon; specificEpithet: monspeliensis; scientificNameAuthorship: (L.) Desf.; **Location:** continent: Africa; country: Tanzania; stateProvince: Arusha; county: Ngorongoro; locality: Klein's camp; minimumElevationInMeters: 1707; decimalLatitude: -1.7; decimalLongitude: 35.216667; **Event:** eventDate: 1962-05-23; **Record Level:** institutionCode: K; collectionCode: Herbarium; ownerInstitutionCode: K; basisOfRecord: PreservedSpecimen
- **Type status:** Other material. **Occurrence:** catalogNumber: K001087138; recordNumber: 2897; recordedBy: Chuwa, S; **Taxon:** scientificName: *Polypogon monspeliensis* (L.) Desf.; kingdom: Plantae; family: Poaceae; genus: Polypogon; specificEpithet: monspeliensis; scientificNameAuthorship: (L.) Desf.; **Location:** continent: Africa; country: Tanzania; stateProvince: Arusha; county: Ngorongoro; locality: Endulen; verbatimLocality: Ngorongoro conservation area. Endulen (Oldogum river); minimumElevationInMeters: 1737; decimalLatitude: -3.183333; decimalLongitude: 35.15; **Event:** eventDate: 1989-10-28; **Record Level:** institutionCode: K; collectionCode: Herbarium; ownerInstitutionCode: K; basisOfRecord: PreservedSpecimen

##### Distribution

Widespread

#### Polypogon schimperianus

(Hochst. ex Steud.) Cope

##### Materials

- **Type status:** Other material. **Occurrence:** catalogNumber: K000984278; recordNumber: 6237; recordedBy: Newbould, JB; **Taxon:** scientificName: *Polypogon schimperianus* (Hochst. ex Steud.) Cope; kingdom: Plantae; family: Poaceae; genus: Polypogon; specificEpithet: schimperianus; scientificNameAuthorship: (Hochst. ex Steud.) Cope; **Location:** continent: Africa; country: Tanzania; stateProvince: Arusha; county: Ngorongoro; locality: Nainokanoka; verbatimLocality: crater highland; minimumElevationInMeters: 3048; decimalLatitude: -3.016667; decimalLongitude: 35.683333; **Event:** eventDate: 1962-07-29; **Record Level:** institutionCode: K; collectionCode: Herbarium; ownerInstitutionCode: K; basisOfRecord: PreservedSpecimen

##### Distribution

Tropical Africa & Arabia

#### Psilolemma jaegeri

(Pilg.) S.M.Phillips

##### Materials

- **Type status:** Other material. **Occurrence:** catalogNumber: 682; recordNumber: 24320; recordedBy: Peterson, PM; Soreng, RJ; Romaschenko, K; Mbago, F; **Taxon:** scientificName: *Psilolemma jaegeri* (Pilg.) S.M.Phillips; kingdom: Plantae; family: Poaceae; genus: Psilolemma; specificEpithet: jaegeri; scientificNameAuthorship: (Pilg.) S.M.Phillips; **Location:** continent: Africa; country: Tanzania; stateProvince: Arusha; county: Ngorongoro; locality: Lake Magadi; verbatimLocality: Ngorongoro Conservation Area, W side of Lake Magadi.; minimumElevationInMeters: 1739; decimalLatitude: -3.17713; decimalLongitude: 35.51548; **Event:** eventDate: 2012-06-19; **Record Level:** institutionCode: US; collectionCode: Herbarium; ownerInstitutionCode: US; basisOfRecord: PreservedSpecimen
- **Type status:** Other material. **Occurrence:** catalogNumber: K001087141; recordNumber: 9028; recordedBy: Greenway, PJ; **Taxon:** scientificName: *Psilolemma jaegeri* (Pilg.) S.M.Phillips; kingdom: Plantae; family: Poaceae; genus: Psilolemma; specificEpithet: jaegeri; scientificNameAuthorship: (Pilg.) S.M.Phillips; **Location:** continent: Africa; country: Tanzania; stateProvince: Shinyanga; locality: Mbalageti river; verbatimLocality: North west serengeti; decimalLatitude: -2.883333; decimalLongitude: 34.9; **Event:** eventDate: 1956-11-16; **Record Level:** institutionCode: K; collectionCode: Herbarium; ownerInstitutionCode: K; basisOfRecord: PreservedSpecimen
- **Type status:** Other material. **Occurrence:** catalogNumber: K001087140; recordNumber: 6314; recordedBy: Newbould, JB; **Taxon:** scientificName: *Psilolemma jaegeri* (Pilg.) S.M.Phillips; kingdom: Plantae; family: Poaceae; genus: Psilolemma; specificEpithet: jaegeri; scientificNameAuthorship: (Pilg.) S.M.Phillips; **Location:** continent: Africa; country: Tanzania; stateProvince: Mara; county: Serengeti; locality: Engare Nanyuki; verbatimLocality: East Serengeti; minimumElevationInMeters: 1768; decimalLatitude: -2.616667; decimalLongitude: 35.216667; **Event:** eventDate: 1962-11; **Record Level:** institutionCode: K; collectionCode: Herbarium; ownerInstitutionCode: K; basisOfRecord: PreservedSpecimen

##### Distribution

Tanzania, Kenya, Uganda & Democratic Republic of Congo

#### Schmidtia pappophoroides

Steud. ex J.A.Schmidt.

Schmidtia bulbosa Stapf

##### Materials

- **Type status:** Other material. **Occurrence:** catalogNumber: K001087139; recordNumber: 10301; recordedBy: Greenway, PJ; **Taxon:** scientificName: *Schmidtia pappophoroides* Steud. ex J.A.Schmidt.; kingdom: Plantae; family: Poaceae; genus: Schmidtia; specificEpithet: pappophoroides; scientificNameAuthorship: Steud. ex J.A.Schmidt.; **Location:** continent: Africa; country: Tanzania; stateProvince: Mara; county: Serengeti; locality: Seronera-Klein's camp; verbatimLocality: Seronera to Klein's camp, mile 55.3.; minimumElevationInMeters: 1798; decimalLatitude: -1.769167; decimalLongitude: 35.3975; **Event:** eventDate: 1961-05-17; **Record Level:** institutionCode: K; collectionCode: Herbarium; ownerInstitutionCode: K; basisOfRecord: PreservedSpecimen

##### Distribution

Tropical Africa & Asia

#### Setaria sp.

##### Materials

- **Type status:** Other material. **Occurrence:** catalogNumber: DB0647; recordNumber: 17; recordedBy: Vesey-FitzGerald, LDEF; **Taxon:** scientificName: Setaria; kingdom: Plantae; family: Poaceae; genus: Setaria; **Location:** continent: Africa; country: Tanzania; stateProvince: Arusha; county: Ngorongoro; locality: Olmoti crater; verbatimLocality: Crater Highlands Olmoti Crater; minimumElevationInMeters: 2700; decimalLatitude: -3; decimalLongitude: 35.083333; **Event:** eventDate: 1959-12-27; **Record Level:** institutionCode: SWRC; collectionCode: Herbarium; ownerInstitutionCode: SWRC; basisOfRecord: PreservedSpecimen

#### Setaria homonyma

(Steud.) Chiov.

##### Materials

- **Type status:** Other material. **Occurrence:** catalogNumber: K000984100; recordNumber: 83; recordedBy: Frame, GW; **Taxon:** scientificName: *Setaria homonyma* (Steud.) Chiov.; kingdom: Plantae; family: Poaceae; genus: Setaria; specificEpithet: homonyma; scientificNameAuthorship: (Steud.) Chiov.; **Location:** continent: Africa; country: Tanzania; stateProvince: Arusha; county: Ngorongoro; locality: Ngorongoro Crater; verbatimLocality: floor near south wall, 0.5 mile south of Lerai forest; decimalLatitude: -3.166667; decimalLongitude: 35.583333; **Event:** eventDate: 1973-03-10; **Record Level:** institutionCode: K; collectionCode: Herbarium; ownerInstitutionCode: K; basisOfRecord: PreservedSpecimen
- **Type status:** Other material. **Occurrence:** catalogNumber: K000984101; recordNumber: 10588; recordedBy: Greenway, PJ; **Taxon:** scientificName: *Setaria homonyma* (Steud.) Chiov.; kingdom: Plantae; family: Poaceae; genus: Setaria; specificEpithet: homonyma; scientificNameAuthorship: (Steud.) Chiov.; **Location:** continent: Africa; country: Tanzania; stateProvince: Shinyanga; locality: Moru Kopjes; minimumElevationInMeters: 1402; decimalLatitude: -2.75; decimalLongitude: 34.75; **Event:** eventDate: 1962-04-11; **Record Level:** institutionCode: K; collectionCode: Herbarium; ownerInstitutionCode: K; basisOfRecord: PreservedSpecimen
- **Type status:** Other material. **Occurrence:** catalogNumber: 1099; recordNumber: 83; recordedBy: Frame, GW; **Taxon:** scientificName: *Setaria homonyma* (Steud.) Chiov.; kingdom: Plantae; family: Poaceae; genus: Setaria; specificEpithet: homonyma; scientificNameAuthorship: (Steud.) Chiov.; **Location:** continent: Africa; country: Tanzania; stateProvince: Arusha; county: Ngorongoro; locality: Ngorongoro Crater; verbatimLocality: floor near south wall, 0.5 mile south of Lerai forest; decimalLatitude: -3.166667; decimalLongitude: 35.583333; **Event:** eventDate: 1973-03-10; **Record Level:** institutionCode: NHT; collectionCode: Herbarium; ownerInstitutionCode: NHT; basisOfRecord: PreservedSpecimen
- **Type status:** Other material. **Occurrence:** catalogNumber: 1130; recordNumber: EN663; recordedBy: Njau, E; Leliyo, G; Andrew, P; Bamford, M; **Taxon:** scientificName: *Setaria homonyma* (Steud.) Chiov.; kingdom: Plantae; family: Poaceae; genus: Setaria; specificEpithet: homonyma; scientificNameAuthorship: (Steud.) Chiov.; **Location:** continent: Africa; country: Tanzania; stateProvince: Arusha; county: Ngorongoro; locality: Enduleni; verbatimLocality: Ngorongoro conservation area, Enduleni, Olindogom stream.; minimumElevationInMeters: 1730; decimalLatitude: -3.217778; decimalLongitude: 35.268333; **Event:** eventDate: 2004-06-29; **Record Level:** institutionCode: NHT; collectionCode: Herbarium; ownerInstitutionCode: NHT; basisOfRecord: PreservedSpecimen

##### Distribution

Tropical Africa & Asia

#### Setaria incrassata

(Hochst.) Hack.

##### Materials

- **Type status:** Other material. **Occurrence:** catalogNumber: DB0585; recordNumber: 4651; recordedBy: Vesey-FitzGerald, LDEF; **Taxon:** scientificName: *Setaria incrassata* (Hochst.) Hack.; kingdom: Plantae; family: Poaceae; genus: Setaria; specificEpithet: incrassata; scientificNameAuthorship: (Hochst.) Hack.; **Location:** continent: Africa; country: Tanzania; stateProvince: Mara; county: Serengeti; locality: Kirawira; verbatimLocality: Kiriwira; minimumElevationInMeters: 1200; decimalLatitude: -2.166667; decimalLongitude: 34.15; **Event:** eventDate: 1965-04-25; **Record Level:** institutionCode: SWRC; collectionCode: Herbarium; ownerInstitutionCode: SWRC; basisOfRecord: PreservedSpecimen
- **Type status:** Other material. **Occurrence:** catalogNumber: 683; recordNumber: 374; recordedBy: Ellemann, L; **Taxon:** scientificName: *Setaria incrassata* (Hochst.) Hack.; kingdom: Plantae; family: Poaceae; genus: Setaria; specificEpithet: incrassata; scientificNameAuthorship: (Hochst.) Hack.; **Location:** continent: Africa; country: Tanzania; stateProvince: Arusha; county: Ngorongoro; locality: Endulen; verbatimLocality: Ngorongoro Conservation Area. Endulen Hospital.; minimumElevationInMeters: 1900; decimalLatitude: -3.2; decimalLongitude: 35.266; **Event:** eventDate: 1993-04-20; **Record Level:** institutionCode: AAU; collectionCode: Herbarium; ownerInstitutionCode: AAU; basisOfRecord: PreservedSpecimen
- **Type status:** Other material. **Occurrence:** catalogNumber: K000765110; recordNumber: 433; recordedBy: Paulo, S; **Taxon:** scientificName: *Setaria incrassata* (Hochst.) Hack.; kingdom: Plantae; family: Poaceae; genus: Setaria; specificEpithet: incrassata; scientificNameAuthorship: (Hochst.) Hack.; **Location:** continent: Africa; country: Tanzania; stateProvince: Mara; county: Serengeti; locality: Serengeti; verbatimLocality: Masapi Serengeti area; decimalLatitude: -2.333333; decimalLongitude: 34.833333; **Event:** eventDate: 1958-05-09; **Record Level:** institutionCode: K; collectionCode: Herbarium; ownerInstitutionCode: K; basisOfRecord: PreservedSpecimen

##### Distribution

Tropical Africa

#### Setaria kagerensis

Mez

##### Materials

- **Type status:** Other material. **Occurrence:** catalogNumber: K000984102; recordNumber: 9946; recordedBy: Greenway, PJ; **Taxon:** scientificName: *Setaria kagerensis* Mez; kingdom: Plantae; family: Poaceae; genus: Setaria; specificEpithet: kagerensis; scientificNameAuthorship: Mez; **Location:** continent: Africa; country: Tanzania; stateProvince: Mara; county: Serengeti; locality: Seronera; verbatimLocality: Seronera to Banagi; minimumElevationInMeters: 1524; decimalLatitude: -2.45; decimalLongitude: 34.833333; **Event:** eventDate: 1961-03-30; **Record Level:** institutionCode: K; collectionCode: Herbarium; ownerInstitutionCode: K; basisOfRecord: PreservedSpecimen
- **Type status:** Other material. **Occurrence:** catalogNumber: K000984103; recordNumber: 5973; recordedBy: Newbould, JB; **Taxon:** scientificName: *Setaria kagerensis* Mez; kingdom: Plantae; family: Poaceae; genus: Setaria; specificEpithet: kagerensis; scientificNameAuthorship: Mez; **Location:** continent: Africa; country: Tanzania; stateProvince: Arusha; county: Ngorongoro; locality: Ngorongoro Crater; verbatimLocality: Olairadad S. side of Ngorongoro Crater wall.; minimumElevationInMeters: 2012; decimalLatitude: -3.166667; decimalLongitude: 35.583333; **Event:** eventDate: 1962-02-02; **Record Level:** institutionCode: K; collectionCode: Herbarium; ownerInstitutionCode: K; basisOfRecord: PreservedSpecimen
- **Type status:** Other material. **Occurrence:** catalogNumber: DB0648; recordNumber: 9946; recordedBy: Greenway, PJ; **Taxon:** scientificName: *Setaria kagerensis* Mez; kingdom: Plantae; family: Poaceae; genus: Setaria; specificEpithet: kagerensis; scientificNameAuthorship: Mez; **Location:** continent: Africa; country: Tanzania; stateProvince: Mara; county: Serengeti; locality: Seronera; verbatimLocality: Seronera to Banagi; minimumElevationInMeters: 1524; decimalLatitude: -2.45; decimalLongitude: 34.833333; **Event:** eventDate: 1961-03-30; **Record Level:** institutionCode: SWRC; collectionCode: Herbarium; ownerInstitutionCode: SWRC; basisOfRecord: PreservedSpecimen

##### Distribution

Tropical Africa

#### Setaria longiseta

P.Beauv.

##### Materials

- **Type status:** Other material. **Occurrence:** catalogNumber: DB0620; recordNumber: 732a; recordedBy: Schmidt, W; **Taxon:** scientificName: *Setaria longiseta* P.Beauv.; kingdom: Plantae; family: Poaceae; genus: Setaria; specificEpithet: longiseta; scientificNameAuthorship: P.Beauv.; **Location:** continent: Africa; country: Tanzania; stateProvince: Mara; county: Serengeti; locality: Bolgonja; verbatimLocality: Bologonja; decimalLatitude: -1.783333; decimalLongitude: 35.2; **Event:** eventDate: 1972-04-24; **Record Level:** institutionCode: SWRC; collectionCode: Herbarium; ownerInstitutionCode: SWRC; basisOfRecord: PreservedSpecimen
- **Type status:** Other material. **Occurrence:** catalogNumber: DB0619; recordNumber: 614; recordedBy: Schmidt, W; **Taxon:** scientificName: *Setaria longiseta* P.Beauv.; kingdom: Plantae; family: Poaceae; genus: Setaria; specificEpithet: longiseta; scientificNameAuthorship: P.Beauv.; **Location:** continent: Africa; country: Tanzania; stateProvince: Mara; county: Serengeti; locality: Bolgonja; verbatimLocality: Balogonja; decimalLatitude: -1.783333; decimalLongitude: 35.2; **Event:** eventDate: 1972-04-10; **Record Level:** institutionCode: SWRC; collectionCode: Herbarium; ownerInstitutionCode: SWRC; basisOfRecord: PreservedSpecimen

##### Distribution

Tropical Africa

#### Setaria megaphylla

(Steud.) T.Durand & Schinz

##### Materials

- **Type status:** Other material. **Occurrence:** catalogNumber: K000984106; recordNumber: 284; recordedBy: Staples, RR; **Taxon:** scientificName: *Setaria megaphylla* (Steud.) T.Durand & Schinz; kingdom: Plantae; family: Poaceae; genus: Setaria; specificEpithet: megaphylla; scientificNameAuthorship: (Steud.) T.Durand & Schinz; **Location:** continent: Africa; country: Tanzania; stateProvince: Arusha; county: Ngorongoro; locality: Ngorongoro; minimumElevationInMeters: 2134; decimalLatitude: -3.166667; decimalLongitude: 35.583333; **Event:** eventDate: 1930-07-27; **Record Level:** institutionCode: K; collectionCode: Herbarium; ownerInstitutionCode: K; basisOfRecord: PreservedSpecimen
- **Type status:** Other material. **Occurrence:** catalogNumber: K000984107; recordNumber: 10757; recordedBy: Greenway, PJ; **Taxon:** scientificName: *Setaria megaphylla* (Steud.) T.Durand & Schinz; kingdom: Plantae; family: Poaceae; genus: Setaria; specificEpithet: megaphylla; scientificNameAuthorship: (Steud.) T.Durand & Schinz; **Location:** continent: Africa; country: Tanzania; stateProvince: Mara; county: Serengeti; locality: Bolgonja river; minimumElevationInMeters: 1463; decimalLatitude: -1.783333; decimalLongitude: 35.2; **Event:** eventDate: 1962-08-18; **Record Level:** institutionCode: K; collectionCode: Herbarium; ownerInstitutionCode: K; basisOfRecord: PreservedSpecimen
- **Type status:** Other material. **Occurrence:** catalogNumber: DB0624; recordNumber: 698; recordedBy: Herlocker, D; Asao, M; **Taxon:** scientificName: *Setaria megaphylla* (Steud.) T.Durand & Schinz; kingdom: Plantae; family: Poaceae; genus: Setaria; specificEpithet: megaphylla; scientificNameAuthorship: (Steud.) T.Durand & Schinz; **Location:** continent: Africa; country: Tanzania; stateProvince: Mara; county: Serengeti; locality: Kogatende; verbatimLocality: Kogatende - Nyamburi; decimalLatitude: -1.583333; decimalLongitude: 34.933333; **Record Level:** institutionCode: SWRC; collectionCode: Herbarium; ownerInstitutionCode: SWRC; basisOfRecord: PreservedSpecimen
- **Type status:** Other material. **Occurrence:** catalogNumber: DB0622; recordNumber: 10757; recordedBy: Greenway, PJ; **Taxon:** scientificName: *Setaria megaphylla* (Steud.) T.Durand & Schinz; kingdom: Plantae; family: Poaceae; genus: Setaria; specificEpithet: megaphylla; scientificNameAuthorship: (Steud.) T.Durand & Schinz; **Location:** continent: Africa; country: Tanzania; stateProvince: Mara; county: Serengeti; locality: Bolgonja river; minimumElevationInMeters: 1463; decimalLatitude: -1.783333; decimalLongitude: 35.2; **Event:** eventDate: 1962-08-18; **Record Level:** institutionCode: SWRC; collectionCode: Herbarium; ownerInstitutionCode: SWRC; basisOfRecord: PreservedSpecimen
- **Type status:** Other material. **Occurrence:** catalogNumber: 1131; recordNumber: 2793; recordedBy: Chuwa, S; **Taxon:** scientificName: *Setaria megaphylla* (Steud.) T.Durand & Schinz; kingdom: Plantae; family: Poaceae; genus: Setaria; specificEpithet: megaphylla; scientificNameAuthorship: (Steud.) T.Durand & Schinz; **Location:** continent: Africa; country: Tanzania; stateProvince: Arusha; county: Ngorongoro; locality: Ngorongoro Crater; verbatimLocality: Crater wall.; minimumElevationInMeters: 1890; decimalLatitude: -3.166667; decimalLongitude: 35.583333; **Event:** eventDate: 1989-06-01; **Record Level:** institutionCode: NHT; collectionCode: Herbarium; ownerInstitutionCode: NHT; basisOfRecord: PreservedSpecimen

##### Distribution

Tropical Africa & Arabia

#### Setaria orthosticha

K.Schum. ex R.A.W.Herrm.

##### Materials

- **Type status:** Other material. **Occurrence:** catalogNumber: 1135; recordNumber: EN545; recordedBy: Njau, E; Leliyo, G; Andrew, P; Bamford, M; **Taxon:** scientificName: *Setaria orthosticha* K.Schum. ex R.A.W.Herrm.; kingdom: Plantae; family: Poaceae; genus: Setaria; specificEpithet: orthosticha; scientificNameAuthorship: K.Schum. ex R.A.W.Herrm.; **Location:** continent: Africa; country: Tanzania; stateProvince: Arusha; county: Ngorongoro; locality: Enduleni; verbatimLocality: Ngorongoro conservation area, Gurusi river, west of Enduleni.; minimumElevationInMeters: 1700; decimalLatitude: -3.211111; decimalLongitude: 35.220556; **Event:** eventDate: 2004-06-25; **Record Level:** institutionCode: NHT; collectionCode: Herbarium; ownerInstitutionCode: NHT; basisOfRecord: PreservedSpecimen

##### Distribution

Tropical Africa

#### Setaria poiretiana

(Schult.) Kunth

Setaria caudula Stapf

##### Materials

- **Type status:** Other material. **Occurrence:** catalogNumber: K000984105; recordNumber: 94; recordedBy: Moreau; **Taxon:** scientificName: *Setaria caudula* Stapf; kingdom: Plantae; family: Poaceae; genus: Setaria; specificEpithet: caudula; scientificNameAuthorship: Stapf; **Location:** continent: Africa; country: Tanzania; stateProvince: Arusha; county: Ngorongoro; locality: Ngorongoro; minimumElevationInMeters: 2286; decimalLatitude: -3.166667; decimalLongitude: 35.583333; **Event:** eventDate: 1935-1; **Record Level:** institutionCode: K; collectionCode: Herbarium; ownerInstitutionCode: K; basisOfRecord: PreservedSpecimen
- **Type status:** Other material. **Occurrence:** catalogNumber: K000984104; recordNumber: 2794; recordedBy: Chuwa, S; **Taxon:** scientificName: *Setaria poiretiana* (Schult.) Kunth; kingdom: Plantae; family: Poaceae; genus: Setaria; specificEpithet: poiretiana; scientificNameAuthorship: (Schult.) Kunth; **Location:** continent: Africa; country: Tanzania; stateProvince: Arusha; county: Ngorongoro; locality: Ngorongoro Crater; verbatimLocality: Wall along the road leading up from crater; minimumElevationInMeters: 1890; decimalLatitude: -3.166667; decimalLongitude: 35.583333; **Event:** eventDate: 1989-06-01; **Record Level:** institutionCode: K; collectionCode: Herbarium; ownerInstitutionCode: K; basisOfRecord: PreservedSpecimen
- **Type status:** Other material. **Occurrence:** catalogNumber: 1098; recordNumber: 2794; recordedBy: Chuwa, S; **Taxon:** scientificName: *Setaria poiretiana* (Schult.) Kunth; kingdom: Plantae; family: Poaceae; genus: Setaria; specificEpithet: poiretiana; scientificNameAuthorship: (Schult.) Kunth; **Location:** continent: Africa; country: Tanzania; stateProvince: Arusha; county: Ngorongoro; locality: Ngorongoro Crater; verbatimLocality: Wall along the road leading up from crater; minimumElevationInMeters: 1890; decimalLatitude: -3.166667; decimalLongitude: 35.583333; **Event:** eventDate: 1989-06-01; **Record Level:** institutionCode: NHT; collectionCode: Herbarium; ownerInstitutionCode: NHT; basisOfRecord: PreservedSpecimen

##### Distribution

Widespread

#### Setaria pumila

(Poir.) Roem. & Schult.

Setaria pallide-fusca (Schumach.) Stapf & C.E.Hubb

##### Materials

- **Type status:** Other material. **Occurrence:** catalogNumber: K000984111; recordNumber: 10107; recordedBy: Greenway, PJ; **Taxon:** scientificName: *Setaria pallide-fusca* (Schumach.) Stapf & C.E.Hubb; kingdom: Plantae; family: Poaceae; genus: Setaria; specificEpithet: pallide-fusca; scientificNameAuthorship: (Schumach.) Stapf & C.E.Hubb; **Location:** continent: Africa; country: Tanzania; stateProvince: Mara; county: Serengeti; locality: Seronera; verbatimLocality: Seronera to Simiyu Guard Post and return, Mile 41.3; minimumElevationInMeters: 1554; decimalLatitude: -2.45; decimalLongitude: 34.833333; **Event:** eventDate: 1961-04-27; **Record Level:** institutionCode: K; collectionCode: Herbarium; ownerInstitutionCode: K; basisOfRecord: PreservedSpecimen
- **Type status:** Other material. **Occurrence:** catalogNumber: K000984112; recordNumber: 10545; recordedBy: Greenway, PJ; **Taxon:** scientificName: *Setaria pallide-fusca* (Schumach.) Stapf & C.E.Hubb; kingdom: Plantae; family: Poaceae; genus: Setaria; specificEpithet: pallide-fusca; scientificNameAuthorship: (Schumach.) Stapf & C.E.Hubb; **Location:** continent: Africa; country: Tanzania; stateProvince: Shinyanga; locality: Beacon Area; verbatimLocality: S.W. of Beacon area; minimumElevationInMeters: 1554; decimalLatitude: -3.283333; decimalLongitude: 34.8; **Event:** eventDate: 1962-03-26; **Record Level:** institutionCode: K; collectionCode: Herbarium; ownerInstitutionCode: K; basisOfRecord: PreservedSpecimen
- **Type status:** Other material. **Occurrence:** catalogNumber: DB0640; recordNumber: 10107; recordedBy: Greenway, PJ; **Taxon:** scientificName: *Setaria pallide-fusca* (Schumach.) Stapf & C.E.Hubb; kingdom: Plantae; family: Poaceae; genus: Setaria; specificEpithet: pallide-fusca; scientificNameAuthorship: (Schumach.) Stapf & C.E.Hubb; **Location:** continent: Africa; country: Tanzania; stateProvince: Mara; county: Serengeti; locality: Seronera; verbatimLocality: Seronera to Simiyu Guard Post and return, Mile 41.3; minimumElevationInMeters: 1554; decimalLatitude: -2.45; decimalLongitude: 34.833333; **Event:** eventDate: 1961-04-27; **Record Level:** institutionCode: SWRC; collectionCode: Herbarium; ownerInstitutionCode: SWRC; basisOfRecord: PreservedSpecimen
- **Type status:** Other material. **Occurrence:** catalogNumber: DB0642; recordNumber: 10545; recordedBy: Greenway, PJ; **Taxon:** scientificName: *Setaria pallide-fusca* (Schumach.) Stapf & C.E.Hubb; kingdom: Plantae; family: Poaceae; genus: Setaria; specificEpithet: pallide-fusca; scientificNameAuthorship: (Schumach.) Stapf & C.E.Hubb; **Location:** continent: Africa; country: Tanzania; stateProvince: Shinyanga; locality: Beacon Area; verbatimLocality: S.W. of Beacon area; minimumElevationInMeters: 1554; decimalLatitude: -3.283333; decimalLongitude: 34.8; **Event:** eventDate: 1962-03-26; **Record Level:** institutionCode: SWRC; collectionCode: Herbarium; ownerInstitutionCode: SWRC; basisOfRecord: PreservedSpecimen
- **Type status:** Other material. **Occurrence:** catalogNumber: DB0641; recordNumber: 108; recordedBy: Schmidt, W; **Taxon:** scientificName: *Setaria pumila* (Poir.) Roem. & Schult.; kingdom: Plantae; family: Poaceae; genus: Setaria; specificEpithet: pumila; scientificNameAuthorship: (Poir.) Roem. & Schult.; **Location:** continent: Africa; country: Tanzania; stateProvince: Mara; county: Serengeti; locality: Serengeti Plains; verbatimLocality: South of Seronera Serengeti plains; decimalLatitude: -2.333333; decimalLongitude: 34.833333; **Event:** eventDate: 1972-03-14; **Record Level:** institutionCode: SWRC; collectionCode: Herbarium; ownerInstitutionCode: SWRC; basisOfRecord: PreservedSpecimen
- **Type status:** Other material. **Occurrence:** catalogNumber: DB0639; recordNumber: 10552; recordedBy: Greenway, PJ; **Taxon:** scientificName: *Setaria pumila* (Poir.) Roem. & Schult.; kingdom: Plantae; family: Poaceae; genus: Setaria; specificEpithet: pumila; scientificNameAuthorship: (Poir.) Roem. & Schult.; **Location:** continent: Africa; country: Tanzania; stateProvince: Mara; county: Serengeti; locality: Serengeti Plains; verbatimLocality: South West Beacon Area; minimumElevationInMeters: 1500; decimalLatitude: -2.333333; decimalLongitude: 34.833333; **Event:** eventDate: 1962-03-27; **Record Level:** institutionCode: SWRC; collectionCode: Herbarium; ownerInstitutionCode: SWRC; basisOfRecord: PreservedSpecimen
- **Type status:** Other material. **Occurrence:** catalogNumber: 684; recordNumber: 369; recordedBy: Ellemann, L; **Taxon:** scientificName: *Setaria pumila* (Poir.) Roem. & Schult.; kingdom: Plantae; family: Poaceae; genus: Setaria; specificEpithet: pumila; scientificNameAuthorship: (Poir.) Roem. & Schult.; **Location:** continent: Africa; country: Tanzania; stateProvince: Arusha; county: Ngorongoro; locality: Endulen; verbatimLocality: Ngorongoro Conservation Area. Endulen Hospital.; minimumElevationInMeters: 1900; decimalLatitude: -3.2; decimalLongitude: 35.266; **Event:** eventDate: 1993-04-20; **Record Level:** institutionCode: AAU; collectionCode: Herbarium; ownerInstitutionCode: AAU; basisOfRecord: PreservedSpecimen
- **Type status:** Other material. **Occurrence:** catalogNumber: 1133; recordNumber: 1138; recordedBy: Mollel, NP; Mboya, EI; **Taxon:** scientificName: *Setaria pumila* (Poir.) Roem. & Schult.; kingdom: Plantae; family: Poaceae; genus: Setaria; specificEpithet: pumila; scientificNameAuthorship: (Poir.) Roem. & Schult.; **Location:** continent: Africa; country: Tanzania; stateProvince: Arusha; county: Ngorongoro; locality: Laetoli; verbatimLocality: Esere-Laetoli road, Laetoli road, 900m, from the junction to the footprints site.; minimumElevationInMeters: 1625; decimalLatitude: -3.291389; decimalLongitude: 35.173889; **Event:** eventDate: 2008-04-08; **Record Level:** institutionCode: NHT; collectionCode: Herbarium; ownerInstitutionCode: NHT; basisOfRecord: PreservedSpecimen
- **Type status:** Other material. **Occurrence:** catalogNumber: 1134; recordNumber: 1138; recordedBy: Mollel, NP; Mboya, EI; **Taxon:** scientificName: *Setaria pumila* (Poir.) Roem. & Schult.; kingdom: Plantae; family: Poaceae; genus: Setaria; specificEpithet: pumila; scientificNameAuthorship: (Poir.) Roem. & Schult.; **Location:** continent: Africa; country: Tanzania; stateProvince: Arusha; county: Ngorongoro; locality: Laetoli; verbatimLocality: Esere-Laetoli road, Laetoli road, 900m, from the junction to the footprints site.; minimumElevationInMeters: 1625; decimalLatitude: -3.291389; decimalLongitude: 35.173889; **Event:** eventDate: 2008-04-08; **Record Level:** institutionCode: NCAA; collectionCode: Herbarium; ownerInstitutionCode: NCAA; basisOfRecord: PreservedSpecimen

##### Distribution

Widespread

#### Setaria sphacelata

(Schumach.) Stapf & C.E.Hubb ex Moss

Setaria sphacelata (Schumach.) Stapf & C.E.Hubb ex Moss var. torta (Stapf) Clayton | Setaria sphacelata (Schumach.) Stapf & C.E.Hubb ex Moss var. sericea (Stapf) Clayton

##### Materials

- **Type status:** Other material. **Occurrence:** catalogNumber: K000984116; recordNumber: 36; recordedBy: Brooks, GP; **Taxon:** scientificName: *Setaria sphacelata* (Schumach.) Stapf & C.E.Hubb ex Moss; kingdom: Plantae; family: Poaceae; genus: Setaria; specificEpithet: sphacelata; scientificNameAuthorship: (Schumach.) Stapf & C.E.Hubb ex Moss; **Location:** continent: Africa; country: Tanzania; stateProvince: Mara; county: Serengeti; locality: Ikoma; minimumElevationInMeters: 1371; decimalLatitude: -2.066667; decimalLongitude: 34.616667; **Event:** eventDate: 1954-02-19; **Record Level:** institutionCode: K; collectionCode: Herbarium; ownerInstitutionCode: K; basisOfRecord: PreservedSpecimen
- **Type status:** Other material. **Occurrence:** catalogNumber: DB0606; recordNumber: 534; recordedBy: Frame, GW; **Taxon:** scientificName: *Setaria sphacelata* (Schumach.) Stapf & C.E.Hubb ex Moss; kingdom: Plantae; family: Poaceae; genus: Setaria; specificEpithet: sphacelata; scientificNameAuthorship: (Schumach.) Stapf & C.E.Hubb ex Moss; **Location:** continent: Africa; country: Tanzania; stateProvince: Arusha; county: Ngorongoro; locality: Empakai Crater; verbatimLocality: Empakai Crater; decimalLatitude: -2.933333; decimalLongitude: 35.816667; **Event:** eventDate: 1974-02-01; **Record Level:** institutionCode: SWRC; collectionCode: Herbarium; ownerInstitutionCode: SWRC; basisOfRecord: PreservedSpecimen
- **Type status:** Other material. **Occurrence:** catalogNumber: DB0596; recordNumber: 5; recordedBy: Frame, GW; **Taxon:** scientificName: *Setaria sphacelata* (Schumach.) Stapf & C.E.Hubb ex Moss; kingdom: Plantae; family: Poaceae; genus: Setaria; specificEpithet: sphacelata; scientificNameAuthorship: (Schumach.) Stapf & C.E.Hubb ex Moss; **Location:** continent: Africa; country: Tanzania; stateProvince: Arusha; county: Ngorongoro; locality: Empakai Crater; verbatimLocality: Empakai Crater, top of South rim.; minimumElevationInMeters: 2900; decimalLatitude: -2.933333; decimalLongitude: 35.816667; **Event:** eventDate: 1973-02-26; **Record Level:** institutionCode: SWRC; collectionCode: Herbarium; ownerInstitutionCode: SWRC; basisOfRecord: PreservedSpecimen
- **Type status:** Other material. **Occurrence:** catalogNumber: DB0592; recordNumber: 676; recordedBy: Schmidt, W; **Taxon:** scientificName: *Setaria sphacelata* (Schumach.) Stapf & C.E.Hubb ex Moss; kingdom: Plantae; family: Poaceae; genus: Setaria; specificEpithet: sphacelata; scientificNameAuthorship: (Schumach.) Stapf & C.E.Hubb ex Moss; **Location:** continent: Africa; country: Tanzania; stateProvince: Mara; county: Serengeti; locality: Seronera river; verbatimLocality: Seronera river, the 3rd crossing from Seronera on the Banagi road.; minimumElevationInMeters: 1410; decimalLatitude: -2.45; decimalLongitude: 34.833333; **Event:** eventDate: 1961-04-03; **Record Level:** institutionCode: SWRC; collectionCode: Herbarium; ownerInstitutionCode: SWRC; basisOfRecord: PreservedSpecimen
- **Type status:** Other material. **Occurrence:** catalogNumber: DB0589; recordNumber: 278; recordedBy: Belsky, PJ; **Taxon:** scientificName: *Setaria sphacelata* (Schumach.) Stapf & C.E.Hubb ex Moss; kingdom: Plantae; family: Poaceae; genus: Setaria; specificEpithet: sphacelata; scientificNameAuthorship: (Schumach.) Stapf & C.E.Hubb ex Moss; **Location:** continent: Africa; country: Tanzania; stateProvince: Mara; county: Serengeti; locality: Lobo Lodge; verbatimLocality: 5 km north of Lobo Lodge; decimalLatitude: -1.933333; decimalLongitude: 35.166667; **Event:** eventDate: 1981-05-31; **Record Level:** institutionCode: SWRC; collectionCode: Herbarium; ownerInstitutionCode: SWRC; basisOfRecord: PreservedSpecimen
- **Type status:** Other material. **Occurrence:** catalogNumber: DB0588; recordNumber: 805; recordedBy: Schmidt, W; **Taxon:** scientificName: *Setaria sphacelata* (Schumach.) Stapf & C.E.Hubb ex Moss; kingdom: Plantae; family: Poaceae; genus: Setaria; specificEpithet: sphacelata; scientificNameAuthorship: (Schumach.) Stapf & C.E.Hubb ex Moss; **Location:** continent: Africa; country: Tanzania; stateProvince: Mara; county: Serengeti; locality: Bolgonja; verbatimLocality: Belongonja; decimalLatitude: -1.783333; decimalLongitude: 35.2; **Event:** eventDate: 1972-04-27; **Record Level:** institutionCode: SWRC; collectionCode: Herbarium; ownerInstitutionCode: SWRC; basisOfRecord: PreservedSpecimen
- **Type status:** Other material. **Occurrence:** catalogNumber: 685; recordNumber: s.n.; recordedBy: Mboya, E; **Taxon:** scientificName: *Setaria sphacelata* (Schumach.) Stapf & C.E.Hubb ex Moss; kingdom: Plantae; family: Poaceae; genus: Setaria; specificEpithet: sphacelata; scientificNameAuthorship: (Schumach.) Stapf & C.E.Hubb ex Moss; **Location:** continent: Africa; country: Tanzania; stateProvince: Mara; county: Serengeti; locality: Serengeti National Park; verbatimLocality: T1. Serengeti National Park. Ndabaka-Seronera Road.; minimumElevationInMeters: 1315; decimalLatitude: -2.27; decimalLongitude: 34.61; **Event:** eventDate: 2004-03-09; **Record Level:** institutionCode: MO; collectionCode: Herbarium; ownerInstitutionCode: MO; basisOfRecord: PreservedSpecimen
- **Type status:** Other material. **Occurrence:** catalogNumber: 686; recordNumber: s.n.; recordedBy: Mboya, E; **Taxon:** scientificName: *Setaria sphacelata* (Schumach.) Stapf & C.E.Hubb ex Moss; kingdom: Plantae; family: Poaceae; genus: Setaria; specificEpithet: sphacelata; scientificNameAuthorship: (Schumach.) Stapf & C.E.Hubb ex Moss; **Location:** continent: Africa; country: Tanzania; stateProvince: Mara; county: Serengeti; locality: Serengeti National Park; verbatimLocality: T1. Serengeti National Park. Ndabaka-Seronera Road.; minimumElevationInMeters: 1494; decimalLatitude: -2.43; decimalLongitude: 34.82; **Event:** eventDate: 2004-02-05; **Record Level:** institutionCode: MO; collectionCode: Herbarium; ownerInstitutionCode: MO; basisOfRecord: PreservedSpecimen
- **Type status:** Other material. **Occurrence:** catalogNumber: 687; recordNumber: 11; recordedBy: Metele, P; **Taxon:** scientificName: *Setaria sphacelata* (Schumach.) Stapf & C.E.Hubb ex Moss; kingdom: Plantae; family: Poaceae; genus: Setaria; specificEpithet: sphacelata; scientificNameAuthorship: (Schumach.) Stapf & C.E.Hubb ex Moss; **Location:** continent: Africa; country: Tanzania; stateProvince: Arusha; county: Ngorongoro; locality: Ngorongoro Crater; verbatimLocality: T2. Ngorongoro Crater, Misigiyo, 15.1 km from NCAA headquarters; minimumElevationInMeters: 2270; decimalLatitude: -3; decimalLongitude: 35; **Event:** eventDate: 1997-06-17; **Record Level:** institutionCode: MO; collectionCode: Herbarium; ownerInstitutionCode: MO; basisOfRecord: PreservedSpecimen
- **Type status:** Other material. **Occurrence:** catalogNumber: 1096; recordNumber: 11; recordedBy: Metele, P; **Taxon:** scientificName: *Setaria sphacelata* (Schumach.) Stapf & C.E.Hubb ex Moss; kingdom: Plantae; family: Poaceae; genus: Setaria; specificEpithet: sphacelata; scientificNameAuthorship: (Schumach.) Stapf & C.E.Hubb ex Moss; **Location:** continent: Africa; country: Tanzania; stateProvince: Arusha; county: Ngorongoro; locality: Ngorongoro Crater; verbatimLocality: T2. Ngorongoro Crater, Misigiyo, 15.1 km from NCAA headquarters; minimumElevationInMeters: 2270; decimalLatitude: -3; decimalLongitude: 35; **Event:** eventDate: 1997-06-17; **Record Level:** institutionCode: NHT; collectionCode: Herbarium; ownerInstitutionCode: NHT; basisOfRecord: PreservedSpecimen
- **Type status:** Other material. **Occurrence:** catalogNumber: 1128; recordNumber: 882; recordedBy: Mollel, NP; Rusch, GM; Mwakalebe, G; **Taxon:** scientificName: *Setaria sphacelata* (Schumach.) Stapf & C.E.Hubb ex Moss; kingdom: Plantae; family: Poaceae; genus: Setaria; specificEpithet: sphacelata; scientificNameAuthorship: (Schumach.) Stapf & C.E.Hubb ex Moss; **Location:** continent: Africa; country: Tanzania; stateProvince: Mara; county: Serengeti; locality: Robanda village; verbatimLocality: Near Robanda Hill, 36M, 689097, 9763522 UTM.; minimumElevationInMeters: 1416; decimalLatitude: -2.083333; decimalLongitude: 34.666667; **Event:** eventDate: 2003-01-29; **Record Level:** institutionCode: NHT; collectionCode: Herbarium; ownerInstitutionCode: NHT; basisOfRecord: PreservedSpecimen
- **Type status:** Other material. **Occurrence:** catalogNumber: 1129; recordNumber: 708; recordedBy: Mboya, EI; Shombe, H; **Taxon:** scientificName: *Setaria sphacelata* (Schumach.) Stapf & C.E.Hubb ex Moss; kingdom: Plantae; family: Poaceae; genus: Setaria; specificEpithet: sphacelata; scientificNameAuthorship: (Schumach.) Stapf & C.E.Hubb ex Moss; **Location:** continent: Africa; country: Tanzania; stateProvince: Mara; county: Serengeti; locality: Serengeti NP; verbatimLocality: Ndabaka- Seronera road. 0702390, 9731772 UTM; minimumElevationInMeters: 1494; decimalLatitude: -2.25; decimalLongitude: 34.333333; **Event:** eventDate: 2004-02-04; **Record Level:** institutionCode: NHT; collectionCode: Herbarium; ownerInstitutionCode: NHT; basisOfRecord: PreservedSpecimen
- **Type status:** Other material. **Occurrence:** catalogNumber: 1132; recordNumber: EN551; recordedBy: Njau, E; Leliyo, G; Andrew, P; Bamford, M; **Taxon:** scientificName: *Setaria sphacelata* (Schumach.) Stapf & C.E.Hubb ex Moss; kingdom: Plantae; family: Poaceae; genus: Setaria; specificEpithet: sphacelata; scientificNameAuthorship: (Schumach.) Stapf & C.E.Hubb ex Moss; **Location:** continent: Africa; country: Tanzania; stateProvince: Arusha; county: Ngorongoro; locality: Enduleni; verbatimLocality: Ngorongoro conservation area, Curusi river, west of Enduleni.; minimumElevationInMeters: 1700; decimalLatitude: -3.211111; decimalLongitude: 35.220556; **Event:** eventDate: 2004-06-25; **Record Level:** institutionCode: NHT; collectionCode: Herbarium; ownerInstitutionCode: NHT; basisOfRecord: PreservedSpecimen
- **Type status:** Other material. **Occurrence:** catalogNumber: K000984114; recordNumber: 9968; recordedBy: Greenway, PJ; **Taxon:** scientificName: Setaria sphacelata (Schumach.) Stapf & C.E.Hubb ex Moss var. sericea (Stapf) Clayton; kingdom: Plantae; family: Poaceae; genus: Setaria; specificEpithet: sphacelata; infraspecificEpithet: sericea; scientificNameAuthorship: (Stapf) Clayton; **Location:** continent: Africa; country: Tanzania; stateProvince: Mara; county: Serengeti; locality: Seronera river; minimumElevationInMeters: 1432; decimalLatitude: -2.45; decimalLongitude: 34.833333; **Event:** eventDate: 1961-04-03; **Record Level:** institutionCode: K; collectionCode: Herbarium; ownerInstitutionCode: K; basisOfRecord: PreservedSpecimen
- **Type status:** Other material. **Occurrence:** catalogNumber: K000984115; recordNumber: 10594; recordedBy: Greenway, PJ; Tanner, M; Rensbjurgh, HJ van; **Taxon:** scientificName: Setaria sphacelata (Schumach.) Stapf & C.E.Hubb ex Moss var. sericea (Stapf) Clayton; kingdom: Plantae; family: Poaceae; genus: Setaria; specificEpithet: sphacelata; infraspecificEpithet: sericea; scientificNameAuthorship: (Stapf) Clayton; **Location:** continent: Africa; country: Tanzania; stateProvince: Shinyanga; locality: Lake Magadi; verbatimLocality: E. side; minimumElevationInMeters: 1371; decimalLatitude: -2.633333; decimalLongitude: 34.9; **Event:** eventDate: 1962-04-11; **Record Level:** institutionCode: K; collectionCode: Herbarium; ownerInstitutionCode: K; basisOfRecord: PreservedSpecimen
- **Type status:** Other material. **Occurrence:** catalogNumber: K000984117; recordNumber: P5; recordedBy: Frame, GW; **Taxon:** scientificName: Setaria sphacelata (Schumach.) Stapf & C.E.Hubb ex Moss var. sericea (Stapf) Clayton; kingdom: Plantae; family: Poaceae; genus: Setaria; specificEpithet: sphacelata; infraspecificEpithet: sericea; scientificNameAuthorship: (Stapf) Clayton; **Location:** continent: Africa; country: Tanzania; stateProvince: Arusha; county: Ngorongoro; locality: Empakai Crater; verbatimLocality: top of the southern rim; minimumElevationInMeters: 2900; decimalLatitude: -2.933333; decimalLongitude: 35.816667; **Event:** eventDate: 1973-02-28; **Record Level:** institutionCode: K; collectionCode: Herbarium; ownerInstitutionCode: K; basisOfRecord: PreservedSpecimen
- **Type status:** Other material. **Occurrence:** catalogNumber: DB0591; recordNumber: 10594; recordedBy: Greenway, PJ; Tanner, M; Rensbjurgh, HJ van; **Taxon:** scientificName: Setaria sphacelata (Schumach.) Stapf & C.E.Hubb ex Moss var. sericea (Stapf) Clayton; kingdom: Plantae; family: Poaceae; genus: Setaria; specificEpithet: sphacelata; infraspecificEpithet: sericea; scientificNameAuthorship: (Stapf) Clayton; **Location:** continent: Africa; country: Tanzania; stateProvince: Shinyanga; locality: Lake Magadi; verbatimLocality: E. side; minimumElevationInMeters: 1371; decimalLatitude: -2.633333; decimalLongitude: 34.9; **Event:** eventDate: 1962-04-11; **Record Level:** institutionCode: SWRC; collectionCode: Herbarium; ownerInstitutionCode: SWRC; basisOfRecord: PreservedSpecimen
- **Type status:** Other material. **Occurrence:** catalogNumber: DB0590; recordNumber: 9968; recordedBy: Greenway, PJ; **Taxon:** scientificName: Setaria sphacelata (Schumach.) Stapf & C.E.Hubb ex Moss var. sericea (Stapf) Clayton; kingdom: Plantae; family: Poaceae; genus: Setaria; specificEpithet: sphacelata; infraspecificEpithet: sericea; scientificNameAuthorship: (Stapf) Clayton; **Location:** continent: Africa; country: Tanzania; stateProvince: Mara; county: Serengeti; locality: Seronera river; minimumElevationInMeters: 1432; decimalLatitude: -2.45; decimalLongitude: 34.833333; **Event:** eventDate: 1961-04-03; **Record Level:** institutionCode: SWRC; collectionCode: Herbarium; ownerInstitutionCode: SWRC; basisOfRecord: PreservedSpecimen
- **Type status:** Other material. **Occurrence:** catalogNumber: 1097; recordNumber: P5; recordedBy: Frame, GW; **Taxon:** scientificName: Setaria sphacelata (Schumach.) Stapf & C.E.Hubb ex Moss var. sericea (Stapf) Clayton; kingdom: Plantae; family: Poaceae; genus: Setaria; specificEpithet: sphacelata; infraspecificEpithet: sericea; scientificNameAuthorship: (Stapf) Clayton; **Location:** continent: Africa; country: Tanzania; stateProvince: Arusha; county: Ngorongoro; locality: Empakai Crater; verbatimLocality: top of the southern rim; minimumElevationInMeters: 2900; decimalLatitude: -2.933333; decimalLongitude: 35.816667; **Event:** eventDate: 1973-02-28; **Record Level:** institutionCode: NHT; collectionCode: Herbarium; ownerInstitutionCode: NHT; basisOfRecord: PreservedSpecimen
- **Type status:** Other material. **Occurrence:** catalogNumber: K000984113; recordNumber: 6290; recordedBy: Newbould, JB; **Taxon:** scientificName: Setaria sphacelata (Schumach.) Stapf & C.E.Hubb ex Moss var. torta (Stapf) Clayton; kingdom: Plantae; family: Poaceae; genus: Setaria; specificEpithet: sphacelata; infraspecificEpithet: torta; scientificNameAuthorship: (Stapf) Clayton; **Location:** continent: Africa; country: Tanzania; stateProvince: Shinyanga; locality: Ngamuriak; verbatimLocality: E. Serengeti; minimumElevationInMeters: 2042; decimalLatitude: -2.833333; decimalLongitude: 35; **Event:** eventDate: 1962-11-21; **Record Level:** institutionCode: K; collectionCode: Herbarium; ownerInstitutionCode: K; basisOfRecord: PreservedSpecimen

##### Distribution

Widespread

#### Setaria sulcata

Raddi

##### Materials

- **Type status:** Other material. **Occurrence:** catalogNumber: DB0629; recordNumber: 521; recordedBy: Schmidt, W; **Taxon:** scientificName: *Setaria sulcata* Raddi; kingdom: Plantae; family: Poaceae; genus: Setaria; specificEpithet: sulcata; scientificNameAuthorship: Raddi; **Location:** continent: Africa; country: Tanzania; stateProvince: Mara; county: Serengeti; locality: Bolgonja; verbatimLocality: Serengeti National Park; decimalLatitude: -1.783333; decimalLongitude: 35.2; **Event:** eventDate: 1970-04-13; **Record Level:** institutionCode: SWRC; collectionCode: Herbarium; ownerInstitutionCode: SWRC; basisOfRecord: PreservedSpecimen

##### Distribution

Native to Central & South America, introduced in Tanzania

#### Setaria verticillata

(L.) P.Beauv.

##### Materials

- **Type status:** Other material. **Occurrence:** catalogNumber: K000984108; recordNumber: 9980; recordedBy: Greenway, PJ; **Taxon:** scientificName: *Setaria verticillata* (L.) P.Beauv.; kingdom: Plantae; family: Poaceae; genus: Setaria; specificEpithet: verticillata; scientificNameAuthorship: (L.) P.Beauv.; **Location:** continent: Africa; country: Tanzania; stateProvince: Mara; county: Serengeti; locality: Seronera; verbatimLocality: Dam site; minimumElevationInMeters: 1448; decimalLatitude: -2.433333; decimalLongitude: 34.983333; **Event:** eventDate: 1961-04-05; **Record Level:** institutionCode: K; collectionCode: Herbarium; ownerInstitutionCode: K; basisOfRecord: PreservedSpecimen
- **Type status:** Other material. **Occurrence:** catalogNumber: K000984109; recordNumber: 5810; recordedBy: Newbould, JB; **Taxon:** scientificName: *Setaria verticillata* (L.) P.Beauv.; kingdom: Plantae; family: Poaceae; genus: Setaria; specificEpithet: verticillata; scientificNameAuthorship: (L.) P.Beauv.; **Location:** continent: Africa; country: Tanzania; stateProvince: Arusha; county: Ngorongoro; locality: Kakesio; verbatimLocality: Kakessio; minimumElevationInMeters: 1768; decimalLatitude: -3.316667; decimalLongitude: 35.033333; **Event:** eventDate: 1961-04-07; **Record Level:** institutionCode: K; collectionCode: Herbarium; ownerInstitutionCode: K; basisOfRecord: PreservedSpecimen
- **Type status:** Other material. **Occurrence:** catalogNumber: K000984110; recordNumber: 16; recordedBy: Msuya, EZM; **Taxon:** scientificName: *Setaria verticillata* (L.) P.Beauv.; kingdom: Plantae; family: Poaceae; genus: Setaria; specificEpithet: verticillata; scientificNameAuthorship: (L.) P.Beauv.; **Location:** continent: Africa; country: Tanzania; stateProvince: Arusha; county: Ngorongoro; locality: Endulen; minimumElevationInMeters: 1829; decimalLatitude: -3.183333; decimalLongitude: 35.15; **Event:** eventDate: 1961-06-07; **Record Level:** institutionCode: K; collectionCode: Herbarium; ownerInstitutionCode: K; basisOfRecord: PreservedSpecimen
- **Type status:** Other material. **Occurrence:** catalogNumber: DB0650; recordNumber: 136; recordedBy: Belsky, PJ; **Taxon:** scientificName: *Setaria verticillata* (L.) P.Beauv.; kingdom: Plantae; family: Poaceae; genus: Setaria; specificEpithet: verticillata; scientificNameAuthorship: (L.) P.Beauv.; **Location:** continent: Africa; country: Tanzania; stateProvince: Mara; county: Serengeti; locality: Serengeti Research Institute; verbatimLocality: Serengeti Research Institute; decimalLatitude: -2.433333; decimalLongitude: 34.85; **Event:** eventDate: 1981-05-11; **Record Level:** institutionCode: SWRC; collectionCode: Herbarium; ownerInstitutionCode: SWRC; basisOfRecord: PreservedSpecimen
- **Type status:** Other material. **Occurrence:** catalogNumber: 688; recordNumber: 694; recordedBy: Ellemann, L; **Taxon:** scientificName: *Setaria verticillata* (L.) P.Beauv.; kingdom: Plantae; family: Poaceae; genus: Setaria; specificEpithet: verticillata; scientificNameAuthorship: (L.) P.Beauv.; **Location:** continent: Africa; country: Tanzania; stateProvince: Arusha; county: Ngorongoro; locality: Endulen; verbatimLocality: Ngorongoro Conservation Area, Ndeyan 4 km North-west of Endulen Village.; minimumElevationInMeters: 1900; decimalLatitude: -3.2; decimalLongitude: 35.283; **Event:** eventDate: 1993-07-16; **Record Level:** institutionCode: AAU; collectionCode: Herbarium; ownerInstitutionCode: AAU; basisOfRecord: PreservedSpecimen
- **Type status:** Other material. **Occurrence:** catalogNumber: 689; recordNumber: 711; recordedBy: Ellemann, L; **Taxon:** scientificName: *Setaria verticillata* (L.) P.Beauv.; kingdom: Plantae; family: Poaceae; genus: Setaria; specificEpithet: verticillata; scientificNameAuthorship: (L.) P.Beauv.; **Location:** continent: Africa; country: Tanzania; stateProvince: Arusha; county: Ngorongoro; locality: Enja Shori; verbatimLocality: Ngorongoro Conservation Area, along Enja Shori,; minimumElevationInMeters: 1500; decimalLatitude: -3.033; decimalLongitude: 35.266; **Event:** eventDate: 1993-07-19; **Record Level:** institutionCode: AAU; collectionCode: Herbarium; ownerInstitutionCode: AAU; basisOfRecord: PreservedSpecimen
- **Type status:** Other material. **Occurrence:** catalogNumber: 690; recordNumber: 818; recordedBy: Ellemann, L; **Taxon:** scientificName: *Setaria verticillata* (L.) P.Beauv.; kingdom: Plantae; family: Poaceae; genus: Setaria; specificEpithet: verticillata; scientificNameAuthorship: (L.) P.Beauv.; **Location:** continent: Africa; country: Tanzania; stateProvince: Arusha; county: Ngorongoro; locality: Nasiporiong; verbatimLocality: Ngorongoro Conservation Area, Nasiporiong'.; minimumElevationInMeters: 1800; decimalLatitude: -3.2; decimalLongitude: 35.2; **Event:** eventDate: 1993-08-19; **Record Level:** institutionCode: AAU; collectionCode: Herbarium; ownerInstitutionCode: AAU; basisOfRecord: PreservedSpecimen
- **Type status:** Other material. **Occurrence:** catalogNumber: 691; recordNumber: s.n.; recordedBy: Mboya, E; **Taxon:** scientificName: *Setaria verticillata* (L.) P.Beauv.; kingdom: Plantae; family: Poaceae; genus: Setaria; specificEpithet: verticillata; scientificNameAuthorship: (L.) P.Beauv.; **Location:** continent: Africa; country: Tanzania; stateProvince: Mara; county: Serengeti; locality: Serengeti National Park; verbatimLocality: T1. Serengeti National Park. Ndabaka-Seronera Road.; minimumElevationInMeters: 1297; decimalLatitude: -2.27; decimalLongitude: 34.58; **Event:** eventDate: 2004-03-09; **Record Level:** institutionCode: MO; collectionCode: Herbarium; ownerInstitutionCode: MO; basisOfRecord: PreservedSpecimen
- **Type status:** Other material. **Occurrence:** catalogNumber: 692; recordNumber: s.n.; recordedBy: Mboya, E; **Taxon:** scientificName: *Setaria verticillata* (L.) P.Beauv.; kingdom: Plantae; family: Poaceae; genus: Setaria; specificEpithet: verticillata; scientificNameAuthorship: (L.) P.Beauv.; **Location:** continent: Africa; country: Tanzania; stateProvince: Mara; county: Serengeti; locality: Serengeti National Park; verbatimLocality: T1. Serengeti National Park. Ndabaka-Seronera Road.; minimumElevationInMeters: 1290; decimalLatitude: -2.27; decimalLongitude: 34.55; **Event:** eventDate: 2004-02-02; **Record Level:** institutionCode: MO; collectionCode: Herbarium; ownerInstitutionCode: MO; basisOfRecord: PreservedSpecimen
- **Type status:** Other material. **Occurrence:** catalogNumber: 1126; recordNumber: 1119; recordedBy: Mollel, NP; Mboya, EI; **Taxon:** scientificName: *Setaria verticillata* (L.) P.Beauv.; kingdom: Plantae; family: Poaceae; genus: Setaria; specificEpithet: verticillata; scientificNameAuthorship: (L.) P.Beauv.; **Location:** continent: Africa; country: Tanzania; stateProvince: Arusha; county: Ngorongoro; locality: Laetoli; verbatimLocality: Esere-Laetoli road, at the junction to the Laetoli footprints.; minimumElevationInMeters: 1616; decimalLatitude: -3.291389; decimalLongitude: 35.173611; **Event:** eventDate: 2008-04-07; **Record Level:** institutionCode: NHT; collectionCode: Herbarium; ownerInstitutionCode: NHT; basisOfRecord: PreservedSpecimen
- **Type status:** Other material. **Occurrence:** catalogNumber: 1127; recordNumber: 1119; recordedBy: Mollel, NP; Mboya, EI; **Taxon:** scientificName: *Setaria verticillata* (L.) P.Beauv.; kingdom: Plantae; family: Poaceae; genus: Setaria; specificEpithet: verticillata; scientificNameAuthorship: (L.) P.Beauv.; **Location:** continent: Africa; country: Tanzania; stateProvince: Arusha; county: Ngorongoro; locality: Laetoli; verbatimLocality: Esere-Laetoli road, at the junction to the Laetoli footprints.; minimumElevationInMeters: 1616; decimalLatitude: -3.291389; decimalLongitude: 35.173611; **Event:** eventDate: 2008-04-07; **Record Level:** institutionCode: NCAA; collectionCode: Herbarium; ownerInstitutionCode: NCAA; basisOfRecord: PreservedSpecimen
- **Type status:** Other material. **Occurrence:** catalogNumber: DB0651; recordNumber: 9980; recordedBy: Greenway, PJ; **Taxon:** scientificName: *Setaria verticillata* (L.) P.Beauv.; kingdom: Plantae; family: Poaceae; genus: Setaria; specificEpithet: verticillata; scientificNameAuthorship: (L.) P.Beauv.; **Location:** continent: Africa; country: Tanzania; stateProvince: Mara; county: Serengeti; locality: Seronera; verbatimLocality: Dam site; minimumElevationInMeters: 1448; decimalLatitude: -2.433333; decimalLongitude: 34.983333; **Event:** eventDate: 1961-04-05; **Record Level:** institutionCode: SWRC; collectionCode: Herbarium; ownerInstitutionCode: SWRC; basisOfRecord: PreservedSpecimen

##### Distribution

Widespread

#### Snowdenia petitiana

(A.Rich.) C.E.Hubb

##### Materials

- **Type status:** Other material. **Occurrence:** catalogNumber: DB0655; recordNumber: 94; recordedBy: Frame, GW; **Taxon:** scientificName: *Snowdenia petitiana* (A.Rich.) C.E.Hubb; kingdom: Plantae; family: Poaceae; genus: Snowdenia; specificEpithet: petitiana; scientificNameAuthorship: (A.Rich.) C.E.Hubb; **Location:** continent: Africa; country: Tanzania; stateProvince: Arusha; county: Ngorongoro; locality: Empakai Crater; verbatimLocality: Empakai crater, top of east rim; minimumElevationInMeters: 2600; decimalLatitude: -2.933333; decimalLongitude: 35.816667; **Event:** eventDate: 1973-03-14; **Record Level:** institutionCode: SWRC; collectionCode: Herbarium; ownerInstitutionCode: SWRC; basisOfRecord: PreservedSpecimen
- **Type status:** Other material. **Occurrence:** catalogNumber: DB0654; recordNumber: 169; recordedBy: Frame, GW; **Taxon:** scientificName: *Snowdenia petitiana* (A.Rich.) C.E.Hubb; kingdom: Plantae; family: Poaceae; genus: Snowdenia; specificEpithet: petitiana; scientificNameAuthorship: (A.Rich.) C.E.Hubb; **Location:** continent: Africa; country: Tanzania; stateProvince: Arusha; county: Ngorongoro; locality: Empakai Crater; verbatimLocality: Empakaai crater; inside east rim.; minimumElevationInMeters: 2300; decimalLatitude: -2.933333; decimalLongitude: 35.816667; **Event:** eventDate: 1973-05-30; **Record Level:** institutionCode: SWRC; collectionCode: Herbarium; ownerInstitutionCode: SWRC; basisOfRecord: PreservedSpecimen
- **Type status:** Other material. **Occurrence:** catalogNumber: 1101; recordNumber: 94; recordedBy: Frame, GW; **Taxon:** scientificName: *Snowdenia petitiana* (A.Rich.) C.E.Hubb; kingdom: Plantae; family: Poaceae; genus: Snowdenia; specificEpithet: petitiana; scientificNameAuthorship: (A.Rich.) C.E.Hubb; **Location:** continent: Africa; country: Tanzania; stateProvince: Arusha; county: Ngorongoro; locality: Empakai Crater; verbatimLocality: Empakai crater, top of east rim; minimumElevationInMeters: 2600; decimalLatitude: -2.933333; decimalLongitude: 35.816667; **Event:** eventDate: 1973-03-14; **Record Level:** institutionCode: NHT; collectionCode: Herbarium; ownerInstitutionCode: NHT; basisOfRecord: PreservedSpecimen

##### Distribution

Eastern Africa & Yemen

#### Sorghum arundinaceum

(Desv.) Stapf

Sorghum verticilliflorum (Steud.) Stapf

##### Materials

- **Type status:** Other material. **Occurrence:** catalogNumber: 693; recordNumber: s.n.; recordedBy: Mboya, E; **Taxon:** scientificName: *Sorghum arundinaceum* (Desv.) Stapf; kingdom: Plantae; family: Poaceae; genus: Sorghum; specificEpithet: arundinaceum; scientificNameAuthorship: (Desv.) Stapf; **Location:** continent: Africa; country: Tanzania; stateProvince: Mara; county: Serengeti; locality: Serengeti National Park; verbatimLocality: T1. Serengeti National Park. Ndabaka-Seronera Road.; minimumElevationInMeters: 1303; decimalLatitude: -2.27; decimalLongitude: 34.61; **Event:** eventDate: 2004-02-06; **Record Level:** institutionCode: MO; collectionCode: Herbarium; ownerInstitutionCode: MO; basisOfRecord: PreservedSpecimen
- **Type status:** Other material. **Occurrence:** catalogNumber: K000731349; recordNumber: 10321; recordedBy: Greenway, PJ; **Taxon:** scientificName: *Sorghum verticilliflorum* (Steud.) Stapf; kingdom: Plantae; family: Poaceae; genus: Sorghum; specificEpithet: verticilliflorum; scientificNameAuthorship: (Steud.) Stapf; **Location:** continent: Africa; country: Tanzania; stateProvince: Mara; county: Serengeti; locality: Seronera river; verbatimLocality: Seronera River near Seronera-Serengeti.; minimumElevationInMeters: 1432; decimalLatitude: -2.45; decimalLongitude: 34.833333; **Event:** eventDate: 1961-05-20; **Record Level:** institutionCode: K; collectionCode: Herbarium; ownerInstitutionCode: K; basisOfRecord: PreservedSpecimen

##### Distribution

Widespread

#### Sorghum versicolor

Andersson

##### Materials

- **Type status:** Other material. **Occurrence:** catalogNumber: K000984063; recordNumber: 10075; recordedBy: Greenway, PJ; Tanner, M; **Taxon:** scientificName: *Sorghum versicolor* Andersson; kingdom: Plantae; family: Poaceae; genus: Sorghum; specificEpithet: versicolor; scientificNameAuthorship: Andersson; **Location:** continent: Africa; country: Tanzania; stateProvince: Shinyanga; locality: Ndoha Plains; verbatimLocality: Eastwards of Ndoha plains; minimumElevationInMeters: 1219; decimalLatitude: -2.5; decimalLongitude: 34.416667; **Event:** eventDate: 1961-04-20; **Record Level:** institutionCode: K; collectionCode: Herbarium; ownerInstitutionCode: K; basisOfRecord: PreservedSpecimen
- **Type status:** Other material. **Occurrence:** catalogNumber: K000984064; recordNumber: 425; recordedBy: Paulo, S; **Taxon:** scientificName: *Sorghum versicolor* Andersson; kingdom: Plantae; family: Poaceae; genus: Sorghum; specificEpithet: versicolor; scientificNameAuthorship: Andersson; **Location:** continent: Africa; country: Tanzania; stateProvince: Shinyanga; locality: Subiti Hill; verbatimLocality: N.E. of Subiti Hill; decimalLatitude: -3.433333; decimalLongitude: 34.95; **Event:** eventDate: 1958-05-03; **Record Level:** institutionCode: K; collectionCode: Herbarium; ownerInstitutionCode: K; basisOfRecord: PreservedSpecimen
- **Type status:** Other material. **Occurrence:** catalogNumber: K000984065; recordNumber: 10368; recordedBy: Greenway, PJ; Tanner, M; **Taxon:** scientificName: *Sorghum versicolor* Andersson; kingdom: Plantae; family: Poaceae; genus: Sorghum; specificEpithet: versicolor; scientificNameAuthorship: Andersson; **Location:** continent: Africa; country: Tanzania; stateProvince: Shinyanga; locality: Mbono river; minimumElevationInMeters: 1493; decimalLatitude: -3.116667; decimalLongitude: 34.833333; **Event:** eventDate: 1961-06-07; **Record Level:** institutionCode: K; collectionCode: Herbarium; ownerInstitutionCode: K; basisOfRecord: PreservedSpecimen
- **Type status:** Other material. **Occurrence:** catalogNumber: DB0661; recordNumber: 10368; recordedBy: Greenway, PJ; Tanner, M; **Taxon:** scientificName: *Sorghum versicolor* Andersson; kingdom: Plantae; family: Poaceae; genus: Sorghum; specificEpithet: versicolor; scientificNameAuthorship: Andersson; **Location:** continent: Africa; country: Tanzania; stateProvince: Shinyanga; locality: Mbono river; minimumElevationInMeters: 1493; decimalLatitude: -3.116667; decimalLongitude: 34.833333; **Event:** eventDate: 1961-06-07; **Record Level:** institutionCode: SWRC; collectionCode: Herbarium; ownerInstitutionCode: SWRC; basisOfRecord: PreservedSpecimen
- **Type status:** Other material. **Occurrence:** catalogNumber: DB0660; recordNumber: 10075; recordedBy: Greenway, PJ; Tanner, M; **Taxon:** scientificName: *Sorghum versicolor* Andersson; kingdom: Plantae; family: Poaceae; genus: Sorghum; specificEpithet: versicolor; scientificNameAuthorship: Andersson; **Location:** continent: Africa; country: Tanzania; stateProvince: Shinyanga; locality: Ndoha Plains; verbatimLocality: Eastwards of Ndoha plains; minimumElevationInMeters: 1219; decimalLatitude: -2.5; decimalLongitude: 34.416667; **Event:** eventDate: 1961-04-20; **Record Level:** institutionCode: SWRC; collectionCode: Herbarium; ownerInstitutionCode: SWRC; basisOfRecord: PreservedSpecimen

##### Distribution

Tropical Africa & Arabia

#### Sporobolus sp.

##### Materials

- **Type status:** Other material. **Occurrence:** catalogNumber: 716; recordNumber: 805; recordedBy: Ellemann, L; **Taxon:** scientificName: Sporobolus; kingdom: Plantae; family: Poaceae; genus: Sporobolus; **Location:** continent: Africa; country: Tanzania; stateProvince: Arusha; county: Ngorongoro; locality: Olduvai Gorge; verbatimLocality: Ngorongoro Conservation Area, Olduvai Gorge; minimumElevationInMeters: 1400; decimalLatitude: -2.983; decimalLongitude: 35.35; **Event:** eventDate: 1993-08-18; **Record Level:** institutionCode: AAU; collectionCode: Herbarium; ownerInstitutionCode: AAU; basisOfRecord: PreservedSpecimen
- **Type status:** Other material. **Occurrence:** catalogNumber: 718; recordNumber: s.n.; recordedBy: Phillipson, PB; **Taxon:** scientificName: Sporobolus; kingdom: Plantae; family: Poaceae; genus: Sporobolus; **Location:** continent: Africa; country: Tanzania; stateProvince: Arusha; county: Ngorongoro; locality: Lake Magadi; verbatimLocality: Ngorongoro Conservation area Olduvai Gorge.; minimumElevationInMeters: 2250; decimalLatitude: -2.99; decimalLongitude: 35.35; **Event:** eventDate: 1905-06-19; **Record Level:** institutionCode: MO; collectionCode: Herbarium; ownerInstitutionCode: MO; basisOfRecord: PreservedSpecimen

#### Sporobolus africanus

(Poir.) Robyns & Tournay

Sporobolus capensis (P.Beauv.) Kunth

##### Materials

- **Type status:** Other material. **Occurrence:** catalogNumber: K000984168; recordNumber: 12597; recordedBy: Greenway, PJ; Kanuri; **Taxon:** scientificName: *Sporobolus africanus* (Poir.) Robyns & Tournay; kingdom: Plantae; family: Poaceae; genus: Sporobolus; specificEpithet: africanus; scientificNameAuthorship: (Poir.) Robyns & Tournay; **Location:** continent: Africa; country: Tanzania; stateProvince: Arusha; county: Ngorongoro; locality: Ngoitoktok springs; verbatimLocality: E. side of Ngorongoro Crater floor; minimumElevationInMeters: 1707; decimalLatitude: -3.2; decimalLongitude: 35.616667; **Event:** eventDate: 1966-07-21; **Record Level:** institutionCode: K; collectionCode: Herbarium; ownerInstitutionCode: K; basisOfRecord: PreservedSpecimen
- **Type status:** Other material. **Occurrence:** catalogNumber: K000984169; recordNumber: 210; recordedBy: Braun, HMH; **Taxon:** scientificName: *Sporobolus africanus* (Poir.) Robyns & Tournay; kingdom: Plantae; family: Poaceae; genus: Sporobolus; specificEpithet: africanus; scientificNameAuthorship: (Poir.) Robyns & Tournay; **Location:** continent: Africa; country: Tanzania; stateProvince: Mara; county: Serengeti; locality: Togoro Plains; minimumElevationInMeters: 1550; decimalLatitude: -2.166667; decimalLongitude: 34.916667; **Event:** eventDate: 1967-05-05; **Record Level:** institutionCode: K; collectionCode: Herbarium; ownerInstitutionCode: K; basisOfRecord: PreservedSpecimen
- **Type status:** Other material. **Occurrence:** catalogNumber: K000495994; recordNumber: 3367; recordedBy: Greenway, PJ; **Taxon:** scientificName: *Sporobolus africanus* (Poir.) Robyns & Tournay; kingdom: Plantae; family: Poaceae; genus: Sporobolus; specificEpithet: africanus; scientificNameAuthorship: (Poir.) Robyns & Tournay; **Location:** continent: Africa; country: Tanzania; stateProvince: Arusha; county: Ngorongoro; locality: Ngorongoro Crater; verbatimLocality: SE slope of crater.; minimumElevationInMeters: 2439; decimalLatitude: -3.166667; decimalLongitude: 35.583333; **Event:** eventDate: 1933-02-22; **Record Level:** institutionCode: K; collectionCode: Herbarium; ownerInstitutionCode: K; basisOfRecord: PreservedSpecimen
- **Type status:** Other material. **Occurrence:** catalogNumber: K000495993; recordNumber: 974; recordedBy: Pole Evans, IB; Erens, J; **Taxon:** scientificName: *Sporobolus africanus* (Poir.) Robyns & Tournay; kingdom: Plantae; family: Poaceae; genus: Sporobolus; specificEpithet: africanus; scientificNameAuthorship: (Poir.) Robyns & Tournay; **Location:** continent: Africa; country: Tanzania; stateProvince: Arusha; county: Ngorongoro; locality: Ngorongoro Crater; verbatimLocality: On the top ridge of crater.; decimalLatitude: -3.166667; decimalLongitude: 35.583333; **Event:** eventDate: 1938-06-24; **Record Level:** institutionCode: K; collectionCode: Herbarium; ownerInstitutionCode: K; basisOfRecord: PreservedSpecimen
- **Type status:** Other material. **Occurrence:** catalogNumber: K000495992; recordNumber: 5694; recordedBy: Newbould, JB; **Taxon:** scientificName: *Sporobolus africanus* (Poir.) Robyns & Tournay; kingdom: Plantae; family: Poaceae; genus: Sporobolus; specificEpithet: africanus; scientificNameAuthorship: (Poir.) Robyns & Tournay; **Location:** continent: Africa; country: Tanzania; stateProvince: Arusha; county: Ngorongoro; locality: Ngorongoro Crater; verbatimLocality: crater rim, forest near crater road.; decimalLatitude: -3.166667; decimalLongitude: 35.583333; **Event:** eventDate: 1961-03-04; **Record Level:** institutionCode: K; collectionCode: Herbarium; ownerInstitutionCode: K; basisOfRecord: PreservedSpecimen

##### Distribution

Widespread

#### Sporobolus agrostoides

Chiov.

##### Materials

- **Type status:** Other material. **Occurrence:** catalogNumber: K000984158; recordNumber: 115; recordedBy: Braun, HMH; **Taxon:** scientificName: *Sporobolus agrostoides* Chiov.; kingdom: Plantae; family: Poaceae; genus: Sporobolus; specificEpithet: agrostoides; scientificNameAuthorship: Chiov.; **Location:** continent: Africa; country: Tanzania; stateProvince: Mara; county: Serengeti; locality: Mgungu river; verbatimLocality: 2 km upstream from Banagi; minimumElevationInMeters: 1350; decimalLatitude: -2.5; decimalLongitude: 35.333333; **Event:** eventDate: 1957-01-24; **Record Level:** institutionCode: K; collectionCode: Herbarium; ownerInstitutionCode: K; basisOfRecord: PreservedSpecimen

##### Distribution

Tropical Africa

#### Sporobolus confinis

(Steud.) Chiov.

Sporobolus affinis A.Rich. | Sporobolus phyllotrichus Hochst.

##### Materials

- **Type status:** Other material. **Occurrence:** catalogNumber: K000984179; recordNumber: 11844; recordedBy: Greenway, PJ; Kanuri; **Taxon:** scientificName: *Sporobolus affinis* A.Rich.; kingdom: Plantae; family: Poaceae; genus: Sporobolus; specificEpithet: affinis; scientificNameAuthorship: A.Rich.; **Location:** continent: Africa; country: Tanzania; stateProvince: Arusha; county: Ngorongoro; locality: Ngorongoro Crater; verbatimLocality: floor, N.E. side; minimumElevationInMeters: 1524; decimalLatitude: -3.166667; decimalLongitude: 35.583333; **Event:** eventDate: 1965-06-11; **Record Level:** institutionCode: K; collectionCode: Herbarium; ownerInstitutionCode: K; basisOfRecord: PreservedSpecimen
- **Type status:** Other material. **Occurrence:** catalogNumber: K000495985; recordNumber: 1646; recordedBy: Heady, AF; **Taxon:** scientificName: *Sporobolus affinis* A.Rich.; kingdom: Plantae; family: Poaceae; genus: Sporobolus; specificEpithet: affinis; scientificNameAuthorship: A.Rich.; **Location:** continent: Africa; country: Tanzania; stateProvince: Arusha; county: Ngorongoro; locality: Ngorongoro Crater; decimalLatitude: -3.166667; decimalLongitude: 35.583333; **Event:** eventDate: 1959-02-17; **Record Level:** institutionCode: K; collectionCode: Herbarium; ownerInstitutionCode: K; basisOfRecord: PreservedSpecimen
- **Type status:** Other material. **Occurrence:** catalogNumber: K000984180; recordNumber: 167; recordedBy: Braun, HMH; **Taxon:** scientificName: *Sporobolus confinis* (Steud.) Chiov.; kingdom: Plantae; family: Poaceae; genus: Sporobolus; specificEpithet: confinis; scientificNameAuthorship: (Steud.) Chiov.; **Location:** continent: Africa; country: Tanzania; stateProvince: Arusha; county: Ngorongoro; locality: Ngorongoro Crater; verbatimLocality: S. Northern Tanzania. Hill in Northern Eastern part of crater floor; minimumElevationInMeters: 1900; decimalLatitude: -3.25; decimalLongitude: 35.5; **Event:** eventDate: 1967-11-26; **Record Level:** institutionCode: K; collectionCode: Herbarium; ownerInstitutionCode: K; basisOfRecord: PreservedSpecimen
- **Type status:** Other material. **Occurrence:** catalogNumber: 694; recordNumber: 24303; recordedBy: Peterson, PM; Soreng, RJ; Romaschenko, K; Mbago, F; **Taxon:** scientificName: *Sporobolus confinis* (Steud.) Chiov.; kingdom: Plantae; family: Poaceae; genus: Sporobolus; specificEpithet: confinis; scientificNameAuthorship: (Steud.) Chiov.; **Location:** continent: Africa; country: Tanzania; stateProvince: Arusha; county: Ngorongoro; locality: Ngorongoro Crater; verbatimLocality: Ngorongoro Conservation Area, rim of Ngorongoro Crater (descent gate).; minimumElevationInMeters: 2168; decimalLatitude: -3.15462; decimalLongitude: 35.47717; **Event:** eventDate: 2012-06-19; **Record Level:** institutionCode: US; collectionCode: Herbarium; ownerInstitutionCode: US; basisOfRecord: PreservedSpecimen
- **Type status:** Other material. **Occurrence:** catalogNumber: K000495984; recordNumber: 160; recordedBy: Braun, HMH; **Taxon:** scientificName: *Sporobolus confinis* (Steud.) Chiov.; kingdom: Plantae; family: Poaceae; genus: Sporobolus; specificEpithet: confinis; scientificNameAuthorship: (Steud.) Chiov.; **Location:** continent: Africa; country: Tanzania; stateProvince: Arusha; county: Ngorongoro; locality: Ngorongoro Crater; verbatimLocality: SE part of crater floor.; minimumElevationInMeters: 1750; decimalLatitude: -3.25; decimalLongitude: 35.5; **Event:** eventDate: 1967-02-25; **Record Level:** institutionCode: K; collectionCode: Herbarium; ownerInstitutionCode: K; basisOfRecord: PreservedSpecimen

##### Distribution

Eastern Africa & Yemen

#### Sporobolus consimilis

Fresen.

##### Materials

- **Type status:** Other material. **Occurrence:** catalogNumber: K000984174; recordNumber: 10045; recordedBy: Greenway, PJ; Tanner, M; **Taxon:** scientificName: *Sporobolus consimilis* Fresen.; kingdom: Plantae; family: Poaceae; genus: Sporobolus; specificEpithet: consimilis; scientificNameAuthorship: Fresen.; **Location:** continent: Africa; country: Tanzania; stateProvince: Shinyanga; locality: Lake Magadi; verbatimLocality: West side of Lake Magadi; minimumElevationInMeters: 1432; decimalLatitude: -2.633333; decimalLongitude: 34.9; **Event:** eventDate: 1961-04-13; **Record Level:** institutionCode: K; collectionCode: Herbarium; ownerInstitutionCode: K; basisOfRecord: PreservedSpecimen
- **Type status:** Other material. **Occurrence:** catalogNumber: K000984175; recordNumber: 9154; recordedBy: Greenway, PJ; **Taxon:** scientificName: *Sporobolus consimilis* Fresen.; kingdom: Plantae; family: Poaceae; genus: Sporobolus; specificEpithet: consimilis; scientificNameAuthorship: Fresen.; **Location:** continent: Africa; country: Tanzania; stateProvince: Shinyanga; locality: Moru Kopjes; verbatimLocality: Moru Main Water Holes; decimalLatitude: -2.75; decimalLongitude: 34.75; **Event:** eventDate: 1956-12-10; **Record Level:** institutionCode: K; collectionCode: Herbarium; ownerInstitutionCode: K; basisOfRecord: PreservedSpecimen
- **Type status:** Other material. **Occurrence:** catalogNumber: K000984176; recordNumber: 6173; recordedBy: Newbould, JB; **Taxon:** scientificName: *Sporobolus consimilis* Fresen.; kingdom: Plantae; family: Poaceae; genus: Sporobolus; specificEpithet: consimilis; scientificNameAuthorship: Fresen.; **Location:** continent: Africa; country: Tanzania; stateProvince: Arusha; county: Ngorongoro; locality: Ngorongoro Crater; minimumElevationInMeters: 1676; decimalLatitude: -3.166667; decimalLongitude: 35.583333; **Event:** eventDate: 1962-07-14; **Record Level:** institutionCode: K; collectionCode: Herbarium; ownerInstitutionCode: K; basisOfRecord: PreservedSpecimen
- **Type status:** Other material. **Occurrence:** catalogNumber: K000984177; recordNumber: 10352; recordedBy: Greenway, PJ; **Taxon:** scientificName: *Sporobolus consimilis* Fresen.; kingdom: Plantae; family: Poaceae; genus: Sporobolus; specificEpithet: consimilis; scientificNameAuthorship: Fresen.; **Location:** continent: Africa; country: Tanzania; stateProvince: Mara; county: Serengeti; locality: Seronera river; verbatimLocality: mile 3.7; minimumElevationInMeters: 1493; decimalLatitude: -2.45; decimalLongitude: 34.833333; **Event:** eventDate: 1961-06-03; **Record Level:** institutionCode: K; collectionCode: Herbarium; ownerInstitutionCode: K; basisOfRecord: PreservedSpecimen
- **Type status:** Other material. **Occurrence:** catalogNumber: K000984178; recordNumber: 19301; recordedBy: Raynal, J; **Taxon:** scientificName: *Sporobolus consimilis* Fresen.; kingdom: Plantae; family: Poaceae; genus: Sporobolus; specificEpithet: consimilis; scientificNameAuthorship: Fresen.; **Location:** continent: Africa; country: Tanzania; stateProvince: Arusha; county: Ngorongoro; locality: Olduvai; verbatimLocality: Ngorongoro Conservation Area; minimumElevationInMeters: 1500; decimalLatitude: -2.95; decimalLongitude: 35.166667; **Event:** eventDate: 1977-09-26; **Record Level:** institutionCode: K; collectionCode: Herbarium; ownerInstitutionCode: K; basisOfRecord: PreservedSpecimen
- **Type status:** Other material. **Occurrence:** catalogNumber: 695; recordNumber: 544; recordedBy: Ellemann, L; **Taxon:** scientificName: *Sporobolus consimilis* Fresen.; kingdom: Plantae; family: Poaceae; genus: Sporobolus; specificEpithet: consimilis; scientificNameAuthorship: Fresen.; **Location:** continent: Africa; country: Tanzania; stateProvince: Arusha; county: Ngorongoro; locality: Ololgumi; verbatimLocality: Ngorongoro Conservation Area. Collected along dry riverine, ca. 5 km South of Kakesio Village. Ololgumi.; minimumElevationInMeters: 1800; decimalLatitude: -3.066; decimalLongitude: 35.05; **Event:** eventDate: 1993-05-13; **Record Level:** institutionCode: AAU; collectionCode: Herbarium; ownerInstitutionCode: AAU; basisOfRecord: PreservedSpecimen
- **Type status:** Other material. **Occurrence:** catalogNumber: 696; recordNumber: 804; recordedBy: Ellemann, L; **Taxon:** scientificName: *Sporobolus consimilis* Fresen.; kingdom: Plantae; family: Poaceae; genus: Sporobolus; specificEpithet: consimilis; scientificNameAuthorship: Fresen.; **Location:** continent: Africa; country: Tanzania; stateProvince: Arusha; county: Ngorongoro; locality: Olduvai Gorge; verbatimLocality: Ngorongoro Conservation Area, Olduvai Gorge; minimumElevationInMeters: 1400; decimalLatitude: -2.983; decimalLongitude: 35.35; **Event:** eventDate: 1993-08-18; **Record Level:** institutionCode: AAU; collectionCode: Herbarium; ownerInstitutionCode: AAU; basisOfRecord: PreservedSpecimen
- **Type status:** Other material. **Occurrence:** catalogNumber: 697; recordNumber: 1061; recordedBy: Ellemann, L; **Taxon:** scientificName: *Sporobolus consimilis* Fresen.; kingdom: Plantae; family: Poaceae; genus: Sporobolus; specificEpithet: consimilis; scientificNameAuthorship: Fresen.; **Location:** continent: Africa; country: Tanzania; stateProvince: Arusha; county: Ngorongoro; locality: Olbili; verbatimLocality: Ngorongoro Conservation Area, Olbili (12 km from Endulen).; minimumElevationInMeters: 1400; decimalLatitude: -3.283; decimalLongitude: 35.166; **Event:** eventDate: 1994-02-17; **Record Level:** institutionCode: AAU; collectionCode: Herbarium; ownerInstitutionCode: AAU; basisOfRecord: PreservedSpecimen

##### Distribution

Tropical Africa & Arabia

#### Sporobolus cordofanus

(Hochst. ex Steud.) Hérincq ex Coss.

Sporobolus humifusus (Kunth) Kunth var. cordofanus (Hochst. ex Steud.) R.L.Massey

##### Materials

- **Type status:** Other material. **Occurrence:** catalogNumber: K000984192; recordNumber: 9985; recordedBy: Greenway, PJ; **Taxon:** scientificName: *Sporobolus cordofanus* (Hochst. ex Steud.) Hérincq ex Coss.; kingdom: Plantae; family: Poaceae; genus: Sporobolus; specificEpithet: cordofanus; scientificNameAuthorship: (Hochst. ex Steud.) Hérincq ex Coss.; **Location:** continent: Africa; country: Tanzania; stateProvince: Mara; county: Serengeti; locality: Seronera; verbatimLocality: dam site; minimumElevationInMeters: 1432; decimalLatitude: -2.45; decimalLongitude: 34.833333; **Event:** eventDate: 1961-04-05; **Record Level:** institutionCode: K; collectionCode: Herbarium; ownerInstitutionCode: K; basisOfRecord: PreservedSpecimen
- **Type status:** Other material. **Occurrence:** catalogNumber: K000984193; recordNumber: 6405; recordedBy: Newbould, JB; **Taxon:** scientificName: *Sporobolus cordofanus* (Hochst. ex Steud.) Hérincq ex Coss.; kingdom: Plantae; family: Poaceae; genus: Sporobolus; specificEpithet: cordofanus; scientificNameAuthorship: (Hochst. ex Steud.) Hérincq ex Coss.; **Location:** continent: Africa; country: Tanzania; stateProvince: Arusha; county: Ngorongoro; locality: Olkarien; minimumElevationInMeters: 1371; decimalLatitude: -2.6; decimalLongitude: 35.366667; **Event:** eventDate: 1962-12-20; **Record Level:** institutionCode: K; collectionCode: Herbarium; ownerInstitutionCode: K; basisOfRecord: PreservedSpecimen
- **Type status:** Other material. **Occurrence:** catalogNumber: K000984194; recordNumber: 223; recordedBy: Braun, HMH; **Taxon:** scientificName: *Sporobolus cordofanus* (Hochst. ex Steud.) Hérincq ex Coss.; kingdom: Plantae; family: Poaceae; genus: Sporobolus; specificEpithet: cordofanus; scientificNameAuthorship: (Hochst. ex Steud.) Hérincq ex Coss.; **Location:** continent: Africa; country: Tanzania; stateProvince: Arusha; county: Ngorongoro; locality: Gol Kopjes; verbatimLocality: Between Gol Kopjes and depressions in north eastern direction; minimumElevationInMeters: 1500; decimalLatitude: -2.7; decimalLongitude: 35.433333; **Event:** eventDate: 1967-05-18; **Record Level:** institutionCode: K; collectionCode: Herbarium; ownerInstitutionCode: K; basisOfRecord: PreservedSpecimen
- **Type status:** Other material. **Occurrence:** catalogNumber: K000984195; recordNumber: 10329; recordedBy: Greenway, PJ; **Taxon:** scientificName: *Sporobolus cordofanus* (Hochst. ex Steud.) Hérincq ex Coss.; kingdom: Plantae; family: Poaceae; genus: Sporobolus; specificEpithet: cordofanus; scientificNameAuthorship: (Hochst. ex Steud.) Hérincq ex Coss.; **Location:** continent: Africa; country: Tanzania; stateProvince: Mara; county: Serengeti; locality: Grumechem; verbatimLocality: E. boundary from Grumechem entrance to Naabi entrance, mile 23; minimumElevationInMeters: 1707; decimalLatitude: -2.3; decimalLongitude: 35.25; **Event:** eventDate: 1961-05-25; **Record Level:** institutionCode: K; collectionCode: Herbarium; ownerInstitutionCode: K; basisOfRecord: PreservedSpecimen
- **Type status:** Other material. **Occurrence:** catalogNumber: 698; recordNumber: 24313; recordedBy: Peterson, PM; Soreng, RJ; Romaschenko, K; Mbago, F; **Taxon:** scientificName: *Sporobolus cordofanus* (Hochst. ex Steud.) Hérincq ex Coss.; kingdom: Plantae; family: Poaceae; genus: Sporobolus; specificEpithet: cordofanus; scientificNameAuthorship: (Hochst. ex Steud.) Hérincq ex Coss.; **Location:** continent: Africa; country: Tanzania; stateProvince: Arusha; county: Ngorongoro; locality: Lake Magadi; verbatimLocality: Ngorongoro Conservation Area, W side of Lake Magadi.; minimumElevationInMeters: 1739; decimalLatitude: -3.17713; decimalLongitude: 35.51548; **Event:** eventDate: 2012-06-19; **Record Level:** institutionCode: US; collectionCode: Herbarium; ownerInstitutionCode: US; basisOfRecord: PreservedSpecimen
- **Type status:** Other material. **Occurrence:** catalogNumber: K000495975; recordNumber: 393; recordedBy: Paulo, S; **Taxon:** scientificName: *Sporobolus cordofanus* (Hochst. ex Steud.) Hérincq ex Coss.; kingdom: Plantae; family: Poaceae; genus: Sporobolus; specificEpithet: cordofanus; scientificNameAuthorship: (Hochst. ex Steud.) Hérincq ex Coss.; **Location:** continent: Africa; country: Tanzania; stateProvince: Mara; county: Serengeti; locality: Seronera river; decimalLatitude: -2.333333; decimalLongitude: 34.833333; **Event:** eventDate: 1958-04-27; **Record Level:** institutionCode: K; collectionCode: Herbarium; ownerInstitutionCode: K; basisOfRecord: PreservedSpecimen

##### Distribution

Tropical Africa

#### Sporobolus coromandelianus

(Retz.) Kunth

##### Materials

- **Type status:** Other material. **Occurrence:** catalogNumber: 699; recordNumber: 24269; recordedBy: Peterson, PM; Soreng, RJ; Romaschenko, K; Mbago, F; **Taxon:** scientificName: *Sporobolus coromandelianus* (Retz.) Kunth; kingdom: Plantae; family: Poaceae; genus: Sporobolus; specificEpithet: coromandelianus; scientificNameAuthorship: (Retz.) Kunth; **Location:** continent: Africa; country: Tanzania; stateProvince: Shinyanga; locality: Naabi Hill Gate; verbatimLocality: Serengeti National Park, at 12 km NW of Naabi Hill Gate.; minimumElevationInMeters: 1651; decimalLatitude: -2.73597; decimalLongitude: 34.95284; **Event:** eventDate: 2012-06-16; **Record Level:** institutionCode: US; collectionCode: Herbarium; ownerInstitutionCode: US; basisOfRecord: PreservedSpecimen

##### Distribution

Widespread

#### Sporobolus discosporus

Nees

##### Materials

- **Type status:** Other material. **Occurrence:** catalogNumber: K000495977; recordNumber: 363; recordedBy: Paulo, S; **Taxon:** scientificName: *Sporobolus discosporus* Nees; kingdom: Plantae; family: Poaceae; genus: Sporobolus; specificEpithet: discosporus; scientificNameAuthorship: Nees; **Location:** continent: Africa; country: Tanzania; stateProvince: Arusha; county: Ngorongoro; locality: Soitayai; verbatimLocality: (Soit Ayai in Serengeti map); decimalLatitude: -2.416667; decimalLongitude: 35.333333; **Event:** eventDate: 1958-04-23; **Record Level:** institutionCode: K; collectionCode: Herbarium; ownerInstitutionCode: K; basisOfRecord: PreservedSpecimen
- **Type status:** Other material. **Occurrence:** catalogNumber: 890; recordNumber: 363; recordedBy: Paulo, S; **Taxon:** scientificName: *Sporobolus discosporus* Nees; kingdom: Plantae; family: Poaceae; genus: Sporobolus; specificEpithet: discosporus; scientificNameAuthorship: Nees; **Location:** continent: Africa; country: Tanzania; stateProvince: Arusha; county: Ngorongoro; locality: Soitayai; verbatimLocality: (Soit Ayai in Serengeti map); decimalLatitude: -2.416667; decimalLongitude: 35.333333; **Event:** eventDate: 1958-04-23; **Record Level:** institutionCode: EA; collectionCode: Herbarium; ownerInstitutionCode: EA; basisOfRecord: PreservedSpecimen

##### Distribution

Eastern & Southern Africa

#### Sporobolus festivus

Hochst. ex A.Rich.

##### Materials

- **Type status:** Other material. **Occurrence:** catalogNumber: K000984170; recordNumber: 9842; recordedBy: Greenway, PJ; **Taxon:** scientificName: *Sporobolus festivus* Hochst. ex A.Rich.; kingdom: Plantae; family: Poaceae; genus: Sporobolus; specificEpithet: festivus; scientificNameAuthorship: Hochst. ex A.Rich.; **Location:** continent: Africa; country: Tanzania; stateProvince: Mara; county: Serengeti; locality: Seronera; verbatimLocality: Serengeti; minimumElevationInMeters: 1554; decimalLatitude: -2.45; decimalLongitude: 34.833333; **Event:** eventDate: 1961-03-17; **Record Level:** institutionCode: K; collectionCode: Herbarium; ownerInstitutionCode: K; basisOfRecord: PreservedSpecimen
- **Type status:** Other material. **Occurrence:** catalogNumber: K000984171; recordNumber: 41; recordedBy: Brooks, GP; **Taxon:** scientificName: *Sporobolus festivus* Hochst. ex A.Rich.; kingdom: Plantae; family: Poaceae; genus: Sporobolus; specificEpithet: festivus; scientificNameAuthorship: Hochst. ex A.Rich.; **Location:** continent: Africa; country: Tanzania; stateProvince: Mara; county: Serengeti; locality: Ikoma; minimumElevationInMeters: 1371; decimalLatitude: -2.066667; decimalLongitude: 34.616667; **Event:** eventDate: 1954-02-19; **Record Level:** institutionCode: K; collectionCode: Herbarium; ownerInstitutionCode: K; basisOfRecord: PreservedSpecimen
- **Type status:** Other material. **Occurrence:** catalogNumber: K000984172; recordNumber: 6183; recordedBy: Newbould, JB; **Taxon:** scientificName: *Sporobolus festivus* Hochst. ex A.Rich.; kingdom: Plantae; family: Poaceae; genus: Sporobolus; specificEpithet: festivus; scientificNameAuthorship: Hochst. ex A.Rich.; **Location:** continent: Africa; country: Tanzania; stateProvince: Mara; county: Serengeti; locality: Engare Nanyuki; verbatimLocality: E. Serengeti; minimumElevationInMeters: 1524; decimalLatitude: -2.616667; decimalLongitude: 35.216667; **Event:** eventDate: 1962-07-18; **Record Level:** institutionCode: K; collectionCode: Herbarium; ownerInstitutionCode: K; basisOfRecord: PreservedSpecimen
- **Type status:** Other material. **Occurrence:** catalogNumber: K000984173; recordNumber: 9906; recordedBy: Greenway, PJ; **Taxon:** scientificName: *Sporobolus festivus* Hochst. ex A.Rich.; kingdom: Plantae; family: Poaceae; genus: Sporobolus; specificEpithet: festivus; scientificNameAuthorship: Hochst. ex A.Rich.; **Location:** continent: Africa; country: Tanzania; stateProvince: Mara; county: Serengeti; locality: Seronera; verbatimLocality: Seronera to Soitayai, Mile 10; minimumElevationInMeters: 1646; decimalLatitude: -2.45; decimalLongitude: 34.833333; **Event:** eventDate: 1961-03-27; **Record Level:** institutionCode: K; collectionCode: Herbarium; ownerInstitutionCode: K; basisOfRecord: PreservedSpecimen
- **Type status:** Other material. **Occurrence:** catalogNumber: 700; recordNumber: s.n.; recordedBy: Mboya, E; **Taxon:** scientificName: *Sporobolus festivus* Hochst. ex A.Rich.; kingdom: Plantae; family: Poaceae; genus: Sporobolus; specificEpithet: festivus; scientificNameAuthorship: Hochst. ex A.Rich.; **Location:** continent: Africa; country: Tanzania; stateProvince: Mara; county: Serengeti; locality: Serengeti National Park; verbatimLocality: T1. Serengeti National Park. Ndabaka-Seronera Road.; minimumElevationInMeters: 1297; decimalLatitude: -2.28; decimalLongitude: 34.5; **Event:** eventDate: 2004-02-04; **Record Level:** institutionCode: MO; collectionCode: Herbarium; ownerInstitutionCode: MO; basisOfRecord: PreservedSpecimen
- **Type status:** Other material. **Occurrence:** catalogNumber: 701; recordNumber: 24256; recordedBy: Peterson, PM; Soreng, RJ; Romaschenko, K; Mbago, F; **Taxon:** scientificName: *Sporobolus festivus* Hochst. ex A.Rich.; kingdom: Plantae; family: Poaceae; genus: Sporobolus; specificEpithet: festivus; scientificNameAuthorship: Hochst. ex A.Rich.; **Location:** continent: Africa; country: Tanzania; stateProvince: Shinyanga; locality: Naabi Hill Gate; verbatimLocality: Serengeti National Park, Naabi Hill Gate (at 0.5 km N).; minimumElevationInMeters: 1734; decimalLatitude: -2.83139; decimalLongitude: 34.99672; **Event:** eventDate: 2012-06-16; **Record Level:** institutionCode: US; collectionCode: Herbarium; ownerInstitutionCode: US; basisOfRecord: PreservedSpecimen
- **Type status:** Other material. **Occurrence:** catalogNumber: 702; recordNumber: 24289; recordedBy: Peterson, PM; Soreng, RJ; Romaschenko, K; Mbago, F; **Taxon:** scientificName: *Sporobolus festivus* Hochst. ex A.Rich.; kingdom: Plantae; family: Poaceae; genus: Sporobolus; specificEpithet: festivus; scientificNameAuthorship: Hochst. ex A.Rich.; **Location:** continent: Africa; country: Tanzania; stateProvince: Mara; county: Serengeti; locality: Mbuzi Mare camp; verbatimLocality: Serengeti National Park, near Mbuzi Mare camp.; minimumElevationInMeters: 1552; decimalLatitude: -2.23332; decimalLongitude: 34.96467; **Event:** eventDate: 2012-06-17; **Record Level:** institutionCode: US; collectionCode: Herbarium; ownerInstitutionCode: US; basisOfRecord: PreservedSpecimen

##### Distribution

Tropical Africa & Asia

#### Sporobolus fimbriatus

(Nees ex Trin.) Nees

Sporobolus fimbriatus (Nees ex Trin.) Nees var. latifolius Stent

##### Materials

- **Type status:** Other material. **Occurrence:** catalogNumber: K000984159; recordNumber: 9092; recordedBy: Greenway, PJ; **Taxon:** scientificName: *Sporobolus fimbriatus* (Nees ex Trin.) Nees; kingdom: Plantae; family: Poaceae; genus: Sporobolus; specificEpithet: fimbriatus; scientificNameAuthorship: (Nees ex Trin.) Nees; **Location:** continent: Africa; country: Tanzania; stateProvince: Mara; county: Serengeti; locality: Ndabaka Plains; minimumElevationInMeters: 1280; decimalLatitude: -2.116667; decimalLongitude: 34.916667; **Event:** eventDate: 1956-12-01; **Record Level:** institutionCode: K; collectionCode: Herbarium; ownerInstitutionCode: K; basisOfRecord: PreservedSpecimen
- **Type status:** Other material. **Occurrence:** catalogNumber: K000984165; recordNumber: 9852; recordedBy: Greenway, PJ; **Taxon:** scientificName: *Sporobolus fimbriatus* (Nees ex Trin.) Nees; kingdom: Plantae; family: Poaceae; genus: Sporobolus; specificEpithet: fimbriatus; scientificNameAuthorship: (Nees ex Trin.) Nees; **Location:** continent: Africa; country: Tanzania; stateProvince: Mara; county: Serengeti; locality: Seronera; minimumElevationInMeters: 1554; decimalLatitude: -2.45; decimalLongitude: 34.833333; **Event:** eventDate: 1961-03-20; **Record Level:** institutionCode: K; collectionCode: Herbarium; ownerInstitutionCode: K; basisOfRecord: PreservedSpecimen
- **Type status:** Other material. **Occurrence:** catalogNumber: K000984166; recordNumber: 370; recordedBy: Paulo, S; **Taxon:** scientificName: *Sporobolus fimbriatus* (Nees ex Trin.) Nees; kingdom: Plantae; family: Poaceae; genus: Sporobolus; specificEpithet: fimbriatus; scientificNameAuthorship: (Nees ex Trin.) Nees; **Location:** continent: Africa; country: Tanzania; stateProvince: Shinyanga; locality: Naabi; verbatimLocality: N.W. of Naabi; decimalLatitude: -2.933333; decimalLongitude: 35.083333; **Event:** eventDate: 1958-04-24; **Record Level:** institutionCode: K; collectionCode: Herbarium; ownerInstitutionCode: K; basisOfRecord: PreservedSpecimen
- **Type status:** Other material. **Occurrence:** catalogNumber: 703; recordNumber: 24258; recordedBy: Peterson, PM; Soreng, RJ; Romaschenko, K; Mbago, F; **Taxon:** scientificName: *Sporobolus fimbriatus* (Nees ex Trin.) Nees; kingdom: Plantae; family: Poaceae; genus: Sporobolus; specificEpithet: fimbriatus; scientificNameAuthorship: (Nees ex Trin.) Nees; **Location:** continent: Africa; country: Tanzania; stateProvince: Shinyanga; locality: Naabi Hill Gate; verbatimLocality: Serengeti National Park, Naabi Hill Gate (at 0.5 km N).; minimumElevationInMeters: 1734; decimalLatitude: -2.83139; decimalLongitude: 34.99672; **Event:** eventDate: 2012-06-16; **Record Level:** institutionCode: US; collectionCode: Herbarium; ownerInstitutionCode: US; basisOfRecord: PreservedSpecimen
- **Type status:** Other material. **Occurrence:** catalogNumber: 704; recordNumber: 24260; recordedBy: Peterson, PM; Soreng, RJ; Romaschenko, K; Mbago, F; **Taxon:** scientificName: *Sporobolus fimbriatus* (Nees ex Trin.) Nees; kingdom: Plantae; family: Poaceae; genus: Sporobolus; specificEpithet: fimbriatus; scientificNameAuthorship: (Nees ex Trin.) Nees; **Location:** continent: Africa; country: Tanzania; stateProvince: Shinyanga; locality: Naabi Hill Gate; verbatimLocality: Serengeti National Park, Naabi Hill Gate (at 0.5 km N).; minimumElevationInMeters: 1734; decimalLatitude: -2.83139; decimalLongitude: 34.99672; **Event:** eventDate: 2012-06-16; **Record Level:** institutionCode: US; collectionCode: Herbarium; ownerInstitutionCode: US; basisOfRecord: PreservedSpecimen
- **Type status:** Other material. **Occurrence:** catalogNumber: 705; recordNumber: 24280; recordedBy: Peterson, PM; Soreng, RJ; Romaschenko, K; Mbago, F; **Taxon:** scientificName: *Sporobolus fimbriatus* (Nees ex Trin.) Nees; kingdom: Plantae; family: Poaceae; genus: Sporobolus; specificEpithet: fimbriatus; scientificNameAuthorship: (Nees ex Trin.) Nees; **Location:** continent: Africa; country: Tanzania; stateProvince: Mara; county: Serengeti; locality: Mbuzi Mare camp; verbatimLocality: Serengeti National Park, near Mbuzi Mare camp.; minimumElevationInMeters: 1552; decimalLatitude: -2.23332; decimalLongitude: 34.96467; **Event:** eventDate: 2012-06-17; **Record Level:** institutionCode: US; collectionCode: Herbarium; ownerInstitutionCode: US; basisOfRecord: PreservedSpecimen
- **Type status:** Other material. **Occurrence:** catalogNumber: 887; recordNumber: 1320; recordedBy: Bogdan; **Taxon:** scientificName: *Sporobolus fimbriatus* (Nees ex Trin.) Nees; kingdom: Plantae; family: Poaceae; genus: Sporobolus; specificEpithet: fimbriatus; scientificNameAuthorship: (Nees ex Trin.) Nees; **Location:** continent: Africa; country: Tanzania; stateProvince: Mara; county: Serengeti; locality: Seronera; decimalLatitude: -2.45; decimalLongitude: 34.833333; **Event:** eventDate: 1947-10-15; **Record Level:** collectionCode: Herbarium; ownerInstitutionCode: ?; basisOfRecord: PreservedSpecimen
- **Type status:** Other material. **Occurrence:** catalogNumber: K000495986; recordNumber: 6298; recordedBy: Newbould, JB; **Taxon:** scientificName: *Sporobolus fimbriatus* (Nees ex Trin.) Nees; kingdom: Plantae; family: Poaceae; genus: Sporobolus; specificEpithet: fimbriatus; scientificNameAuthorship: (Nees ex Trin.) Nees; **Location:** continent: Africa; country: Tanzania; stateProvince: Mara; county: Serengeti; locality: Engare Nanyuki; verbatimLocality: Serengeti; minimumElevationInMeters: 1768; decimalLatitude: -2.616667; decimalLongitude: 35.216667; **Event:** eventDate: 1962-11-22; **Record Level:** institutionCode: K; collectionCode: Herbarium; ownerInstitutionCode: K; basisOfRecord: PreservedSpecimen
- **Type status:** Other material. **Occurrence:** catalogNumber: K000495989; recordNumber: 374; recordedBy: Paulo, S; **Taxon:** scientificName: *Sporobolus fimbriatus* (Nees ex Trin.) Nees; kingdom: Plantae; family: Poaceae; genus: Sporobolus; specificEpithet: fimbriatus; scientificNameAuthorship: (Nees ex Trin.) Nees; **Location:** continent: Africa; country: Tanzania; stateProvince: Mara; county: Serengeti; locality: East Serengeti; decimalLatitude: -2.383333; decimalLongitude: 34.95; **Event:** eventDate: 1958-04-24; **Record Level:** institutionCode: K; collectionCode: Herbarium; ownerInstitutionCode: K; basisOfRecord: PreservedSpecimen
- **Type status:** Other material. **Occurrence:** catalogNumber: 1121; recordNumber: 846; recordedBy: Mollel, NP; Rusch, GM; Mwakalebe, G; **Taxon:** scientificName: *Sporobolus fimbriatus* (Nees ex Trin.) Nees; kingdom: Plantae; family: Poaceae; genus: Sporobolus; specificEpithet: fimbriatus; scientificNameAuthorship: (Nees ex Trin.) Nees; **Location:** continent: Africa; country: Tanzania; stateProvince: Mara; county: Serengeti; locality: Robanda village; verbatimLocality: Serengeti district, road to the water pool, CG01 plot. .36M, 0386088; 9763754 UTM.; minimumElevationInMeters: 1388; decimalLatitude: -2.083333; decimalLongitude: 34.666667; **Event:** eventDate: 2003-01-28; **Record Level:** institutionCode: NHT; collectionCode: Herbarium; ownerInstitutionCode: NHT; basisOfRecord: PreservedSpecimen
- **Type status:** Other material. **Occurrence:** catalogNumber: 1122; recordNumber: 5729; recordedBy: Chuwa, S; Amiyo, T; **Taxon:** scientificName: *Sporobolus fimbriatus* (Nees ex Trin.) Nees; kingdom: Plantae; family: Poaceae; genus: Sporobolus; specificEpithet: fimbriatus; scientificNameAuthorship: (Nees ex Trin.) Nees; **Location:** continent: Africa; country: Tanzania; stateProvince: Arusha; county: Ngorongoro; locality: Ngorongoro Crater; verbatimLocality: Seneto east. Localized along the crater wall.; minimumElevationInMeters: 1676; decimalLatitude: -3.166667; decimalLongitude: 35.583333; **Event:** eventDate: 1994-11-11; **Record Level:** institutionCode: NHT; collectionCode: Herbarium; ownerInstitutionCode: NHT; basisOfRecord: PreservedSpecimen
- **Type status:** Other material. **Occurrence:** catalogNumber: K000984160; recordNumber: 10735; recordedBy: Greenway, PJ; **Taxon:** scientificName: Sporobolus fimbriatus (Nees ex Trin.) Nees var. latifolius Stent; kingdom: Plantae; family: Poaceae; genus: Sporobolus; specificEpithet: fimbriatus; infraspecificEpithet: latifolius; scientificNameAuthorship: Stent; **Location:** continent: Africa; country: Tanzania; stateProvince: Arusha; county: Ngorongoro; locality: Gol Kopjes; verbatimLocality: 3 miles W. of the Eastern boundary; minimumElevationInMeters: 1646; decimalLatitude: -2.7; decimalLongitude: 35.433333; **Event:** eventDate: 1963-2; **Record Level:** institutionCode: K; collectionCode: Herbarium; ownerInstitutionCode: K; basisOfRecord: PreservedSpecimen
- **Type status:** Other material. **Occurrence:** catalogNumber: K000984161; recordNumber: 10737; recordedBy: Greenway, PJ; **Taxon:** scientificName: Sporobolus fimbriatus (Nees ex Trin.) Nees var. latifolius Stent; kingdom: Plantae; family: Poaceae; genus: Sporobolus; specificEpithet: fimbriatus; infraspecificEpithet: latifolius; scientificNameAuthorship: Stent; **Location:** continent: Africa; country: Tanzania; stateProvince: Shinyanga; locality: Naabi Hill; verbatimLocality: S.E. slope of Naabi Hill on the side of the main track; minimumElevationInMeters: 1646; decimalLatitude: -2.883333; decimalLongitude: 35.033333; **Event:** eventDate: 1962-08-10; **Record Level:** institutionCode: K; collectionCode: Herbarium; ownerInstitutionCode: K; basisOfRecord: PreservedSpecimen
- **Type status:** Other material. **Occurrence:** catalogNumber: K000984167; recordNumber: 5598; recordedBy: Leippert, H; **Taxon:** scientificName: Sporobolus fimbriatus (Nees ex Trin.) Nees var. latifolius Stent; kingdom: Plantae; family: Poaceae; genus: Sporobolus; specificEpithet: fimbriatus; infraspecificEpithet: latifolius; scientificNameAuthorship: Stent; **Location:** continent: Africa; country: Tanzania; stateProvince: Shinyanga; locality: Lake Magadi; verbatimLocality: Serengeti plain entrance of National Park near lake Magadi; minimumElevationInMeters: 1600; decimalLatitude: -2.75; decimalLongitude: 34.833333; **Event:** eventDate: 1965-03-02; **Record Level:** institutionCode: K; collectionCode: Herbarium; ownerInstitutionCode: K; basisOfRecord: PreservedSpecimen
- **Type status:** Other material. **Occurrence:** catalogNumber: K000495991; recordNumber: 9982; recordedBy: Greenway, PJ; **Taxon:** scientificName: Sporobolus fimbriatus (Nees ex Trin.) Nees var. latifolius Stent; kingdom: Plantae; family: Poaceae; genus: Sporobolus; specificEpithet: fimbriatus; infraspecificEpithet: latifolius; scientificNameAuthorship: Stent; **Location:** continent: Africa; country: Tanzania; stateProvince: Mara; county: Serengeti; locality: Seronera dam site; minimumElevationInMeters: 1448; decimalLatitude: -2.433333; decimalLongitude: 34.816667; **Event:** eventDate: 1961-04-05; **Record Level:** institutionCode: K; collectionCode: Herbarium; ownerInstitutionCode: K; basisOfRecord: PreservedSpecimen
- **Type status:** Other material. **Occurrence:** catalogNumber: K000495990; recordNumber: 10189; recordedBy: Greenway, PJ; **Taxon:** scientificName: Sporobolus fimbriatus (Nees ex Trin.) Nees var. latifolius Stent; kingdom: Plantae; family: Poaceae; genus: Sporobolus; specificEpithet: fimbriatus; infraspecificEpithet: latifolius; scientificNameAuthorship: Stent; **Location:** continent: Africa; country: Tanzania; stateProvince: Mara; county: Serengeti; locality: Seronera; verbatimLocality: West of Seronera, Serengeti.; minimumElevationInMeters: 1463; decimalLatitude: -2.333333; decimalLongitude: 34.833333; **Event:** eventDate: 1961-05-15; **Record Level:** institutionCode: K; collectionCode: Herbarium; ownerInstitutionCode: K; basisOfRecord: PreservedSpecimen
- **Type status:** Other material. **Occurrence:** catalogNumber: K000495987; recordNumber: 6189; recordedBy: Newbould, JB; Thesiger, WP; **Taxon:** scientificName: Sporobolus fimbriatus (Nees ex Trin.) Nees var. latifolius Stent; kingdom: Plantae; family: Poaceae; genus: Sporobolus; specificEpithet: fimbriatus; infraspecificEpithet: latifolius; scientificNameAuthorship: Stent; **Location:** continent: Africa; country: Tanzania; stateProvince: Mara; county: Serengeti; locality: Seronera; minimumElevationInMeters: 1524; decimalLatitude: -2.333333; decimalLongitude: 34.833333; **Event:** eventDate: 1962-07-20; **Record Level:** institutionCode: K; collectionCode: Herbarium; ownerInstitutionCode: K; basisOfRecord: PreservedSpecimen
- **Type status:** Other material. **Occurrence:** catalogNumber: K000495988; recordNumber: 10487; recordedBy: Greenway, PJ; **Taxon:** scientificName: Sporobolus fimbriatus (Nees ex Trin.) Nees var. latifolius Stent; kingdom: Plantae; family: Poaceae; genus: Sporobolus; specificEpithet: fimbriatus; infraspecificEpithet: latifolius; scientificNameAuthorship: Stent; **Location:** continent: Africa; country: Tanzania; stateProvince: Mara; county: Serengeti; locality: Serengeti; verbatimLocality: Eastern boundary, Serengeti, mile 10.6 from the Soitayai road.; minimumElevationInMeters: 1646; decimalLatitude: -2.333333; decimalLongitude: 34.833333; **Event:** eventDate: 1962-03-01; **Record Level:** institutionCode: K; collectionCode: Herbarium; ownerInstitutionCode: K; basisOfRecord: PreservedSpecimen

##### Distribution

Tropical Africa

#### Sporobolus ioclados

(Trin.) Nees

Sporobolus marginatus Hochst. ex A.Rich. | Sporobolus verdcourtii Napper | Sporobolus laetevirens Coss. | Sporobolus kentrophyllum (K.Schum.) Clayton | Sporobolus rangei Pilg.

##### Materials

- **Type status:** Other material. **Occurrence:** catalogNumber: K000984188; recordNumber: 9107; recordedBy: Greenway, PJ; **Taxon:** scientificName: *Sporobolus ioclados* (Trin.) Nees; kingdom: Plantae; family: Poaceae; genus: Sporobolus; specificEpithet: ioclados; scientificNameAuthorship: (Trin.) Nees; **Location:** continent: Africa; country: Tanzania; stateProvince: Arusha; county: Ngorongoro; locality: Olduvai Headwaters; minimumElevationInMeters: 1585; decimalLatitude: -2.95; decimalLongitude: 35.166667; **Event:** eventDate: 1956-12-04; **Record Level:** institutionCode: K; collectionCode: Herbarium; ownerInstitutionCode: K; basisOfRecord: PreservedSpecimen
- **Type status:** Other material. **Occurrence:** catalogNumber: 706; recordNumber: 1095; recordedBy: Ellemann, L; **Taxon:** scientificName: *Sporobolus ioclados* (Trin.) Nees; kingdom: Plantae; family: Poaceae; genus: Sporobolus; specificEpithet: ioclados; scientificNameAuthorship: (Trin.) Nees; **Location:** continent: Africa; country: Tanzania; stateProvince: Arusha; county: Ngorongoro; locality: Olbalbal swamp; verbatimLocality: Ngorongoro Conservation Area, south-western part of Olbalbal swamp.; minimumElevationInMeters: 1350; decimalLatitude: -3.05; decimalLongitude: 35.466; **Event:** eventDate: 1994-03-03; **Record Level:** institutionCode: AAU; collectionCode: Herbarium; ownerInstitutionCode: AAU; basisOfRecord: PreservedSpecimen
- **Type status:** Other material. **Occurrence:** catalogNumber: 707; recordNumber: 1118; recordedBy: Ellemann, L; **Taxon:** scientificName: *Sporobolus ioclados* (Trin.) Nees; kingdom: Plantae; family: Poaceae; genus: Sporobolus; specificEpithet: ioclados; scientificNameAuthorship: (Trin.) Nees; **Location:** continent: Africa; country: Tanzania; stateProvince: Arusha; county: Ngorongoro; locality: Oldonyo-o-ogol; verbatimLocality: Ngorongoro Conservation Area, Oldonyo-o-ogol.; minimumElevationInMeters: 1700; decimalLatitude: -2.75; decimalLongitude: 35.45; **Event:** eventDate: 1994-03-07; **Record Level:** institutionCode: AAU; collectionCode: Herbarium; ownerInstitutionCode: AAU; basisOfRecord: PreservedSpecimen
- **Type status:** Other material. **Occurrence:** catalogNumber: 708; recordNumber: s.n.; recordedBy: Mboya, E; **Taxon:** scientificName: *Sporobolus ioclados* (Trin.) Nees; kingdom: Plantae; family: Poaceae; genus: Sporobolus; specificEpithet: ioclados; scientificNameAuthorship: (Trin.) Nees; **Location:** continent: Africa; country: Tanzania; stateProvince: Mara; county: Serengeti; locality: Musabi plains; verbatimLocality: T1. Serengeti National Park. Musabi plains.; minimumElevationInMeters: 1297; decimalLatitude: -2.28; decimalLongitude: 34.5; **Event:** eventDate: 2004-03-07; **Record Level:** institutionCode: MO; collectionCode: Herbarium; ownerInstitutionCode: MO; basisOfRecord: PreservedSpecimen
- **Type status:** Other material. **Occurrence:** catalogNumber: 709; recordNumber: s.n.; recordedBy: Mboya, E; **Taxon:** scientificName: *Sporobolus ioclados* (Trin.) Nees; kingdom: Plantae; family: Poaceae; genus: Sporobolus; specificEpithet: ioclados; scientificNameAuthorship: (Trin.) Nees; **Location:** continent: Africa; country: Tanzania; stateProvince: Mara; county: Serengeti; locality: Serengeti National Park; verbatimLocality: T1. Serengeti National Park. Ndabaka-Seronera Road.; minimumElevationInMeters: 1294; decimalLatitude: -2.27; decimalLongitude: 34.55; **Event:** eventDate: 2004-03-09; **Record Level:** institutionCode: MO; collectionCode: Herbarium; ownerInstitutionCode: MO; basisOfRecord: PreservedSpecimen
- **Type status:** Other material. **Occurrence:** catalogNumber: 710; recordNumber: s.n.; recordedBy: Phillipson, PB; **Taxon:** scientificName: *Sporobolus ioclados* (Trin.) Nees; kingdom: Plantae; family: Poaceae; genus: Sporobolus; specificEpithet: ioclados; scientificNameAuthorship: (Trin.) Nees; **Location:** continent: Africa; country: Tanzania; stateProvince: Arusha; county: Ngorongoro; locality: Lake Magadi; verbatimLocality: Ngorongoro Conservation area Olduvai Gorge.; minimumElevationInMeters: 2250; decimalLatitude: -2.99; decimalLongitude: 35.35; **Event:** eventDate: 1905-06-19; **Record Level:** institutionCode: MO; collectionCode: Herbarium; ownerInstitutionCode: MO; basisOfRecord: PreservedSpecimen
- **Type status:** Other material. **Occurrence:** catalogNumber: K000722864; recordNumber: 10156; recordedBy: Greenway, PJ; Turner, M; Talbots, TL; **Taxon:** scientificName: *Sporobolus ioclados* (Trin.) Nees; kingdom: Plantae; family: Poaceae; genus: Sporobolus; specificEpithet: ioclados; scientificNameAuthorship: (Trin.) Nees; **Location:** continent: Africa; country: Tanzania; stateProvince: Shinyanga; locality: Naabi Hill; verbatimLocality: North east boundary, Mile 15 from Naabi Hill.; decimalLatitude: -2.883333; decimalLongitude: 35.033333; **Event:** eventDate: 1961-05-10; **Record Level:** institutionCode: K; collectionCode: Herbarium; ownerInstitutionCode: K; basisOfRecord: PreservedSpecimen
- **Type status:** Other material. **Occurrence:** catalogNumber: K000722847; recordNumber: 5597; recordedBy: Leippert, H; **Taxon:** scientificName: *Sporobolus ioclados* (Trin.) Nees; kingdom: Plantae; family: Poaceae; genus: Sporobolus; specificEpithet: ioclados; scientificNameAuthorship: (Trin.) Nees; **Location:** continent: Africa; country: Tanzania; stateProvince: Shinyanga; locality: Lake Magadi; verbatimLocality: Serengeti plain, entrance of National park near Lake Magadi.; minimumElevationInMeters: 1600; decimalLatitude: -2.75; decimalLongitude: 34.833333; **Event:** eventDate: 1965-03-02; **Record Level:** institutionCode: K; collectionCode: Herbarium; ownerInstitutionCode: K; basisOfRecord: PreservedSpecimen
- **Type status:** Other material. **Occurrence:** catalogNumber: K000722879; recordNumber: 10652; recordedBy: Greenway, PJ; **Taxon:** scientificName: *Sporobolus ioclados* (Trin.) Nees; kingdom: Plantae; family: Poaceae; genus: Sporobolus; specificEpithet: ioclados; scientificNameAuthorship: (Trin.) Nees; **Location:** continent: Africa; country: Tanzania; stateProvince: Mara; county: Serengeti; locality: Seronera; verbatimLocality: Mile 10 from Seronera on the Nyaraswega-Banagi Hill circuit.; minimumElevationInMeters: 1417; decimalLatitude: -2.188333; decimalLongitude: 34.835833; **Event:** eventDate: 1962-05-22; **Record Level:** institutionCode: K; collectionCode: Herbarium; ownerInstitutionCode: K; basisOfRecord: PreservedSpecimen
- **Type status:** Other material. **Occurrence:** catalogNumber: K000722854; recordNumber: 407; recordedBy: Paulo, S; **Taxon:** scientificName: *Sporobolus ioclados* (Trin.) Nees; kingdom: Plantae; family: Poaceae; genus: Sporobolus; specificEpithet: ioclados; scientificNameAuthorship: (Trin.) Nees; **Location:** continent: Africa; country: Tanzania; stateProvince: Arusha; county: Ngorongoro; locality: Salei Plain; verbatimLocality: West of Salai plains; decimalLatitude: -2.833333; decimalLongitude: 35.666667; **Event:** eventDate: 1958-04-29; **Record Level:** institutionCode: K; collectionCode: Herbarium; ownerInstitutionCode: K; basisOfRecord: PreservedSpecimen
- **Type status:** Other material. **Occurrence:** catalogNumber: K000722865; recordNumber: 6568; recordedBy: Newbould, JB; **Taxon:** scientificName: *Sporobolus ioclados* (Trin.) Nees; kingdom: Plantae; family: Poaceae; genus: Sporobolus; specificEpithet: ioclados; scientificNameAuthorship: (Trin.) Nees; **Location:** continent: Africa; country: Tanzania; stateProvince: Arusha; county: Ngorongoro; locality: Ang'ata Salei; minimumElevationInMeters: 1371; decimalLatitude: -2.666667; decimalLongitude: 35.666667; **Event:** eventDate: 1963-2; **Record Level:** institutionCode: K; collectionCode: Herbarium; ownerInstitutionCode: K; basisOfRecord: PreservedSpecimen
- **Type status:** Other material. **Occurrence:** catalogNumber: K000722919; recordNumber: 9843; recordedBy: Greenway, PJ; **Taxon:** scientificName: *Sporobolus ioclados* (Trin.) Nees; kingdom: Plantae; family: Poaceae; genus: Sporobolus; specificEpithet: ioclados; scientificNameAuthorship: (Trin.) Nees; **Location:** continent: Africa; country: Tanzania; stateProvince: Mara; county: Serengeti; locality: Seronera; minimumElevationInMeters: 1554; decimalLatitude: -2.45; decimalLongitude: 34.833333; **Event:** eventDate: 1961-03-17; **Record Level:** institutionCode: K; collectionCode: Herbarium; ownerInstitutionCode: K; basisOfRecord: PreservedSpecimen
- **Type status:** Other material. **Occurrence:** catalogNumber: K000495978; recordNumber: 10185; recordedBy: Greenway, PJ; **Taxon:** scientificName: *Sporobolus ioclados* (Trin.) Nees; kingdom: Plantae; family: Poaceae; genus: Sporobolus; specificEpithet: ioclados; scientificNameAuthorship: (Trin.) Nees; **Location:** continent: Africa; country: Tanzania; stateProvince: Mara; county: Serengeti; locality: Seronera; verbatimLocality: West of Seronera; minimumElevationInMeters: 1463; decimalLatitude: -2.45; decimalLongitude: 34.833333; **Event:** eventDate: 1961-05-15; **Record Level:** institutionCode: K; collectionCode: Herbarium; ownerInstitutionCode: K; basisOfRecord: PreservedSpecimen
- **Type status:** Other material. **Occurrence:** catalogNumber: K000722920; recordNumber: 10101; recordedBy: Greenway, PJ; **Taxon:** scientificName: *Sporobolus ioclados* (Trin.) Nees; kingdom: Plantae; family: Poaceae; genus: Sporobolus; specificEpithet: ioclados; scientificNameAuthorship: (Trin.) Nees; **Location:** continent: Africa; country: Tanzania; stateProvince: Mara; county: Serengeti; locality: Seronera; verbatimLocality: Seronera to Titushi river, mile 14.7; minimumElevationInMeters: 1493; decimalLatitude: -2.534167; decimalLongitude: 34.750278; **Event:** eventDate: 1961-04-27; **Record Level:** institutionCode: K; collectionCode: Herbarium; ownerInstitutionCode: K; basisOfRecord: PreservedSpecimen
- **Type status:** Other material. **Occurrence:** catalogNumber: K000722922; recordNumber: 6572; recordedBy: Newbould, JB; **Taxon:** scientificName: *Sporobolus ioclados* (Trin.) Nees; kingdom: Plantae; family: Poaceae; genus: Sporobolus; specificEpithet: ioclados; scientificNameAuthorship: (Trin.) Nees; **Location:** continent: Africa; country: Tanzania; stateProvince: Arusha; county: Ngorongoro; locality: Ang'ata Salei; verbatimLocality: Watumi, Ang'ata Salei; minimumElevationInMeters: 1524; decimalLatitude: -2.666667; decimalLongitude: 35.666667; **Event:** eventDate: 1963-12; **Record Level:** institutionCode: K; collectionCode: Herbarium; ownerInstitutionCode: K; basisOfRecord: PreservedSpecimen
- **Type status:** Other material. **Occurrence:** catalogNumber: K000722903; recordNumber: 10506; recordedBy: Greenway, PJ; **Taxon:** scientificName: *Sporobolus ioclados* (Trin.) Nees; kingdom: Plantae; family: Poaceae; genus: Sporobolus; specificEpithet: ioclados; scientificNameAuthorship: (Trin.) Nees; **Location:** continent: Africa; country: Tanzania; stateProvince: Shinyanga; locality: Lake Magadi; verbatimLocality: North from Lake Magadi, mile 7 towards Seronera.; minimumElevationInMeters: 1493; decimalLatitude: -2.633333; decimalLongitude: 34.833333; **Event:** eventDate: 1962-03-06; **Record Level:** institutionCode: K; collectionCode: Herbarium; ownerInstitutionCode: K; basisOfRecord: PreservedSpecimen
- **Type status:** Other material. **Occurrence:** catalogNumber: K000722905; recordNumber: 11352; recordedBy: Greenway, PJ; Hunter, H; Hunter; **Taxon:** scientificName: *Sporobolus ioclados* (Trin.) Nees; kingdom: Plantae; family: Poaceae; genus: Sporobolus; specificEpithet: ioclados; scientificNameAuthorship: (Trin.) Nees; **Location:** continent: Africa; country: Tanzania; stateProvince: Arusha; county: Ngorongoro; locality: Ol Doinyo Lengai; verbatimLocality: In a gorge, north east of Ol Doinyo Lengai.; minimumElevationInMeters: 853; decimalLatitude: -2.75; decimalLongitude: 35.9; **Event:** eventDate: 1964-03-12; **Record Level:** institutionCode: K; collectionCode: Herbarium; ownerInstitutionCode: K; basisOfRecord: PreservedSpecimen
- **Type status:** Other material. **Occurrence:** catalogNumber: K000722906; recordNumber: 10480; recordedBy: Greenway, PJ; **Taxon:** scientificName: *Sporobolus ioclados* (Trin.) Nees; kingdom: Plantae; family: Poaceae; genus: Sporobolus; specificEpithet: ioclados; scientificNameAuthorship: (Trin.) Nees; **Location:** continent: Africa; country: Tanzania; stateProvince: Mara; county: Serengeti; locality: Engare Nanyuki; minimumElevationInMeters: 1524; decimalLatitude: -2.616667; decimalLongitude: 35.216667; **Event:** eventDate: 1962-03-01; **Record Level:** institutionCode: K; collectionCode: Herbarium; ownerInstitutionCode: K; basisOfRecord: PreservedSpecimen
- **Type status:** Other material. **Occurrence:** catalogNumber: K000722907; recordNumber: 327; recordedBy: Paulo, S; **Taxon:** scientificName: *Sporobolus ioclados* (Trin.) Nees; kingdom: Plantae; family: Poaceae; genus: Sporobolus; specificEpithet: ioclados; scientificNameAuthorship: (Trin.) Nees; **Location:** continent: Africa; country: Tanzania; stateProvince: Arusha; county: Ngorongoro; locality: Olbalbal; minimumElevationInMeters: 1250; decimalLatitude: -2.95; decimalLongitude: 35.583333; **Event:** eventDate: 1958-04-20; **Record Level:** institutionCode: K; collectionCode: Herbarium; ownerInstitutionCode: K; basisOfRecord: PreservedSpecimen
- **Type status:** Other material. **Occurrence:** catalogNumber: K000495979; recordNumber: 345; recordedBy: Paulo, S; **Taxon:** scientificName: *Sporobolus ioclados* (Trin.) Nees; kingdom: Plantae; family: Poaceae; genus: Sporobolus; specificEpithet: ioclados; scientificNameAuthorship: (Trin.) Nees; **Location:** continent: Africa; country: Tanzania; stateProvince: Mara; county: Serengeti; locality: Serengeti; verbatimLocality: South Serengeti.; decimalLatitude: -2.333333; decimalLongitude: 34.833333; **Event:** eventDate: 1958-04-22; **Record Level:** institutionCode: K; collectionCode: Herbarium; ownerInstitutionCode: K; basisOfRecord: PreservedSpecimen
- **Type status:** Other material. **Occurrence:** catalogNumber: K000722892; recordNumber: 9904; recordedBy: Greenway, PJ; **Taxon:** scientificName: *Sporobolus ioclados* (Trin.) Nees; kingdom: Plantae; family: Poaceae; genus: Sporobolus; specificEpithet: ioclados; scientificNameAuthorship: (Trin.) Nees; **Location:** continent: Africa; country: Tanzania; stateProvince: Mara; county: Serengeti; locality: Seronera; verbatimLocality: Seronera to Soitayai, mile 10.; minimumElevationInMeters: 1646; decimalLatitude: -2.333333; decimalLongitude: 34.978333; **Event:** eventDate: 1961-03-27; **Record Level:** institutionCode: K; collectionCode: Herbarium; ownerInstitutionCode: K; basisOfRecord: PreservedSpecimen
- **Type status:** Other material. **Occurrence:** catalogNumber: K000722893; recordNumber: 10191; recordedBy: Greenway, PJ; **Taxon:** scientificName: *Sporobolus ioclados* (Trin.) Nees; kingdom: Plantae; family: Poaceae; genus: Sporobolus; specificEpithet: ioclados; scientificNameAuthorship: (Trin.) Nees; **Location:** continent: Africa; country: Tanzania; stateProvince: Mara; county: Serengeti; locality: Seronera Lodge; verbatimLocality: Serengeti; minimumElevationInMeters: 1448; decimalLatitude: -2.44; decimalLongitude: 34.82; **Event:** eventDate: 1961-05-16; **Record Level:** institutionCode: K; collectionCode: Herbarium; ownerInstitutionCode: K; basisOfRecord: PreservedSpecimen
- **Type status:** Other material. **Occurrence:** catalogNumber: K000722890; recordNumber: 349; recordedBy: Paulo, S; **Taxon:** scientificName: *Sporobolus ioclados* (Trin.) Nees; kingdom: Plantae; family: Poaceae; genus: Sporobolus; specificEpithet: ioclados; scientificNameAuthorship: (Trin.) Nees; **Location:** continent: Africa; country: Tanzania; stateProvince: Arusha; county: Ngorongoro; locality: Lemuta hill; decimalLatitude: -2.75; decimalLongitude: 35.266667; **Event:** eventDate: 1958-04-22; **Record Level:** institutionCode: K; collectionCode: Herbarium; ownerInstitutionCode: K; basisOfRecord: PreservedSpecimen
- **Type status:** Other material. **Occurrence:** catalogNumber: K000722883; recordNumber: 439; recordedBy: Paulo, S; **Taxon:** scientificName: *Sporobolus ioclados* (Trin.) Nees; kingdom: Plantae; family: Poaceae; genus: Sporobolus; specificEpithet: ioclados; scientificNameAuthorship: (Trin.) Nees; **Location:** continent: Africa; country: Tanzania; stateProvince: Mara; county: Serengeti; locality: Endarbark; verbatimLocality: Endarbark, Serengeti area.; **Event:** eventDate: 1958-05-09; **Record Level:** institutionCode: K; collectionCode: Herbarium; ownerInstitutionCode: K; basisOfRecord: PreservedSpecimen
- **Type status:** Other material. **Occurrence:** catalogNumber: K000722884; recordNumber: 332; recordedBy: Paulo, S; **Taxon:** scientificName: *Sporobolus ioclados* (Trin.) Nees; kingdom: Plantae; family: Poaceae; genus: Sporobolus; specificEpithet: ioclados; scientificNameAuthorship: (Trin.) Nees; **Location:** continent: Africa; country: Tanzania; stateProvince: Arusha; county: Ngorongoro; locality: Ngorongoro Crater; minimumElevationInMeters: 1341; decimalLatitude: -3.166667; decimalLongitude: 35.583333; **Event:** eventDate: 1958-04-21; **Record Level:** institutionCode: K; collectionCode: Herbarium; ownerInstitutionCode: K; basisOfRecord: PreservedSpecimen
- **Type status:** Other material. **Occurrence:** catalogNumber: K000722885; recordNumber: 391; recordedBy: Paulo, S; **Taxon:** scientificName: *Sporobolus ioclados* (Trin.) Nees; kingdom: Plantae; family: Poaceae; genus: Sporobolus; specificEpithet: ioclados; scientificNameAuthorship: (Trin.) Nees; **Location:** continent: Africa; country: Tanzania; stateProvince: Shinyanga; locality: Moru Kopjes; decimalLatitude: -2.7; decimalLongitude: 34.8; **Event:** eventDate: 1958-04-27; **Record Level:** institutionCode: K; collectionCode: Herbarium; ownerInstitutionCode: K; basisOfRecord: PreservedSpecimen
- **Type status:** Other material. **Occurrence:** catalogNumber: K000495980; recordNumber: 97; recordedBy: Brooks, GP; **Taxon:** scientificName: *Sporobolus ioclados* (Trin.) Nees; kingdom: Plantae; family: Poaceae; genus: Sporobolus; specificEpithet: ioclados; scientificNameAuthorship: (Trin.) Nees; **Location:** continent: Africa; country: Tanzania; stateProvince: Mara; county: Serengeti; locality: Banagi Hill; verbatimLocality: Serengeti plains.; minimumElevationInMeters: 1371; decimalLatitude: -2.3; decimalLongitude: 34.833333; **Event:** eventDate: 1954-03-16; **Record Level:** institutionCode: K; collectionCode: Herbarium; ownerInstitutionCode: K; basisOfRecord: PreservedSpecimen
- **Type status:** Other material. **Occurrence:** catalogNumber: K000495981; recordNumber: 64; recordedBy: Brooks, GP; **Taxon:** scientificName: *Sporobolus ioclados* (Trin.) Nees; kingdom: Plantae; family: Poaceae; genus: Sporobolus; specificEpithet: ioclados; scientificNameAuthorship: (Trin.) Nees; **Location:** continent: Africa; country: Tanzania; stateProvince: Mara; county: Serengeti; locality: Banagi Hill; verbatimLocality: Serengeti plains.; minimumElevationInMeters: 1463; decimalLatitude: -2.3; decimalLongitude: 34.833333; **Event:** eventDate: 1954-02-08; **Record Level:** institutionCode: K; collectionCode: Herbarium; ownerInstitutionCode: K; basisOfRecord: PreservedSpecimen
- **Type status:** Other material. **Occurrence:** catalogNumber: K000495982; recordNumber: 406; recordedBy: Paulo, S; **Taxon:** scientificName: *Sporobolus ioclados* (Trin.) Nees; kingdom: Plantae; family: Poaceae; genus: Sporobolus; specificEpithet: ioclados; scientificNameAuthorship: (Trin.) Nees; **Location:** continent: Africa; country: Tanzania; stateProvince: Arusha; county: Ngorongoro; locality: Salei Plain; decimalLatitude: -2.833333; decimalLongitude: 35.666667; **Event:** eventDate: 1958-04-29; **Record Level:** institutionCode: K; collectionCode: Herbarium; ownerInstitutionCode: K; basisOfRecord: PreservedSpecimen
- **Type status:** Other material. **Occurrence:** catalogNumber: K000722867; recordNumber: 236; recordedBy: Oteke, J; **Taxon:** scientificName: *Sporobolus ioclados* (Trin.) Nees; kingdom: Plantae; family: Poaceae; genus: Sporobolus; specificEpithet: ioclados; scientificNameAuthorship: (Trin.) Nees; **Location:** continent: Africa; country: Tanzania; stateProvince: Arusha; county: Ngorongoro; locality: Ang'ata Salei; verbatimLocality: East Serengeti; decimalLatitude: -2.666667; decimalLongitude: 35.666667; **Event:** eventDate: 1962-11; **Record Level:** institutionCode: K; collectionCode: Herbarium; ownerInstitutionCode: K; basisOfRecord: PreservedSpecimen

##### Distribution

Tropical Africa, Arabia & Asia

#### Sporobolus macranthelus

Chiov.

Sporobolus greenwayi Napper

##### Materials

- **Type status:** Other material. **Occurrence:** catalogNumber: K000984155; recordNumber: 2104; recordedBy: Vesey-FitzGerald, LDEF; **Taxon:** scientificName: *Sporobolus greenwayi* Napper; kingdom: Plantae; family: Poaceae; genus: Sporobolus; specificEpithet: greenwayi; scientificNameAuthorship: Napper; **Location:** continent: Africa; country: Tanzania; stateProvince: Arusha; county: Ngorongoro; locality: Ngorongoro Crater; minimumElevationInMeters: 2134; decimalLatitude: -3.166667; decimalLongitude: 35.583333; **Event:** eventDate: 1959-01-20; **Record Level:** institutionCode: K; collectionCode: Herbarium; ownerInstitutionCode: K; basisOfRecord: PreservedSpecimen
- **Type status:** Other material. **Occurrence:** catalogNumber: K000984156; recordNumber: 12526; recordedBy: Greenway, PJ; **Taxon:** scientificName: *Sporobolus greenwayi* Napper; kingdom: Plantae; family: Poaceae; genus: Sporobolus; specificEpithet: greenwayi; scientificNameAuthorship: Napper; **Location:** continent: Africa; country: Tanzania; stateProvince: Arusha; county: Ngorongoro; locality: Kimba; verbatimLocality: Ngorongoro Crater rim, S.W. side.; minimumElevationInMeters: 2256; decimalLatitude: -3.25; decimalLongitude: 35.5; **Event:** eventDate: 1966-07-03; **Record Level:** institutionCode: K; collectionCode: Herbarium; ownerInstitutionCode: K; basisOfRecord: PreservedSpecimen
- **Type status:** Other material. **Occurrence:** catalogNumber: K000984157; recordNumber: 2631; recordedBy: Chuwa, S; **Taxon:** scientificName: *Sporobolus macranthelus* Chiov.; kingdom: Plantae; family: Poaceae; genus: Sporobolus; specificEpithet: macranthelus; scientificNameAuthorship: Chiov.; **Location:** continent: Africa; country: Tanzania; stateProvince: Arusha; county: Ngorongoro; locality: Kakesio River; minimumElevationInMeters: 1700; decimalLatitude: -3.316667; decimalLongitude: 35.033333; **Event:** eventDate: 1988-04-18; **Record Level:** institutionCode: K; collectionCode: Herbarium; ownerInstitutionCode: K; basisOfRecord: PreservedSpecimen

##### Distribution

Tropical Africa

#### Sporobolus nervosus

Hochst.

##### Materials

- **Type status:** Other material. **Occurrence:** catalogNumber: K000984154; recordNumber: 2636; recordedBy: Chuwa, S; **Taxon:** scientificName: *Sporobolus nervosus* Hochst.; kingdom: Plantae; family: Poaceae; genus: Sporobolus; specificEpithet: nervosus; scientificNameAuthorship: Hochst.; **Location:** continent: Africa; country: Tanzania; stateProvince: Arusha; county: Ngorongoro; locality: Olduvai Gorge; verbatimLocality: Ngorongoro Conservation area, 2 km from the south gorge, Oldupai gorge.; minimumElevationInMeters: 1450; decimalLatitude: -2.95; decimalLongitude: 35.166667; **Event:** eventDate: 1988-04-18; **Record Level:** institutionCode: K; collectionCode: Herbarium; ownerInstitutionCode: K; basisOfRecord: PreservedSpecimen
- **Type status:** Other material. **Occurrence:** catalogNumber: K000984182; recordNumber: 402; recordedBy: Paulo, S; **Taxon:** scientificName: *Sporobolus nervosus* Hochst.; kingdom: Plantae; family: Poaceae; genus: Sporobolus; specificEpithet: nervosus; scientificNameAuthorship: Hochst.; **Location:** continent: Africa; country: Tanzania; stateProvince: Arusha; county: Ngorongoro; locality: Olbalbal; decimalLatitude: -2.95; decimalLongitude: 35.283333; **Event:** eventDate: 1958-04-28; **Record Level:** institutionCode: K; collectionCode: Herbarium; ownerInstitutionCode: K; basisOfRecord: PreservedSpecimen

##### Distribution

Eastern & Southern Africa & Arabia

#### Sporobolus panicoides

A.Rich.

##### Materials

- **Type status:** Other material. **Occurrence:** catalogNumber: 711; recordNumber: 394; recordedBy: Ellemann, L; **Taxon:** scientificName: *Sporobolus panicoides* A.Rich.; kingdom: Plantae; family: Poaceae; genus: Sporobolus; specificEpithet: panicoides; scientificNameAuthorship: A.Rich.; **Location:** continent: Africa; country: Tanzania; stateProvince: Arusha; county: Ngorongoro; locality: Endulen; verbatimLocality: Ngorongoro Conservation Area. Between Endulen Hospital and Ndiyan.; minimumElevationInMeters: 1900; decimalLatitude: -3.2; decimalLongitude: 35.266; **Event:** eventDate: 1993-04-22; **Record Level:** institutionCode: AAU; collectionCode: Herbarium; ownerInstitutionCode: AAU; basisOfRecord: PreservedSpecimen
- **Type status:** Other material. **Occurrence:** catalogNumber: K000495976; recordNumber: 428; recordedBy: Paulo, S; **Taxon:** scientificName: *Sporobolus panicoides* A.Rich.; kingdom: Plantae; family: Poaceae; genus: Sporobolus; specificEpithet: panicoides; scientificNameAuthorship: A.Rich.; **Location:** continent: Africa; country: Tanzania; stateProvince: Arusha; county: Ngorongoro; locality: Kakesio; verbatimLocality: Serengeti, North of Kakesio; decimalLatitude: -3.316667; decimalLongitude: 35.033333; **Event:** eventDate: 1958-05-03; **Record Level:** institutionCode: K; collectionCode: Herbarium; ownerInstitutionCode: K; basisOfRecord: PreservedSpecimen

##### Distribution

Eastern & Southern Africa & Yemen

#### Sporobolus pellucidus

Hochst.

##### Materials

- **Type status:** Other material. **Occurrence:** catalogNumber: K000984162; recordNumber: 9846; recordedBy: Greenway, PJ; **Taxon:** scientificName: *Sporobolus pellucidus* Hochst.; kingdom: Plantae; family: Poaceae; genus: Sporobolus; specificEpithet: pellucidus; scientificNameAuthorship: Hochst.; **Location:** continent: Africa; country: Tanzania; stateProvince: Mara; county: Serengeti; locality: Seronera; verbatimLocality: Serengeti; minimumElevationInMeters: 1554; decimalLatitude: -2.45; decimalLongitude: 34.833333; **Event:** eventDate: 1961-03-18; **Record Level:** institutionCode: K; collectionCode: Herbarium; ownerInstitutionCode: K; basisOfRecord: PreservedSpecimen
- **Type status:** Other material. **Occurrence:** catalogNumber: K000984163; recordNumber: 6375; recordedBy: Newbould, JB; **Taxon:** scientificName: *Sporobolus pellucidus* Hochst.; kingdom: Plantae; family: Poaceae; genus: Sporobolus; specificEpithet: pellucidus; scientificNameAuthorship: Hochst.; **Location:** continent: Africa; country: Tanzania; stateProvince: Arusha; county: Ngorongoro; locality: Endoinyo Emboleh; minimumElevationInMeters: 1676; decimalLatitude: -3.083333; decimalLongitude: 35.416667; **Event:** eventDate: 1962-12-14; **Record Level:** institutionCode: K; collectionCode: Herbarium; ownerInstitutionCode: K; basisOfRecord: PreservedSpecimen
- **Type status:** Other material. **Occurrence:** catalogNumber: K000984164; recordNumber: 10943; recordedBy: Greenway, PJ; **Taxon:** scientificName: *Sporobolus pellucidus* Hochst.; kingdom: Plantae; family: Poaceae; genus: Sporobolus; specificEpithet: pellucidus; scientificNameAuthorship: Hochst.; **Location:** continent: Africa; country: Tanzania; stateProvince: Mara; county: Serengeti; locality: Campi ya Mawi; minimumElevationInMeters: 1554; decimalLatitude: -2.166667; decimalLongitude: 35; **Event:** eventDate: 1963-01-14; **Record Level:** institutionCode: K; collectionCode: Herbarium; ownerInstitutionCode: K; basisOfRecord: PreservedSpecimen
- **Type status:** Other material. **Occurrence:** catalogNumber: K000984183; recordNumber: 211; recordedBy: Braun, HMH; **Taxon:** scientificName: *Sporobolus pellucidus* Hochst.; kingdom: Plantae; family: Poaceae; genus: Sporobolus; specificEpithet: pellucidus; scientificNameAuthorship: Hochst.; **Location:** continent: Africa; country: Tanzania; stateProvince: Mara; county: Serengeti; locality: Togoro Plains; minimumElevationInMeters: 1550; decimalLatitude: -2.166667; decimalLongitude: 34.916667; **Event:** eventDate: 1967-05-05; **Record Level:** institutionCode: K; collectionCode: Herbarium; ownerInstitutionCode: K; basisOfRecord: PreservedSpecimen

##### Distribution

Tropical Africa & Arabia

#### Sporobolus pyramidalis

P.Beauv.

##### Materials

- **Type status:** Other material. **Occurrence:** catalogNumber: K000984184; recordNumber: 13312; recordedBy: Greenway, PJ; **Taxon:** scientificName: *Sporobolus pyramidalis* P.Beauv.; kingdom: Plantae; family: Poaceae; genus: Sporobolus; specificEpithet: pyramidalis; scientificNameAuthorship: P.Beauv.; **Location:** continent: Africa; country: Tanzania; stateProvince: Mara; county: Serengeti; locality: Kirawira Plains; minimumElevationInMeters: 1189; decimalLatitude: -2.166667; decimalLongitude: 34.15; **Event:** eventDate: 1968-02-20; **Record Level:** institutionCode: K; collectionCode: Herbarium; ownerInstitutionCode: K; basisOfRecord: PreservedSpecimen
- **Type status:** Other material. **Occurrence:** catalogNumber: K000984185; recordNumber: 10068; recordedBy: Greenway, PJ; **Taxon:** scientificName: *Sporobolus pyramidalis* P.Beauv.; kingdom: Plantae; family: Poaceae; genus: Sporobolus; specificEpithet: pyramidalis; scientificNameAuthorship: P.Beauv.; **Location:** continent: Africa; country: Tanzania; stateProvince: Mara; county: Serengeti; locality: Seronera; verbatimLocality: Serengeti; minimumElevationInMeters: 1463; decimalLatitude: -2.45; decimalLongitude: 34.833333; **Event:** eventDate: 1961-04-19; **Record Level:** institutionCode: K; collectionCode: Herbarium; ownerInstitutionCode: K; basisOfRecord: PreservedSpecimen
- **Type status:** Other material. **Occurrence:** catalogNumber: K000984186; recordNumber: 10389; recordedBy: Greenway, PJ; **Taxon:** scientificName: *Sporobolus pyramidalis* P.Beauv.; kingdom: Plantae; family: Poaceae; genus: Sporobolus; specificEpithet: pyramidalis; scientificNameAuthorship: P.Beauv.; **Location:** continent: Africa; country: Tanzania; stateProvince: Mara; county: Serengeti; locality: Tabora; minimumElevationInMeters: 1554; decimalLatitude: -1.816667; decimalLongitude: 34.633333; **Event:** eventDate: 1961-06-22; **Record Level:** institutionCode: K; collectionCode: Herbarium; ownerInstitutionCode: K; basisOfRecord: PreservedSpecimen
- **Type status:** Other material. **Occurrence:** catalogNumber: K000984187; recordNumber: 2626; recordedBy: Chuwa, S; **Taxon:** scientificName: *Sporobolus pyramidalis* P.Beauv.; kingdom: Plantae; family: Poaceae; genus: Sporobolus; specificEpithet: pyramidalis; scientificNameAuthorship: P.Beauv.; **Location:** continent: Africa; country: Tanzania; stateProvince: Arusha; county: Ngorongoro; locality: Kakesio plain; minimumElevationInMeters: 1750; decimalLatitude: -3.316667; decimalLongitude: 35.033333; **Event:** eventDate: 1988-04-17; **Record Level:** institutionCode: K; collectionCode: Herbarium; ownerInstitutionCode: K; basisOfRecord: PreservedSpecimen
- **Type status:** Other material. **Occurrence:** catalogNumber: 712; recordNumber: s.n.; recordedBy: Mboya, E; **Taxon:** scientificName: *Sporobolus pyramidalis* P.Beauv.; kingdom: Plantae; family: Poaceae; genus: Sporobolus; specificEpithet: pyramidalis; scientificNameAuthorship: P.Beauv.; **Location:** continent: Africa; country: Tanzania; stateProvince: Mara; county: Serengeti; locality: Serengeti Research Centre; verbatimLocality: T1. Serengeti National Park. Serengeti Research Centre. Tawiri Hostel.; minimumElevationInMeters: 1551; decimalLatitude: -2.38; decimalLongitude: 34.85; **Event:** eventDate: 2004-03-04; **Record Level:** institutionCode: MO; collectionCode: Herbarium; ownerInstitutionCode: MO; basisOfRecord: PreservedSpecimen
- **Type status:** Other material. **Occurrence:** catalogNumber: 713; recordNumber: 24298; recordedBy: Peterson, PM; Soreng, RJ; Romaschenko, K; Mbago, F; **Taxon:** scientificName: *Sporobolus pyramidalis* P.Beauv.; kingdom: Plantae; family: Poaceae; genus: Sporobolus; specificEpithet: pyramidalis; scientificNameAuthorship: P.Beauv.; **Location:** continent: Africa; country: Tanzania; stateProvince: Mara; county: Serengeti; locality: Lobo Lodge; verbatimLocality: Serengeti National Park, on top of Lobo Ridge above Lobo Lodge.; minimumElevationInMeters: 1817; decimalLatitude: -1.99967; decimalLongitude: 35.16778; **Event:** eventDate: 2012-06-17; **Record Level:** institutionCode: US; collectionCode: Herbarium; ownerInstitutionCode: US; basisOfRecord: PreservedSpecimen

##### Distribution

Widespread

#### Sporobolus rigidifolius

(Trin.) Mez ex Veldkamp

##### Materials

- **Type status:** Other material. **Occurrence:** catalogNumber: 714; recordNumber: 24317; recordedBy: Peterson, PM; Soreng, RJ; Romaschenko, K; Mbago, F; **Taxon:** scientificName: *Sporobolus rigidifolius* (Trin.) Mez ex Veldkamp; kingdom: Plantae; family: Poaceae; genus: Sporobolus; specificEpithet: rigidifolius; scientificNameAuthorship: (Trin.) Mez ex Veldkamp; **Location:** continent: Africa; country: Tanzania; stateProvince: Arusha; county: Ngorongoro; locality: Lake Magadi; verbatimLocality: Ngorongoro Conservation Area, W side of Lake Magadi.; minimumElevationInMeters: 1739; decimalLatitude: -3.17713; decimalLongitude: 35.51548; **Event:** eventDate: 2012-06-19; **Record Level:** institutionCode: US; collectionCode: Herbarium; ownerInstitutionCode: US; basisOfRecord: PreservedSpecimen

##### Distribution

Tropical Africa

#### Sporobolus sanguineus

Rendle

Sporobolus homblei De Wild.

##### Materials

- **Type status:** Other material. **Occurrence:** catalogNumber: K000984189; recordNumber: 165; recordedBy: Braun, HMH; **Taxon:** scientificName: *Sporobolus homblei* De Wild.; kingdom: Plantae; family: Poaceae; genus: Sporobolus; specificEpithet: homblei; scientificNameAuthorship: De Wild.; **Location:** continent: Africa; country: Tanzania; stateProvince: Arusha; county: Ngorongoro; locality: Lake Magadi; verbatimLocality: Southern shore of Lake Magadi, North - East of Lerai forest, Ngorongoro Crater; minimumElevationInMeters: 1650; decimalLatitude: -3.25; decimalLongitude: 35.5; **Event:** eventDate: 1967-11-26; **Record Level:** institutionCode: K; collectionCode: Herbarium; ownerInstitutionCode: K; basisOfRecord: PreservedSpecimen
- **Type status:** Other material. **Occurrence:** catalogNumber: K000984190; recordNumber: 6170; recordedBy: Newbould, JB; **Taxon:** scientificName: *Sporobolus homblei* De Wild.; kingdom: Plantae; family: Poaceae; genus: Sporobolus; specificEpithet: homblei; scientificNameAuthorship: De Wild.; **Location:** continent: Africa; country: Tanzania; stateProvince: Arusha; county: Ngorongoro; locality: Ngorongoro Crater; minimumElevationInMeters: 1676; decimalLatitude: -3.166667; decimalLongitude: 35.583333; **Event:** eventDate: 1962-07-14; **Record Level:** institutionCode: K; collectionCode: Herbarium; ownerInstitutionCode: K; basisOfRecord: PreservedSpecimen
- **Type status:** Other material. **Occurrence:** catalogNumber: K000984191; recordNumber: 6297; recordedBy: Newbould, JB; **Taxon:** scientificName: *Sporobolus homblei* De Wild.; kingdom: Plantae; family: Poaceae; genus: Sporobolus; specificEpithet: homblei; scientificNameAuthorship: De Wild.; **Location:** continent: Africa; country: Tanzania; stateProvince: Mara; county: Serengeti; locality: Engare Nanyuki; verbatimLocality: Serengeti; minimumElevationInMeters: 1768; decimalLatitude: -2.616667; decimalLongitude: 35.216667; **Event:** eventDate: 1962-11-22; **Record Level:** institutionCode: K; collectionCode: Herbarium; ownerInstitutionCode: K; basisOfRecord: PreservedSpecimen
- **Type status:** Other material. **Occurrence:** catalogNumber: K000495997; recordNumber: 10138; recordedBy: Greenway, PJ; Turner, M; **Taxon:** scientificName: *Sporobolus homblei* De Wild.; kingdom: Plantae; family: Poaceae; genus: Sporobolus; specificEpithet: homblei; scientificNameAuthorship: De Wild.; **Location:** continent: Africa; country: Tanzania; stateProvince: Mara; county: Serengeti; locality: Barelamangi salt lick; verbatimLocality: T1; **Event:** eventDate: 1961-04-30; **Record Level:** institutionCode: K; collectionCode: Herbarium; ownerInstitutionCode: K; basisOfRecord: PreservedSpecimen
- **Type status:** Other material. **Occurrence:** catalogNumber: K000495996; recordNumber: 12594; recordedBy: Greenway, PJ; Kanuri; **Taxon:** scientificName: *Sporobolus homblei* De Wild.; kingdom: Plantae; family: Poaceae; genus: Sporobolus; specificEpithet: homblei; scientificNameAuthorship: De Wild.; **Location:** continent: Africa; country: Tanzania; stateProvince: Arusha; county: Ngorongoro; locality: Hippo pool - Ngorongoro; verbatimLocality: Near Hippo pool, SE side of Ngorongoro crater floor.; decimalLatitude: -3.25; decimalLongitude: 35.583333; **Event:** eventDate: 1966-07-21; **Record Level:** institutionCode: K; collectionCode: Herbarium; ownerInstitutionCode: K; basisOfRecord: PreservedSpecimen
- **Type status:** Other material. **Occurrence:** catalogNumber: K000495995; recordNumber: 2105; recordedBy: Vesey-FitzGerald, LDEF; **Taxon:** scientificName: *Sporobolus homblei* De Wild.; kingdom: Plantae; family: Poaceae; genus: Sporobolus; specificEpithet: homblei; scientificNameAuthorship: De Wild.; **Location:** continent: Africa; country: Tanzania; stateProvince: Arusha; county: Ngorongoro; locality: Ngorongoro Crater; verbatimLocality: Lowest part of crater floor.; minimumElevationInMeters: 1829; decimalLatitude: -3.166667; decimalLongitude: 35.583333; **Event:** eventDate: 1959-01-20; **Record Level:** institutionCode: K; collectionCode: Herbarium; ownerInstitutionCode: K; basisOfRecord: PreservedSpecimen

##### Distribution

Tropical Africa

#### Sporobolus spicatus

(Vahl) Kunth

##### Materials

- **Type status:** Other material. **Occurrence:** catalogNumber: 715; recordNumber: 24315; recordedBy: Peterson, PM; Soreng, RJ; Romaschenko, K; Mbago, F; **Taxon:** scientificName: *Sporobolus spicatus* (Vahl) Kunth; kingdom: Plantae; family: Poaceae; genus: Sporobolus; specificEpithet: spicatus; scientificNameAuthorship: (Vahl) Kunth; **Location:** continent: Africa; country: Tanzania; stateProvince: Arusha; county: Ngorongoro; locality: Lake Magadi; verbatimLocality: Ngorongoro Conservation Area, W side of Lake Magadi.; minimumElevationInMeters: 1739; decimalLatitude: -3.17713; decimalLongitude: 35.51548; **Event:** eventDate: 2012-06-19; **Record Level:** institutionCode: US; collectionCode: Herbarium; ownerInstitutionCode: US; basisOfRecord: PreservedSpecimen
- **Type status:** Other material. **Occurrence:** catalogNumber: K000765003; recordNumber: 9984; recordedBy: Greenway, PJ; **Taxon:** scientificName: *Sporobolus spicatus* (Vahl) Kunth; kingdom: Plantae; family: Poaceae; genus: Sporobolus; specificEpithet: spicatus; scientificNameAuthorship: (Vahl) Kunth; **Location:** continent: Africa; country: Tanzania; stateProvince: Mara; county: Serengeti; locality: Seronera dam site; minimumElevationInMeters: 1448; decimalLatitude: -2.433333; decimalLongitude: 34.816667; **Event:** eventDate: 1961-04-05; **Record Level:** institutionCode: K; collectionCode: Herbarium; ownerInstitutionCode: K; basisOfRecord: PreservedSpecimen
- **Type status:** Other material. **Occurrence:** catalogNumber: K000495983; recordNumber: 286; recordedBy: Clair-Thompson, GN; **Taxon:** scientificName: *Sporobolus spicatus* (Vahl) Kunth; kingdom: Plantae; family: Poaceae; genus: Sporobolus; specificEpithet: spicatus; scientificNameAuthorship: (Vahl) Kunth; **Location:** continent: Africa; country: Tanzania; stateProvince: Arusha; county: Ngorongoro; locality: Oldonyo Lengai Mt.; verbatimLocality: SE slopes (occurs also on NE slopes & probably on hill sides); minimumElevationInMeters: 1219; maximumElevationInMeters: 1829; decimalLatitude: -2.75; decimalLongitude: 35.9; **Event:** eventDate: 1932-02-02; **Record Level:** institutionCode: K; collectionCode: Herbarium; ownerInstitutionCode: K; basisOfRecord: PreservedSpecimen
- **Type status:** Other material. **Occurrence:** catalogNumber: K000722994; recordNumber: 19298; recordedBy: Raynal, J; **Taxon:** scientificName: *Sporobolus spicatus* (Vahl) Kunth; kingdom: Plantae; family: Poaceae; genus: Sporobolus; specificEpithet: spicatus; scientificNameAuthorship: (Vahl) Kunth; **Location:** continent: Africa; country: Tanzania; stateProvince: Arusha; county: Ngorongoro; locality: Olduvai; verbatimLocality: Ngorongoro conservancy area. Olduvai, confluence of main and side gorges. Bottom of gorge, west bank, site of Zinjanthropus.; minimumElevationInMeters: 1500; maximumElevationInMeters: 1520; decimalLatitude: -2.95; decimalLongitude: 35.166667; **Record Level:** institutionCode: K; collectionCode: Herbarium; ownerInstitutionCode: K; basisOfRecord: PreservedSpecimen
- **Type status:** Other material. **Occurrence:** catalogNumber: K000722997; recordNumber: 11350; recordedBy: Greenway, PJ; Hunter, J; Hunter; **Taxon:** scientificName: *Sporobolus spicatus* (Vahl) Kunth; kingdom: Plantae; family: Poaceae; genus: Sporobolus; specificEpithet: spicatus; scientificNameAuthorship: (Vahl) Kunth; **Location:** continent: Africa; country: Tanzania; stateProvince: Arusha; county: Ngorongoro; locality: Ol Doinyo Lengai; verbatimLocality: East of; minimumElevationInMeters: 914; decimalLatitude: -2.75; decimalLongitude: 35.9; **Event:** eventDate: 1964-03-12; **Record Level:** institutionCode: K; collectionCode: Herbarium; ownerInstitutionCode: K; basisOfRecord: PreservedSpecimen
- **Type status:** Other material. **Occurrence:** catalogNumber: K000722986; recordNumber: 25538; recordedBy: Richards, M; **Taxon:** scientificName: *Sporobolus spicatus* (Vahl) Kunth; kingdom: Plantae; family: Poaceae; genus: Sporobolus; specificEpithet: spicatus; scientificNameAuthorship: (Vahl) Kunth; **Location:** continent: Africa; country: Tanzania; stateProvince: Arusha; county: Ngorongoro; locality: Oldonyo Lengai Mt.; verbatimLocality: At foot of volcano.; minimumElevationInMeters: 914; decimalLatitude: -2.75; decimalLongitude: 35.9; **Event:** eventDate: 1970-02-26; **Record Level:** institutionCode: K; collectionCode: Herbarium; ownerInstitutionCode: K; basisOfRecord: PreservedSpecimen
- **Type status:** Other material. **Occurrence:** catalogNumber: K000722992; recordNumber: 330; recordedBy: Paulo, S; **Taxon:** scientificName: *Sporobolus spicatus* (Vahl) Kunth; kingdom: Plantae; family: Poaceae; genus: Sporobolus; specificEpithet: spicatus; scientificNameAuthorship: (Vahl) Kunth; **Location:** continent: Africa; country: Tanzania; stateProvince: Arusha; county: Ngorongoro; locality: Ngorongoro Crater; minimumElevationInMeters: 1341; decimalLatitude: -3.166667; decimalLongitude: 35.583333; **Event:** eventDate: 1958-04-21; **Record Level:** institutionCode: K; collectionCode: Herbarium; ownerInstitutionCode: K; basisOfRecord: PreservedSpecimen
- **Type status:** Other material. **Occurrence:** catalogNumber: K000765010; recordNumber: 10158; recordedBy: Greenway, PJ; Turner, M; Talbots, TL; **Taxon:** scientificName: *Sporobolus spicatus* (Vahl) Kunth; kingdom: Plantae; family: Poaceae; genus: Sporobolus; specificEpithet: spicatus; scientificNameAuthorship: (Vahl) Kunth; **Location:** continent: Africa; country: Tanzania; stateProvince: Shinyanga; locality: Naabi Hill; verbatimLocality: NE boundary, mile 15 from Naabi hill.; decimalLatitude: -2.883333; decimalLongitude: 35.033333; **Event:** eventDate: 1961-05-10; **Record Level:** institutionCode: K; collectionCode: Herbarium; ownerInstitutionCode: K; basisOfRecord: PreservedSpecimen
- **Type status:** Other material. **Occurrence:** catalogNumber: K000765002; recordNumber: 10151; recordedBy: Greenway, PJ; **Taxon:** scientificName: *Sporobolus spicatus* (Vahl) Kunth; kingdom: Plantae; family: Poaceae; genus: Sporobolus; specificEpithet: spicatus; scientificNameAuthorship: (Vahl) Kunth; **Location:** continent: Africa; country: Tanzania; stateProvince: Mara; county: Serengeti; locality: Seronera; minimumElevationInMeters: 1463; decimalLatitude: -2.333333; decimalLongitude: 34.833333; **Event:** eventDate: 1961-05-09; **Record Level:** institutionCode: K; collectionCode: Herbarium; ownerInstitutionCode: K; basisOfRecord: PreservedSpecimen
- **Type status:** Other material. **Occurrence:** catalogNumber: K000765004; recordNumber: 10525; recordedBy: Greenway, PJ; Turner, M; **Taxon:** scientificName: *Sporobolus spicatus* (Vahl) Kunth; kingdom: Plantae; family: Poaceae; genus: Sporobolus; specificEpithet: spicatus; scientificNameAuthorship: (Vahl) Kunth; **Location:** continent: Africa; country: Tanzania; stateProvince: Arusha; county: Ngorongoro; locality: Gol Kopjes; verbatimLocality: Gol kopjes, mile 8 east of Naabi hill and mile 3 from the eastern boundary.; minimumElevationInMeters: 1646; **Event:** eventDate: 1962-03-15; **Record Level:** institutionCode: K; collectionCode: Herbarium; ownerInstitutionCode: K; basisOfRecord: PreservedSpecimen

##### Distribution

Widespread

#### Sporobolus stapfianus

Gand.

##### Materials

- **Type status:** Other material. **Occurrence:** catalogNumber: K000984181; recordNumber: 214; recordedBy: Braun, HMH; **Taxon:** scientificName: *Sporobolus stapfianus* Gand.; kingdom: Plantae; family: Poaceae; genus: Sporobolus; specificEpithet: stapfianus; scientificNameAuthorship: Gand.; **Location:** continent: Africa; country: Tanzania; stateProvince: Arusha; county: Ngorongoro; locality: Ngorongoro; verbatimLocality: Between Lake Largaja and Naabi entrance; minimumElevationInMeters: 1500; decimalLatitude: -3; decimalLongitude: 35.033333; **Event:** eventDate: 1967-05-15; **Record Level:** institutionCode: K; collectionCode: Herbarium; ownerInstitutionCode: K; basisOfRecord: PreservedSpecimen

##### Distribution

Tropical Africa

#### Tetrapogon roxburghiana

(Schult.) P.M.Peterson

Chloris roxburghiana Schult

##### Materials

- **Type status:** Other material. **Occurrence:** catalogNumber: K000984207; recordNumber: 9851; recordedBy: Greenway, PJ; **Taxon:** scientificName: *Chloris roxburghiana* Schult.; kingdom: Plantae; family: Poaceae; genus: Chloris; specificEpithet: roxburghiana; scientificNameAuthorship: Schult.; **Location:** continent: Africa; country: Tanzania; stateProvince: Mara; county: Serengeti; locality: Seronera; verbatimLocality: Serengeti; minimumElevationInMeters: 1554; decimalLatitude: -2.45; decimalLongitude: 34.833333; **Event:** eventDate: 03/20/1961; **Record Level:** institutionCode: K; collectionCode: Herbarium; ownerInstitutionCode: K; basisOfRecord: PreservedSpecimen
- **Type status:** Other material. **Occurrence:** catalogNumber: K000984208; recordNumber: 59; recordedBy: Brooks, GP; **Taxon:** scientificName: *Chloris roxburghiana* Schult.; kingdom: Plantae; family: Poaceae; genus: Chloris; specificEpithet: roxburghiana; scientificNameAuthorship: Schult.; **Location:** continent: Africa; country: Tanzania; stateProvince: Mara; county: Serengeti; locality: Banagi Hill; minimumElevationInMeters: 1371; decimalLatitude: -2.3; decimalLongitude: 34.833333; **Event:** eventDate: 02/08/1954; **Record Level:** institutionCode: K; collectionCode: Herbarium; ownerInstitutionCode: K; basisOfRecord: PreservedSpecimen
- **Type status:** Other material. **Occurrence:** catalogNumber: K000984209; recordNumber: 353; recordedBy: Paulo, S; **Taxon:** scientificName: *Chloris roxburghiana* Schult.; kingdom: Plantae; family: Poaceae; genus: Chloris; specificEpithet: roxburghiana; scientificNameAuthorship: Schult.; **Location:** continent: Africa; country: Tanzania; stateProvince: Mara; county: Serengeti; locality: Seronera; verbatimLocality: rest camp; decimalLatitude: -2.45; decimalLongitude: 34.833333; **Event:** eventDate: 04/23/1958; **Record Level:** institutionCode: K; collectionCode: Herbarium; ownerInstitutionCode: K; basisOfRecord: PreservedSpecimen
- **Type status:** Other material. **Occurrence:** catalogNumber: DB1290; recordNumber: 168; recordedBy: Belsky, PJ; **Taxon:** scientificName: *Chloris roxburghiana* Schult.; kingdom: Plantae; family: Poaceae; genus: Chloris; specificEpithet: roxburghiana; scientificNameAuthorship: Schult.; **Location:** continent: Africa; country: Tanzania; stateProvince: Mara; county: Serengeti; locality: Serengeti Research Institute; verbatimLocality: Serengeti Research Institute Compound; decimalLatitude: -2.433333; decimalLongitude: 34.85; **Event:** eventDate: 05/11/1981; **Record Level:** institutionCode: SWRC; collectionCode: Herbarium; ownerInstitutionCode: SWRC; basisOfRecord: PreservedSpecimen
- **Type status:** Other material. **Occurrence:** catalogNumber: DB1291; recordNumber: s.n.; recordedBy: Unknown; **Taxon:** scientificName: *Chloris roxburghiana* Schult.; kingdom: Plantae; family: Poaceae; genus: Chloris; specificEpithet: roxburghiana; scientificNameAuthorship: Schult.; **Location:** continent: Africa; country: Tanzania; stateProvince: Mara; county: Serengeti; locality: Serengeti Research Institute; verbatimLocality: Serengeti Research Institute A; decimalLatitude: -2.433333; decimalLongitude: 34.85; **Event:** eventDate: 11/24/1972; **Record Level:** institutionCode: SWRC; collectionCode: Herbarium; ownerInstitutionCode: SWRC; basisOfRecord: PreservedSpecimen
- **Type status:** Other material. **Occurrence:** catalogNumber: 571; recordNumber: s.n.; recordedBy: Mboya, E; **Taxon:** scientificName: *Chloris roxburghiana* Schult.; kingdom: Plantae; family: Poaceae; genus: Chloris; specificEpithet: roxburghiana; scientificNameAuthorship: Schult.; **Location:** continent: Africa; country: Tanzania; stateProvince: Mara; county: Serengeti; locality: Serengeti Research Centre; verbatimLocality: T1. Serengeti Research Centre.; minimumElevationInMeters: 1550; decimalLatitude: -2.38; decimalLongitude: 34.85; **Event:** eventDate: 02/09/2004; **Record Level:** institutionCode: MO; collectionCode: Herbarium; ownerInstitutionCode: MO; basisOfRecord: PreservedSpecimen
- **Type status:** Other material. **Occurrence:** catalogNumber: DB1294; recordNumber: 9851; recordedBy: Greenway, PJ; **Taxon:** scientificName: *Chloris roxburghiana* Schult.; kingdom: Plantae; family: Poaceae; genus: Chloris; specificEpithet: roxburghiana; scientificNameAuthorship: Schult.; **Location:** continent: Africa; country: Tanzania; stateProvince: Mara; county: Serengeti; locality: Seronera; verbatimLocality: Serengeti; minimumElevationInMeters: 1554; decimalLatitude: -2.45; decimalLongitude: 34.833333; **Event:** eventDate: 03/20/1961; **Record Level:** institutionCode: SWRC; collectionCode: Herbarium; ownerInstitutionCode: SWRC; basisOfRecord: PreservedSpecimen
- **Type status:** Other material. **Occurrence:** catalogNumber: 1116; recordNumber: 747; recordedBy: Mboya, EI; Shombe, H; **Taxon:** scientificName: *Chloris roxburghiana* Schult.; kingdom: Plantae; family: Poaceae; genus: Chloris; specificEpithet: roxburghiana; scientificNameAuthorship: Schult.; **Location:** continent: Africa; country: Tanzania; stateProvince: Mara; county: Serengeti; locality: Serengeti Research Centre; minimumElevationInMeters: 1417; decimalLatitude: -2.433333; decimalLongitude: 34.85; **Event:** eventDate: 02/09/2004; **Record Level:** institutionCode: NHT; collectionCode: Herbarium; ownerInstitutionCode: NHT; basisOfRecord: PreservedSpecimen

##### Distribution

Tropical Africa & Asia

#### Themeda sp.

##### Materials

- **Type status:** Other material. **Occurrence:** catalogNumber: 728; recordNumber: 943; recordedBy: Ellemann, L; **Taxon:** scientificName: Themeda; kingdom: Plantae; family: Poaceae; genus: Themeda; **Location:** continent: Africa; country: Tanzania; stateProvince: Arusha; county: Ngorongoro; locality: Irmisigiyo; verbatimLocality: Ngorongoro Conservation Area, Irmisigiyo; minimumElevationInMeters: 2350; decimalLatitude: -3.2; decimalLongitude: 35.366; **Event:** eventDate: 1993-09-30; **Record Level:** institutionCode: AAU; collectionCode: Herbarium; ownerInstitutionCode: AAU; basisOfRecord: PreservedSpecimen

#### Themeda triandra

Forssk.

##### Materials

- **Type status:** Other material. **Occurrence:** catalogNumber: 311; recordNumber: 1045L; recordedBy: Brown, ES; **Taxon:** scientificName: *Themeda triandra* Forssk.; kingdom: Plantae; family: Poaceae; genus: Themeda; specificEpithet: triandra; scientificNameAuthorship: Forssk.; **Location:** continent: Africa; country: Tanzania; stateProvince: Shinyanga; locality: Lake Magadi; verbatimLocality: Serengeti, near lage Magadi.; minimumElevationInMeters: 1524; decimalLatitude: -2.633333; decimalLongitude: 34.9; **Event:** eventDate: 1905-05-18; **Record Level:** institutionCode: EA; collectionCode: Herbarium; ownerInstitutionCode: EA; basisOfRecord: PreservedSpecimen
- **Type status:** Other material. **Occurrence:** catalogNumber: 719; recordNumber: 699; recordedBy: Ellemann, L; **Taxon:** scientificName: *Themeda triandra* Forssk.; kingdom: Plantae; family: Poaceae; genus: Themeda; specificEpithet: triandra; scientificNameAuthorship: Forssk.; **Location:** continent: Africa; country: Tanzania; stateProvince: Arusha; county: Ngorongoro; locality: Endulen; verbatimLocality: Ngorongoro Conservation Area, Ndeyan 4 km North-west of Endulen Village.; minimumElevationInMeters: 1900; decimalLatitude: -3.2; decimalLongitude: 35.283; **Event:** eventDate: 1993-07-16; **Record Level:** institutionCode: AAU; collectionCode: Herbarium; ownerInstitutionCode: AAU; basisOfRecord: PreservedSpecimen
- **Type status:** Other material. **Occurrence:** catalogNumber: 720; recordNumber: 722; recordedBy: Ellemann, L; **Taxon:** scientificName: *Themeda triandra* Forssk.; kingdom: Plantae; family: Poaceae; genus: Themeda; specificEpithet: triandra; scientificNameAuthorship: Forssk.; **Location:** continent: Africa; country: Tanzania; stateProvince: Arusha; county: Ngorongoro; locality: Sendui; verbatimLocality: Ngorongoro Conservation Area Sendui.; minimumElevationInMeters: 2450; decimalLatitude: -2.916; decimalLongitude: 35.7; **Event:** eventDate: 1993-07-22; **Record Level:** institutionCode: AAU; collectionCode: Herbarium; ownerInstitutionCode: AAU; basisOfRecord: PreservedSpecimen
- **Type status:** Other material. **Occurrence:** catalogNumber: 721; recordNumber: 772; recordedBy: Ellemann, L; **Taxon:** scientificName: *Themeda triandra* Forssk.; kingdom: Plantae; family: Poaceae; genus: Themeda; specificEpithet: triandra; scientificNameAuthorship: Forssk.; **Location:** continent: Africa; country: Tanzania; stateProvince: Arusha; county: Ngorongoro; locality: Oloronyo; verbatimLocality: Ngorongoro Conservation Area, Oloronyo,; minimumElevationInMeters: 2700; decimalLatitude: -3.2; decimalLongitude: 35.35; **Event:** eventDate: 1993-08-05; **Record Level:** institutionCode: AAU; collectionCode: Herbarium; ownerInstitutionCode: AAU; basisOfRecord: PreservedSpecimen
- **Type status:** Other material. **Occurrence:** catalogNumber: 722; recordNumber: 918; recordedBy: Ellemann, L; **Taxon:** scientificName: *Themeda triandra* Forssk.; kingdom: Plantae; family: Poaceae; genus: Themeda; specificEpithet: triandra; scientificNameAuthorship: Forssk.; **Location:** continent: Africa; country: Tanzania; stateProvince: Arusha; county: Ngorongoro; locality: Olmekeke; verbatimLocality: Ngorongoro Conservation Area, Olmekeke.; minimumElevationInMeters: 2100; decimalLatitude: -3.166; decimalLongitude: 35.283; **Event:** eventDate: 1993-09-21; **Record Level:** institutionCode: AAU; collectionCode: Herbarium; ownerInstitutionCode: AAU; basisOfRecord: PreservedSpecimen
- **Type status:** Other material. **Occurrence:** catalogNumber: 723; recordNumber: 932; recordedBy: Ellemann, L; **Taxon:** scientificName: *Themeda triandra* Forssk.; kingdom: Plantae; family: Poaceae; genus: Themeda; specificEpithet: triandra; scientificNameAuthorship: Forssk.; **Location:** continent: Africa; country: Tanzania; stateProvince: Arusha; county: Ngorongoro; locality: Oltebesi; verbatimLocality: Ngorongoro Conservation Area, Oltebesi above Ndiyan village; minimumElevationInMeters: 2100; decimalLatitude: -3.166; decimalLongitude: 35.283; **Event:** eventDate: 1993-09-29; **Record Level:** institutionCode: AAU; collectionCode: Herbarium; ownerInstitutionCode: AAU; basisOfRecord: PreservedSpecimen
- **Type status:** Other material. **Occurrence:** catalogNumber: 724; recordNumber: s.n.; recordedBy: Mboya, E; **Taxon:** scientificName: *Themeda triandra* Forssk.; kingdom: Plantae; family: Poaceae; genus: Themeda; specificEpithet: triandra; scientificNameAuthorship: Forssk.; **Location:** continent: Africa; country: Tanzania; stateProvince: Mara; county: Serengeti; locality: Serengeti National Park; verbatimLocality: T1. Serengeti National Park. Ndabaka-Seronera Road.; minimumElevationInMeters: 1297; decimalLatitude: -2.28; decimalLongitude: 34.5; **Event:** eventDate: 2004-02-07; **Record Level:** institutionCode: MO; collectionCode: Herbarium; ownerInstitutionCode: MO; basisOfRecord: PreservedSpecimen
- **Type status:** Other material. **Occurrence:** catalogNumber: 725; recordNumber: 10; recordedBy: Metele, P; **Taxon:** scientificName: *Themeda triandra* Forssk.; kingdom: Plantae; family: Poaceae; genus: Themeda; specificEpithet: triandra; scientificNameAuthorship: Forssk.; **Location:** continent: Africa; country: Tanzania; stateProvince: Arusha; county: Ngorongoro; locality: Ngorongoro Crater; verbatimLocality: T2. Ngorongoro Crater, Misigiyo, 15.1 km from NCAA headquarters; minimumElevationInMeters: 2270; decimalLatitude: -3; decimalLongitude: 35; **Event:** eventDate: 1997-06-17; **Record Level:** institutionCode: MO; collectionCode: Herbarium; ownerInstitutionCode: MO; basisOfRecord: PreservedSpecimen
- **Type status:** Other material. **Occurrence:** catalogNumber: 726; recordNumber: 24273; recordedBy: Peterson, PM; Soreng, RJ; Romaschenko, K; Mbago, F; **Taxon:** scientificName: *Themeda triandra* Forssk.; kingdom: Plantae; family: Poaceae; genus: Themeda; specificEpithet: triandra; scientificNameAuthorship: Forssk.; **Location:** continent: Africa; country: Tanzania; stateProvince: Shinyanga; locality: Naabi Hill Gate; verbatimLocality: Serengeti National Park, at 12 km NW of Naabi Hill Gate.; minimumElevationInMeters: 1651; decimalLatitude: -2.73597; decimalLongitude: 34.95284; **Event:** eventDate: 2012-06-16; **Record Level:** institutionCode: US; collectionCode: Herbarium; ownerInstitutionCode: US; basisOfRecord: PreservedSpecimen
- **Type status:** Other material. **Occurrence:** catalogNumber: 727; recordNumber: 24310; recordedBy: Peterson, PM; Soreng, RJ; Romaschenko, K; Mbago, F; **Taxon:** scientificName: *Themeda triandra* Forssk.; kingdom: Plantae; family: Poaceae; genus: Themeda; specificEpithet: triandra; scientificNameAuthorship: Forssk.; **Location:** continent: Africa; country: Tanzania; stateProvince: Arusha; county: Ngorongoro; locality: Ngorongoro Crater; verbatimLocality: Ngorongoro Conservation Area, rim of Ngorongoro Crater (descent gate).; minimumElevationInMeters: 2168; decimalLatitude: -3.15462; decimalLongitude: 35.47717; **Event:** eventDate: 2012-06-19; **Record Level:** institutionCode: US; collectionCode: Herbarium; ownerInstitutionCode: US; basisOfRecord: PreservedSpecimen
- **Type status:** Other material. **Occurrence:** catalogNumber: K000748908; recordNumber: 293; recordedBy: Paulo, S; **Taxon:** scientificName: *Themeda triandra* Forssk.; kingdom: Plantae; family: Poaceae; genus: Themeda; specificEpithet: triandra; scientificNameAuthorship: Forssk.; **Location:** continent: Africa; country: Tanzania; stateProvince: Mara; county: Serengeti; locality: Seronera airdrome; verbatimLocality: Musoma district.; minimumElevationInMeters: 1524; decimalLatitude: -2.333333; decimalLongitude: 34.833333; **Event:** eventDate: 1958-04-18; **Record Level:** institutionCode: K; collectionCode: Herbarium; ownerInstitutionCode: K; basisOfRecord: PreservedSpecimen
- **Type status:** Other material. **Occurrence:** catalogNumber: K000748918; recordNumber: 9835; recordedBy: Greenway, PJ; **Taxon:** scientificName: *Themeda triandra* Forssk.; kingdom: Plantae; family: Poaceae; genus: Themeda; specificEpithet: triandra; scientificNameAuthorship: Forssk.; **Location:** continent: Africa; country: Tanzania; stateProvince: Mara; county: Serengeti; locality: Seronera; minimumElevationInMeters: 1554; decimalLatitude: -2.45; decimalLongitude: 34.833333; **Event:** eventDate: 1961-03-17; **Record Level:** institutionCode: K; collectionCode: Herbarium; ownerInstitutionCode: K; basisOfRecord: PreservedSpecimen
- **Type status:** Other material. **Occurrence:** catalogNumber: K000748930; recordNumber: 19343; recordedBy: Raynal, J; **Taxon:** scientificName: *Themeda triandra* Forssk.; kingdom: Plantae; family: Poaceae; genus: Themeda; specificEpithet: triandra; scientificNameAuthorship: Forssk.; **Location:** continent: Africa; country: Tanzania; stateProvince: Mara; county: Serengeti; locality: Lake Lagarja; verbatimLocality: Ngorongoro conservation area, Serengeti NP, southern edge of Lake Lagarka (=Ndutu); decimalLatitude: -3; decimalLongitude: 35.033333; **Event:** eventDate: 1977-10-01; **Record Level:** institutionCode: K; collectionCode: Herbarium; ownerInstitutionCode: K; basisOfRecord: PreservedSpecimen
- **Type status:** Other material. **Occurrence:** catalogNumber: K000748933; recordNumber: 971; recordedBy: Pole Evans, IB; Erens, J; **Taxon:** scientificName: *Themeda triandra* Forssk.; kingdom: Plantae; family: Poaceae; genus: Themeda; specificEpithet: triandra; scientificNameAuthorship: Forssk.; **Location:** continent: Africa; country: Tanzania; stateProvince: Arusha; county: Ngorongoro; locality: Ngorongoro Crater; verbatimLocality: On top ridge of Ngorongoro crater.; decimalLatitude: -3.166667; decimalLongitude: 35.583333; **Event:** eventDate: 1938-06-24; **Record Level:** institutionCode: K; collectionCode: Herbarium; ownerInstitutionCode: K; basisOfRecord: PreservedSpecimen
- **Type status:** Other material. **Occurrence:** catalogNumber: K000748955; recordNumber: 95; recordedBy: Moreau; Moreau; **Taxon:** scientificName: *Themeda triandra* Forssk.; kingdom: Plantae; family: Poaceae; genus: Themeda; specificEpithet: triandra; scientificNameAuthorship: Forssk.; **Location:** continent: Africa; country: Tanzania; stateProvince: Arusha; county: Ngorongoro; locality: Ngorongoro; minimumElevationInMeters: 2286; decimalLatitude: -3.166667; decimalLongitude: 35.583333; **Event:** eventDate: 1935-1; **Record Level:** institutionCode: K; collectionCode: Herbarium; ownerInstitutionCode: K; basisOfRecord: PreservedSpecimen
- **Type status:** Other material. **Occurrence:** catalogNumber: K000748909; recordNumber: 316; recordedBy: Paulo, S; **Taxon:** scientificName: *Themeda triandra* Forssk.; kingdom: Plantae; family: Poaceae; genus: Themeda; specificEpithet: triandra; scientificNameAuthorship: Forssk.; **Location:** continent: Africa; country: Tanzania; stateProvince: Arusha; county: Ngorongoro; locality: Ngorongoro Crater; decimalLatitude: -3.166667; decimalLongitude: 35.583333; **Event:** eventDate: 1958-04-19; **Record Level:** institutionCode: K; collectionCode: Herbarium; ownerInstitutionCode: K; basisOfRecord: PreservedSpecimen
- **Type status:** Other material. **Occurrence:** catalogNumber: K001087191; recordNumber: 944; recordedBy: Pole Evans, IB; Erens, J; **Taxon:** scientificName: *Themeda triandra* Forssk.; kingdom: Plantae; family: Poaceae; genus: Themeda; specificEpithet: triandra; scientificNameAuthorship: Forssk.; **Location:** continent: Africa; country: Tanzania; stateProvince: Arusha; county: Ngorongoro; locality: Ngorongoro Crater; verbatimLocality: Growing on plains at bottom of crater.; decimalLatitude: -3.166667; decimalLongitude: 35.583333; **Event:** eventDate: 1958-06-24; **Record Level:** institutionCode: K; collectionCode: Herbarium; ownerInstitutionCode: K; basisOfRecord: PreservedSpecimen
- **Type status:** Other material. **Occurrence:** catalogNumber: K001087192; recordNumber: 413; recordedBy: Clair-Thompson, GN; **Taxon:** scientificName: *Themeda triandra* Forssk.; kingdom: Plantae; family: Poaceae; genus: Themeda; specificEpithet: triandra; scientificNameAuthorship: Forssk.; **Location:** continent: Africa; country: Tanzania; stateProvince: Arusha; county: Ngorongoro; locality: Empakai Crater; verbatimLocality: Embagai; minimumElevationInMeters: 1524; maximumElevationInMeters: 1829; decimalLatitude: -2.933333; decimalLongitude: 35.816667; **Event:** eventDate: 1932-02-05; **Record Level:** institutionCode: K; collectionCode: Herbarium; ownerInstitutionCode: K; basisOfRecord: PreservedSpecimen
- **Type status:** Other material. **Occurrence:** catalogNumber: 1110; recordNumber: 10; recordedBy: Metele, P; **Taxon:** scientificName: *Themeda triandra* Forssk.; kingdom: Plantae; family: Poaceae; genus: Themeda; specificEpithet: triandra; scientificNameAuthorship: Forssk.; **Location:** continent: Africa; country: Tanzania; stateProvince: Arusha; county: Ngorongoro; locality: Ngorongoro Crater; verbatimLocality: T2. Ngorongoro Crater, Misigiyo, 15.1 km from NCAA headquarters; minimumElevationInMeters: 2270; decimalLatitude: -3; decimalLongitude: 35; **Event:** eventDate: 1997-06-17; **Record Level:** institutionCode: NHT; collectionCode: Herbarium; ownerInstitutionCode: NHT; basisOfRecord: PreservedSpecimen
- **Type status:** Other material. **Occurrence:** catalogNumber: 1157; recordNumber: 819C; recordedBy: Mollel, NP; Rusch, GM; Mwakalebe, G; **Taxon:** scientificName: *Themeda triandra* Forssk.; kingdom: Plantae; family: Poaceae; genus: Themeda; specificEpithet: triandra; scientificNameAuthorship: Forssk.; **Location:** continent: Africa; country: Tanzania; stateProvince: Mara; county: Serengeti; locality: Robanda village; verbatimLocality: Serengeti district, Robanda village, road to the water pool, CG01 plot, 36M 0386088, 9763754 UTM; minimumElevationInMeters: 1388; decimalLatitude: -2.083333; decimalLongitude: 34.666667; **Event:** eventDate: 2003-01-28; **Record Level:** institutionCode: NHT; collectionCode: Herbarium; ownerInstitutionCode: NHT; basisOfRecord: PreservedSpecimen

##### Distribution

Widespread

#### Trachypogon spicatus

(L.f.) Kuntze

##### Materials

- **Type status:** Other material. **Occurrence:** catalogNumber: K001087190; recordNumber: 10200; recordedBy: Greenway, PJ; **Taxon:** scientificName: *Trachypogon spicatus* (L.f.) Kuntze; kingdom: Plantae; family: Poaceae; genus: Trachypogon; specificEpithet: spicatus; scientificNameAuthorship: (L.f.) Kuntze; **Location:** continent: Africa; country: Tanzania; stateProvince: Mara; county: Serengeti; locality: Seronera-Klein's camp; verbatimLocality: Seronera to Klein's camp, mile 55.3; minimumElevationInMeters: 1768; decimalLatitude: -1.769167; decimalLongitude: 35.3975; **Event:** eventDate: 1961-05-17; **Record Level:** institutionCode: K; collectionCode: Herbarium; ownerInstitutionCode: K; basisOfRecord: PreservedSpecimen
- **Type status:** Other material. **Occurrence:** catalogNumber: K001087189; recordNumber: 10088; recordedBy: Greenway, PJ; **Taxon:** scientificName: *Trachypogon spicatus* (L.f.) Kuntze; kingdom: Plantae; family: Poaceae; genus: Trachypogon; specificEpithet: spicatus; scientificNameAuthorship: (L.f.) Kuntze; **Location:** continent: Africa; country: Tanzania; stateProvince: Mara; county: Serengeti; locality: Seronera-Karawera; verbatimLocality: Seronera to Karawera guard post, Mile 55.; minimumElevationInMeters: 1219; **Event:** eventDate: 1961-04-24; **Record Level:** institutionCode: K; collectionCode: Herbarium; ownerInstitutionCode: K; basisOfRecord: PreservedSpecimen

##### Distribution

Tropical Africa & Americas

#### Tragus sp.

##### Materials

- **Type status:** Other material. **Occurrence:** catalogNumber: 730; recordNumber: 24314; recordedBy: Peterson, PM; Soreng, RJ; Romaschenko, K; Mbago, F; **Taxon:** scientificName: Tragus; kingdom: Plantae; family: Poaceae; genus: Tragus; **Location:** continent: Africa; country: Tanzania; stateProvince: Arusha; county: Ngorongoro; locality: Lake Magadi; verbatimLocality: Ngorongoro Conservation Area, W side of Lake Magadi.; minimumElevationInMeters: 1739; decimalLatitude: -3.17713; decimalLongitude: 35.51548; **Event:** eventDate: 2012-06-19; **Record Level:** institutionCode: US; collectionCode: Herbarium; ownerInstitutionCode: US; basisOfRecord: PreservedSpecimen

#### Tragus berteronianus

Schult.

##### Materials

- **Type status:** Other material. **Occurrence:** catalogNumber: 729; recordNumber: s.n.; recordedBy: Mboya, E; **Taxon:** scientificName: *Tragus berteronianus* Schult.; kingdom: Plantae; family: Poaceae; genus: Tragus; specificEpithet: berteronianus; scientificNameAuthorship: Schult.; **Location:** continent: Africa; country: Tanzania; stateProvince: Mara; county: Serengeti; locality: Serengeti Research Centre; verbatimLocality: T1. Serengeti National Park. Serengeti Research Centre. Tawiri Hostel.; minimumElevationInMeters: 1551; decimalLatitude: -2.38; decimalLongitude: 34.85; **Event:** eventDate: 2004-03-04; **Record Level:** institutionCode: MO; collectionCode: Herbarium; ownerInstitutionCode: MO; basisOfRecord: PreservedSpecimen
- **Type status:** Other material. **Occurrence:** catalogNumber: K001087176; recordNumber: 9864; recordedBy: Greenway, PJ; **Taxon:** scientificName: *Tragus berteronianus* Schult.; kingdom: Plantae; family: Poaceae; genus: Tragus; specificEpithet: berteronianus; scientificNameAuthorship: Schult.; **Location:** continent: Africa; country: Tanzania; stateProvince: Mara; county: Serengeti; locality: Seronera; verbatimLocality: Serengeti; minimumElevationInMeters: 1554; decimalLatitude: -2.45; decimalLongitude: 34.833333; **Event:** eventDate: 1961-03-21; **Record Level:** institutionCode: K; collectionCode: Herbarium; ownerInstitutionCode: K; basisOfRecord: PreservedSpecimen
- **Type status:** Other material. **Occurrence:** catalogNumber: K001087177; recordNumber: 408; recordedBy: Paulo, S; **Taxon:** scientificName: *Tragus berteronianus* Schult.; kingdom: Plantae; family: Poaceae; genus: Tragus; specificEpithet: berteronianus; scientificNameAuthorship: Schult.; **Location:** continent: Africa; country: Tanzania; stateProvince: Arusha; county: Ngorongoro; locality: Salei Plain; verbatimLocality: West of Salei plain.; decimalLatitude: -2.833333; decimalLongitude: 35.666667; **Event:** eventDate: 1958-04-29; **Record Level:** institutionCode: K; collectionCode: Herbarium; ownerInstitutionCode: K; basisOfRecord: PreservedSpecimen
- **Type status:** Other material. **Occurrence:** catalogNumber: 1123; recordNumber: 817; recordedBy: Mollel, NP; Rusch, GM; Mwakalebe, G; **Taxon:** scientificName: *Tragus berteronianus* Schult.; kingdom: Plantae; family: Poaceae; genus: Tragus; specificEpithet: berteronianus; scientificNameAuthorship: Schult.; **Location:** continent: Africa; country: Tanzania; stateProvince: Mara; county: Serengeti; locality: Robanda village; verbatimLocality: Serengeti district, road to the water pool, CG01 plot. .36M, 0386088; 9763754 UTM.; minimumElevationInMeters: 1388; decimalLatitude: -2.083333; decimalLongitude: 34.666667; **Event:** eventDate: 2003-01-28; **Record Level:** institutionCode: NHT; collectionCode: Herbarium; ownerInstitutionCode: NHT; basisOfRecord: PreservedSpecimen

##### Distribution

Widespread

#### Tricholaena teneriffae

(L.f.) Link

##### Materials

- **Type status:** Other material. **Occurrence:** catalogNumber: K001087187; recordNumber: 6266; recordedBy: Newbould, JB; **Taxon:** scientificName: *Tricholaena teneriffae* (L.f.) Link; kingdom: Plantae; family: Poaceae; genus: Tricholaena; specificEpithet: teneriffae; scientificNameAuthorship: (L.f.) Link; **Location:** continent: Africa; country: Tanzania; stateProvince: Arusha; county: Ngorongoro; locality: Oldiang'arangar; verbatimLocality: East Serengeti; minimumElevationInMeters: 2134; decimalLatitude: -2.8; decimalLongitude: 35.416667; **Event:** eventDate: 1962-11-19; **Record Level:** institutionCode: K; collectionCode: Herbarium; ownerInstitutionCode: K; basisOfRecord: PreservedSpecimen
- **Type status:** Other material. **Occurrence:** catalogNumber: K001087186; recordNumber: 10697; recordedBy: Greenway, PJ; Kanuri; **Taxon:** scientificName: *Tricholaena teneriffae* (L.f.) Link; kingdom: Plantae; family: Poaceae; genus: Tricholaena; specificEpithet: teneriffae; scientificNameAuthorship: (L.f.) Link; **Location:** continent: Africa; country: Tanzania; stateProvince: Mara; county: Serengeti; locality: Serengeti Central Plains; verbatimLocality: Serengeti central plains, NE side.; minimumElevationInMeters: 1676; decimalLatitude: -2.333333; decimalLongitude: 34.833333; **Event:** eventDate: 1962-06-04; **Record Level:** institutionCode: K; collectionCode: Herbarium; ownerInstitutionCode: K; basisOfRecord: PreservedSpecimen
- **Type status:** Other material. **Occurrence:** catalogNumber: K001087185; recordNumber: 10904; recordedBy: Greenway, PJ; Turner, M; **Taxon:** scientificName: *Tricholaena teneriffae* (L.f.) Link; kingdom: Plantae; family: Poaceae; genus: Tricholaena; specificEpithet: teneriffae; scientificNameAuthorship: (L.f.) Link; **Location:** continent: Africa; country: Tanzania; stateProvince: Mara; county: Serengeti; locality: Tutiahi river; verbatimLocality: NW of Tutiahi river, near the Lake Magadi Track.; minimumElevationInMeters: 1402; **Event:** eventDate: 1962-12-22; **Record Level:** institutionCode: K; collectionCode: Herbarium; ownerInstitutionCode: K; basisOfRecord: PreservedSpecimen
- **Type status:** Other material. **Occurrence:** catalogNumber: K001087184; recordNumber: 6179; recordedBy: Newbould, JB; **Taxon:** scientificName: *Tricholaena teneriffae* (L.f.) Link; kingdom: Plantae; family: Poaceae; genus: Tricholaena; specificEpithet: teneriffae; scientificNameAuthorship: (L.f.) Link; **Location:** continent: Africa; country: Tanzania; stateProvince: Arusha; county: Ngorongoro; locality: Lemuta; verbatimLocality: East Serengeti; minimumElevationInMeters: 1707; decimalLatitude: -2.7; decimalLongitude: 35.266667; **Event:** eventDate: 1962-07-16; **Record Level:** institutionCode: K; collectionCode: Herbarium; ownerInstitutionCode: K; basisOfRecord: PreservedSpecimen
- **Type status:** Other material. **Occurrence:** catalogNumber: K001087183; recordNumber: 1631; recordedBy: Heady, HF; **Taxon:** scientificName: *Tricholaena teneriffae* (L.f.) Link; kingdom: Plantae; family: Poaceae; genus: Tricholaena; specificEpithet: teneriffae; scientificNameAuthorship: (L.f.) Link; **Location:** continent: Africa; country: Tanzania; stateProvince: Arusha; county: Ngorongoro; locality: Ngorongoro Crater; decimalLatitude: -3.166667; decimalLongitude: 35.583333; **Event:** eventDate: 1959-02-16; **Record Level:** institutionCode: K; collectionCode: Herbarium; ownerInstitutionCode: K; basisOfRecord: PreservedSpecimen
- **Type status:** Other material. **Occurrence:** catalogNumber: K001087188; recordNumber: 10718; recordedBy: Greenway, PJ; **Taxon:** scientificName: *Tricholaena teneriffae* (L.f.) Link; kingdom: Plantae; family: Poaceae; genus: Tricholaena; specificEpithet: teneriffae; scientificNameAuthorship: (L.f.) Link; **Location:** continent: Africa; country: Tanzania; stateProvince: Mara; county: Serengeti; locality: Seronera river; verbatimLocality: Headwaters of the Seronera river, Musoma district. On the northern side of the Serengeti Central Plains.; minimumElevationInMeters: 1463; decimalLatitude: -2.333333; decimalLongitude: 34.833333; **Event:** eventDate: 1962-06-13; **Record Level:** institutionCode: K; collectionCode: Herbarium; ownerInstitutionCode: K; basisOfRecord: PreservedSpecimen

##### Distribution

Africa, Asia & Europe

#### Trichoneura ciliata

(Peter) S.M.Phillips

##### Materials

- **Type status:** Other material. **Occurrence:** catalogNumber: K001087178; recordNumber: 10196; recordedBy: Greenway, PJ; **Taxon:** scientificName: *Trichoneura ciliata* (Peter) S.M.Phillips; kingdom: Plantae; family: Poaceae; genus: Trichoneura; specificEpithet: ciliata; scientificNameAuthorship: (Peter) S.M.Phillips; **Location:** continent: Africa; country: Tanzania; stateProvince: Mara; county: Serengeti; locality: Seronera-Klein's camp; verbatimLocality: Seronera to Klein's camp, Mile 55.; minimumElevationInMeters: 1768; decimalLatitude: -1.769167; decimalLongitude: 35.3975; **Event:** eventDate: 1961-05-17; **Record Level:** institutionCode: K; collectionCode: Herbarium; ownerInstitutionCode: K; basisOfRecord: PreservedSpecimen
- **Type status:** Other material. **Occurrence:** catalogNumber: 895; recordNumber: 10196; recordedBy: Greenway, PJ; **Taxon:** scientificName: *Trichoneura ciliata* (Peter) S.M.Phillips; kingdom: Plantae; family: Poaceae; genus: Trichoneura; specificEpithet: ciliata; scientificNameAuthorship: (Peter) S.M.Phillips; **Location:** continent: Africa; country: Tanzania; stateProvince: Mara; county: Serengeti; locality: Seronera-Klein's camp; verbatimLocality: Seronera to Klein's camp, Mile 55.; minimumElevationInMeters: 1768; decimalLatitude: -1.769167; decimalLongitude: 35.3975; **Event:** eventDate: 1961-05-17; **Record Level:** institutionCode: EA; collectionCode: Herbarium; ownerInstitutionCode: EA; basisOfRecord: PreservedSpecimen

##### Distribution

Tanzania, Ethiopia & Kenya

#### Tripogon minimus

(A.Rich.) Hochst. ex Steud.

##### Materials

- **Type status:** Other material. **Occurrence:** catalogNumber: 783; recordNumber: 322; recordedBy: Braun, HMH; **Taxon:** scientificName: *Tripogon minimus* (A.Rich.) Hochst. ex Steud.; kingdom: Plantae; family: Poaceae; genus: Tripogon; specificEpithet: minimus; scientificNameAuthorship: (A.Rich.) Hochst. ex Steud.; **Location:** continent: Africa; country: Tanzania; stateProvince: Mara; county: Serengeti; locality: Seronera; minimumElevationInMeters: 1500; decimalLatitude: -2.45; decimalLongitude: 35.833333; **Event:** eventDate: 1968-12-19; **Record Level:** institutionCode: EA; collectionCode: Herbarium; ownerInstitutionCode: EA; basisOfRecord: PreservedSpecimen
- **Type status:** Other material. **Occurrence:** catalogNumber: K001087180; recordNumber: 322; recordedBy: Braun, H; **Taxon:** scientificName: *Tripogon minimus* (A.Rich.) Hochst. ex Steud.; kingdom: Plantae; family: Poaceae; genus: Tripogon; specificEpithet: minimus; scientificNameAuthorship: (A.Rich.) Hochst. ex Steud.; **Location:** continent: Africa; country: Tanzania; stateProvince: Mara; county: Serengeti; locality: Seronera; minimumElevationInMeters: 1500; decimalLatitude: -2.45; decimalLongitude: 34.833333; **Event:** eventDate: 1968-12-19; **Record Level:** institutionCode: K; collectionCode: Herbarium; ownerInstitutionCode: K; basisOfRecord: PreservedSpecimen

##### Distribution

Tropical Africa

#### Urochloa brachyura

(Hack.) Stapf

Urochloa geniculata C.E.Hubb

##### Materials

- **Type status:** Other material. **Occurrence:** catalogNumber: 731; recordNumber: s.n.; recordedBy: Mboya, E; **Taxon:** scientificName: *Urochloa brachyura* (Hack.) Stapf; kingdom: Plantae; family: Poaceae; genus: Urochloa; specificEpithet: brachyura; scientificNameAuthorship: (Hack.) Stapf; **Location:** continent: Africa; country: Tanzania; stateProvince: Mara; county: Serengeti; locality: Seronera Lodge; verbatimLocality: T1. Area around Frankfurt Zoological Society Headquarters and Seronera Lodge.; minimumElevationInMeters: 1512; decimalLatitude: -2.44; decimalLongitude: 34.82; **Event:** eventDate: 2004-02-09; **Record Level:** institutionCode: MO; collectionCode: Herbarium; ownerInstitutionCode: MO; basisOfRecord: PreservedSpecimen
- **Type status:** Other material. **Occurrence:** catalogNumber: K001087181; recordNumber: 13344; recordedBy: Greenway, PJ; Kanuri; **Taxon:** scientificName: *Urochloa brachyura* (Hack.) Stapf; kingdom: Plantae; family: Poaceae; genus: Urochloa; specificEpithet: brachyura; scientificNameAuthorship: (Hack.) Stapf; **Location:** continent: Africa; country: Tanzania; stateProvince: Mara; county: Serengeti; locality: Togoro Plains; minimumElevationInMeters: 1463; decimalLatitude: -2.166667; decimalLongitude: 34.916667; **Event:** eventDate: 1968-02-28; **Record Level:** institutionCode: K; collectionCode: Herbarium; ownerInstitutionCode: K; basisOfRecord: PreservedSpecimen
- **Type status:** Other material. **Occurrence:** catalogNumber: K001087182; recordNumber: 198; recordedBy: Braun, H; **Taxon:** scientificName: *Urochloa brachyura* (Hack.) Stapf; kingdom: Plantae; family: Poaceae; genus: Urochloa; specificEpithet: brachyura; scientificNameAuthorship: (Hack.) Stapf; **Location:** continent: Africa; country: Tanzania; stateProvince: Mara; county: Serengeti; locality: Banagi Hill; verbatimLocality: Roadside south east of Banagi Hill; minimumElevationInMeters: 1400; decimalLatitude: -2.3; decimalLongitude: 34.833333; **Event:** eventDate: 1967-03-31; **Record Level:** institutionCode: K; collectionCode: Herbarium; ownerInstitutionCode: K; basisOfRecord: PreservedSpecimen
- **Type status:** Other material. **Occurrence:** catalogNumber: 1140; recordNumber: 934; recordedBy: Mollel, NP; Rusch, GM; Mwakalebe, G; **Taxon:** scientificName: *Urochloa brachyura* (Hack.) Stapf; kingdom: Plantae; family: Poaceae; genus: Urochloa; specificEpithet: brachyura; scientificNameAuthorship: (Hack.) Stapf; **Location:** continent: Africa; country: Tanzania; stateProvince: Mara; county: Serengeti; locality: Serengeti NP; verbatimLocality: Along the road to TAWIRI research centre, 36M, 0360699, 9732458 UTM.; minimumElevationInMeters: 1519; decimalLatitude: -2.25; decimalLongitude: 34.333333; **Event:** eventDate: 2003-02-02; **Record Level:** institutionCode: NHT; collectionCode: Herbarium; ownerInstitutionCode: NHT; basisOfRecord: PreservedSpecimen
- **Type status:** Other material. **Occurrence:** catalogNumber: 1164; recordNumber: 934; recordedBy: Mollel, NP; Rusch, GM; Mwakalebe, G; **Taxon:** scientificName: *Urochloa brachyura* (Hack.) Stapf; kingdom: Plantae; family: Poaceae; genus: Urochloa; specificEpithet: brachyura; scientificNameAuthorship: (Hack.) Stapf; **Location:** continent: Africa; country: Tanzania; stateProvince: Mara; county: Serengeti; locality: Serengeti NP; verbatimLocality: Along the road to TAWIRI research centre, 36M, 0360699, 9732458 UTM.; minimumElevationInMeters: 1519; decimalLatitude: -2.25; decimalLongitude: 34.333333; **Event:** eventDate: 2003-02-02; **Record Level:** institutionCode: NHT; collectionCode: Herbarium; ownerInstitutionCode: NHT; basisOfRecord: PreservedSpecimen

##### Distribution

Eastern & Southern Africa

#### Urochloa panicoides

P.Beauv.

##### Materials

- **Type status:** Other material. **Occurrence:** catalogNumber: K000984118; recordNumber: 5512; recordedBy: Newbould, JB; **Taxon:** scientificName: *Urochloa panicoides* P.Beauv.; kingdom: Plantae; family: Poaceae; genus: Urochloa; specificEpithet: panicoides; scientificNameAuthorship: P.Beauv.; **Location:** continent: Africa; country: Tanzania; stateProvince: Arusha; county: Ngorongoro; locality: Endulen; verbatimLocality: Ngorongoro Conservation Area; minimumElevationInMeters: 1829; decimalLatitude: -3.183333; decimalLongitude: 35.15; **Event:** eventDate: 1963-1; **Record Level:** institutionCode: K; collectionCode: Herbarium; ownerInstitutionCode: K; basisOfRecord: PreservedSpecimen

##### Distribution

Widespread

## Analysis

**Species**

In total 939 herbarium specimens, housed in six herbaria were digitised for the SER. These represented 200 species from 77 genera. The most frequently collected species was *Sporobolus ioclados* (Trin.) Nees with 30 specimens (Table 1). Twenty species are known from ten or more herbarium specimens. However 54 species in the SER are only known from one herbarium record. The most commonly collected genus was *Sporobolus*, followed by *Panicum* and *Pennisetum* (Table 2). Most grass species recorded from the SER have a widespread distribution across Tropical Africa with only one Tanzanian endemic species recorded, *Apochiton burtii* C.E.Hubb. One species has not been recorded in mainland Africa before (*Bothriochloa pertusa* (L.) A.Camus), and this could be a new introduction or a possible misidentification and needs further investigation. We have not been able to verify reported records of *Aristida pilgeri* Henrad and *Digitaria milanjiana* (Rendle) Stapf and these species are not included in the checklist.

**Collectors**

Gustav Albert Peter collected the first ever grass specimen from the Ngorongoro crater in July 1926 (the type of *Eragrostis polysperma*, now accepted as *E. cilianensis* (All.) Janch.). The first Serengeti National Park grass specimen was not collected until October 1947 by Alexey Bogdan (*Sporobolus fimbriatus* (Nees ex Trin.) Nees). In total there were 66 different principal collectors of herbarium specimens. However one collector, Peter Greenway collected 250 specimens from SER, 26% of the total (Table 3). Due to Greenway's collecting efforts along with John Newbould the 1960’s was the most prolific decade for collecting with over 350 specimens. Four type specimens were collected from SER, three with Greenway as the principal collector.

**Collecting intensity across SER**

**Figure 1.**
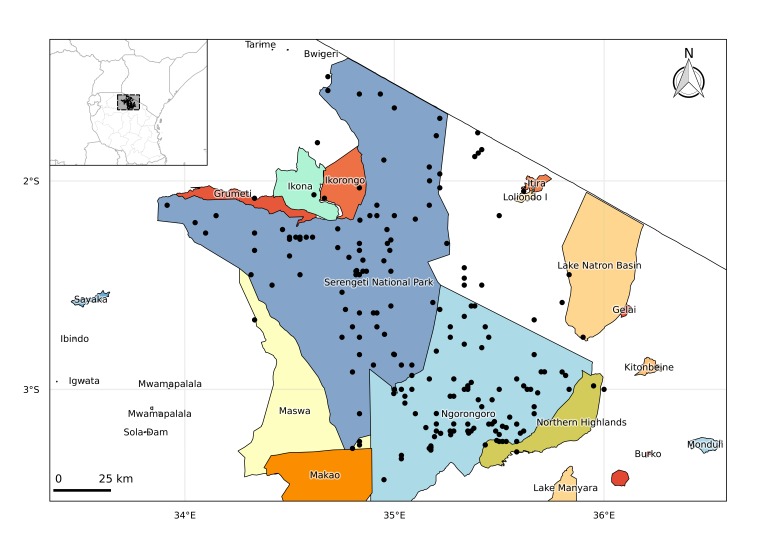
Distribution of grass specimens within the Serengeti Ecosystem Region

Specimens have been collected across the SER (Fig. 1) but there has been a collecting bias towards certain areas. For example 124 herbarium specimens have been collected in the area around the Serengeti Wildlife Research Centre and 103 specimens from the Ngorongoro Crater. The five smaller reserves (Maswa, Grumeti, Ikoma, Ikorongo and Loliondo) have few collections compared to the Serengeti National Park and the Ngorongoro Conservation Area.

## Discussion

The SER has a comparable number of grass species to other Tanzania National Parks with 200 species. The Selous Game Reserve checklist (Vollesen 1980) recorded 239 species of Poaceae and the Mkomazi National Park (Vollesen et al. 1999) recorded 123 species. There is also a similar low level of endemicity with only one Tanzanian endemic species found in the SER, one Tanzanian endemic in the Selous Game Reserve and no endemic species in the Mkomazi National Park. In this checklist over a quarter of species are only known from one herbarium specimen. Further research into these species is required, to determine whether they are genuinely rare or merely undercollected. The digitisation of grasses from East African herbaria, Kew and using GBIF records allowed us to find records of these undercollected species: if the checklist had been produced with only East African or only European herbarium specimens then some of these species would have been missed.

The checklist allows researchers to see which species have been commonly collected by botanists but there may be some bias. There is a strong bias in the places that botanists have collected with high numbers of specimens from near the Serengeti Wildlife Research Institute and at the Ngorongoro Crater but there also could be a bias towards collection of the most attractive or well known species.

## Supplementary Material

Supplementary material 1 Checklist of Serengeti Ecosystem Grasses Data type: Species occurences
Brief description: Checklist of species with species names including authorities, place of publication, global distribution and specimen citation (locality collected, principal collector, collector's number and herbaria duplicates).
File: oo_81713.pdf E.V.Williams, J. Elia Ntandu, P.Ficinski & M.Vorontsova

## Figures and Tables

**Table 1.** The top ten commonly collected species in the Serengeti Ecosystem Region

Species	Number of specimens
Sporobolus ioclados (Trin.) Nees	30
Cenchrus ciliaris L.	22
Chloris gayana Kunth	22
Eleusine jaegeri Pilg.	21
Aristida adoensis Hochst.	18
Aristida kenyensis Henrard	18
Themeda triandra Forssk.	18
Digitaria abyssinica (Hochst. ex A.Rich.) Stapf	17
Sporobolus fimbriatus (Nees ex Trin.) Nees	16
Panicum coloratum L.	14

**Table 2.** The top ten commonly collected genera in the Serengeti Ecosystem Region

Genus	Number of specimens
Sporobolus	119
Panicum	65
Pennisetum	59
Eragrostis	57
Aristida	47
Setaria	45
Digitaria	41
Chloris	39
Brachiaria	36
Eleusine	27

**Table 3.** Principal collectors with highest numbers of specimens collected

Principal collector	Years of collections	Number of specimens collected (% of total specimens)
Greenway, P.J.	1956-1968	250 (28%)
Ellemann, L.	1993-1994	79 (9%)
Peterson, P.M.	2012	70 (8%)
Newbould, J.B.	1962-1965	66 (7%)
Paulo, S.	1958	65 (7%)
