# Supplementary material for: Checklist of Serengeti Ecosystem Grasses
Source: Biodivers Data J. 2016 Apr 15;(4):e8286. doi: 10.3897/BDJ.4.e8286 (PMC4867701; doi:10.3897/BDJ.4.e8286)
Supplement: Supplementary material 1 — Checklist of Serengeti Ecosystem Grasses [file biodiversity_data_journal-4-e8286-s001.pdf]

CHECKLIST OF SERENGETI ECOSYSTEM GRASSES

BIODIVERSITY DATA JOURNAL – SUPPLEMENTARY FILE

AGROSTIS L.
***Agrostis kilimandscharica* Mez , Repert. Spec. Nov. Regni Veg. 18: 2 (1922)**
Distribution: Eastern & Central Africa
Specimens: Arusha Mokilal *Chuwa, S* 2757 (K); Empakai Crater *Frame, GW* 165 (K);

***Agrostis producta* Pilg. , Bot. Jahrb. Syst. 39: 600 (1907)**
Distribution: Tropical Africa
Specimens: Arusha Empakai Crater *Frame, GW* P 3 (EA,K); Olmoti crater *Vesey-FitzGerald, LDEF* 14 (?);

ALLOTEROPSIS J.Presl.
Alloteropsis cimicina* (L.) Stapf , Fl. Trop. Afr. 9: 487 (1919*)
Distribution: Tropical Africa & Asia
Specimens: Mara Seronera-Klein's camp *Greenway, PJ* 10197 (K);

ANDROPOGON L.
***Andropogon amethystinus* Steud. , Syn. Pl. Glumac. 1: 371 (1854)**
(Synonym: *Andropogon pratensis* Hochst. ex Hack.)
Distribution: Tropical Africa, Yemen, India & Myanmar
Specimens: Arusha Empakai Crater *Frame, GW* P 2 (EA,K,NHT); Empakai Crater *Frame, GW* P 7 (EA,K); Empakai Crater *Frame, GW* 96 (EA,K,NHT); Ngorongoro Crater *Pole Evans, IB* 976 (K); Empakai Crater *Terry, PJ* 1959 (EA,NHT);

***Andropogon chrysostachyus* Steud. , Syn. Pl. Glumac. 1: 377 (1854)**
Distribution: Tanzania, Kenya & Ethiopia
Specimens: Arusha Ngorongoro Crater *Chuwa, S* 2746 (K);

***Andropogon distachyos* L. , Sp. Pl. 2: 1046 (1753)**
Distribution: Europe, Arabia & Africa
Specimens: Arusha Ngorongoro Crater *Greenway, PJ* 11842 (K); Ngorongoro Crater *Newbould, JB* 5977 (K);

***Andropogon greenwayi* Napper , Kirkia 3: 121 (1963)**
Distribution: Tanzania, Kenya, Ethiopia, Somalia & Yemen
Specimens: Arusha Kakesio *Chuwa, S* 2784 (K); Olbalbal *Newbould, JB* 6108 (EA); Ngorongoro Crater *Paulo, S* 312 (EA,K); Ngorongoro Crater *Peterson, PM* 24307 (US); Ngorongoro *Robson, TO* 173 (EA,K); **Mara** Seronera *Greenway, PJ* 10330 (EA,K); Serengeti National Park *Kreulen, AR* 326 (EA); Serengeti *Paulo, S* 371 (EA,K); Lake Lagarja *Robson, TO* 207 (EA,K); Serengeti National Park *Vesey-FitzGerald, LDEF* 4692 (EA); **Shinyanga** Naabi Hill Gate *Belsky, JB* 259 (EA); Naabi Hill *Greenway, PJ* 10148 (EA,K); Naabi Hill *Greenway, PJ* 10165 (EA,K,PRE);

***Andropogon lima* (Hack.) Stapf , Fl. Trop. Afr. 9: 217 (1918)**
Distribution: Tropical Africa
Specimens: Arusha Lemagarut Mt. *Chuwa, S* 2674 (K); Oldeani Mt *Chuwa, S* 89004 T (K);

***Andropogon schirensis* Hochst. , Tent. Fl. Abyss. 2: 456 (1850)**
Distribution: Tropical & Southern Africa
Specimens: Mara Mara River Guard Post *Greenway, PJ* 10768 (K); Wogakuria Hill *Greenway, PJ* 12006 (K);

APOCHITON C.E.Hubb.
***Apochiton burttii* C.E.Hubb , Hooker's Icon. Pl. 34: t. 3319 (1936)**
Distribution: Tanzania endemic
Specimens: Arusha Ololgumi *Ellemann, L* 657 (AAU); Olduvai *Vesey-FitzGerald, LDEF* 4487 (SWRC); Olduvai *Vesey-FitzGerald, LDEF* 7266 (SWRC);

ARISTIDA L.
***Aristida adoensis* Hochst. , Tent. Fl. Abyss. 2: 390 (1850)**
Distribution: Eastern Africa
Specimens: Arusha Olbalbal *Kirika, P* 5925 (EA); Endulen *Kirika, P* 6449 (EA); Olbalbal *Newbould, JB* 5925 (EA); Endulen *Newbould, JB* 6449 (EA); Ngorongoro *Vesey-FitzGerald, LDEF* 12 (SWRC); **Mara** Lobo Lodge *Belsky, PJ* 285 (SWRC); Seronera *Greenway, PJ* 9902 (EA,K,SWRC); Seronera *Greenway, PJ* 10183 (EA,K,SWRC); Serengeti *Herlocker, D* 601 A (SWRC); Serengeti National Park *Kirika, P* 363 (EA); Serengeti National Park *Kirika, P* 5599 (EA); Banagi *Leippert, H* 5599 (EA,K); Seronera *Ludanga, RI* R 56 (EA); Seronera *Schmidt, W* 109 (SWRC); **Shinyanga** Lake Magadi *Greenway, PJ* 10050 (EA,SWRC); Granite Kopje *Kirika, P* 6561 (EA); Granite Kopje *Newbould, JB* 6561 (EA); Lake Magadi *Paulo, S* 368 (K);

***Aristida adscensionis* L. , Sp. Pl. 1: 82 (1753)**
Distribution: Widespread
Specimens: Arusha Olduvai *Chuwa, S* 2613 (K); Ngorongoro Crater *Chuwa, S* 2798 (K); Olbalbal *Kirika, P* 6026 (EA); **Mara** Serengeti Research Centre *Mboya, E* s.n. (MO);

***Aristida barbicollis* Trin. & Rupr. , Sp. Gram. Stipac.: 152 (1842)**
Distribution: Eastern & Southern Africa
Specimens: Mara Seronera *Greenway, PJ* 10303 (EA,K,SWRC); Serengeti *Greenway, PJ* 13324 (SWRC);

***Aristida hordeacea* Kunth , Révis. Gramin. 2: t. 173 (1831)**
Distribution: Tropical & Southern Africa
Specimens: Mara Seronera *Greenway, PJ* 10089 (EA,K,SWRC); Serengeti *Paulo, S* 443 (EA,K); Serengeti National Park *Suleiman, HO* 1077 (SWRC);

***Aristida kenyensis* Henrard , Meded. Rijks-Herb. 54C: 722 (1933)**
Distribution: Eastern Africa
Specimens: Arusha Olduvai *Belsky, PJ* 120 (SWRC); Lemuta hill *Newbould, JB* 163 (EA); Olbalbal *Newbould, JB* 5925 (EA); Olbalbal *Newbould, JB* 6026 (EA); Lemuta hill *Oteke, J* 193 (EA,K); Olbalbal *Paulo, S* 326 (EA,K); Lake Magadi *Peterson, PM* 24316 (US); **Mara** Ndutu Lodge *Belsky, PJ* 68 (SWRC); Seronera *Greenway, PJ* 9091 (EA,SWRC); Seronera *Greenway, PJ* 9901 (EA,K); Seronera *Greenway, PJ* 9973 (EA,K,SWRC); Seronera *Greenway, PJ* 10188 (EA,K,SWRC); Musoma *Greenway, PJ* 10327 (EA,SWRC); Serengeti Plains *Greenway, PJ* 11181 (EA); Seronera *Paulo, S* 354 (EA,K); Lobo Lodge *Peterson, PM* 24297 (US); Simba Kopjes *Schmidt, W* 186 (SWRC); **Shinyanga** Lake Magadi *Greenway, PJ* 10051 (EA,K,SWRC);

***Aristida mutabilis* Trin. & Rupr. , Sp. Gram. Stipac. 150 (1842)**
Distribution: Tropical Africa & Asia
Specimens: Arusha Ol Doinyo Lengai *Greenway, PJ* 11349 (EA,K);

Aristida sp.
Specimens: Mara Serengeti National Park, near Mbuzi Mare camp *Peterson, PM* 24279 (US);

BOTHRIOCHLOA Kuntze
***Bothriochloa bladhii* (Retz.) S.T.Blake , Proc. Roy. Soc. Queensland 80: 62 (1969)**
(Synonym: *Bothriochloa bladhii* (Retz.) S.T.Blake var. *bladhii*)
Distribution: Widespread
Specimens: Mara Grumeti River *Greenway, PJ* 13167 (K,SWRC);

***Bothriochloa insculpta (*Hochst. ex A.Rich.) A.Camus , Ann. Soc. Linn. Lyon, n.s., 76: 165 (1931)**
Distribution: Tropical Africa, Southern Africa, Arabia & Asia
Specimens: Arusha Endulen *Ellemann, L* 373 (AAU); Endulen *Ellemann, L* 375 (AAU); **Mara** Serengeti Research Institute *Belsky, PJ* 173 (SWRC); Serengeti Research Institute *Belsky, PJ* 194 (SWRC); Seronera *Paulo, S* 355 (K); Banagi Hill *Paulo, S* 365 (K); **Shinyanga** Lake Magadi *Greenway, PJ* 10048 (K,SWRC); Naabi Hill Gate *Peterson, PM* 24274 (US);

***Bothriochloa pertusa* (L.) A.Camus , Ann. Soc. Linn. Lyon, n.s., 76: 164 (1931)**
Distribution: Asia & Pacific, not previously recorded for East Africa
Specimens: Mara Seronera *Schmidt, W* 102 (SWRC); Simba Kopjes *Schmidt, W* 273 (SWRC); Bolgonja *Schmidt, W* 439 (SWRC);

***Bothriochloa radicans* (Lehm.) A.Camus , Ann. Soc. Linn. Lyon, n.s., 76: 164 (1931)**
Distribution: Eastern & Southern Africa, Arabia,
Specimens: Mara Serengeti Research Institute *Belsky, PJ* 2 (EA,SWRC); Seronera *Greenway, PJ* 9838 (EA,K,SWRC); Ndabaka *Greenway, PJ* 10378 (EA,K,SWRC); Seronera plain *Mbano, BNN* CAWM 5549 (EA); Simba Kopjes *Schmidt, W* 39 (SWRC);

BRACHIARIA (Trin.) Griseb.
***Brachiaria ambigens* Chiov. , Webbia 8: 62 (1951)**
Distribution: Tanzania, Kenya & Ethiopia
Specimens: Arusha Olbalbal *Newbould, JB* 6361 (EA,K);

***Brachiaria arrecta* (T.Durand & Schinz) Stent , Bothalia 1: 263 (1924)**
Distribution: Eastern & Southern Africa
Specimens: Mara Serengeti National Park *Mbano, BNN* CAWM 5525 (K); **Shinyanga** Naabi Hill Gate *Peterson, PM* 24275 (US);

***Brachiaria bovonei* (Chiov.) Robyns , Bull. Jard. Bot. État Bruxelles 9: 174 (1932)**
Distribution: Eastern & Southern Africa
Specimens: Mara Tabora Air Strip *Greenway, PJ* 10915 (K);

***Brachiaria brizantha* (A.Rich.) Stapf , Fl. Trop. Afr. 9: 531 (1919)**
Distribution: Tropical & Southern Africa, introduced elsewhere in the tropics
Specimens: Mara Bolgonja river *Greenway, PJ* 10749 (K,SWRC); Bolgonja *Schmidt, W* 690 (SWRC);

***Brachiaria dictyoneura* (Fig. & De Not.) Stapf , Fl. Trop. Afr. 9: 512 (1919)**
(Synonym: *Urochloa dictyoneura* (Fig. & De Not.) Veldkamp)
Distribution: Eastern & Southern Africa, introduced elsewhere
Specimens: Mara Lobo Lodge *Belsky, PJ* 281 (SWRC); Seronera *Greenway, PJ* 10115 (K,SWRC); Serengeti National Park *Greenway, PJ* 10669 (SWRC); Mara River Guard Post *Greenway, PJ* 10771 (K,SWRC); Mbuzi Mare camp *Peterson, PM* 24284 (US); Bolgonja *Schmidt, W* 328 (SWRC);

***Brachiaria eruciformis* (Sm.) Griseb. , Fl. Ross. 4: 469 (1853)**
Distribution: Widespread in Europe, Arabia, Africa & Asia
Specimens: Arusha Esere *Chuwa, S* 2587 (K); **Mara** Seronera *Greenway, PJ* 9858 (SWRC); Seronera *Greenway, PJ* 10334 (K,SWRC); Serengeti Plains *Schmidt, W* 212 (SWRC);

***Brachiaria leersioides* (Hochst.) Stapf , Fl. Trop. Afr. 9: 551 (1919)**
Distribution: Eastern Africa & Arabia
Specimens: Arusha Engaruka *Vesey-FitzGerald, LDEF* 6577 (SWRC); **Mara** Serengeti Research Institute *Belsky, PJ* 146 (SWRC);

***Brachiaria rugulosa* Stapf , Fl. Trop. Afr. 9: 529 (1919)**
Distribution: Tanzania, Kenya, Zambia & Zimbabwe
Specimens: Mara Simiyu *Kreulen, AR* 325 (SWRC); **Shinyanga** Naabi Hill *Greenway, PJ* 10645 (SWRC);

***Brachiaria scalaris* (Mez.) Pilg. , Notizbl. Bot. Gart. Berlin-Dahlem 10: 269 (1928)**
Distribution: Tropical Africa & Arabia
Specimens: Arusha Ngorongoro Crater *Newbould, JB* 5952 (EA);

***Brachiaria semiundulata* (Hochst. ex A.Rich.) Stapf , Fl. Trop. Afr. 9: 556 (1919)**
Distribution: Eastern Africa & Asia
Specimens: Arusha Mokilal *Chuwa, S* 2753 (K); Ngorongoro *Newbould, JB* 5856 (EA); **Mara** Downeys Dam

Braun, HMH 249 (SWRC); Robanda village *Mollel, NP* 855 (NHT); Robanda village *Mollel, NP* 885 (NHT); Simba Kopjes *Schmidt, W* 15 (SWRC); Seronera *Schmidt, W* 87 (SWRC); **Shinyanga** Moru Kopjes *Greenway, PJ* 13179 (K,SWRC); Naabi Hill Gate *Vesey-FitzGerald, LDEF* 6591 (SWRC);

Brachiaria serrifolia **(Hochst.) Stapf , Fl. Trop. Afr. 9: 548 (1919)**
Distribution: Tropical Africa
Specimens: Arusha Olongogo *Newbould, JB* 6380 (EA,K);

Brachiaria sp.
Specimens: Arusha Olbalbal swamp *Ellemann, L.* 1100 (AAU); Olbalbal swamp *Ellemann, L.* 1101 (AAU);

Brachiaria subulifolia **(Mez) Clayton , Kew Bull. 34: 559 (1980)**
Distribution: Eastern & Southern Africa
Specimens: Mara Bolgonja *Schmidt, W* 578 (SWRC);

Brachiaria xantholeuca **(Hack.) Stapf , Fl. Trop. Afr. 9: 541 (1919)**
Distribution: Tropical Africa & Arabia
Specimens: Mara Serengeti Research Institute *Belsky, PJ* 186 (SWRC); Seronera *Braun, HMH* 250 (SWRC,WAG);

BRACHYPODIUM P.Beauv.
Brachypodium flexum **Nees , Fl. Afr. Austral. Ill. 456 (1841)**
Distribution: Tropical Africa
Specimens: Arusha Empakai Crater *Frame, GW* 504 (SWRC);

BROMUS L.
Bromus leptoclados **Nees , Fl. Afr. Austral. Ill.: 453 (1841)**
Distribution: Eastern & Southern Africa
Specimens: Arusha Olmoti crater *Burtt, BD* 4362 (K); Oloronyo forest *Ellemann, L* 768 (AAU); Empakai Crater *Frame, GW* P 58 (K); Ngorongoro Crater *Greenway, PJ* 3363 (K); Empakai Crater *Greenway, PJ* 9131 (K); Ngorongoro *Staples, RR* 283 (K);

CALAMAGROSTIS Adans.
Calamagrostis epigejos **(L.) Roth , Tent. Fl. Germ. 1: 34 (1788)**
Distribution: Widespread
Specimens: Arusha Empakai Crater *Frame, GW* 167 (EA,SWRC);

CENCHRUS L.
Cenchrus abyssinicus **(Hack.) Morrone , Ann. Bot. (Oxford) 106: 127 (2010)**
(Synonym: *Odontelytrum abyssinicum* Hack.)
Distribution: Eastern Africa & Arabia
Specimens: Arusha Ngorongoro Crater *Goddard, J* 118 (?); Ngorongoro Crater *Greenway, PJ* 12617 (K);

Cenchrus ciliaris **L. , Mant. Pl. 2: 302 (1771)**
Distribution: Widespread
Specimens: Arusha Ntudu Lodge *Belsky, PJ* 69 (SWRC); Olduvai *Belsky, PJ* 116 (SWRC); Olduvai Gorge *Goddard, J* 253 (EA); Olduvai *Leakey, MD* 19306 (K); Loliondo *Macha* 1111 (NHT); Olbalbal *Newbould, JB* 5725 (EA); Olbalbal *Newbould, JB* 5931 (EA); Gol Kopjes *Schmidt, W* 250 (SWRC); Empakai Mt *Thompson, IH* 344 (K); **Mara** Serengeti Research Institute *Belsky, PJ* 1 (SWRC); Serengeti Research Institute *Belsky, PJ* 165 (SWRC); Serengeti Research Institute *Bonnefille, R* 73/46 (EA); Banagi *Brown, ES* 2143 B (EA); Seronera *Greenway, PJ* 9856 (EA,K,SWRC); Seronera *Greenway, PJ* 9955 (EA,K,SWRC); Simba Kopjes *Harris, BJ* 5454 (SWRC); Seronera camp *Jacob, J* 12 (EA); Serengeti National Park *Mboya, E* s.n. (MO); Robanda village *Mollel, NP* 883 (NHT); Serengeti *Paulo, S* 336 (EA,K); Serengeti *Paulo, S* 398 (EA); Mbuzi Mare camp *Peterson, PM* 24281 (US); Bolgonja *Schmidt, W* 416 (SWRC); Lake Lagarja *Vesey-FitzGerald, LDEF* 4464 (EA,SWRC);

CHLORIS Sw.
Chloris gayana Kunth , Révis. Gramin. 1: 89 (1829)
Distribution: Widespread, native to Tropical Africa

Specimens: Arusha Endulen *Ellemann, L* 372 (AAU); Endulen *Ellemann, L* 376 (AAU); Endulen *Ellemann, L* 703 (AAU); Endulen *Ellemann, L* 714 (AAU); Oloronyo forest *Ellemann, L* 766 (AAU); Nasiporiong *Ellemann, L* 819 (AAU); Masek *Ellemann, L* 923 (AAU); Irmisigiyo *Ellemann, L* 942 (AAU); Empakai Crater *Frame, GW* 531 (SWRC); Ngorongoro Crater *Macha* 1048 (NHT); Laetoli valley *Mollel, NP* 1186 (NCAA,NHT); Ngorongoro *Newbould, JB* 5650 (K); Ngorongoro Crater *Paulo, S* 317 (K); Olduvai *Paulo, S* 681 (K); Ngorongoro *Vesey-FitzGerald, LDEF* 130 (SWRC); **Mara** Serengeti Research Institute *Belsky, PJ* 164 (SWRC); Ikoma *Brooks, GP* 34 (K); Seronera river *Greenway, PJ* 9969 (K); Seronera Lodge *Mboya, E* s.n. (MO); Serengeti Central Plains *Mboya, E* s.n. (MO); Serengeti NP *Mollel, NP* 935 (NHT); Engare Nanyuki *Newbould, JB* 6302 (K); Bolgonja *Schmidt, W* 575 (SWRC); Bolgonja *Schmidt, W* 656 (SWRC); Lake Lagarja *Vesey-FitzGerald, LDEF* 4462 (SWRC);

***Chloris pycnothrix* Trin. , Gram. Unifl. Sesquifl.: 234 (1824)**
Distribution: Widespread
Specimens: Arusha Endulen *Ellemann, L* 366 (AAU); Endulen *Ellemann, L* 706 (AAU); Nasiporiong *Ellemann, L* 814 (AAU); Olbalbal *Ellemann, L* 882 (AAU); Ngorongoro Crater *Paulo, S* 303 (K); Olbalbal *Paulo, S* 329 (K); **Mara** Ndutu Lodge *Belsky, PJ* 50 (SWRC); Serengeti Research Institute *Belsky, PJ* 192 (SWRC); Seronera *Greenway, PJ* 9874 (K,SWRC); Serengeti *Paulo, S* 440 (K);

Chloris sp.
Specimens: Arusha Ngorongoro Crater *Peterson, P.M.* 24305 (US); **Mara** Mbuzi Mare camp *Peterson, P.M.* 24286 (US); Lobo Lodge *Peterson, P.M.* 24296 (US); Shinyanga Naabi Hill Gate *Peterson, P.M.* 24262 (US);

***Chloris virgata* Sw. , Fl. Ind. Occid. 1: 203 (1797)**
Distribution: Widespread
Specimens: Mara Serengeti Research Institute *Belsky, PJ* 204 (SWRC); Seronera *Greenway, PJ* 9863 (K,SWRC); Serengeti National Park *Mboya, E* s.n. (MO);

CHRYSOCHLOA Swallen
***Chrysochloa orientalis* (C.E.Hubb) Swallen , Proc. Biol. Soc. Wash. 54: 44 (1941)**
Distribution: Tanzania, Kenya, Uganda & Democratic Republic of Congo
Specimens: Mara Serengeti Research Institute *Belsky, PJ* 219 (SWRC); Seronera *Greenway, PJ* 9873 (K); Endarbark *Paulo, S* 438 (K); Serengeti Plains *Schmidt, W* 216 (SWRC); Serengeti National Park *Suleiman, HO* 2004 (DSM,SWRC,SWRC); Grumeti Plains *Vesey-FitzGerald, LDEF* 4655 (SWRC);

CHRYSOPOGON Trin.
***Chrysopogon aucheri* (Boiss.) Stapf , Bull. Misc. Inform. Kew 1907: 211 (1907)**
Distribution: Eastern Africa & Arabia
Chrysopogon plumulosus Hochst. , Jahresh. Vereins Vaterl. Naturk. Württemberg 3: 62 (1847)
(Synonym: *Chrysopogon aucheri* (Boiss.) Stapf var. *quinqueplumis* (A.Rich) Stapf)
Distribution: Tropical Africa & Arabia
Specimens: Arusha Olkarien *Newbould, JB* 6396 (K); Lemuta *Oteke, J* 251 (K); **Mara** Lake Lagarja *Tanner, M* EAH 12695 (K);

CTENIUM Panz.
***Ctenium somalense* (Chiov.) Chiov. , Nuovo Giorn. Bot. Ital., n.s., 26: 82 (1919)**
(Synonym: *Ctenium concinnum* Nees var. *minus* Pilg.)
Distribution: Tropical Africa
Specimens: Mara Mara River Guard Post *Greenway, PJ* 10767 (K);

CYMBOPOGON Spreng.
***Cymbopogon caesius* (Hook. & Arn.) Stapf , Bull. Misc. Inform. Kew 1906: 341 (1906)**
(Synonym: *Cymbopogon excavatus* (Hochst.) Stapf ex Burtt Davy)
Distribution: Tropical Africa & Asia
Specimens: Arusha Engitati Hill *Greenway, PJ* 12607 (K); **Mara** Ikoma *Brooks, GP* 39 (K); Tabora *Greenway, PJ* 10129 (K); Seronera *Greenway, PJ* 10186 (K); **Shinyanga** Lake Magadi *Greenway, PJ* 10046 (K);

***Cymbopogon pospischilii* (K.Schum.) C.E.Hubb , Kew Bull. 4: 175 (1949)**
Distribution: Tropical Africa & Asia
Specimens: Mara Serengeti National Park *Herlocker, D* 2 (K); **Shinyanga** Simiyu river *Greenway, PJ* 10380 (K);

Cymbopogon sp.
Specimens: Mara Serengeti NP *Mollel, N.P.* 917 (NHT);

CYNODON Rich.
***Cynodon aethiopicus* Clayton & Harlan , Kew Bull. 24: 187 (1970)**
Distribution: Eastern & Southern Africa
Specimens: Arusha Endulen *Ellemann, L* 446 (AAU); Enja Shori *Ellemann, L* 708 (AAU);

***Cynodon dactylon* (L.) Pers. , Syn. Pl. 1: 85 (1805)**
Distribution: Widespread
Specimens: Arusha Olbalbal *Paulo, S* 321 (K); Salei Plain *Paulo, S* 383 (K); **Mara** Engare Nanyuki *Newbould, JB* 6315 (K);

***Cynodon nlemfuensis* Vanderyst , Bull. Agric. Congo Belge 13: 342 (1922)**
Distribution: Tropical Africa, introduced elsewhere
Specimens: Arusha Ngorongoro Crater *Raynal, J* 19178 (K); Olduvai *Raynal, J* 19305 (K);

Cynodon nlemfuensis* Vanderyst *var. nlemfuensis
Distribution: Tropical Africa, introduced elsewhere
Specimens: Arusha Olduvai Gorge *Chuwa, S* 2791 (K); Gol Kopjes *Chuwa, S* 2867 (K); Ngorongoro Crater *Frame, GW* 80 (K); Empakai Crater *Frame, GW* 95 (K); Lemuta hill *Paulo, S* 350 (K); **Mara** Seronera *Greenway, PJ* 9859 (K); Engare Nanyuki *Paulo, S* 360 (K);

***Cynodon nlemfuensis* Vanderyst *var. robustus* Clayton & J.R.Harlan , Kew Bull. 24: 189 (1970)**
Distribution: Tropical Africa
Specimens: Mara Seronera *Paulo, S* 300 (K);

***Cynodon plectostachyus* (K.Schum.) Pilg. , Bot. Jahrb. Syst. 40: 82 (1907)**
Distribution: Eastern Africa, introduced elsewhere
Specimens: Arusha Olduvai Gorge *Chuwa, S* 2790 (K); Engaruka *Leippert* 6356 (K); Olbalbal *Paulo, S* 410 (K); **Mara** Seronera *Greenway, PJ* 9855 (K); Seronera *Greenway, PJ* 10190 (K); Seronera Lodge *Mboya, E* s.n. (MO);

Cynodon sp.
Specimens: Arusha Endulen *Ellemann, L.* 452 (AAU); Lake Madagi *Peterson, P.M.* 24318 (US); **Shinyanga** Naabi Hill Gate *Peterson, P.M.* 24259 (US); Naabi Hill Gate *Peterson, P.M.* 24268 (US); Naabi Hill Gate *Peterson, P.M.* 24270 (US);

DACTYLOCTENIUM Willd.
***Dactyloctenium aegyptium* (L.) Willd. , Enum. Pl.: 1029 (1809)**
Distribution: Widespread
Specimens: Mara Seronera Lodge *Mboya, E* s.n. (MO);

***Dactyloctenium geminatum* Hack. , Bull. Herb. Boissier 7: 26 (1899)**
(Synonym: *Dactyloctenium bogdanii* S.M.Phillips)
Distribution: Eastern & Southern Africa
Specimens: Arusha Orkuman *Ellemann, L* 487 (AAU); Enja Shori *Ellemann, L* 707 (AAU); Oldonyo-o-ogol *Ellemann, L* 1128 (AAU);

Dactyloctenium sp.
Specimens: Arusha Ngorongoro Crater *Peterson, P.M.* 24306 (US);

DESCHAMPSIA P.Beauv.
***Deschampsia flexuosa* (L.) Trin. , Bull. Sci. Acad. Imp. Sci. Saint-Pétersbourg 1: 66 1836**
(Synonym: *Aira caryophyllaea* Leers)
Distribution: Widespread
Specimens: Arusha Oldeani Mt *Newbould, JB* 6097 (?);

DICHANTHIUM Willemet
***Dichanthium annulatum* (Forssk.) Stapf , Fl. Trop. Afr. 9: 178 (1917)**
Distribution: Widespread
Specimens: Mara Serengeti National Park *Mboya, E* s.n. (MO);

Dichanthium annulatum* (Forssk.) Stapf *var. annulatum
Distribution: Widespread
Specimens: Mara Robanda village *Mollel, NP* 819 B (NHT);

DISAKISPERMA Steud.
***Disakisperma yemenicum* (Schweinf.) P.M.Peterson & N.Snow, PhytoKeys 26: 63 (2013)**
(Synonym: *Coelachyrum yemenicum* (Schweinf.) S.M.Phillips, *Cypholepis yemenica* (Schweinf.) Chiov.)
Distribution: Arabia, Eastern & Southern Africa
Specimens: Arusha Olduvai Gorge *Chuwa, S* 2608 (K); Olduvai Gorge *Chuwa, S* 2857 (K); Engaruka *Leippert* 6362 (K); Oldiang'arangar *Newbould, JB* 6543 (K); Engaruka *Richards, M* 25551 (K); **Mara** Serengeti *Banyikwa, FF* 2 (NHT); Serengeti *Greenway, PJ* 10678 (K); Engare Nanyuki springs *Greenway, PJ* 10694 (K); **Shinyanga** Naabi Hill Gate *Peterson, PM* 24254 (US);

DIGITARIA Haller
***Digitaria abyssinica* (Hochst. ex A.Rich.) Stapf , Bull. Misc. Inform. Kew 1907: 213 (1907)**
(Synonym: *Digitaria scalarum* (Schweinf.) Chiov.)
Distribution: Tropical Africa & Arabia
Specimens: Arusha Endulen *Ellemann, L* 370 (AAU); Empakai Crater *Frame, GW* 147 (EA,K); Empakai Crater *Frame, GW* 510 (EA); Empakai Crater *Frame, GW* 535 (EA); Ngorongoro Crater *Gilbert, VC* E 27 (EA); Ngorongoro Crater *Goddard, J* 81 (EA); Ngorongoro Crater *Goddard, J* 89 (EA); Ngorongoro Crater *Goddard, J* 97 (EA); Ngorongoro Crater *Greenway, PJ* 3366 (EA,K); Lake Magadi *Peterson, PM* 24321 (US); Ngorongoro Crater *Pole Evans, IB* 972 (K); Ngorongoro Crater *West, D* 4155 (K); **Mara** Serengeti National Park *Braun, HMH* 283 (EA); Serengeti National Park *Greenway, PJ* 10488 (EA,K); Engare Nanyuki *Newbould, JB* 6304 (EA,K); Serengeti Plains *Paulo, S* 372 (EA,K); Simba Kopjes *Vesey-FitzGerald, LDEF* 6617 (EA);

***Digitaria diagonalis* (Nees) Stapf , Fl. Cap. 7: 381 (1898)**
(Synonym: *Digitaria diagonalis* Stapf var. *uniglumis* (A.Rich) Pilg.)
Distribution: Tropical Africa & Arabia
Specimens: Arusha Lerong *Newbould, JB* 5634 (K); Mokilal *Newbould, JB* 6498 (K); **Mara** Tabora river *Greenway, PJ* 10127 (K);

***Digitaria macroblephara* (Hack. ex Schinz) Paoli , Miss. Somal. Ital. Merid., Relaz.: 245 (1916)**
Distribution: Tanzania, Kenya, Uganda, Ethiopia, Somalia & Sudan
Specimens: Arusha Kakesio River *Hornby* 2107 (K); Oldiang'arang'ar Ang'ata *Newbould, JB* 6544 (K); Lemuta hill *Paulo, S* 351 (K); Olduvai *Raynal, J* 19304 (K); **Mara** Nyaraswiga *Braun, HMH* 99 (K); Sand hill *Brooks, GP* 48 (K); Banagi Hill *Brooks, GP* 96 (K); Seronera *Greenway, PJ* 9837 (K); Robanda village *Mollel, NP* 829 (NHT); Serengeti *Paulo, S* 339 (K); Soitayai *Paulo, S* 362 (K); **Shinyanga** Lake Magadi *Leippert, H* 5595 (K); Lake Magadi *Paulo, S* 401 (K); Naabi Hill Gate *Peterson, PM* 24257 (US);

***Digitaria nodosa* Parl. , Pl. Nov. 39 (1842)**
Distribution: Tropical Africa, Asia & Arabia
Specimens: Arusha Endoinyo Emboleh *Newbould, JB* 6521 (K);

***Digitaria rivae* (Chiov.) Stapf , Bull. Misc. Inform. Kew 1907: 213 (1907)**
Distribution: Eastern Africa & Arabia
Specimens: Mara Lobo Lodge *Peterson, PM* 24294 (US).

Digitaria sp.
Specimens: Arusha Kelogi *Ellemann, L.* 482 (AAU); Enja Shori *Ellemann, L.* 709 (AAU); Olmekeke *Ellemann, L.* 880 (AAU); Olbalbal swamp *Ellemann, L.* 1094 (AAU); Oldonyo-o-ogol *Ellemann, L.* 1119 (AAU);

***Digitaria ternata* A.Rich.Stapf , Fl. Cap. 7: 376 (1898)**
Distribution: Widespread
Specimens: Arusha Endulen *Chuwa, S* 2637 (NHT); Endulen *Ellemann, L* 367 (AAU); Empakaai crater *Frame, GW* 87 (NHT);

***Digitaria velutina* (Forssk.) P.Beauv. , Ess. Agrostogr. 51 (1812)**
Distribution: Tropical Africa & Arabia
Specimens: Arusha Olkarien *Newbould, JB* 6409 (K); **Mara** Banagi *Greenway, PJ* 9972 (K); Seronera *Greenway, PJ* 10573 (K);

DIHETEROPOGON Stapf
***Diheteropogon amplectens* (Nees) *Clayton var. amplectens* , Kew Bull. 20: 75 (1966)**
Distribution: Tropical Africa
Specimens: Mara Seronera *Greenway, PJ* 9900 (K); Banagi *Greenway, PJ* 9975 (K); Mara River Guard Post *Greenway, PJ* 10770 (K); Tutishi river *Greenway, PJ* 10905 (K);

DINEBRA Jacq.
***Dinebra caudata* (K.Schum.) P.M. Peterson & N.Snow , Ann. Bot. (Oxford) 109: 1326 (2012)**
(Synonym: *Leptochloa caudata* (K.Schum.) N.Snow)
Distribution: Tanzania, Kenya, Uganda, Rwanda and Democratic Republic of Congo
Specimens: Arusha Klein's camp *Greenway, PJ* 10665 (K); **Mara** Seronera *Greenway, PJ* 10152 (K); Campi ya Mawi *Greenway, PJ* 13345 (K);

***Dinebra retroflexa* (Vahl) Panz. , Denkschr. Königl. Akad. Wiss. München 4: 270 (1813)**
Distribution: Widespread
Specimens: Arusha Laetoli valley *Mollel, NP* 1202 (NCAA,NHT);

***Dinebra retroflexa* (Vahl) Panz. *var. condensata* S.M.Philips , Kew Bull. 28(3): 414 (1974)**
Distribution: Tropical Africa & Asia
Specimens: Mara Nyakoromo Guard Post *Greenway, PJ* 10613 (K); Musabi *Greenway, PJ* 13168 (?);

DIPLACHNE P.Beauv.
***Diplachne fusca* (L.) P.Beauv. ex Roem. & Schult. , Syst. Veg. 2: 615 (1817)**
(Synonym: *Leptochloa fusca* (L.) Kunth ; *Leptochloa fusca* (L.) Kunth subsp. *fusca*)
Distribution: Widespread
Specimens: Arusha Ngorongoro Crater *Greenway, PJ* 12593 (K); Lake Magadi *Peterson, PM* 24322 (US); Ngorongoro Crater *Raynal, J* 19521 (K); **Mara** Titushi river flats *Greenway, PJ* 10501 (K); Serengeti Plains *Greenway, PJ* 13183 (K); **Shinyanga** Mbalageti river *Greenway, PJ* 9027 (K); Lake Magadi *Greenway, PJ* 10043 (K); Lake Magadi *Greenway, PJ* 12535 (K);

DISAKISPERMA Steud.
***Disakisperma obtusiflorum* (Hochst.) P.M. Peterson & N.Snow , Ann. Bot. (Oxford) 109: 1327 (2012)**
(Synonym: *Leptochloa obtusiflora* Hochst.)
Distribution: Tropical Africa & Arabia
Specimens: Arusha Klein's camp *Greenway, PJ* 10666 (K); **Mara** Banagi *Greenway, PJ* 9971 (K);

ECHINOCHLOA P.Beauv.
***Echinochloa brevipedicellata* (Peter) Clayton , Kew Bull. 32: 580 (1978)**
Distribution: Tanzania & Kenya
Specimens: Mara Serengeti National Park *Banyikwa, FF* 3 B (EA);

Echinochloa colona **(L.) Link , Hort. Berol. 2: 209 (1833)**
Distribution: Widespread
Specimens: Mara Seronera Lodge *Mboya, E* s.n. (MO); Serengeti National Park *Mboya, E* s.n. (MO); Serengeti Research Centre *Mboya, E* s.n. (MO);

Echinochloa haploclada **(Stapf) Stapf , Fl. Trop. Afr. 9: 613 (1920)**
Distribution: Tropical Africa
Specimens: Mara Seronera *Greenway, PJ* 9839 (K); Banagi *Leippert* 5605 (K); Lobo springs *Vesey-FitzGerald, LDEF* 5391 (K);

Echinochloa ugandensis **Snowden & C.E.Hubb. , Bull. Misc. Inform. Kew 1936: 315 (1936)**
Distribution: Eastern & Southern Africa
Specimens: Arusha Ngorongoro Crater *Greenway, PJ* 12616 (?); **Mara** Seronera *Greenway, PJ* 9860 (?);

EHRHARTA Thunb.
Ehrharta erecta **Lam. , Encycl. 2: 347 (1786)**
(Synonym: *Ehrharta erecta* Lam. var. *abyssinica* (Hochst.) Pilg.)
Distribution: Tropical Africa & Arabia
Specimens: Arusha Empakai Crater *Frame, GW* 186 (K); Nainokanoka *Newbould, JB* 6248 (K); Lemigrut *Njau, E* EN 803 (NHT);

ELEUSINE Gaertn.
Eleusine jaegeri **Pilg. , Bot. Jahrb. Syst. 43: 93 (1909)**
Distribution: East Africa
Specimens: Arusha Ngorongoro Crater *Bogdan* G41 (K); Ngorongoro Crater *Burtt, BD* 4302 (K); Oloronyo *Ellemann, L* 664 (AAU); Sendui *Ellemann, L* 718 (AAU); Rhino lodge *Ellemann, L* 861 (AAU); Armakutian *Ellemann, L* 909 (AAU); Empakai Crater *Frame, GW* 43 (EA); Empakai Crater *Frame, GW* 46 (EA,K); Ngorongoro *Goddard, J* 122 (EA); Ngorongoro Crater *Greenway, PJ* 3368 (EA,K); Nainokanoka *Greenway, PJ* 9149 (K); Ngorongoro Crater *Khayota, B* 244 (EA); Oldeani Mt *Moreau* 90 (EA,K); Ngorongoro *Moreau, RE* 89 (EA); Ngorongoro Crater *Napper, D* s.n. (EA,K); Ngorongoro Crater *Peterson, PM* 24299 (US); Ngorongoro Crater *Pole Evans, IB* 898 (K); Ngorongoro Crater *Pole Evans, IB* 979 (K); Ngorongoro Crater *Pole Evans, IB* 981 (K); Ngorongoro Crater *Raynal, J* 19056 (K); Lemagarut Mt. *Salt, G* 49 (K);

Eleusine multiflora **Hochst. ex A.Rich. , Tent. Fl. Abyss. 2: 412 (1850)**
Distribution: Eastern Africa & Arabia
Specimens: Arusha Ngorongoro Crater *Bally, PRO* B 2584 (EA); Olduvai Gorge *Chuwa, S* 2787 (K); Ngorongoro Crater *Frame, GW* 501 (EA); Ngorongoro *Newbould, JB* 5652 (EA,K); **Mara** Seronera *Greenway, PJ* 10574 (EA,K); **Shinyanga** Naabi Hill Gate *Peterson, PM* 24272 (US);

ELIONURUS Humb. & Bonpl. ex Willd.
Elionurus muticus **(Spreng.) Kuntze , Revis. Gen. Pl. 3(2): 350 (1898)**
(Synonym: *Elionurus argenteus* Nees)
Distribution: Widespread
Specimens: Mara Mara River Guard Post *Greenway, PJ* 10238 (K); Lobo range *Greenway, PJ* 10307 (K); Mara River Guard Post *Greenway, PJ* 10769 (K);

ENNEAPOGON Desv. ex P.Beauv.
Enneapogon cenchroides **(Licht.) C.E.Hubb , Bull. Misc. Inform. Kew 1934: 119 (1934)**
Distribution: Tropical Africa, Arabia & Asia
Specimens: Arusha Mosonik *Newbould, JB* 6356 (K); **Mara** Banagi *Greenway, PJ* 10345 (K); Serengeti Research Centre *Mboya, E* s.n. (MO); Salambala *Newbould, JB* 6294 (K);

Enneapogon desvauxii **P.Beauv. , Ess. Agrostogr. 82 (1812)**
(Synonym: *Enneapogon brachystachyus* Stapf)
Distribution: Tropical Africa, Asia & Americas
Specimens: Arusha Olbalbal *Paulo, S* 412 (K); Ngorongoro *Robson, TO* 202 (K);

***Enneapogon persicus* Boiss. , Diagn. Pl. Orient. ser. 1, 5: 71 (1844)**
(Synonym: *Enneapogon elegans* (Nees ex Steud.) Stapf)
Distribution: Tropical Africa & Asia
Specimens: Arusha Gol Kopjes *Greenway, PJ* 10524 (K); Ngorongoro Crater *Greenway, PJ* 13605 (K); **Mara** Seronera *Greenway, PJ* 10071 (K); Banagi *Greenway, PJ* 10347 (K); **Shinyanga** Ipumba Kopje *Greenway, PJ* 10367 (K);

Enneapogon sp.
Specimens: Shinyanga Naabi Hill Gate *Peterson, P.M.* 24265 (US);

ENTEROPOGON Nees
***Enteropogon macrostachyus* (Hochst. ex A.Rich) Munro ex Benth. , J. Linn. Soc., Bot. 19: 101 (1881)**
Distribution: Tropical Africa & Arabia
Specimens: Mara Seronera *Greenway, PJ* 9928 (K);

Enteropogon sp.
Specimens: Mara Musabi plains *Mboya, E.* s.n. (MO);

ERAGROSTIS Wolf
***Eragrostis aethiopica* Chiov. , Somalia & Benadir: 726 (1899)**
Distribution: Tropical Africa & Arabia
Specimens: Mara Seronera *Greenway, PJ* 10542 (K); **Shinyanga** Moru Kopjes *Paulo, S* 388 (K);

***Eragrostis aspera* (Jacq.) Nees , Fl. Afr. Austral. Ill. 408. (1841)**
Distribution: Tropical Africa, Arabia & Asia
Specimens: Mara Seronera *Greenway, PJ* 10192 (K); **Shinyanga** Naabi Hill *Greenway, PJ* 10359 (K);
***Eragrostis cilianensis* (All.) Janch. , Mitt. Naturwiss. Vereins Univ. Wien 5(9): 110 (1907)**
(Synonym: *Eragrostis polysperma* Peter)
Distribution: Widespread
Specimens: Arusha Ngorongoro Crater *Chuwa, S* 2799 (K); Ol Doinyo Lengai *Greenway, PJ* 11353 (K); Engaruka *Leippert, H* 6359 (K); Olkarien *Newbould, JB* 6392 (K); Olbalbal *Paulo, S* 322 (K); Ngorongoro Crater *Peter, A* 43114 (W); **Mara** Seronera *Greenway, PJ* 9983 (K); Nyaraswiga Hill *Greenway, PJ* 10495 (K); Kirawira Plains *Greenway, PJ* 13313 (K); **Shinyanga** Lake Magadi *Greenway, PJ* 10504 (K);

***Eragrostis exasperata* Peter , Repert. Spec. Nov. Regni Veg. Beih. 40(1 Anh.): 108 (1930)**
Distribution: Tropical Africa
Specimens: Mara Seronera *Greenway, PJ* 9952 (K); Tabora *Greenway, PJ* 10388 (K); Lobo springs *Vesey-FitzGerald, LDEF* 5392 (K);

***Eragrostis heteromera* Stapf , Fl. Cap. 7: 610 (1900)**
Distribution: Tropical Africa
Specimens: Mara Simba Kopjes *Braun, HMH* 228 (K); Ikoma *Brooks, GP* 37 (K); Seronera *Greenway, PJ* 9840 (K); Ndabaka Guard Post *Greenway, PJ* 13330 (K); Serengeti National Park *Mboya, E* s.n. (MO);

***Eragrostis hispida* K.Schum. , Pflanzenw. Ost-Afrikas, C: 114 (1895)**
Distribution: Tropical Africa
Specimens: Arusha Klein's camp *Greenway, PJ* 10939 (K); **Mara** Seronera *Greenway, PJ* 9996 (K);

***Eragrostis humidicola* Napper , Kirkia 3: 114 (1963)**
Distribution: Kenya, Tanzania, Uganda, Democratic Republic of Congo & Rwanda
Specimens: Mara Tabora *Greenway, PJ* 10134 (K);

***Eragrostis papposa* (Roem. & Schult.) Steud. , Nomencl. Bot. ed. 2, 1: 564(1840)**
(Synonym: *Eragrostis aulacosperma* (Fresen.) Steud.)
Distribution: Africa & Asia
Specimens: Arusha Ngorongoro Crater *Heady, AF* 1695 (EA); Ngorongoro *Heady, HF* 1675 (EA); Olbalbal *Newbould, JB* 5934 (EA); Olbalbal *Newbould, JB* 5991 (EA); Olbalbal *Newbould, JB* 6374 (EA,K); Olduvai Gorge

Vesey-FitzGerald, LDEF 4687 (EA); **Mara** Serengeti National Park *Kreulen, AR* 99 (EA); **Shinyanga** Naabi Hill *Braun, HMH* 256 (EA); Naabi Hill *Braun, HMH* 274 (EA);

Eragrostis pilosa **(L.) P.Beauv. , Ess. Agrostogr. 71 (1812)**
Distribution: Widespread
Specimens: Mara Seronera *Greenway, PJ* 9875 (K); Nyaraswiga Hill *Paulo, S* 395 (K);

Eragrostis racemosa **(Thunb.) Steud. , Syn. Pl. Glumac. 1(3): 271 (1854)**
Distribution: Tropical Africa
Specimens: Arusha Endulen *Ellemann, L* 776 (AAU); **Mara** Tabora *Greenway, PJ* 10130 (K); Seronera *Greenway, PJ* 10302 (K); **Shinyanga** Handajenga *Greenway, PJ* 13318 (K);

Eragrostis schweinfurthii **Chiov. , Annuario Reale Ist. Bot. Roma 8: 368 (1908)**
Distribution: Tropical Africa & Arabia
Specimens: Arusha Mokilal *Chuwa, S* 2751 (K); Ngorongoro Crater *Pole Evans, IB* 908 (K);

Eragrostis sp.
Specimens: Arusha Ngorongoro Crater *Peterson, P.M.* 24302 (US); **Mara** Mbuzi Mare camp *Peterson, P.M.* 24283 (US); Lobo Lodge *Peterson, P.M.* 24290 (US); Lobo Lodge *Peterson, P.M.* 24291 (US); Lobo Lodge *Peterson, P.M.* 24292 (US); **Shinyanga** Naabi Hill Gate *Peterson, P.M.* 24261 (US); Naabi Hill Gate *Peterson, P.M.* 24267 (US); Naabi Hill Gate *Peterson, P.M.* 24271 (US);

Eragrostis tenuifolia (A.Rich.) Hochst. ex Steud. , Syn. Pl. Glumac. 1(3): 268 (1854)
Distribution: Tropical Africa
Specimens: Arusha Mokilal *Chuwa, S* 2751b (K); Sendui *Ellemann, L* 717 (AAU); Empakai Crater *Frame, GW* 90 (K,NHT); Ngorongoro Crater *Pole Evans, IB* 25130 (K); **Mara** Banagi Hill *Brooks, GP* 72 (K); Seronera *Greenway, PJ* 9847 (K);

Eragrostis viscosa **Trin. , Mém. Acad. Imp. Sci. St.-Pétersbourg, Sér. 6, Sci. Math. 1: 397 (1830)**
Distribution: Tropical Africa, Asia & Arabia
Specimens: Arusha Olduvai Gorge *Verdcourt, B* 4028 (K); **Mara** Engare Nanyuki springs *Greenway, PJ* 10703 (K);

ERIOCHLOA Kunth
Eriochloa fatmensis **(Hochst. & Steud.) Clayton , Kew Bull. 30: 108 (1975)**
(Synonym: *Eriochloa nubica* (Steud.) Hack. & Stapf ex Thell.)
Distribution: Tropical Africa & Arabia
Specimens: Arusha Esere *Chuwa, S* 2585 (K,NHT); Ngorongoro Crater *Chuwa, SM* 2097 K (K); Endulen *Ellemann, L* 371 (AAU); Naibardad Hill *Newbould, JB* 6515 (K); Ngorongoro Crater *Paulo, S* 319 (K); **Mara** Seronera *Greenway, PJ* 9861 (K); Robanda village *Mollel, NP* 839 (NHT); Serengeti NP *Mollel, NP* 899 (NHT); Olndulen *Paulo, S* 414 (K);

Eriochloa meyeriana **(Nees) Pilg. , Nat. Pflanzenfam. ed. 2, 14e: 56 (1940)**
Distribution: Tropical Africa & Arabia
Specimens: Mara Banagi Hill *Greenway, PJ* 9036 (K); Seronera river *Greenway, PJ* 10179 (K,SWRC); Seronera river *Greenway, PJ* 10600 (SWRC);

Eriochloa sp.
Specimens: Mara Serengeti NP *Mboya, E.* s.n. (MO);

EUSTACHYS Desv.
Eustachys paspaloides **(Vahl) Lanza & Mattei , Reale Orto Bot. Palermo 9: 56 (1910)**
Distribution: Tropical Africa & Arabia
Specimens: Mara Banagi Hill *Brooks, GP* 65 (K); Ndabaka Plains *Greenway, PJ* 9093 (K); Seronera *Greenway, PJ* 9845 (K); Serengeti Plains *Leippert, H* 5596 (K); Serengeti NP *Mollel, NP* 909 (NHT); Serengeti Plains *Paulo, S* 347 (K); Banagi Hill *Paulo, S* 364 (K); Lobo Lodge *Peterson, PM* 24295 (US); **Shinyanga** Naabi Hill Gate *Peterson, PM* 24264 (US);

EXOTHECA Andersson
Exotheca abyssinica **(Hochst. ex A.Rich.) Andersson , Nova Acta Regiae Soc. Sci. Upsal., ser. 3, 2: 253 (1856)**
Distribution: Eastern & Southern Africa
Specimens: Arusha Oloronyo *Ellemann, L* 771 (AAU); Lemigrut *Njau, E* EN 758 (NHT);

FESTUCA Tourn. ex L.
Festuca obturbans **St.-Yves , Rev. Bretonne Bot. Pure Appl. 2: 83 (1927)**
Distribution: Tanzania, Kenya & Yemen
Specimens: Arusha Empakai Crater *Greenway, PJ* 9140 (K);

HARPACHNE Hochst. ex A.Rich.
Harpachne schimperi **A.Rich. , Tent. Fl. Abyss. 2: 431 (1850)**
Distribution: Eastern Africa & Arabia
Specimens: Arusha Lake Magadi *Peterson, PM* 24319 (US); **Mara** Serengeti Research Centre *Mboya, E* s.n. (MO); Mbuzi Mare camp *Peterson, PM* 24285 (US); Lobo Lodge *Peterson, PM* 24293 (US); **Shinyanga** Naabi Hill Gate *Peterson, PM* 24266 (US);

HELICTOTRICHON Besser
Helictotrichon elongatum **(Hochst. ex A.Rich.) C.E.Hubb , Bull. Misc. Inform. Kew 1936: 335 (1936)**
Distribution: Tropical Africa
Specimens: Arusha Empakai Crater *Frame, GW* P 82 (K);

Helictotrichon lachnanthum **(Hochst. ex A.Rich.) C.E.Hubb. , Bull. Misc. Inform. Kew 1936: 335 (1936)**
Distribution: Ghana, Ethiopia, Kenya, Tanzania & Uganda
Specimens: Arusha Empakai Crater *Frame, GW* 189 (K); Empakai Crater *Greenway, PJ* 9130 (K); Nainokanoka *Newbould, JB* 5614 (K);

HETEROPOGON Pers.
Heteropogon contortus **(L.) P.Beauv. ex Roem. & Schult. , Syst. Veg. 2: 836 (1817)**
Distribution: Widespread
Specimens: Arusha Ngorongoro Crater *Metele, P* 3 (NHT); **Mara** Seronera *Greenway, PJ* 9857 (K); Serengeti Research Centre *Mboya, E* s.n. (MO); Robanda village *Mollel, NP* 847 (NHT);

HYPARRHENIA Andersson ex E.Fourn.
Hyparrhenia anamesa **Clayton , Kew Bull., Addit. Ser. 2: 85 (1969)**
Distribution: Tropical Africa
Specimens: Arusha Ngorongoro Crater *Greenway, PJ* 12598 (K);

Hyparrhenia anthistirioides **(Hochst. & A.Rich.) Andersson ex Stapf , Fl. Trop. Afr. 9: 331 (1918)**
Distribution: Tropical Africa
Specimens: Arusha Laetoli *Mollel, NP* 1165 (NHT); **Shinyanga** Ndugani Kopjes *Greenway, PJ* 10363 (K); Moru Kopjes *Greenway, PJ* 13177 (K);

Hyparrhenia cymbaria **(L.) Stapf , Fl. Trop. Afr. 9: 332 (1919).**
Distribution: Tropical Africa & Asia
Specimens: Arusha Ngorongoro *Tosbrooke, J* 119 (K);

Hyparrhenia dregeana **(Nees) Stapf ex Stent , Bothalia 1: 249 (1923)**
Distribution: Tropical Africa & Arabia
Specimens: Arusha Ngorongoro Crater *Heady, AF* 1343 (K);

Hyparrhenia filipendula **(Hochst.) Stapf , Fl. Trop. Afr. 9: 322 (1918)**
Distribution: Tropical Africa & Asia
Specimens: Arusha Klein's camp *Greenway, PJ* 10125 (K); **Mara** Tabora *Greenway, PJ* 10128 (K); Duma river air strip *Greenway, PJ* 10911 (K);

Hyparrhenia finitima **(Hochst.) Andersson ex Stapf , Fl. Trop. Afr. 9: 299 (1918)**
Distribution: Tropical Africa
Specimens: Mara Seronera *Greenway, PJ* 10072 (K);

Hyparrhenia hirta **(L.) Stapf , Fl. Trop. Afr. 9: 315 (1918)**
Distribution: Widespread
Specimens: Arusha Endulen *Chuwa, S* 2227 (K); Ngorongoro Crater *Chuwa, S* 2745 (K,NHT); Empakaai crater *Frame, GW* 182 (NHT); Klein's camp *Greenway, PJ* 9999 (K); Engitati Hill *Greenway, PJ* 12608 (K,NHT); Ngorongoro Crater *Heady, AF* 1637 (K); Ngorongoro Crater *Metele, P* s.n. (MO);

Hyparrhenia papillipes **(Hochst. ex A.Rich.) Andersson ex Stapf , Fl. Trop. Afr. 9: 347 (1918)**
Distribution: Eastern Africa, Yemen & Madagascar
Specimens: Arusha Ngorongoro *Giemeisel, QL* 494 (K); **Mara** Serengeti *Greenway, PJ* 10690 (K); Engare Nanyuki *Greenway, PJ* 10704 (K); Engare Nanyuki *Newbould, JB* 6185 (K);

Hyparrhenia pilgeriana **C.E.Hubb , Bull. Misc. Inform. Kew 1928: 39 (1928)**
Distribution: Tropical Africa
Specimens: Arusha Empakai Crater *Frame, GW* 92 (K,NHT); Empakai Crater *Frame, GW* 140 (K,NHT);

Hyparrhenia quarrei **Robyns , Fl. Agrost. Congo Belge 1: 171 (1929)**
Distribution: Tropical Africa
Specimens: Arusha Ngorongoro Crater *Peterson, PM* 24309 (US);

Hyparrhenia rufa **(Nees) Stapf , Fl. Trop. Afr. 9: 304 (1918)**
Distribution: Tropical Africa
Specimens: Mara Nyamakachowe *Greenway, PJ* 10507 (K); Musabi *Greenway, PJ* 10619 (K); **Shinyanga** Beacon Area *Greenway, PJ* 10554 (K); Subiti Hill *Paulo, S* 426 (K);

Hyparrhenia sp.
Specimens: Mara Serengeti NP *Mboya, E.* s.n. (MO);

HYPERTHELIA Clayton
Hyperthelia dissoluta **(Nees ex Steud.) Clayton , Kew Bull. 20: 441 (1966)**
Distribution: Tropical Africa
Specimens: Mara Seronera *Greenway, PJ* 9992 (K); Kirawira Guard Post *Greenway, PJ* 10090 (K); Mbuzi Mare camp *Peterson, PM* 24287 (US);

ISCHAEMUM L.
Ischaemum afrum **(J.F.Gmel.) Dandy , Fl. Pl. Sudan 3: 476 (1956)**
Distribution: Tropical Africa & India
Specimens: Arusha Ngorongoro Crater *Greenway, PJ* 11846 (K); Ngorongoro CA *Njau, E* EN 729 (NHT);

LEERSIA Sw.
Leersia denudata **Launert , Biol. 46: 144 (1965)**
Distribution: Eastern & Southern Africa
Specimens: Arusha Ngorongoro Crater *Gilbert, VC* E 25 (EA); Ngorongoro Crater *Greenway, PJ* 12570 (EA);

Leersia hexandra **Sw. , Prodr. Veg. Ind. Occ. 21 (1788)**
Distribution: Widespread
Specimens: Arusha Ngorongoro Crater *Newbould, JB* 6523 (K);

Leersia sp.
Specimens: Arusha Lake Madagi *Peterson, P.M.* 24323 (US);

LOUDETIA Hochst. ex Steud.
Loudetia arundinacea **(Hochst. ex A.Rich.) Hochst. ex Steud. , Syn. Pl. Glumac. 1: 238 (1854)**
Distribution: Tropical Africa

Specimens: Mara Kirawira *Greenway, PJ* 10087 (K); Mbuzi Mare camp *Peterson, PM* 24282 (US);

Loudetia kagerensis **(K.Schum.) C.E.Hubb , Fl. Trop. Afr. 10: 28 (1937)**
Distribution: Tropical Africa
Specimens: Mara Seronera *Greenway, PJ* 9991 (K); Tabora *Greenway, PJ* 10133 (K); Kampi ya pofu *Greenway, PJ* 13347 (K); Nyamakachowe Hill *Paulo, S* 397 (K);

Loudetia sp.
Specimens: Mara Serengeti Research Centre *Mboya, E.* s.n. (MO);

MELINIS P.Beauv.
Melinis repens **(Willd.) Zizka , Biblioth. Bot. 38: 55 (1988)**
Distribution: Widespread
Specimens: Mara Serengeti Research Centre *Mboya, E* s.n. (MO);

Melinis sp.
Specimens: Mara Serengeti NP *Mboya, E.* s.n. (MO);

MICROCHLOA R.Br.
Microchloa kunthii **Desv. , Mém. Soc. Acad. Maine Loire 1: 179 (1831)**
Distribution: Widespread
Specimens: Arusha Enja Shori *Ellemann, L* 710 (AAU); Olbili-Esere *Ellemann, L* 1016 (AAU); **Mara** Mbuzi Mare camp *Peterson, PM* 24288 (US); Seronera *Tanner, M* 12969 (EA,SWRC); **Shinyanga** Naabi Hill Gate *Peterson, PM* 24255 (US);

ODYSSEA Stapf.
Odyssea paucinervis **(Nees) Stapf , Hooker's Icon. Pl. 31: t. 3100 (1922*)***
Distribution: Eastern & Southern Africa
Specimens: Arusha Lake Magadi *Gilbert, VC* E 40 (K); Lake Magadi *Greenway, PJ* 12576 (K); Lake Magadi *Greenway, PJ* 13597 (K); Lake Magadi *Peterson, PM* 24312 (US);

OLDEANIA Stapleton
Oldeania alpina **(K.Schum.) Stapleton , PhytoKeys 25: 100. (2013)**
(Synonym: *Arundinaria alpina* K.Schum.)
Distribution: Tropical Africa
Specimens: Arusha Oldeani Mt *Burtt, BD* 4223 (K); Oldeani Mt *Chuwa, S* 2679 (K);

OPLISMENUS P.Beauv.
Oplismenus compositus **(L.) P.Beauv. , Ess. Agrostogr. 54 (1812)**
Distribution: Widespread
Specimens: Mara Bolgonja *Greenway, PJ* 10754 (SWRC);

Oplismenus hirtellus **(L.) P.Beauv. , Ess. Agrostogr. 54: 170 (1812)**
Distribution: Widespread
Specimens: Mara Serengeti National Park *Vesey-FitzGerald, LDEF* 4657 (SWRC);

OROPETIUM Trin.
Oropetium capense **Stapf , Fl. Cap. 7: 742(1900)**
Distribution: Africa & Arabia
Specimens: Mara Seronera *Greenway, PJ* 9854 (SWRC);

ORYZA L.
Oryza eichingeri **Peter , Repert. Spec. Nov. Regni Veg. Beih. 40(1 Anh.): 74 (1930)**
Distribution: Tropical Africa & Sri Lanka
Specimens: Mara Serengeti National Park *Vesey-FitzGerald, LDEF* 7591 (SWRC);

PANICUM L.

***Panicum atrosanguineum* Hochst. ex A.Rich. , Tent. Fl. Abyss. 2: 375 (1850)**
Distribution: Widespread
Specimens: Arusha Endulen *Chuwa, S* 2586 (K); Endoinyo Emboleh *Newbould, JB* 6569 (K); **Mara** Lake Lagarja *Belsky, PJ* 79 (SWRC); Lake Lagarja *Greenway, PJ* 10149 (K); Robanda village *Mollel, NP* 858 A (NHT); Serengeti Plains *Schmidt, W* 128 (SWRC); **Shinyanga** Naabi Hill *Brown, HM* 270 (SWRC,WAG); Lake Magadi *Greenway, PJ* 10081 (SWRC); Kitu Hill *Greenway, PJ* 10621 (K,SWRC);

***Panicum calvum* Stapf , Fl. Trop. Afr. 9: 723 (1920)**
Distribution: Tropical Africa
Specimens: Arusha Ngorongoro *Pocs, T* 89038 C (K);

***Panicum chionachne* Mez , Fl. Trop. Afr. 9: 723 (1920)**
Distribution: Tropical Africa
Specimens: Arusha Empakai Crater *Frame, GW* 181 (EA,SWRC); Empakai Crater *Vesey-FitzGerald, LDEF* 7606 (SWRC);

***Panicum coloratum* L. , Mant. Pl. 1: 30 (1767)**
(Synonym: *Panicum coloratum* L. var. *minus* Stapf ex Chiov.)
Distribution: Widespread
Specimens: Arusha Esere *Chuwa, S* 2583 (K); Lemuta *Oteke, J* 208 (K); Kambi ya Nyoka *Pocs, T* 89033 (K); **Mara** Serengeti Research Institute *Belsky, PJ* 212 (SWRC); Lobo Lodge *Belsky, PJ* 237 (SWRC); Serengeti Research Institute *Belsky, PJ* 244 (SWRC); Serengeti National Park *Braun, MH* 365 (SWRC); Seronera *Greenway, PJ* 9862 (SWRC); Seronera *Greenway, PJ* 10187 (K,SWRC); Serengeti Plains *Schmidt, W* 166 (SWRC); **Shinyanga** Lake Magadi *Greenway, PJ* 10505 (K,SWRC); Naabi Hill *Greenway, PJ* 10529 (K,SWRC); Naabi *Paulo, S* 369 (K); Naabi Hill *Schmidt, W* 228 (SWRC);

***Panicum commutatum* Schult. , Mant. 2: 242 (1824)**
(Synonym: *Panicum joorii* Vasey)
Distribution: North & South America
Specimens: Shinyanga Naabi Hill Gate *Peterson, PM* 24263 (US); Naabi Hill Gate *Peterson, PM* 24277 (US); Naabi Hill Gate *Peterson, PM* 24278 (US);

***Panicum deustum* Thunb. , Prodr. Pl. Cap. 19 (1794)**
Distribution: Tropical Africa & Afghanistan
Specimens: Arusha Ngorongoro Crater *Peterson, PM* 24311 (US); **Mara** Bolgonja *Schmidt, W* 418 (SWRC); Bolgonja *Schmidt, W* 522 (SWRC); Bolgonja *Schmidt, W* 523 (SWRC); Seronera *Unknown* s.n. (SWRC);

***Panicum humile* Steud. , Syn. Pl. Glumac. 1: 84 (1854)**
(Synonym: *Panicum watense* Mez)
Distribution: Widespread
Specimens: Arusha Ngorongoro Crater *Peterson, PM* 24304 (US);

***Panicum hygrocharis* Steud. , Syn. Pl. Glumac. 1: 72 (1853)**
Distribution: Tropical Africa
Specimens: Arusha Munge Swamp *Greenway, PJ* 12573 (K); Lerai stream *Newbould, JB* 6252 (K); Ngorongoro *Vesey-FitzGerald, LDEF* 4770 (SWRC); **Mara** Seronera *Greenway, PJ* 10354 (K,SWRC); Seronera river *Greenway, PJ* 10599 (K,SWRC); Serengeti *Kreulen, AR* 321 (SWRC);

***Panicum hymeniochilum* Nees , Fl. Afr. Austral. Ill. 46 (1841)**
Distribution: Tropical Africa
Specimens: Arusha Oldeani Mt *Chuwa, S* 2471 (K);

***Panicum infestum* Andersson , Naturw. Reise Mossambique 2: 546 (1864)**
Distribution: Tropical Africa
Specimens: Mara Kirawira *Vesey-FitzGerald, LDEF* 4658 (SWRC);

***Panicum massaiense* Stapf , Bot. Jahrb. Syst. 34: 144 (1904)**
Distribution: Tropical Africa
Specimens: Shinyanga Mbono river *Greenway, PJ* 10371 (K,SWRC); Beacon Area *Greenway, PJ* 10551 (K,SWRC); Naabi Hill *Paulo, S* 417 (K);

***Panicum maximum* Jacq. , Icon. Pl. Rar. 1: t. 13 (1781)**
(Synonym: *Megathyrsus maximus* (Jacq.) B.K.Simon & S.W.L.Jacobs)
Distribution: Widespread
Specimens: Arusha Nasiporiong *Ellemann, L* 817 (AAU); Naibardad Hill *Newbould, JB* 6434 (K); **Mara** Serengeti Research Institute *Belsky, PJ* 5 (SWRC); Serengeti *Belsky, PJ* 226 (SWRC); Seronera dam *Greenway, PJ* 9981 (K,SWRC); Serengeti Research Centre *Mboya, E* s.n. (MO); Serengeti Research Centre *Mboya, E* s.n. (MO); Bolgonja *Schmidt, W* 371 (SWRC); **Shinyanga** Ngamuriak *Newbould, JB* 6279 (K);

***Panicum monticola* Hook.f. , J. Proc. Linn. Soc., Bot. 7: 226 (1864)**
Distribution: Tropical Africa
Specimens: Arusha Empakai Crater *Frame, GW* 154 (EA,K,NHT,SWRC);

***Panicum poioides* Stapf , Fl. Trop. Afr. 9: 681 (1920)**
(Synonym: *Panicum graciliculme* Napper)
Distribution: Tropical Africa
Specimens: Mara Serengeti *Greenway, PJ* 247 (SWRC); Seronera *Greenway, PJ* 9841 (K,NY,PRE,PRE,SWRC,US); Seronera *Hubert, MHB* 264 (SWRC); Seronera *Hubert, MHB* 269 (SWRC); Serengeti *Kreulen, AR* 323 (SWRC);

***Panicum repens* L. , Sp. Pl. ed. 2, 1: 87 (1762)**
Distribution: Widespread
Specimens: Arusha Oldeani Mt *Chuwa, S* 2678 (K); **Mara** Serengeti National Park *Suleiman, HO* 1094 (SWRC);

Panicum sp.
Specimens: Shinyanga Moru Kapjes *Braun, H.M.H.* 26 (SWRC);

PASPALIDIUM Stapf.
***Paspalidium geminatum* (Forssk.) Stapf , Fl. Trop. Afr. 9: 583 (1920)**
(Synonym: *Setaria geminata* (Forssk.) Veldkamp)
Distribution: Widespread
Specimens: Mara Banagi *Greenway, PJ* 10348 (K,SWRC);

PASPALUM L.
***Paspalum scrobiculatum* L. , Mant. Pl. 1: 29 (1767)**
(Synonym: *Paspalum commersonii* Lam.)
Distribution: Widespread
Specimens: Mara Bolgonja river *Greenway, PJ* 10744 (K);

PENNISETUM Pers.
***Pennisetum clandestinum* Hochst. ex Chiov. , Annuario Reale Ist. Bot. Roma 8: 41 (1903)**
(Synonym: *Cenchrus clandestinus* (Hochst. ex Chiov.) Morrone)
Distribution: Native to Eastern Africa, introduced widely
Specimens: Arusha Sendui *Ellemann, L* 715 (AAU); Irmisigiyo *Ellemann, L* 774 (AAU); Esongoyo *Ellemann, L* 833 (AAU); Rhino lodge *Ellemann, L* 863 (AAU); Armakutian *Ellemann, L* 910b (AAU); Irmisigiyo *Ellemann, L* 944 (AAU); Empakai Crater *Frame, GW* 103 (K,SWRC);

***Pennisetum mezianum* Leeke , Z. Naturwiss. 79: 39 (1907)**
Distribution: Tropical Africa
Specimens: Arusha Olbalbal *Ellemann, L* 985 (AAU); Olbili-Esere *Ellemann, L* 1017 (AAU); Ngorongoro Crater *Heady, AF* 1505 (K); Ngorongoro Crater *Paulo, S* 318 (K); **Mara** Serengeti Research Institute *Belsky, PJ* 172 (SWRC); Banagi Hill *Brooks, GP* 70 (K); Seronera *Greenway, PJ* 9836 (K,SWRC); Serengeti National Park *Mboya, E* s.n. (MO); Seronera airdrome *Paulo, S* 294 (K); Engare Nanyuki *Paulo, S* 361 (K); Lake Lagarja *Raynal, J* 19340 (K); Simba Kopjes *Schmidt, W* 17 (SWRC); **Shinyanga** Naabi Hill *Kreulen, AR* 229 (SWRC);

***Pennisetum polystachion* (L.) Schult. , Mant. 2: 146 (1824)**
Distribution: Widespread
Specimens: Arusha Ololgumi *Ellemann, L* 658 (AAU);

***Pennisetum polystachion* (L.) Schult. subsp atrichum (Stapf & C.E.Hubb) Brunken , Bot. J. Linn. Soc. 79: 63 (1979)**
Distribution: Tropical Africa
Specimens: Arusha Endulen *Ellemann, L* 355 (AAU);

Pennisetum polystachion* (L.) Schult. *subsp polystachion
Distribution: Widespread
Specimens: Arusha Sendui *Ellemann, L* 721 (AAU); Oltebesi *Ellemann, L* 934 (AAU); Irmisigiyo *Ellemann, L* 945 (AAU); Oldonyo *Ellemann, L* 7763 (AAU);

***Pennisetum riparium* Hochst. ex A.Rich. , Tent. Fl. Abyss. 2: 381 (1850)**
(Synonym: *Cenchrus dowsonii* (Stapf & C.E.Hubb) Morrone ; *Pennisetum dowsonii* Stapf)
Distribution: Tanzania, Kenya, Uganda, Ethiopia & Burundi
Specimens: Arusha Munge Swamp *Greenway, PJ* 12547 (K); Ngorongoro Crater *Heady, AF* 1663 (?); Ngorongoro Crater *Heady, HF* 1663 (K); Ngorongoro *Pocs, T* 89016 A (K); Ngorongoro *Senga, C* 31 (K);

***Pennisetum setaceum* (Forssk.) Chiov. , Boll. Soc. Bot. Ital. 1923: 113 (1923)**
(Synonym: *Cenchrus setaceus* (Forssk.) Morrone)
Distribution: Widespread
Specimens: Arusha Ngorongoro *Chuwa, S* 2599 (K); Kelogi *Ellemann, L* 483 (AAU); Lolgarien *Newbould, JB* 4 (K); **Mara** Seronera *Greenway, PJ* 10356 (K,SWRC); Simba Kopjes *Greenway, PJ* 13152 (K,SWRC);

Pennisetum sp.
Specimens: Arusha Kelogi *Ellemann, L.* 481 (AAU);

***Pennisetum sphacelatum* (Nees) T.Durand & Schinz , Consp. Fl. Afric. 5: 784 (1894)**
(Synonym: *Cenchrus sphacelatus* (Nees) Morrone ; *Pennisetum schimperi* A.Rich.)
Distribution: Tropical Africa
Specimens: Arusha Oloronyo *Ellemann, L* 665 (AAU); Endulen *Ellemann, L* 698 (AAU); Armakutian *Ellemann, L* 910 a (AAU); Empakai Crater *Frame, GW* 124 (NHT,SWRC); Ngorongoro Crater *Heady, AF* 1670 (K); Ngorongoro *Newbould, JB* 5651 (K); Ngorongoro Crater *Peterson, PM* 24308 (US); Ngorongoro Crater *Pole Evans, IB* 970 (K); Ngorongoro *Welch, JR* 601 (K);

***Pennisetum squamulatum* Fresen. , Mus. Senckenberg. 2: 137 (1837)**
(Synonym: *Cenchrus squamulatum* (Fresen.) Morrone)
Distribution: Tanzania, Kenya, Ethiopia & Eritrea
Specimens: Arusha Olkarien *Newbould, JB* 6401 (K); **Shinyanga** Barafu Kopjes *Greenway, PJ* 13173 (K,SWRC);

***Pennisetum stramineum* Peter , Repert. Spec. Nov. Regni Veg. Beih. 40(1 Anh.): 71 (1930)**
(Synonym: *Cenchrus stramineus* (A. Peter) Morrone)
Distribution: Eastern Africa & Arabia
Specimens: Arusha Empakai Crater *Frame, GW* 533 (SWRC); Ngorongoro Crater *Heady, HF* 1503 (K); **Mara** Ndutu Lodge *Belsky, PJ* 66 (SWRC); Seronera *Greenway, PJ* 10053 (K,SWRC); Seronera *Paulo, S* 299 (K); Serengeti *Paulo, S* 341 (K); Simba Kopjes *Schmidt, W* 43 (SWRC); **Shinyanga** Naabi Hill *Greenway, PJ* 9159 (K); Naabi Hill *Kreulen, AR* 230 (SWRC); Naabi Hill Gate *Peterson, PM* 24276 (US);

***Pennisetum trachyphyllum* Pilg. , Bot. Jahrb. Syst. 30: 122 (1901)**
(Synonym: *Cenchrus trachyphyllum* (Pilg.) Morrone)
Distribution: Eastern & Central Africa
Specimens: Arusha Empakai Crater *Frame, GW* 238 (SWRC);

PEROTIS Aiton
***Perotis patens* Gand. , Bull. Soc. Bot. France 66: 301 (1919 publ. 1920)**
Distribution: Tropical Africa
Specimens: Mara Seronera *Greenway, PJ* 10198 (SWRC); Bolgonja *Schmidt, W* 495 (SWRC);

PHALARIS L.
***Phalaris arundinacea* L. , Sp. Pl. 2: 55 (1753)**
Distribution: Widespread
Specimens: Arusha Lemala Forest *Chuwa, S* 2725 (K); Empakai Crater *Frame, GW* 187 (K,SWRC); Olmoti crater *Greenway, PJ* 9147 (K); Ngorongoro Crater *Metele, P* s.n. (MO); Ngorongoro *Napper, D* 1682 (K); Rotian glade *Pocs, T* 89016 E (K); Olmoti crater *Raynal, J* 19559 (K); Ngorongoro *Tanner, M* 3270 (K);

PHRAGMITES Adans.
***Phragmites australis* (Cav.) Trin. ex Steud. , Nomencl. Bot. ed. 2, 2: 324 (1841)**
Distribution: Widespread
Specimens: Mara Kirawira *Greenway, PJ* 10377 (K);

POA L.
***Poa annua* L. , Sp. Pl. 1: 68 (1753)**
Distribution: Widespread
Specimens: Arusha Ngorongoro Crater *Greenway, PJ* 12531 (K); Ngorongoro Crater *Peterson, PM* 24301 (US);

***Poa leptoclada* Hochst. ex A.Rich. , Tent. Fl. Abyss. 2: 422 (1850)**
Distribution: Tropical Africa & Arabia
Specimens: Arusha Empakai Crater *Frame, GW* P 17 (K); Ngorongoro Crater *Greenway, PJ* 3361 (K); Empakai Crater *Greenway, PJ* 9137 (K); Ngorongoro Crater *Greenway, PJ* 12530 (K); Oldeani Mt *Moreau* 96 (K); Ngorongoro FR *Newbould, JB* 5681 (K); Nainokanoka *Newbould, JB* 6244 (K); Ngorongoro Crater *Peterson, PM* 24300 (US);

POGONARTHRIA Stapf
***Pogonarthria squarrosa* (Roem. & Schult.) Pilg. , Notizbl. Königl. Bot. Gart. Berlin 5: 149 (1910)**
Distribution: Tropical Africa
Specimens: Mara Seronera-Klein's camp *Greenway, PJ* 10199 (K);

POGONONEURA Napper
***Pogononeura biflora* Napper , Kirkia 3: 112 (1963)**
Distribution: Tanzania & Uganda
Specimens: Mara Seronera river *Greenway, PJ* 10091 (EA,K,NY,PRE,US); Kirawira *Greenway, PJ* 10608 (K); Musabi-Nyamuma *Greenway, PJ* 10620 (K);

POLYPOGON Desf.
***Polypogon monspeliensis* (L.) Desf. , Fl. Atlant. 1: 66 (1798)**
Distribution: Widespread
Specimens: Arusha Endulen *Chuwa, S* 2897 (K); Klein's camp *Greenway, PJ* 10657 (K); Endulen *Herlocker, D* H 217 (?); Klein's camp *Tanner, M* 1738 (K); **Mara** Lengusa *Ole Sayalal, P* 5 (K);

***Polypogon schimperianus* (Hochst. ex Steud.) Cope , Kew Bull. 50: 116 (1995)**
Distribution: Tropical Africa & Arabia
Specimens: Arusha Nainokanoka *Newbould, JB* 6237 (K);

PSILOLEMMA S.M.Phillips
***Psilolemma jaegeri* (Pilg.) S.M.Phillips , Kew Bull. 29: 267 (1974)**
Distribution: Tanzania, Kenya, Uganda & Democratic Republic of Congo
Specimens: Arusha Lake Magadi *Peterson, PM* 24320 (US); **Mara** Engare Nanyuki *Newbould, JB* 6314 (K); **Shinyanga** Mbalageti river *Greenway, PJ* 9028 (K);

SCHMIDTIA Steud. ex J.A.Schmidt
***Schmidtia pappophoroides* Steud. ex J.A.Schmidt. , Beitr. Fl. Cap Verd. Ins. 145 (1852)**
(Synonym: *Schmidtia bulbosa* Stapf)
Distribution: Tropical Africa & Asia
Specimens: Mara Seronera-Klein's camp *Greenway, PJ* 10301 (K);

SETARIA P.Beauv.
***Setaria homonyma* (Steud.) Chiov. , Nuovo Giorn. Bot. Ital. n.s., 26: 78 (1919)**
Distribution: Tropical Africa & Asia
Specimens: Arusha Ngorongoro Crater *Frame, GW* 83 (K,NHT); Enduleni *Njau, E* EN 663 (NHT); **Shinyanga** Moru Kopjes *Greenway, PJ* 10588 (K);

***Setaria incrassata* (Hochst.) Hack. , Hochgebirgsfl. Afrika 122 (1891)**
Distribution: Tropical Africa
Specimens: Arusha Endulen *Ellemann, L* 374 (AAU); **Mara** Serengeti *Paulo, S* 433 (K); Kirawira *Vesey-FitzGerald, LDEF* 4651 (SWRC);

***Setaria kagerensis* Mez , Notizbl. Bot. Gart. Berlin-Dahlem 7: 58 (1917)**
Distribution: Tropical Africa
Specimens: Arusha Ngorongoro Crater *Newbould, JB* 5973 (K); **Mara** Seronera *Greenway, PJ* 9946 (K,SWRC);

***Setaria longiseta* P.Beauv. , Fl. Oware 2: 81 (1819)**
Distribution: Tropical Africa
Specimens: Mara Bolgonja *Schmidt, W* 614 (SWRC); Bolgonja *Schmidt, W* 732a (SWRC);

***Setaria megaphylla* (Steud.) T.Durand & Schinz , Consp. Fl. Afric. 5: 773 (1894)**
Distribution: Tropical Africa & Arabia
Specimens: Arusha Ngorongoro Crater *Chuwa, S* 2793 (NHT); Ngorongoro *Staples, RR* 284 (K); **Mara** Bolgonja river *Greenway, PJ* 10757 (K,SWRC); Kogatende *Herlocker, D* 698 (SWRC);

***Setaria orthosticha* K.Schum. ex R.A.W.Herrm. , Beitr. Biol. Pflanzen 10: 49 (1910)**
Distribution: Tropical Africa
Specimens: Arusha Enduleni *Njau, E* EN 545 (NHT);

***Setaria poiretiana* (Schult.) Kunth , Révis. Gramin. 1: 47 (1829)**
(Synonym: *Setaria caudula* Stapf)
Distribution: Widespread
Specimens: Arusha Ngorongoro Crater *Chuwa, S* 2794 (K,NHT); Ngorongoro *Moreau* 94 (K);

***Setaria pumila* (Poir.) Roem. & Schult. , Syst. Veg. 2: 891 (1817)**
(Synonym: *Setaria pallide-fusca* (Schumach.) Stapf & C.E.Hubb)
Distribution: Widespread
Specimens: Arusha Endulen *Ellemann, L* 369 (AAU); Laetoli *Mollel, NP* 1138 (NCAA,NHT); **Mara** Seronera *Greenway, PJ* 10107 (K,SWRC); Serengeti Plains *Greenway, PJ* 10552 (SWRC); Serengeti Plains *Schmidt, W* 108 (SWRC); **Shinyanga** Beacon Area *Greenway, PJ* 10545 (K,SWRC);

Setaria sp.
Specimens: Arusha Olmoti crater *Vesey-FitzGerald, L.D.E.F.* 17 (SWRC);

***Setaria sphacelata* (Schumach.) Stapf & C.E.Hubb ex Moss , Bull. Misc. Inform. Kew 1929: 195 (1929)**
(Synonym: *Setaria sphacelata* (Schumach.) Stapf & C.E.Hubb ex Moss var. *sericea* (Stapf) Clayton ; *Setaria sphacelata* (Schumach.) Stapf & C.E.Hubb ex Moss var. *torta* (Stapf) Clayton)
Distribution: Widespread
Specimens: Arusha Empakai Crater *Frame, GW* P 5 (K,NHT); Empakai Crater *Frame, GW* 5 (SWRC); Empakai Crater *Frame, GW* 534 (SWRC); Ngorongoro Crater *Metele, P* 11 (MO,NHT); Enduleni *Njau, E* EN 551 (NHT); **Mara** Lobo Lodge *Belsky, PJ* 278 (SWRC); Ikoma *Brooks, GP* 36 (K); Seronera river *Greenway, PJ* 9968 (K,SWRC); Serengeti National Park *Mboya, E* s.n. (MO); Serengeti National Park *Mboya, E* s.n. (MO); Serengeti NP *Mboya,*

El 708 (NHT); Robanda village *Mollel, NP* 882 (NHT); Seronera river *Schmidt, W* 676 (SWRC); Bolgonja *Schmidt, W* 805 (SWRC); **Shinyanga** Lake Magadi *Greenway, PJ* 10594 (K,SWRC); Ngamuriak *Newbould, JB* 6290 (K);

***Setaria sulcata* Raddi , Agrostogr. Bras. 50 (1823)**
Distribution: Native to Central & South America, introduced in Tanzania
Specimens: Mara Bolgonja *Schmidt, W* 521 (SWRC);

***Setaria verticillata* (L.) P.Beauv. , Ess. Agrostogr. 51 (1812)**
Distribution: Widespread
Specimens: Arusha Endulen *Ellemann, L* 694 (AAU); Enja Shori *Ellemann, L* 711 (AAU); Nasiporiong *Ellemann, L* 818 (AAU); Laetoli *Mollel, NP* 1119 (NCAA,NHT); Endulen *Msuya, EZM* 16 (K); Kakesio *Newbould, JB* 5810 (K); **Mara** Serengeti Research Institute *Belsky, PJ* 136 (SWRC); Seronera *Greenway, PJ* 9980 (K,SWRC); Serengeti National Park *Mboya, E* s.n. (MO); Serengeti National Park *Mboya, E* s.n. (MO);

SNOWDENIA C.E.Hubb.
***Snowdenia petitiana* (A.Rich.) C.E.Hubb , Hooker's Icon. Pl. 37: t. 3647 (1967)**
Distribution: Eastern Africa & Yemen
Specimens: Arusha Empakai Crater *Frame, GW* 94 (NHT,SWRC); Empakai Crater *Frame, GW* 169 (SWRC);

SORGHUM Moench.
***Sorghum arundinaceum* (Desv.) Stapf , Fl. Trop. Afr. 9: 114 (1917)**
(Synonym: *Sorghum verticilliflorum* (Steud.) Stapf)
Distribution: Widespread
Specimens: Mara Seronera river *Greenway, PJ* 10321 (K); Serengeti National Park *Mboya, E* s.n. (MO);

***Sorghum versicolor* Andersson , Naturw. Reise Mossambique 2: 563 (1864)**
Distribution: Tropical Africa & Arabia
Specimens: Shinyanga Ndoha Plains *Greenway, PJ* 10075 (K,SWRC); Mbono river *Greenway, PJ* 10368 (K,SWRC); Subiti Hill *Paulo, S* 425 (K);

SPOROBOLUS R.Br.
***Sporobolus africanus* (Poir.) Robyns & Tournay , Bull. Jard. Bot. État 25: 242 (1955)**
(Synonym: *Sporobolus capensis* (P.Beauv.) Kunth)
Distribution: Widespread
Specimens: Arusha Ngorongoro Crater *Greenway, PJ* 3367 (K); Ngoitoktok springs *Greenway, PJ* 12597 (K); Ngorongoro Crater *Newbould, JB* 5694 (K); Ngorongoro Crater *Pole Evans, IB* 974 (K); **Mara** Togoro Plains *Braun, HMH* 210 (K);

***Sporobolus agrostoides* Chiov. , Annuario Reale Ist. Bot. Roma 7: 67 (1897)**
Distribution: Tropical Africa
Specimens: Mara Mgungu river *Braun, HMH* 115 (K);

***Sporobolus confinis* (Steud.) Chiov. , Annuario Reale Ist. Bot. Roma 8: 341 (1908)**
(Synonym: *Sporobolus affinis* A.Rich. ; *Sporobolus phyllotrichus* Hochst.)
Distribution: Eastern Africa & Yemen
Specimens: Arusha Ngorongoro Crater *Braun, HMH* 160 (K); Ngorongoro Crater *Braun, HMH* 167 (K); Ngorongoro Crater *Greenway, PJ* 11844 (K); Ngorongoro Crater *Heady, AF* 1646 (K); Ngorongoro Crater *Peterson, PM* 24303 (US);

***Sporobolus consimilis* Fresen. , Mus. Senckenberg. 2: 140 (1837)**
Distribution: Tropical Africa & Arabia
Specimens: Arusha Ololgumi *Ellemann, L* 544 (AAU); Olduvai Gorge *Ellemann, L* 804 (AAU); Olbili *Ellemann, L* 1061 (AAU); Ngorongoro Crater *Newbould, JB* 6173 (K); Olduvai *Raynal, J* 19301 (K); **Mara** Seronera river *Greenway, PJ* 10352 (K); **Shinyanga** Moru Kopjes *Greenway, PJ* 9154 (K); Lake Magadi *Greenway, PJ* 10045 (K);

***Sporobolus cordofanus* (Hochst. ex Steud.) Hérincq ex Coss. , Bull. Soc. Bot. France 36: 253 (1889)**
(Synonym: *Sporobolus humifusus* (Kunth) Kunth var. *cordofanus* (Hochst. ex Steud.) R.L.Massey)
Distribution: Tropical Africa
Specimens: Arusha Gol Kopjes *Braun, HMH* 223 (K); Olkarien *Newbould, JB* 6405 (K); Lake Magadi *Peterson, PM* 24313 (US); **Mara** Seronera *Greenway, PJ* 9985 (K); Grumechem *Greenway, PJ* 10329 (K); Seronera river *Paulo, S* 393 (K);

***Sporobolus coromandelianus* (Retz.) Kunth , Révis. Gramin. 1: 68 (1829)**
Distribution: Widespread
Specimens: Shinyanga Naabi Hill Gate *Peterson, PM* 24269 (US);

***Sporobolus discosporus* Nees , Fl. Afr. Austral. Ill.: 158 (1841)**
Distribution: Eastern & Southern Africa
Specimens: Arusha Soitayai *Paulo, S* 363 (EA,K);

***Sporobolus festivus* Hochst. ex A.Rich. , Tent. Fl. Abyss. 2: 398 (1850)**
Distribution: Tropical Africa & Asia
Specimens: Mara Ikoma *Brooks, GP* 41 (K); Seronera *Greenway, PJ* 9842 (K); Seronera *Greenway, PJ* 9906 (K); Serengeti National Park *Mboya, E* s.n. (MO); Engare Nanyuki *Newbould, JB* 6183 (K); Mbuzi Mare camp *Peterson, PM* 24289 (US); **Shinyanga** Naabi Hill Gate *Peterson, PM* 24256 (US);

***Sporobolus fimbriatus* (Nees ex Trin.) Nees , Fl. Afr. Austral. Ill. 156 (1841)**
(Synonym: *Sporobolus fimbriatus* (Nees ex Trin.) Nees var. *latifolius* Stent)
Distribution: Tropical Africa
Specimens: Arusha Ngorongoro Crater *Chuwa, S* 5729 (NHT); Gol Kopjes *Greenway, PJ* 10735 (K); **Mara** Seronera *Bogdan* 1320 (?); Ndabaka Plains *Greenway, PJ* 9092 (K); Seronera *Greenway, PJ* 9852 (K); Seronera dam site *Greenway, PJ* 9982 (K); Seronera *Greenway, PJ* 10189 (K); Serengeti *Greenway, PJ* 10487 (K); Robanda village *Mollel, NP* 846 (NHT); Seronera *Newbould, JB* 6189 (K); Engare Nanyuki *Newbould, JB* 6298 (K); East Serengeti *Paulo, S* 374 (K); Mbuzi Mare camp *Peterson, PM* 24280 (US); **Shinyanga** Naabi Hill *Greenway, PJ* 10737 (K); Lake Magadi *Leippert, H* 5598 (K); Naabi *Paulo, S* 370 (K); Naabi Hill Gate *Peterson, PM* 24258 (US); Naabi Hill Gate *Peterson, PM* 24260 (US);

***Sporobolus ioclados* (Trin.) Nees , Fl. Afr. Austral. Ill.: 161 (1841)**
(Synonym: *Sporobolus kentrophyllum* (K.Schum.) Clayton ; *Sporobolus laetevirens* Coss. ; *Sporobolus marginatus* Hochst. ex A.Rich. ; *Sporobolus rangei* Pilg. ; *Sporobolus verdcourtii* Napper)
Distribution: Tropical Africa, Arabia & Asia
Specimens: Arusha Olbalbal swamp *Ellemann, L* 1095 (AAU); Oldonyo-o-ogol *Ellemann, L* 1118 (AAU); Olduvai Headwaters *Greenway, PJ* 9107 (K); Ol Doinyo Lengai *Greenway, PJ* 11352 (K); Ang'ata Salei *Newbould, JB* 6568 (K); Ang'ata Salei *Newbould, JB* 6572 (K); Ang'ata Salei *Oteke, J* 236 (K); Olbalbal *Paulo, S* 327 (K); Ngorongoro Crater *Paulo, S* 332 (K); Lemuta hill *Paulo, S* 349 (K); Salei Plain *Paulo, S* 406 (K); Salei Plain *Paulo, S* 407 (K); Lake Magadi *Phillipson, PB* s.n. (MO); **Mara** Banagi Hill *Brooks, GP* 64 (K); Banagi Hill *Brooks, GP* 97 (K); Seronera *Greenway, PJ* 9843 (K); Seronera *Greenway, PJ* 9904 (K); Seronera *Greenway, PJ* 10101 (K); Seronera *Greenway, PJ* 10185 (K); Seronera Lodge *Greenway, PJ* 10191 (K); Engare Nanyuki *Greenway, PJ* 10480 (K); Seronera *Greenway, PJ* 10652 (K); Musabi plains *Mboya, E* s.n. (MO); Serengeti National Park *Mboya, E* s.n. (MO); Serengeti *Paulo, S* 345 (K); Endarbark *Paulo, S* 439 (K); **Shinyanga** Naabi Hill *Greenway, PJ* 10156 (K); Lake Magadi *Greenway, PJ* 10506 (K); Lake Magadi *Leippert, H* 5597 (K); Moru Kopjes *Paulo, S* 391 (K);

***Sporobolus macranthelus* Chiov. , Fl. Somala 2: 452. (1932)**
(Synonym: *Sporobolus greenwayi* Napper)
Distribution: Tropical Africa
Specimens: Arusha Kakesio River *Chuwa, S* 2631 (K); Kimba *Greenway, PJ* 12526 (K); Ngorongoro Crater *Vesey-FitzGerald, LDEF* 2104 (K);

***Sporobolus nervosus* Hochst. , Flora 38: 202 (1855)**
Distribution: Eastern & Southern Africa & Arabia
Specimens: Arusha Olduvai Gorge *Chuwa, S* 2636 (K); Olbalbal *Paulo, S* 402 (K);

***Sporobolus panicoides* A.Rich. , Tent. Fl. Abyss. 2: 399 (1850)**
Distribution: Eastern & Southern Africa & Yemen
Specimens: Arusha Endulen *Ellemann, L* 394 (AAU); Kakesio *Paulo, S* 428 (K);

***Sporobolus pellucidus* Hochst. , Flora 38: 201 (1855)**
Distribution: Tropical Africa & Arabia
Specimens: Arusha Endoinyo Emboleh *Newbould, JB* 6375 (K); **Mara** Togoro Plains *Braun, HMH* 211 (K); Seronera *Greenway, PJ* 9846 (K); Campi ya Mawi *Greenway, PJ* 10943 (K);

***Sporobolus pyramidalis* P.Beauv. , Fl. Oware 2: 36 (1816)**
Distribution: Widespread
Specimens: Arusha Kakesio plain *Chuwa, S* 2626 (K); **Mara** Seronera *Greenway, PJ* 10068 (K); Tabora *Greenway, PJ* 10389 (K); Kirawira Plains *Greenway, PJ* 13312 (K); Serengeti Research Centre *Mboya, E* s.n. (MO); Lobo Lodge *Peterson, PM* 24298 (US);

***Sporobolus rigidifolius* (Trin.) Mez ex Veldkamp , Kew Bull. 45: 582 (1990)**
Distribution: Tropical Africa
Specimens: Arusha Lake Magadi *Peterson, PM* 24317 (US);

***Sporobolus sanguineus* Rendle , Cat. Afr. Pl. 2: 209 (1899)**
(Synonym: *Sporobolus homblei* De Wild.)
Distribution: Tropical Africa
Specimens: Arusha Lake Magadi *Braun, HMH* 165 (K); Hippo pool - Ngorongoro *Greenway, PJ* 12594 (K); Ngorongoro Crater *Newbould, JB* 6170 (K); Ngorongoro Crater *Vesey-FitzGerald, LDEF* 2105 (K); **Mara** Barelamangi salt lick *Greenway, PJ* 10138 (K); Engare Nanyuki *Newbould, JB* 6297 (K);

Sporobolus sp.
Specimens: Arusha Olduvai Gorge *Ellemann, L.* 805 (AAU); Lake Madagi *Phillipson, P.B.* s.n. (MO); Lake Madagi *Phillipson, P.B.* s.n. (MO);

***Sporobolus spicatus* (Vahl) Kunth , Révis. Gramin. 1: 67 (1829)**
Distribution: Widespread
Specimens: Arusha Oldonyo Lengai Mt. *Clair-Thompson, GN* 286 (K); Gol Kopjes *Greenway, PJ* 10525 (K); Ol Doinyo Lengai *Greenway, PJ* 11350 (K); Ngorongoro Crater *Paulo, S* 330 (K); Lake Magadi *Peterson, PM* 24315 (US); Olduvai *Raynal, J* 19298 (K); Oldonyo Lengai Mt. *Richards, M* 25538 (K); **Mara** Seronera dam site *Greenway, PJ* 9984 (K); Seronera *Greenway, PJ* 10151 (K); **Shinyanga** Naabi Hill *Greenway, PJ* 10158 (K);

***Sporobolus stapfianus* Gand. , Bull. Soc. Bot. France 66: 302 (1919 publ. 1920)**
Distribution: Tropical Africa
Specimens: Arusha Ngorongoro *Braun, HMH* 214 (K);

TETRAPOGON Desf.
Tetrapogon roxburghiana (Schult.) P.M.Peterson, Taxon 64(3): 460 (2015)
(Synonym: *Chloris roxburghiana* Schult.)
Distribution: Tropical Africa & Asia
Specimens: Mara Serengeti Research Institute *Belsky, PJ* 168 (SWRC); Banagi Hill *Brooks, GP* 59 (K); Seronera *Greenway, PJ* 9851 (K,SWRC); Serengeti Research Centre *Mboya, E* s.n. (MO); Serengeti Research Centre *Mboya, EI* 747 (NHT); Seronera *Paulo, S* 353 (K); Serengeti Research Institute *Unknown* s.n. (SWRC);

THEMEDA Forssk.
Themeda sp.
Specimens: Arusha Irmisigiyo *Ellemann, L.* 943 (AAU);

***Themeda triandra* Forssk. , Fl. Aegypt.-Arab. 178 (1775)**
Distribution: Widespread
Specimens: Arusha Empakai Crater *Clair-Thompson, GN* 413 (K); Endulen *Ellemann, L* 699 (AAU); Sendui *Ellemann, L* 722 (AAU); Oloronyo *Ellemann, L* 772 (AAU); Olmekeke *Ellemann, L* 918 (AAU); Oltebesi *Ellemann,*

L 932 (AAU); Ngorongoro Crater *Metele, P* 10 (MO,NHT); Ngorongoro *Moreau* 95 (K); Ngorongoro Crater *Paulo, S* 316 (K); Ngorongoro Crater *Peterson, PM* 24310 (US); Ngorongoro Crater *Pole Evans, IB* 944 (K); Ngorongoro Crater *Pole Evans, IB* 971 (K); **Mara** Seronera *Greenway, PJ* 9835 (K); Serengeti National Park *Mboya, E* s.n. (MO); Robanda village *Mollel, NP* 819 C (NHT); Seronera airdrome *Paulo, S* 293 (K); Lake Lagarja *Raynal, J* 19343 (K); **Shinyanga** Lake Magadi *Brown, ES* 1045 L (EA); Naabi Hill Gate *Peterson, PM* 24273 (US);

TRACHYPOGON Nees
***Trachypogon spicatus* (L.f.) Kuntze , Revis. Gen. Pl. 2: 794 (1891)**
Distribution: Tropical Africa & Americas
Specimens: Mara Seronera-Karawera *Greenway, PJ* 10088 (K); Seronera-Klein's camp *Greenway, PJ* 10200 (K);

TRAGUS Haller
***Tragus berteronianus* Schult. , Mant. 2: 205 (1824)**
Distribution: Widespread
Specimens: Arusha Salei Plain *Paulo, S* 408 (K); **Mara** Seronera *Greenway, PJ* 9864 (K); Serengeti Research Centre *Mboya, E* s.n. (MO); Robanda village *Mollel, NP* 817 (NHT);

Tragus sp.
Specimens: Arusha Lake Madagi *Peterson, P.M.* 24314 (US);

TRICHOLAENA Schrad.
***Tricholaena teneriffae* (L.f.) Link , Handbuch 1: 91 (1829)**
Distribution: Africa, Asia & Europe
Specimens: Arusha Ngorongoro Crater *Heady, HF* 1631 (K); Lemuta *Newbould, JB* 6179 (K); Oldiang'arangar *Newbould, JB* 6266 (K); **Mara** Serengeti Central Plains *Greenway, PJ* 10697 (K); Seronera river *Greenway, PJ* 10718 (K); Tutiahi river *Greenway, PJ* 10904 (K);

TRICHONEURA Andersson
***Trichoneura ciliata* (Peter) S.M.Phillips , Kew Bull. 29: 270 (1974)**
Distribution: Tanzania, Ethiopia & Kenya
Specimens: Mara Seronera-Klein's camp *Greenway, PJ* 10196 (EA,K);

TRIPOGON Roem. & Schult.
***Tripogon minimus (*A.Rich.) Hochst. ex Steud. , Syn. Pl. Glumac. 1: 301 (1854)**
Distribution: Tropical Africa
Specimens: Mara Seronera *Braun, H* 322 (K); Seronera *Braun, HMH* 322 (EA);

UROCHLOA P.Beauv.
***Urochloa brachyura* (Hack.) Stapf , Fl. Trop. Afr. 9: 592 (1920)**
(Synonym: *Urochloa geniculata* C.E.Hubb)
Distribution: Eastern & Southern Africa
Specimens: Mara Banagi Hill *Braun, H* 198 (K); Togoro Plains *Greenway, PJ* 13344 (K); Seronera Lodge *Mboya, E* s.n. (MO); Serengeti NP *Mollel, NP* 934 (NHT);

***Urochloa panicoides* P.Beauv. , Ess. Agrostogr. 52 (1812)**
Distribution: Widespread
Specimens: Arusha Endulen *Newbould, JB* 5512 (K);
